# Development of Highly Potent, G-Protein Pathway Biased, Selective, and Orally Bioavailable GPR84 Agonists

**DOI:** 10.1021/acs.jmedchem.3c00951

**Published:** 2023-12-26

**Authors:** Pinqi Wang, Arun Raja, Vincent B. Luscombe, Carole J. R. Bataille, Daniel Lucy, Vanessa V. Rogga, David R. Greaves, Angela J. Russell

**Affiliations:** † Department of Chemistry, University of Oxford, Mansfield Road, Oxford OX1 3TA, U.K.; ‡ Department of Pharmacology, University of Oxford, Mansfield Road, Oxford OX1 3QT, U.K.; § Sir William Dunn School of Pathology, University of Oxford, South Parks Road, Oxford OX1 3RE, U.K.

## Abstract

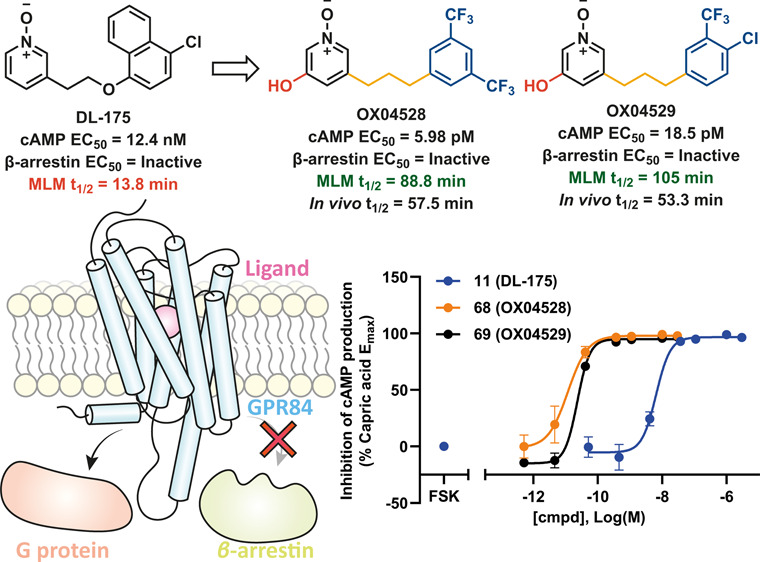

Orphan G-protein-coupled receptor 84 (GPR84) is a receptor that has been linked to cancer, inflammatory, and fibrotic diseases. We have reported DL-175 as a biased agonist at GPR84 which showed differential signaling via G_αi_/cAMP and β-arrestin, but which is rapidly metabolized. Herein, we describe an optimization of DL-175 through a systematic structure–activity relationship (SAR) analysis. This reveals that the replacement of the naphthalene group improved metabolic stability and the addition of a 5-hydroxy substituent to the pyridine *N*-oxide group, yielding compounds **68** (OX04528) and **69** (OX04529), enhanced the potency for cAMP signaling by 3 orders of magnitude to low picomolar values. Neither compound showed detectable effects on β-arrestin recruitment up to 80 μM. Thus, the new GPR84 agonists **68** and **69** displayed excellent potency, high G-protein signaling bias, and an appropriate *in vivo* pharmacokinetic profile that will allow investigation of GPR84 biased agonist activity *in vivo*.

## Introduction

**Figure 1:**
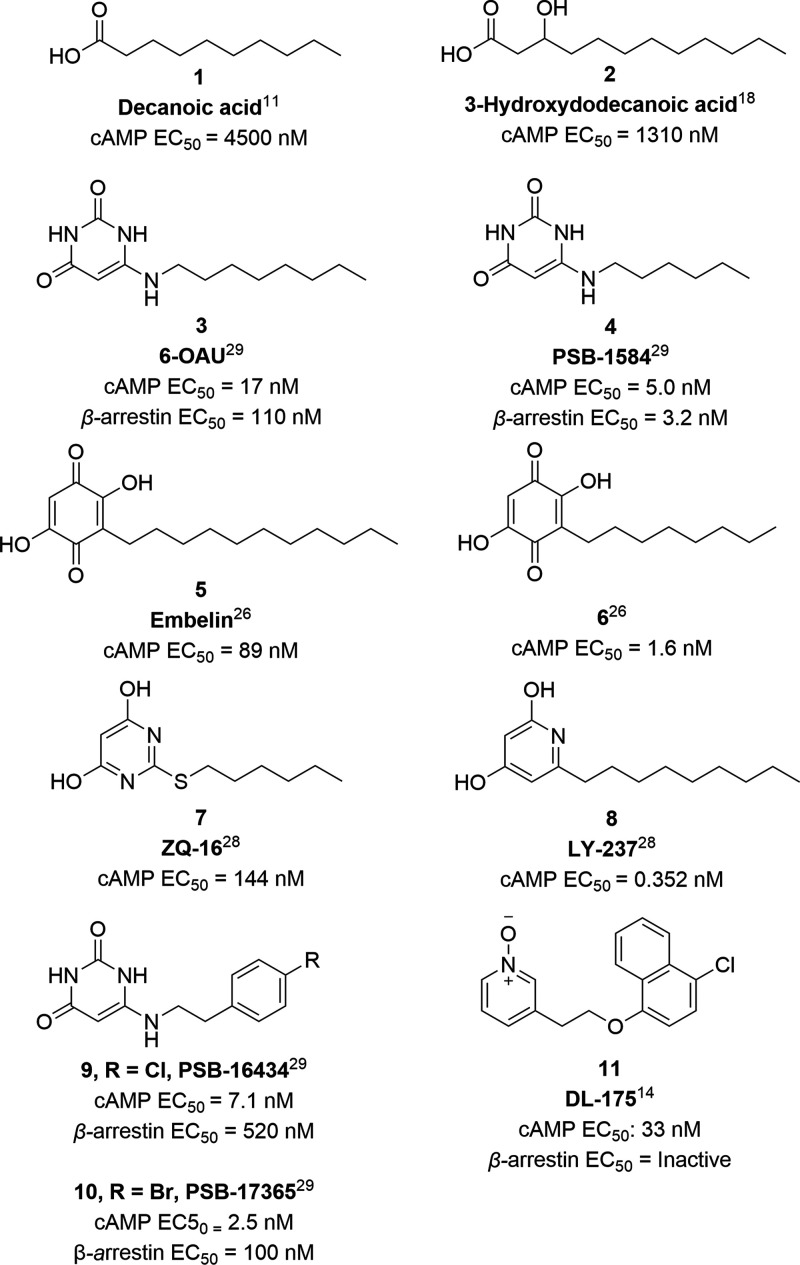
Structures of GPR84 natural and synthetic agonists.

G-protein-coupled receptors (GPCRs) are the largest membrane protein family encoded by the human genome, regulating numerous diverse physiological processes.^1,2^ To service this broad role of cellular communication, the GPCR superfamily responds to a wide range of ligands.^3^ Among these, free fatty acids (FFAs) are essential nutrients having diverse effects on numerous biological process related to cardiovascular health, metabolism, and inflammation.^4^ The family of GPCRs using FFAs as endogenous ligands to regulate their functions are classified as free fatty acid receptors (FFARs), including medium- to long-chain FFARs GPR40 (FFA1) and GPR120 (FFA4) and short-chain FFARs GPR43 (FFA2) and GPR41 (FFA3).^5−7^ G-protein-coupled receptor 84 (GPR84) is one of the rhodopsin-like class A GPCRs and a putative fifth fatty acid receptor, which was discovered by a comprehensive expressed sequence tag database search method and then cloned and characterized from GPR84-encoded human peripheral blood neutrophils.^8,9^ GPR84 is expressed predominantly in myeloid cells, including monocytes, macrophages, neutrophils, eosinophils, phorbol ester-activated peripheral blood mononuclear cells, and microglia in the central nervous system.^10^ Saturated medium-chain fatty acids (MCFAs) with chain length 9–14 are agonists of GPR84 that engage G_αi_ signaling to reduce cyclic adenosine monophosphate (cAMP) production by inhibiting adenylate cyclase.^11^ However, the most potent MCFA, decanoic acid **1** (Figure 1), as well as the MCFA oxidized metabolite, 3-hydroxydodecanoic acid **2**, show weak micromolar potency and fail to recruit β-arrestin, consistent with the view that GPR84 remains an orphan receptor.^11−19^

*GPR84* mRNA expression in leukocytes and adipocytes can be significantly upregulated by vitamin D^20^ and inflammatory stimuli like lipopolysaccharide (LPS)^21^ and tumor necrosis factor alpha (TNFα),^22^ as well as chronic low-grade inflammation.^16^ Activation of GPR84 by synthetic agonists *in vitro* results in enhanced phagocytosis, immune cell migration, and increased secretion of cytokines, chemokines, and other inflammatory mediators.^11,16,23,24^ GPR84 agonists have been shown to mediate enhanced phagocytosis of adipocyte plasma membrane-associated protein (APMAP)-deficient cancer cells, suppress lipotoxicity-induced macrophage over-activation, trigger increased bacterial adhesion, show anti-atherosclerotic effects, and play a role in the regulation of mitochondrial metabolism, suggesting that the activation of GPR84 may be beneficial for cancer, bacteria killing, and metabolic dysfunction.^16,24−27^

As the endogenous ligand of GPR84 was still unknown, a compound library was screened to discover the first synthetic agonist, 6-octylaminouracil **3** (6-OAU, Figure 1).^23^ It has a fatty acid-mimetic structure with a polar headgroup and a lipophilic chain, which has been a commonly used positive control that can both activate G protein and recruit β-arrestin.^14,16,19,28,29^ Its derivative **4** (PSB-1584, Figure 1) shows an enhanced activity in both G-protein and β-arrestin pathways.^29^ Embelin **5** (Figure 1) is a natural product that can activate GPR84.^15,17,26,30^ It has been found to have analgesic, antitumor, anti-inflammatory, antioxidant, and wound-healing activities.^31−33^ With minor modification on the aliphatic chain, **6** (Figure 1) showed a higher potency and better selectivity toward the activation of GPR84.^26^ Based on a high-throughput screening with 160,000 compounds from the Chinese National Compound Library, **7** (ZQ-16, Figure 1) was found to be a more potent agonist than 6-OAU.^14,17,19,28^ Following a structure–activity relationship (SAR) study, **8** (LY-237, Figure 1) was found to be more potent than **7** and **3** in calcium ion and cAMP assays in hGPR84-transfected Chinese hamster ovary (CHO) cells.^14,28^ Uracil derivatives **9** and **10** (PSB-16434 and PSB-17365, Figure 1) were reported with enhanced potency at GPR84 and G-protein signaling bias.^29^ Recently, the cryo-EM structures of **3** and **8** bound to GPR84 have been reported, revealing the structural basis of GPR84 activation.^34,35^

At many GPCRs, signaling events mediated by G proteins and β-arrestins have been shown to have distinct biomedical and physiological actions from each other, which make them capable of directing functional effects toward a specific pathway.^36^ The development of biased agonists has become an increasingly active area of research, as it may identify compounds with increased efficacy and reduced on-target side effects.^37,38^

Compound **11** (DL-175, Figure 1) was reported from our group following a virtual screen using a quantitative structure–activity relationship (QSAR) model followed by a preliminary SAR analysis.^14^ It shows a comparable potency to **3** in assays monitoring inhibition of cAMP accumulation.^14,39^ However, it shows no measurable effect on β-arrestin recruitment up to the highest concentration tested (60 μM), indicating a significant bias toward G-protein signaling. Compared to **3**, compound **11** fails to promote chemotaxis of M1-polarized U937 macrophages, demonstrating that GPR84-driven effects on phagocytosis and chemotaxis can be separated, and a biased agonist inducing less chemotaxis may potentially reduce side effects *in vivo*. Additionally, while **7** induces phosphorylation of GPR84 on two threonine residues (Thr263/Thr264), **11** does not, and introducing an Arg172Ala mutation in the receptor does not affect the activity of **11**, while it abolishes the activity of **7**, potentially suggesting they have different binding modes.^40^ However, **11** is rapidly metabolized when exposed to whole mouse hepatocytes (*t*_1/2_ < 10 min), precluding its use as an *in vivo* tool compound. As the implications of biased signaling at GPR84 *in vitro* and *in vivo* remain to be fully understood, biased agonists with a suitable pharmacokinetic (PK) profile for *in vivo* studies are needed.

Herein, we set out to optimize **11** into a suitable *in vivo* tool compound for the further investigation of GPR84 pathophysiology in preclinical models. We conducted systematic SAR studies on **11**, leading to the development of exceptionally potent, highly G-protein signaling biased agonists **68** (OX04528) and **69** (OX04529) at GPR84 with appropriate selectivities and absorption, distribution, metabolism, and excretion (ADME) profiles for progression to *in vivo* studies.

## Results and Discussion

### Structure–Activity Relationship

In order to inform our strategy to optimize the chemistry around the GPR84 biased agonist **11** toward a molecule suitable for *in vivo* studies, we first sought to develop an understanding of its metabolic liabilities. Incubation of **11** with mouse liver microsomes (MLMs) and whole hepatocytes showed rapid metabolism with *t*_1/2_ of 13.8 min and <10 min^14^ respectively. Incubation of **11** with whole-cell murine hepatocytes was performed for 60 min, and the resulting metabolites were characterized using liquid chromatography–tandem mass spectrometry (LC-MS/MS; Table S1): 76% of the metabolites detected showed monooxidation, of which 8% formed the glucouronide conjugate (Table S1, M3); 12% of the metabolites identified were dihydroxylated (Table S1, M2); and the remaining 12% were unidentified metabolites.

**Figure 2:**
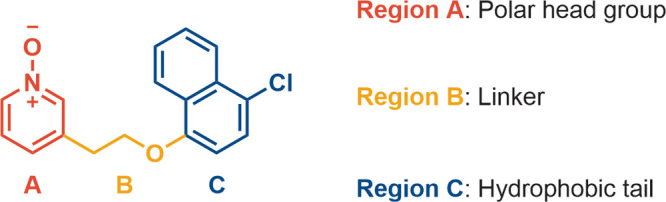
SAR investigation strategy.

Given the metabolite profile, it was predicted that the oxidation predominantly occurred on the naphthalene moiety, as the oxidative metabolism of naphthalenes to naphthols and dihydrodiols is well-documented by ourselves and others.^41−43^ To systematically interrogate the SAR and develop an understanding of the metabolic liabilities of **11**, the structure was divided into three regions (Figure 2), i.e., the hydrophobic tail (Region C), linker (Region B), and polar headgroup (Region A), which will be focused on separately.

#### SAR Investigation of Region C

We initially performed modification on region C to address first the issue of metabolic instability and gain deeper insights into the SARs (Table 1). The potency of the synthesized agonists was measured by the inhibition of forskolin-induced (FSK-induced) cAMP production in CHO-hGPR84 cells (EC_50_ and pEC_50_ ± SEM). To maximize the minimally acceptable lipophilicity per unit of *in vitro* potency, lipophilic ligand efficienc (LLE) was also monitored.^44^ Removal or replacement of the 4-chloro substitution with halogens, electron-donating groups, or hydrogen (**12**–**15**) illustrated the preference for halogens. The decrease of 15- to 118-fold potency from **16** and **17** (tested as a racemic mixture) indicated that planar and aromatic fragments were preferred. The reduced potency of **18** (tested as a racemic mixture) compared to **17** suggested an optimal vector to define placement of the second aromatic ring.

**Table 1:**
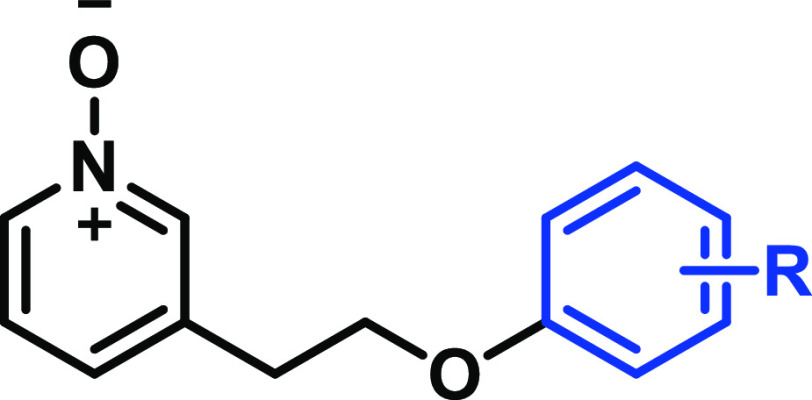

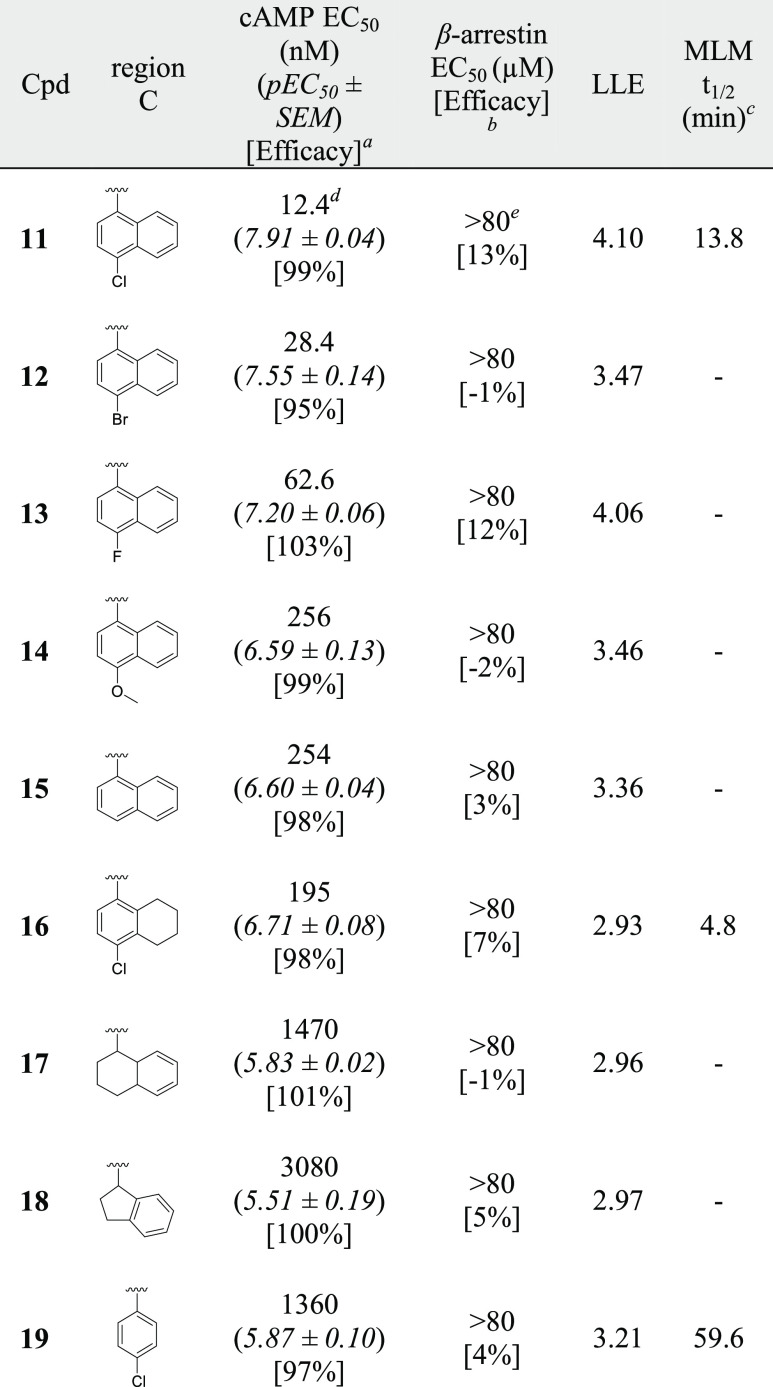
Structure–Activity Relationship of Region C

a Inhibition of FSK (25 μM)-induced cAMP production in CHO-hGPR84 cells. Efficacy is expressed as a percentage of the maximum response observed, normalized to the maximum response for capric acid (100 μM). *n* = 3 if not specified.

b Recruitment of β-arrestin in CHO-β-arrestin-hGPR84 cells. *n* = 1 if no detectable activation up to 80 μM. Efficacy at 80 μM was normalized to the maximum response of 6-OAU (80 μM).

c Mouse liver microsomes.

d *n* = 34.

e *n* = 3.

The *para*-chlorophenyl-substituted derivative **19** was prepared to investigate the effect of replacement of the naphthyl group and showed a 110-fold decrease in potency compared to **11**. Although they showed reduced potency compared to **11**, **16** and **19** were still profiled in a metabolic stability study. The rapid turnover of **16** (MLM *t*_1/2_ = 4.8 min) could be attributed to the metabolism of the tetralin group, which is well documented.^45,46^ Encouragingly, **19** was found to have enhanced metabolic stability (MLM *t*_1/2_ = 59.6 min) compared to **11**, consistent with our hypothesis that the major site of metabolism was the naphthyl group. None of the new analogues showed higher LLE than **11**, due to the decreased potency and the absence of additional hydrophilic groups. All active compounds were found to be full agonists in cAMP assays compared to the reference ligand (capric acid). All compounds tested were inactive in β-arrestin assays (Table 1), demonstrating the consistent G-protein pathway bias of derivatives of **11** at GPR84. The identification of **19** prompted the further exploration of region C with substituted arenes in order to maintain metabolic stability but enhance potency.

To further investigate the phenyl substitution at region C, *para*-substituted derivatives were then designed and synthesized (Table 2). The potency of the *para*-halo-substituted derivatives **20** and **21** being comparable to that of **19** is consistent with the naphthalene series (Table 1). The *para*-substituted **22** and **23** showed a decrease of potency, which could be attributed to the unfavorable hydrogen bond acceptor (HBA) from cyano^47^ and nitro groups,^48^ the reduced arene electron density, or the decrease of hydrophobicity.^49^ The *ortho*-substituted derivatives **24**–**29** showed reduced or diminished potency, which terminated further exploration at the *ortho* position. In contrast to **25** and **31**, the higher potency of **29** and **30** further emphasized the significance of a *para-*substituted halogen. The investigation on the *meta-*substituted derivatives showed the possibility of enhanced potency. The *meta*-iodo-substituted **32** showed a 20-fold increase in potency with enhanced LLE, indicating that appropriate *meta* substitution could increase potency. The decrease of potency with a variety of *meta* substitutions (**33–40**) gave insights into a relationship between steric effects and activity. By changing the halogen from iodo **32** to sterically less hindered bromo **33**, chloro **34**, and fluoro **35**, the potency incrementally decreased. With the more hindered 3-phenyl **36**, the potency was greatly reduced, while the smaller 3-methyl, 3-methoxy, and 3-ethyl compounds showed a rank order ethyl > methoxy > methyl. The *meta*-nitro-substituted **38** showed no activity. Intriguingly, the 3-cyclopropyl-substituted **41** showed a comparable potency but slightly decreased LLE relative to **32**, suggesting *meta* substitution with an appropriate balance between steric demand and lipophilicity is required. However, in a MLM study, **41** was found to be quickly metabolized (MLM *t*_1/2_ = 7.2 min), which may result from the precedented oxidation of the cyclopropyl substituent.^50^ Gratifyingly, the 4-Cl-3-CF_3_-substituted **42** (EC_50_ = 898 nM) and the 3,5-diCF_3_-substituted **43** (EC_50_ = 776 nM) showed enhanced potency compared to **19**, with **42** showing good metabolic stability (MLM *t*_1/2_ = 49.9 min). All active compounds were found to be full agonists in cAMP assays compared to a reference ligand (capric acid) and showed no detectable activity in recruiting β-arrestin (Table S2), in line with our previous observations with **11**. The diminished potency of all phenyl analogues compared to **11** suggested an exploration of regions A and B to find a balance between potency and stability for progressing the project.

**Table 2:**
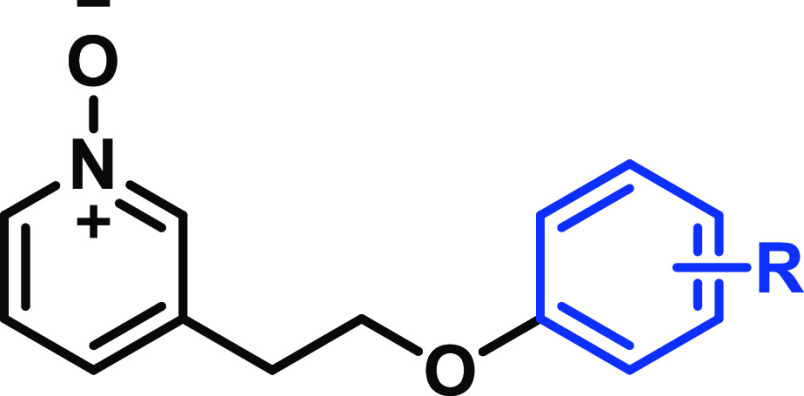

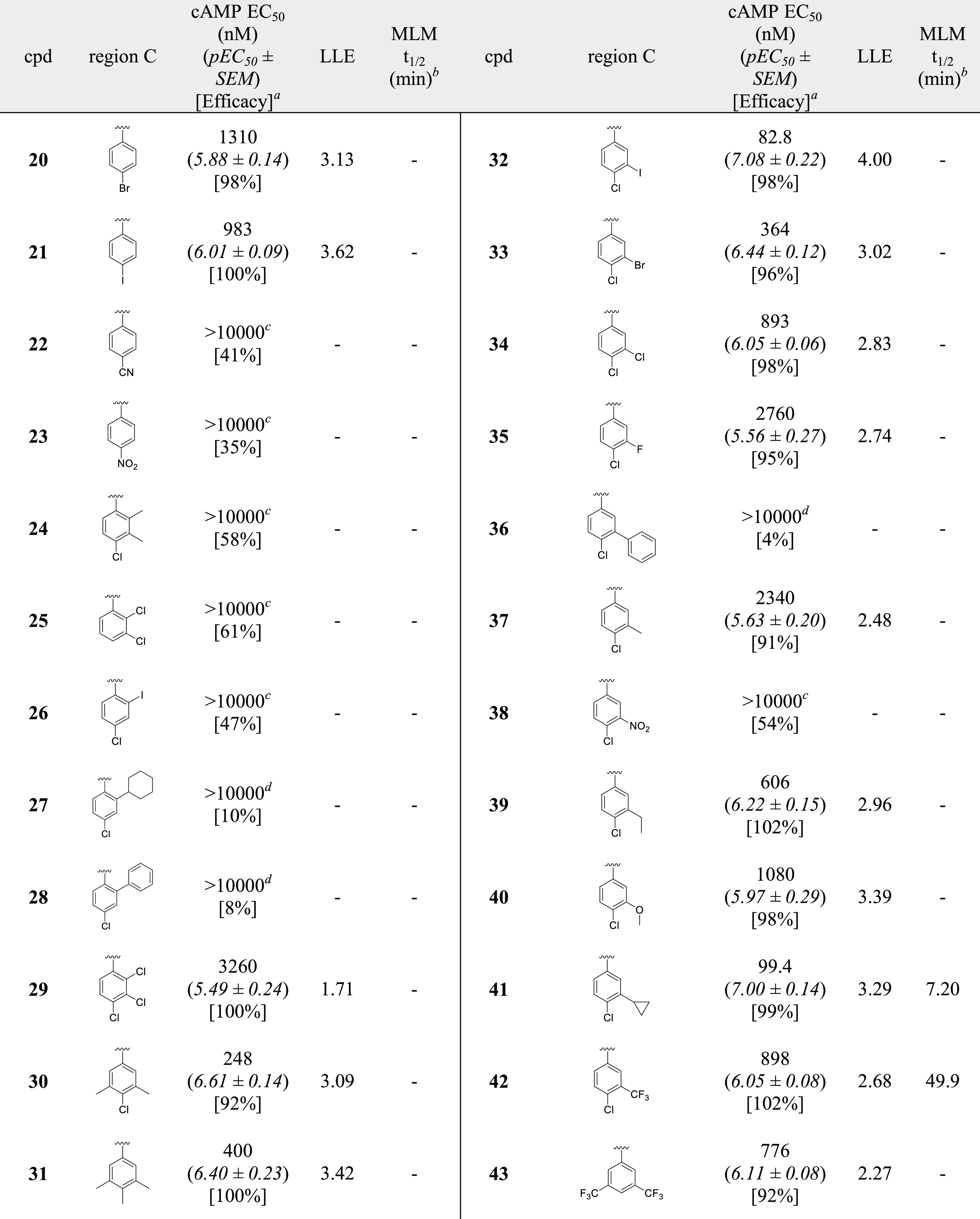
Further Structure–Activity Relationship of Region C

a Inhibition of FSK (25 μM)-induced cAMP production in CHO-hGPR84 cells. Efficacy is expressed as a percentage of the maximum response observed, normalized to the maximum response for capric acid (100 μM). *n* = 3 if not specified. Efficacy at 30 μM is shown for compounds with potency >10 μM.

b Mouse liver microsomes.

c *n* = 2.

d *n* = 1.

#### SAR Investigation of Region B

We next investigated the linker moiety (region B) to explore the effects of chain length, the introduction/removal of heteroatoms, and the tolerance of a selection of functional groups (Table 3). From an analysis of **44**, **45**, and **11**, it was evident that the length of the linker was optimal with 3 atoms. The comparable potencies of **46** and **11** illustrated that an ether linker was not required and could be replaced with an alkyl linker. However, the decreased LLE could become a potential problem with the alkyl linker. **47** showed a 20-fold decrease of potency compared to **15** and **46**, indicating that introduction of a carbonyl at X compromised potency. The regioisomeric ether linker in **48** resulted in a 2-fold decrease in potency compared to **19**. Interestingly, **49** with an alkyl linker showed a 4-fold increase in potency compared to **19**, which is not consistent with the naphthalene series **46** but showed the potential of replacing naphthalene while maintaining the potency and LLE. Further addition of carbonyl at position X in **49** yielded **50** with a 2-fold potency decrease. By changing position Y on the *para*-chlorophenyl derivatives (Table 3C), sulfone **51** and hydroxy **52** showed diminished potency, and fluoro and carbonyl decreased the potency by more than 10-fold compared to **19**. Incorporating an amino group at position X of the linker in **55** led to a comparable potency and higher LLE than **19** but a lower potency than **49**. All active compounds were found to be full agonists in cAMP assays compared to a reference ligand (capric acid) and showed no detectable activity in recruiting β-arrestin.

**Table 3:**
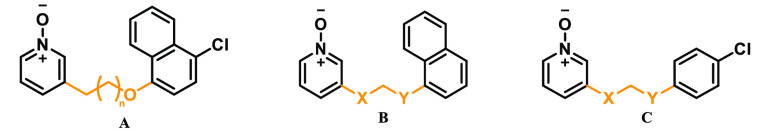
Structure–Activity Relationship of Region B

				cAMP EC_50_ (nM)	β-arrestin	
				(*pEC*_50_ *± SEM*)	EC_50_ (μM)	
cpd	*n*	X	Y	[Efficacy]^a^	[Efficacy]^b^	LLE
**A**						
**44**	0	–	–	497	>80	2.93
				(*6.30 ± 0.08*)	[−6%]	
				[99%]		
**11**	1	–	–	12.4^c^	>80^e^	4.10
				(*7.91 ± 0.04*)	[13%]	
				[99%]		
**45**	2	–	–	370	>80	2.19
				(*6.43 ± 0.12*)	[15%]	
				[99%]		

**B**						
**15**	–	CH_2_	O	254	>80	3.36
				(*6.60 ± 0.04*)	[3%]	
				[98%]		
**46**	–	CH_2_	CH_2_	257	>80	2.51
				(*6.59 ± 0.05*)	[0%]	
				[98%]		
**47**	–	C=O	CH_2_	5300	>80	2.00
				(*5.28 ± 0.02*)	[−12%]	
				[94%]		

**C**						
**19**	–	CH_2_	O	1360	>80	3.21
				(*5.87 ± 0.10*)	[4%]	
				[97%]		
**48**	–	O	CH_2_	2180	>80	2.86
				(*5.66 ± 0.10*)	[4%]	
				[93%]		
**49**	–	CH_2_	CH_2_	325	>80	2.85
				(*6.49 ± 0.13*)	[3%]	
				[94%]		
**50**	–	C=O	CH_2_	665	>80	3.39
				(*6.18 ± 0.12*)	[−3%]	
				[94%]		
**51**	–	CH_2_	SO_2_	>10000^d^	>80	
				[14%]	[1%]	
**52**	–	CH_2_	CHF	4160	>80	2.30
				(*5.38 ± 0.14*)	[9%]	
				[100%]		
**53**	–	CH_2_	CHOH	>10000^d^	–	
				[15%]		
**54**	–	CH_2_	C=O	4680	>80	2.58
				(*5.33 ± 0.04*)	[2%]	
				[93%]		
**55**	–	NH	CH_2_	591	>80	4.07
				(*6.23 ± 0.22*)	[18%]	
				[103%]		

a Inhibition of FSK (25 μM)-induced cAMP production in CHO-hGPR84 cells. Efficacy is expressed as a percentage of the maximum response observed, normalized to the maximum response for capric acid (100 μM). *n* = 3 if not specified. Efficacy at 30 μM is shown for compounds with potency >10 μM.

b Recruitment of β-arrestin in CHO-β-arrestin-hGPR84 cells. *n* = 1 if no detectable activation up to 80 μM. Efficacy at 80 μM was normalized to the maximum response of 6-OAU (80 μM).

c *n* = 34.

d *n* = 1.

e *n* = 3.

#### Bioisosteric Replacement of Pyridine *N*-Oxide

Before we started the SAR investigation on region A, a bioisosteric replacement strategy was first explored to potentially mitigate against pyridine *N*-oxide decomposition through aldehyde oxidase metabolism.^51^ It is well-documented that pyridone can be a bioisostere of pyridine *N*-oxide^52^ due to their structural resemblance and similar hydrogen bond accepting ability. The *O*-methyl-protected hydroxypyridines **56** and **57** showed no activity in the cAMP assay, possibly due to the methyl substituent blocking a HBA interaction (Table 4). The 6-substituted pyridone **58** showed cAMP inhibition (full agonism), β-arrestin potency, and LLE comparable to those of **11**, suggesting the pyridone moiety is engaging in similar interactions to the pyridine *N*-oxide. 4-Substituted pyridone **59** had a 17-fold decrease in potency, possibly suggesting that the regiochemistry of the hydrogen bond donor (HBD) N–H can attenuate potency. **60** was therefore prepared as a bioisosteric analogue of **19** in order to compare metabolic stability. However, the poor stability of **60** (MLM *t*_1/2_ = 11.7 min) compared to pyridine *N*-oxide **19** suggested the pyridone moiety itself was the main source of the metabolic liability. Pyridones were therefore not explored further, and modifications to the pyridine *N-*oxide group were explored as an alternative.

**Table 4:**
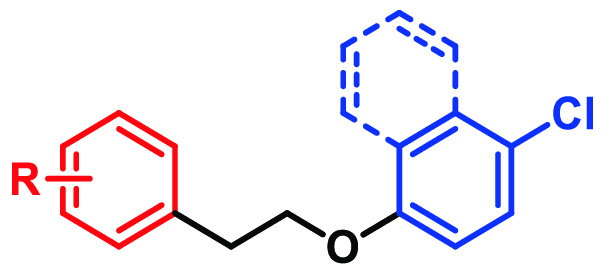

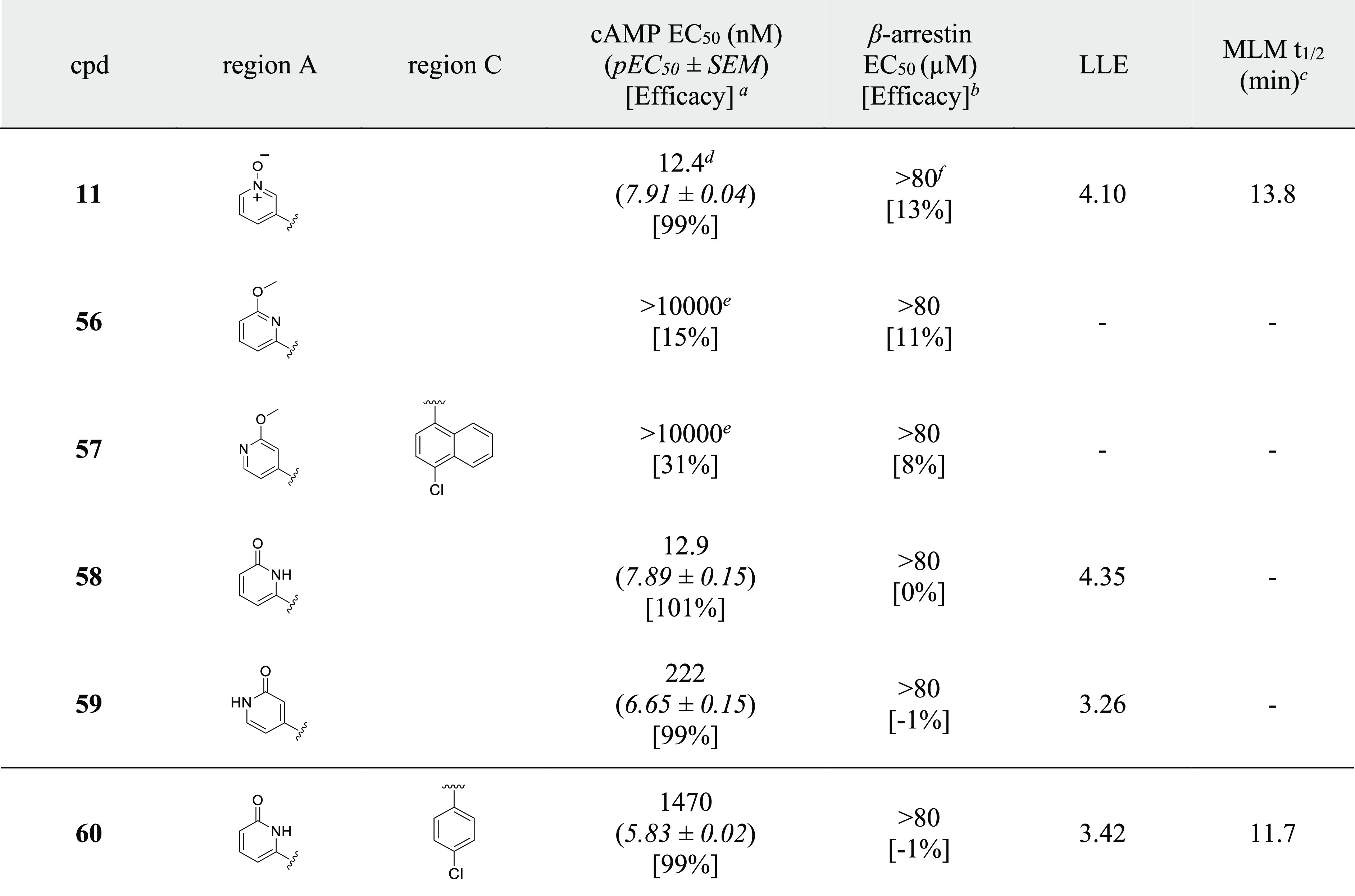
Bioisosteric Replacement of Region A

a Inhibition of FSK (25 μM)-induced cAMP production in CHO-hGPR84 cells. Efficacy is expressed as a percentage of the maximum response observed, normalized to the maximum response for capric acid (100 μM). *n* = 3 if not specified. Efficacy at 30 μM is shown for compounds with potency >10 μM.

b Recruitment of β-arrestin in CHO-β-arrestin-hGPR84 cells. *n* = 1 if no detectable activation up to 80 μM. Efficacy at 80 μM was normalized to the maximum response of 6-OAU (80 μM).

c Mouse liver microsomes.

d *n* = 34.

e *n* = 1.

f *n* = 3.

#### SAR of Region A

The investigation of region A was focused on enhancing the activity by varying substitutions at R_1_, R_2_, and R_3_ (Table 5). The substitution of R_2_ with methyl or ethyl (**61**, **62**) showed a moderate to large drop in potency, suggesting a steric limitation at this position. In contrast to **61**, the dramatically decreased activity with the additional methyl group at R_1_ (**63**) indicated that substitution at R_1_ was not tolerated. By switching the ether linker into an alkyl linker, **64** showed a comparable potency and slightly decreased LLE compared to **11**, which is consistent with the naphthalene series in Table 3. However, introducing a hydroxy substituent at R_3_ (**66**) gave a 2000-fold increase in potency, measured through inhibition of cAMP levels, functioning as a full agonist in comparison to a reference ligand (capric acid), while still showing no β-arrestin recruitment up to the highest concentration tested (80 μM). Region A of compound **66** was noted to bear some structural similarity with the pyrimidinedione functionality within 6-OAU and PSB-16434 (Figure 1), leading us to speculate whether the additional hydroxy group could function as a HBA, similar to the proposed binding mode of the pyrimidinedione tautomeric form of 6-OAU and PSB-16434 with GPR84. Interestingly, however, the introduction of a fluoro or methoxy substituent at R3 (**67** and **65**, respectively) led to a decrease in activity, suggesting that the new HBD at R3 may in fact be responsible for the enhanced potency. These experimental observations may suggest a closer similarity of **66** to the dihydroxypyrimidine and dihydroxypyridine tautomers of ZQ-16 and LY-237, respectively. This enhanced potency of **66** is presumably due to a new hydrogen bond formed with the adjacent residues, potentially with Arg172, in GPR84, as **11** initiates GPR84 activation via a mechanism that is independent of Arg172, which is essential for other lipid-like ligands.^40^ With the enhanced potency, the LLE of **66** increased to a value over 5, well within an acceptable range in drug discovery projects.^53^

**Table 5:**
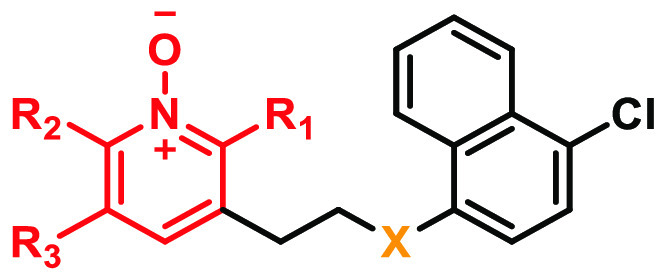
Structure–Activity Relationship of Region A

					cAMP EC_50_ (nM)	β-arrestin	
					(*pEC*_50_ *± SEM*)	EC_50_ (μM)	
cpd	R_1_	R_2_	R_3_	X	[Efficacy]^a^	[Efficacy]^b^	LLE
**11**	H	H	H	O	12.4^c^	>80^e^	4.10
					(*7.91 ± 0.04*)	[13%]	
					[99%]		
**61**	H	CH_3_	H	O	76.7	>80	2.81
					(*7.12 ± 0.12*)	[3%]	
					[95%]		
**63**	CH_3_	CH_3_	H	O	3920	>80	0.85
					(*5.41 ± 0.13*)	[2%]	
					[101%]		
**64**	H	H	H	CH_2_	8.6	>80	3.42
					(*8.07 ± 0.30*)	[7%]	
					[100%]		
**65**	H	H	OCH_3_	CH_2_	56.4	>80	2.66
					(*7.25 ± 0.12*)	[18%]	
					[99%]		
**66**	H	H	OH	CH_2_	0.00320	>80^e^	7.01
					(*11.50 ± 0.20*)	[14%]	
					[97%]		
**67**	H	H	F	O	17.9	>80	3.77
					(*7.75 ± 0.20*)	[3%]	
					[97%]		

a Inhibition of FSK (25 μM)-induced cAMP production in CHO-hGPR84 cells. Efficacy is expressed as a percentage of the maximum response observed, normalized to the maximum response for capric acid (100 μM). *n* = 3 if not specified. Efficacy at 30 μM is shown for compounds with potency >10 μM.

b Recruitment of β-arrestin in CHO-β-arrestin-hGPR84 cells. *n* = 1 if no detectable activation up to 80 μM. Efficacy at 80 μM was normalized to the maximum response of 6-OAU (80 μM).

c *n* = 34.

d *n* = 1.

e *n* = 3.

#### SAR Summary

**Figure 3:**
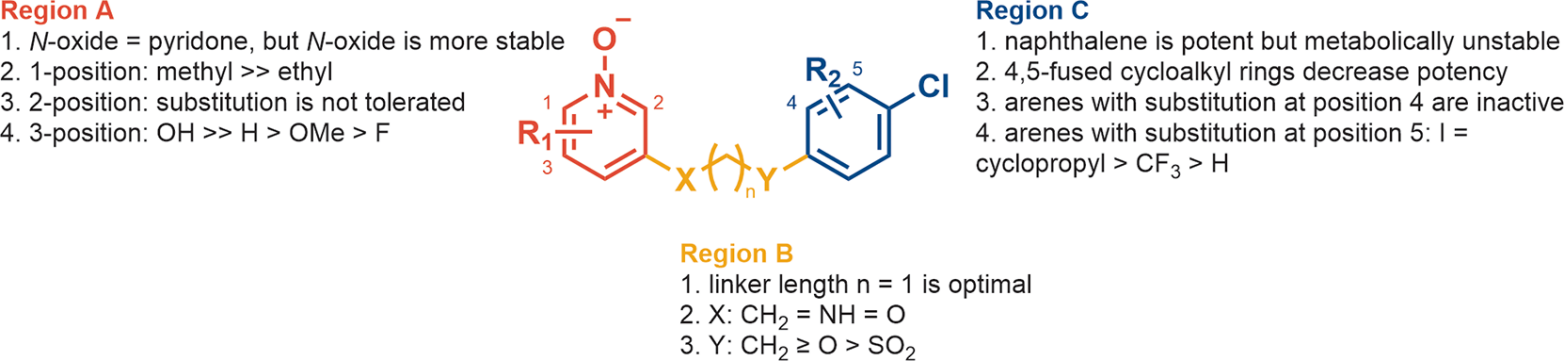
SAR summary of **11** at human GPR84.

To summarize, the SAR surrounding compound **11** was investigated by looking at three different regions (Figure 3): region A did not tolerate substitution at positions 1 and 2 (Figure 3, region A), while the addition of a HBD at position 3 boosted the potency by ≥1000-fold; 3-atom linker length was found to be optimal for region B; region C was the hotspot for metabolic liability, but this was mitigated by replacing the naphthyl substituent with an arene. This led to a loss of activity that was alleviated by adding a substituent on position 5 (Figure 3, region C) and changing the O atom at position Y to a CH_2_ (Figure 3, region B).

### Design and Synthesis of Highly Potent, Biased, and Metabolically Stable Agonists

**Figure 4:**
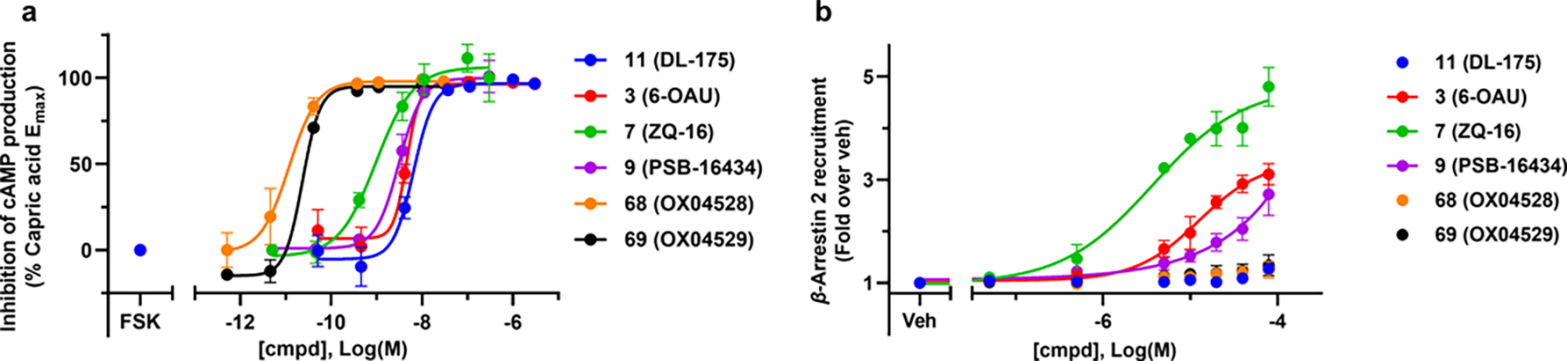
Representative cAMP inhibition and β-arrestin-2 recruitment assays illustrating **68** and **69** as highly potent G-protein pathway biased agonists in GPR84 transfected CHO cells. (a) **68** and **69** show an increased potency compared to **3**, **7**, **9**, and **11**. The assay tested inhibition of FSK (25 μM)-induced cAMP production in CHO-hGPR84 cells. (Data plotted from *n* = 2 independent experiments and shown as mean ± SD.) (b) Despite robust activation of G_i_, **68** and **69** do not show β-arrestin-2 recruitment compared to **3**, **7**, and **9**. (Data plotted from *n* = 3 independent experiments and shown as mean ± SEM.)

**68** (OX04528) and **69** (OX04529) were designed (Table 6) and synthesized by merging the key findings from the three regions together. Both **68** and **69** showed high potency and G-protein signaling bias. It is well-known that receptor expression levels can impact the measurement of apparent potency in cell-based assays, with higher GPR84 expression levels giving higher apparent potency values for the same ligand.^39^ To circumvent this potentially confounding issue, we compared **68** and **69** for their effects in cAMP and β-arrestin recruitment assays with several reference ligands with varying degrees of reported bias to compare relative potency values—these included 6-OAU (**3**), ZQ-16 (**7**), and PSB-16343 (**9**). A potency rank order was determined as **68** > **69** > **7** > **9** > **3** > **11** in cAMP assays and **7** > **3** > **9** > **11** = **68** = **69** in β-arrestin assays. Compared to previously reported ligands, **68** and **69** showed a greatly improved activity in cAMP inhibition (Figure 4a and Table s2), while both ligands showed no β-arrestin recruitment at the highest concentrations tested (Figure 4b). This may be due to a different binding mode compared to other ligands, consistent with the previously observed differences in pharmacology between 6-OAU and DL-175.^40^ These differences will need to be further investigated with **68** and **69**.

**Table 6:**
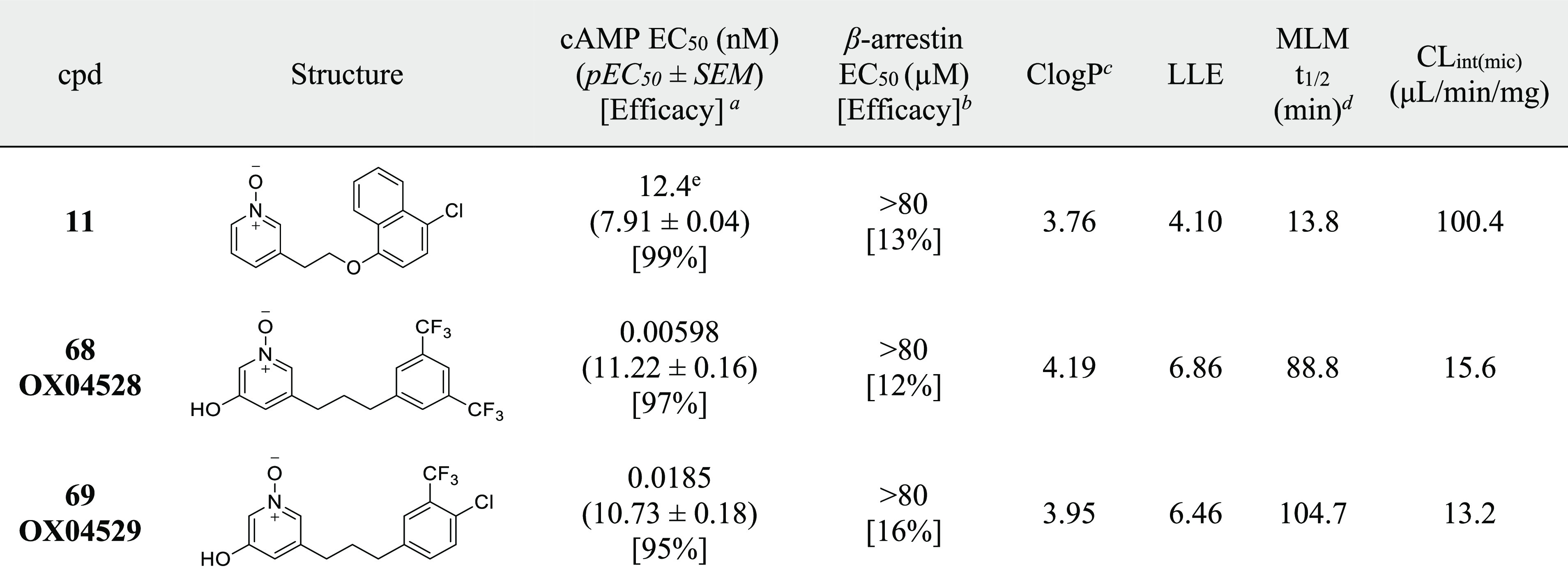
*In Vitro* ADME

a Inhibition of FSK (25 μM)-induced cAMP production in CHO-hGPR84 cells. Efficacy is expressed as a percentage of the maximum response observed, normalized to the maximum response for capric acid (100 μM). *n* = 3 if not specified. Efficacy at 30 μM is shown for compounds with potency >10 μM.

b Recruitment of β-arrestin in CHO-β-arrestin-hGPR84 cells. *n* = 3. Efficacy at 80 μM was normalized to the maximum response of 6-OAU (80 μM).

c ClogP is calculated by DataWarrior (5.5.0).

d Mouse liver microsomes.

e *n* = 34.

*In vitro* ADME profiling revealed that **68** and **69** were slowly degraded in MLM fractions (*t*_1/2_ = 89 and 105 min, respectively) and were more metabolically stable than **42** and **19**, suggesting the hydroxy group at position 3 may also be slowing down metabolism of the pyridine *N*-oxide. With a 3-log magnitude increase in potency, both compounds **68** and **69** showed an improved LLE of >5.

### Selectivity of New GPR84 Agonists

**Figure 5:**
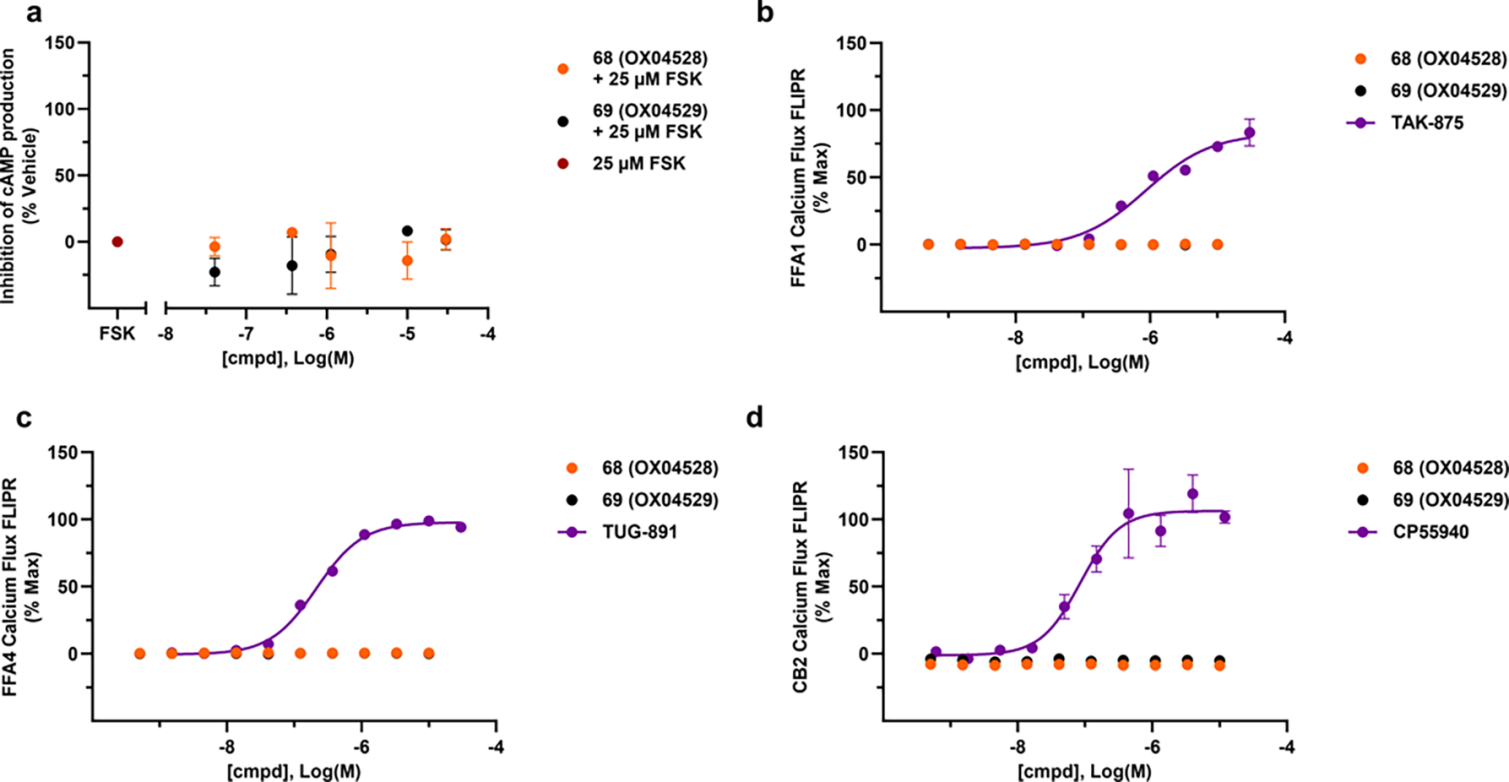
**68** and **69** inhibit cAMP production through GPR84, and both ligands show no activity at FFA1, FFA4, and CB2. (a) **68** and **69** show no inhibition of cAMP production in CHO-K1 cells. (Data plotted from *n* = 3 independent experiments and shown as mean ± SEM.) (b–d) **68** and **69** are inactive in FLIPR assays at FFA1, FFA4, and CB2. (Data plotted from *n* = 2 and shown as mean ± SD.)

To confirm that the inhibition of cAMP production induced by **68** and **69** is mediated by GPR84, cAMP assays were performed on untransfected CHO-K1 parental cells, and no inhibition of cAMP was detected (Figure 5a). Based on previously published pharmacological evidence,^40^ the binding of **11** was proposed to overlap with the binding site of **3** and MCFAs, although likely with a different binding mode, as receptor mutagenesis studies showed that GPR84 Arg172Ala mutagenesis abolished the interaction of MCFAs and **3**, but not **11**.^17,40^ We have previously reported that **11** shows high selectivity for GPR84 against 168 human GPCRs in β-arrestin recruitment agonist and antagonist screening assays.^14^ The activation of GPR84 by MCFAs indicates there might be potential off-target effects of **68** and **69** toward lipid sensors FFA1 and FFA4. Additionally, the lipid-sensing cannabinoid receptor 2 (CB2) can be activated by capric acid **1** in the micromolar range, and thus these three GPCRs were tested for selectivity counterscreening.^13^ In fluorometric imaging plate reader (FLIPR) Ca^2+^ assays (Figure 5b–d), **68** and **69** displayed no activity at FFA1, FFA4, and CB2.

### *In Vitro* Cytotoxicity

Toxicity is an essential consideration in drug development. Therefore, cytotoxicity of **68** and **69** was examined by measuring lactate dehydrogenase (LDH) release from CHO-hGPR84 cells and CHO-K1 cells (Figure S4). Neither **68** nor **69** showed evidence of cytotoxicity after a 20 h incubation at all concentration tested (up to 30 μM).

### *In Vivo* Pharmacokinetics Studies

**Figure 6:**
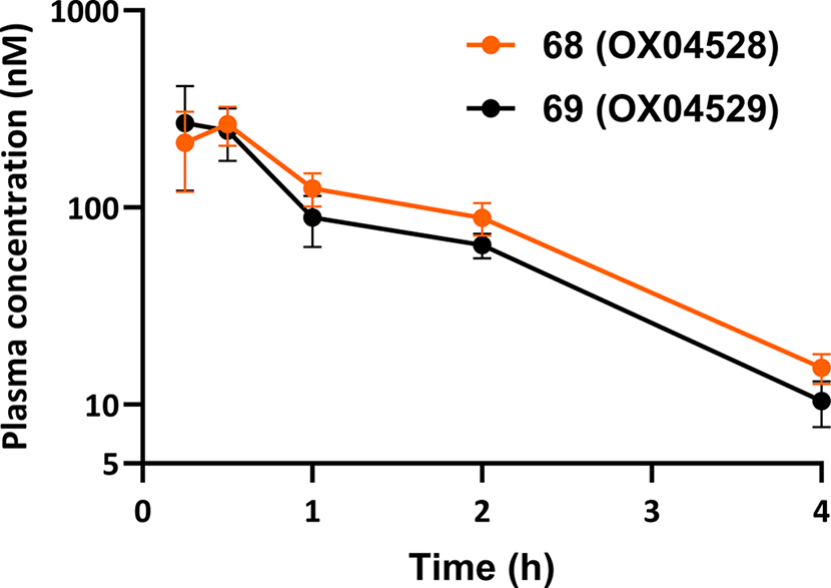
Concentrations of **68** and **69** in plasma (total) following oral dosing (10 mg/kg) of male C57BL/6J mice. Values are mean ± SD, *n* = 2.

As **68** and **69** both showed significantly enhanced potency, signaling bias, and improved metabolic stability in *in vitro* assays, both were taken forward for *in vivo* PK profiling. Both compounds were found to be orally bioavailable and to have an appropriate *in vivo* half-life of 58 and 53 min, respectively (Table 7). Following oral dosing at 10 mg/kg, both compounds showed a total concentration of around 10 nM in plasma after 4 h, well above the determined values for cellular potency, supporting their progression into *in vivo* efficacy studies (Figure 6). Taken together, these data suggest that both **68** and **69** will be useful *in vivo* probes that warrant further investigation.

**Table 7:**
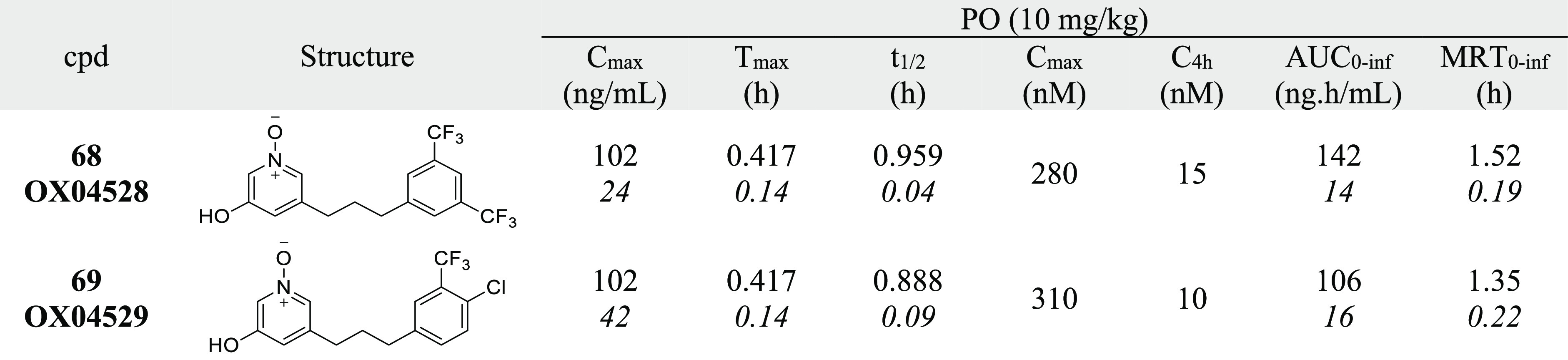
*In Vivo* Pharmacokinetics^a^

a Italics indicates standard deviation (SD). *C*_max_ is the peak drug concentration, *T*_max_ is the peak time, AUC is the area under the curve, and MRT is the mean residence time.

## Conclusion

Discovery of the potent GPR84 biased agonists **68** and **69** began with the finding that replacement of the *para*-chloronaphthalene (region C, Figure 2) in **11** with 4-chlorophenyl resulted in a 100-fold decrease in potency but enhanced metabolic stability. Further optimization of region C led to some recovery of activity and maintained MLM stability. The comparable potency of the ether and the all-carbon linker (region B, Figure 2) broadened the variety for subsequent SAR investigations. Attempted bioisosteric replacement of *N*-oxide with pyridone failed to improve either activity or metabolic stability. The SAR investigation on region A found that the addition of a hydroxy group caused a 3-log boost in potency compared to **11** while retaining high signaling bias, as evidenced by its inactivity in a β-arrestin recruitment assay. In order to develop candidates with suitable drug metabolism and pharmacokinetics profiles, **68** and **69** were designed and synthesized. Both tool compounds showed extremely high potency, with EC_50_ values of 5.98 and 18.5 pM, respectively, and no detectable activity in the β-arrestin assay (Figure 4). Moreover, these compounds were selective over FFA1, FFA4, and CB2 receptors. Exposure of cells to **68** and **69** for 20 h did not result in LDH release, indicating a maintenance of cell viability and low cytotoxicity of these compounds *in vitro*. *In vitro* metabolism studies of **68** and **69** showed an enhanced MLM stability. In mouse PK studies, oral dosing of **68** and **69** showed *in vivot*_1/2_ values of 58 and 53 min, respectively. However, given the high activity of the two compounds, the total concentration in plasma at 4 h is higher than each of their EC_50_, which provides the opportunity for further *in vivo* studies. In conclusion, **68** and **69** are highly potent, G-protein biased, and orally bioavailable agonists at GPR84 that show no detectable activity at FFA1, FFA4, and CB2 and *ergo* are suitable for *in vitro* and *in vivo* studies to help unravel the complex pharmacology and physiological and pathophysiological functions of this receptor in the future.

## Chemistry

**Scheme 1:**
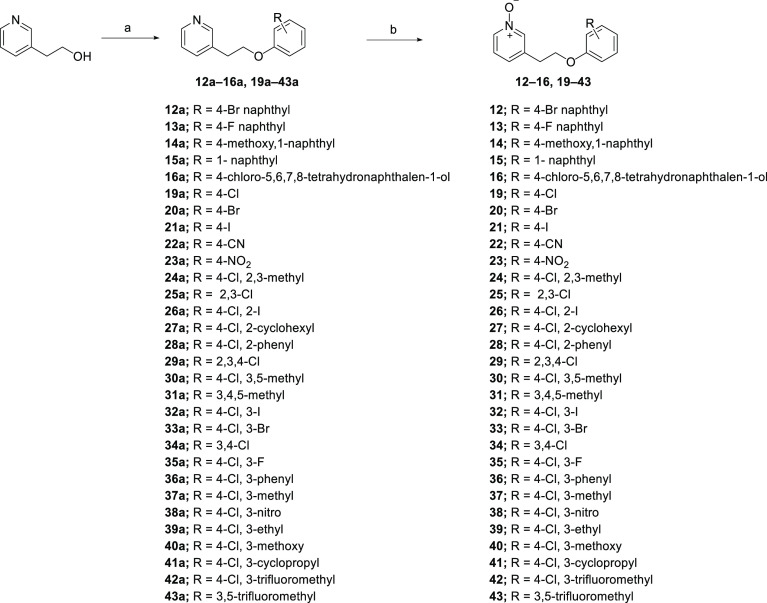
Synthesis of Systematic Region C Analogues **12**–**16**, **19**–**43** Reagents and conditions: (a) substituted phenols, DIAD, PPh_3_, THF, rt, 16 h, 26–86%; (b) *m-*CPBA, CH_2_Cl_2_, rt, 2 h, 44–96%.

The synthesis of a series of analogues bearing varied aryl groups (**12–16**, **19–43**) is depicted in Scheme 1. Mitsunobu reaction with appropriately substituted phenols using diisopropyl azodicarboxylate (DIAD) and PPh_3_ yielded the corresponding pyridines **12a–16a**, **19a–43a**. Oxidation of these intermediates in the presence of *meta*-chloroperoxybenzoic acid (*m*-CPBA) at room temperature (rt) afforded the desired pyridine *N*-oxide analogues **12–16**, **19–43**.

**Scheme 2:**
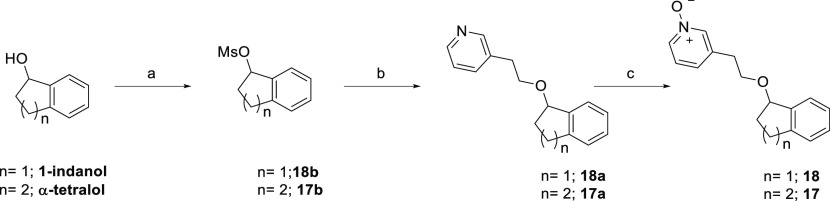
Synthesis of Analogues **17** and **18** Reagents and conditions: (a) MsCl, Et_3_N, CH_2_Cl_2_, 0 °C to rt, 16 h, 60–90%; (b) **70**, NaH, DMF, 100 °C, 16 h; (c) *m-*CPBA, CH_2_Cl_2_, rt, 2 h, 80–82%.

Mesylation of 1-indanol and tetralol followed by displacement of the corresponding mesylates **18b** and **17b** with 2-(pyridin-3-yl)ethan-1-ol led to the formation of alkoxyetherpyridine intermediates **18a** and **17a** (Scheme 2). Treatment of **18a** and **17a** with *m*-CPBA at rt afforded the corresponding pyridine *N*-oxide analogues **18** and **17**.

**Scheme 3:**
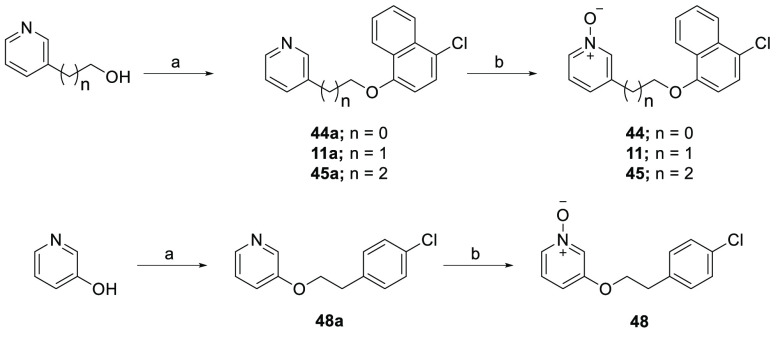
Synthesis of Systematic Region B Analogues **11**, **44**, **45**, and **48** Reagents and conditions: (a) 4-chloronaphthalen-1-ol, DIAD, PPh_3_, THF, rt, 16 h, 53–60%; (b) *m-*CPBA, CH_2_Cl_2_, rt, 2 h, 72–85%.

The preparation of analogues designed to explore the SAR for the region B analogues **44**, **11**, and **45** is shown in Scheme 3. Mitsunobu reaction of a series of different length pyridine alcohols with 4-chloronaphthalen-1-ol using DIAD and PPh_3_ afforded the corresponding phenoxyethylpyridine intermediates **44a**, **11a**, and **45a**. The resulting pyridine intermediates were converted into the corresponding pyridine *N*-oxide analogues **44**, **11**, and **45** by using *m*-CPBA. Similarly, compound **48** was synthesized via Mitsunobu reaction followed by *m*-CPBA oxidation reaction.

**Scheme 4:**
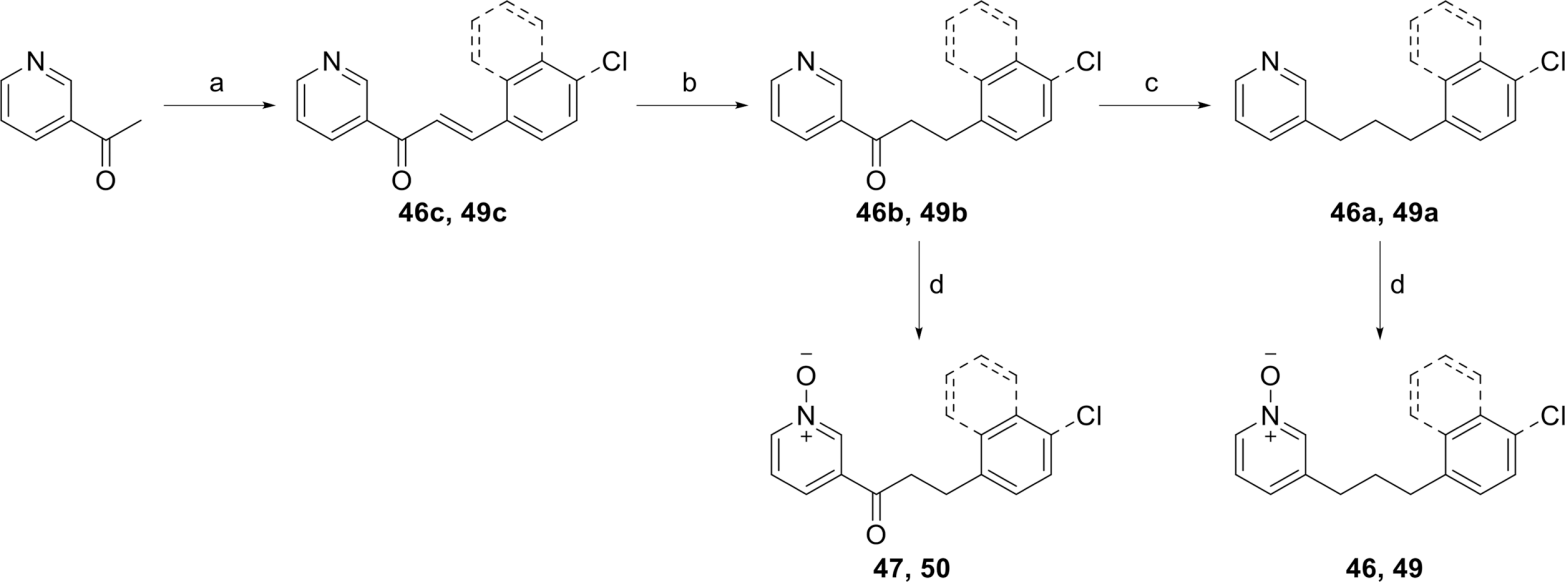
Synthesis of Naphthalene Analogues **46**, **47**, **49** and **50a** Reagents and conditions: (a) 1-naphthaldehyde or 4-chlorobenzaldehyde, NaOH, MeOH, rt, 48 h, 25–76%; (b) 10% Pd/C, H_2_, MeOH, rt, 1 h, 72–78%; (c) KOH, N_2_H_4_·H_2_O, ethylene glycol, 180 °C, 6 h, 68–98%; (d) *m*-CPBA, CH_2_Cl_2_, rt, 2 h, 78–94%.

Preparation of pyridine *N*-oxides bearing naphthalene ring on the right-hand side is shown in Scheme 4. Aldol condensation of 1-naphthaldehyde with 1-(pyridin-3-yl)ethan-1-one using NaOH at rt afforded α,β-unsaturated ketone **46c**. Pd-catalyzed hydrogenation of α,β-unsaturated ketone **46c** yielded intermediate **46b**, which was oxidized with *m*-CPBA to obtain ketone-linked pyridine *N*-oxide analogue **47**. Wolff–Kishner reduction of ketone intermediate **46b** followed by subsequent *m*-CPBA oxidation gave the desired carbon-linked pyridine *N*-oxide **46**. Compounds **49** and **50** were prepared via the same synthetic route.

**Scheme 5:**
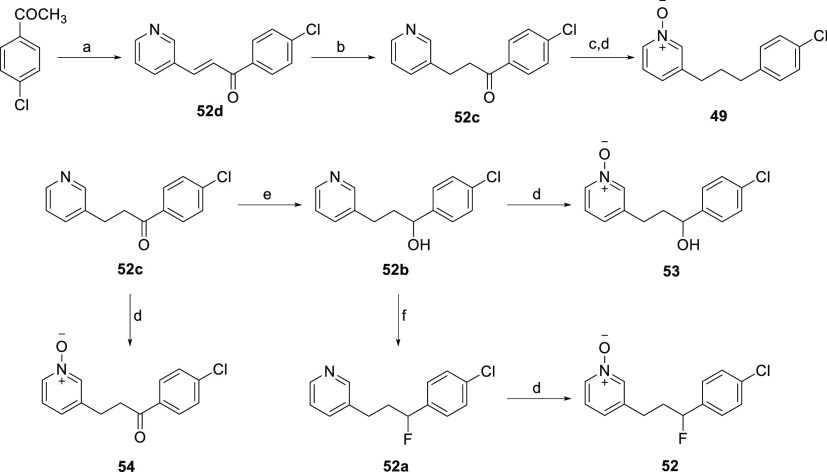
Synthesis of Systematic Linker Analogues **49**, **52**, **53**, and **54** Reagents and conditions: (a) 3-pyridinecarboxaldehyde, DBU, THF, rt, 48 h, 63%; (b) 5% PtO_2_, H_2_, MeOH, rt, 1 h, 78%; (c) KOH, N_2_H_4_·H_2_O, ethylene glycol, 180 °C, 8 h, 94%; (d) *m-*CPBA, rt, 2 h, 59–74%; (e) NaBH_4_, MeOH, 0 °C to rt, 2 h, 90% (f) DAST, CH_2_Cl_2_, 0 °C to rt 2 h, 87%.

Scheme 5 illustrates the synthetic route for the preparation of linker analogues **49**, **52**, **53**, and **54**. The synthesis of common ketone intermediate **52c** started from the base-catalyzed aldol condensation reaction of 4-chloroacetophenone with 3-pyridine carboxaldehyde in the presence of 1,8-diazabicyclo[5.4.0]undec-7-ene (DBU) to afford α,β-unsaturated ketone **52d**. Hydrogenation of **52d** using 5% PtO_2_ yielded ketone intermediate **52c**. Wolff–Kishner reduction of ketone **52c** with hydrazine hydrate and KOH at 180 °C followed by *m*-CPBA oxidation gave the desired carbon-linked pyridine *N*-oxide **49**. The ketone-linked pyridine *N*-oxide derivative **54** was obtained by *m*-CPBA oxidation of ketone intermediate **52c**. NaBH_4_ reduction of ketone **52c** afforded the corresponding hydroxy intermediate **52b**, which on further treatment with *m*-CPBA gave the desired pyridine *N*-oxide **53**. Conversion of the resulting hydroxy compound **52b** to fluorine derivative **52a** was achieved by treatment with diethylaminosulfur trifluoride (DAST) at 0 °C. Fluoropyridine intermediate **52a** was subjected to *m*-CPBA oxidation to yield the corresponding pyridine *N*-oxide **52**.

**Scheme 6:**
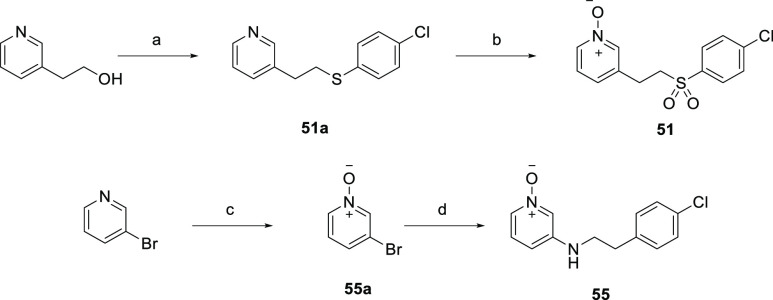
Synthesis of Sulfone and Amine Linker Analogues **51** and **55** Reagents and conditions: (a) (i) MsCl, Et_3_N, CH_2_Cl_2_, 0 °C to rt, 16 h, 93%; (ii) 4-chlorobenzenethiol, K_2_CO_3_, THF, 50 °C, 16 h, 34%; (b) *m-*CPBA, rt, 2 h, 80%; (c) *m-*CPBA, rt, 16 h, 37%; (d) 2-(4-chlorophenyl)ethan-1-amine, *rac*-BINAP, Pd_2_(dba)_3_, NaOtBu, toluene, 80 °C, 16 h, 15%.

Scheme 6 depicts the synthesis of *N*-oxide derivatives that contain sulfone (**51**) or a secondary amino group (**55**) in the linker. Mesylation of the primary alcohol **70** followed by displacement of the mesylate with 4-chlorobenzenethiol gave the corresponding thioether pyridine intermediate **51a**. The formation of pyridine *N*-oxide sulfone **51** from **51a** was achieved via *m*-CPBA oxidation. 3-((4-Chlorophenethyl)amino)pyridine 1-oxide **55** was prepared from *m*-CPBA oxidation of 3-bromopyridine followed by the Pd-catalyzed amination reaction between 2-(4-chlorophenyl)ethan-1-amine and 3-bromopyridine *N*-oxide **55a** using *rac*-BINAP and Pd_2_(dba)_3_ in the presence of NaOtBu (Scheme 6).

**Scheme 7:**
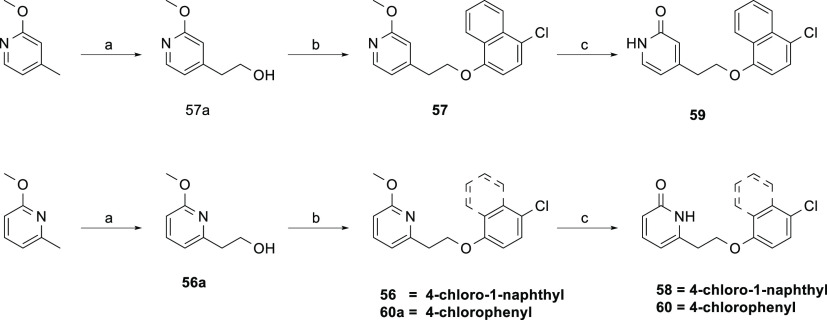
Synthesis of Pyridone Derivatives **58**–**60** Reagents and conditions. (a) paraformaldehyde, *n*-BuLi, THF, −78 °C to rt, 4 h, 18–28%; (b) 4-chloronaphthalen-1-ol, DIAD, PPh_3_, THF, rt, 16 h, 52–72%; (c) TMSCl, NaI, MeCN, 80 °C, 4 h, 66–99%.

The synthesis of pyridone derivatives **58**–**60** (Scheme 7) started with the homologation of the corresponding pyridine to afford **56a** and **57a**, followed by Mitsunobu reaction to give methoxypyridine intermediates **56**, **57**, and **60a**. Demethylation of the intermediates using TMSCl and NaI at 80 °C yielded the pyridine analogues **58**, **59**, and **60**.

**Scheme 8:**
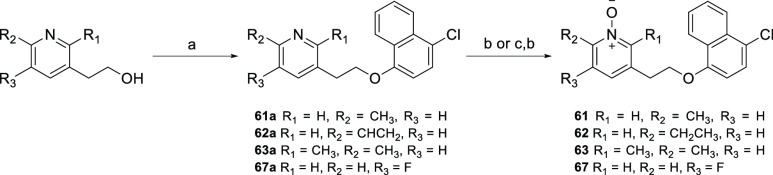
Synthesis of Polar Head Group Analogues **61**–**63** and **67** Reagents and conditions: (a) 4-chloronaphthalen-1-ol, DIAD, PPh_3_, THF, rt, 16 h, 58–89%; (b) *m-*CPBA, CH_2_Cl_2_, rt, 2 h, 89–95%; (c) 10% Pd/C, H_2_, MeOH, rt, 1 h, 79%.

Mitsunobu reaction of 4-chloronaphthalen-1-ol with substituted pyridine alcohols using PPh_3_ and DIAD afforded intermediates **61a–63a** and **67a**, which were oxidized with *m*-CPBA to yield the corresponding *N*-oxides **61–63** and **67** (Scheme 8). The olefin intermediate **62a** was subjected to Pd-catalyzed hydrogenation before being treated with *m*-CPBA to afford the desired compound **62**.

**Scheme 9:**
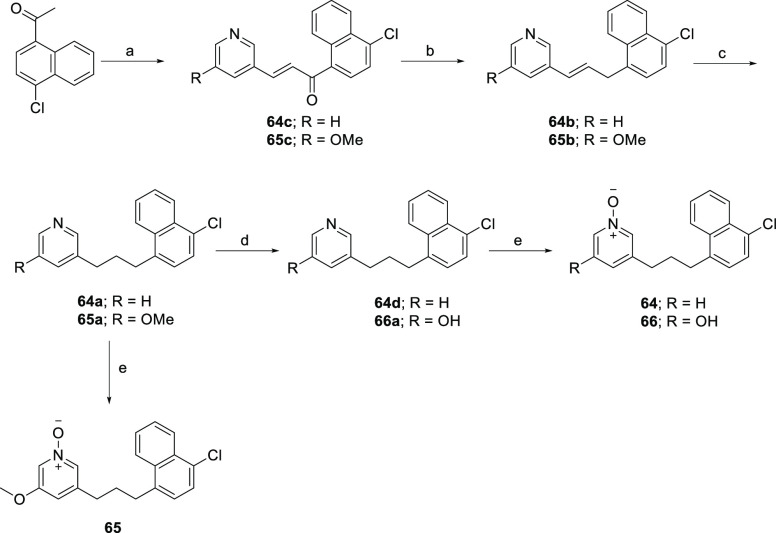
Synthesis of Polar Head Group Analogues **64**–**66** Reagents and conditions: (a) 3-pyridinecarboxaldehyde or 5-methoxynicotinaldehyde, BF_3_·Et_2_O, 1,4-dioxane, 105 °C, 16 h, 53–94%; (b) Et_3_SiH, TFA, rt, 48 h, 50–75%; (c) 5% PtO_2_ or 10% Pd/C, H_2_, MeOH, rt, 1 h, 39–62%; (d) BBr_3_, −78 °C to rt, 16 h, 30%; (e) *m-*CPBA, CH_2_Cl_2_, rt, 2 h, 43–86%;

The synthetic route for the naphthalene pyridine *N*-oxide analogues **64**–**66** started with the BF_3_·Et_2_O-induced aldol condensation reaction between pyridine aldehydes and 1-(4-chloronaphthalen-1-yl)ethan-1-one in 1,4-dioxane at 105 °C, providing intermediates **64c** and **65c** (Scheme 9). Selective reduction of the ketone was achieved by treatment of α,β-unsaturated ketones **64c** and **65c** with Et_3_SiH in TFA to give the desired olefins **64b** and **65b**, which were treated with PtO_2_ or subjected to Pd-catalyzed hydrogenation to produce intermediates **64a** and **65a**. Preparation of the hydroxy intermediate **66a** was achieved by treatment of compound **65a** with BBr_3_. Finally, the desired naphthalene pyridine *N*-oxide analogues **64**–**66** were prepared via *m*-CPBA oxidation of the pyridine intermediates.

**Scheme 10:**
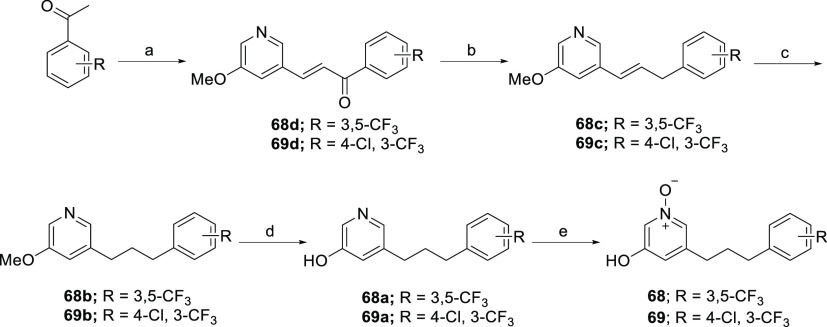
Synthesis of 5-Hydroxypyridine *N*-Oxide Analogues **68** and **69** Reagents and conditions: (a) 5-methoxynicotinaldehyde, BF_3_·Et_2_O, 1,4-dioxane, 105 °C, 16 h, 78–80%; (b) Et_3_SiH, TFA, rt, 48 h, 42–70%; (c) 5% PtO_2_ or 10% Pd/C, H_2_, MeOH, rt, 1 h, 78–85%; (d) 48% HBr, reflux, 48 h, 77–82%; (e) *m-*CPBA, CH_2_Cl_2_, rt, 2 h, 70–73%.

Following the same synthetic route outlined in Scheme 9, 5-methoxypyridine intermediates **68b** and **69b** were synthesized from 5-methoxynicotinaldehyde and the requisite substituted acetophenones. The demethylation of **68b** and **69b** was achieved by using 48% HBr in water at 120 °C; this was followed by *m*-CPBA oxidation of hydroxypyridine **68a** and **69a**, providing the desired 5-hydroxypyridine *N*-oxide analogues **68** and **69** (Scheme 10).

## Experimental Section

### General Information

All reactions involving moisture-sensitive reagents were carried out under a nitrogen or an argon atmosphere. Anhydrous solvents were dried by passing over an activated alumina column, under an inert atmosphere, using a solvent purification system. All other solvents and reagents were used as supplied (analytical or HPLC grade) without prior purification. Flash column chromatography was performed on Kieselgel 60 silica gel (230–400 mesh particle size) on a glass column or on a Biotage SP4 automated flash column chromatography platform. NMR spectra were recorded on Bruker Advance spectrometers at 400, 500, or 600 MHz in the deuterated solvent stated at room temperature. The field was locked by external referencing to the relevant deuteron resonance. Chemical shifts (δ) are reported in parts per million (ppm), and coupling constants (*J*) are quoted in hertz (Hz). Data are reported as follows: chemical shift, multiplicity (s = singlet, d = doublet, t = triplet, q = quartet, sext = sextuplet, hept = heptet, and m = multiplet), coupling constant, and integration. Low-resolution mass spectra (*m*/*z*) were recorded on an Agilent 1260 Infinity II with diode array and single-quadrupole detectors in MeOH. A selected peak is reported in daltons (Da), and its intensity is given as percentage of the base peak. High-resolution mass spectra (HRMS) were run on a Bruker microTOF (ESI and APCI) or on a Waters GCT (EI) instrument. Experiments conducted in contract research organizations (CROs) used their standard equipment. All compounds subjected to biological testing were of >95% purity as measured by HPLC on a Shimadzu SIL-20AC HT instrument. HPLC conditions were as follows: Atlantis dC18, 100 Å, 5 μm, column 4.6 × 150 mm, 35–100% acetonitrile in water, 15 min run, flow rate 1.5 mL/min, UV detection (λ = 220, 254, 280 nm).

### General Procedure A

To a solution of the requisite alcohol (1.0 equiv) in tetrahydrofuran (0.1 M) at rt were added sequentially the corresponding phenol (1.5 equiv), triphenylphosphine (1.5 equiv), and diisopropyl azodicarboxylate (1.5 equiv), and the resulting mixture was stirred for 16 h. The reaction was concentrated *in vacuo*, and the residue was taken up in HCl (1 M, aq.) and extracted with Et_2_O (×3). The aqueous phase was neutralized with NaOH (2 M, aq.) and extracted with EtOAc (×2). The EtOAc layer was washed with brine, dried (Na_2_SO_4_), filtered, and concentrated *in vacuo*. The compound was then purified by flash column chromatography (silica gel).

### General Procedure B

To a solution of the requisite pyridine (1.0 equiv) in CH_2_Cl_2_ (0.2 M) at rt was added *m*-CPBA (1.5 equiv), and the resulting mixture was stirred for 2 h at rt. After completion, the reaction was quenched with NaOH (2 M, aq.). The organic phase was washed with brine and H_2_O, dried (Na_2_SO_4_), filtered, and concentrated *in vacuo*. The compound was then purified by flash column chromatography (silica gel).

### General Procedure B′

To a solution of the requisite pyridine (1.0 equiv) in CH_2_Cl_2_ (0.2 M) at rt was added *m*-CPBA (1.5 equiv), and the resulting mixture was stirred for 2 h at rt. After completion of the reaction, the compound was directly purified by flash column chromatography (silica gel).

### General Procedure C

To a solution of the requisite ketone (1.0 equiv) in MeOH (0.2 M) were added NaOH (1 M, aq., 1.0 equiv) and the aldehyde (1.0 equiv) at 0 °C, and the resulting mixture was stirred for 16 h. After completion, the reaction was filtered, the solid was taken up with EtOAc, and the organic phase was washed with brine and water, dried (Na_2_SO_4_), and concentrated *in vacuo* to afford the desired product.

### General Procedure D

Pd/C (10% M/w) was added in one portion to a degassed solution of the requisite alkene (1.0 equiv) in MeOH (0.2 M) at rt, the atmosphere was replaced with H_2_, and the reaction was stirred for 2 h. After completion, the reaction was filtered through Celite using MeOH as an eluent, concentrated *in vacuo*, and purified by flash column chromatography (silica gel).

### General Procedure D′

PtO_2_ (5% M/w) was added in one portion to a degassed solution of the requisite alkene (1.0 equiv) in MeOH (0.2 M) at rt, the atmosphere was replaced with H_2_, and the reaction was stirred for 1 h. After completion, the reaction was filtered through Celite using MeOH as an eluent, concentrated *in vacuo*, and purified by flash column chromatography (silica gel).

### General Procedure E

To a solution of the requisite ketone (1.0 equiv) in ethylene glycol (0.5 M) were added KOH (1.5 equiv) and N_2_H_4_·H_2_O (10 equiv). The resulting mixture was heated at 180 °C for 6 h. After completion, the reaction was quenched with acetone and concentrated *in vacuo*, and the resulting residue was purified by flash column chromatography (silica gel).

### General Procedure F

*n*-BuLi (1.1 equiv) was added dropwise to a solution of the requisite methoxy pyridine (1.0 equiv) in THF (0.5 M) at −78 °C. The reaction was stirred for 30 min before addition of paraformaldehyde (4.0 equiv) in one portion, warmed to rt, and stirred for 4 h. After completion, the mixture was poured into water and extracted with EtOAc, and the organic phase was washed with brine and H_2_O, dried (Na_2_SO_4_), and concentrated *in vacuo*. The crude product was purified by flash column chromatography (silica gel).

### General Procedure G

TMSCl (3.5 equiv) and NaI (3.5 equiv) were added sequentially to a solution of the requisite methoxypyridine (1.0 equiv) in MeCN (0.1 M), and the resulting mixture was stirred at 90 °C for 5 h. After completion, the reaction mixture was diluted with EtOAc, washed with brine and H_2_O, dried (Na_2_SO_4_), and concentrated *in vacuo*. The compound was then purified by flash column chromatography (silica gel).

#### 3-(2-((4-Bromonaphthalen-1-yl)oxy)ethyl)pyridine (**12a**)

Following general procedure A, **12a** was obtained from 4-bromo-1-naphthol (68 mg, 0.304 mmol) and 3-(2-hydroxyethyl)pyridine (25 mg, 0.204 mmol). Purification by flash column chromatography (3% MeOH in CH_2_Cl_2_) afforded the title compound as a white solid (63 mg, 95%): ^1^H NMR (400 MHz, CDCl_3_) δ 8.65 (s, 1H), 8.51 (d, *J* = 5.2 Hz, 1H), 8.19 (ddd, *J* = 8.3, 1.4, 0.7 Hz, 1H), 8.14 (dt, *J* = 8.4, 1.0 Hz, 1H), 7.69 (dt, *J* = 7.9, 1.9 Hz, 1H), 7.63–7.56 (m, 2H), 7.51 (ddd, *J* = 8.2, 6.8, 1.3 Hz, 1H), 7.30–7.23 (m, 1H), 6.63 (d, *J* = 8.2 Hz, 1H), 4.31 (t, *J* = 6.4 Hz, 2H), 3.22 (t, *J* = 6.4 Hz, 2H); *m*/*z* LRMS (ESI^+^) 328 [M+H]^+^.

#### 3-(2-((4-Bromonaphthalen-1-yl)oxy)ethyl)pyridine 1-oxide (**12**)

Following general procedure B′, **12** was obtained from **12a** (13 mg, 0.048 mmol). Purification by flash column chromatography (5% MeOH in CH_2_Cl_2_) afforded the title compound as a beige solid (12 mg, 91%): ^1^H NMR (600 MHz, CDCl_3_) δ 8.29 (s, 1H), 8.16 (d, *J* = 8.5 Hz, 2H), 8.13 (dd, *J* = 6.4, 1.6 Hz, 1H), 7.64 (dd, *J* = 8.1, 0.9 Hz, 1H), 7.61 (ddt, *J* = 8.2, 6.9, 1.2 Hz, 1H), 7.56–7.50 (m, 1H), 7.32–7.27 (m, 1H), 7.26–7.22 (m, 1H), 6.66 (d, *J* = 8.2 Hz, 1H), 4.37 (t, *J* = 6.1 Hz, 2H), 3.21 (t, *J* = 6.1 Hz, 2H); ^13^C NMR (151 MHz, CDCl_3_) δ 153.9, 139.7, 138.0, 137.8, 132.7, 129.4, 128.1, 127.2, 126.9, 126.8, 126.5, 125.9, 122.2, 114.2, 105.5, 67.5, 32.9; *m*/*z* LRMS (ESI^+^) 344 [M+H]^+^; HRMS (ESI^+^) C_17_H_15_NO_2_Br [M+H]^+^ calcd 344.0281, found 344.0271; HPLC 95% (AUC), *t*_R_ = 7.0 min.

#### 3-(2-((4-Fluoronaphthalen-1-yl)oxy)ethyl)pyridine (**13a**)

Following general procedure A, **13a** was obtained from 4-fluoro-1-naphthol (125 mg, 0.771 mmol) and 3-(2-hydroxyethyl)pyridine (64 mg, 0.514 mmol). Purification by flash column chromatography (3% MeOH in CH_2_Cl_2_) afforded the title compound as a white solid (118 mg, 86%): ^1^H NMR (400 MHz, CDCl_3_) δ 8.66 (s, 1H), 8.51 (d, *J* = 4.7 Hz, 1H), 8.20–8.13 (m, 1H), 8.03 (dd, *J* = 7.2, 1.7 Hz, 1H), 7.71 (dt, *J* = 7.8, 1.9 Hz, 1H), 7.63–7.42 (m, 2H), 7.29 (dd, *J* = 13.2, 4.6 Hz, 1H), 7.00 (dd, *J* = 10.3, 8.4 Hz, 1H), 6.66 (dd, *J* = 8.4, 3.9 Hz, 1H), 4.34 (t, *J* = 6.4 Hz, 2H), 3.24 (t, *J* = 6.4 Hz, 2H); *m*/*z* LRMS (ESI^+^) 268 [M+H]^+^.

#### 3-(2-((4-Fluoronaphthalen-1-yl)oxy)ethyl)pyridine 1-oxide (**13**)

Following general procedure B′, **13** was obtained from **13a** (13 mg, 0.048 mmol). Purification by flash column chromatography (5% MeOH in CH_2_Cl_2_) afforded the title compound as a beige solid (13 mg, 93%): ^1^H NMR (400 MHz, CDCl_3_) δ 8.30 (s, 1H), 8.13 (dd, *J* = 8.1, 6.6 Hz, 2H), 8.03 (dd, *J* = 7.5, 2.1 Hz, 1H), 7.54 (pd, *J* = 6.9, 1.6 Hz, 2H), 7.31 (d, *J* = 7.8 Hz, 1H), 7.24 (d, *J* = 7.9 Hz, 1H), 7.01 (dd, *J* = 10.2, 8.4 Hz, 1H), 6.65 (dd, *J* = 8.4, 3.8 Hz, 1H), 4.34 (t, *J* = 6.0 Hz, 2H), 3.20 (t, *J* = 6.1 Hz, 2H); ^19^F NMR (376 MHz, CDCl_3_) δ −106.39; ^13^C NMR (101 MHz, CDCl_3_) δ 154.7, 150.3, 139.7, 138.2, 137.7, 127.1, 127.0, 126.6, 125.8, 121.9, 120.6, 108.6, 108.4, 104.0, 103.9, 67.6, 32.9; *m*/*z* LRMS (ESI^+^) 284 [M+H]^+^; HRMS (ESI^+^) C_17_H_15_NO_2_F [M+H]^+^ calcd 284.1081, found 284.1074. HPLC 96% (AUC), *t*_R_ = 5.9 min.

#### 3-(2-((4-Methoxynaphthalen-1-yl)oxy)ethyl)pyridine (**14a**)

Following general procedure A, **14a** was obtained from 4-methoxynaphthalen-1-ol (212 mg, 1.22 mmol) and 2-(pyridin-3-yl)ethan-1-ol (100 mg, 0.813 mmol). Purification by flash column chromatography (20% EtOAc in CH_2_Cl_2_) afforded the title compound as a brown oil (90 mg, 40%): ^1^H NMR (600 MHz, CDCl_3_) δ 8.66 (s, 1H), 8.51 (s, 1H), 8.22–8.17 (m, 1H), 8.15–8.10 (m, 1H), 7.73 (dt, *J* = 7.9, 1.9 Hz, 1H), 7.52–7.46 (m, 2H), 7.28 (dd, *J* = 7.8, 4.8 Hz, 1H), 6.68 (q, *J* = 8.3 Hz, 2H), 4.32 (t, *J* = 6.4 Hz, 2H), 3.95 (s, 3H), 3.23 (t, *J* = 6.4 Hz, 2H); ^13^C NMR (151 MHz, CDCl_3_) δ 149.9, 149.8, 148.3, 147.5, 137.1, 134.6, 126.4, 126.4, 126.0, 125.9, 123.6, 121.8, 121.6, 104.4, 103.1, 68.4, 55.7, 33.2; *m*/*z* LRMS (ESI^+^) 280 [M+H]^+^; HRMS (ESI^+^) C_18_H_17_NO_2_ [M+H]^+^ calcd 280.1332, found 280.1332.

#### 3-(2-((4-Methoxynaphthalen-1-yl)oxy)ethyl)pyridine 1-oxide (**14**)

Following general procedure A, **14** was obtained from **14a** (30 mg, 0.108 mmol). Purification by flash column chromatography (5% MeOH in CH_2_Cl_2_) afforded the title compound as a brown oil (17 mg, 53%): ^1^H NMR (400 MHz, CDCl_3_) δ 8.28 (td, *J* = 1.6, 0.7 Hz, 1H), 8.23–8.17 (m, 1H), 8.14–8.11 (m, 1H), 8.09 (dtd, *J* = 6.8, 3.4, 0.7 Hz, 1H), 7.50 (dt, *J* = 6.4, 3.4 Hz, 2H), 7.30 (dt, *J* = 7.9, 1.3 Hz, 1H), 7.23 (dd, *J* = 7.9, 6.3 Hz, 1H), 6.68 (d, *J* = 1.9 Hz, 2H), 4.32 (t, *J* = 6.1 Hz, 2H), 3.96 (s, 3H), 3.18 (t, *J* = 6.1 Hz, 2H); ^13^C NMR (101 MHz, CDCl_3_) δ 150.1, 148.1, 139.7, 138.4, 137.6, 127.0, 126.5, 126.4, 126.3, 126.1, 125.8, 122.0, 121.6, 104.7, 103.1, 67.7, 55.9, 33.1; *m*/*z* LRMS (ESI^+^) 296 [M+H]^+^; HRMS (ESI^+^) C_18_H_17_NO_3_ [M+H]^+^ calcd 296.1281, found 296.1281; HPLC 98% (AUC), *t*_R_ = 5.0 min.

#### 3-(2-(Naphthalen-1-yloxy)ethyl)pyridine (**15a**)

Following general procedure A, **15a** was obtained from naphthalen-1-ol (121 mg, 0.840 mmol) and 2-(pyridin-3-yl)ethan-1-ol (69 mg, 0.561 mmol). Purification by flash column chromatography (2% MeOH in CH_2_Cl_2_) afforded the title compound as a yellow oil (42 mg, 30%): ^1^H NMR (400 MHz, CDCl_3_) δ 8.67 (d, *J* = 2.2 Hz, 1H), 8.51 (dd, *J* = 4.9, 1.7 Hz, 1H), 8.23–8.16 (m, 1H), 7.82–7.72 (m, 2H), 7.52–7.41 (m, 3H), 7.35 (t, 1H), 7.32–7.27 (m, 1H), 6.79 (dd, *J* = 7.6, 1.0 Hz, 1H), 4.37 (t, *J* = 6.3 Hz, 2H), 3.26 (t, *J* = 6.3 Hz, 2H); ^13^C NMR (101 MHz, CDCl_3_) δ 154.4, 149.8, 147.4, 137.4, 134.7, 134.6, 127.6, 126.6, 125.9, 125.7, 125.5, 123.8, 122.0, 120.7, 104.8, 68.1, 33.2; *m*/*z* LRMS (ESI^+^) 250 [M+H]^+^; HRMS (ESI^+^) C_17_H_15_NO [M+H]^+^ calcd 250.1226, found 250.1221.

#### 3-(2-(Naphthalen-1-yloxy)ethyl)pyridine 1-oxide (**15**)

Following general procedure B, **15** was obtained from **15a** (30 mg, 0.169 mmol). Purification by flash column chromatography (6% MeOH in CH_2_Cl_2_) afforded the title compound as a white solid (17 mg, 53%): ^1^H NMR (400 MHz, CDCl_3_) δ 8.32 (s, 1H), 8.14 (dt, *J* = 5.5, 2.7 Hz, 2H), 7.81–7.75 (m, 1H), 7.51–7.41 (m, 3H), 7.38–7.31 (m, 2H), 7.24 (d, *J* = 7.9 Hz, 1H), 6.78 (d, *J* = 7.5 Hz, 1H), 4.36 (t, *J* = 6.1 Hz, 2H), 3.20 (t, *J* = 6.0 Hz, 2H); ^13^C NMR (101 MHz, CDCl_3_) δ 154.0, 139.6, 138.4, 137.6, 134.6, 127.8, 127.7, 126.7, 125.9, 125.8, 125.6, 125.5, 121.8, 121.0, 104.8, 67.1, 32.9; *m*/*z* LRMS (ESI^+^) 266 [M+H]^+^; HRMS (ESI^+^) C_17_H_15_NO_2_ [M+H]^+^ calcd 266.1176, found 266.1175; HPLC 95% (AUC), *t*_R_ = 5.1 min.

#### 3-(2-((4-Chloro-5,6,7,8-tetrahydronaphthalen-1-yl)oxy)ethyl)pyridine (**16a**)

Following general procedure A, **16a** was obtained from 4-chloro-5,6,7,8-tetrahydronaphthalen-1-ol (150 mg, 0.824 mmol) and 2-(pyridin-3-yl)ethan-1-ol (69 mg, 0.561 mmol). Purification by flash column chromatography (20% EtOAc in CH_2_Cl_2_) afforded the title compound as a yellow solid (60 mg, 37%): ^1^H NMR (400 MHz, CDCl_3_) δ 8.57 (d, *J* = 2.3 Hz, 1H), 8.49 (dd, *J* = 4.8, 1.7 Hz, 1H), 7.61 (dt, *J* = 7.8, 2.0 Hz, 1H), 7.24 (ddd, *J* = 7.8, 4.9, 0.9 Hz, 1H), 7.10 (dt, *J* = 8.6, 0.9 Hz, 1H), 6.56 (d, *J* = 8.7 Hz, 1H), 4.13 (t, *J* = 6.3 Hz, 2H), 3.09 (t, *J* = 6.3 Hz, 2H), 2.71 (t, *J* = 6.0 Hz, 2H), 2.58 (t, *J* = 6.1 Hz, 2H), 1.81–1.63 (m, 4H); ^13^C NMR (101 MHz, CDCl_3_) δ 154.9, 150.6, 148.1, 136.6, 136.2, 134.2, 128.5, 126.4, 126.1, 123.4, 108.7, 68.1, 33.2, 27.7, 23.8, 22.5, 22.2; *m*/*z* LRMS (ESI^+^) 290 [M(^37^Cl)+H]^+^ 288 [M(^35^Cl)+H]^+^; HRMS (ESI^+^) C_17_H_18_ClNO [M+H]^+^ calcd 288.1150, found 288.1146.

#### 3-(2-((4-Chloro-5,6,7,8-tetrahydronaphthalen-1-yl)oxy)ethyl)pyridine 1-oxide (**16**)

Following general procedure B, **16** was obtained from **16a** (30 mg, 0.104 mmol). Purification by flash column chromatography (6% MeOH in CH_2_Cl_2_) afforded the title compound as a white solid (18 mg, 57%): ^1^H NMR (400 MHz, CDCl_3_) δ 8.21 (s, 1H), 8.11 (td, *J* = 3.8, 1.8 Hz, 1H), 7.24–7.19 (m, 2H), 7.11 (d, *J* = 8.6 Hz, 1H), 6.55 (d, *J* = 8.7 Hz, 1H), 4.14 (t, *J* = 6.0 Hz, 2H), 3.05 (t, *J* = 6.0 Hz, 2H), 2.71 (t, *J* = 5.9 Hz, 2H), 2.57 (t, *J* = 6.0 Hz, 2H), 1.81–1.67 (m, 4H); ^13^C NMR (101 MHz, CDCl_3_) δ 154.6, 139.8, 138.2, 137.6, 136.4, 128.5, 126.9, 126.8, 126.2, 125.7, 108.6, 67.1, 33.0, 27.7, 23.8, 22.5, 22.1; *m*/*z* LRMS (ESI^+^) 306 [M(^37^Cl)+H]^+^ 304 [M(^35^Cl)+H]^+^; HRMS (ESI^+^) C_17_H_18_^35^ClNO_2_ [M+H]^+^ calcd 304.1099, found 304.1097; HPLC 99% (AUC), *t*_R_ = 7.0 min.

#### (±)-1,2,3,4-Tetrahydronaphthalen-1-yl methanesulfonate (**17b**)

To an ice-cold solution of 1,2,3,4-tetrahydronaphthalen-1-ol (800 mg, 5.40 mmol) in CH_2_Cl_2_ (15 mL) were added sequentially Et_3_N (3.8 mL, 27.0 mmol) and MsCl (0.80 mL, 10.8 mmol). The resulting solution was stirred for 16 h at rt before addition of NaHCO_3_ (aq. sat. sol., 20 mL) and EtOAc (25 mL). The aqueous phase was extracted with EtOAc (2 × 25 mL), and then the combined organic phase was washed with H_2_O (20 mL) and brine (20 mL), dried (Na_2_SO_4_), filtered, and concentrated *in vacuo* to afford title compound **17b** as a yellow oil (1.10 g, 90%), which was taken into next step without purification.

#### (±)-1,2,3,4-Tetrahydronaphthalen-1-yl methanesulfonate (**17a**)

NaH (97 mg, 2.43 mmol) was added to an ice-cold solution of 2-(pyridin-3-yl)ethan-1-ol (200 mg, 1.62 mmol) in DMF (5 mL). The reaction mixture was stirred at 0 °C for 30 min before addition of **17b** (733 mg, 3.24 mmol), and the new resulting mixture was stirred for 16 h at 100 °C. The reaction was then cooled to 0 °C and quenched with H_2_O (20 mL). The aqueous phase was extracted with EtOAc (3 × 30 mL), and the combined organic phase was washed with H_2_O (3 × 30 mL) and brine (30 mL), dried (Na_2_SO_4_), filtered, and concentrated *in vacuo* to afford title compound **17a** (55 mg, crude), which was taken into next step without purification.

#### (±)-3-(2-((1,2,3,4-Tetrahydronaphthalen-1-yl)oxy)ethyl)pyridine 1-oxide (**17**)

Following the general procedure B′, **17** was obtained from **17a** (55 mg, 0.217 mmol). Purification by flash column chromatography (5% MeOH in CH_2_Cl_2_) afforded the title compound as a white solid (48 mg, 82%): ^1^H NMR (400 MHz, CDCl_3_) δ 8.16 (p, *J* = 1.0 Hz, 1H), 8.13–8.06 (m, 1H), 7.23–7.10 (m, 5H), 7.08 (dd, *J* = 7.5, 1.5 Hz, 1H), 4.38 (t, *J* = 4.6 Hz, 1H), 3.81 (dt, *J* = 9.2, 6.2 Hz, 1H), 3.70 (dt, *J* = 9.2, 6.3 Hz, 1H), 2.84 (t, *J* = 6.2 Hz, 2H), 2.78 (q, *J* = 5.8 Hz, 1H), 2.68 (ddd, *J* = 16.9, 7.9, 5.4 Hz, 1H), 1.99–1.78 (m, 3H), 1.76–1.62 (m, 1H); ^13^C NMR (101 MHz, CDCl_3_) δ 139.6, 139.2, 137.6, 137.2, 136.3, 129.2, 129.2, 127.8, 127.6, 125.8, 125.4, 76.1, 67.6, 33.7, 29.1, 28.1, 18.8; *m*/*z* LRMS (ESI^+^) 270 [M+H]^+^; HRMS (ESI^+^) C_17_H_19_NO_2_ [M+H]^+^ calcd 270.1489, found 270.1488.

#### (±)-2,3-Dihydro-1*H*-inden-1-yl methanesulfonate (**18b**)

To an ice-cold solution of 2,3-dihydro-1*H*-inden-1-ol (500 mg, 3.73 mmol) in CH_2_Cl_2_ (10 mL) were added sequentially Et_3_N (1.3 mL, 9.33 mmol) and MsCl (0.40 mL, 5.60 mmol). The resulting solution was stirred for 16 h at rt before addition of NaHCO_3_ (aq. sat. sol., 20 mL) and EtOAc (25 mL). The aqueous phase was extracted with EtOAc (2 × 25 mL), and the combined organic phase was washed with H_2_O (20 mL) and brine (20 mL), dried (Na_2_SO_4_), filtered, and concentrated *in vacuo* to afford the title compound **18b** as a yellow oil (470 mg, 60%), which was taken into the next step without purification.

#### (±)-3-(2-((2,3-Dihydro-1*H*-inden-1-yl)oxy)ethyl)pyridine (**18a**)

To a 0 °C cooled solution of 2-(pyridin-3-yl)ethan-1-ol (200 mg, 1.62 mmol) in DMF (5 mL) was added NaH (97 mg, 2.43 mmol). The reaction mixture was stirred at 0 °C for 30 min before addition of **18b** (516 mg, 2.43 mmol), and the resulting mixture stirred for 16 h at 100 °C. The reaction was cooled back to 0 °C and carefully quenched by addition of H_2_O (20 mL). The aqueous phase was extracted with EtOAc (3 × 30 mL), and the combined organic phase was washed with H_2_O (3 × 30 mL) and brine (30 mL), dried (Na_2_SO_4_), filtered, and concentrated *in vacuo* to give 3-(2-((2,3-dihydro-1*H*-inden-1-yl)oxy)ethyl)pyridine **18a** (112 mg, crude) as colorless oil, which was used without further purification in the next step.

#### (±)-3-(2-((2,3-Dihydro-1*H*-inden-1-yl)oxy)ethyl)pyridine 1-oxide (**18**)

Following general procedure B, **18** was obtained from **18a** (20 mg, 0.084 mmol). Purification by flash column chromatography (5% MeOH in CH_2_Cl_2_) afforded the title compound as a white solid (17 mg, 81%): ^1^H NMR (400 MHz, CDCl_3_) δ 8.20 (s, 1H), 8.13 (td, *J* = 3.8, 1.7 Hz, 1H), 7.31 (d, *J* = 7.3 Hz, 1H), 7.26 (dd, *J* = 3.9, 1.0 Hz, 2H), 7.24–7.17 (m, 3H), 4.90 (dd, *J* = 6.6, 4.1 Hz, 1H), 3.76 (t, *J* = 6.3 Hz, 2H), 3.06 (ddd, *J* = 16.1, 8.4, 6.0 Hz, 1H), 2.90–2.77 (m, 3H), 2.33 (ddt, *J* = 13.0, 8.5, 6.3 Hz, 1H), 2.03 (dddd, *J* = 13.4, 8.3, 5.3, 4.1 Hz, 1H); ^13^C NMR (101 MHz, CDCl_3_) δ 144.1, 142.4, 139.2, 128.7, 128.7, 128.0, 126.5, 126.5, 125.5, 125.1, 125.1, 83.7, 67.5, 33.6, 32.4, 30.3; *m*/*z* LRMS (ESI^+^) 256 [M+H]^+^; HRMS (ESI^+^) C_16_H_17_NO_2_ [M+H]^+^ calcd 256.1332, found 256.1330; HPLC 95% (AUC), *t*_R_ = 3.6 min.

#### 3-(2-(4-Chlorophenoxy)ethyl)pyridine (**19a**)

Following general procedure A, **19a** was obtained from 4-chlorophenol (121 mg, 0.84 mmol) and 2-(pyridin-3-yl)ethan-1-ol (69 mg, 0.56 mmol). Purification by flash column chromatography (2% MeOH in CH_2_Cl_2_) afforded the title compound as a transparent oil (42 mg, 32%): ^1^H NMR (400 MHz, CDCl_3_) δ 8.58 (d, *J* = 2.2 Hz, 1H), 8.51 (dd, *J* = 4.9, 1.6 Hz, 1H), 7.69 (dt, *J* = 7.8, 2.0 Hz, 1H), 7.31 (ddd, *J* = 7.8, 4.9, 0.9 Hz, 1H), 7.25–7.19 (m, 2H), 6.85–6.75 (m, 2H), 4.16 (t, *J* = 6.5 Hz, 2H), 3.11 (t, *J* = 6.5 Hz, 2H); ^13^C NMR (101 MHz, CDCl_3_) δ 157.2, 149.5, 147.2, 137.6, 134.5, 129.5, 129.5, 126.1, 123.9, 116.0, 116.0, 68.2, 33.1; *m*/*z* LRMS (ESI^+^) 236 [M(^37^Cl)+H]^+^ 234 [M(^35^Cl)+H]^+^; HRMS (ESI^+^) C_13_H_12_^35^ClNO [M+H]^+^ calcd 234.0680, found 234.0679.

#### 3-(2-(4-Chlorophenoxy)ethyl)pyridine 1-oxide (**19**)

Following general procedure A, **19** was obtained from **19a** (30 mg, 0.129 mmol). Purification by flash column chromatography (6% MeOH in CH_2_Cl_2_) afforded the title compound as a white solid (23 mg, 72%): ^1^H NMR (400 MHz, CDCl_3_) δ 8.30 (s, 1H), 8.20 (dt, *J* = 5.9, 1.7 Hz, 1H), 7.33–7.27 (m, 2H), 7.26–7.20 (m, 2H), 6.83–6.76 (m, 2H), 4.16 (t, *J* = 6.1 Hz, 2H), 3.07 (t, *J* = 6.0 Hz, 2H); ^13^C NMR (101 MHz, CDCl_3_) δ 156.9, 139.7, 138.3, 137.7, 129.6, 129.6, 128.4, 126.4, 125.9, 115.9, 115.9, 67.2, 32.8; *m*/*z* LRMS (ESI^+^) 252 [M(^37^Cl)+H]^+^ 250 [M(^35^Cl)+H]^+^; HRMS (ESI^+^) C_13_H_12_^35^ClNO_2_ [M+H]^+^ calcd 250.0629, found 250.0628; HPLC 97% (AUC), *t*_R_ = 4.1 min.

#### 3-(2-(4-Bromophenoxy)ethyl)pyridine (**20a**)

Following general procedure A, **20a** was obtained from 4-bromophenol (259 mg, 1.50 mmol) and 2-(pyridin-3-yl)ethan-1-ol (123 mg, 1.00 mmol). Purification by flash column chromatography (15% EtOAc in CH_2_Cl_2_) afforded the title compound as a yellow oil (83 mg, 30%): ^1^H NMR (400 MHz, CDCl_3_) δ 8.55 (s, 1H), 8.50 (d, *J* = 4.2 Hz, 1H), 7.65–7.58 (m, 1H), 7.38–7.32 (m, 2H), 7.27–7.22 (m, 1H), 6.79–6.70 (m, 2H), 4.14 (t, *J* = 6.5 Hz, 2H), 3.08 (t, *J* = 6.5 Hz, 2H); ^13^C NMR (101 MHz, CDCl_3_) δ 157.8, 150.3, 148.1, 136.7, 133.9, 132.4, 132.4, 123.6, 116.5, 116.5, 113.3, 68.3, 33.0; *m*/*z* LRMS (ESI^+^) 280 [M(^81^Br)+H]^+^ 278 [M(^79^Br)+H]^+^; HRMS (ESI^+^) C_13_H_12_^79^BrNO [M+H]^+^ calcd 278.0175, found 278.0175.

#### 3-(2-(4-Bromophenoxy)ethyl)pyridine 1-oxide (**20**)

Following general procedure B, **20** was obtained from **20a** (60 mg, 0.220 mmol). Purification by flash column chromatography (6% MeOH in CH_2_Cl_2_) afforded the title compound as a white solid (39 mg, 60%): ^1^H NMR (400 MHz, CDCl_3_) δ 8.18 (s, 1H), 8.12–8.05 (m, 1H), 7.38–7.29 (m, 2H), 7.22–7.16 (m, 2H), 6.76–6.68 (m, 2H), 4.12 (t, *J* = 6.1 Hz, 2H), 3.01 (t, *J* = 6.1 Hz, 2H); ^13^C NMR (101 MHz, CDCl_3_) δ 157.4, 139.6, 137.9, 137.6, 132.4, 132.4, 127.0, 125.7, 116.4, 116.4, 113.5, 67.2, 32.7; *m*/*z* LRMS (ESI^+^) 296 [M(^81^Br)+H]^+^ 294 [M(^79^Br)+H]^+^; HRMS (ESI^+^) C_13_H_12_^79^BrNO_2_ [M+H]^+^ calcd 294.0124, found 294.0128; HPLC 99% (AUC), *t*_R_ = 4.1 min.

#### 3-(2-(4-Iodophenoxy)ethyl)pyridine (**21a**)

Following general procedure A, **21a** was obtained from 4-iodophenol (330 mg, 1.50 mmol) and 2-(pyridin-3-yl)ethan-1-ol (123 mg, 1.00 mmol). Purification by flash column chromatography (2% MeOH in CH_2_Cl_2_) afforded the title compound as a yellow solid (198 mg, 61%): ^1^H NMR (400 MHz, CDCl_3_) δ 8.63–8.32 (m, 2H), 7.63 (dt, *J* = 7.8, 2.0 Hz, 1H), 7.56–7.52 (m, 2H), 7.51–7.45 (m, 1H), 6.67–6.58 (m, 2H), 4.15 (t, *J* = 6.5 Hz, 2H), 3.09 (t, *J* = 6.5 Hz, 2H); ^13^C NMR (101 MHz, CDCl_3_) δ 150.3, 148.0, 138.5, 138.4, 138.4, 136.8, 134.0, 123.6, 118.2, 117.1, 117.1, 68.1, 33.0; *m*/*z* LRMS (ESI^+^) 326 [M+H]^+^; HRMS (ESI^+^) C_13_H_12_INO [M+H]^+^ calcd 326.0036, found 326.0027.

#### 3-(2-(4-Cyanophenoxy)ethyl)pyridine 1-oxide (**21**)

Following general procedure B, **21** was obtained from **21a** (30 mg, 0.092 mmol). Purification by flash column chromatography (4% MeOH in CH_2_Cl_2_) afforded the title compound as a yellow solid (21 mg, 67%): ^1^H NMR (400 MHz, CDCl_3_) δ 8.18 (s, 1H), 8.10–8.06 (m, 1H), 7.55–7.47 (m, 2H), 7.21–7.16 (m, 2H), 6.67–6.58 (m, 2H), 4.12 (t, *J* = 6.1 Hz, 2H), 3.01 (t, *J* = 6.1 Hz, 2H); ^13^C NMR (101 MHz, CDCl_3_) δ 158.2, 139.6, 138.4, 138.4, 137.8, 137.6, 126.8, 125.7, 116.9, 116.9, 83.5, 67.0, 32.6; *m*/*z* LRMS (ESI^+^) 341 [M+H]^+^; HRMS (ESI^+^) C_13_H_12_INO_2_ [M+H]^+^ calcd 341.9985, found 341.9987; HPLC 96% (AUC), *t*_R_ = 4.9 min.

#### 4-(2-(Pyridin-3-yl)ethoxy)benzonitrile (**22a**)

Following general procedure A, **22a** was obtained from 4-hydroxybenzonitrile (180 mg, 1.50 mmol) and 2-(pyridin-3-yl)ethan-1-ol (123 mg, 1.00 mmol). Purification by flash column chromatography (5% EtOAc in pentane) afforded the title compound as a yellow solid (150 mg, 67%): ^1^H NMR (400 MHz, CDCl_3_) δ 8.58–8.53 (m, 1H), 8.49 (dd, *J* = 4.8, 1.6 Hz, 1H), 7.65–7.58 (m, 1H), 7.58–7.52 (m, 2H), 7.25 (ddd, *J* = 7.8, 4.8, 0.9 Hz, 1H), 6.95–6.88 (m, 2H), 4.21 (t, *J* = 6.5 Hz, 2H), 3.11 (t, *J* = 6.5 Hz, 2H); ^13^C NMR (101 MHz, CDCl_3_) δ 161.9, 150.3, 148.2, 136.7, 134.1, 134.1, 133.5, 123.6, 119.2, 115.3, 115.3, 104.4, 68.3, 32.8; *m*/*z* LRMS (ESI^+^) 225 [M+H]^+^; HRMS (ESI^+^) C_14_H_12_N_2_O [M+H]^+^ calcd 225.1022, found 225.1025.

#### 3-(2-(4-Cyanophenoxy)ethyl)pyridine 1-oxide (**22**)

Following general procedure B, **22** was obtained from **22a** (60 mg, 0.270 mmol). Purification by flash column chromatography (6% MeOH in CH_2_Cl_2_) afforded the title compound as a yellow solid (56 mg, 86%): ^1^H NMR (400 MHz, CDCl_3_) δ 8.23 (s, 1H), 8.13 (dt, *J* = 5.7, 1.8 Hz, 1H), 7.60–7.54 (m, 2H), 7.28–7.19 (m, 2H), 6.95–6.88 (m, 2H), 4.23 (t, *J* = 6.1 Hz, 2H), 3.08 (t, *J* = 6.1 Hz, 2H); ^13^C NMR (101 MHz, CDCl_3_) δ 161.6, 139.7, 137.8, 137.6, 134.2, 134.2, 127.1, 125.9, 119.1, 115.3, 115.3, 104.8, 67.3, 32.6; *m*/*z* LRMS (ESI^+^) 241 [M+H]^+^; HRMS (ESI^+^) C_14_H_12_N_2_O_2_ [M+H]^+^ calcd 241.0972, found 241.0974; HPLC 96% (AUC), *t*_R_ = 3.0 min.

#### 3-(2-(4-Nitrophenoxy)ethyl)pyridine (**23a**)

Following general procedure A, **23a** was obtained from 4-nitrophenol (209 mg, 1.50 mmol) and 2-(pyridin-3-yl)ethan-1-ol (123 mg, 1.00 mmol). Purification by flash column chromatography (15% EtOAc in CH_2_Cl_2_) afforded the title compound as a white solid (110 mg, 45%): ^1^H NMR (400 MHz, CDCl_3_) δ 8.23 (d, *J* = 1.8 Hz, 1H), 8.18–8.09 (m, 3H), 7.24 (dd, *J* = 4.1, 2.0 Hz, 2H), 6.95–6.88 (m, 2H), 4.27 (t, *J* = 6.1 Hz, 2H), 3.09 (t, *J* = 6.1 Hz, 2H); ^13^C NMR (101 MHz, CDCl_3_) δ 163.6, 150.3, 148.3, 141.8, 136.7, 133.4, 126.0, 126.0, 123.6, 114.6, 114.6, 68.8, 32.8; *m*/*z* LRMS (ESI^+^) 245 [M+H]^+^; HRMS (ESI^+^) C_13_H_12_N_2_O_3_ [M+H]^+^ calcd 245.0921, found 245.0922.

#### 3-(2-(4-nitrophenoxy)ethyl)pyridine 1-oxide (**23**)

Following general procedure B, **23** was obtained from **23a** (60 mg, 0.246 mmol). Purification by flash column chromatography (6% MeOH in CH_2_Cl_2_) afforded the title compound as a white solid (48 mg, 75%): ^1^H NMR (400 MHz, CDCl_3_) δ 8.23 (d, *J* = 1.8 Hz, 1H), 8.18–8.09 (m, 3H), 7.24 (dd, *J* = 4.1, 2.0 Hz, 2H), 6.95–6.88 (m, 2H), 4.27 (t, *J* = 6.1 Hz, 2H), 3.09 (t, *J* = 6.1 Hz, 2H); ^13^C NMR (101 MHz, CDCl_3_) δ 163.2, 142.0, 139.6, 137.8, 137.4, 127.1, 126.0, 126.0, 125.9, 114.5, 114.5, 67.7, 32.5; *m*/*z* LRMS (ESI^+^) 261 [M+H]^+^; HRMS (ESI^+^) C_13_H_12_N_2_O_4_ [M+H]^+^ calcd 261.0870, found 261.0870; HPLC 97% (AUC), *t*_R_ = 3.3 min.

#### 3-(2-(4-Chloro-2,3-dimethylphenoxy)ethyl)pyridine (**24a**)

Following general procedure A, **24a** was obtained from 4-chloro-2,3-dimethylphenol (190 mg, 1.22 mmol) and 2-(pyridin-3-yl)ethan-1-ol (100 mg, 0.813 mmol). Purification by flash column chromatography (15% EtOAc in CH_2_Cl_2_) afforded the title compound as a yellow oil (55 mg, 26%): ^1^H NMR (600 MHz, CDCl_3_) δ 8.58 (d, *J* = 2.2 Hz, 1H), 8.50 (dd, *J* = 4.9, 1.6 Hz, 1H), 7.66 (dt, *J* = 7.9, 2.0 Hz, 1H), 7.32–7.21 (m, 1H), 7.12 (d, *J* = 8.8 Hz, 1H), 6.60 (d, *J* = 8.8 Hz, 1H), 4.14 (t, *J* = 6.3 Hz, 2H), 3.11 (t, *J* = 6.3 Hz, 2H), 2.30 (s, 3H), 2.12 (s, 3H); ^13^C NMR (101 MHz, CDCl_3_) δ 155.1, 150.0, 147.6, 137.2, 135.7, 134.5, 127.3, 126.7, 126.5, 123.6, 109.9, 68.5, 33.3, 16.9, 12.8; *m*/*z* LRMS (ESI^+^) 264 [M(^37^Cl)+H]^+^ 262 [M(^35^Cl)+H]^+^; HRMS (ESI^+^) C_15_H_16_ClNO [M+H]^+^ calcd 262.0993, found 262.0993.

#### 3-(2-(4-Chloro-2,3-dimethylphenoxy)ethyl)pyridine 1-oxide (**24**)

Following general procedure B, **24** was obtained from **24a** (30 mg, 0.115 mmol). Purification by flash column chromatography (5% MeOH in CH_2_Cl_2_) afforded the title compound as a transparent oil (14 mg, 44%): ^1^H NMR (400 MHz, CDCl_3_) δ 8.21 (dt, *J* = 1.9, 1.0 Hz, 1H), 8.12 (td, *J* = 3.9, 1.8 Hz, 1H), 7.24–7.20 (m, 2H), 7.13 (d, *J* = 8.7 Hz, 1H), 6.59 (d, *J* = 8.7 Hz, 1H), 4.14 (t, *J* = 6.1 Hz, 2H), 3.07 (t, *J* = 6.0 Hz, 2H), 2.30 (s, 3H), 2.13 (s, 3H); ^13^C NMR (101 MHz, CDCl_3_) δ 154.8, 139.8, 138.2, 137.6, 135.8, 127.3, 127.1, 126.9, 126.5, 125.7, 109.8, 67.6, 33.0, 16.9, 12.9; *m*/*z* LRMS (ESI^+^) 280 [M(^37^Cl)+H]^+^ 278 [M(^35^Cl)+H]^+^; HRMS (ESI^+^) C_15_H_16_^35^ClNO_2_ [M+H]^+^ calcd 278.0942, found 278.0944; HPLC 93% (AUC), *t*_R_ = 6.0 min.

#### 3-(2-(2,3-Dichlorophenoxy)ethyl)pyridine (**25a**)

Following general procedure A, **25a** was obtained from 2,3-dichlorophenol (437 mg, 2.68 mmol) and 2-(pyridin-3-yl)ethan-1-ol (300 mg, 2.44 mmol). Purification by flash column chromatography (60% EtOAc in pentane) afforded the title compound as a white solid (220 mg, 34%): ^1^H NMR (400 MHz, CDCl_3_) δ 8.57 (d, *J* = 2.6 Hz, 1H), 8.47 (dd, *J* = 4.9, 1.7 Hz, 1H), 7.73–7.67 (m, 1H), 7.23 (ddd, *J* = 7.8, 4.8, 0.9 Hz, 1H), 7.07 (t, *J* = 8.1 Hz, 1H), 7.02 (dd, *J* = 8.2, 1.6 Hz, 1H), 6.74 (dd, *J* = 8.1, 1.6 Hz, 1H), 4.18 (t, *J* = 6.3 Hz, 2H), 3.11 (t, *J* = 6.3 Hz, 2H); ^13^C NMR (101 MHz, CDCl_3_) δ 155.5, 150.3, 148.1, 137.1, 133.9, 133.7, 127.4, 123.4, 122.5, 122.0, 111.0, 69.4, 32.9; *m*/*z* LRMS (ESI^+^) 268 [M+H]^+^; HRMS (ESI^+^) C_13_H_11_^35^Cl_2_NO [M+H]^+^ calcd 268.0290, found 268.0288.

#### 3-(2-(2,3-Dichlorophenoxy)ethyl)pyridine 1-oxide (**25**)

Following general procedure B, **25** was obtained from **25a** (140 mg, 0.522 mmol). Purification by flash column chromatography (6% MeOH in CH_2_Cl_2_) afforded the title compound as a white solid (140 mg, 95%): ^1^H NMR (400 MHz, CDCl_3_) δ 8.23 (d, *J* = 1.8 Hz, 1H), 8.09 (dt, *J* = 6.4, 1.4 Hz, 1H), 7.32 (dt, *J* = 7.9, 1.3 Hz, 1H), 7.20 (dd, *J* = 7.9, 6.4 Hz, 1H), 7.08 (t, *J* = 8.1 Hz, 1H), 7.02 (dd, *J* = 8.2, 1.6 Hz, 1H), 6.74 (dd, *J* = 8.1, 1.6 Hz, 1H), 4.18 (t, *J* = 5.9 Hz, 2H), 3.07 (t, *J* = 5.9 Hz, 2H); ^13^C NMR (101 MHz, CDCl_3_) δ 155.2, 139.6, 137.7, 137.6, 134.0, 127.7, 127.4, 125.7, 122.8, 122.0, 111.0, 68.5, 32.7; *m*/*z* LRMS (ESI^+^) 284 [M+H]^+^; HRMS (ESI^+^) C_13_H_11_^35^Cl_2_NO_2_ [M+H]^+^ calcd 284.0240, found 284.0240; HPLC 99% (AUC), *t*_R_ = 5.0 min.

#### 3-(2-(4-Chloro-2-iodophenoxy)ethyl)pyridine (**26a**)

Following general procedure A, **26a** was obtained from 4-chloro-2-iodophenol (453 mg, 1.78 mmol) and 2-(pyridin-3-yl)ethan-1-ol (200 mg, 1.62 mmol). Purification by flash column chromatography (60% EtOAc in pentane) afforded the title compound as a white solid (390 mg, 67%): ^1^H NMR (400 MHz, CDCl_3_) δ 8.63 (d, *J* = 2.4 Hz, 1H), 8.50 (dd, *J* = 4.9, 1.7 Hz, 1H), 7.77 (dt, *J* = 7.9, 2.0 Hz, 1H), 7.71 (d, *J* = 2.5 Hz, 1H), 7.27 (ddd, *J* = 7.8, 4.9, 0.9 Hz, 1H), 7.22 (dd, *J* = 8.8, 2.5 Hz, 1H), 6.65 (d, *J* = 8.8 Hz, 1H), 4.17 (t, *J* = 6.2 Hz, 2H), 3.15 (t, *J* = 6.2 Hz, 2H); ^13^C NMR (101 MHz, CDCl_3_) δ 156.2, 150.4, 147.9, 138.8, 137.5, 133.9, 129.3, 126.8, 123.6, 112.4, 86.7, 69.7, 33.0; *m*/*z* LRMS (ESI^+^) 360 [M+H]^+^; HRMS (ESI^+^) C_13_H_11_^35^ClINO [M+H]^+^ calcd 359.9647, found 359.9642.

#### 3-(2-(4-Chloro-2-iodophenoxy)ethyl)pyridine 1-oxide (**26**)

Following general procedure B, **26** was obtained from **26a** (100 mg, 0.278 mmol). Purification by flash column chromatography (6% MeOH in CH_2_Cl_2_) afforded the title compound as a white solid (83 mg, 80%): ^1^H NMR (400 MHz, CDCl_3_) δ 8.32 (d, *J* = 2.0 Hz, 1H), 8.14 (d, *J* = 6.4 Hz, 1H), 7.69 (d, *J* = 2.4 Hz, 1H), 7.40 (s, 1H), 7.28–7.17 (m, 2H), 6.64 (d, *J* = 8.7 Hz, 1H), 4.16 (t, *J* = 5.8 Hz, 2H), 3.09 (t, *J* = 5.8 Hz, 2H); ^13^C NMR (101 MHz, CDCl_3_) δ 155.9, 140.0, 138.9, 137.9, 137.7, 129.3, 128.4, 127.2, 125.8, 112.5, 86.7, 68.8, 32.8; *m*/*z* LRMS (ESI^+^) 376 [M+H]^+^; HRMS (ESI^+^) C_13_H_11_^35^ClINO_2_ [M+H]^+^ calcd 375.9596, found 375.9594; HPLC 97% (AUC), *t*_R_ = 6.1 min.

#### 3-(2-(4-Chloro-2-cyclohexylphenoxy)ethyl)pyridine (**27a**)

Following general procedure A, **27a** was obtained from 4-chloro-2-cyclohexylphenol (374 mg, 1.78 mmol) and 2-(pyridin-3-yl)ethan-1-ol (200 mg, 1.62 mmol). Purification by flash column chromatography (50% EtOAc in pentane) afforded the title compound as a white solid (230 mg, 45%). The crude product was taken into next step without purification.

#### 3-(2-(4-Chloro-2-cyclohexylphenoxy)ethyl)pyridine 1-oxide (**27**)

Following general procedure B, **27** was obtained from **27a** (100 mg, 0.317 mmol). Purification by flash column chromatography (6% MeOH in CH_2_Cl_2_) afforded the title compound as a white solid (63 mg, 60%): ^1^H NMR (400 MHz, CDCl_3_) δ 8.21 (d, *J* = 1.9 Hz, 1H), 8.13 (dt, *J* = 5.5, 1.8 Hz, 1H), 7.28–7.21 (m, 2H), 7.10 (d, *J* = 2.6 Hz, 1H), 7.06 (dd, *J* = 8.6, 2.7 Hz, 1H), 6.69 (d, *J* = 8.7 Hz, 1H), 4.15 (t, *J* = 6.0 Hz, 2H), 3.07 (t, *J* = 6.0 Hz, 2H), 2.76 (tt, *J* = 11.4, 3.2 Hz, 1H), 1.85–1.65 (m, 4H), 1.41–1.15 (m, 6H); ^13^C NMR (101 MHz, CDCl_3_) δ 154.1, 139.6, 138.3, 138.2, 137.6, 127.3, 127.1, 127.1, 126.4, 126.2, 126.2, 125.6, 112.4, 67.5, 37.2, 33.1, 33.0, 27.1, 26.3; *m*/*z* LRMS (ESI^+^) 332 [M+H]^+^; HRMS (ESI^+^) C_19_H_22_^35^ClNO_2_ [M+H]^+^ calcd 332.1412, found 332.1410; HPLC 96% (AUC), *t*_R_ = 7.7 min.

#### 3-(2-((5-Chloro-[1,1′-biphenyl]-2-yl)oxy)ethyl)pyridine (**28a**)

A mixture of **26a** (150 mg, 0.417 mmol), phenylboronic acid (61 mg, 0.500 mmol), and K_2_CO_3_ (144 mg, 1.04 mmol) in 1,4-dioxane (3 mL) and water (1 mL) was degassed with N_2_ for 5 min before addition of Pd(PPh_3_)_4_ (48 mg, 0.042 mmol). The mixture was heated in a sealed tube at 100 °C for 16 h. After completion, the mixture was cooled to rt, diluted with EtOAc, filtered through Celite (EtOAc), and concentrated *in vacuo*. The residue was purified by flash column chromatography (50% EtOAc in pentane) to afford title compound **28a** as a white solid (99 mg, 77%): ^1^H NMR (400 MHz, CDCl_3_) δ 8.46 (dd, *J* = 4.8, 1.7 Hz, 1H), 8.41 (d, *J* = 2.3 Hz, 1H), 7.43–7.32 (m, 6H), 7.27 (d, *J* = 2.7 Hz, 1H), 7.22 (dd, *J* = 8.7, 2.7 Hz, 1H), 7.12 (ddd, *J* = 7.8, 4.8, 0.9 Hz, 1H), 6.84 (d, *J* = 8.8 Hz, 1H), 4.13 (t, *J* = 6.2 Hz, 2H), 2.97 (t, *J* = 6.2 Hz, 2H); ^13^C NMR (101 MHz, CDCl_3_) δ 154.3, 150.3, 147.9, 137.2, 136.9, 134.0, 132.8, 130.7, 129.6, 129.6, 128.2, 128.1, 128.1, 127.5, 126.2, 123.3, 113.6, 69.0, 33.0; *m*/*z* LRMS (ESI^+^) 310 [M+H]^+^; HRMS (ESI^+^) C_19_H_16_^35^ClNO [M+H]^+^ calcd 310.0993, found 310.0986.

#### 3-(2-((5-Chloro-[1,1′-biphenyl]-2-yl)oxy)ethyl)pyridine 1-oxide (**28**)

Following general procedure B, **28** was obtained from 3-(2-((5-chloro-[1,1′-biphenyl]-2-yl)oxy)ethyl)pyridine **28a** (40 mg, 0.129 mmol). Purification by flash column chromatography (6% MeOH in CH_2_Cl_2_) afforded the title compound as a white solid (37 mg, 88%): ^1^H NMR (400 MHz, CDCl_3_) δ 8.04 (dd, *J* = 6.1, 1.2 Hz, 2H), 7.42–7.30 (m, 5H), 7.26–7.19 (m, 2H), 7.03 (ddd, *J* = 7.9, 5.9, 1.1 Hz, 1H), 6.90 (dt, *J* = 8.0, 1.3 Hz, 1H), 6.82 (d, *J* = 8.7 Hz, 1H), 4.10 (t, *J* = 5.9 Hz, 2H), 2.89 (t, *J* = 5.9 Hz, 2H); ^13^C NMR (101 MHz, CDCl_3_) δ 153.9, 139.4, 137.9, 137.4, 137.0, 132.9, 130.7, 129.5, 128.2, 128.2, 128.2, 128.1, 127.6, 127.5, 126.5, 125.5, 113.9, 68.2, 32.7; *m*/*z* LRMS (ESI^+^) 326 [M+H]^+^; HRMS (ESI^+^) C_19_H_16_^35^ClNO_2_ [M+H]^+^ calcd 326.0942, found 326.0941; HPLC 99% (AUC), *t*_R_ = 6.5 min.

#### 3-(2-(2,3,4-Trichlorophenoxy)ethyl)pyridine (**29a**)

Following general procedure A, **29a** was obtained from 2,3,4-trichlorophenol (100 mg, 0.507 mmol) and 2-(pyridin-3-yl)ethan-1-ol (75 mg, 0.608 mmol). Purification by flash column chromatography (60% EtOAc in pentane) afforded the title compound as a white solid (60 mg, 39%): ^1^H NMR (400 MHz, CDCl_3_) δ 8.58 (dd, *J* = 2.3, 0.8 Hz, 1H), 8.50 (dd, *J* = 4.8, 1.7 Hz, 1H), 7.74–7.64 (m, 1H), 7.28 (d, *J* = 9.0 Hz, 1H), 7.25 (ddd, *J* = 7.8, 4.8, 0.9 Hz, 1H), 6.74 (d, *J* = 9.0 Hz, 1H), 4.21 (t, *J* = 6.3 Hz, 2H), 3.15 (t, *J* = 6.3 Hz, 2H); ^13^C NMR (101 MHz, CDCl_3_) δ 154.1, 150.5, 148.4, 137.0, 133.5, 132.7, 128.0, 125.7, 123.8, 123.5, 111.5, 69.8, 33.0; *m*/*z* LRMS (ESI^+^) 302 [M+H]^+^; HRMS (ESI^+^) C_13_H_10_^35^Cl_3_NO [M+H]^+^ calcd 301.9901, found 301.9901.

#### 3-(2-(2,3,4-Trichlorophenoxy)ethyl)pyridine 1-oxide (**29**)

Following general procedure B, **29** was obtained from **29a** (60 mg, 0.198 mmol). Purification by flash column chromatography (5% MeOH in CH_2_Cl_2_) afforded the title compound as a white solid (38.0 mg, 60%): ^1^H NMR (400 MHz, CDCl_3_) δ 8.23 (t, *J* = 1.7 Hz, 1H), 8.11 (dt, *J* = 6.3, 1.4 Hz, 1H), 7.35–7.26 (m, 2H), 7.22 (dd, *J* = 7.9, 6.3 Hz, 1H), 6.73 (d, *J* = 8.9 Hz, 1H), 4.20 (t, *J* = 5.9 Hz, 2H), 3.10 (t, *J* = 5.9 Hz, 2H); ^13^C NMR (101 MHz, CDCl_3_) δ 153.8, 139.7, 137.8, 137.5, 132.8, 128.1, 127.3, 126.1, 125.8, 123.8, 111.5, 68.8, 32.8; *m*/*z* LRMS (ESI^+^) 317 [M+H]^+^; HRMS (ESI^+^) C_13_H_10_^35^Cl_3_NO_2_ [M+H]^+^ calcd 317.9850, found 317.9847; HPLC 98% (AUC), *t*_R_ = 6.1 min.

#### 3-(2-(4-Chloro-3,5-dimethylphenoxy)ethyl)pyridine (**30a**)

Following general procedure A, **30a** was obtained from 4-chloro-3,5-dimethylphenol (279 mg, 1.78 mmol) and 2-(pyridin-3-yl)ethan-1-ol (200 mg, 1.62 mmol). Purification by flash column chromatography (60% EtOAc in pentane) afforded the title compound as a white solid (168 mg, 40%): ^1^H NMR (400 MHz, CDCl_3_) δ 8.55 (d, *J* = 2.3 Hz, 1H), 8.49 (dd, *J* = 4.9, 1.7 Hz, 1H), 7.61 (dt, *J* = 7.8, 2.0 Hz, 1H), 7.23 (ddd, *J* = 7.8, 4.9, 0.8 Hz, 1H), 6.61 (s, 2H), 4.13 (t, *J* = 6.5 Hz, 2H), 3.06 (t, *J* = 6.5 Hz, 2H), 2.32 (s, 6H); ^13^C NMR (101 MHz, CDCl_3_) δ 156.5, 150.5, 148.2, 148.2, 137.3, 136.5, 136.5, 134.0, 126.6, 123.4, 114.7, 68.2, 33.1, 21.0, 21.0; *m*/*z* LRMS (ESI^+^) 262 [M+H]^+^; HRMS (ESI^+^) C_15_H_16_^35^ClNO [M+H]^+^ calcd 262.0993, found 262.0990.

#### 3-(2-(4-Chloro-3,5-dimethylphenoxy)ethyl)pyridine 1-oxide (**30**)

Following general procedure B, **30** was obtained from **30a** (50 mg, 0.191 mmol). Purification by flash column chromatography (5% MeOH in CH_2_Cl_2_) afforded the title compound as a white solid (48 mg, 91%): ^1^H NMR (400 MHz, CDCl_3_) δ 8.21 (dt, *J* = 1.9, 0.9 Hz, 1H), 8.11 (td, *J* = 3.9, 1.8 Hz, 1H), 7.23–7.19 (m, 2H), 6.60 (s, 2H), 4.12 (t, *J* = 6.1 Hz, 2H), 3.02 (t, *J* = 6.1 Hz, 2H), 2.32 (d, *J* = 0.7 Hz, 6H); ^13^C NMR (101 MHz, CDCl_3_) δ 156.1, 139.7, 138.1, 137.6, 137.4, 137.4, 127.0, 126.9, 125.7, 125.7, 114.6, 67.1, 32.8, 21.1, 21.1; *m*/*z* LRMS (ESI^+^) 278 [M+H]^+^; HRMS (ESI^+^) C_15_H_16_^35^ClNO_2_ [M+H]^+^ calcd 278.0942, found 278.0940. HPLC 99% (AUC), *t*_R_ = 6.0 min.

#### 3-(2-(3,4,5-Trimethylphenoxy)ethyl)pyridine (**31a**)

Following general procedure A, **31a** was obtained from 3,4,5-trimethylphenol (242 mg, 1.78 mmol) and 2-(pyridin-3-yl)ethan-1-ol (200 mg, 1.62 mmol). Purification by flash column chromatography (60% EtOAc in pentane) afforded the title compound as a white solid (120 mg, 31%): ^1^H NMR (400 MHz, CDCl_3_) δ 8.56 (d, *J* = 2.3 Hz, 1H), 8.49 (dd, *J* = 4.8, 1.7 Hz, 1H), 7.62 (dt, *J* = 7.8, 2.0 Hz, 1H), 7.23 (ddd, *J* = 7.8, 4.8, 0.9 Hz, 1H), 6.58 (s, 2H), 4.15 (t, *J* = 6.6 Hz, 2H), 3.06 (t, *J* = 6.6 Hz, 2H), 2.25 (s, 6H), 2.10 (s, 3H); ^13^C NMR (101 MHz, CDCl_3_) δ 156.0, 150.5, 148.0, 148.0, 137.6, 136.5, 134.3, 127.5, 123.4, 113.8, 113.8, 67.9, 33.1, 22.1, 20.9, 14.6; *m*/*z* LRMS (ESI^+^) 242 [M+H]^+^; HRMS (ESI^+^) C_16_H_19_NO [M+H]^+^ calcd 242.1534, found 242.1539.

#### 3-(2-(3,4,5-Trimethylphenoxy)ethyl)pyridine 1-oxide (**31**)

Following general procedure B, **31** was obtained from **31a** (90 mg, 0.373 mmol). Purification by flash column chromatography (5% MeOH in CH_2_Cl_2_) afforded the title compound as a white solid (73 mg, 76%): ^1^H NMR (400 MHz, CDCl_3_) δ 8.21 (d, *J* = 1.8 Hz, 1H), 8.10 (dt, *J* = 5.6, 1.8 Hz, 1H), 7.25–7.16 (m, 2H), 6.55 (s, 2H), 4.14 (t, *J* = 6.1 Hz, 2H), 3.01 (t, *J* = 6.1 Hz, 2H), 2.24 (s, 6H), 2.08 (s, 3H); ^13^C NMR (101 MHz, CDCl_3_) δ 155.7, 139.7, 138.4, 137.8, 137.8, 137.5, 127.9, 127.0, 127.0, 125.6, 125.6, 113.8, 66.9, 32.9, 20.9, 14.7; *m*/*z* LRMS (ESI^+^) 258 [[M+H]^+^; HRMS (ESI^+^) C_16_H_19_NO_2_ [M+H]^+^ calcd 258.1489, found 258.1487; HPLC 97% (AUC), *t*_R_ = 5.1 min.

#### 3-(2-(4-Chloro-3-iodophenoxy)ethyl)pyridine (**32a**)

Following general procedure A, **32a** was obtained from 4-chloro-3-iodophenol (453 mg, 1.78 mmol) and 2-(pyridin-3-yl)ethan-1-ol (200 mg, 1.62 mmol). Purification by flash column chromatography (60% EtOAc in pentane) afforded the title compound as a white solid (190 mg, 33%): ^1^H NMR (400 MHz, CDCl_3_) δ 8.52 (d, *J* = 2.3 Hz, 1H), 8.48 (dd, *J* = 4.9, 1.7 Hz, 1H), 7.58 (dt, *J* = 7.8, 2.0 Hz, 1H), 7.32 (d, *J* = 2.9 Hz, 1H), 7.26 (d, *J* = 8.8 Hz, 1H), 7.22 (ddd, *J* = 7.8, 4.8, 0.9 Hz, 1H), 6.78 (dd, *J* = 8.8, 2.9 Hz, 1H), 4.10 (t, *J* = 6.5 Hz, 2H), 3.04 (t, *J* = 6.5 Hz, 2H); ^13^C NMR (101 MHz, CDCl_3_) δ 157.2, 150.3, 148.2, 136.5, 133.6, 130.4, 129.4, 125.8, 123.5, 116.2, 98.1, 68.5, 32.9; *m*/*z* LRMS (ESI^+^) 359 [M+H]^+^; HRMS (ESI^+^) C_13_H_11_^35^ClINO [M+H]^+^ calcd 359.9647, found 359.9637.

#### 3-(2-(4-Chloro-3-iodophenoxy)ethyl)pyridine 1-oxide (**32**)

Following general procedure B, **32** was obtained from **32a** (60 mg, 0.167 mmol). Purification by flash column chromatography (5% MeOH in CH_2_Cl_2_) afforded the title compound as a white solid (54 mg, 86%): ^1^H NMR (400 MHz, CDCl_3_) δ 8.23 (s, 1H), 8.14 (dt, *J* = 5.3, 2.0 Hz, 1H), 7.33 (d, *J* = 2.9 Hz, 1H), 7.29 (s, 1H), 7.23 (dd, *J* = 4.1, 1.9 Hz, 2H), 6.79 (dd, *J* = 8.9, 2.9 Hz, 1H), 4.13 (t, *J* = 6.0 Hz, 2H), 3.03 (t, *J* = 6.1 Hz, 2H); ^13^C NMR (101 MHz, CDCl_3_) δ 156.9, 139.7, 137.8, 137.7, 130.8, 129.5, 127.5, 125.8, 125.8, 116.2, 98.1, 67.5, 32.7; *m*/*z* LRMS (ESI^+^) 375 [M+H]^+^; HRMS (ESI^+^) C_13_H_11_^35^ClINO_2_ [M+H]^+^ calcd 375.9596, found 375.9595; HPLC 95% (AUC), *t*_R_ = 5.8 min.

#### 3-(2-(3-Bromo-4-chlorophenoxy)ethyl)pyridine (**33a**)

Following general procedure A, **33a** was obtained from 4-chloro-3-bromophenol (369 mg, 1ss.78 mmol) and 2-(pyridin-3-yl)ethan-1-ol (200 mg, 1.62 mmol). Purification by flash column chromatography (50% EtOAc in pentane) afforded the title compound as a white solid (206 mg, 40%): ^1^H NMR (400 MHz, CDCl_3_) δ 8.58 (d, *J* = 2.2 Hz, 1H), 8.53 (dd, *J* = 5.0, 1.6 Hz, 1H), 7.67–7.58 (m, 1H), 7.33 (d, *J* = 8.9 Hz, 1H), 7.28 (ddd, *J* = 7.8, 4.8, 0.9 Hz, 1H), 7.16 (s, 1H), 6.80 (dd, *J* = 8.9, 2.9 Hz, 1H), 4.16 (t, *J* = 6.5 Hz, 2H), 3.11 (t, *J* = 6.5 Hz, 2H); ^13^C NMR (101 MHz, CDCl_3_) δ 157.6, 150.4, 148.2, 136.5, 133.5, 130.6, 126.2, 123.5, 122.6, 119.5, 115.2, 68.5, 32.9; *m*/*z* LRMS (ESI^+^) 313 [M+H]^+^; HRMS (ESI^+^) C_13_H_11_^79^Br^35^ClNO [M+H]^+^ calcd 313.9763, found 313.9763.

#### 3-(2-(3-Bromo-4-chlorophenoxy)ethyl)pyridine 1-oxide (**33**)

Following general procedure B, **33** was obtained from **33a** (140 mg, 0.448 mmol). Purification by flash column chromatography (5% MeOH in CH_2_Cl_2_) afforded the title compound as a white solid (130 mg, 88%): ^1^H NMR (400 MHz, CDCl_3_) δ 8.18 (td, *J* = 1.6, 0.8 Hz, 1H), 8.10 (dt, *J* = 5.9, 1.7 Hz, 1H), 7.29 (d, *J* = 8.9 Hz, 1H), 7.23–7.15 (m, 2H), 7.10 (s, 1H), 6.75 (dd, *J* = 8.8, 2.9 Hz, 1H), 4.12 (t, *J* = 6.1 Hz, 2H), 3.02 (t, *J* = 6.1 Hz, 2H); ^13^C NMR (101 MHz, CDCl_3_) δ 157.2, 139.6, 137.7, 137.6, 130.7, 126.8, 126.6, 125.8, 122.7, 119.5, 115.2, 67.5, 32.6; *m*/*z* LRMS (ESI^+^) 329 [M+H]^+^; HRMS (ESI^+^) C_13_H_11_^79^Br^35^ClNO_2_ [M+H]^+^ calcd 329.9712, found 329.9709; HPLC 98% (AUC), *t*_R_ = 5.5 min.

#### 3-(2-(3,4-Dichlorophenoxy)ethyl)pyridine (**34a**)

Following general procedure A, **34a** was obtained from 3,4-dichlorophenol (290 mg, 1.78 mmol) and 2-(pyridin-3-yl)ethan-1-ol (200 mg, 1.62 mmol). Purification by flash column chromatography (50% EtOAc in pentane) afforded the title compound as a colorless oil (160 mg, 37%): ^1^H NMR (400 MHz, CDCl_3_) δ 8.55 (d, *J* = 2.4 Hz, 1H), 8.50 (dd, *J* = 4.9, 1.7 Hz, 1H), 7.63 (dt, *J* = 7.8, 2.1 Hz, 1H), 7.32–7.22 (m, 2H), 6.96 (d, *J* = 2.9 Hz, 1H), 6.72 (dd, *J* = 8.9, 2.9 Hz, 1H), 4.14 (t, *J* = 6.5 Hz, 2H), 3.09 (t, *J* = 6.5 Hz, 2H); ^13^C NMR (101 MHz, CDCl_3_) δ 157.7, 150.0, 147.9, 137.0, 133.9, 133.0, 130.8, 124.4, 123.7, 116.5, 114.7, 68.5, 32.9; *m*/*z* LRMS (ESI^+^) 268 [M+H]^+^; HRMS (ESI^+^) C_13_H_11_^35^Cl_2_NO [M+H]^+^ calcd 268.0290, found 268.0288.

#### 3-(2-(3,4-Dichlorophenoxy)ethyl)pyridine 1-oxide (**34**)

Following general procedure B, **34** was obtained from **34a** (30 mg, 0.12 mmol). Purification by flash column chromatography (5% MeOH in CH_2_Cl_2_) afforded the title compound as a white solid (28 mg, 82%): ^1^H NMR (400 MHz, CDCl_3_) δ 8.25 (s, 1H), 8.21–8.11 (m, 1H), 7.29 (s, 1H), 7.27–7.23 (m, 2H), 6.96 (d, *J* = 2.9 Hz, 1H), 6.72 (dd, *J* = 8.9, 2.9 Hz, 1H), 4.15 (t, *J* = 6.1 Hz, 2H), 3.05 (t, *J* = 6.0 Hz, 2H); ^13^C NMR (101 MHz, CDCl_3_) δ 157.3, 139.7, 137.9, 137.7, 133.1, 130.9, 127.7, 125.9, 124.7, 116.4, 114.6, 67.5, 32.7; *m*/*z* LRMS (ESI^+^) 284 [M+H]^+^; HRMS (ESI^+^) C_13_H_11_^35^Cl_2_NO_2_ [M+H]^+^ calcd 284.0240, found 284.0236; HPLC 97% (AUC), *t*_R_ = 5.2 min.

#### 3-(2-(4-Chloro-3-fluorophenoxy)ethyl)pyridine (**35a**)

Following general procedure A, **35a** was obtained from 4-chloro-3-fluorophenol (260 mg, 1.78 mmol) and 2-(pyridin-3-yl)ethan-1-ol (200 mg, 1.62 mmol). Purification by flash column chromatography (50% EtOAc in pentane) afforded the title compound as a white solid (196 mg, 49%): ^1^H NMR (400 MHz, CDCl_3_) δ 8.59 (d, *J* = 2.3 Hz, 1H), 8.54 (dd, *J* = 4.8, 1.7 Hz, 1H), 7.68–7.58 (m, 1H), 7.33–7.24 (m, 2H), 6.72 (dd, *J* = 10.7, 2.8 Hz, 1H), 6.66 (ddd, *J* = 8.9, 2.8, 1.2 Hz, 1H), 4.18 (t, *J* = 6.5 Hz, 2H), 3.12 (t, *J* = 6.5 Hz, 2H); ^13^C NMR (101 MHz, CDCl_3_) δ 158.5 (d, *J* = 247.0 Hz), 158.4 (d, *J* = 10.0 Hz), 150.4, 148.3, 136.5, 133.6, 130.7, 123.5, 112.6 (d, *J* = 18.0 Hz), 111.3 (d, *J* = 4.0 Hz), 103.6 (d, *J* = 24.0 Hz), 68.6, 32.9; *m*/*z* LRMS (ESI^+^) 252 [M+H]^+^; HRMS (ESI^+^) C_13_H_11_^35^ClFNO [M+H]^+^ calcd 252.0586, found 252.0584.

#### 3-(2-(4-Chloro-3-fluorophenoxy)ethyl)pyridine 1-oxide (**35**)

Following general procedure B, **35** was obtained from **35a** (120 mg, 0.477 mmol). Purification by flash column chromatography (5% MeOH in CH_2_Cl_2_) afforded the title compound as a white solid (106 mg, 83%): ^1^H NMR (400 MHz, CDCl_3_) δ 8.20 (dq, *J* = 1.6, 0.8 Hz, 1H), 8.12 (dt, *J* = 5.9, 1.7 Hz, 1H), 7.29–7.25 (m, 1H), 7.24–7.18 (m, 2H), 6.68 (dd, *J* = 10.6, 2.8 Hz, 1H), 6.61 (ddd, *J* = 8.9, 2.9, 1.2 Hz, 1H), 4.15 (t, *J* = 6.1 Hz, 2H), 3.05 (t, *J* = 6.1 Hz, 2H); ^13^C NMR (101 MHz, CDCl_3_) δ 158.6 (d, *J* = 247.0 Hz), 158.1 (d, *J* = 10.0 Hz), 139.7, 137.7 (d, *J* = 8.0 Hz), 130.9, 126.8, 125.8, 113.1 (d, *J* = 17.0 Hz), 111.3 (d, *J* = 4.0 Hz), 103.6 (d, *J* = 25.0 Hz), 67.6, 32.6; ^19^F NMR (376 MHz, CDCl_3_) δ −112.71. *m*/*z* LRMS (ESI^+^) 268 [M+H]^+^; HRMS (ESI^+^) C_13_H_11_^35^ClFNO_2_ [M+H]^+^ calcd 268.0535, found 268.0534; HPLC 99% (AUC), *t*_R_ = 4.2 min.

#### 3-(2-((6-Chloro-[1,1′-biphenyl]-3-yl)oxy)ethyl)pyridine (**36a**)

A mixture of **32a** (120 mg, 0.330 mmol), phenylboronic acid (49 mg, 0.400 mmol), and K_2_CO_3_ (114 mg, 0.825 mmol) in 1,4-dioxane (3 mL) and water (1 mL) was degassed with N_2_ for 5 min before addition of Pd(PPh_3_)_4_ (38 mg, 0.033 mmol). The mixture was heated in a sealed tube at 100 °C for 16 h. After completion, the mixture was cooled to rt, diluted with EtOAc, filtered through Celite (EtOAc), and concentrated *in vacuo*. The residue was then purified by flash column chromatography (50% EtOAc in pentane) to afford the title compound as a white solid (57 mg, 56%): ^1^H NMR (400 MHz, CDCl_3_) δ 8.60 (d, *J* = 2.8 Hz, 1H), 8.54 (dd, *J* = 4.9, 1.6 Hz, 1H), 7.67 (dt, *J* = 7.7, 1.9 Hz, 1H), 7.44–7.36 (m, 5H), 7.34 (d, *J* = 8.7 Hz, 1H), 7.29 (ddd, *J* = 7.8, 5.0, 0.9 Hz, 1H), 6.86 (d, *J* = 3.0 Hz, 1H), 6.80 (dd, *J* = 8.7, 3.0 Hz, 1H), 4.18 (t, *J* = 6.4 Hz, 2H), 3.11 (t, *J* = 6.4 Hz, 2H); ^13^C NMR (101 MHz, CDCl_3_) δ 157.3, 149.7, 147.4, 141.6, 139.5, 137.4, 130.8, 129.4, 129.4, 128.2, 128.2, 127.9, 127.7, 124.4, 123.8, 117.4, 115.0, 68.3, 33.1; *m*/*z* LRMS (ESI^+^) 310 [M+H]^+^; HRMS (ESI^+^) C_19_H_16_^35^ClNO [M+H]^+^ calcd 310.0993, found 310.0990.

#### 3-(2-((6-Chloro-[1,1′-biphenyl]-3-yl)oxy)ethyl)pyridine 1-oxide (**36**)

Following general procedure B, **36** was obtained from **36a** (20 mg, 0.065 mmol). Purification by flash column chromatography (6% MeOH in CH_2_Cl_2_) afforded the title compound as a white solid (18 mg, 86%): ^1^H NMR (400 MHz, CDCl_3_) δ 8.24 (s, 1H), 8.18–8.08 (m, 1H), 7.46–7.37 (m, 5H), 7.35 (d, *J* = 8.7 Hz, 1H), 7.24 (d, *J* = 4.0 Hz, 2H), 6.85 (d, *J* = 3.1 Hz, 1H), 6.80 (dd, *J* = 8.8, 3.1 Hz, 1H), 4.18 (t, *J* = 6.1 Hz, 2H), 3.05 (t, *J* = 6.0 Hz, 2H); ^13^C NMR (101 MHz, CDCl_3_) δ 157.0, 141.6, 139.4, 138.0, 137.7, 130.8, 129.4, 129.4, 128.2, 128.2, 127.9, 127.8, 127.4, 125.8, 124.7, 117.3, 114.9, 67.3, 32.8; *m*/*z* LRMS (ESI^+^) 326 [M+H]^+^; HRMS (ESI^+^) C_19_H_16_^35^ClNO_2_ [M+H]^+^ calcd 326.0942, found 326.0940; HPLC 98% (AUC), *t*_R_ = 6.4 min.

#### 3-(2-(4-Chloro-3-methylphenoxy)ethyl)pyridine (**37a**)

Following general procedure A, **37a** was obtained from 4-chloro-3-methylphenol (254 mg, 1.78 mmol) and 2-(pyridin-3-yl)ethan-1-ol (200 mg, 1.62 mmol). Purification by flash column chromatography (50% EtOAc in pentane) afforded the title compound as a white solid (180 mg, 45%): ^1^H NMR (400 MHz, CDCl_3_) δ 8.54 (d, *J* = 2.4 Hz, 1H), 8.47 (dt, *J* = 4.1, 2.0 Hz, 1H), 7.59 (dt, *J* = 7.9, 2.1 Hz, 1H), 7.25–7.20 (m, 1H), 7.18 (dd, *J* = 8.6, 2.2 Hz, 1H), 6.74 (d, *J* = 3.0 Hz, 1H), 6.63 (dd, *J* = 8.7, 2.9 Hz, 1H), 4.12 (t, *J* = 6.6 Hz, 2H), 3.05 (t, *J* = 6.6 Hz, 2H), 2.30 (s, 3H); ^13^C NMR (101 MHz, CDCl_3_) δ 157.2, 150.4, 148.1, 137.1, 136.5, 133.9, 129.7, 126.2, 123.4, 117.2, 113.1, 68.2, 33.0, 20.4; *m*/*z* LRMS (ESI^+^) 248 [M+H]^+^; HRMS (ESI^+^) C_14_H_14_^35^ClNO [M+H]^+^ calcd 248.0837, found 248.0838.

#### 3-(2-(4-Chloro-3-methylphenoxy)ethyl)pyridine 1-oxide (**37**)

Following general procedure B, **37** was obtained from **37a** (50 mg, 0.21 mmol). Purification by flash column chromatography (5% MeOH in CH_2_Cl_2_) afforded the title compound as a white solid (48 mg, 87%): ^1^H NMR (400 MHz, CDCl_3_) δ 8.20 (dd, *J* = 1.8, 0.9 Hz, 1H), 8.10 (td, *J* = 3.9, 1.8 Hz, 1H), 7.22–7.16 (m, 3H), 6.75–6.70 (m, 1H), 6.62 (dd, *J* = 8.7, 3.0 Hz, 1H), 4.12 (t, *J* = 6.1 Hz, 2H), 3.01 (t, *J* = 6.1 Hz, 2H), 2.30 (s, 3H); ^13^C NMR (101 MHz, CDCl_3_) δ 156.9, 139.7, 138.0, 137.6, 137.3, 129.8, 127.0, 126.5, 125.7, 117.3, 113.1, 67.2, 32.8, 20.4; *m*/*z* LRMS (ESI^+^) 264 [M+H]^+^; HRMS (ESI^+^) C_14_H_14_^35^ClNO_2_ [M+H]^+^ calcd 264.0786, found 264.0784; HPLC 99% (AUC), *t*_R_ = 4.9 min.

#### 2-(Pyridin-3-yl)ethylmethanesulfonate (**38b**)

To an ice-cold solution of 2-(pyridin-3-yl)ethan-1-ol (200 mg, 1.62 mmol) in CH_2_Cl_2_ (5 mL) were added sequentially Et_3_N (0.60 mL, 4.05 mmol) and MsCl (0.20 mL, 2.44 mmol). The resulting solution was stirred for 16 h at rt before addition of NaHCO_3_ (aq. sat. sol., 20 mL) and EtOAc (25 mL). The aqueous phase was extracted with EtOAc (2 × 25 mL), and the combined organic phase was washed with H_2_O (20 mL) and brine (20 mL), dried (Na_2_SO_4_), filtered, and concentrated *in vacuo* to afford the title compound **38b** as a yellow oil (306 mg, 93%), which was taken into next step without purification.

#### 3-(2-(4-Chloro-3-nitrophenoxy)ethyl)pyridine (**38a**)

K_2_CO_3_ (239 mg, 1.73 mmol) and 4-chloro-3-nitrophenol (150 mg, 0.864 mmol) were added sequentially to a solution of **38b** (209 mg, 1.03 mmol) in DMF (4 mL), and the resulting solution was stirred at 80 °C for 16 h in a sealed vessel. The mixture was to cooled to rt before addition of H_2_O (20 mL). The mixture was extracted with EtOAc (3 × 25 mL), and the combined organic phase was washed with H_2_O (3 × 25 mL) and brine (25 mL), dried (Na_2_SO_4_), filtered, and concentrated *in vacuo*. Purification of the resulting residue by flash column chromatography (0–60% EtOAc in pentane) afforded title compound **38a** as a white solid (86 mg, 36%): ^1^H NMR (400 MHz, CDCl_3_) δ 8.54 (dd, *J* = 2.4, 0.8 Hz, 1H), 8.49 (dd, *J* = 4.8, 1.6 Hz, 1H), 7.64–7.54 (m, 1H), 7.39 (d, *J* = 8.9 Hz, 1H), 7.35 (d, *J* = 3.0 Hz, 1H), 7.27–7.21 (m, 1H), 7.02 (dd, *J* = 8.9, 2.9 Hz, 1H), 4.20 (t, *J* = 6.4 Hz, 2H), 3.11 (t, *J* = 6.4 Hz, 2H); ^13^C NMR (101 MHz, CDCl_3_) δ 157.5, 150.4, 148.4, 148.2, 136.5, 133.2, 132.6, 123.6, 120.3, 118.6, 111.1, 69.0, 32.8; *m*/*z* LRMS (ESI^+^) 279 [M+H]^+^; HRMS (ESI^+^) C_13_H_11_^35^ClN_2_O_3_ [M+H]^+^ calcd 279.0531, found 279.0531.

#### 3-(2-(4-Chloro-3-nitrophenoxy)ethyl)pyridine 1-oxide (**38**)

Following general procedure B, **38** was obtained from **38a** (70 mg, 0.251 mmol). Purification by flash column chromatography (5% MeOH in CH_2_Cl_2_) afforded the title compound as a white solid (65 mg, 88%): ^1^H NMR (400 MHz, CDCl_3_) δ 8.20 (td, *J* = 1.6, 0.7 Hz, 1H), 8.12 (dt, *J* = 6.1, 1.6 Hz, 1H), 7.42 (d, *J* = 8.9 Hz, 1H), 7.36 (d, *J* = 2.9 Hz, 1H), 7.25–7.18 (m, 2H), 7.03 (dd, *J* = 8.9, 3.0 Hz, 1H), 4.22 (t, *J* = 6.1 Hz, 2H), 3.08 (t, *J* = 6.0 Hz, 2H); ^13^C NMR (101 MHz, CDCl_3_) δ 157.2, 148.3, 139.7, 137.7, 137.3, 132.7, 126.7, 125.9, 120.4, 119.1, 111.0, 67.9, 32.5; *m*/*z* LRMS (ESI^+^) 295 [M+H]^+^; HRMS (ESI^+^) C_13_H_11_^35^ClN_2_O_4_ [M+H]^+^ calcd 295.0480, found 295.0479; HPLC 98% (AUC), *t*_R_ = 3.9 min.

#### 3-(2-(4-Chloro-3-ethylphenoxy)ethyl)pyridine (**39a**)

Following general procedure A, **39a** was obtained from 4-chloro-3-ethylphenol (279 mg, 1.78 mmol) and 2-(pyridin-3-yl)ethan-1-ol (200 mg, 1.62 mmol). Purification by flash column chromatography (50% EtOAc in pentane) afforded the title compound as a white solid (180 mg, 43%): ^1^H NMR (400 MHz, CDCl_3_) δ 8.55 (dd, *J* = 2.4, 0.8 Hz, 1H), 8.48 (dd, *J* = 4.9, 1.7 Hz, 1H), 7.60 (ddd, *J* = 7.8, 2.3, 1.7 Hz, 1H), 7.23 (ddd, *J* = 7.8, 4.8, 0.9 Hz, 1H), 7.19 (d, *J* = 8.7 Hz, 1H), 6.75 (d, *J* = 3.0 Hz, 1H), 6.64 (dd, *J* = 8.7, 3.0 Hz, 1H), 4.13 (t, *J* = 6.5 Hz, 2H), 3.06 (t, *J* = 6.6 Hz, 2H), 2.68 (q, *J* = 7.5 Hz, 2H), 1.20 (t, *J* = 7.5 Hz, 3H); ^13^C NMR (101 MHz, CDCl_3_) δ 157.4, 150.4, 148.1, 142.8, 136.5, 133.9, 130.0, 125.6, 123.4, 115.9, 113.0, 68.2, 33.1, 27.0, 14.0; *m*/*z* LRMS (ESI^+^) 262 [M+H]^+^; HRMS (ESI^+^) C_15_H_16_^35^ClNO [M+H]^+^ calcd 262.0993, found 262.0992.

#### 3-(2-(4-Chloro-3-ethylphenoxy)ethyl)pyridine 1-oxide (**39**)

Following general procedure B, **39** was obtained from **39a** (60 mg, 0.229 mmol). Purification by flash column chromatography (5% MeOH in CH_2_Cl_2_) afforded the title compound as a white solid (55 mg, 87%): ^1^H NMR (400 MHz, CDCl_3_) δ 8.25–8.18 (m, 1H), 8.11 (td, *J* = 3.8, 1.8 Hz, 1H), 7.24–7.18 (m, 3H), 6.74 (d, *J* = 3.0 Hz, 1H), 6.63 (dd, *J* = 8.7, 3.0 Hz, 1H), 4.14 (t, *J* = 6.1 Hz, 2H), 3.03 (t, *J* = 6.1 Hz, 2H), 2.69 (q, *J* = 7.5 Hz, 2H), 1.20 (t, *J* = 7.5 Hz, 3H); ^13^C NMR (101 MHz, CDCl_3_) δ 157.1, 142.9, 139.6, 138.0, 137.5, 130.0, 127.0, 125.9, 125.7, 115.9, 112.8, 67.1, 32.7, 27.0, 14.0; *m*/*z* LRMS (ESI^+^) 278 [M+H]^+^; HRMS (ESI^+^) C_15_H_16_^35^ClNO_2_ [M+H]^+^ calcd 278.0942, found 278.0939; HPLC 97% (AUC), *t*_R_ = 5.9 min.

#### 3-(2-(4-Chloro-3-methoxyphenoxy)ethyl)pyridine (**40a**)

Following general procedure A, **40a** was obtained from 4-chloro-3-methoxyphenol (150 mg, 0.945 mmol) and 2-(pyridin-3-yl)ethan-1-ol (140 mg, 1.13 mmol). Purification by flash column chromatography (60% EtOAc in pentane) afforded the title compound as a white solid (140 mg, 56%): ^1^H NMR (400 MHz, CDCl_3_) δ 8.58–8.51 (m, 1H), 8.47 (dd, *J* = 4.8, 1.7 Hz, 1H), 7.63–7.55 (m, 1H), 7.22 (ddd, *J* = 7.8, 4.9, 0.9 Hz, 1H), 7.19 (d, *J* = 8.7 Hz, 1H), 6.45 (d, *J* = 2.7 Hz, 1H), 6.37 (dd, *J* = 8.7, 2.7 Hz, 1H), 4.13 (t, *J* = 6.5 Hz, 2H), 3.82 (s, 3H), 3.06 (t, *J* = 6.5 Hz, 2H); ^13^C NMR (101 MHz, CDCl_3_) δ 158.5, 155.7, 150.4, 148.1, 136.5, 133.8, 130.2, 123.5, 114.5, 105.8, 100.6, 68.3, 56.1, 33.0; *m*/*z* LRMS (ESI^+^) 264 [M+H]^+^; HRMS (ESI^+^) C_14_H_14_^35^ClNO_2_ [M+H]^+^ calcd 264.0786, found 264.0785.

#### 3-(2-(4-Chloro-3-methoxyphenoxy)ethyl)pyridine 1-oxide (**40**)

Following general procedure B, **40** was obtained from **40a** (120 mg, 0.455 mmol). Purification by flash column chromatography (5% MeOH in CH_2_Cl_2_) afforded the title compound as a white solid (110 mg, 86%): ^1^H NMR (400 MHz, CDCl_3_) δ 8.19 (dq, *J* = 1.8, 0.9 Hz, 1H), 8.08 (dt, *J* = 5.3, 2.1 Hz, 1H), 7.23–7.14 (m, 3H), 6.43 (d, *J* = 2.7 Hz, 1H), 6.35 (dd, *J* = 8.7, 2.7 Hz, 1H), 4.12 (t, *J* = 6.1 Hz, 2H), 3.82 (s, 3H), 3.00 (t, *J* = 6.1 Hz, 2H); ^13^C NMR (101 MHz, CDCl_3_) δ 158.2, 155.7, 139.6, 137.9, 137.5, 130.2, 126.8, 125.7, 114.8, 105.6, 100.6, 67.2, 56.2, 32.7; *m*/*z* LRMS (ESI^+^) 280 [M+H]^+^; HRMS (ESI^+^) C_14_H_14_^35^ClNO_3_ [M+H]^+^ calcd 280.0735, found 280.0735; HPLC 99% (AUC), *t*_R_ = 3.8 min.

#### 3-(2-(4-Chloro-3-cyclopropylphenoxy)ethyl)pyridine (**41a**)

A mixture of **32a** (100 mg, 0.330 mmol), cyclopropyl boronic acid (36 mg, 0.400 mmol), tricyclohexylphosphine (8 mg, 0.028 mmol), and K_3_PO_4_ (206 mg, 0.973 mmol) in toluene (3 mL) and water (1 mL) was degassed with N_2_ for 5 min before addition of Pd(OAc)_2_ (3 mg, 0.014 mmol). The mixture was heated in a sealed tube at 110 °C for 16 h. After completion, the mixture was cooled to rt, diluted with EtOAc, filtered through Celite (EtOAc), and concentrated *in vacuo*. The residue was purified by flash column chromatography (50% EtOAc in pentane) to afford title compound **41a** (40 mg, 44%) as a white solid: ^1^H NMR (400 MHz, CDCl_3_) δ 8.57–8.51 (m, 1H), 8.49 (dd, *J* = 4.8, 1.7 Hz, 1H), 7.63–7.54 (m, 1H), 7.26–7.23 (m, 1H), 7.21 (d, *J* = 8.7 Hz, 1H), 6.61 (dd, *J* = 8.7, 3.0 Hz, 1H), 6.43 (d, *J* = 2.9 Hz, 1H), 4.11 (t, *J* = 6.6 Hz, 2H), 3.06 (t, *J* = 6.6 Hz, 2H), 2.15 (tt, *J* = 8.5, 5.3 Hz, 1H), 1.03–0.93 (m, 2H), 0.69–0.60 (m, 2H); ^13^C NMR (101 MHz, CDCl_3_) δ 157.5, 150.5, 148.2, 142.3, 136.5, 133.9, 129.8, 127.1, 123.5, 112.8, 112.4, 68.3, 33.1, 13.6, 8.3, 8.3; *m*/*z* LRMS (ESI^+^) 274 [M+H]^+^; HRMS (ESI^+^) C_16_H_16_^35^ClNO [M+H]^+^ calcd 274.0993, found 274.0991.

#### 3-(2-(4-Chloro-3-cyclopropylphenoxy)ethyl)pyridine 1-oxide (**41**)

Following general procedure B, **41** was obtained from **41a** (30 mg, 0.110 mmol). Purification by flash column chromatography (6% MeOH in CH_2_Cl_2_) afforded the title compound as a white solid (27 mg, 85%): ^1^H NMR (400 MHz, CDCl_3_) δ 8.20 (d, *J* = 1.9 Hz, 1H), 8.11 (dt, *J* = 5.3, 2.0 Hz, 1H), 7.25–7.17 (m, 3H), 6.60 (dd, *J* = 8.7, 3.0 Hz, 1H), 6.43 (d, *J* = 3.0 Hz, 1H), 4.12 (t, *J* = 6.1 Hz, 2H), 3.02 (t, *J* = 6.1 Hz, 2H), 2.15 (tt, *J* = 8.5, 5.4 Hz, 1H), 1.03–0.95 (m, 2H), 0.70–0.61 (m, 2H); ^13^C NMR (101 MHz, CDCl_3_) δ 157.3, 142.5, 139.8, 138.1, 137.7, 129.9, 127.5, 127.0, 125.8, 112.9, 112.4, 67.3, 32.9, 13.7, 8.3, 8.3; *m*/*z* LRMS (ESI^+^) 290 [M+H]^+^; HRMS (ESI^+^) C_16_H_16_^35^ClNO_2_ [M+H]^+^ calcd 290.0942, found 290.0942; HPLC 95% (AUC), *t*_R_ = 5.9 min.

#### 3-(2-(4-Chloro-3-(trifluoromethyl)phenoxy)ethyl)pyridine (**42a**)

Following general procedure A, **42a** was obtained from 4-chloro-3-(trifluoromethyl)phenol (350 mg, 1.78 mmol) and 2-(pyridin-3-yl)ethan-1-ol (200.0 mg, 1.62 mmol). Purification by flash column chromatography (50% EtOAc in pentane) afforded the title compound as a white solid (205 mg, 42%): ^1^H NMR (400 MHz, CDCl_3_) δ 8.56 (d, *J* = 2.3 Hz, 1H), 8.51 (dd, *J* = 4.9, 1.7 Hz, 1H), 7.61 (dt, *J* = 7.9, 1.9 Hz, 1H), 7.37 (dd, *J* = 8.9, 0.8 Hz, 1H), 7.29–7.21 (m, 1H), 7.17 (d, *J* = 3.0 Hz, 1H), 6.95 (dd, *J* = 8.8, 3.0 Hz, 1H), 4.18 (t, *J* = 6.5 Hz, 2H), 3.10 (t, *J* = 6.5 Hz, 2H); ^13^C NMR (101 MHz, CDCl_3_) δ 157.1, 150.5, 148.4, 136.6, 136.6, 133.5, 132.5, 132.5, 129.4, 123.6, 118.8, 114.1, 68.7, 32.9; *m*/*z* LRMS (ESI^+^) 302 [M+H]^+^; HRMS (ESI^+^) C_14_H_11_^35^ClF_3_NO [M+H]^+^ calcd 302.0554, found 302.0555.

#### 3-(2-(4-Chloro-3-(trifluoromethyl)phenoxy)ethyl)pyridine 1-oxide (**42**)

Following general procedure B, **42** was obtained from **42a** (30 mg, 0.099 mmol). Purification by flash column chromatography (5% MeOH in CH_2_Cl_2_) afforded the title compound as a white solid (28 mg, 90%): ^1^H NMR (400 MHz, CDCl_3_) δ 8.21 (d, *J* = 1.7 Hz, 1H), 8.13 (dt, *J* = 5.9, 1.7 Hz, 1H), 7.40 (s, 1H), 7.25–7.19 (m, 2H), 7.17 (d, *J* = 3.0 Hz, 1H), 6.96 (dd, *J* = 8.8, 3.0 Hz, 1H), 4.19 (t, *J* = 6.1 Hz, 2H), 3.07 (t, *J* = 6.1 Hz, 2H); ^13^C NMR (101 MHz, CDCl_3_) δ 156.8, 139.7, 137.8, 137.6, 132.6, 132.6, 126.8, 125.9, 125.9, 121.3, 118.8, 114.0, 67.7, 32.7; ^19^F NMR (376 MHz, CDCl_3_) δ −62.90; *m*/*z* LRMS (ESI^+^) 318 [M+H]^+^; HRMS (ESI^+^) C_14_H_11_^35^ClF_3_NO_2_ [M+H]^+^ calcd 318.0503, found 318.0500; HPLC 97% (AUC), *t*_R_ = 5.7 min.

#### 3-(2-(3,5-Bis(trifluoromethyl)phenoxy)ethyl)pyridine (**43a**)

Following general procedure A, **43a** was obtained from 2-(pyridin-3-yl)ethan-1-ol (200 mg, 1.62 mmol) and 3,5-bis(trifluoromethyl)phenol (410 mg, 1.78 mmol). Purification by flash column chromatography (50% EtOAc in pentane) afforded the title compound as a colorless oil (320 mg, 59%): ^1^H NMR (400 MHz, CDCl_3_) δ 8.58 (dd, *J* = 2.3, 0.8 Hz, 1H), 8.52 (dd, *J* = 4.8, 1.7 Hz, 1H), 7.63 (ddd, *J* = 7.8, 2.3, 1.7 Hz, 1H), 7.45 (tt, *J* = 1.6, 0.8 Hz, 1H), 7.29–7.24 (m, 3H), 4.26 (t, *J* = 6.4 Hz, 2H), 3.14 (t, *J* = 6.4 Hz, 2H); ^13^C NMR (101 MHz, CDCl_3_) δ 159.3, 150.5, 148.5, 136.6, 133.3, 133.0 (q, *J* = 33 Hz), 127.3, 124.6, 123.6, 121.9, 119.2, 114.9 (d, *J* = 4 Hz), 114.7 (t, *J* = 3 Hz), 68.9, 32.9; ^19^F NMR (376 MHz, CDCl_3_) δ −63.05; *m*/*z* LRMS (ESI^+^) 336 [M+H]^+^; HRMS (ESI^+^) C_15_H_11_F_6_NO [M+H]^+^ calcd 336.0818, found 336.0809.

#### 3-(2-(3,5-Bis(trifluoromethyl)phenoxy)ethyl)pyridine 1-oxide (**43**)

Following general procedure B, **43** was obtained from **43a** (80 mg, 0.214 mmol). Purification by flash column chromatography (5% MeOH in CH_2_Cl_2_) afforded the title compound as a white solid (72 mg, 96%): ^1^H NMR (400 MHz, CDC_l3_) δ 8.22 (d, *J* = 1.8 Hz, 1H), 8.12 (dt, *J* = 5.8, 1.7 Hz, 1H), 7.46 (tt, *J* = 1.6, 0.8 Hz, 1H), 7.30–7.18 (m, 4H), 4.26 (t, *J* = 6.0 Hz, 2H), 3.10 (t, *J* = 6.0 Hz, 2H); ^13^C NMR (101 MHz, CDCl_3_) δ 158.9, 139.7, 137.9, 137.3, 133.1 (q, *J* = 66 Hz), 127.2, 126.7, 125.9, 124.5, 121.8, 119.1, 115.0 (m), 114.9 (m), 67.8, 32.6; ^19^F NMR (376 MHz, CDCl_3_) δ −63.06; *m*/*z* LRMS (ESI^+^) 352 [M+H]^+^; HRMS (ESI^+^) C_15_H_11_F_6_NO_2_ [M+H]^+^ calcd 352.0767, found 352.0758; HPLC 99% (AUC), *t*_R_ = 6.4 min.

#### 3-(((4-Chloronaphthalen-1-yl)oxy)methyl)pyridine (**44a**)

Following general procedure A, **44a** was obtained from pyridin-3-ylmethanol (150 mg, 1.37 mmol) and 4-chloronaphthalen-1-ol (294 mg, 1.65 mmol). Purification by flash column chromatography (50% EtOAc in pentane) afforded the title compound as a colorless oil (229 mg, 62%): ^1^H NMR (400 MHz, CDCl_3_) δ 8.91–8.51 (m, 2H), 8.31 (ddd, *J* = 8.4, 1.4, 0.7 Hz, 1H), 8.22 (dt, *J* = 8.4, 1.0 Hz, 1H), 7.89–7.83 (m, 1H), 7.63 (ddd, *J* = 8.4, 6.8, 1.3 Hz, 1H), 7.55 (ddd, *J* = 8.2, 6.8, 1.2 Hz, 1H), 7.46 (d, *J* = 8.2 Hz, 1H), 7.37 (dd, *J* = 7.9, 4.8 Hz, 1H), 6.80 (d, *J* = 8.2 Hz, 1H), 5.24 (s, 2H); ^13^C NMR (101 MHz, CDCl_3_) δ 153.3, 149.7, 149.0, 135.4, 131.5, 127.8, 126.8, 126.4, 126.3 125.7, 124.5, 124.1, 123.8, 122.5, 105.3, 68.1; *m*/*z* LRMS (ESI^+^) 270 [M+H]^+^; HRMS (ESI^+^) C_16_H_12_^35^ClNO [M+H]^+^ calcd 270.0680, found 270.0676;

#### 3-(((4-Chloronaphthalen-1-yl)oxy)methyl)pyridine 1-oxide (**44**)

Following general procedure B, **44** was obtained from **44a** (50 mg, 0.185 mmol). Purification by flash column chromatography (5% MeOH in CH_2_Cl_2_) afforded the title compound as a white solid (38 mg, 72%): ^1^H NMR (400 MHz, CDCl_3_) δ 8.45 (dq, *J* = 1.7, 0.9 Hz, 1H), 8.30 (ddd, *J* = 8.3, 1.4, 0.7 Hz, 1H), 8.26–8.18 (m, 2H), 7.65 (ddd, *J* = 8.4, 6.9, 1.4 Hz, 1H), 7.57 (ddd, *J* = 8.2, 6.9, 1.3 Hz, 1H), 7.44 (d, *J* = 8.2 Hz, 1H), 7.40 (dt, *J* = 8.0, 1.3 Hz, 1H), 7.33 (dd, *J* = 7.9, 6.3 Hz, 1H), 6.74 (d, *J* = 8.2 Hz, 1H), 5.20 (s, 2H); ^13^C NMR (101 MHz, CDCl_3_) δ 152.6, 138.8, 138.1, 136.6, 131.5, 128.0, 126.6, 126.5, 126.1, 125.5, 124.7, 124.5, 124.5, 122.3, 105.3, 66.7; *m*/*z* LRMS (ESI^+^) 286 [M+H]^+^; HRMS (ESI^+^) C_16_H_12_^35^ClNO_2_ [M+H]^+^ calcd 286.0629, found 286.0624; HPLC 97% (AUC), *t*_R_ = 6.5 min.

#### 3-(3-((4-Chloronaphthalen-1-yl)oxy)propyl)pyridine (**45a**)

Following general procedure A, **45a** was obtained from 3-(pyridin-3-yl)propan-1-ol (200 mg, 1.46 mmol) and 4-chloronaphthalen-1-ol (313 mg, 1.75 mmol). Purification by flash column chromatography (50% EtOAc in pentane) afforded the title compound as a colorless oil (261 mg, 60%): ^1^H NMR (400 MHz, CDCl_3_) δ 8.51 (d, *J* = 22.0 Hz, 2H), 8.28 (ddd, *J* = 8.3, 1.4, 0.7 Hz, 1H), 8.24–8.17 (m, 1H), 7.62 (ddd, *J* = 8.4, 6.9, 1.4 Hz, 1H), 7.58–7.50 (m, 2H), 7.43 (d, *J* = 8.2 Hz, 1H), 7.22 (dd, *J* = 7.8, 4.7 Hz, 1H), 6.67 (d, *J* = 8.2 Hz, 1H), 4.12 (t, *J* = 6.0 Hz, 2H), 2.93 (t, 2H), 2.32–2.19 (m, 2H); ^13^C NMR (101 MHz, CDCl_3_) δ 153.7, 150.1, 147.7, 136.8, 136.1, 131.5, 127.6, 126.7, 126.1, 125.9, 124.4, 123.6, 123.4, 122.4, 104.7, 67.0, 30.6, 29.7; *m*/*z* LRMS (ESI^+^) 298 [M+H]^+^; HRMS (ESI^+^) C_18_H_16_^35^ClNO [M+H]^+^ calcd 298.0993, found 298.0988.

#### 3-(3-((4-Chloronaphthalen-1-yl)oxy)propyl)pyridine 1-oxide (**45**)

Following general procedure B, 3-(3-((4-chloronaphthalen-1-yl)oxy)propyl)pyridine 1-oxide 45 was obtained from 3-(3-((4-chloronaphthalen-1-yl)oxy)propyl)pyridine 45a (65 mg, 0.217 mmol). Purification by flash column chromatography (5% MeOH in CH_2_Cl_2_) afforded the title compound as a white solid (56 mg, 82%): ^1^H NMR (400 MHz, CDCl_3_) δ 8.27–8.15 (m, 3H), 8.11 (dt, *J* = 6.1, 1.6 Hz, 1H), 7.63 (ddd, *J* = 8.4, 6.9, 1.4 Hz, 1H), 7.55 (ddd, *J* = 8.2, 6.8, 1.3 Hz, 1H), 7.43 (d, *J* = 8.2 Hz, 1H), 7.23–7.13 (m, 2H), 6.68 (d, *J* = 8.2 Hz, 1H), 4.14 (t, *J* = 5.9 Hz, 2H), 2.92 (t, 2H), 2.31–2.20 (m, 2H); ^13^C NMR (101 MHz, CDCl_3_) δ 153.5, 140.7, 139.3, 137.3, 131.5, 127.7, 126.9, 126.7, 126.2, 125.84, 125.8, 124.5, 123.6, 122.2, 104.8, 66.6, 29.9, 29.6; *m*/*z* LRMS (ESI^+^) 314 [M+H]^+^; HRMS (ESI^+^) C_18_H_16_^35^ClNO_2_ [M+H]^+^ calcd 314.0942, found 314.0936; HPLC 95% (AUC), *t*_R_ = 7.3 min.

#### (*E*)-3-(Naphthalen-1-yl)-1-(pyridin-3-yl)prop-2-en-1-one (**46c**)

Following general procedure C, **46c** was obtained from 1-naphthaldehyde (407 μL, 3.00 mmol) and 1-(pyridin-3-yl)ethan-1-one (330 μL, 3.00 mmol) as a yellow oil (590 mg, 76%): ^1^H NMR (400 MHz, CDCl_3_) δ 9.30 (d, *J* = 2.2 Hz, 1H), 8.82 (dd, *J* = 4.9, 1.7 Hz, 1H), 8.71 (d, *J* = 15.4 Hz, 1H), 8.34 (dt, *J* = 7.9, 2.0 Hz, 1H), 8.23 (dd, *J* = 8.4, 1.2 Hz, 1H), 7.98–7.87 (m, 3H), 7.65–7.43 (m, 5H); ^13^C NMR (101 MHz, CDCl_3_) δ 188.9, 153.2, 149.9, 143.0, 136.1, 133.9, 133.6, 132.0, 131.9, 131.4, 129.0, 127.3, 126.5, 125.6, 125.4, 123.9, 123.9, 123.4; *m*/*z* LRMS (ESI^+^) 260 [M+H]^+^; HRMS (ESI^+^) C_18_H_13_NO [M+H]^+^ calcd 260.1070, found 260.1070.

#### 3-(Naphthalen-1-yl)-1-(pyridin-3-yl)propan-1-one (**46b**)

Following general procedure D, **46b** was obtained from **46c** (90 mg, 0.347 mmol). Purification by flash column chromatography (30% EtOAc in CH_2_Cl_2_) afforded the title compound as a yellow oil (71 mg, 78%): ^1^H NMR (400 MHz, CDCl_3_) δ 9.15 (d, 1H), 8.76 (dd, *J* = 4.9, 1.7 Hz, 1H), 8.22 (dt, *J* = 8.0, 2.0 Hz, 1H), 8.04 (dt, *J* = 8.6, 0.9 Hz, 1H), 7.92–7.85 (m, 1H), 7.78–7.72 (m, 1H), 7.57–7.47 (m, 2H), 7.45–7.38 (m, 3H), 3.56 (dd, *J* = 8.3, 6.2 Hz, 2H), 3.44 (ddd, *J* = 8.0, 6.6, 0.9 Hz, 2H); ^13^C NMR (101 MHz, CDCl_3_) δ 198.2, 153.4, 149.5, 136.9, 135.7, 134.1, 132.3, 131.7, 129.2, 127.4, 126.4, 126.4, 125.8, 125.8, 123.8, 123.5, 40.1, 27.1; *m*/*z* LRMS (ESI^+^) 262 [M+H]^+^; HRMS (ESI^+^) C_18_H_15_NO [M+H]^+^ calcd 262.1226, found 262.1226.

#### 3-(3-(Naphthalen-1-yl)propyl)pyridine (**46a**)

Following general procedure E, **46a** was obtained from **46b** (50 mg, 0.191 mmol). Purification by flash column chromatography (10% EtOAc in CH_2_Cl_2_) afforded the title compound as a brown oil (32 mg, 68%): ^1^H NMR (600 MHz, CDCl_3_) δ 8.50 (d, *J* = 2.2 Hz, 1H), 8.47 (d, *J* = 4.9 Hz, 1H), 7.95 (d, *J* = 8.1 Hz, 1H), 7.86 (dd, *J* = 7.7, 1.7 Hz, 1H), 7.73 (d, *J* = 8.2 Hz, 1H), 7.61 (dt, *J* = 7.8, 2.1 Hz, 1H), 7.52–7.46 (m, 2H), 7.40 (t, *J* = 7.6 Hz, 1H), 7.30 (dd, *J* = 11.0, 5.2 Hz, 2H), 3.14 (t, *J* = 7.7 Hz, 2H), 2.77 (t, *J* = 7.8 Hz, 2H), 2.13 (tt, *J* = 9.2, 6.7 Hz, 2H); ^13^C NMR (151 MHz, CDCl_3_) δ 148.7, 146.1, 138.3, 137.7, 137.4, 134.1, 131.9, 129.0, 127.0, 126.2, 126.0, 125.7, 125.7, 123.9, 123.7, 33.0, 32.6, 31.9; *m*/*z* LRMS (ESI^+^) 248 [M+H]^+^; HRMS (ESI^+^) C_18_H_17_N [M+H]^+^ calcd 248.1434, found 248.1435.

#### 3-(3-(Naphthalen-1-yl)propyl)pyridine 1-oxide (**46**)

Following general procedure B, **46** was obtained from **46a** (20 mg, 0.080 mmol). Purification by flash column chromatography (6% MeOH in CH_2_Cl_2_) afforded the title compound as a brown oil (18 mg, 86%): ^1^H NMR (400 MHz, CDCl_3_) δ 8.22 (d, *J* = 1.8 Hz, 1H), 8.18 (dt, *J* = 6.3, 1.4 Hz, 1H), 8.09–8.03 (m, 1H), 7.97 (dd, *J* = 8.0, 1.6 Hz, 1H), 7.84 (dt, *J* = 8.2, 1.1 Hz, 1H), 7.66–7.56 (m, 2H), 7.51 (dd, *J* = 8.2, 7.0 Hz, 1H), 7.40 (dd, *J* = 7.1, 1.3 Hz, 1H), 7.24 (dd, *J* = 7.9, 6.3 Hz, 1H), 7.16 (dt, *J* = 7.9, 1.3 Hz, 1H), 3.28–3.15 (m, 2H), 2.82–2.70 (m, 2H), 2.26–2.11 (m, 2H); ^13^C NMR (101 MHz, CDCl_3_) δ 141.1, 139.0, 137.1, 136.8, 133.9, 131.6, 128.8, 126.9, 126.2, 126.0, 125.9, 125.5, 125.4, 125.4, 123.4, 32.4, 32.1, 30.9; *m*/*z* LRMS (ESI^+^) 264 [M+H]^+^; HRMS (ESI^+^) C_18_H_17_NO [M+H]^+^ calcd 264.1383, found 264.1384; HPLC 96% (AUC), *t*_R_ = 5.8 min.

#### 3-(3-(Naphthalen-1-yl)propanoyl)pyridine 1-oxide (**47**)

Following general procedure B, **47** was obtained from **46b** (40 mg, 0.153 mmol). Purification by flash column chromatography (6% MeOH in CH_2_Cl_2_) afforded the title compound as a white solid (33 mg, 78%): ^1^H NMR (400 MHz, CDCl_3_) δ 8.68 (t, *J* = 1.6 Hz, 1H), 8.29 (ddd, *J* = 6.4, 1.8, 1.0 Hz, 1H), 8.02–7.96 (m, 1H), 7.90–7.84 (m, 1H), 7.74 (dt, *J* = 7.8, 1.2 Hz, 1H), 7.68 (dt, *J* = 8.0, 1.3 Hz, 1H), 7.57–7.47 (m, 2H), 7.43–7.35 (m, 2H), 7.31 (dd, *J* = 8.0, 6.4 Hz, 1H), 3.53 (t, *J* = 7.8, 7.1 Hz, 2H), 3.35 (t, *J* = 7.4 Hz, 2H); ^13^C NMR (101 MHz, CDCl_3_) δ 195.5, 142.4, 139.2, 136.3, 135.6, 134.1, 131.6, 129.2, 127.5, 126.4, 126.4, 126.1, 125.9, 125.7, 124.5, 123.3, 40.1, 26.7; *m*/*z* LRMS (ESI^+^) 278 [M+H]^+^; HRMS (ESI^+^) C_18_H_15_NO_2_ [M+H]^+^ calcd 278.1176, found 278.1173; HPLC 97% (AUC), *t*_R_ = 4.5 min.

#### 3-(4-Chlorophenethoxy)pyridine (**48a**)

Following general procedure A, **48a** was obtained from 2-(4-chlorophenyl)ethan-1-ol (200 mg, 1.28 mmol) and pyridin-3-ol (145.7 mg, 1.53 mmol). Purification by flash column chromatography (50% EtOAc in pentane) afforded the title compound as a white solid (158 mg, 53%): ^1^H NMR (400 MHz, CDCl_3_) δ 8.29 (dd, *J* = 2.8, 0.8 Hz, 1H), 8.20 (dd, *J* = 4.5, 1.5 Hz, 1H), 7.33–7.24 (m, 2H), 7.25–7.09 (m, 4H), 4.18 (td, *J* = 6.7, 1.5 Hz, 2H), 3.07 (td, *J* = 6.7, 1.5 Hz, 2H); ^13^C NMR (101 MHz, CDCl_3_) δ 154.9, 142.4, 138.1, 136.5, 132.6, 130.4, 130.4, 128.8, 128.8, 123.9, 121.2, 68.7, 35.1; *m*/*z* LRMS (ESI^+^) 234 [M+H]^+^; HRMS (ESI^+^) C_13_H_12_ClNO [M+H]^+^ calcd 234.0677, found 234.0680.

#### 3-(4-Chlorophenethoxy)pyridine 1-oxide (**48**)

Following general procedure B, **48** was obtained from **48a** (75 mg, 0.321 mmol). Purification by flash column chromatography (5% MeOH in CH_2_Cl_2_) afforded the title compound as a white solid (68 mg, 85%): ^1^H NMR (400 MHz, CDCl_3_) δ 7.93 (t, *J* = 1.9 Hz, 1H), 7.87 (ddd, *J* = 6.4, 1.7, 0.9 Hz, 1H), 7.35–7.23 (m, 2H), 7.23–7.15 (m, 2H), 7.13 (dd, *J* = 8.7, 6.3 Hz, 1H), 6.82 (ddd, *J* = 8.7, 2.2, 0.9 Hz, 1H), 4.15 (t, *J* = 6.6 Hz, 2H), 3.06 (t, *J* = 6.6 Hz, 2H); ^13^C NMR (101 MHz, CDCl_3_) δ 157.2, 135.8, 132.9, 132.7, 130.4, 130.4, 128.9, 128.9, 128.3, 125.5, 113.3, 69.5, 34.8; *m*/*z* LRMS (ESI^+^) 250 [M+H]^+^; HRMS (ESI^+^) C_13_H_12_^35^ClNO_2_ [M+H]^+^ calcd 250.0625, found 250.0629; HPLC 98% (AUC), *t*_R_ = 4.3 min.

#### (*E*)-3-(4-Chlorophenyl)-1-(pyridin-3-yl)prop-2-en-1-one (**49c**)

Following general procedure C, **49c** was obtained from 4-chlorobenzaldehyde (2.80 g, 20 mmol) and 1-(pyridin-3-yl)ethan-1-one (1.21 g, 10.0 mmol) as a yellow solid (600 mg, 25%): ^1^H NMR (400 MHz, CDCl_3_) δ 9.24–9.11 (m, 1H), 8.79 (dd, *J* = 4.8, 1.7 Hz, 1H), 8.26 (dt, *J* = 8.0, 2.0 Hz, 1H), 7.77 (d, *J* = 15.7 Hz, 1H), 7.59–7.53 (m, 2H), 7.47–7.42 (m, 2H), 7.41–7.36 (m, 2H); ^13^C NMR (101 MHz, CDCl_3_) δ 188.9, 153.4, 149.8, 144.5, 137.0, 136.0, 133.4, 133.0, 129.9, 129.9, 129.5, 129.5, 123.8, 121.8; *m*/*z* LRMS (ESI^+^) 233 [M(^37^Cl)+H]^+^ 231 [M(^35^Cl)+H]^+^; HRMS (ESI^+^) C_14_H_10_^35^ClNO [M+H]^+^ calcd 244.0524, found 244.0521.

#### 3-(4-Chlorophenyl)-1-(pyridin-3-yl)propan-1-one (**49b**)

Following general procedure D, **49b** was obtained from **49c** (580 mg, 2.39 mmol). Purification by flash column chromatography (40% EtOAc in pentane) afforded the title compound as a yellow oil (421 mg, 72%): ^1^H NMR (400 MHz, CDCl_3_) δ 9.10 (dd, *J* = 2.3, 0.9 Hz, 1H), 8.71 (dd, *J* = 4.8, 1.7 Hz, 1H), 8.15 (ddd, *J* = 8.0, 2.3, 1.7 Hz, 1H), 7.35 (ddd, *J* = 8.0, 4.8, 0.9 Hz, 1H), 7.22–7.16 (m, 2H), 7.18–7.08 (m, 2H), 3.24 (t, *J* = 7.3 Hz, 2H), 2.99 (t, *J* = 7.4 Hz, 2H); ^13^C NMR (101 MHz, CDCl_3_) δ 197.6, 153.5, 149.5, 139.2, 135.2, 131.9, 131.9, 129.8, 129.8, 128.6, 128.6, 123.6, 40.3, 28.9; *m*/*z* LRMS (ESI^+^) 247 [M(^37^Cl)+H]^+^ 245 [M(^35^Cl)+H]^+^; HRMS (ESI^+^) C_14_H_12_^35^ClNO [M+H]^+^ calcd 246.068, 246.0677.

#### 3-(3-(4-Chlorophenyl)propyl)pyridine (**49a**)

Following general procedure E, **49a** was obtained from **49b** (83 mg, 0.339 mmol). Purification by flash column chromatography (40% EtOAc in pentane) afforded the title compound as a brown oil (77 mg, 98%): ^1^H NMR (400 MHz, CDCl_3_) δ 8.47–8.40 (m, 2H), 7.47 (dt, *J* = 7.8, 2.0 Hz, 1H), 7.28–7.21 (m, 2H), 7.23–7.17 (m, 1H), 7.12–7.06 (m, 2H), 2.61 (td, *J* = 7.7, 2.1 Hz, 4H), 1.98–1.86 (m, 2H); ^13^C NMR (101 MHz, CDCl_3_) δ 150.0, 147.5, 140.2, 137.2, 135.9, 131.7, 129.8, 129.8, 128.6, 128.6, 123.4, 34.7, 32.6, 32.4; *m*/*z* LRMS (ESI^+^) 234 [M(^37^Cl)+H]^+^ 232 [M(^35^Cl)+H]^+^; HRMS (ESI^+^) C_14_H_14_^35^ClN [M+H]^+^ calcd 232.0888, found 232.0885.

#### 3-(3-(4-Chlorophenyl)propyl)pyridine 1-oxide (**49**)

Following general procedure B, **49** was obtained from **49a** (40 mg, 0.173 mmol). Purification by flash column chromatography (6% MeOH in CH_2_Cl_2_) afforded the title compound as a transparent oil (40 mg, 94%): ^1^H NMR (400 MHz, CDCl_3_) δ 8.05 (dt, *J* = 2.8, 1.3 Hz, 2H), 7.25–7.20 (m, 2H), 7.17 (dd, *J* = 7.9, 6.9 Hz, 1H), 7.08–7.04 (m, 3H), 2.58 (dt, *J* = 15.8, 7.7 Hz, 4H), 1.96–1.85 (m, 2H); ^13^C NMR (101 MHz, CDCl_3_) δ 141.1, 139.6, 139.1, 137.0, 131.9, 129.7, 129.7, 128.7, 128.7, 126.6, 125.7, 34.4, 32.0, 31.7; *m*/*z* LRMS (ESI^+^) 250 [M(^37^Cl)+H]^+^ 248 [M(^35^Cl)+H]^+^; HRMS (ESI^+^) C_14_H_14_^35^ClNO [M+H]^+^ calcd 248.0837, found 248.0834; HPLC 99% (AUC), *t*_R_ = 5.8 min.

#### 3-(3-(4-Chlorophenyl)propanoyl)pyridine 1-oxide (**50**)

Following general procedure B, **50** was obtained from **49b** (15 mg, 0.061 mmol). Purification by flash column chromatography (6% MeOH in CH_2_Cl_2_) afforded the title compound as a white solid (13 mg, 81%): ^1^H NMR (400 MHz, CDCl_3_) δ 8.97 (t, *J* = 1.6 Hz, 1H), 8.60 (ddd, *J* = 6.5, 1.8, 1.1 Hz, 1H), 8.00 (dt, *J* = 8.0, 1.3 Hz, 1H), 7.65 (dd, *J* = 8.0, 6.4 Hz, 1H), 7.57–7.51 (m, 2H), 7.46–7.40 (m, 2H), 3.49 (t, *J* = 7.1 Hz, 2H), 3.31 (t, *J* = 7.3 Hz, 2H); ^13^C NMR (101 MHz, CDCl_3_) δ 195.0, 142.5, 139.2, 138.7, 135.5, 132.4, 129.9, 129.9, 128.9, 128.9, 126.2, 124.5, 40.6, 28.8; *m*/*z* LRMS (ESI^+^) 264 [M(^37^Cl)+H]^+^ 262 [M(^35^Cl)+H]^+^; HRMS (ESI^+^) C_14_H_12_^35^ClNO_2_ [M+H]^+^ calcd 262.0629, found 262.0625; HPLC 99% (AUC), *t*_R_ = 4.3 min.

#### 3-(2-((4-Chlorophenyl)thio)ethyl)pyridine (**51a**)

K_2_CO_3_ (382 mg, 2.76 mmol) and 4-chlorobenzenethiol (200 mg, 1.38 mmol) were added sequentially to a solution of **38b** (306 mg, 1.52 mmol) in THF (4 mL), and the resulting solution was stirred at 50 °C for 16 h in a sealed vessel. The mixture was allowed to cool to rt before addition of H_2_O (20 mL). The mixture was extracted with EtOAc (3 × 25 mL), and the combined organic phase was washed with H_2_O (3 × 25 mL) and brine (25 mL), dried (Na_2_SO_4_), filtered, and concentrated *in vacuo*. Purification of the resulting residue by flash column chromatography (0 to 60% EtOAc in pentane) afforded title compound **51a** as a yellow oil (130 mg, 34%): ^1^H NMR (400 MHz, CDCl_3_) δ 8.52–8.43 (m, 2H), 7.58–7.51 (m, 1H), 7.26 (d, *J* = 3.2 Hz, 5H), 3.15 (t, *J* = 7.5 Hz, 2H), 2.92 (t, *J* = 7.5 Hz, 2H); ^13^C NMR (101 MHz, CDCl_3_) δ 150.0, 148.0, 136.2, 135.3, 134.3, 132.5, 131.1, 131.1, 129.3, 129.3, 123.5, 35.3, 32.7; *m*/*z* LRMS (ESI^+^) 250 [M+H]^+^; HRMS (ESI^+^) C_13_H_12_^35^ClNS [M+H]^+^ calcd 250.0452, found 250.0454.

#### 3-(2-((4-Chlorophenyl)sulfonyl)ethyl)pyridine 1-oxide (**51**)

Following general procedure B, **51** was obtained from **51a** (50 mg, 0.200 mmol). Purification by flash column chromatography (5% MeOH in CH_2_Cl_2_) afforded the title compound as a white solid (48 mg, 80%): ^1^H NMR (400 MHz, acetone-*d*^6^) δ 8.10 (td, *J* = 1.6, 0.8 Hz, 1H), 8.00–7.94 (m, 3H), 7.74–7.67 (m, 2H), 7.30–7.18 (m, 2H), 3.72–3.63 (m, 2H), 3.07–2.97 (m, 2H); ^13^C NMR (101 MHz, acetone-*d*^6^) δ 140.6, 139.8, 139.2, 138.4, 138.0, 130.9, 130.9, 130.4, 130.4, 126.8, 125.4, 55.9, 26.5; *m*/*z* LRMS (ESI^+^) 298 [M+H]^+^; HRMS (ESI^+^) C_13_H_12_^35^ClNO_3_S [M+H]^+^ calcd 298.0299, found 298.0297; HPLC 99% (AUC), *t*_R_ = 2.8 min.

#### (*E*)-1-(4-Chlorophenyl)-3-(pyridin-3-yl)prop-2-en-1-one (**52d**)

A mixture of 4-chloroacetophenone (1.00 g, 6.5 mmol), 3-pyridinecarboxaldehyde (1.40 g, 13.0 mmol), and DBU (1.0 mL, 6.5 mmol) in THF (15 mL) was stirred at rt for 48 h. After completion, the mixture was concentrated *in vacuo* and the resulting residue purified by flash column chromatography (50% EtOAc in pentane) to afford title compound **52d** as a yellow oil (1.0 g, 63%): ^1^H NMR (400 MHz, CDCl_3_) δ 8.94–8.80 (m, 1H), 8.64 (d, *J* = 4.5 Hz, 1H), 8.02–7.91 (m, 3H), 7.79 (d, *J* = 15.8 Hz, 1H), 7.55 (d, *J* = 15.8 Hz, 1H), 7.52–7.44 (m, 2H), 7.37 (dd, *J* = 7.9, 4.8 Hz, 1H); ^13^C NMR (101 MHz, CDCl_3_) δ 188.6, 151.3, 150.0, 141.5, 139.8, 136.2, 134.9, 130.7, 130.1, 130.1, 129.2, 129.2, 124.0, 123.5; *m*/*z* LRMS (ESI^+^) 244 [M+H]^+^; HRMS (ESI^+^) C_14_H_10_^35^ClNO [M+H]^+^ calcd 244.0524, found 244.0523.

#### 1-(4-Chlorophenyl)-3-(pyridin-3-yl)propan-1-one (**52c**)

Following general procedure D′, **52c** was obtained from **52d** (600 mg, 2.46 mmol). Purification by flash column chromatography (50% EtOAc in pentane) afforded the title compound as a yellow solid (468 mg, 78%): ^1^H NMR (400 MHz, CDCl_3_) δ 8.53 (d, *J* = 2.3 Hz, 1H), 8.46 (dd, *J* = 4.8, 1.6 Hz, 1H), 7.92–7.84 (m, 2H), 7.64–7.56 (m, 1H), 7.46–7.40 (m, 2H), 7.23 (ddt, *J* = 6.8, 4.9, 0.9 Hz, 1H), 3.29 (t, *J* = 7.4 Hz, 2H), 3.08 (t, *J* = 7.4 Hz, 2H); ^13^C NMR (101 MHz, CDCl_3_) δ 197.3, 149.8, 147.6, 139.9, 136.7, 136.5, 135.1, 129.5, 129.5, 129.2, 128.1, 123.6, 39.9, 27.2; *m*/*z* LRMS (ESI^+^) 246 [M+H]^+^; HRMS (ESI^+^) C_14_H_12_^35^ClNO [M+H]^+^ calcd 246.0680, found 246.0681.

#### 1-(4-Chlorophenyl)-3-(pyridin-3-yl)propan-1-ol (**52b**)

NaBH_4_ (46 mg, 1.22 mmol) was added to an ice-cold solution of **52c** (200 mg, 0.814 mmol) in MeOH (5 mL), and the resulting mixture was stirred at rt for 1 h. After completion, the reaction was quenched with water and concentrated *in vacuo*. The residue was purified by flash column chromatography (60% EtOAc in pentane) to afford title compound **52b** as a colorless oil (181 mg, 90%): ^1^H NMR (400 MHz, CDCl_3_) δ 8.43 (d, *J* = 2.3 Hz, 1H), 8.40 (dd, *J* = 4.9, 1.6 Hz, 1H), 7.57–7.50 (m, 1H), 7.36–7.21 (m, 5H), 4.66 (dd, *J* = 8.1, 5.0 Hz, 1H), 2.80–2.66 (m, 3H), 2.15–2.03 (m, 1H), 2.02–1.92 (m, 1H); ^13^C NMR (101 MHz, CDCl_3_) δ 149.6, 147.1, 143.1, 137.4, 136.5, 133.5, 128.8, 128.8, 127.4, 127.4, 123.7, 72.8, 40.2, 29.2; *m*/*z* LRMS (ESI^+^) 248 [M+H]^+^; HRMS (ESI^+^) C_14_H_14_^35^ClNO [M+H]^+^ calcd 248.0837, found 248.0836.

#### 3-(3-(4-Chlorophenyl)-3-fluoropropyl)pyridine (**52a**)

DAST (0.5 mL, 4.04 mmol) was added to an ice-cold solution of **52b** (100 mg, 0.403 mmol) in CHCl_3_ (5 mL), and the resulting mixture was stirred at 0 °C for 2 h. After completion, the reaction was quenched with NaHCO_3_ (sat. aq. sol., 5 mL) and the aqueous layer extracted with CH_2_Cl_2_ (2 × 20 mL). The combined organic phase was washed with H_2_O (20 mL) and brine (20 mL), dried (Na_2_SO_4_), filtered, and concentrated *in vacuo*. The residue was purified by flash column chromatography (70% EtOAc in pentane) to afford title compound **52a** as a colorless oil (87 mg, 87%): ^1^H NMR (400 MHz, CDCl_3_) δ 8.45 (dd, *J* = 4.8, 1.9 Hz, 2H), 7.49 (dt, *J* = 7.8, 2.0 Hz, 1H), 7.38–7.27 (m, 2H), 7.29–7.16 (m, 3H), 5.50–5.29 (m, 1H), 2.86–2.65 (m, 2H), 2.31–2.16 (m, 1H), 2.16–1.98 (m, 1H); ^13^C NMR (101 MHz, CDCl_3_) δ 149.9, 147.7, 138.3 (d, *J* = 20.0 Hz), 136.2, 136.0, 134.3 (d, *J* = 2.0 Hz), 128.8, 126.9 (d, *J* = 7.0 Hz), 123.5, 92.6 (d, *J* = 172.0 Hz), 38.4 (d, *J* = 24.0 Hz), 28.4 (d, *J* = 5.0 Hz); ^19^F NMR (377 MHz, CDCl_3_) δ −177.03; *m*/*z* LRMS (ESI^+^) 248 [M+H]^+^; HRMS (ESI^+^) C_14_H_13_ClFN [M+H]^+^ calcd 248.0837, found 248.0836.

#### 3-(3-(4-Chlorophenyl)-3-fluoropropyl)pyridine 1-oxide (**52**)

Following general procedure B, **52** was obtained **52a** (50 mg, 0.200 mmol). Purification by flash column chromatography (5% MeOH in CH_2_Cl_2_) afforded the title compound as a white solid (31.0 mg, 59%): ^1^H NMR (400 MHz, CDCl_3_) ^1^H NMR (400 MHz, CDCl_3_) δ 8.10 (dt, *J* = 7.9, 1.7 Hz, 2H), 7.39–7.32 (m, 2H), 7.22 (dd, *J* = 14.6, 7.2 Hz, 3H), 7.12 (dt, *J* = 8.0, 1.3 Hz, 1H), 5.41 (ddd, *J* = 47.6, 8.6, 4.0 Hz, 1H), 2.86–2.65 (m, 2H), 2.34–1.96 (m, 2H); ^13^C NMR (101 MHz, CDCl_3_) δ 140.2, 139.2, 138.0, 137.8, 137.4, 134.6 (d, *J* = 2.0 Hz), 129.0, 128.99, 126.8 (d, *J* = 7.0 Hz), 126.7, 125.9, 92.3 (d, *J* = 158.0 Hz), 37.7 (d, *J* = 24.0 Hz), 28.3 (d, *J* = 4.0 Hz); ^19^F NMR (376 MHz, CDCl_3_) δ −177.80; *m*/*z* LRMS (ESI^+^) 266 [M+H]^+^; HRMS (ESI^+^) C_14_H_13_^35^ClFNO [M+H]^+^ calcd 266.0742, found 266.0742; HPLC 94% (AUC), *t*_R_ = 4.4 min.

#### 3-(3-(4-Chlorophenyl)-3-hydroxypropyl)pyridine 1-oxide (**53**)

Following general procedure B, **53** was obtained from **52b** (60 mg, 0.242 mmol). Purification by flash column chromatography (5% MeOH in CH_2_Cl_2_) afforded the title compound as a white solid (45 mg, 70%): ^1^H NMR (400 MHz, CDCl_3_) δ 8.05 (brs, 1H), 7.96 (dq, *J* = 6.3, 2.2 Hz, 1H), 7.27–7.15 (m, 4H), 7.16–7.07 (m, 2H), 4.57 (dd, *J* = 8.3, 4.6 Hz, 1H), 2.68–2.58 (m, 2H), 2.05–1.92 (m, 1H), 1.91–1.80 (m, 1H); ^13^C NMR (101 MHz, CDCl_3_) δ 143.2, 141.4, 139.3, 137.0, 133.3, 128.7, 128.7, 127.8, 127.3, 127.3, 125.7, 72.0, 39.5, 28.9; *m*/*z* LRMS (ESI^+^) 264 [M+H]^+^; HRMS (ESI^+^) C_14_H_14_^35^ClNO_2_ [M+H]^+^ calcd 264.0786, found 264.0786; HPLC 92% (AUC), *t*_R_ = 3.1 min.

#### 3-(3-(4-Chlorophenyl)-3-oxopropyl)pyridine 1-oxide (**54**)

Following general procedure B, **54** was obtained from **52c** (50 mg, 0.203 mmol). Purification by flash column chromatography (5% MeOH in CH_2_Cl_2_) afforded the title compound as a white solid (39 mg, 74%): ^1^H NMR (400 MHz, CDCl_3_) δ 8.16 (dt, *J* = 1.9, 0.9 Hz, 1H), 8.11–8.04 (m, 1H), 7.90–7.84 (m, 2H), 7.47–7.40 (m, 2H), 7.22–7.16 (m, 2H), 3.29 (t, *J* = 7.2 Hz, 2H), 3.04 (t, *J* = 7.1 Hz, 2H); ^13^C NMR (101 MHz, CDCl_3_) δ 196.5, 140.4, 140.2, 139.3, 137.3, 134.8, 129.5, 129.5, 129.2, 129.2, 126.7, 125.8, 38.9, 26.7; *m*/*z* LRMS (ESI^+^) 262 [M+H]^+^; HRMS (ESI^+^) C_14_H_12_^35^ClNO_2_ [M+H]^+^ calcd 262.0629, found 262.0625; HPLC 95% (AUC), *t*_R_ = 3.6 min.

#### 3-Bromopyridine 1-oxide (**55a**)

Following general procedure B, **55a** was obtained from 3-bromopyridine (790 mg, 5.00 mmol). Purification by flash column chromatography (2% MeOH in CH_2_Cl_2_) afforded the title compound as a transparent oil (320 mg, 37%): ^1^H NMR (400 MHz, CDCl_3_) δ 8.35 (t, *J* = 1.7 Hz, 1H), 8.14 (ddd, *J* = 6.5, 1.7, 0.9 Hz, 1H), 7.40 (ddd, *J* = 8.3, 1.7, 0.9 Hz, 1H), 7.15 (dd, *J* = 8.3, 6.5 Hz, 1H); ^13^C NMR (101 MHz, CDCl_3_) δ 141.0, 138.2, 128.9, 126.2, 120.7; *m*/*z* LRMS (ESI^+^) 174 [M(^81^Br)+H]^+^ 176 [M(^79^Br)+H]^+^; HRMS (ESI^+^) C_5_H_4_^79^BrNO [M+H]^+^ calcd 173.9549, found 173.9550.

#### 3-((4-Chlorophenethyl)amino)pyridine 1-oxide (**55**)

To a vial with **55a** (200 mg, 1.16 mmol), 2-(4-chlorophenyl)ethan-1-amine (360 mg, 2.32 mmol), NaOtBu (334 mg, 3.48 mmol), and *rac*-BINAP (144 mg, 0.232 mmol) was added degassed toluene (10 mL). The mixture was degassed for 5 min before adding Pd_2_(dba)_3_ (106 mg, 0.116 mmol). The mixture was degassed for another 5 min, then sealed and heated to 80 °C for 16 h. After completion, the mixture was diluted with EtOAc, filtered through Celite, and concentrated *in vacuo*. The compound was then purified by flash column chromatography (4% MeOH in CH_2_Cl_2_) to afford the title compound as a black oil (43 mg, 15%):^1^H NMR (400 MHz, CDCl_3_) δ 8.30 (s, 1H), 7.61 (dd, *J* = 6.5, 1.8 Hz, 1H), 7.29–7.26 (m, 2H), 7.20–7.16 (m, 2H), 7.13 (dd, *J* = 8.6, 6.2 Hz, 1H), 6.67 (dd, *J* = 8.7, 2.2 Hz, 1H), 5.86 (s, 1H), 3.36 (t, *J* = 7.3 Hz, 2H), 2.97 (t, *J* = 7.2 Hz, 2H); ^13^C NMR (101 MHz, CDCl_3_) δ 147.7, 137.1, 132.7, 130.3, 130.3, 129.0, 129.0, 127.2, 126.3, 126.0, 113.7, 44.8, 34.5; *m*/*z* LRMS (ESI^+^) 251 [M(^37^Cl)+H]^+^ 249 [M(^35^Cl)+H]^+^; HRMS (ESI^+^) C_13_H_13_^35^ClN_2_O [M+H]^+^ calcd 249.0789, found 249.0790; HPLC 95% (AUC), *t*_R_ = 4.6 min.

#### 2-(6-Methoxypyridin-2-yl)ethan-1-ol (**56a**)

Following general procedure F, **56a** was obtained from 2-methoxy-6-methylpyridine (500 mg, 4.06 mmol). Purification by flash column chromatography (50% EtOAc in pentane) afforded the title compound as a yellow oil (112 mg, 18%): ^1^H NMR (400 MHz, CDCl_3_) δ 7.48 (dd, *J* = 8.3, 7.2 Hz, 1H), 6.70 (dd, *J* = 7.3, 0.8 Hz, 1H), 6.59 (dd, *J* = 8.3, 0.8 Hz, 1H), 3.98 (t, 2H), 3.88 (s, 3H), 2.91 (t, 2H); ^13^C NMR (101 MHz, CDCl_3_) δ 163.6, 158.3, 139.3, 115.8, 108.6, 62.1, 53.4, 38.5; *m*/*z* LRMS (ESI^+^) 154 [M+H]^+^; HRMS (ESI^+^) C_8_H_11_NO_2_ [M+H]^+^ calcd 154.0863, found 154.0862.

#### 2-(2-((4-Chloronaphthalen-1-yl)oxy)ethyl)-6-methoxypyridine (**56**)

Following general procedure A, **56** was obtained from 4-chloro-1-naphthol (141 mg, 0.792 mmol) and **56a** (80 mg, 0.526 mmol). Purification by flash column chromatography (50% CH_2_Cl_2_ in pentane) afforded the title compound as a brown solid (85 mg, 52%): ^1^H NMR (400 MHz, CDCl_3_) δ 8.25–8.15 (m, 2H), 7.59 (ddd, *J* = 8.3, 6.8, 1.3 Hz, 1H), 7.55–7.47 (m, 2H), 7.44 (d, *J* = 8.2 Hz, 1H), 6.89 (dd, *J* = 7.2, 0.7 Hz, 1H), 6.78 (d, *J* = 8.3 Hz, 1H), 6.61 (dd, *J* = 8.3, 0.7 Hz, 1H), 4.53 (t, *J* = 6.6 Hz, 2H), 3.92 (s, 3H), 3.31 (t, *J* = 6.6 Hz, 2H); ^13^C NMR (101 MHz, CDCl_3_) δ 164.0, 156.1, 153.9, 139.1, 131.5, 127.5, 126.9, 125.9, 125.9, 124.3, 123.3, 122.6, 116.3, 108.4, 105.0, 67.7, 53.4, 37.7; *m*/*z* LRMS (ESI^+^) 314 [M+H]^+^; HRMS (ESI^+^) C_18_H_16_^35^ClNO_2_ [M+H]^+^ calcd 314.0942, found 314.0944; HPLC 95% (AUC), *t*_R_ = 8.3 min.

#### 2-(2-Methoxypyridin-4-yl)ethan-1-ol (**57a**)

Following general procedure F, **57a** was obtained from 2-methoxy-4-methylpyridine (500 mg, 4.06 mmol). Purification by flash column chromatography (50% EtOAc in pentane) afforded the title compound as a yellow oil (175 mg, 28%): ^1^H NMR (400 MHz, CDCl_3_) δ 8.05 (dd, *J* = 5.2, 0.7 Hz, 1H), 6.75 (dd, *J* = 5.3, 1.4 Hz, 1H), 6.61 (dq, *J* = 1.4, 0.7 Hz, 1H), 3.91 (s, 3H), 3.86 (t, *J* = 6.5 Hz, 2H), 2.80 (t, *J* = 6.5 Hz, 2H); ^13^C NMR (101 MHz, CDCl_3_) δ 164.7, 150.8, 146.9, 117.9, 111.1, 62.6, 53.5, 38.5; *m*/*z* LRMS (ESI^+^) 154 [M+H]^+^; HRMS (ESI^+^) C_8_H_11_NO_2_ [M+H]^+^ calcd 154.0863, found 154.0862.

#### 4-(2-((4-Chloronaphthalen-1-yl)oxy)ethyl)-2-methoxypyridine (**57**)

Following general procedure A, **57** was obtained from 4-chloro-1-naphthanol (300 mg, 1.67 mmol) and **57a** (170 mg, 1.12 mmol). Purification by flash column chromatography (50% CH_2_Cl_2_ in pentane) afforded the title compound as a yellow oil (163 mg, 47%): ^1^H NMR (400 MHz, CDCl_3_) δ 8.25–8.13 (m, 2H), 8.12 (dd, *J* = 5.3, 0.7 Hz, 1H), 7.61 (ddd, *J* = 8.4, 6.9, 1.4 Hz, 1H), 7.53 (ddd, *J* = 8.2, 6.8, 1.3 Hz, 1H), 7.43 (d, *J* = 8.2 Hz, 1H), 6.89 (dd, *J* = 5.3, 1.5 Hz, 1H), 6.75 (dd, *J* = 1.5, 0.7 Hz, 1H), 6.71 (d, *J* = 8.2 Hz, 1H), 4.36 (t, *J* = 6.5 Hz, 2H), 3.94 (s, 3H), 3.20 (t, *J* = 6.5 Hz, 2H); ^13^C NMR (101 MHz, CDCl_3_) δ 164.7, 153.5, 150.3, 146.9, 131.5, 127.7, 126.7, 126.2, 125.8, 124.4, 123.6, 122.5, 117.9, 111.1, 104.8, 67.8, 53.5, 35.1; *m*/*z* LRMS (ESI^+^) 316 [M(^37^Cl)+H]^+^ 314 [M(^35^Cl)+H]^+^; HRMS (ESI^+^) C_18_H_16_ClNO_2_ [M+H]^+^ calcd 314.0942, found 314.0947; HPLC 96% (AUC), *t*_R_ = 7.9 min.

#### 6-(2-((4-Chloronaphthalen-1-yl)oxy)ethyl)pyridin-2-ol (**58**)

Following general procedure G, **58** was obtained from **56** (20 mg, 0.064 mmol). Purification by flash column chromatography (5% MeOH in CH_2_Cl_2_) afforded the title compound as a white solid (19 mg, 99%): ^1^H NMR (600 MHz, DMSO) δ 11.73 (s, 1H), 8.18 (d, *J* = 8.4 Hz, 1H), 8.10 (d, *J* = 8.3 Hz, 1H), 7.69 (ddd, *J* = 8.3, 6.8, 1.3 Hz, 1H), 7.63–7.57 (m, 2H), 7.37 (dd, *J* = 9.1, 6.8 Hz, 1H), 7.01 (d, *J* = 8.3 Hz, 1H), 6.25–6.14 (m, 2H), 4.41 (t, *J* = 6.3 Hz, 2H), 3.06 (t, *J* = 6.3 Hz, 2H); ^13^C NMR (151 MHz, DMSO) δ 163.1, 153.0, 146.4, 141.0, 130.4, 128.0, 126.3, 126.3, 125.9, 123.6, 122.2, 121.8, 117.4, 105.8, 104.5, 66.5, 32.3; *m*/*z* LRMS (ESI^+^) 302 [M(^37^Cl)+H]^+^ 300 [M(^35^Cl)+H]^+^; HRMS (ESI^+^) C_17_H_14_ClNO_2_ [M+H]^+^ calcd 300.0786, found 300.0785; HPLC 95% (AUC), *t*_R_ = 6.2 min.

#### 4-(2-((4-Chloronaphthalen-1-yl)oxy)ethyl)pyridin-2-ol (**59**)

Following general procedure G, **59** was obtained from **57** (60 mg, 0.192 mmol). Purification by flash column chromatography (5% MeOH in CH_2_Cl_2_) afforded the title compound as a white solid (38 mg, 66%): ^1^H NMR (400 MHz, MeOD) δ 8.19 (dt, *J* = 8.3, 1.0 Hz, 1H), 8.14 (dt, *J* = 8.5, 1.0 Hz, 1H), 7.61 (ddd, *J* = 8.4, 6.9, 1.3 Hz, 1H), 7.52 (ddd, *J* = 8.2, 6.8, 1.2 Hz, 1H), 7.47 (d, *J* = 8.3 Hz, 1H), 7.38 (dd, *J* = 6.7, 0.7 Hz, 1H), 6.89 (d, *J* = 8.3 Hz, 1H), 6.58 (dd, *J* = 1.7, 0.8 Hz, 1H), 6.51 (dd, *J* = 6.7, 1.7 Hz, 1H), 4.42 (t, *J* = 6.1 Hz, 2H), 3.14 (t, *J* = 6.0 Hz, 2H); ^13^C NMR (101 MHz, MeOD) δ 165.8, 156.9, 154.9, 135.4, 132.5, 128.6, 127.9, 127.1, 127.0, 125.0, 124.2, 123.4, 119.8, 110.3, 106.1, 68.3, 36.3; *m*/*z* LRMS (ESI^+^) 302 [M(^37^Cl)+H]^+^ 300 [M(^35^Cl)+H]^+^; HRMS (ESI^+^) C_17_H_14_^35^ClNO_2_ [M+H]^+^ calcd 300.0786, found 300.0786; HPLC 96% (AUC), *t*_R_ = 6.2 min.

#### 2-(2-(4-Chlorophenoxy)ethyl)-6-methoxypyridine (**60a**)

Following general procedure B, **60a** was obtained from 4-chlorophenol (100 mg, 0.784 mmol) and **56a** (80.0 mg, 0.523 mmol). Purification by flash column chromatography (50% CH_2_Cl_2_ in pentane) afforded the title compound as a transparent oil (80.0 mg, 58%): ^1^H NMR (400 MHz, CDCl_3_) δ 7.49 (dd, *J* = 8.3, 7.2 Hz, 1H), 7.24–7.19 (m, 2H), 6.87–6.82 (m, 2H), 6.81–6.78 (m, 1H), 6.59 (dd, *J* = 8.3, 0.8 Hz, 1H), 4.34 (t, *J* = 6.8 Hz, 2H), 3.91 (s, 3H), 3.15 (t, *J* = 6.8 Hz, 2H); ^13^C NMR (101 MHz, CDCl_3_) δ 163.9, 157.7, 155.9, 139.0, 129.4, 129.4, 125.6, 116.2, 116.1, 116.1, 108.4, 67.5, 53.4, 37.6; *m*/*z* LRMS (ESI^+^) 266 [M(^37^Cl)+H]^+^ 264 [M(^35^Cl)+H]^+^; HRMS (ESI^+^) C_14_H_14_ClNO_2_ [M+H]^+^ calcd 264.0786, found 264.0786.

#### 6-(2-(4-Chlorophenoxy)ethyl)pyridin-2-ol (**60**)

Following general procedure G, **60** was obtained from **60a** (40 mg, 0.152 mmol). Purification by flash column chromatography (5% MeOH in CH_2_Cl_2_) afforded the title compound as a white solid (34 mg, 90%): ^1^H NMR (600 MHz, MeOD) δ 7.53 (dd, *J* = 9.1, 6.9 Hz, 1H), 7.26–7.21 (m, 2H), 6.93–6.87 (m, 2H), 6.41 (dd, *J* = 9.1, 1.0 Hz, 1H), 6.34 (dd, *J* = 6.9, 1.0 Hz, 1H), 4.23 (t, *J* = 6.2 Hz, 2H), 3.03 (t, *J* = 6.2 Hz, 2H); ^13^C NMR (101 MHz, CDCl_3_) δ 165.8, 157.1, 146.3, 142.1, 129.5, 129.5, 126.2, 117.9, 117.9, 116.1, 106.9, 66.3, 33.3; *m*/*z* LRMS (ESI^+^) 252 [M(^37^Cl)+H]^+^ 250 [M(^35^Cl)+H]^+^; HRMS (ESI^+^) C_13_H_12_^35^ClNO_2_ [M+H]^+^ calcd 250.0629, found 250.0631; HPLC 96% (AUC), *t*_R_ = 4.0 min.

#### 5-(2-((4-Chloronaphthalen-1-yl)oxy)ethyl)-2-methylpyridine (**61a**)

Following general procedure A, **61a** was obtained from 4-chloro-1-naphthanol (75 mg, 0.418 mmol) and 2-(6-methyl-3-pyridinyl)ethanol (38 mg, 0.279 mmol). Purification by flash column chromatography (4% MeOH in CH_2_Cl_2_) afforded the title compound as a yellow-beige solid (48 mg, 58%): ^1^H NMR (400 MHz, CDCl_3_) δ 8.53 (d, *J* = 2.3 Hz, 1H), 8.23 (dd, *J* = 8.0, 1.2 Hz, 1H), 8.19 (dd, *J* = 7.9, 1.1 Hz, 1H), 7.60 (dtd, *J* = 8.0, 6.8, 1.9 Hz, 2H), 7.53 (ddd, *J* = 8.2, 6.8, 1.3 Hz, 1H), 7.42 (d, *J* = 8.2 Hz, 1H), 7.12 (d, *J* = 7.9 Hz, 1H), 6.69 (d, *J* = 8.2 Hz, 1H), 4.32 (t, *J* = 6.4 Hz, 2H), 3.20 (t, *J* = 6.4 Hz, 2H), 2.54 (s, 3H); *m*/*z* LRMS (ESI^+^) 298 [M+H]^+^.

#### 5-(2-((4-Chloronaphthalen-1-yl)oxy)ethyl)-2-methylpyridine 1-oxide (**61**)

Following general procedure B′, **61** was obtained from **61a** (18 mg, 0.060 mmol). Purification by flash column chromatography (5% MeOH in CH_2_Cl_2_) afforded the title compound as a light orange solid (17 mg, 90%): ^1^H NMR (400 MHz, CDCl_3_) δ 8.75 (dt, *J* = 8.7, 0.9 Hz, 1H), 8.39 (s, 1H), 8.37 (d, *J* = 8.7 Hz, 1H), 8.28 (dd, *J* = 8.2, 1.1 Hz, 1H), 7.74 (ddd, *J* = 8.6, 6.9, 1.4 Hz, 1H), 7.61 (ddd, *J* = 8.2, 6.9, 1.2 Hz, 1H), 7.25–7.22 (m, 2H), 6.80 (d, *J* = 8.7 Hz, 1H), 4.47 (t, *J* = 6.2 Hz, 2H), 3.24 (t, *J* = 6.1 Hz, 2H), 2.52 (s, 3H); ^13^C NMR (101 MHz, CDCl_3_) δ 139.6, 134.7, 130.3, 127.1, 127.0, 126.6, 123.7, 122.6, 102.7, 68.3, 32.3, 17.7; *m*/*z* LRMS (ESI^+^) 314 [M+H]^+^; HRMS (ESI^+^) C_18_H_16_^35^ClNO_2_ [M+H]^+^ calcd 314.0943, found 314.0934; HPLC 97% (AUC), *t*_R_ = 5.9 min.

#### 2-(6-Vinylpyridin-3-yl)ethan-1-ol (**62c**)

A mixture of 2-(6-chloropyridin-3-yl)ethan-1-ol (660 mg, 4.20 mmol), vinylboronic acid pinacol ester (970 mg, 6.30 mmol), and K_2_CO_3_ (1.45 g, 10.5 mmol) in 1,4-dioxane (10 mL) and water (5 mL) was degassed with N_2_ for 5 min before addition of Pd(dppf)Cl_2_ (310 mg, 0.420 mmol). After another 10 min degassing, the mixture was sealed and heated to 100 °C for 16 h. After completion, the mixture was cooled to rt, diluted with EtOAc, filtered through Celite and concentrated *in vacuo*. The residue was then purified by flash column chromatography (3% MeOH in CH_2_Cl_2_) to afford the title compound **62c** as a brown oil (400 mg, 64%).

#### 5-(2-((4-Chloronaphthalen-1-yl)oxy)ethyl)-2-vinylpyridine (**62b**)

Following general procedure A, **62b** was obtained from 4-chloro-1-naphthol (535 mg, 3.00 mmol) and 2-(6-vinylpyridin-3-yl)ethan-1-ol (300 mg, 2.00 mmol). Purification by flash column chromatography (2% MeOH in CH_2_Cl_2_) afforded the title compound as a brown oil (414 mg, 67%): ^1^H NMR (400 MHz, CDCl_3_) δ 8.60 (d, *J* = 2.3 Hz, 1H), 8.27–8.15 (m, 2H), 7.65 (dd, *J* = 8.0, 2.3 Hz, 1H), 7.61 (ddd, *J* = 8.4, 6.8, 1.4 Hz, 1H), 7.53 (ddd, *J* = 8.3, 6.9, 1.3 Hz, 1H), 7.42 (d, *J* = 8.3 Hz, 1H), 7.32 (dd, *J* = 8.0, 0.8 Hz, 1H), 6.81 (dd, *J* = 17.5, 10.8 Hz, 1H), 6.69 (d, *J* = 8.2 Hz, 1H), 6.16 (dd, *J* = 17.5, 1.2 Hz, 1H), 5.46 (dd, *J* = 10.8, 1.2 Hz, 1H), 4.33 (t, *J* = 6.4 Hz, 2H), 3.23 (t, *J* = 6.4 Hz, 2H); ^13^C NMR (101 MHz, CDCl_3_) δ 154.5, 153.6, 150.2, 137.1, 136.8, 132.8, 131.5, 127.7, 126.7, 126.2, 125.8, 124.4, 123.7, 122.5, 121.1, 117.9, 104.8, 68.5, 32.9; *m*/*z* LRMS (ESI^+^) 312 [M(^37^Cl)+H]^+^ 310 [M(^35^Cl)+H]^+^; HRMS (ESI^+^) C_19_H_16_^35^ClNO [M+H]^+^ calcd 310.0993, found 310.0988.

#### 5-(2-((4-Chloronaphthalen-1-yl)oxy)ethyl)-2-ethylpyridine (**62a**)

Following general procedure D′, **62a** was obtained from **62b** (40 mg, 0.129 mmol) as a pale brown oil (32 mg, 80%): ^1^H NMR (400 MHz, CDCl_3_) δ 8.55 (d, *J* = 2.3 Hz, 1H), 8.27–8.20 (m, 1H), 8.18 (dt, *J* = 8.4, 1.1 Hz, 1H), 7.62–7.56 (m, 2H), 7.52 (ddd, *J* = 8.2, 6.9, 1.3 Hz, 1H), 7.40 (d, *J* = 8.2 Hz, 1H), 7.12 (d, *J* = 7.9 Hz, 1H), 6.66 (d, *J* = 8.2 Hz, 1H), 4.28 (t, *J* = 6.4 Hz, 2H), 3.18 (t, *J* = 6.4 Hz, 2H), 2.81 (q, *J* = 7.6 Hz, 2H), 1.30 (t, *J* = 7.6 Hz, 3H); ^13^C NMR (101 MHz, CDCl_3_) δ 161.9, 153.6, 149.8, 137.0, 131.4, 131.0, 127.6, 126.7, 126.1, 125.8, 124.3, 123.5, 122.5, 121.9, 104.7, 68.6, 32.7, 31.1, 14.0; *m*/*z* LRMS (ESI^+^) 314 [M(^37^Cl)+H]^+^ 312 [M(^35^Cl)+H]^+^; HRMS (ESI^+^) C_19_H_18_^35^ClNO [M+H]^+^ calcd 312.115, found 312.1143.

#### 5-(2-((4-Chloronaphthalen-1-yl)oxy)ethyl)-2-ethylpyridine 1-oxide (**62**)

Following general procedure B, **62** was obtained from **62a** (40 mg, 0.129 mmol). Purification by flash column chromatography (6% MeOH in CH_2_Cl_2_) afforded the title compound as a pink oil (36 mg, 86%): ^1^H NMR (400 MHz, CDCl_3_) δ 8.35 (d, *J* = 1.7 Hz, 1H), 8.19 (ddt, *J* = 8.7, 1.4, 0.8 Hz, 2H), 7.61 (ddd, *J* = 8.5, 6.9, 1.4 Hz, 1H), 7.54 (ddd, *J* = 8.1, 6.9, 1.3 Hz, 1H), 7.43 (d, *J* = 8.2 Hz, 1H), 7.27 (dd, *J* = 8.1, 1.7 Hz, 1H), 7.20 (d, *J* = 8.0 Hz, 1H), 6.69 (d, *J* = 8.3 Hz, 1H), 4.33 (t, *J* = 6.2 Hz, 2H), 3.17 (t, *J* = 6.2 Hz, 2H), 2.94 (q, *J* = 7.5 Hz, 2H), 1.30 (t, *J* = 7.5 Hz, 4H); ^13^C NMR (101 MHz, CDCl_3_) δ 153.3, 152.1, 139.7, 134.8, 131.5, 127.7, 127.2, 126.6, 126.3, 125.7, 124.5, 124.3, 123.9, 122.3, 104.9, 67.8, 32.5, 23.6, 10.7; *m*/*z* LRMS (ESI^+^) 330 [M(^37^Cl)+H]^+^ 328 [M(^35^Cl)+H]^+^; HRMS (ESI^+^) C_19_H_18_ClNO_2_ [M+H]^+^ calcd 328.1099, found 328.1097; HPLC 96% (AUC), *t*_R_ = 7.1 min.

#### 3-(2-((4-Chloronaphthalen-1-yl)oxy)ethyl)-2,6-dimethylpyridine (**63a**)

Following general procedure A, **63a** was obtained from 2-(2,6-dimethylpyridin-3-yl)ethan-1-ol (92 mg, 0.609 mmol) and 4-chloronaphthol (163 mg, 0.914 mmol.) Purification by flash column chromatography (2% MeOH in CH_2_Cl_2_) afforded the title compound as a yellow solid (168 mg, 89%): ^1^H NMR (400 MHz, CDCl_3_) δ 8.22 (ddd, *J* = 8.4, 1.4, 0.7 Hz, 1H), 8.19 (dt, *J* = 8.4, 1.0 Hz, 1H), 7.60 (ddd, *J* = 8.4, 6.9, 1.4 Hz, 1H), 7.52 (ddd, *J* = 8.3, 6.9, 1.3 Hz, 1H), 7.47 (d, *J* = 7.8 Hz, 1H), 7.42 (d, *J* = 8.2 Hz, 1H), 6.96 (d, *J* = 7.8 Hz, 1H), 6.69 (d, *J* = 8.3 Hz, 1H), 4.29 (t, *J* = 6.7 Hz, 2H), 3.21 (t, *J* = 6.7 Hz, 2H), 2.62 (s, 3H), 2.51 (s, 3H); ^13^C NMR (101 MHz, CDCl_3_) δ 156.1, 156.0, 153.7, 137.7, 131.5, 128.3, 127.6, 126.7, 126.1, 125.8, 124.4, 123.6, 122.5, 121.0, 104.8, 67.7, 32.2, 24.2, 22.5; *m*/*z* LRMS (ESI^+^) 312 [M+H]^+^.

#### 3-(2-((4-Chloronaphthalen-1-yl)oxy)ethyl)-2,6-dimethylpyridine 1-oxide (**63**)

Following general procedure B, **63** was obtained from **63a** (27 mg, 0.087 mmol). Purification by flash column chromatography (5% MeOH in CH_2_Cl_2_) afforded the title compound as a beige solid (27 mg, 95%): ^1^H NMR (400 MHz, CDCl_3_) δ 8.18 (td, *J* = 8.5, 1.2 Hz, 2H), 7.61 (ddd, *J* = 8.4, 6.9, 1.3 Hz, 1H), 7.52 (ddd, *J* = 8.2, 6.9, 1.3 Hz, 1H), 7.43 (d, *J* = 8.2 Hz, 1H), 7.16 (d, *J* = 8.1 Hz, 1H), 7.11 (d, *J* = 8.0 Hz, 1H), 6.69 (d, *J* = 8.3 Hz, 1H), 4.32 (t, *J* = 6.4 Hz, 2H), 3.27 (t, *J* = 6.5 Hz, 2H), 2.66 (s, 3H), 2.52 (s, 3H); ^13^C NMR (101 MHz, CDCl_3_) δ 153.4, 148.7, 132.7, 131.5, 127.7, 126.6, 126.3, 125.7, 125.7, 124.5, 124.5, 123.9, 123.0, 122.3, 104.8, 67.4, 33.0, 18.5, 14.6; *m*/*z* LRMS (ESI^+^) 328 [M+H]^+^; HRMS (ESI^+^) C_19_H_18_^35^ClNO_2_ [M+H]^+^ calcd 328.1099, found 328.1139; HPLC 99% (AUC), *t*_R_ = 8.0 min.

#### (E)-1-(4-Chloronaphthalen-1-yl)-3-(pyridin-3-yl)prop-2-en-1-one (**64c**)

Following general procedure C, **64c** was obtainsed from 1-(4-chloronaphthalen-1-yl)ethan-1-one (500 mg, 2.44 mmol) and nicotinaldehyde (523 mg, 4.89 mmol) as a yellow solid (705 mg, 98%): ^1^H NMR (400 MHz, CDCl_3_) δ 8.70 (d, *J* = 2.3 Hz, 1H), 8.55 (dd, *J* = 4.8, 1.7 Hz, 1H), 8.34–8.24 (m, 2H), 7.83 (dt, *J* = 7.9, 2.0 Hz, 1H), 7.64–7.54 (m, 4H), 7.52 (d, *J* = 16.3 Hz, 1H), 7.30–7.27 (m, 1H), 7.21 (d, *J* = 24.6 Hz, 1H); ^13^C NMR (101 MHz, CDCl_3_) δ 194.2, 151.6, 150.3, 142.4, 136.2, 135.8, 134.7, 131.7, 131.3, 130.4, 128.6, 128.5, 127.9, 127.2, 126.1, 125.1, 125.1, 124.0,; *m*/*z* LRMS (ESI^+^) 296 [M(^37^Cl)+H]^+^ 294 [M(^35^Cl)+H]^+^; HRMS (ESI^+^) C_18_H_12_^35^ClNO [M+H]^+^ calcd 294.0680, found 294.0676.

#### (*E*)-3-(3-(4-Chloronaphthalen-1-yl)prop-1-en-1-yl)pyridine (**64b**)

Triethylsilane (5 mL) was added to a solution of **64c** (705 mg, 2.41 mmol) in TFA (5 mL). The mixture was stirred at rt for 48 h. After completion the reaction was quenched with NaHCO_3_ (aq. sat. sol., 30 mL), extracted with CH_2_Cl_2_ (2 × 20 mL), the organic layer washed with brine, water, dried (Na_2_SO_4_), and concentrated *in vacuo* to give the crude compound as a brown oil. Purification by flash column chromatography (0–60% EtOAc in pentane) afforded the title compound as a brown oil (500 mg, 75%).

#### 3-(3-(4-Chloronaphthalen-1-yl)propyl)pyridine (**64a**)

Following general procedure D′, **64a** was obtained **64b** (500 mg, 1.79 mmol). Purification by flash column chromatography (30% EtOAc in pentane) afforded the title compound as a transparent oil (195 mg, 39%): ^1^H NMR (400 MHz, CDCl_3_) δ 8.48–8.43 (m, 2H), 8.31–8.25 (m, 1H), 7.91–7.86 (m, 1H), 7.57–7.47 (m, 2H), 7.45–7.38 (m, 2H), 7.16–7.10 (m, 2H), 2.99 (t, 2H), 2.64 (t, *J* = 7.7 Hz, 2H), 2.03–1.92 (m, 2H); ^13^C NMR (101 MHz, CDCl_3_) δ 149.8, 147.4, 137.1, 136.9, 135.6, 132.7, 130.8, 130.1, 126.5, 126.5, 125.7, 125.6, 125.1, 123.9, 123.2, 32.6, 32.0, 31.6; *m*/*z* LRMS (ESI^+^) 284 [M(^37^Cl)+H]^+^ 282 [M(^35^Cl)+H]^+^; HRMS (ESI^+^) C_18_H_16_^35^ClN [M+H]^+^ calcd 282.1044, found 282.1035.

#### 3-(3-(4-Chloronaphthalen-1-yl)propyl)pyridine 1-oxide (**64**)

Following general procedure B, **64** was obtained from **64a** (60 mg, 0.213 mmol). Purification by flash column chromatography (5% MeOH in CH_2_Cl_2_) afforded the title compound as a transparent oil (47 mg, 74%): ^1^H NMR (400 MHz, CDCl_3_) δ 8.33–8.26 (m, 1H), 8.08 (td, *J* = 1.7, 0.7 Hz, 1H), 8.05 (dt, *J* = 6.2, 1.4 Hz, 1H), 7.95–7.90 (m, 1H), 7.61–7.52 (m, 2H), 7.46 (d, *J* = 7.6 Hz, 1H), 7.19–7.13 (m, 2H), 7.08–7.04 (m, 1H), 3.06 (t, 2H), 2.64 (t, 2H), 2.08–1.98 (m, 2H); ^13^C NMR (101 MHz, CDCl_3_) δ 141.03, 139.09, 137.04, 136.59, 132.79, 131.07, 130.58, 126.81, 126.78, 126.40, 126.02, 125.78, 125.63, 125.37, 123.94, 32.48, 32.10, 31.06.; *m*/*z* LRMS (ESI^+^) 300 [M(^37^Cl)+H]^+^ 298 [M(^35^Cl)+H]^+^; HRMS (ESI^+^) C_18_H_16_^35^ClNO [M+H]^+^ calcd 298.0993, found 298.0987; HPLC 99% (AUC), *t*_R_ = 7.3 min.

#### 1-(4-Chloronaphthalen-1-yl)ethan-1-one (**65d**)

To a solution of 1-chloronaphthalene (2.72 mL, 20 mmol) in CH_2_Cl_2_ (50 mL) were added acetyl chloride (1.56 mL, 22 mmol) and AlCl_3_ (4.00 g, 30 mmol) at 0 °C. The reaction was warmed to rt and stirred for 16 h. After completion, the reaction was poured onto 1 M HCl (1M, aq., 30 mL), and the organic layer was washed with water (3 × 30 mL) and brine (3 × 30 mL), dried (Na_2_SO_4_), and concentrated *in vacuo*. The crude product was purified by flash column chromatography (10% EtOAc/pentane) to afford title compound **65d** as a yellow oil (1.90 g, 47%): ^1^H NMR (400 MHz, CDCl_3_) δ 8.83–8.71 (m, 1H), 8.34–8.25 (m, 1H), 7.76 (d, *J* = 7.8 Hz, 1H), 7.65–7.58 (m, 2H), 7.52 (d, *J* = 7.8 Hz, 1H), 2.69 (s, 3H); ^13^C NMR (101 MHz, CDCl_3_) δ 200.9, 136.8, 134.4, 131.3, 131.1, 128.7, 128.4, 127.5, 126.5, 124.8, 124.7, 29.9; *m*/*z* LRMS (ESI^+^) 207 [M(^37^Cl)+H]^+^ 205 [M(^35^Cl)+H]^+^; HRMS (ESI^+^) C_12_H_9_^35^ClO [M+H]^+^ calcd 205.0415, found 205.0413.

#### (*E*)-1-(4-Chloronaphthalen-1-yl)-3-(5-methoxypyridin-3-yl)prop-2-en-1-one (**65c**)

To a solution of **65d** (516 mg, 2.53 mmol) and 5-methoxynicotinaldehyde (693 mg, 5.06 mmol) in 1,4-dioxane (5 mL) was added BF_3_·Et_2_O (2.5 mL), and the resulting mixture was stirred at 105 °C for 16 h. After completion, the reaction was cooled to rt before addition of NaHCO_3_ (aq. sat. sol., 80 mL) and the mixture extracted with EtOAc (3 × 50 mL). The combined organic phase was washed with brine (100 mL), dried (Na_2_SO_4_), filtered, and concentrated *in vacuo*. Purification of the resulting residue by flash column chromatography (0–60% EtOAc in pentane) afforded title compound (*E*)-1-(4-chloronaphthalen-1-yl)-3-(5-methoxypyridin-3-yl)prop-2-en-1-one 65c as a yellow oil (640 mg, 78%): ^1^H NMR (400 MHz, CDCl_3_) δ 8.40–8.30 (m, 4H), 7.70–7.59 (m, 4H), 7.56 (d, *J* = 16.1 Hz, 1H), 7.34 (dd, *J* = 2.8, 1.8 Hz, 1H), 7.30 (d, *J* = 16.1 Hz, 1H), 3.87 (s, 3H); ^13^C NMR (101 MHz, CDCl_3_) δ 194.1, 155.9, 142.6, 142.4, 140.0, 136.1, 135.7, 131.7, 131.2, 130.9, 128.7, 128.5, 127.8, 127.2, 126.1, 125.0, 125.0, 118.0, 55.8; *m*/*z* LRMS (ESI^+^) 325 [M(^37^Cl)+H]^+^ 323 [M(^35^Cl)+H]^+^; HRMS (ESI^+^) C_19_H_14_^35^ClNO_2_ [M+H]^+^ calcd 324.0786, found 324.0788.

#### (*E*)-3-(3-(4-Chloronaphthalen-1-yl)prop-1-en-1-yl)-5-methoxypyridine (**65b**)

To a solution of **65c** (840 mg, 2.59 mmol) in TFA (3 mL) was added triethylsilane (3 mL), and the resulting mixture was stirred at rt for 48 h. After completion, the mixture was diluted with CH_2_Cl_2_ (20 mL), quenched with NaOH (1M, aq., 20 mL), and extracted with CH_2_Cl_2_ (3 × 20 mL). The combined organic phase was washed with brine (50 mL), dried (Na_2_SO_4_), filtered, and concentrated *in vacuo*. Purification of the resulting residue by flash column chromatography (0–60% EtOAc in pentane) to afford title compound **65b** as a pale yellow oil (400 mg, 50%): ^1^H NMR (400 MHz, CDCl_3_) δ 8.25–8.20 (m, 1H), 8.04 (dd, *J* = 5.8, 2.3 Hz, 2H), 7.97–7.91 (m, 1H), 7.54–7.42 (m, 2H), 7.41 (d, *J* = 7.6 Hz, 1H), 7.17 (d, *J* = 7.7 Hz, 1H), 6.99 (dd, *J* = 2.7, 1.8 Hz, 1H), 6.41 (dt, *J* = 15.9, 6.3 Hz, 1H), 6.26 (dt, *J* = 15.9, 1.6 Hz, 1H), 3.85 (d, *J* = 6.1 Hz, 2H), 3.69 (s, 3H); ^13^C NMR (101 MHz, CDCl_3_) δ 155.7, 140.7, 136.5, 135.0, 133.5, 133.0, 131.1, 131.0, 128.0, 126.9, 126.9, 126.5, 126.0, 125.3, 124.4, 124.4, 116.7, 55.5, 36.3; *m*/*z* LRMS (ESI^+^) 310 [M(^37^Cl)+H]^+^ 312 [M(^35^Cl)+H]^+^; HRMS (ESI^+^) C_19_H_16_^35^ClNO_2_ [M+H]^+^ calcd 310.0993, found 310.0989.

#### 3-(3-(4-Chloronaphthalen-1-yl)propyl)-5-methoxypyridine (**65a**)

Following general procedure D′, **65a** was obtained from **65b** (370 mg, 1.20 mmol) as a pale yellow oil (230 mg, 62%): ^1^H NMR (400 MHz, CDCl_3_) δ 8.26–8.20 (m, 1H), 8.08 (d, *J* = 2.8 Hz, 1H), 8.05–8.00 (m, 1H), 7.90–7.84 (m, 1H), 7.54–7.44 (m, 2H), 7.40 (d, *J* = 7.6 Hz, 1H), 7.13 (d, *J* = 7.6 Hz, 1H), 6.92 (dd, *J* = 2.7, 1.7 Hz, 1H), 3.74 (s, 3H), 3.04–2.94 (m, 2H), 2.63 (t, *J* = 7.7 Hz, 2H), 2.04–1.93 (m, 2H); ^13^C NMR (101 MHz, CDCl_3_) δ 155.7, 142.5, 138.0, 137.3, 135.1, 133.0, 131.1, 130.4, 126.7, 126.7, 126.0, 125.9, 125.4, 124.2, 120.7, 55.6, 55.6, 32.8, 32.3, 31.8; *m*/*z* LRMS (ESI^+^) 312 [M(^37^Cl)+H]^+^ 314 [M(^35^Cl)+H]^+^; HRMS (ESI^+^) C_19_H_18_^35^ClNO [M+H]^+^ calcd 312.1150, found 312.1145.

#### 3-(3-(4-Chloronaphthalen-1-yl)propyl)-5-methoxypyridine 1-oxide (**65**)

Following general procedure B, **65** was obtained from **65a** (30 mg, 0.096 mmol). Purification by flash column chromatography (5% MeOH in CH_2_Cl_2_) afforded the title compound as a transparent oil (27 mg, 86%): ^1^H NMR (400 MHz, CDCl_3_) δ 8.30–8.25 (m, 1H), 7.94–7.86 (m, 1H), 7.81 (t, *J* = 1.9 Hz, 1H), 7.76 (s, 1H), 7.54 (tt, *J* = 6.8, 5.2 Hz, 2H), 7.44 (d, *J* = 7.6 Hz, 1H), 7.15 (d, *J* = 7.6 Hz, 1H), 6.63 (t, *J* = 1.7 Hz, 1H), 3.75 (s, 3H), 3.03 (t, *J* = 7.6 Hz, 2H), 2.58 (t, *J* = 7.7 Hz, 2H), 2.06–1.94 (m, 2H); ^13^C NMR (101 MHz, CDCl_3_) δ 157.6, 140.5, 136.6, 132.7, 132.3, 131.0, 130.5, 126.7, 126.7, 126.0, 125.7, 125.4, 125.3, 123.9, 113.5, 56.1, 32.6, 32.0, 30.9; *m*/*z* LRMS (ESI^+^) 330 [M(^37^Cl)+H]^+^ 328 [M(^35^Cl)+H]^+^; HRMS (ESI^+^) C_19_H_18_^35^ClNO_2_ [M+H]^+^ calcd 328.1099, found 328.1092; HPLC 97% (AUC), *t*_R_ = 7.5 min.

#### 5-(3-(4-Chloronaphthalen-1-yl)propyl)pyridin-3-ol (**66a**)

BBr_3_ (1 M in CH_2_Cl_2_, 1.45 mL, 1.45 mmol) was added to a solution of **65a** (150 mg, 0.482 mmol) in CH_2_Cl_2_ (10 mL) at −78 °C. The resulting mixture was warmed to rt and stirred for 16 h. After completion, the reaction was quenched with MeOH (5 mL) at 0 °C and concentrated *in vacuo*. The crude product was purified by flash column chromatography (4% MeOH in CH_2_Cl_2_) to afford title product **66a** as a yellow oil (43 mg, 30%): ^1^H NMR (400 MHz, CDCl_3_) δ 8.83–8.71 (m, 1H), 8.34–8.25 (m, 1H), 7.76 (d, *J* = 7.8 Hz, 1H), 7.65–7.58 (m, 2H), 7.52 (d, *J* = 7.8 Hz, 1H), 2.69 (s, 3H); ^13^C NMR (101 MHz, CDCl_3_) δ 155.4, 139.7, 139.2, 137.2, 134.0, 133.0, 131.1, 130.5, 126.8, 126.8, 126.0, 125.9, 125.4, 125.3, 124.1, 32.7, 32.3, 31.6; *m*/*z* LRMS (ESI^+^) 296 [M(^37^Cl)–H]^−^ 298 [M(^35^Cl)–H]^−^; HRMS (ESI^+^) C_18_H_16_ClNO [M+H]^+^ calcd 296.0848, found 296.0845.

#### 3-(3-(4-Chloronaphthalen-1-yl)propyl)-5-hydroxypyridine 1-oxide (**66**)

Following general procedure B′, **66** was obtained from **66a** (20 mg, 0.067 mmol). Purification by flash column chromatography (7% MeOH in CH_2_Cl_2_) afforded the title compound as a yellow oil (9.0 mg, 43%): ^1^H NMR (400 MHz, MeOD) δ 8.30–8.20 (m, 1H), 8.05 (ddt, *J* = 7.6, 5.0, 2.2 Hz, 1H), 7.77 (dt, *J* = 6.4, 1.8 Hz, 2H), 7.64–7.54 (m, 2H), 7.49 (d, *J* = 7.6 Hz, 1H), 7.27 (d, *J* = 7.6 Hz, 1H), 6.94 (s, 1H), 3.12–3.06 (m, 2H), 2.72–2.62 (m, 2H), 2.02 (tt, *J* = 9.5, 6.7 Hz, 2H); ^13^C NMR (101 MHz, MeOD) δ 157.8, 143.5, 138.6, 134.2, 132.2, 131.9, 131.2, 127.9, 127.8, 127.3, 127.0, 126.9, 125.9, 125.3, 119.4, 33.3, 33.0, 32.5; *m*/*z* LRMS (ESI^+^) 312 [M(^37^Cl)–H]^−^ 314 [M(^35^Cl)–H]^−^; HRMS (ESI^+^) C_18_H_16_ClNO [M+H]^+^ calcd 312.0797, found 312.0794; HPLC 98% (AUC), *t*_R_ = 5.2 min.

#### 3-(2-((4-Chloronaphthalen-1-yl)oxy)ethyl)-5-fluoropyridine (**67a**)

Following general procedure A, **67a** was obtained from 2-(5-fluoropyridin-3-yl)ethan-1-ol (200 mg, 1.42 mmol) and 4-chloronaphthalen-1-ol (304 mg, 1.70 mmol). Purification by flash column chromatography (10% EtOAc in CH_2_Cl_2_) afforded the title compound as a white solid (280 mg, 66%): ^1^H NMR (400 MHz, CDCl_3_) δ 8.47 (t, *J* = 1.8 Hz, 1H), 8.38 (d, *J* = 2.8 Hz, 1H), 8.27–8.08 (m, 2H), 7.61 (ddd, *J* = 8.3, 6.9, 1.4 Hz, 1H), 7.53 (ddd, *J* = 8.3, 6.9, 1.3 Hz, 1H), 7.46–7.37 (m, 2H), 6.68 (d, *J* = 8.3 Hz, 1H), 4.33 (t, *J* = 6.2 Hz, 2H), 3.25 (t, *J* = 6.2 Hz, 2H); ^13^C NMR (101 MHz, CDCl_3_) δ 159.6 (d, *J* = 179 Hz), 153.3, 146.3 (d, *J* = 4.0 Hz), 136.6 (d, *J* = 23 Hz), 135.9 (d, *J* = 4.0 Hz), 131.4, 127.7, 126.6, 126.3, 125.7, 124.4, 123.8, 123.4 (d, *J* = 18 Hz), 122.3, 104.8, 68.0, 32.6; *m*/*z* LRMS (ESI^+^) 302 [M+H]^+^; HRMS (ESI^+^) C_17_H_13_^35^ClFNO [M+H]^+^ calcd 302.0739, found 302.0743.

#### 3-(2-((4-Chloronaphthalen-1-yl)oxy)ethyl)-5-fluoropyridine 1-oxide (**67**)

Following general procedure B, **67** was obtained from **67a** (100 mg, 0.332 mmol). Purification by flash column chromatography (5% MeOH in CH_2_Cl_2_) afforded the title compound as a white solid (93.8 mg, 89%): ^1^H NMR (400 MHz, CDCl_3_) δ 8.28–8.11 (m, 3H), 8.06 (dt, *J* = 4.0, 1.9 Hz, 1H), 7.62 (ddd, *J* = 8.4, 6.9, 1.4 Hz, 1H), 7.55 (ddd, *J* = 8.2, 6.9, 1.3 Hz, 1H), 7.43 (d, *J* = 8.2 Hz, 1H), 7.10 (ddd, *J* = 7.6, 2.1, 1.3 Hz, 1H), 6.69 (d, *J* = 8.2 Hz, 1H), 4.35 (t, *J* = 6.0 Hz, 2H), 3.19 (t, *J* = 6.0 Hz, 2H); ^13^C NMR (101 MHz, CDCl_3_) δ 160.4 (d, *J* = 252 Hz), 153.1, 138.3 (d, *J* = 9.0 Hz), 136.4, 131.5, 128.1 (d, *J* = 35.0 Hz), 127.8, 126.5, 126.4, 125.6, 124.5, 124.2, 122.1 114.7 (d, *J* = 20 Hz), 104.8, 67.1, 32.8; *m*/*z* LRMS (ESI^+^) 318 [M+H]^+^; HRMS (ESI^+^) C_17_H_13_ClFNO_2_ [M+H]^+^ calcd 318.0692, found 318.0686; HPLC 98% (AUC), *t*_R_ = 6.3 min.

#### (*E*)-1-(3,5-Bis(trifluoromethyl)phenyl)-3-(5-methoxypyridin-3-yl)prop-2-en-1-one (**68d**)

BF_3_·Et_2_O (5.0 mL, 40.5 mmol) was added to an ice-cold mixture of 5-methoxynicotinaldehyde (2.14 g, 15.6 mmol) and 1-(3,5-bis(trifluoromethyl)phenyl)ethan-1-one (2.00 g, 7.81 mmol) in 1,4-dioxane (15 mL), and the resulting mixture was stirred at 105 °C for 16 h. After completion, the reaction was cooled to rt before addition of NaHCO_3_ (aq. sat. sol., 80 mL), and the mixture was extracted with EtOAc (3 × 50 mL). The combined organic phase was washed with brine (100 mL), dried (Na_2_SO_4_), filtered, and concentrated *in vacuo*. Purification of the resulting residue by flash column chromatography (0–60% EtOAc in pentane) afforded title compound **68d** as a white solid (2.30 g, 78%): ^1^H NMR (400 MHz, CDCl_3_) δ 8.44 (d, *J* = 1.8 Hz, 1H), 8.41–8.34 (m, 2H), 8.33 (d, *J* = 2.8 Hz, 1H), 8.08–8.02 (m, 1H), 7.82 (d, *J* = 15.7 Hz, 1H), 7.47 (d, *J* = 15.7 Hz, 1H), 7.38 (dd, *J* = 2.9, 1.8 Hz, 1H), 3.88 (s, 3H); ^13^C NMR (101 MHz, CDCl_3_) δ 187.0, 156.0, 143.7, 143.7, 142.9, 140.2, 139.4, 132.6 (d, *J* = 34.0 Hz), 130.7, 128.6, 126.4, 124.4, 122.3, 121.7, 118.7, 118.7, 56.0; ^19^F NMR (376 MHz, CDCl_3_) δ −62.85; *m*/*z* LRMS (ESI^+^) 376 [M+H]^+^; HRMS (ESI^+^) C_17_H_11_F_6_NO_2_ [M+H]^+^ calcd 376.0761, found 376.0767.

#### (*E*)-3-(3-(3,5-Bis(trifluoromethyl)phenyl)prop-1-en-1-yl)-5-methoxypyridine (**68c**)

Triethylsilane (1.0 mL, 6.26 mmol) was added to an ice-cold solution of **68d** (300 mg, 0.80 mmol) in TFA (1 mL) and the resulting mixture stirred at rt for 48 h. After completion, the mixture was diluted with CH_2_Cl_2_ (20 mL), quenched with NaOH (1 M, aq., 20 mL), and extracted with CH_2_Cl_2_ (3 × 20 mL). The combined organic phase was washed with brine (50 mL), dried (Na_2_SO_4_), filtered, and concentrated *in vacuo*. Purification of the resulting residue by flash column chromatography (0–60% EtOAc in pentane) to afford title compound **68c** as a colorless oil (120 mg, 42%): ^1^H NMR (400 MHz, CDCl_3_) δ 8.43–8.25 (m, 2H), 7.79 (s, 1H), 7.67 (d, *J* = 1.6 Hz, 2H), 7.58 (dd, *J* = 2.5, 1.5 Hz, 1H), 6.62–6.44 (m, 2H), 3.96 (s, 3H), 3.75 (d, *J* = 6.3 Hz, 2H); ^13^C (101 MHz, CDCl_3_) δ 157.7, 141.0, 136.41, 134.4, 134.0, 132.2 (d, *J* = 33.0 Hz), 131.7, 129.1, 129.0, 126.7, 126.7, 124.7, 124.1, 122.0, 121.1 (q, *J* = 3.0 Hz), 56.7, 39.0; ^19^F NMR (376 MHz, CDCl_3_) δ −62.85; *m*/*z* LRMS (ESI^+^) 362 [M+H]^+^; HRMS (ESI^+^) C_17_H_13_F_6_NO [M+H]^+^ calcd 362.0974, found 362.0965.

#### 3-(3-(3,5-Bis(trifluoromethyl)phenyl)propyl)-5-methoxypyridine (**68b**)

Following general procedure D, **68b** was obtained from **68c** (390 mg, 1.08 mmol). Purification by flash column chromatography (0–40% EtOAc in pentane) afforded the title compound as a white solid (305 mg, 78%): ^1^H NMR (400 MHz, CDCl_3_) δ 8.16 (d, *J* = 2.8 Hz, 1H), 8.07 (d, *J* = 1.8 Hz, 1H), 7.72 (s, 1H), 7.61 (d, *J* = 1.6 Hz, 2H), 7.00 (dd, *J* = 2.7, 1.8 Hz, 1H), 3.85 (s, 3H), 2.83–2.74 (m, 2H), 2.67 (t, *J* = 7.6 Hz, 2H), 2.01 (tdd, *J* = 9.5, 6.9, 4.3 Hz, 2H); ^13^C NMR (101 MHz, CDCl_3_) δ 155.9, 144.2, 142.3, 142.3, 137.4, 135.2, 131.9 (d, *J* = 30 Hz), 128.6, 124.9, 122.2, 120.9, 120.9, 120.3, 55.7, 35.1, 32.4, 32.2; ^19^F NMR (376 MHz, CDCl_3_) δ −62.85; *m*/*z* LRMS (ESI^+^) 364 [M+H]^+^; HRMS (ESI^+^) C_17_H_15_ClF_6_NO [M+H]^+^ calcd 364.1131, found 364.1124.

#### 5-(3-(3,5-Bis(trifluoromethyl)phenyl)propyl)pyridin-3-ol (**68a**)

48% HBr in water (4 mL) was added to a solution of **68b** (140 mg, 0.386 mmol) and the resulting mixture stirred at 120 °C for 48 h. After completion, the reaction was cooled to rt, neutralized with NaHCO_3_ (aq. sat. sol.), and extracted with EtOAc (3 × 30 mL). The combined organic phase was washed with brine (100 mL), dried (Na_2_SO_4_), filtered, and concentrated *in vacuo*. Purification of the resulting residue by flash column chromatography (0–10% MeOH in CH_2_Cl_2_) afforded 5-(3-(3,5-bis(trifluoromethyl)phenyl)propyl)pyridin-3-ol **68a** as a white solid (110 mg, 82%): ^1^H NMR (400 MHz, CDCl_3_) δ 8.16 (d, *J* = 2.6 Hz, 1H), 7.92 (d, *J* = 1.8 Hz, 1H), 7.72 (s, 1H), 7.61 (s, 2H), 7.17 (t, *J* = 2.2 Hz, 1H), 2.79 (dd, *J* = 9.0, 6.7 Hz, 2H), 2.67 (t, *J* = 7.6 Hz, 2H), 2.07–1.95 (m, 2H); ^13^C NMR (101 MHz, CDCl_3_) δ 155.4, 144.1, 144.1, 139.3, 138.9, 138.9, 134.0, 131.9 (q, *J* = 30 Hz), 128.7, 125.4, 124.9, 122.2, 120.4, 35.1, 32.4, 32.1; ^19^F NMR (376 MHz, CDCl_3_) δ −62.87; *m*/*z* LRMS (ESI^+^) 350 [M+H]^+^; HRMS (ESI^+^) C_16_H_13_F_6_NO [M+H]^+^ calcd 350.0969, found 350.0974.

#### 3-(3-(3,5-Bis(trifluoromethyl)phenyl)propyl)-5-hydroxypyridine 1-oxide (**68**)

Following general procedure B, **68** was obtained from **68a** (130 mg, 0.372 mmol). Purification by flash column chromatography (0–6% MeOH in CH_2_Cl_2_) afforded the title compound as a white solid (95 mg, 70%): ^1^H NMR (400 MHz, CDCl_3_) δ 8.03 (t, *J* = 1.9 Hz, 1H), 7.72 (s, 1H), 7.69–7.54 (m, 3H), 6.94 (t, *J* = 1.7 Hz, 1H), 2.77 (t, *J* = 8.0 Hz, 2H), 2.60 (t, *J* = 7.7 Hz, 2H), 2.06–1.89 (m, 2H); ^13^C NMR (101 MHz, CDCl_3_) δ 157.7, 143.6, 141.0, 132.0 (q, *J* = 33.1 Hz), 129.8, 128.6 (q, *J* = 3.8 Hz), 126.9, 126.2, 124.4, 122.6, 120.8, 120.51 (p, *J* = 3.9 Hz), 119.9, 35.0, 32.4, 31.6; ^19^F NMR (376 MHz, CDCl_3_) δ −62.86; *m*/*z* LRMS (ESI^+^) 366 [M+H]^+^; HRMS (ESI^+^) C_16_H_13_F_6_NO_2_ [M+H]^+^ calcd 366.0919, found 366.0923; HPLC 98% (AUC), *t*_R_ = 4.9 min.

#### (*E*)-1-(4-Chloro-3-(trifluoromethyl)phenyl)-3-(5-methoxypyridin-3-yl)prop-2-en-1-one (**69d**)

BF_3_·Et_2_O (2.7 mL, 21.9 mmol) was added to an ice-cold mixture of 5-methoxynicotinaldehyde (923 mg, 6.74 mmol) and 1-(4-chloro-3-(trifluoromethyl)phenyl)ethan-1-one (1.00 g, 4.49 mmol) in 1,4-dioxane (10 mL), and the resulting mixture was stirred at 105 °C for 16 h. After completion, the reaction was cooled to rt before addition of NaHCO_3_ (aq. sat. sol., 80 mL), and the mixture was extracted with EtOAc (3 × 30 mL). The combined organic phase was washed with brine (100 mL), dried (Na_2_SO_4_), filtered, and concentrated *in vacuo*. Purification of the resulting residue by flash column chromatography (0–60% EtOAc in pentane) afforded title compound **69d** as a white solid (1.22 g, 80%): ^1^H NMR (400 MHz, acetone-*d*^6^) δ 8.58 (d, *J* = 1.8 Hz, 1H), 8.48 (d, *J* = 2.2 Hz, 1H), 8.44 (ddd, *J* = 8.3, 2.2, 0.7 Hz, 1H), 8.34 (d, *J* = 2.8 Hz, 1H), 8.09 (d, *J* = 15.7 Hz, 1H), 7.91–7.84 (m, 3H), 3.95 (s, 3H); ^13^C NMR (101 MHz, acetone-*d*_6_) δ 187.7, 156.9, 143.9, 142.9, 140.9, 137.7, 137.0, 134.5, 133.2, 132.0, 128.6 (q, *J* = 10 Hz), 125.1, 124.1, 122.4, 118.8, 56.2; ^19^F NMR (376 MHz, acetone-*d*_6_) δ −63.16, −63.17; *m*/*z* LRMS (ESI^+^) 342 [M+H]^+^; HRMS (ESI^+^) C_16_H_11_^35^ClF_3_NO_2_ [M+H]^+^ calcd 342.0503, found 342.0497.

#### (*E*)-3-(3-(4-Chloro-3-(trifluoromethyl)phenyl)prop-1-en-1-yl)-5-methoxypyridine (**69c**)

Triethylsilane (3.7 mL, 23.2 mmol) was added to an ice-cold solution of **69d** (1.00 g, 2.93 mmol) in TFA (3.6 mL) and the resulting mixture stirred at rt for 48 h. After completion, the mixture was diluted with CH_2_Cl_2_ (50 mL), quenched with NaOH (1M, aq., 30 mL), and extracted with CH_2_Cl_2_ (3 × 30 mL). The combined organic phase was washed with brine (50 mL), dried (Na_2_SO_4_), filtered, and concentrated *in vacuo*. Purification of the resulting residue by flash column chromatography (0–60% EtOAc in pentane) to afford title compound **69c** as a colorless oil (670 mg, 70%): ^1^H NMR (400 MHz, CDCl_3)_ δ 8.30 (d, *J* = 1.7 Hz, 1H), 8.22 (d, *J* = 2.7 Hz, 1H), 7.51 (d, *J* = 2.1 Hz, 1H), 7.49–7.41 (m, 2H), 7.32 (dd, *J* = 8.3, 2.2 Hz, 1H), 6.56–6.37 (m, 2H), 3.91 (s, 3H), 3.61 (d, *J* = 6.3 Hz, 2H); ^13^C NMR (101 MHz, CDCl_3_) δ 157.2, 137.8, 135.9, 135.5, 133.8, 133.3, 131.8, 130.7, 127.8 (q, *J* = 10 Hz), 126.9, 126.6, 124.2, 122.3, 121.5, 56.3, 38.5; ^19^F NMR (376 MHz, CDCl_3_) δ −62.59, −75.64; *m*/*z* LRMS (ESI^+^) 328 [M+H]^+^; HRMS (ESI^+^) C_16_H_13_^35^ClF_3_NO [M+H]^+^ calcd 328.0704, found 328.0711.

#### 3-(3-(4-Chloro-3-(trifluoromethyl)phenyl)propyl)-5-methoxypyridine (**69b**)

Following general procedure D′, **69b** was obtained from **69c** (640 mg, 1.96 mmol). Purification by flash column chromatography (0–40% EtOAc in pentane) afforded the title compound as a white solid (550 mg, 85%): ^1^H NMR (400 MHz, CDCl_3_) δ 8.19 (d, *J* = 29.5 Hz, 2H), 7.48 (d, *J* = 2.2 Hz, 1H), 7.42 (d, *J* = 8.2 Hz, 1H), 7.30–7.26 (m, 1H), 7.19 (s, 1H), 3.89 (s, 3H), 2.70 (t, *J* = 7.4 Hz, 4H), 2.04–1.89 (m, 2H); ^19^F NMR (376 MHz, CDCl_3_) δ −62.6, −75.7; *m*/*z* LRMS (ESI^+^) 330 [M+H]^+^; HRMS (ESI^+^) C_16_H_15_^35^ClF_3_NO [M+H]^+^ calcd 330.0862, found 330.0867.

#### 5-(3-(4-Chloro-3-(trifluoromethyl)phenyl)propyl)pyridin-3-ol (**69a**)

48% HBr in water (10 mL) was added to a solution of **69b** (400 mg, 1.21 mmol) and the resulting mixture stirred at 120 °C for 48 h. After completion, the reaction was cooled to rt, neutralized with NaHCO_3_ (aq. sat. sol.), and extracted with EtOAc (3 × 30 mL). The combined organic phase was washed with brine (100 mL), dried (Na_2_SO_4_), filtered, and concentrated *in vacuo*. Purification of the resulting residue by flash column chromatography (0–10% MeOH in CH_2_Cl_2_) afforded 5-(3-(4-chloro-3-(trifluoromethyl) phenyl)propyl)pyridin-3-ol 69a as a white solid (294 mg, 77%): ^1^H NMR (400 MHz, CDCl_3_) δ 8.12 (d, *J* = 2.7 Hz, 1H), 7.91 (d, *J* = 1.8 Hz, 1H), 7.47 (d, *J* = 2.2 Hz, 1H), 7.40 (d, *J* = 8.2 Hz, 1H), 7.26 (dd, *J* = 8.2, 2.2 Hz, 1H), 7.14 (t, *J* = 2.2 Hz, 1H), 2.65 (dt, *J* = 18.0, 7.7 Hz, 4H), 2.00–1.90 (m, 2H); ^13^C NMR (101 MHz, CDCl_3_) δ 155.4, 140.8, 139.4 (d, *J* = 4 Hz), 134.1, 132.9, 131.6, 129.9, 128.4 (d, *J* = 31 Hz), 127.5 (d, *J* = 6 Hz), 125.2, 124.4, 121.6, 34.6, 32.3, 32.2; ^19^F NMR (376 MHz, CDCl_3_) δ −62.54; *m*/*z* LRMS (ESI^+^) 316 [M+H]^+^; HRMS (ESI^+^) C_15_H_13_^35^ClF_3_NO [M+H]^+^ calcd 316.0711, found 316.0706.

#### 3-(3-(4-Chloro-3-(trifluoromethyl)phenyl)propyl)-5-hydroxypyridine 1-oxide (**69**)

Following general procedure B, **69** was obtained from **69a** (200 mg, 0.633 mmol). Purification by flash column chromatography (0–6% MeOH in CH_2_Cl_2_) afforded the title compound as a white solid (152 mg, 73%): ^1^H NMR (400 MHz, CDCl_3_) δ 8.01 (t, *J* = 1.9 Hz, 1H), 7.62 (d, *J* = 1.6 Hz, 1H), 7.46 (d, *J* = 2.1 Hz, 1H), 7.40 (d, *J* = 8.2 Hz, 1H), 7.29–7.21 (m, 1H), 6.92 (t, *J* = 1.6 Hz, 1H), 2.70–2.60 (m, 2H), 2.55 (t, *J* = 7.7 Hz, 2H), 1.97–1.85 (m, 2H); ^13^C NMR (101 MHz, CDCl_3_) δ 157.6, 140.7 (d, *J* = 80 Hz), 132.9, 131.7, 130.1, 129.7, 128.5 (d, *J* = 30 Hz), 127.5 (q, *J* = 10 Hz), 126.8, 124.3, 121.6, 119.7, 34.5, 32.3, 31.6; ^19^F NMR (377 MHz, CDCl_3_) δ −62.55. *m*/*z* LRMS (ESI^+^) 332 [M+H]^+^; HRMS (ESI^+^) C_15_H_13_^35^ClF_3_NO_2_ [M+H]^+^ calcd 332.0660, found 332.0651; HPLC 98% (AUC), *t*_R_ = 4.2 min.

### Calculation of LLE

LLE is the difference between pEC_50_ and cLogP (eq 1), used to estimate the specificity of the molecule binding to the protein-of-interest compared to partitioning into 1-octanol.^40^
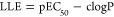
1

### Cell-Based cAMP Assay

CHO-K1 cells or CHO-K1 cells stably expressing the human GPR84 receptor (DiscoverX 95-0158C2) were plated into a 384-well plate at 15,000 cells/20 μL/well in corresponding media (Table S3) and incubated overnight at 37 °C/5% CO_2_. Cells were then stimulated with forskolin (25 μM) and agonist for 30 min. Ligands were dissolved in DMSO and prepared in Dulbecco’s phosphate-buffered saline (DPBS) + 0.1% bovine serum albumin (BSA). Cell lysis and detection of cAMP were performed using the HitHunter cAMP Assay for Small Molecules (DiscoverX 90-0075SM2 as per the manufacturer’s instructions). Luminescence was measured 18–24 h after the final step on a PHERAstar FS microplate reader (BMG Labtech). EC_50_ values were calculated in GraphPad Prism (v9.5.0) using a four-parameter dose–response model. Curves were normalized to forskolin (25 μM) and the maximum effect of capric acid (100 μM) or vehicle for CHO-K1 cells counterscreening.

### Cell-Based β-Arrestin Recruitment Assay

CHO-K1 cells stably expressing prolink tagged human GPR84 and enzyme acceptor tagged β-arrestin (DiscoverX 93-0647C2) were seeded in a 384-well plate at 5000 cells/20 μL/well in corresponding media (Table S3) and incubated overnight at 37 °C/5% CO_2_. Cells were then stimulated with agonist for 90 min. Ligands were dissolved in DMSO and prepared at 5× in DPBS + 0.1% BSA + 0.125% Tween-80 to prevent compound aggregation at high concentrations. All compounds were observed to be soluble at the highest concentrations tested. Cell lysis and detection of β-arrestin were performed using the PathHunter cAMP Assay for Small Molecules (DiscoverX 93-0001 as per the manufacturer’s instructions). Luminescence was measured 1 h after the final step on a PHERAstar FS microplate reader (BMG Labtech). EC_50_ values were calculated in GraphPad Prism (v9.5.0) using a four-parameter dose–response model.

### Non-radioactive Cytotoxicity Assay

CHO-K1 cells or CHO-K1 cells stably expressing the human GPR84 receptor (DiscoverX 95-0158C2) were plated into a 384-well plate at 15,000 cells/20 μL/well in corresponding media (Table S3). Ligands were dissolved in DMSO and prepared in DPBS + 0.1% BSA. Cells were then incubated with ligands for 20 h. Detection of cell viability was performed using the CytoTox 96 LDH Cytotoxicity Assay (Promega G1780). The absorbance signal was measured at 490 nm in a SPECTROstar Omega microplate reader. Data were first baseline subtracted to media-only conditions and then normalized to vehicle (0%) and maximum cell lysis (100%).

### Mouse Liver Microsome Stability

MLM studies were performed by either Wuxi AppTec Ltd. (Nanjing) or Cyprotex. Compounds (1 μM) were incubated at 37 °C for 60 min in a 0.5 mg protein/mL liver microsome in 100 mM potassium phosphate buffer. At the end of the designated time point, 5, 15, 30, 45, and 60 min samples were quenched with acetonitrile spiked with 200 ng/mL tolbutamide and 200 ng/mL labetalol (internal standard). Samples were shaken for 10 min before centrifuging, and supernatants were analyzed by LC-MS/MS.

### Human GPR40/FFA1, GPR120/FFA4, and CB2 FLIPR Assays

Selectivity assays were performed by Wuxi AppTec Ltd. (Shanghai). CHO cells expressing human GPR40, GPR120, and CB2 were cultured in corresponding media (Table S3). Cells were dye-loaded with Fluo-4 for 50 min at 37 °C/5% CO_2_ then 10 min at rt. Cells were then stimulated with agonist, and the calcium response was then measured over time. The data was analyzed in GraphPad Prism (v9.5.0).

### *In Vivo* Pharmacokinetic Studies

Mouse pharmacokinetic studies were performed by Wuxi AppTec Ltd. All mouse studies were conducted in accordance with the local Ethics Review process. Male C57BL/6J mice (*n* = 3) were orally administered with a dose of 10 mg/kg of the selected compounds (formulation 1 mg/mL in 30% propylene glycol:10% Chermophor EL:20% Solutol:40% water). Blood was taken at predose and 0.25, 0.5, 1, 2, 4, 8, and 24 h after dosing. Plasma concentrations were determined by LC-MS/MS. The plasma concentrations were simulated by using a PO-Noncompartmental model 200 instrument from the plasma concentrations obtained in the PK study using Phoenix WinNonlin 8.3.5.

## Supporting Information Available

Proposed major metabolites of **11** (DL-175) following incubation with mouse hepatocytes (Table S1); detailed EC_50_ and pEC_50_ ± SEM values for GPR84 agonists in cAMP and β-arrestin assays (Table S2); incubation media for CHO-hGPR84 (cAMP), CHO-hGPR84 (β-arrestin), CHO-hFFA1, CHO-hFFA4, CHO-hCB2, and CHO-K1 cells (Table S3); ^1^H, ^13^C, and ^19^F NMR spectra of representative intermediate and final products; HPLC traces and HRMS spectra of representative compounds (PDF)
Molecular formula strings (CSV)

## Abbreviations Used

ADME: absorption, distribution, metabolism, and excretion
APMAP: adipocyte plasma membrane-associated protein
AUC: area under curve
BSA: bovine serum albumin
cAMP: cyclic adenosine monophosphate
CB2: cannabinoid receptor 2
CHO: Chinese hamster ovary
*C*_max_: peak drug concentration
δ: chemical shift in parts per million downfield from tetramethylsilane
DAST: diethylaminosulfur trifluoride
DBU: 1,8-diazabicyclo[5.4.0]undec-7-ene
DIAD: diisopropyl azodicarboxylate
DMF: *N,N*-dimethylformamide
DPBS: Dulbecco’s phosphate-buffered saline
FFA: free fatty acid
FFAR: free fatty acid receptor
FLIPR: fluorometric image plate reader
FSK: Forskolin
GPR84: G-protein-coupled receptor 84
HBA: hydrogen bond acceptor
HBD: hydrogen bond donor
HPLC: high-performance liquid chromatography
HRMS: high-resolution mass spectra
LC-MS/MS: liquid chromatography–tandem mass spectrometry
LDH: lactate dehydrogenase
LLE: lipophilic ligand efficiency
LPS: lipopolysaccharide
MCFA: medium-chain fatty acid
*m*-CPBA: *meta*-chloroperoxybenzoic acid
MLM: mouse liver microsome
MRT: mean residence time
PK: pharmacokinetics
ppm: parts per million
QSAR: quantitative structure–activity relationship
SAR: structure–activity relationship
SD: standard deviation
SEM: standard error of the mean
TFA: trifluoroacetic acid
THF: tetrahydrofuran
*T*_max_: peak time
TNFα: tumor necrosis factor alpha
