# Supplementary material for: Development of Highly Potent, G-Protein Pathway Biased, Selective, and Orally Bioavailable GPR84 Agonists
Source: J Med Chem. 2023 Dec 26;67(1):110–37. doi: 10.1021/acs.jmedchem.3c00951 (PMC10788923; doi:10.1021/acs.jmedchem.3c00951)

SUPPORTING INFORMATION

Development of Highly Potent, G-Protein Pathway Biased, Selective and Orally Bioavailable GPR84 Agonists

Pinqi Wang[1,2], Arun Raja[1,2], Vincent B. Luscombe[3], Carole J. R. Bataille[1,2], Daniel Lucy[1,3], Vanessa V. Rogga[1], David R. Greaves[3], Angela J. Russell*[,1,2]

[1]Department of Chemistry, University of Oxford, Mansfield Road Oxford OX1 3TA, U.K.

[2]Department of Pharmacology, University of Oxford, Mansfield Road, Oxford OX1 3QT, U.K.

[3]Sir William Dunn School of Pathology, University of Oxford, South Parks Road, Oxford OX1 3RE, U.K.

*To whom correspondence should be addressed. Email: angela.russell@chem.ox.ac.uk.

Contents

Supplementary Tables......S3

Table S1. Proposed major metabolites of 11 (DL-175) following incubation with mouse hepatocytes......S3

Table S2. Detailed information of compounds......S3

Table S3. Media components for hGPR84 (cAMP), hGPR84 (*β*-arrestin), hFFA1, hFFA4 and hCB2 CHO cells......S5

Supplementary Figures......S5

Figure S1. In vitro cytotoxicity......S5

NMR spectra......S6

HPLC traces for final products......S148

HRMS spectra for final products......S205

Supplementary Tables

Table S1. Proposed major metabolites of 11 (DL-175) following incubation with mouse hepatocytes

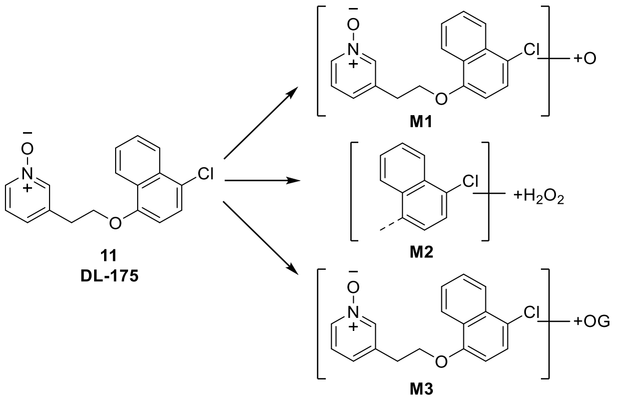

metabolite	m/z	proposed pathway	% peak area
M1	316	monooxidation	68
M2	334	dihydroxylation	12
M3	492	monooxidation; *O*-glucuronidation	8

Table S2. Detailed information of compounds.

cAMP assays measure the inhibition of 25 µM FSK-induced cAMP production in CHO-hGPR84 cells. Efficacy is calculated from capric acid E_{max} (If inactive, the efficacy at 30 uM is presented). Data are mean ± SEM. Recruitment of *β*-arrestin in CHO-*β*-arrestin-hGPR84 cells, inactive means no detectable effect at 80 µM. ClogP values were calculated by DataWarrior (5.5.0).

cmpd	pEC_{50}	SEM	cAMP EC_{50} (nM)	E% C10	n	SEM	*β*-arrestin EC_{50} (nM)	E% 6-OAU (80 µM)	n	ClogP	LLE
3	8.20	0.12	6.28	99%	11	0.01	12520	100%	3		
7	9.82	0.34	0.151	94%	3	0.02	3707	180%	3		
9	8.97	0.16	1.07	98%	3	0.02	>20000	82%	3		
11	7.91	0.04	12.4	99%	34	0.00	>80000	13%	3	3.76	4.10
12	7.55	0.14	28.4	95%	3	0.03	>80000	-1%	1	3.88	3.47
13	7.20	0.06	62.6	103%	3	0.04	>80000	12%	1	3.26	4.06
14	6.59	0.13	256	99%	3	0.01	>80000	-2%	1	3.09	3.46
15	6.60	0.04	254	98%	4	0.01	>80000	3%	1	3.16	3.36
16	6.71	0.08	195	98%	4	0.01	>80000	7%	1	3.72	2.93
17	5.83	0.02	1470	101%	3	0.02	>80000	-1%	1	2.89	2.96
18	5.51	0.19	3080	100%	3	0.02	>80000	5%	1	2.55	2.97
19	5.87	0.10	1360	97%	3	0.01	>80000	4%	1	2.57	3.21
20	5.88	0.14	1310	98%	3	0.02	>80000	12%	1	2.69	3.13
21	6.01	0.09	983	100%	3	0.00	>80000	0%	1	2.40	3.62

22			>10000	41%	2		>80000	-12%	1	1.80	
23			>10000	35%	2		>80000	2%	1	1.04	
24			>10000	58%	2		>80000	8%	1	3.26	
25			>10000	61%	2		>80000	10%	1	3.17	
26			>10000	47%	2		>80000	8%	1	3.00	
27			>10000	10%	1					4.70	
28			>10000	8%	1					4.23	
29	5.49	0.24	3260	100%	3	0.02	>80000	0%	1	3.78	1.71
30	6.61	0.14	248	92%	3	0.05	>80000	5%	1	3.26	3.09
31	6.40	0.23	400	100%	3	0.03	>80000	1%	1	2.99	3.42
32	7.08	0.22	82.8	98%	3	0.01	>80000	0%	1	3.00	4.00
33	6.44	0.12	364	96%	3	0.03	>80000	0%	1	3.29	3.02
34	6.05	0.06	893	98%	3	0.01	>80000	1%	1	3.17	2.83
35	5.56	0.27	2760	95%	3	0.03	>80000	-5%	1	2.67	2.74
36			>10000	4%	1					4.23	
37	5.63	0.20	2340	91%	3	0.07	>80000	-2%	1	2.91	2.48
38			>10000	54%	2		>80000	13%	1	1.65	
39	6.22	0.15	606	102%	3	0.05	>80000	-4%	1	3.33	2.96
40	5.97	0.29	1080	98%	3	0.00	>80000	-14%	1	2.50	3.39
41	7.00	0.14	99.4	99%	3	0.01	>80000	-2%	1	3.68	3.29
42	6.05	0.08	898	102%	3	0.05	>80000	0%	1	3.42	2.68
43	6.11	0.08	776	92%	3	0.05	>80000	2%	1	3.66	2.27
44	6.30	0.08	497	99%	3	0.01	>80000	-6%	1	3.33	2.93
45	6.43	0.12	370	99%	3	0.01	>80000	15%	1	4.22	2.19
46	6.59	0.05	257	98%	3	0.01	>80000	0%	1	4.03	2.51
47	5.28	0.02	5300	94%	3	0.05	>80000	-12%	1	3.15	2.00
48	5.66	0.10	2180	93%	3	0.06	>80000	4%	1	2.57	2.86
49	6.49	0.13	325	94%	3	0.05	>80000	3%	1	3.45	2.85
50	6.18	0.12	665	94%	3	0.05	>80000	-3%	1	2.56	3.39
51			>10000	14%	1		>80000	1%	1	1.73	
52	5.38	0.14	4160	100%	3	0.01	>80000	9%	1	3.08	2.30
53			>10000	15%	1					2.37	
54	5.33	0.04	4680	93%	3	0.06	>80000	2%	1	2.56	2.58
55	6.23	0.22	591	103%	3	0.04	>80000	18%	1	2.28	4.07
56			>10000	15%	1		>80000	11%	1	4.57	
57			>10000	31%	1		>80000	8%	1	4.52	
58	7.89	0.15	12.9	101%	3	0.01	>80000	0%	1	3.58	4.35
59	6.65	0.15	222	99%	3	0.01	>80000	-1%	1	3.35	3.26
60	5.83	0.02	1470	99%	3	0.01	>80000	-1%	1	2.38	3.42
61	7.12	0.12	76.7	95%	3	0.03	>80000	3%	1	4.16	2.81
62			>10000	36%	1		>80000	-16%	1	4.58	
63	5.41	0.13	3920	101%	3	0.07	>80000	2%	1	4.56	0.85
64	8.07	0.30	8.6	100%	3	0.01	>80000	7%	1	4.64	3.42
65	7.25	0.12	56.4	99%	3	0.01	>80000	18%	1	4.57	2.66
66	11.50	0.20	0.0032	97%	4	0.01	>80000	14%	3	4.29	7.01
67	7.75	0.20	17.9	97%	3	0.02	>80000	3%	1	3.86	3.77
68	11.22	0.16	0.00598	97%	3	0.00	>80000	12%	3	4.19	6.86
69	10.73	0.18	0.0185	95%	3	0.01	>80000	16%	3	3.95	6.46

Table S3. Media components for hGPR84 (cAMP), hGPR84 (*β*-arrestin), hFFA1, hFFA4 and hCB2 CHO cells.

hGPR84-CHO (cAMP)	F12 + 10% FBS + 19 mM HEPES + 600µg/mL G418 + 1% PS
hGPR84-CHO (*β*-arrestin)	F12 +10% FBS +19 mM HEPES + 600 µg/mL G418 + 1% PS + 300 µg/mL Hygromycin B
hCB2-CHO	F12 +10% FBS + 300 µg/mL G418 + 2 µg/mL BS + 1% PS
hFFA1-CHO	F12 + 10% FBS + 300 µg/mL G418 + 1% PS
hFFA4-CHO	DMEM + F12 + 15 mM HEPES + 10% FBS + 1% PS + 200 µg/mL G418
CHO-K1	F12 + 10% FBS + 19 mM HEPES + 1% PS
Assay media	F12 + 10% FBS + 19 mM HEPES + 1% PS

Supplementary Figures

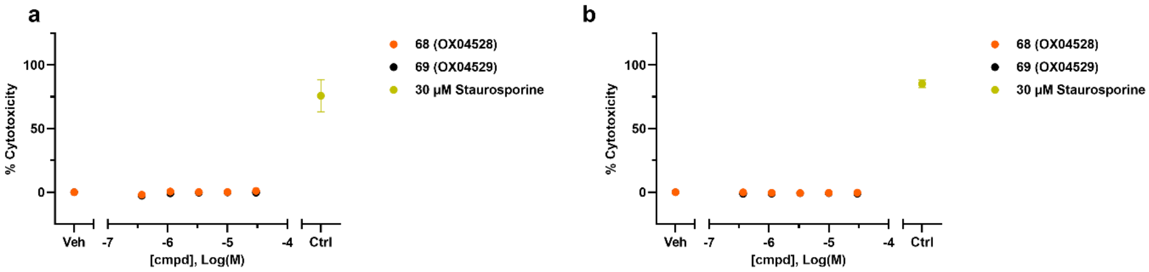

Figure S1. In vitro cytotoxicity. 68 and **69** show no cytotoxicity on (a) CHO-hGPR84 cells or (b) CHO-K1 cells after 20 h incubation (Data plotted from n = 3 independent experiments and shown as mean ± SEM).

NMR spectra

3-(2-((4-bromonaphthalen-1-yl)oxy)ethyl)pyridine (12a)

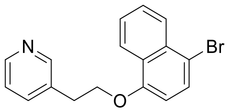

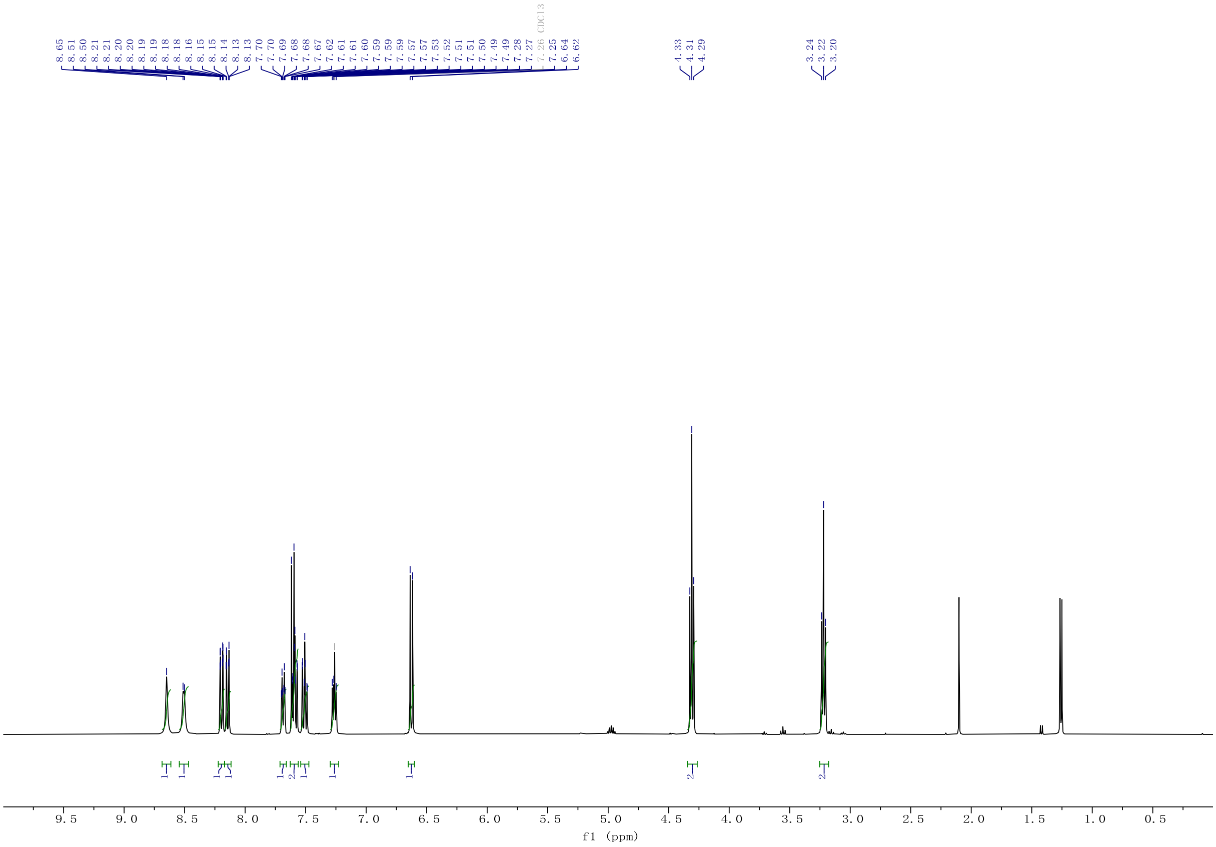

3-(2-((4-bromonaphthalen-1-yl)oxy)ethyl)pyridine 1-oxide (12)

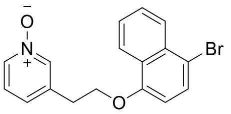

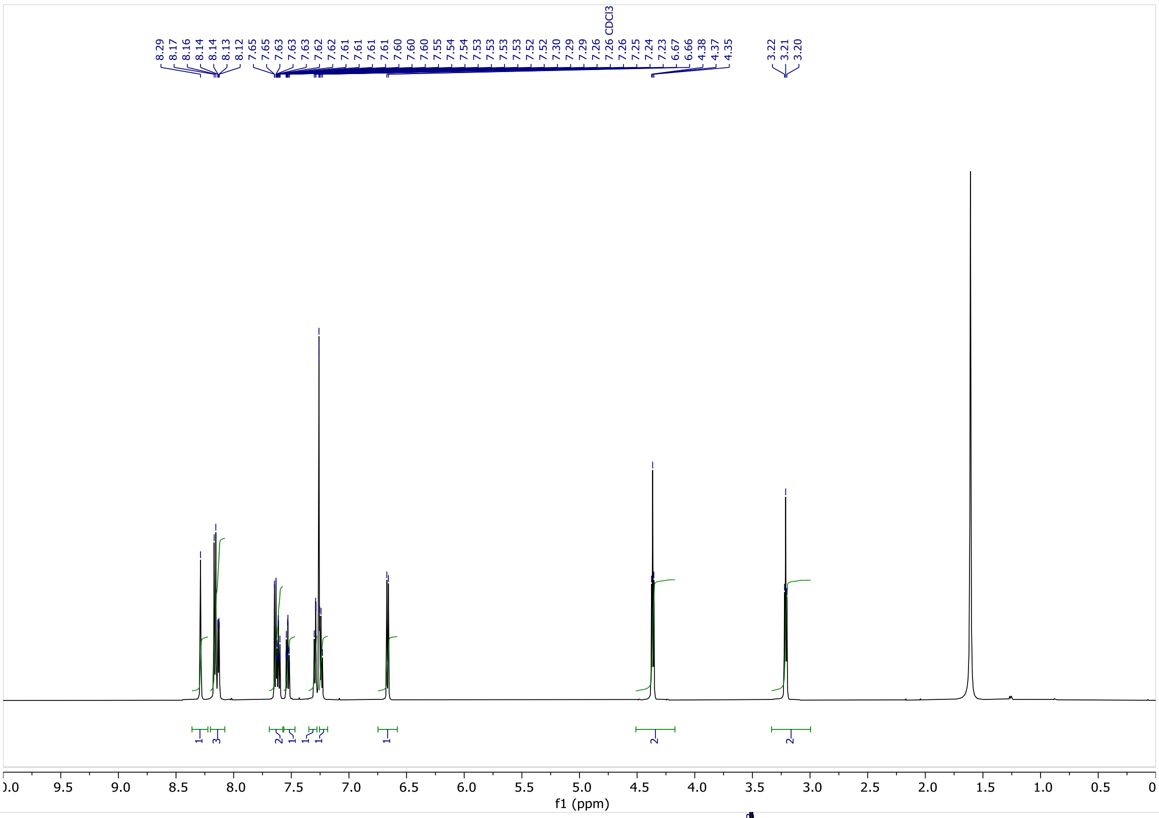

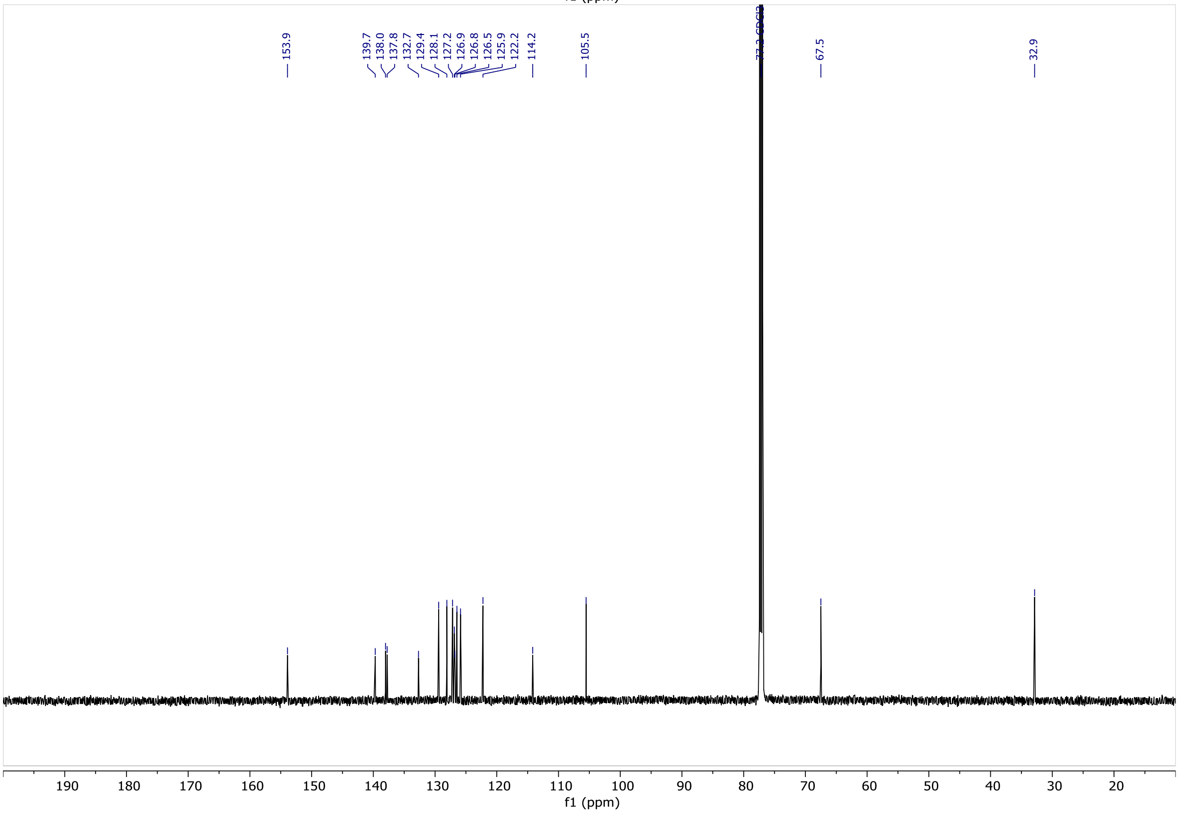

3-(2-((4-fluoronaphthalen-1-yl)oxy)ethyl)pyridine (13a)

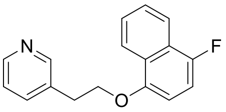

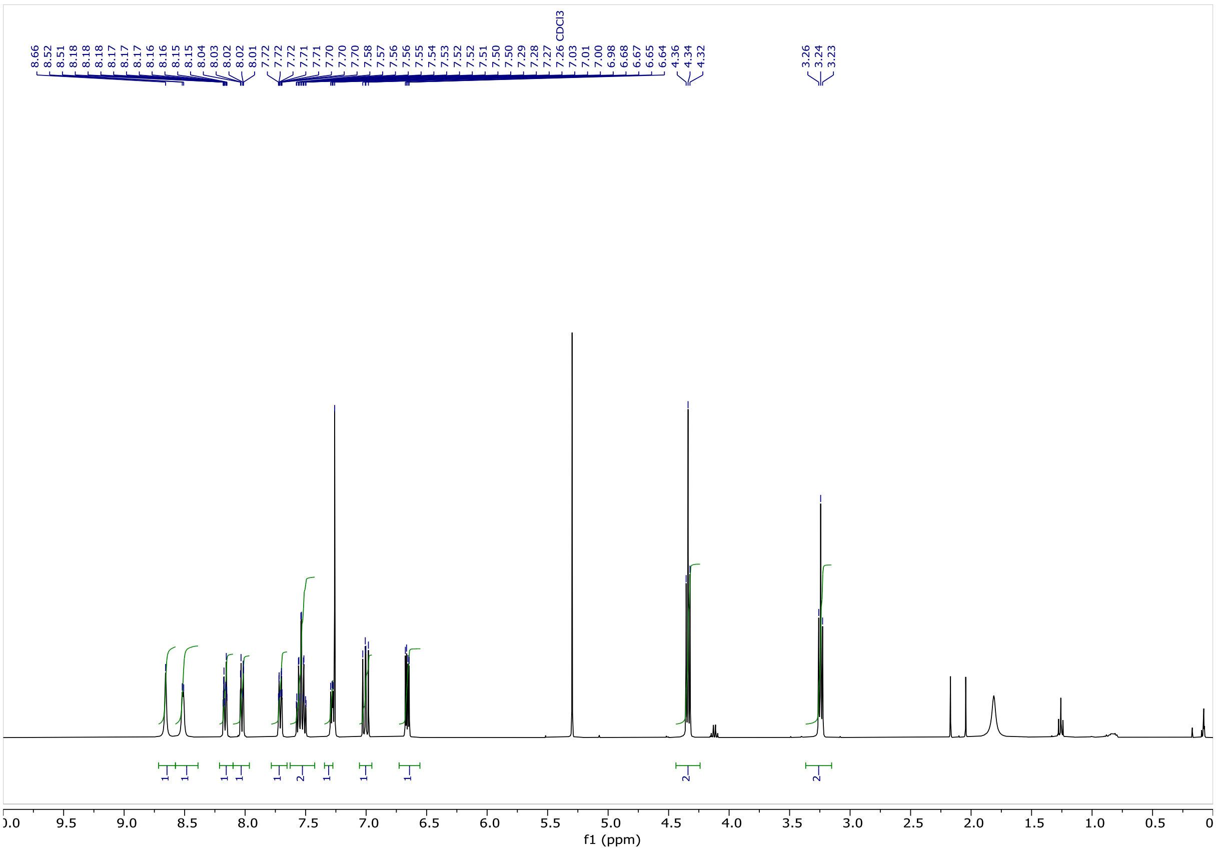

3-(2-((4-fluoronaphthalen-1-yl)oxy)ethyl)pyridine 1-oxide (13)

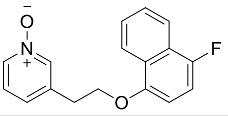

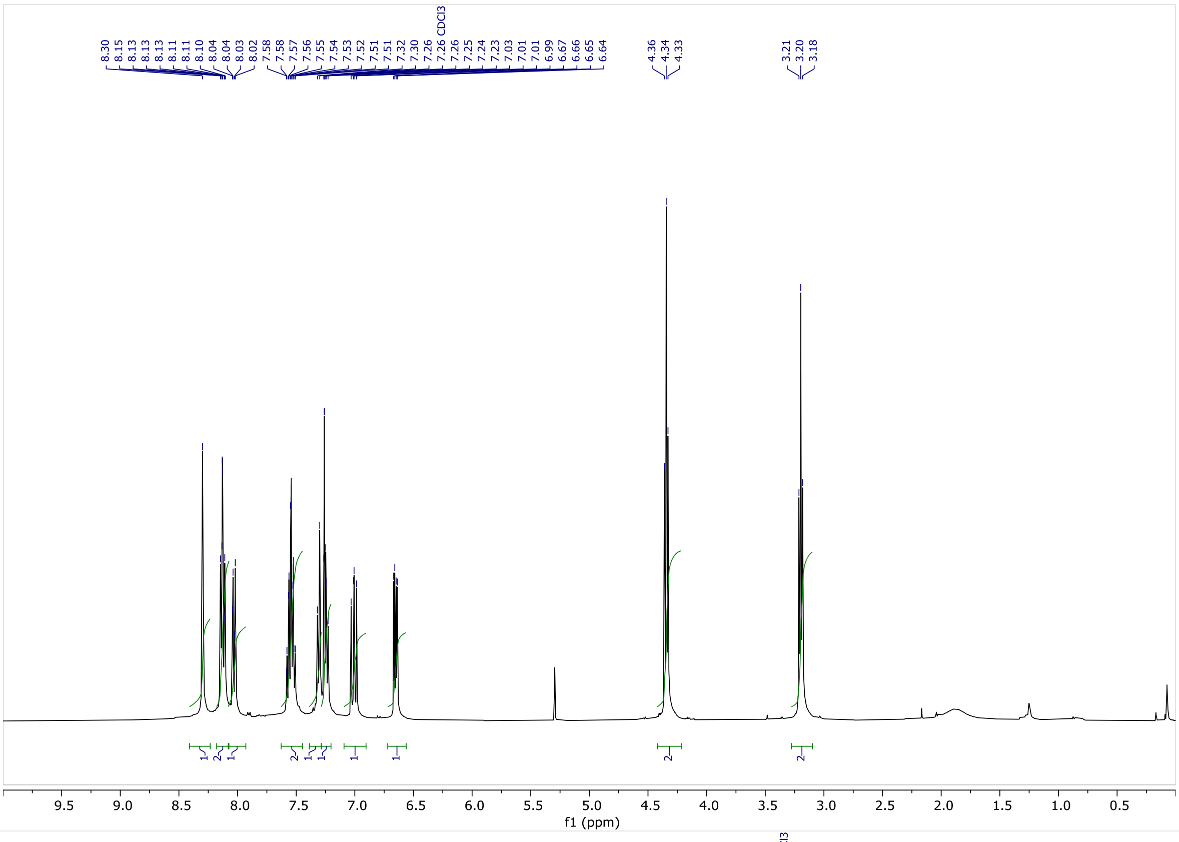

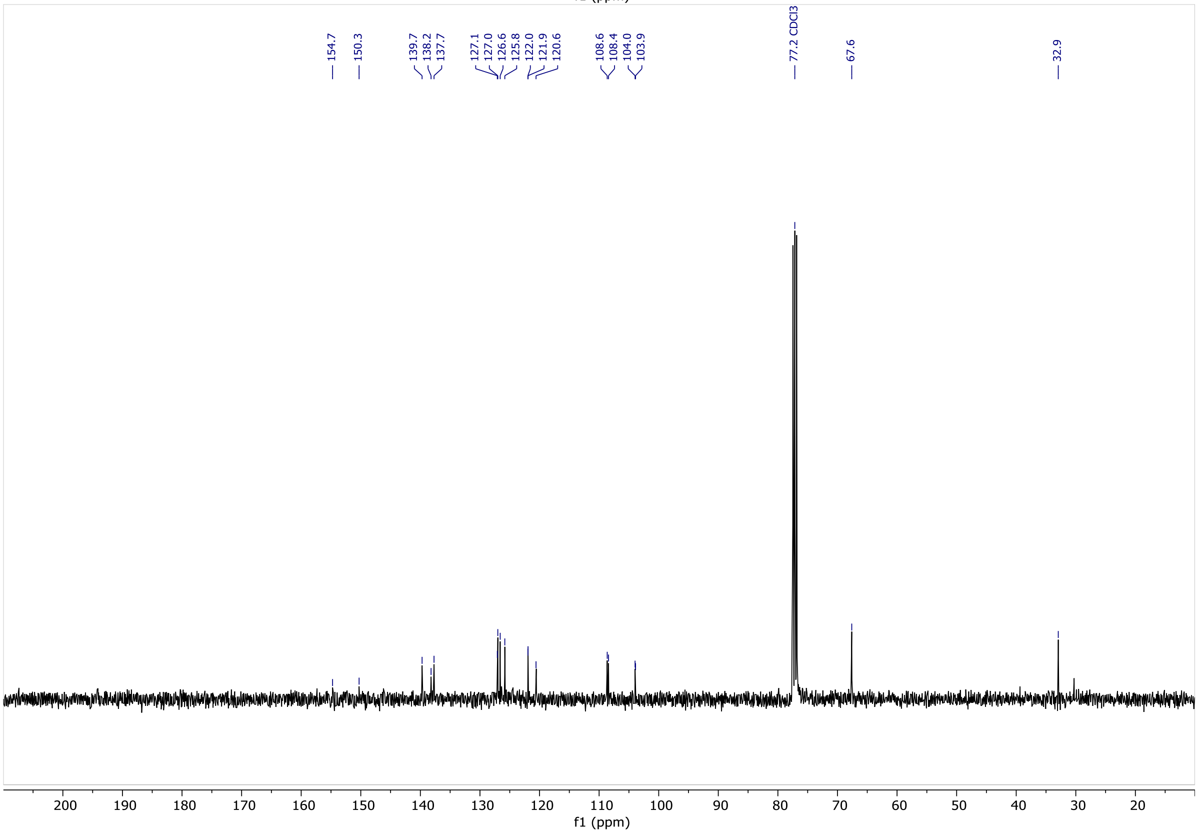

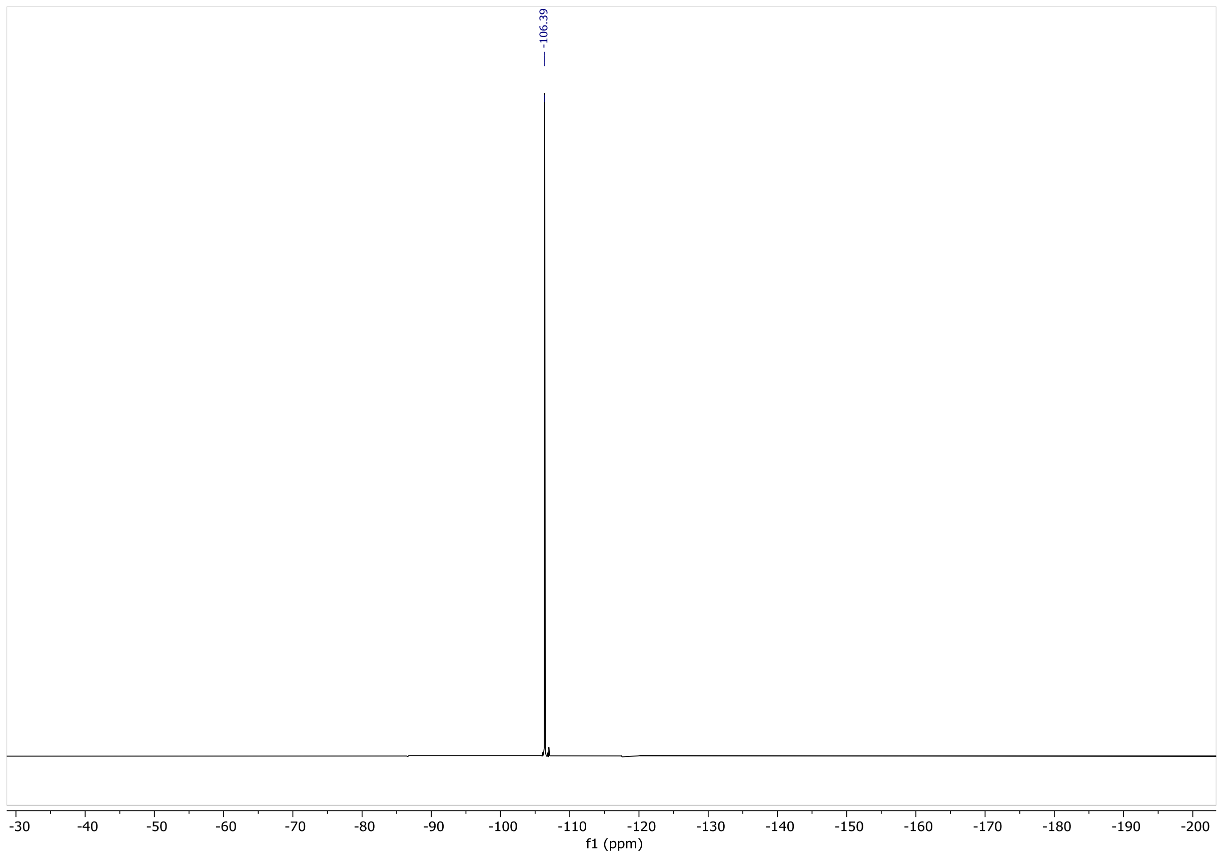
-106.39
-30 -40 -50 -60 -70 -80 -90 -100 -110 -120 -130 -140 -150 -160 -170 -180 -190 -200
f1 (ppm)

3-(2-((4-methoxynaphthalen-1-yl)oxy)ethyl)pyridine (14a)

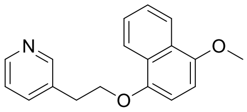

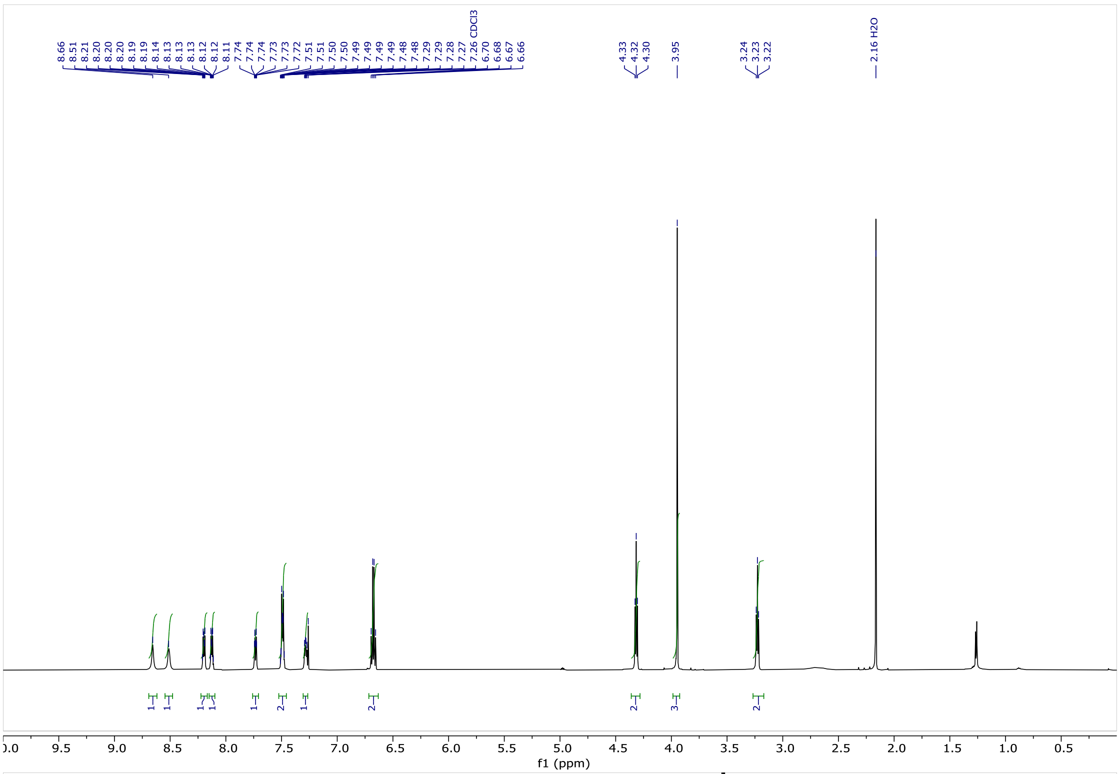

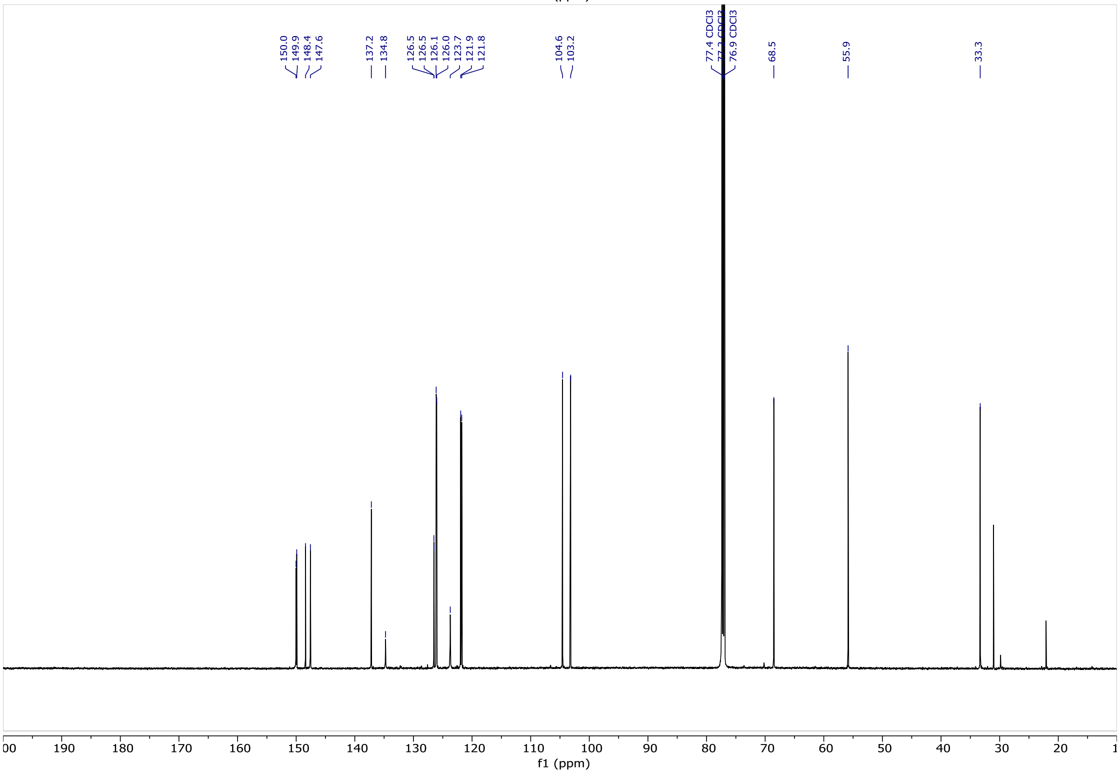

3-(2-((4-methoxynaphthalen-1-yl)oxy)ethyl)pyridine 1-oxide (14)

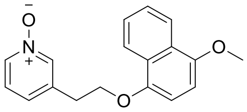

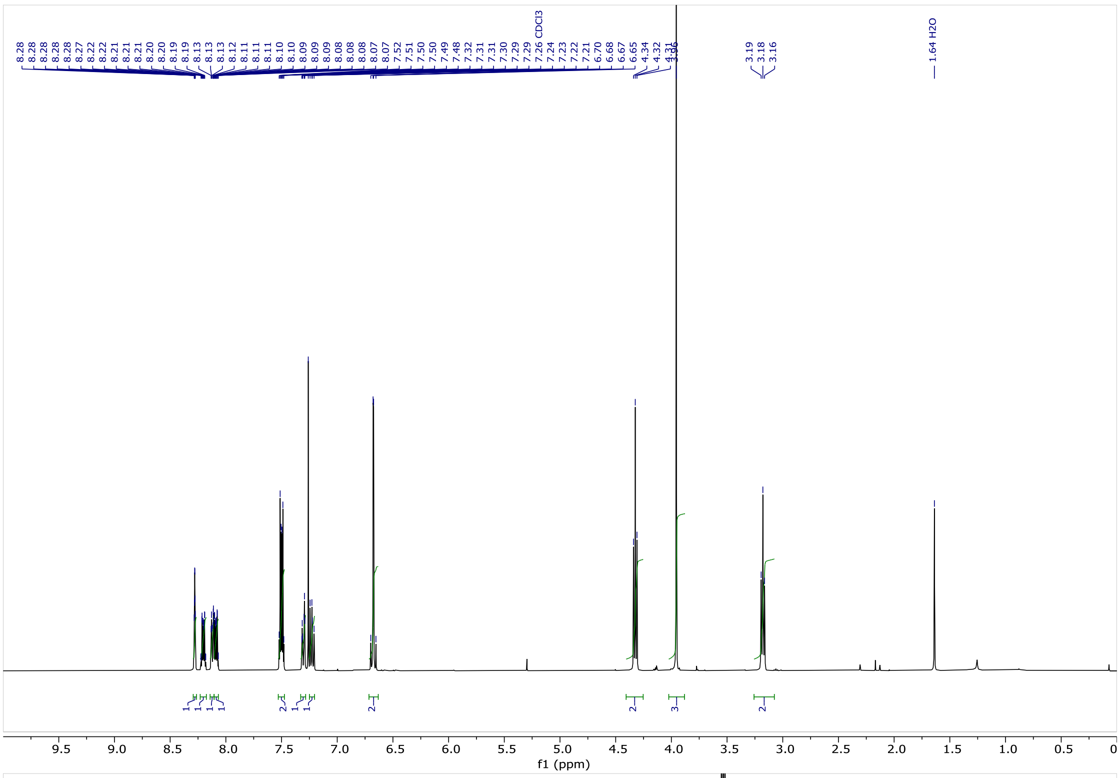

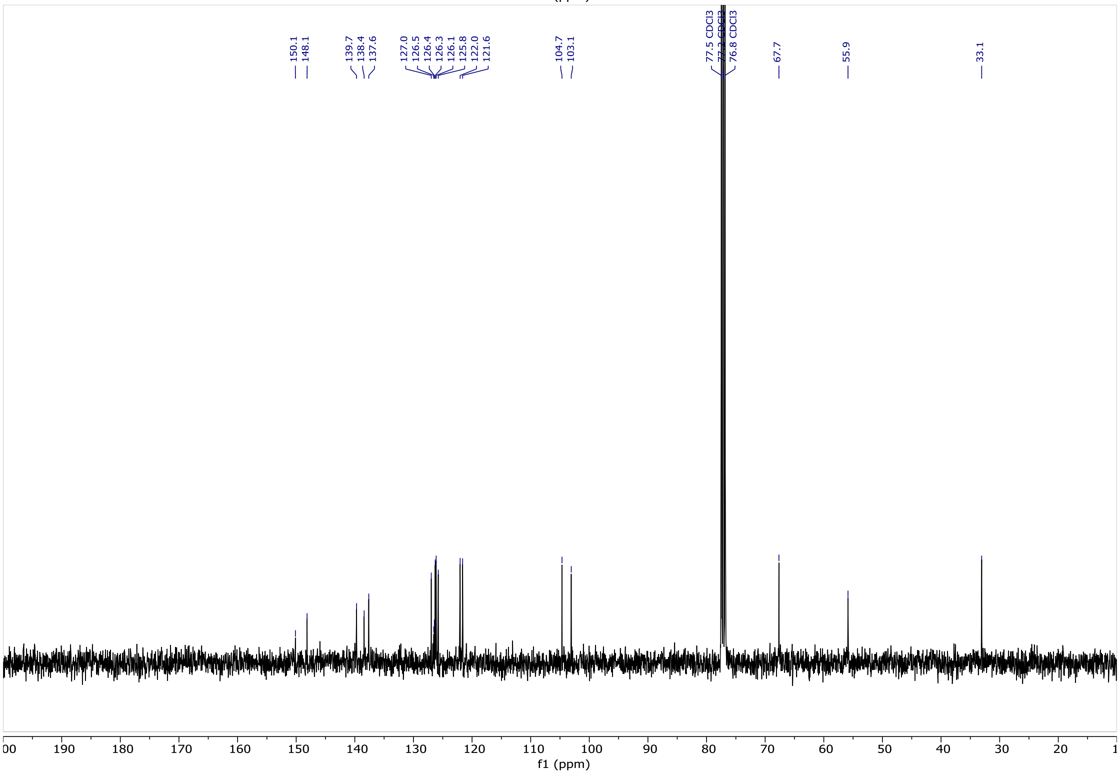

3-(2-(naphthalen-1-yloxy)ethyl)pyridine (15a)

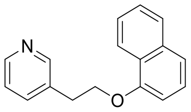

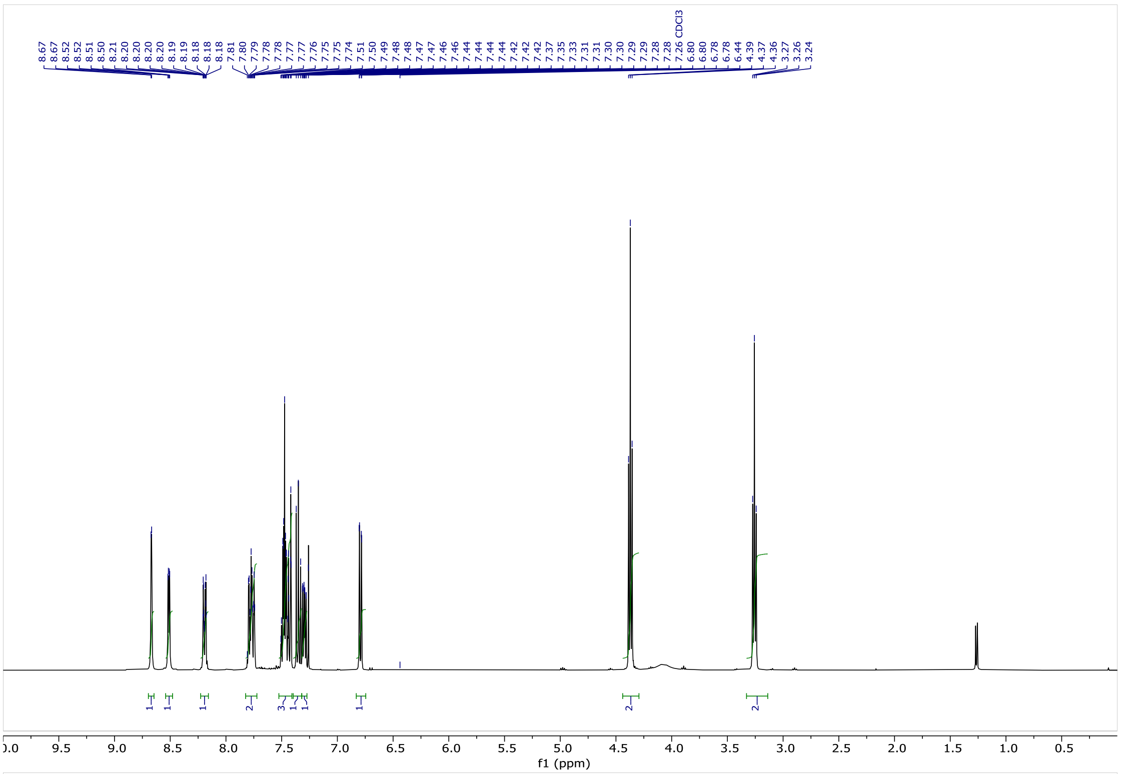

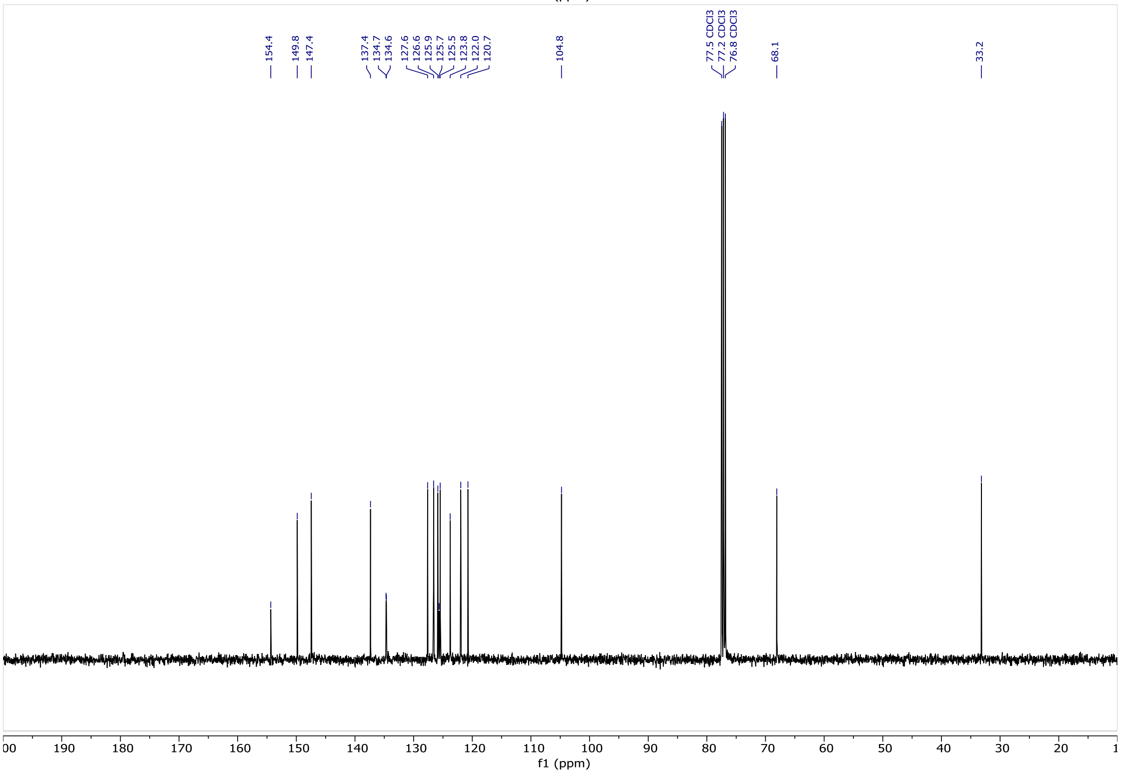

3-(2-(naphthalen-1-yloxy)ethyl)pyridine 1-oxide (15)

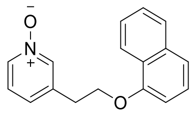

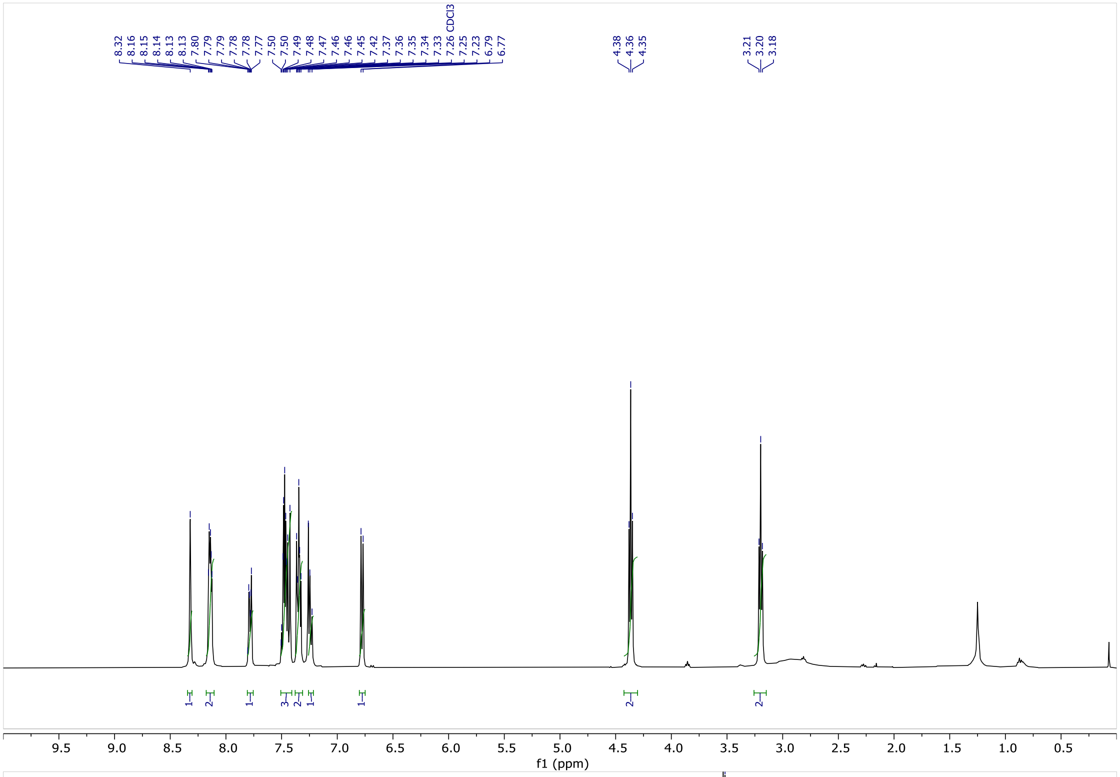

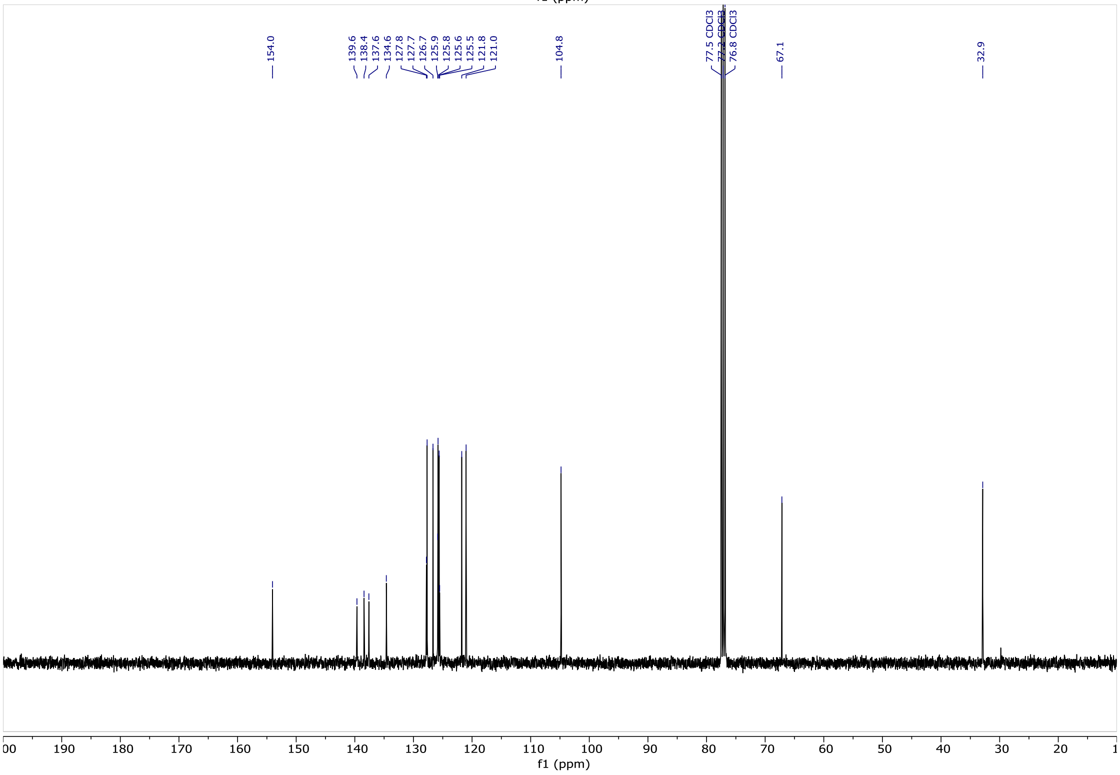

3-(2-((4-chloro-5,6,7,8-tetrahydronaphthalen-1-yl)oxy)ethyl)pyridine (16a)

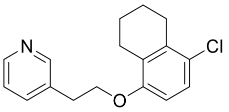

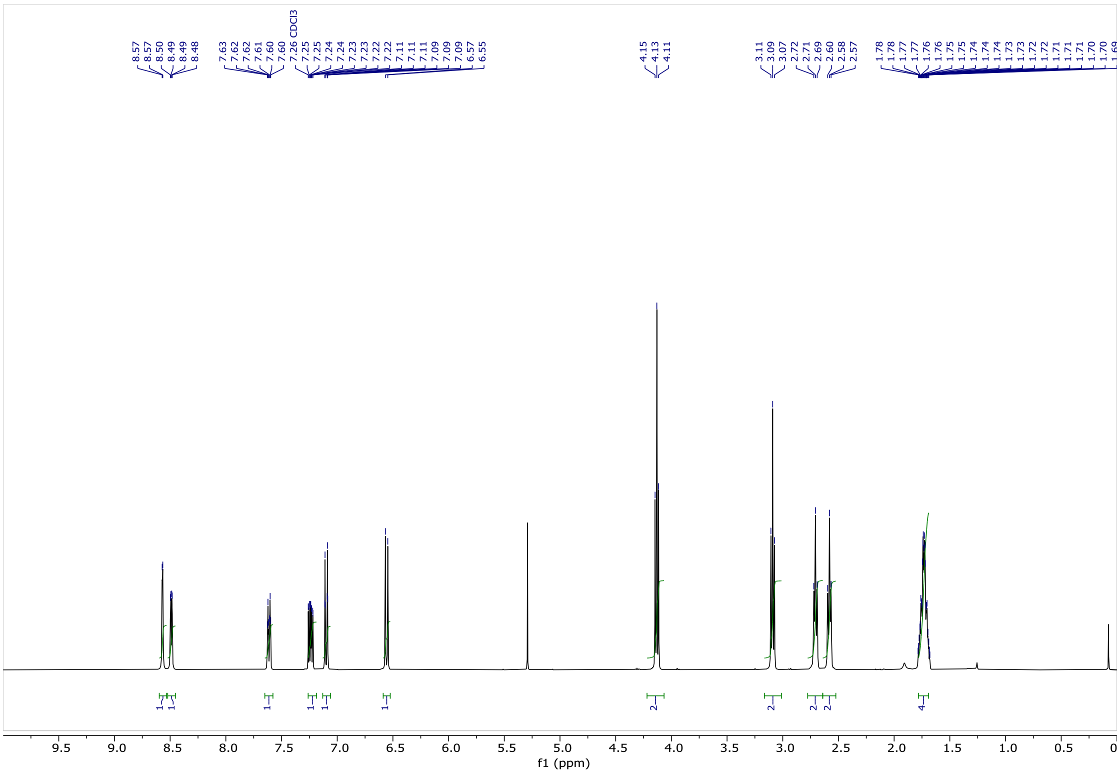

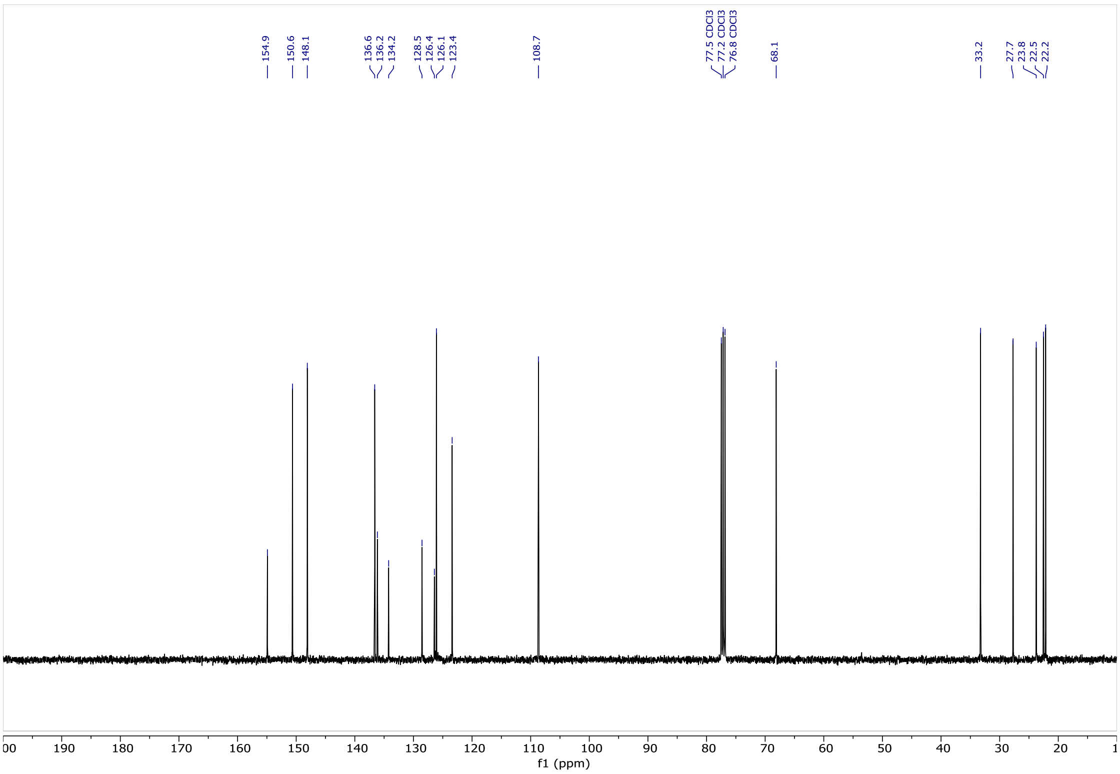

3-(2-((4-chloro-5,6,7,8-tetrahydronaphthalen-1-yl)oxy)ethyl)pyridine 1-oxide (16)

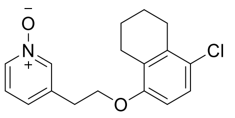

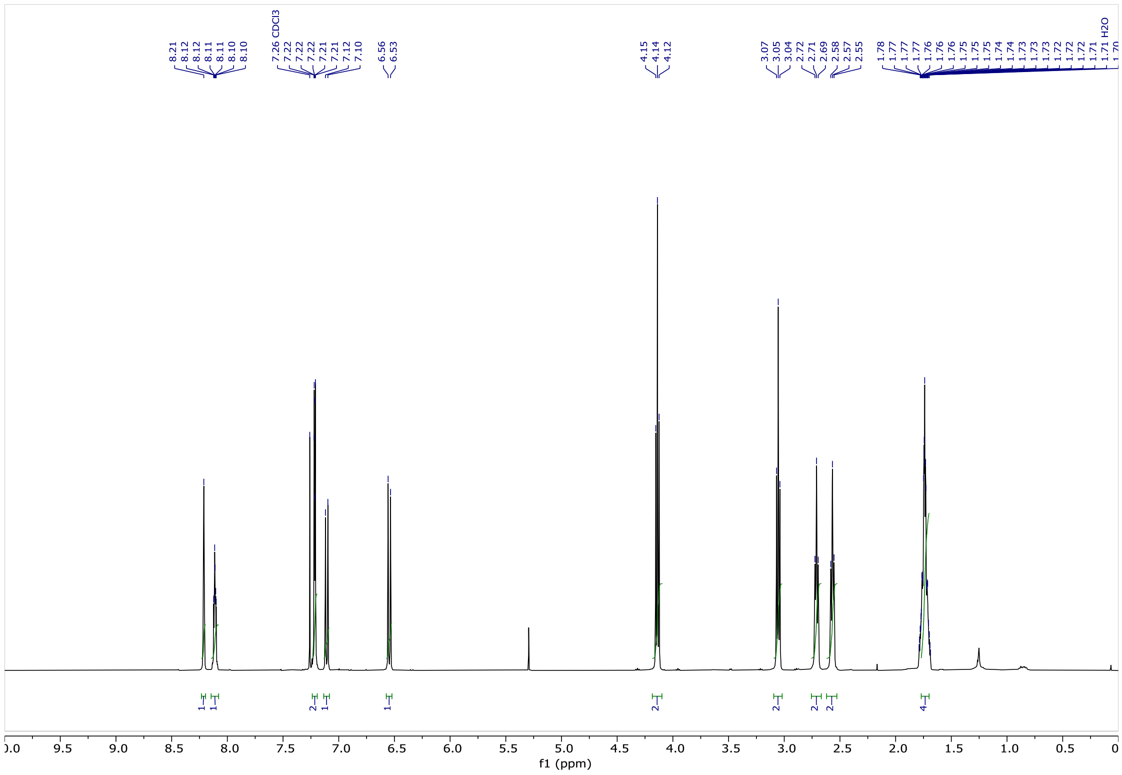

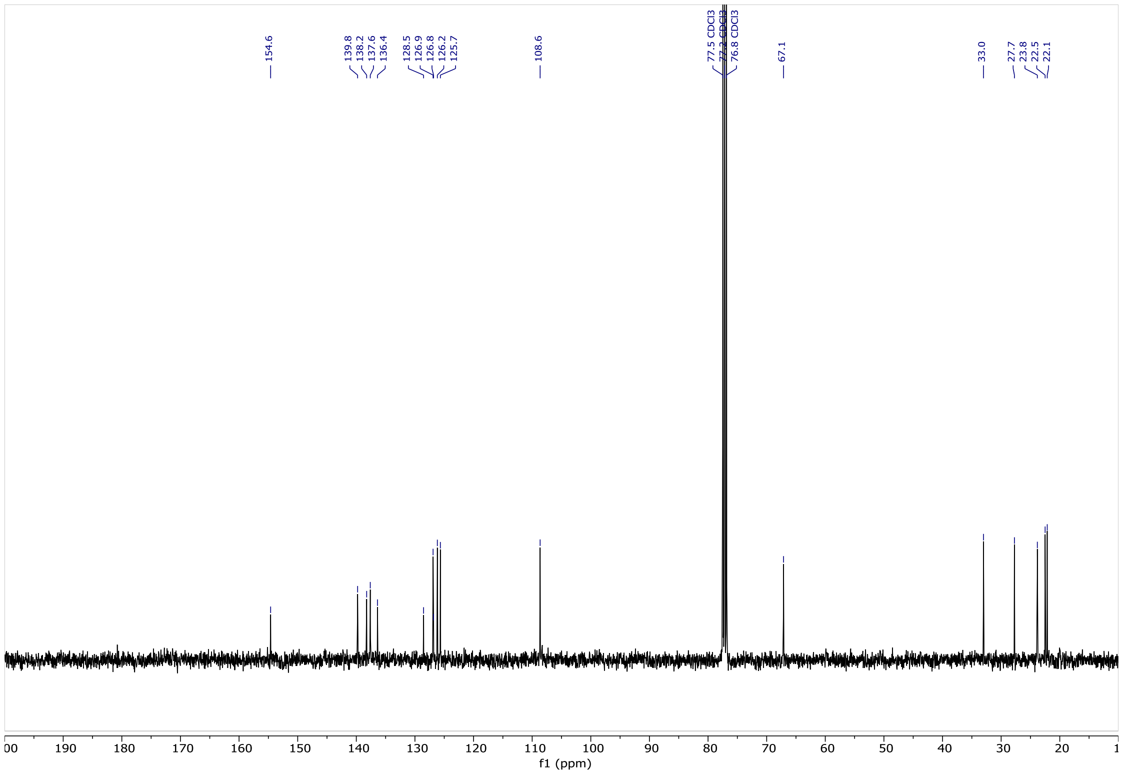

3-(2-((1,2,3,4-tetrahydronaphthalen-1-yl)oxy)ethyl)pyridine 1-oxide (17)

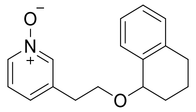

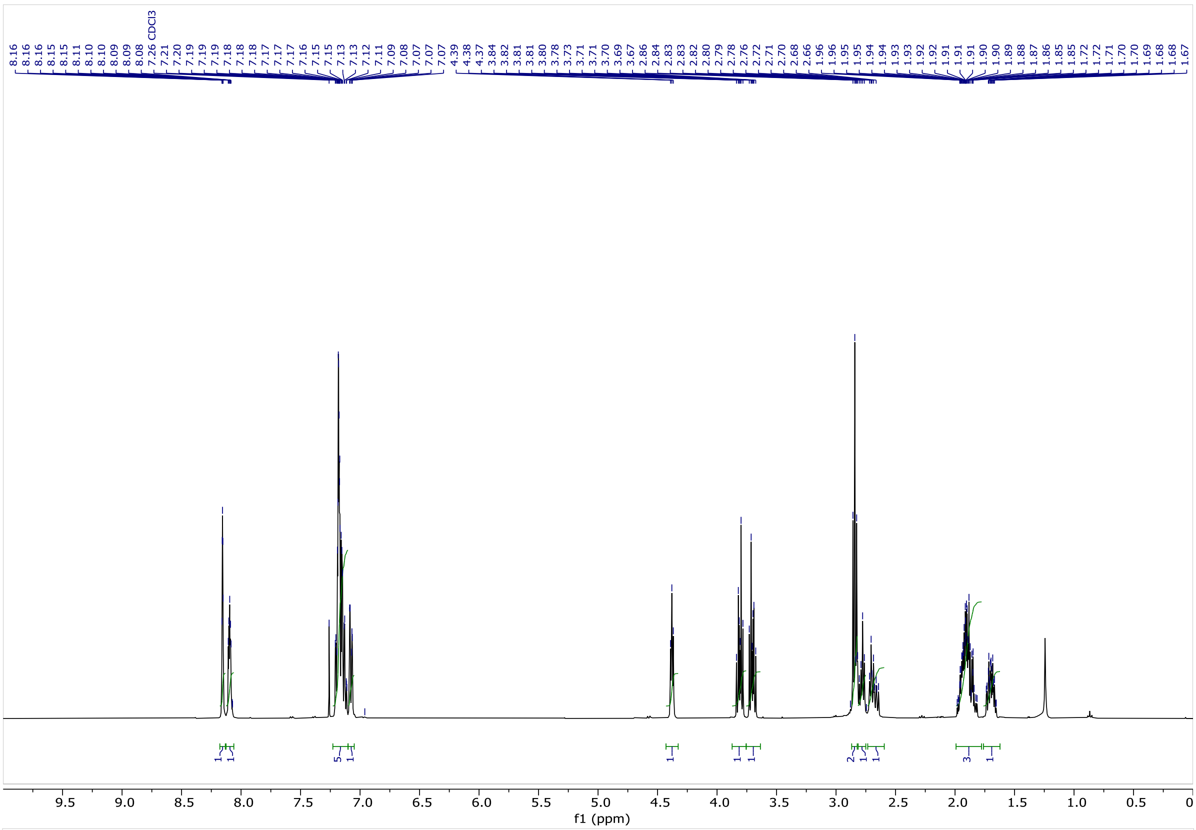

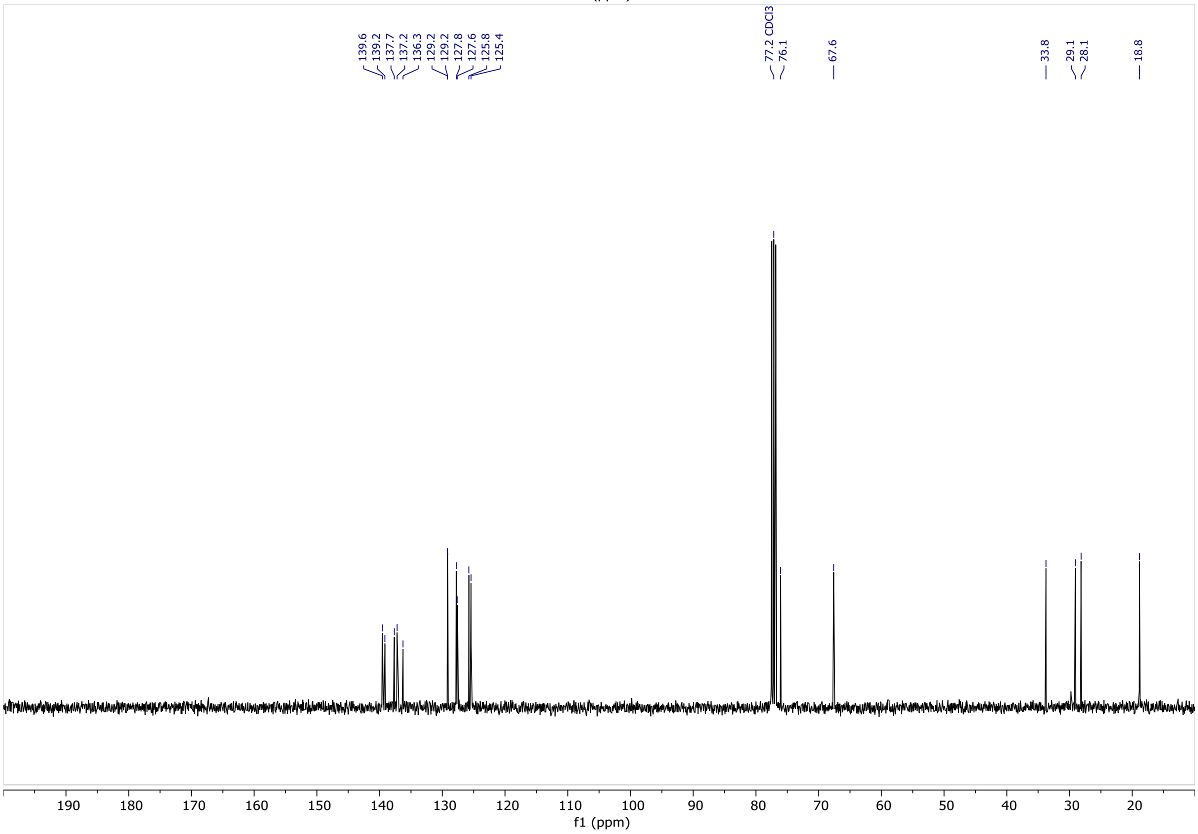

3-(2-((2,3-dihydro-1H-inden-1-yl)oxy)ethyl)pyridine 1-oxide (18)

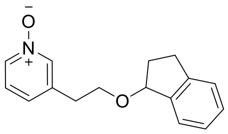

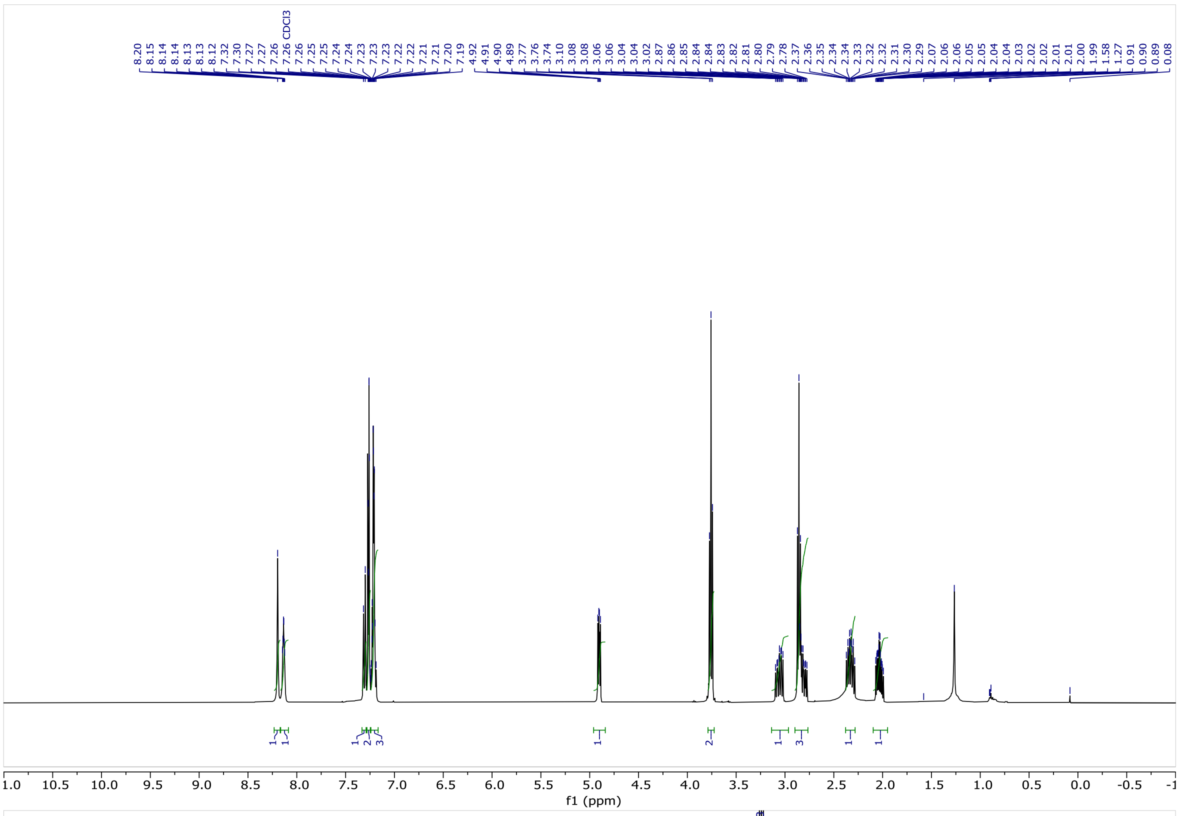

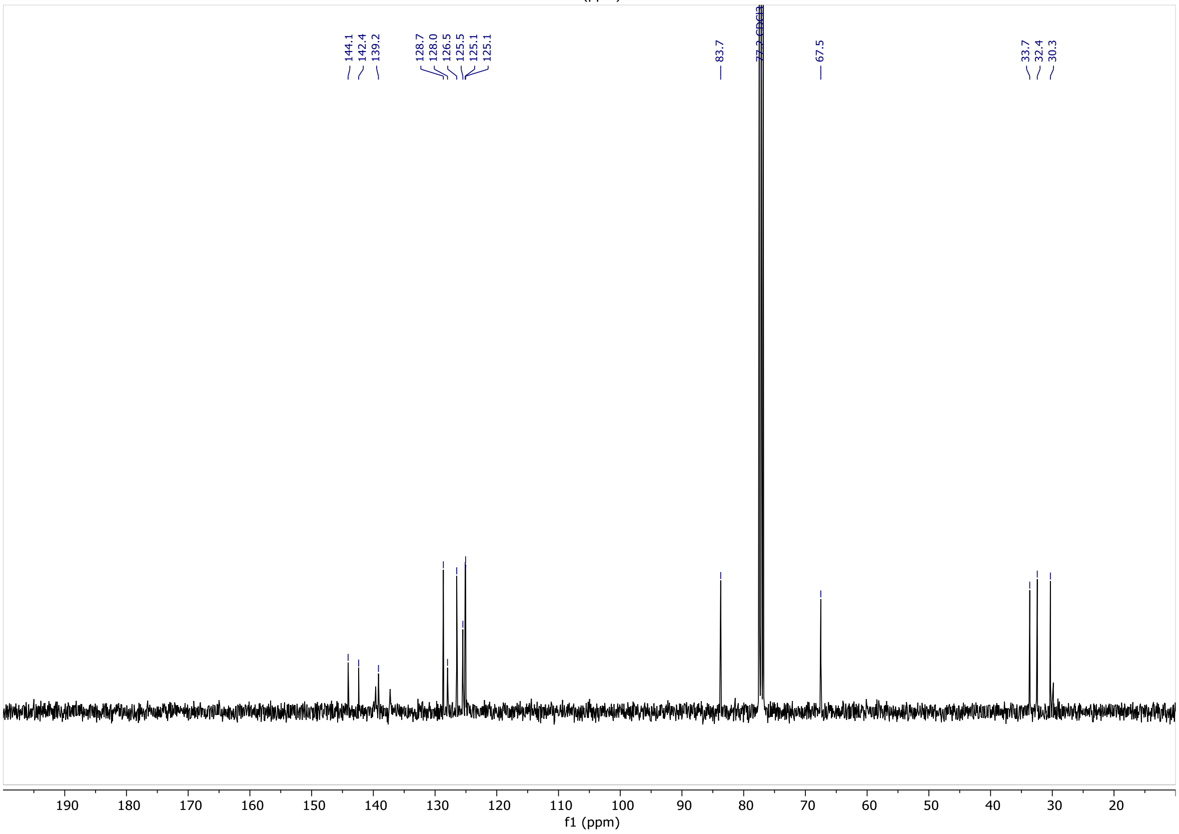

3-(2-(4-chlorophenoxy)ethyl)pyridine (19a)

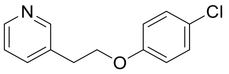

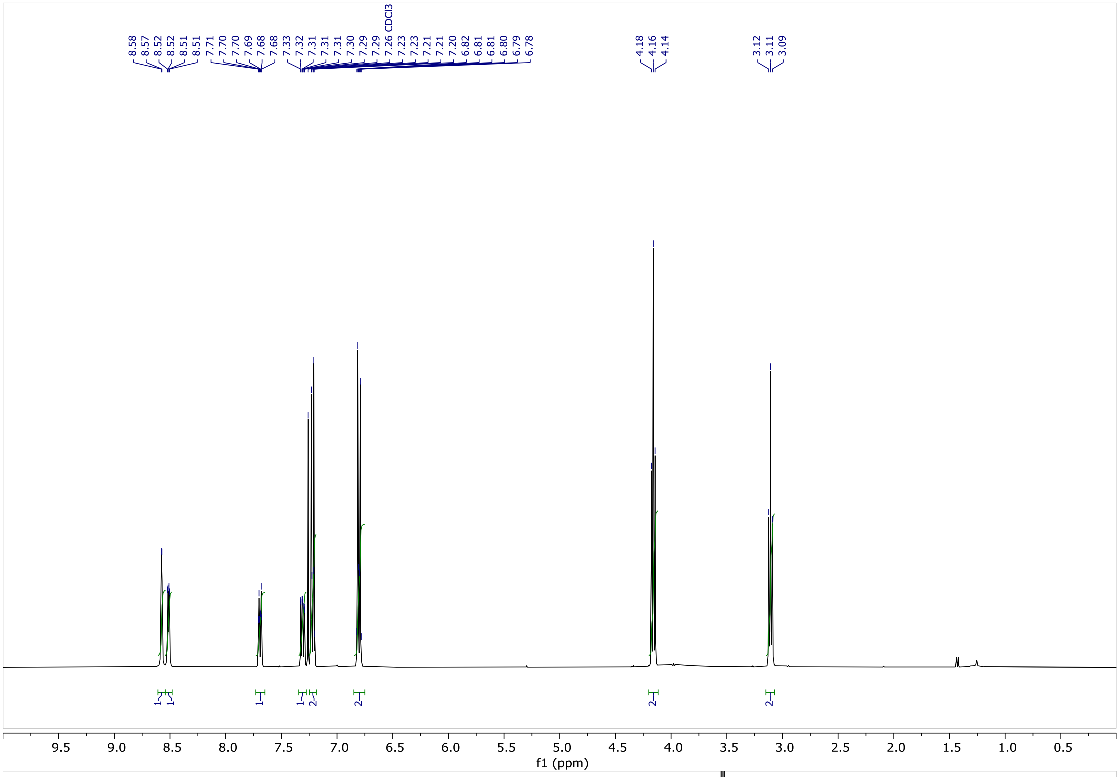

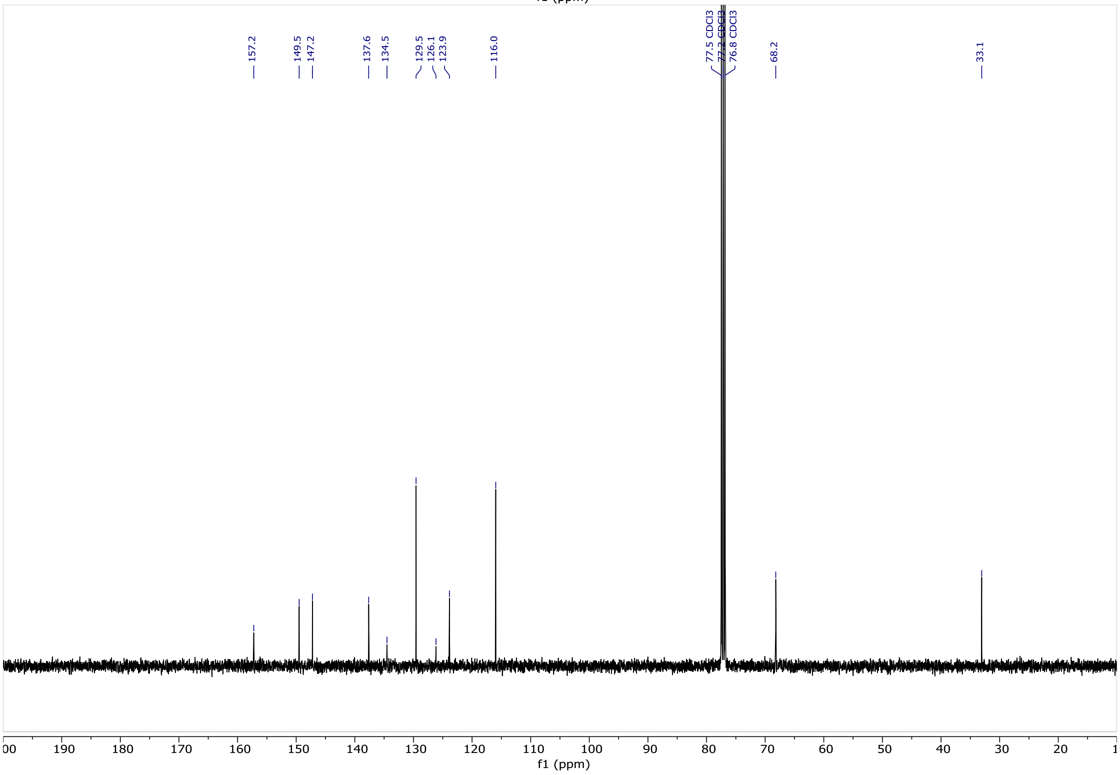

3-(2-(4-chlorophenoxy)ethyl)pyridine 1-oxide (19)

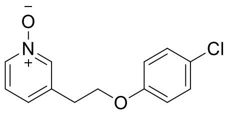

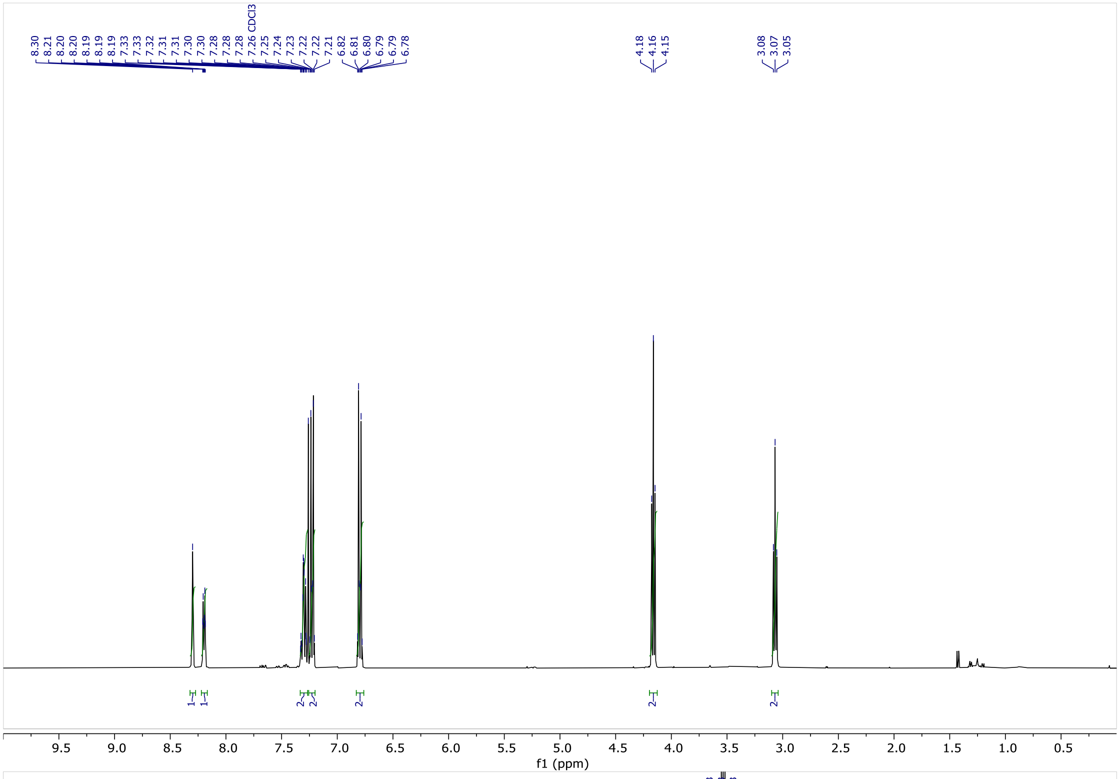

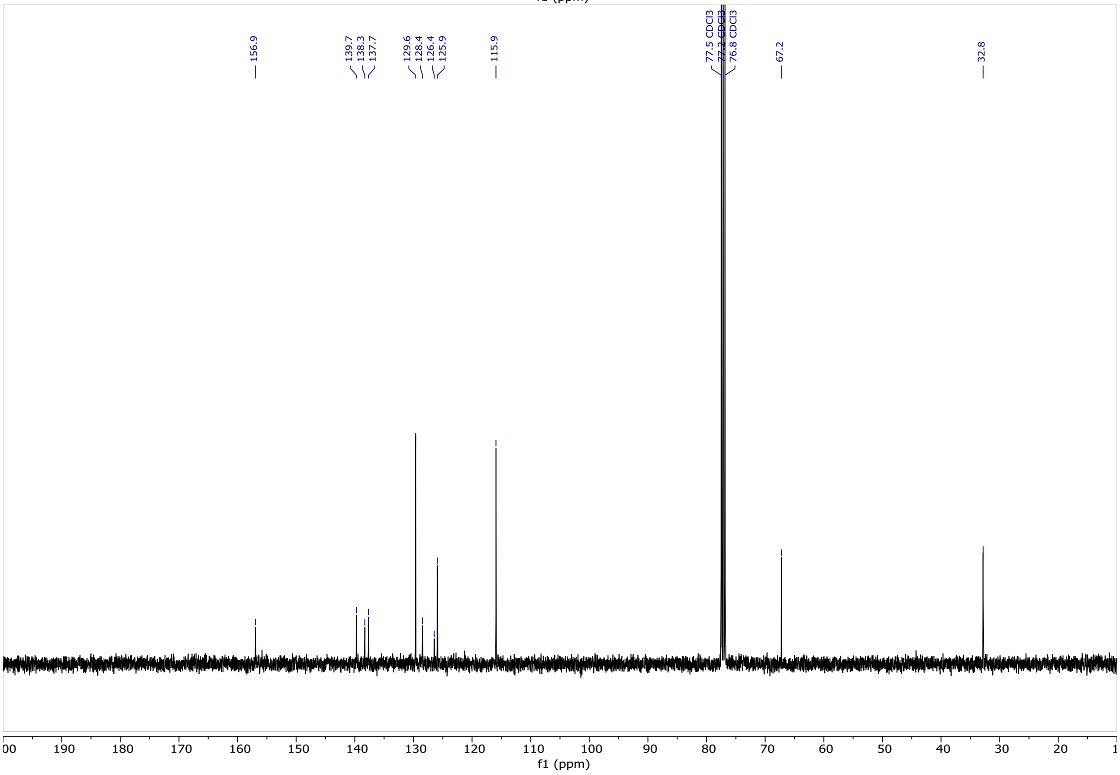

3-(2-(4-bromophenoxy)ethyl)pyridine (20a)

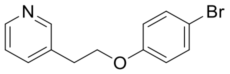

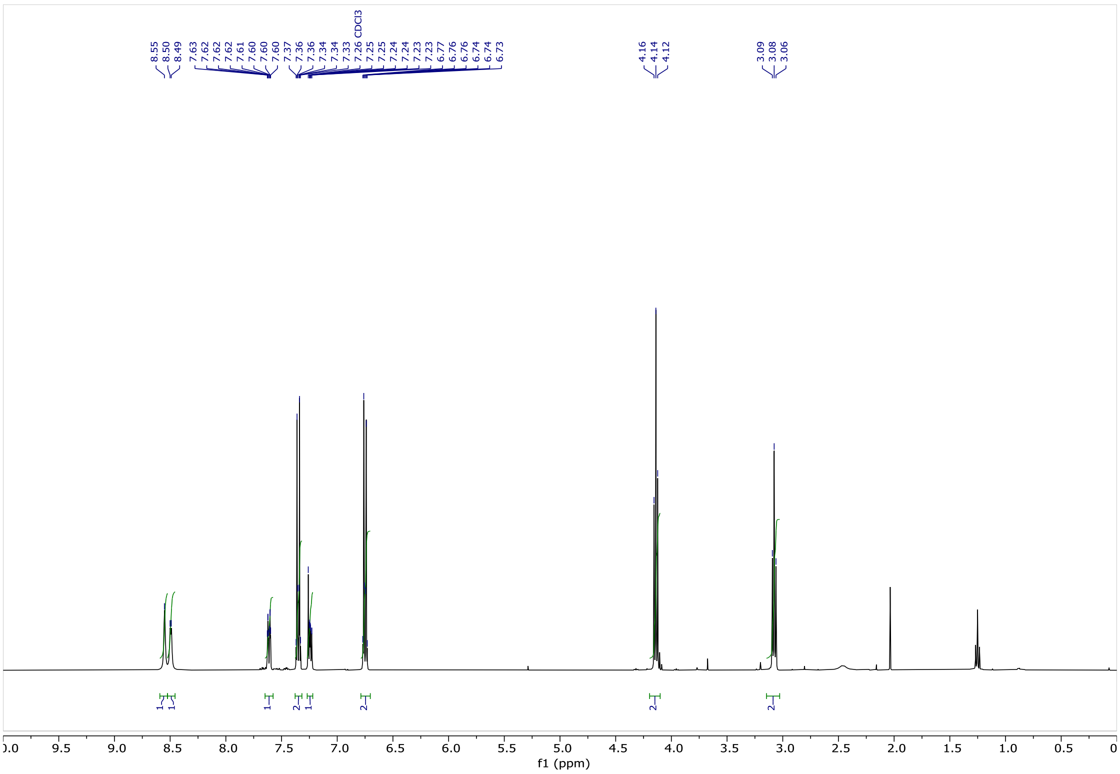

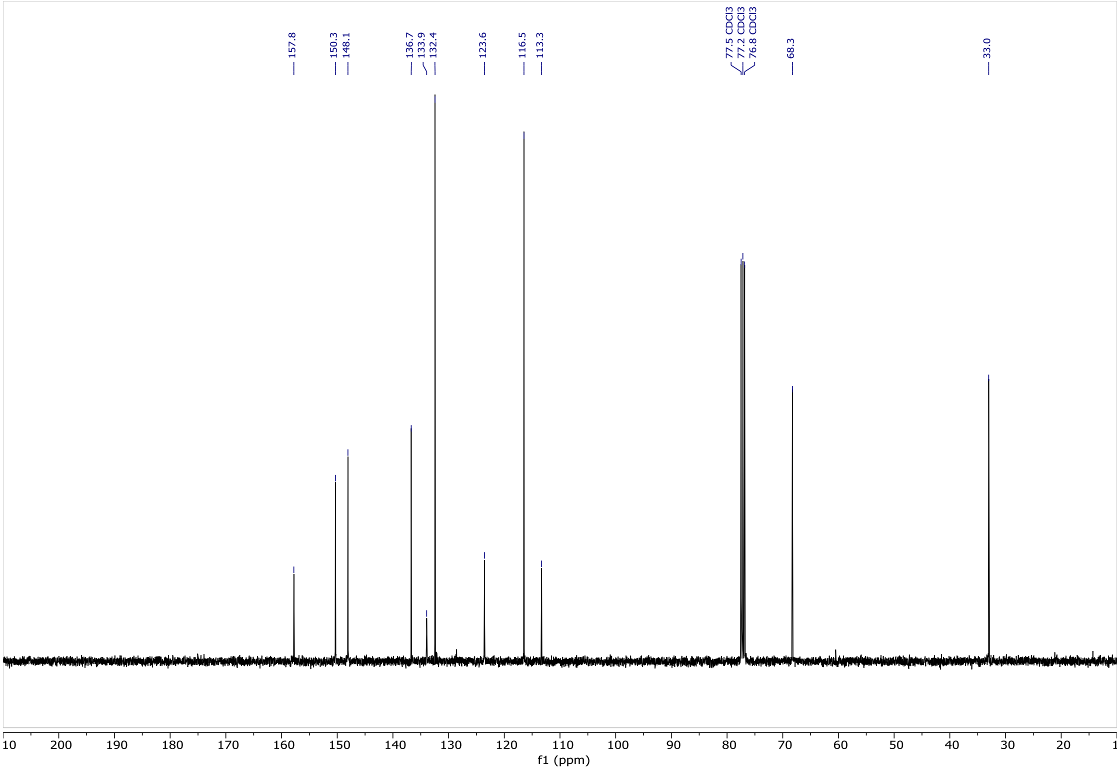

3-(2-(4-bromophenoxy)ethyl)pyridine 1-oxide (20)

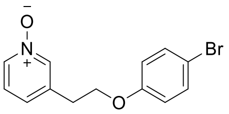

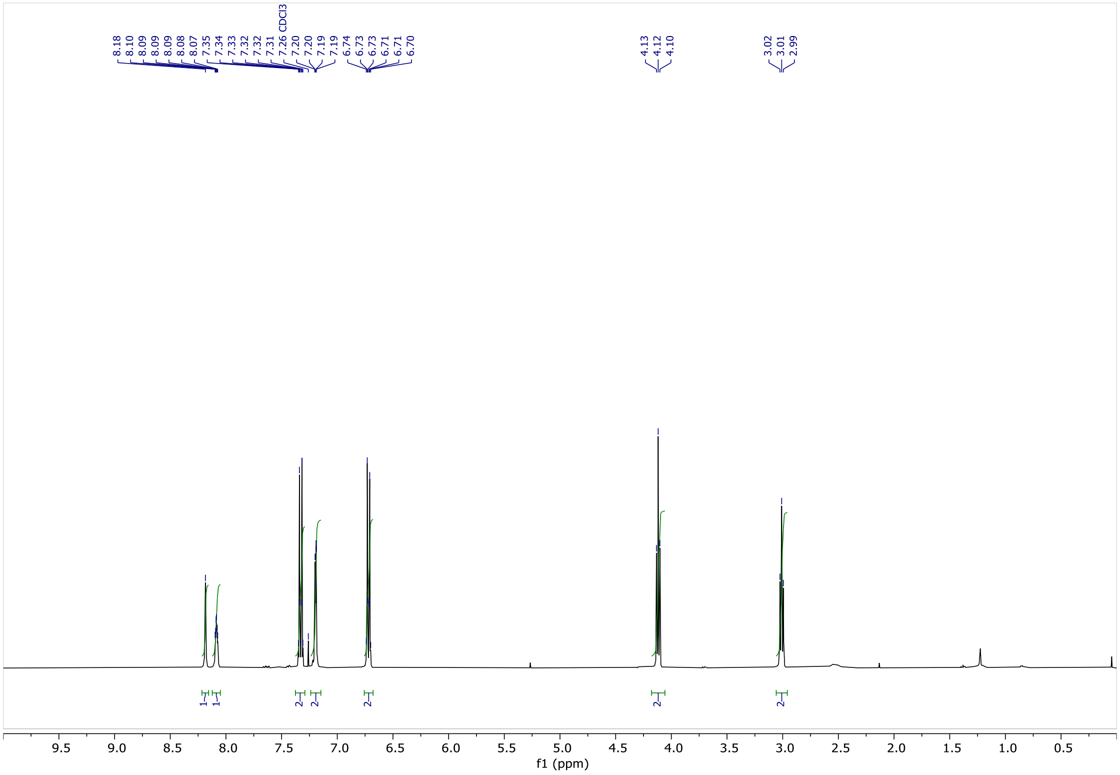

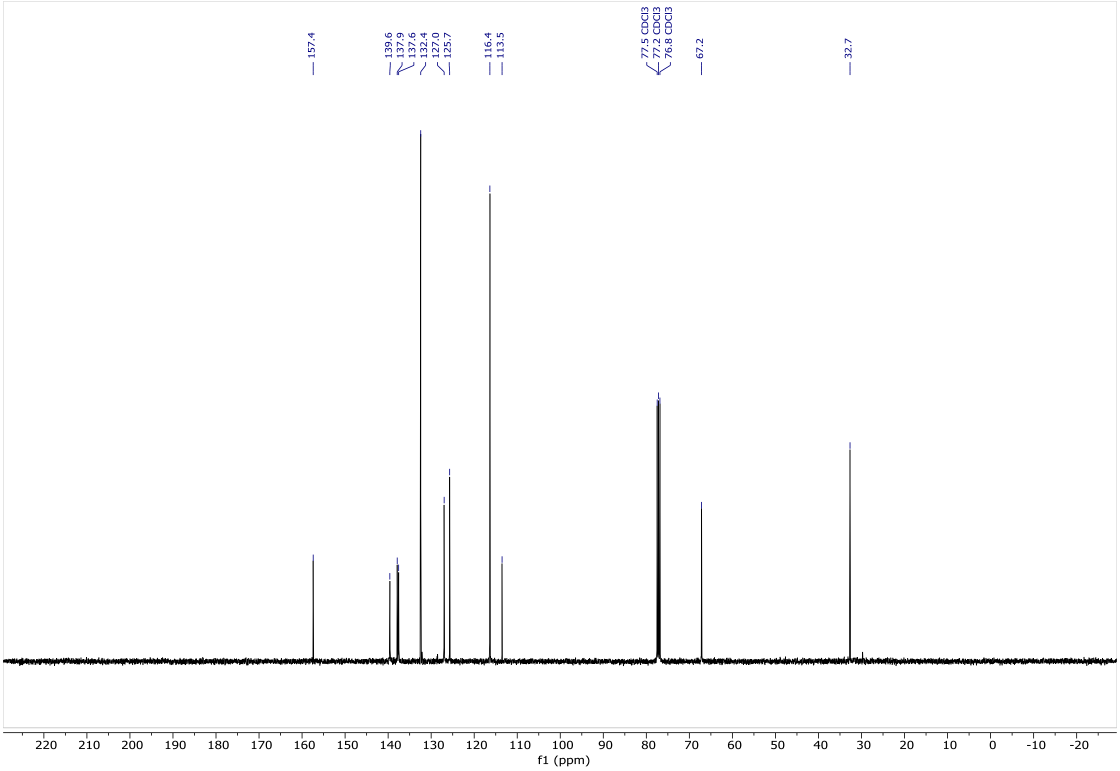

3-(2-(4-iodophenoxy)ethyl)pyridine (21a)

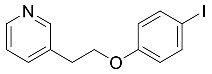

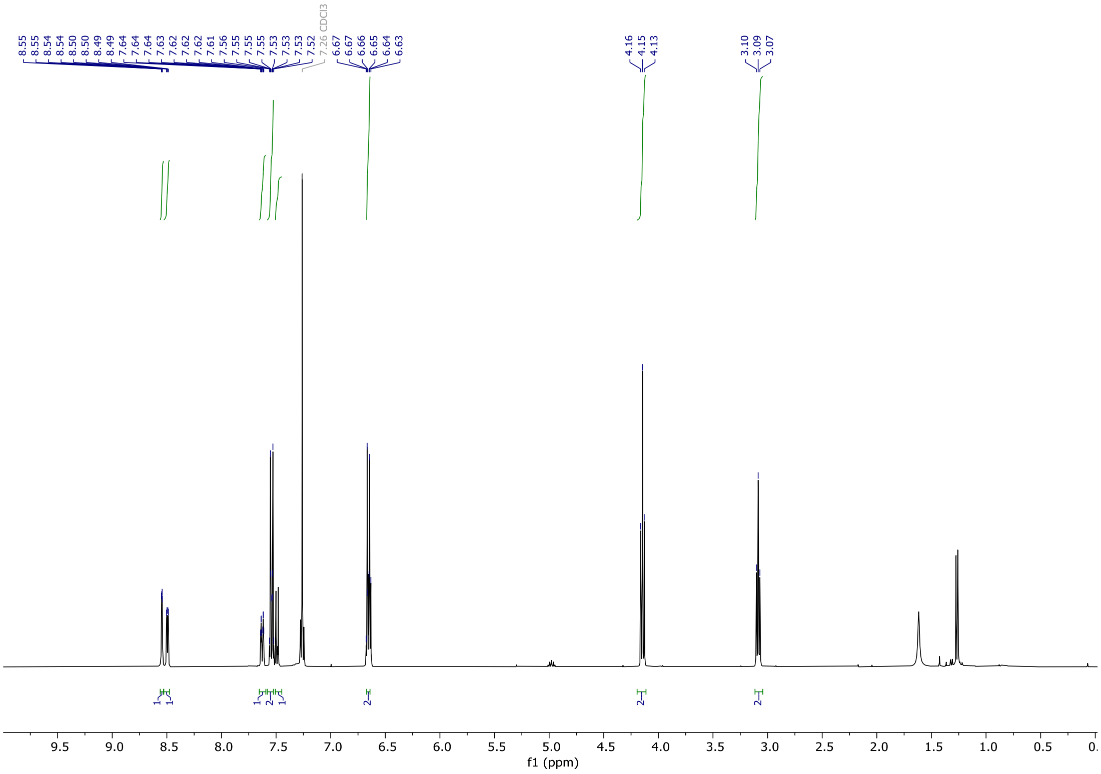

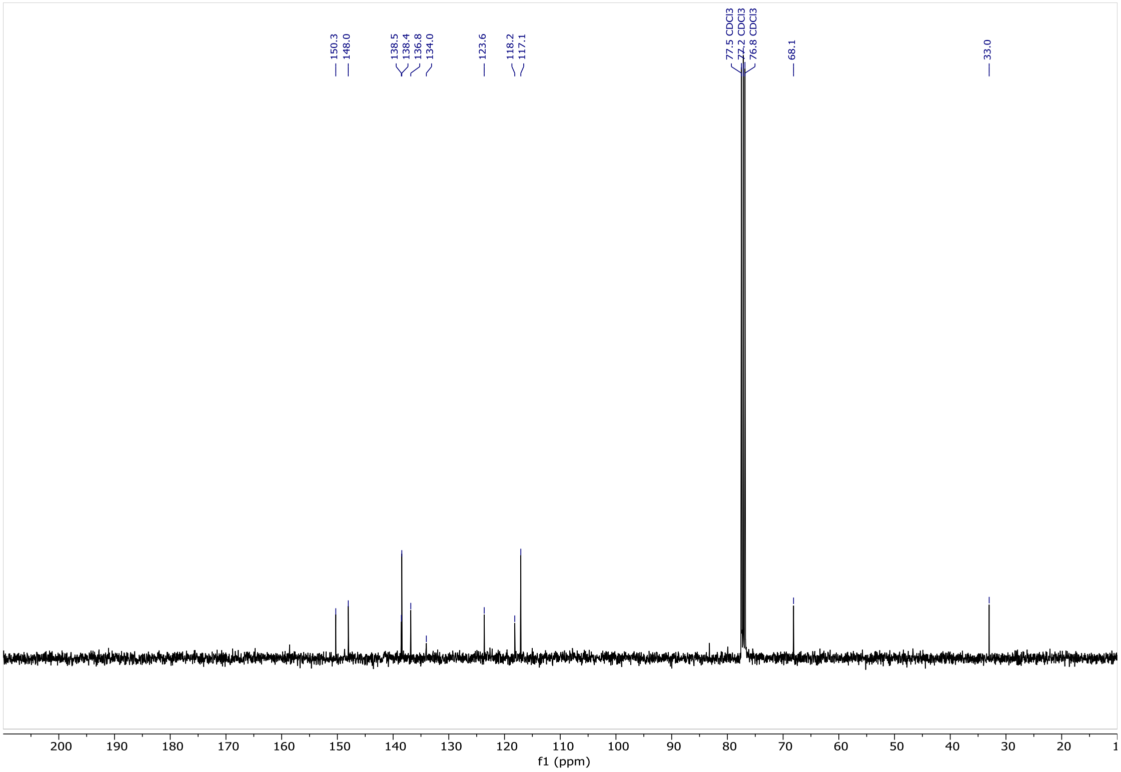

3-(2-(4-cyanophenoxy)ethyl)pyridine 1-oxide (21)

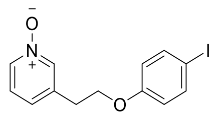

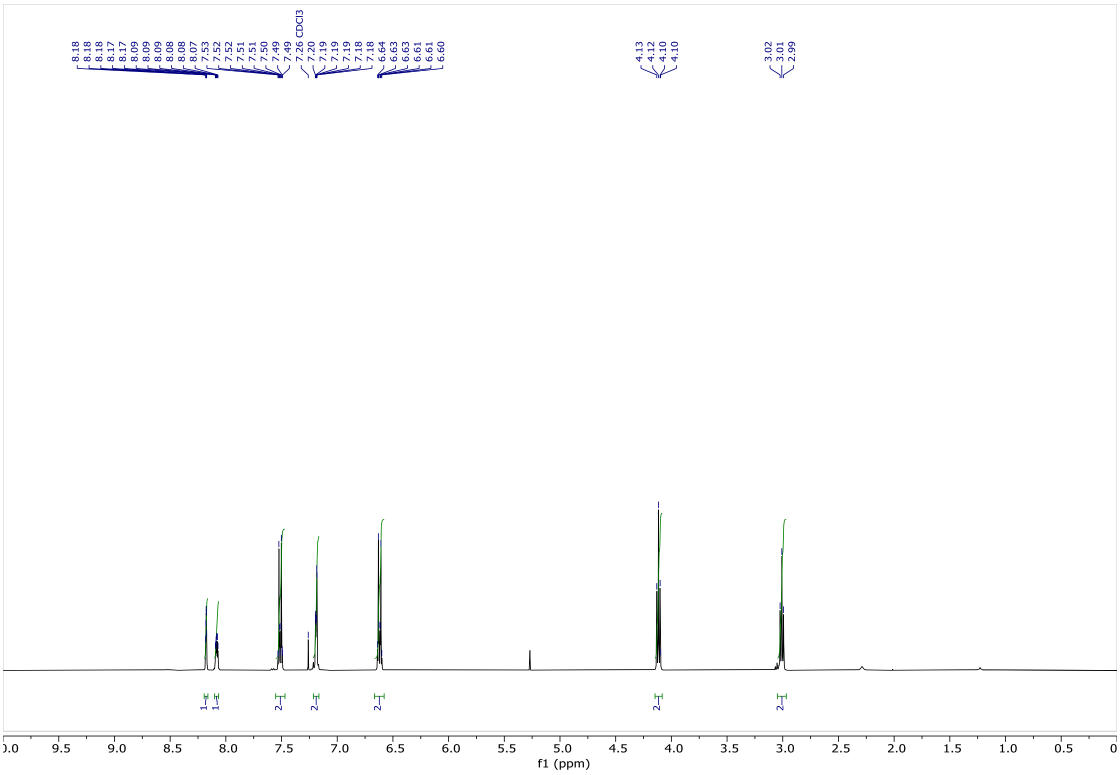

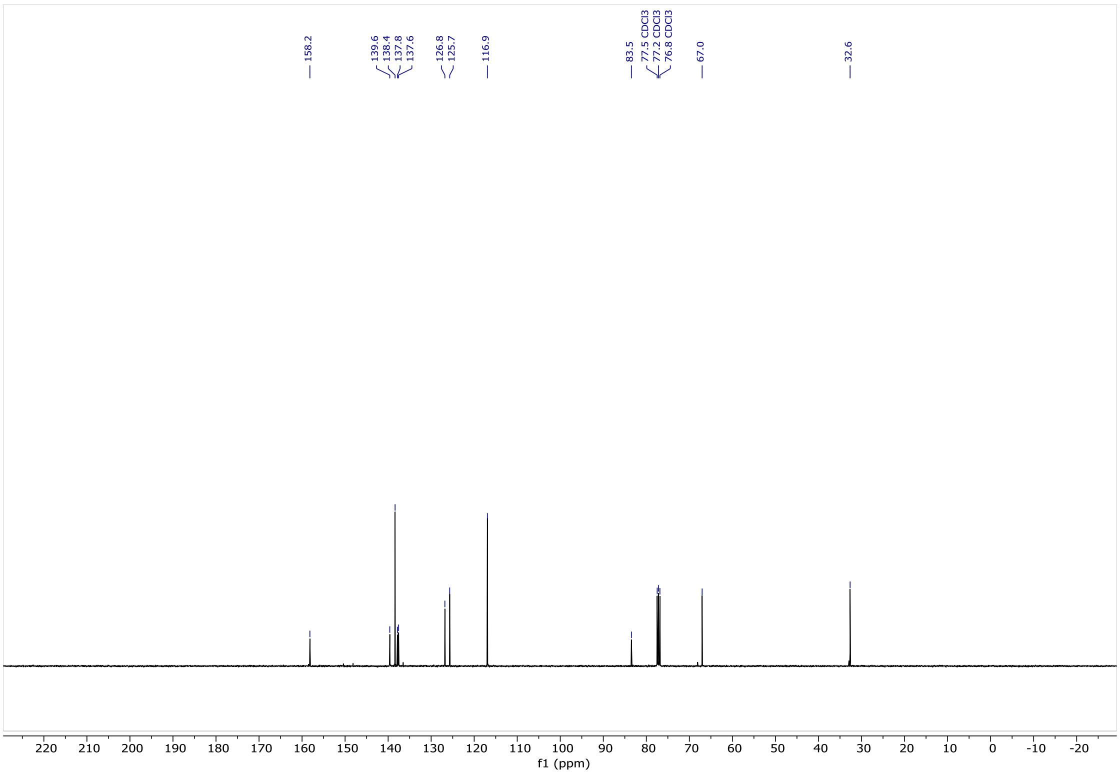

4-(2-(pyridin-3-yl)ethoxy)benzonitrile (22a)

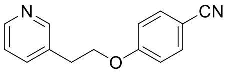

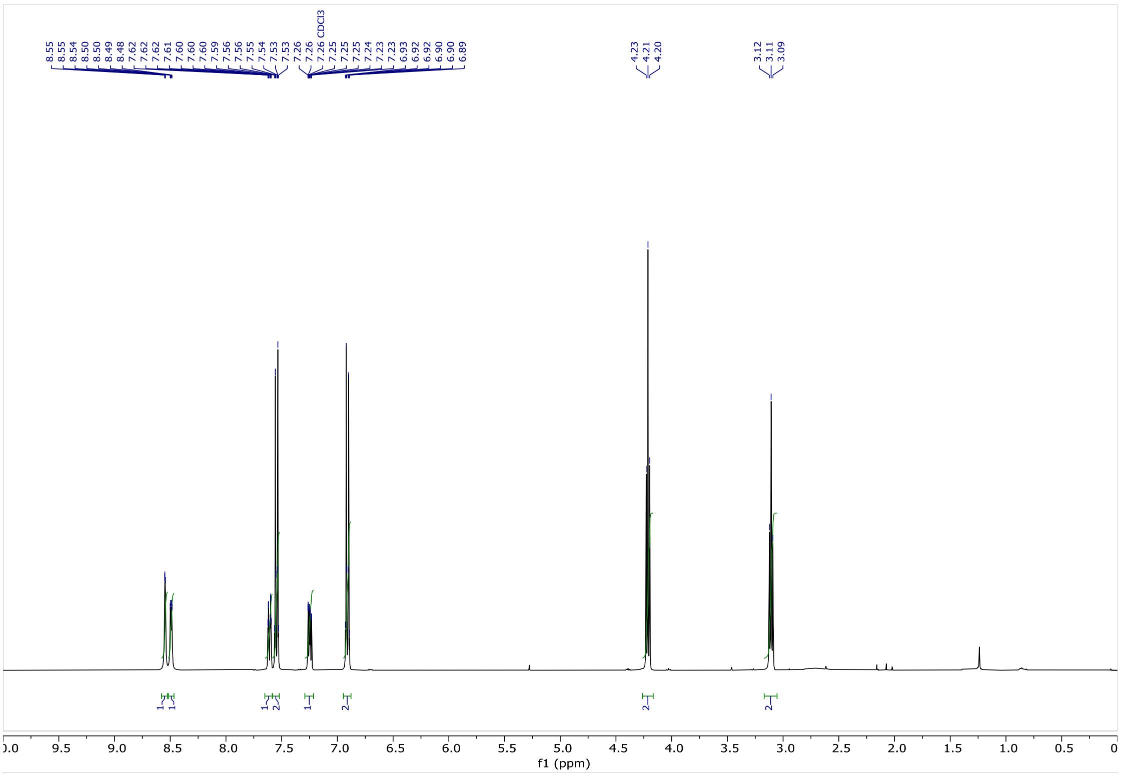

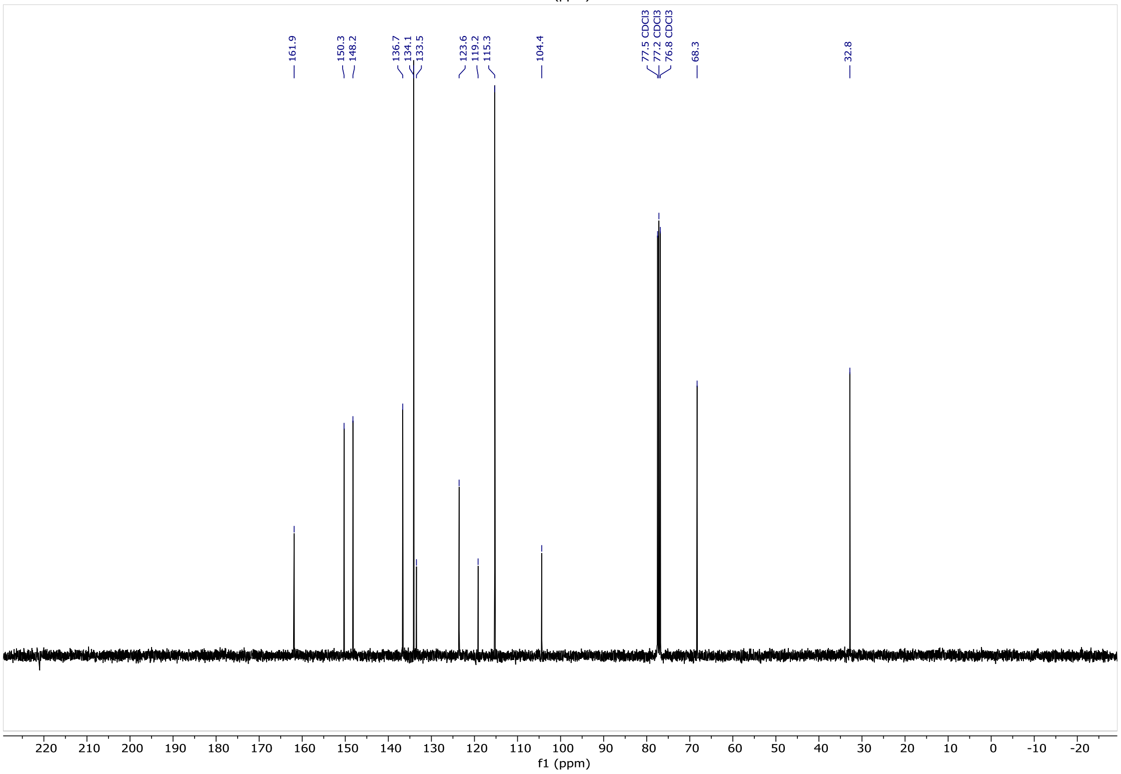

3-(2-(4-cyanophenoxy)ethyl)pyridine 1-oxide (22)

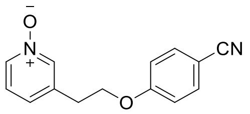

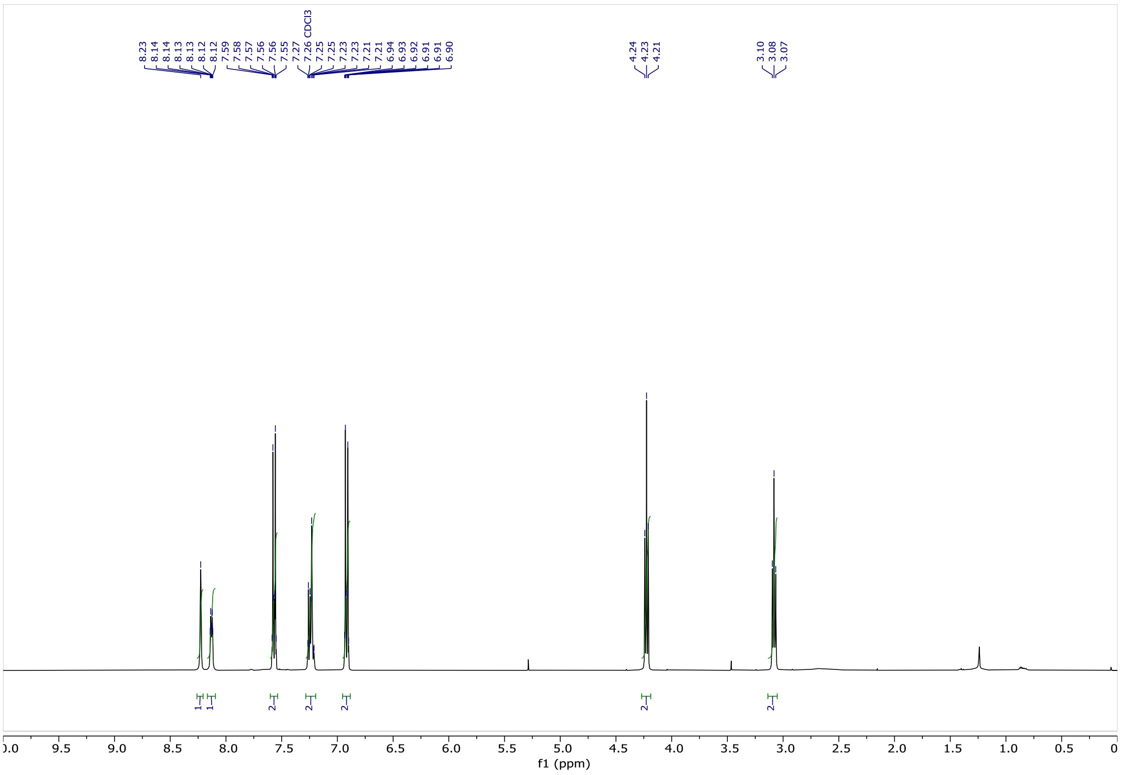

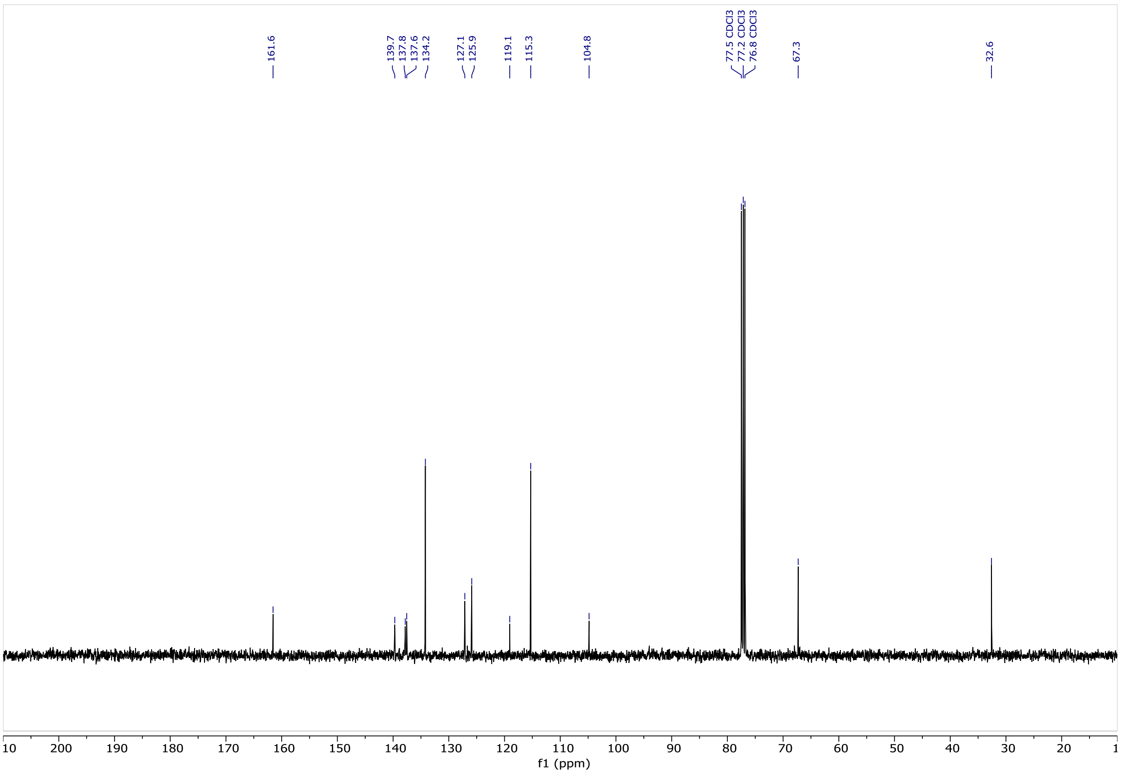

3-(2-(4-nitrophenoxy)ethyl)pyridine (23a)

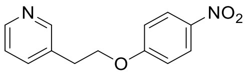

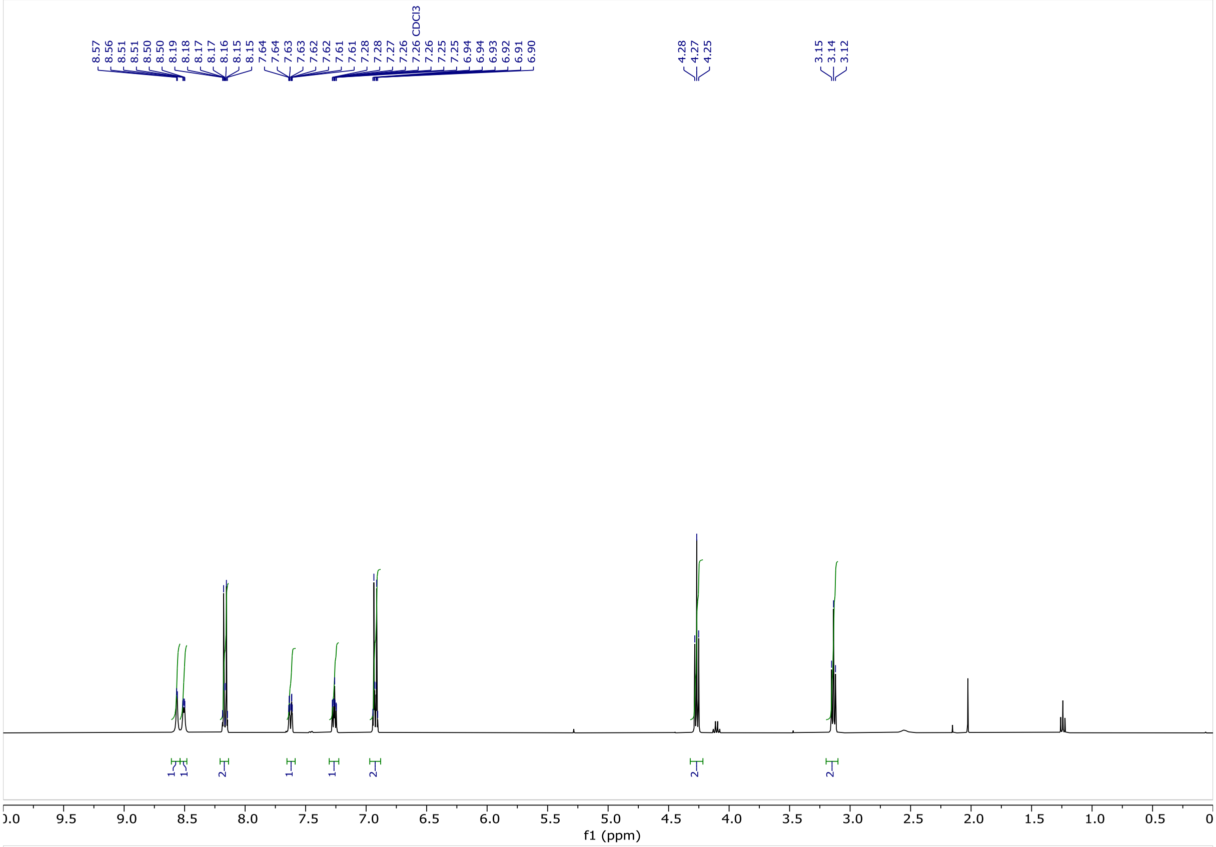

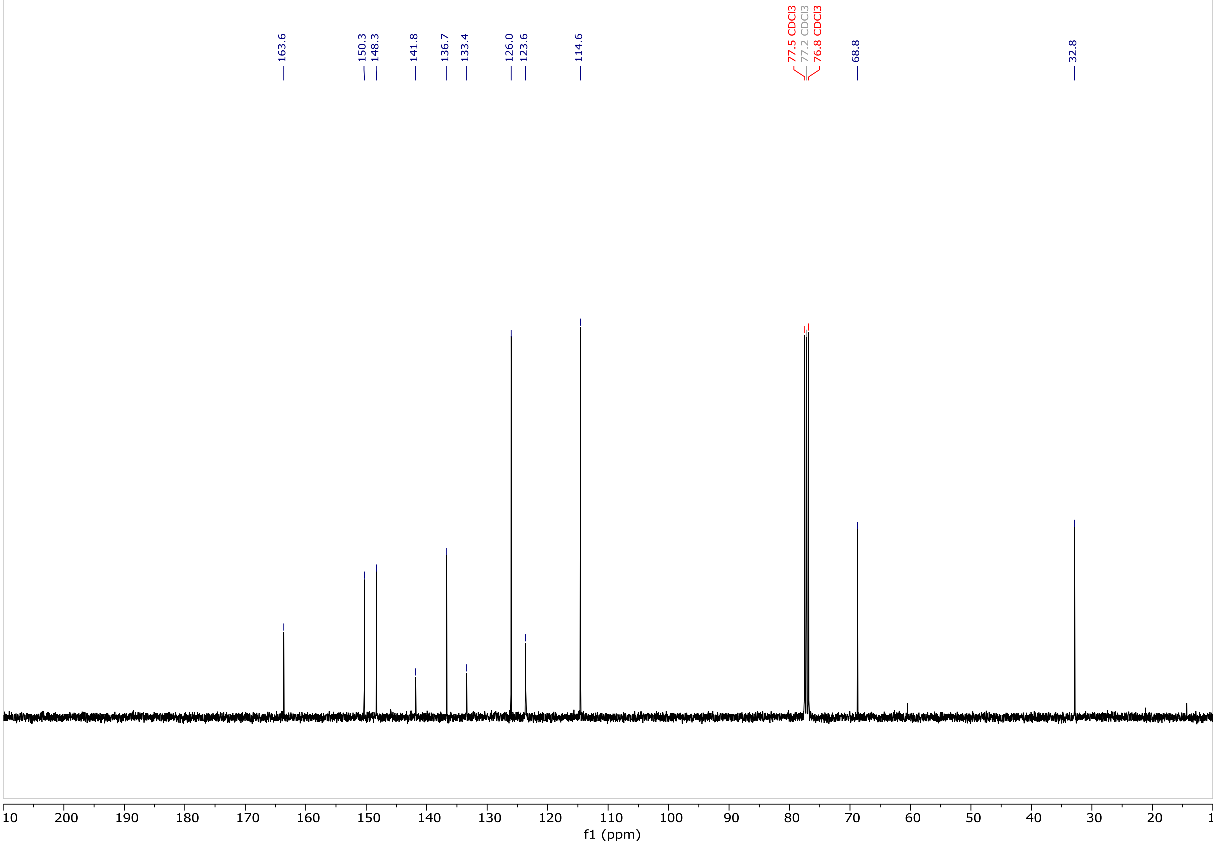

3-(2-(4-nitrophenoxy)ethyl)pyridine 1-oxide (23)

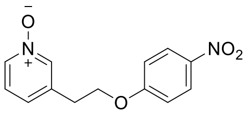

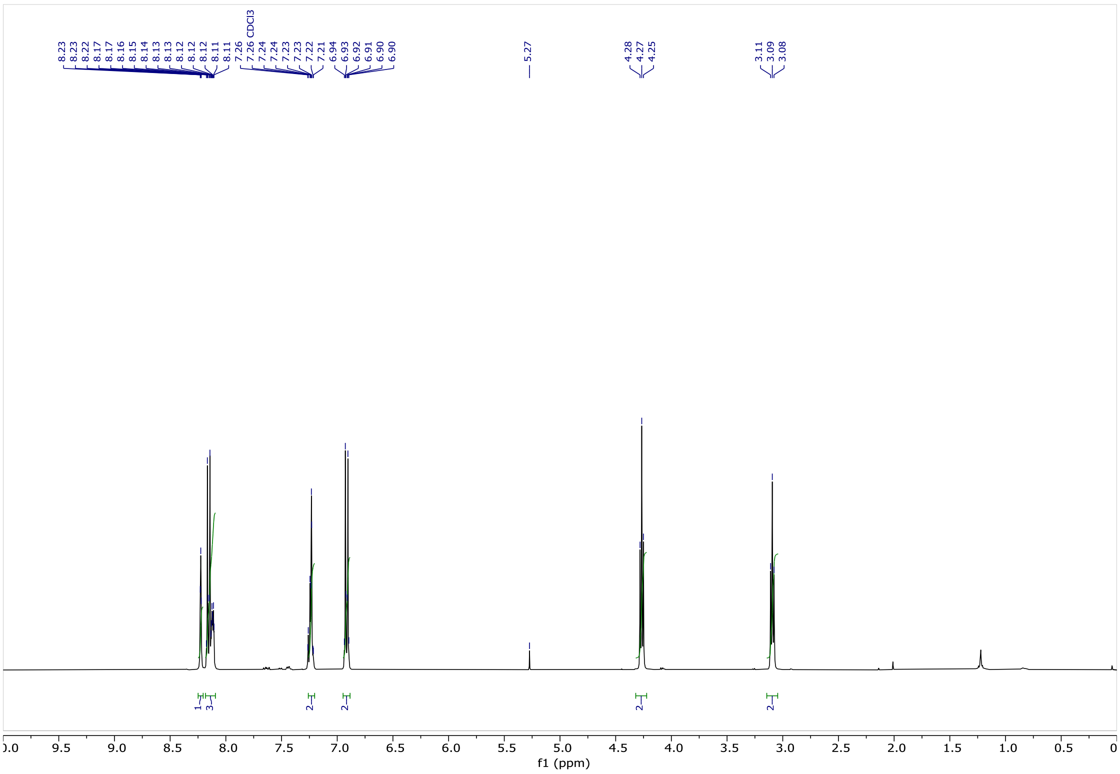

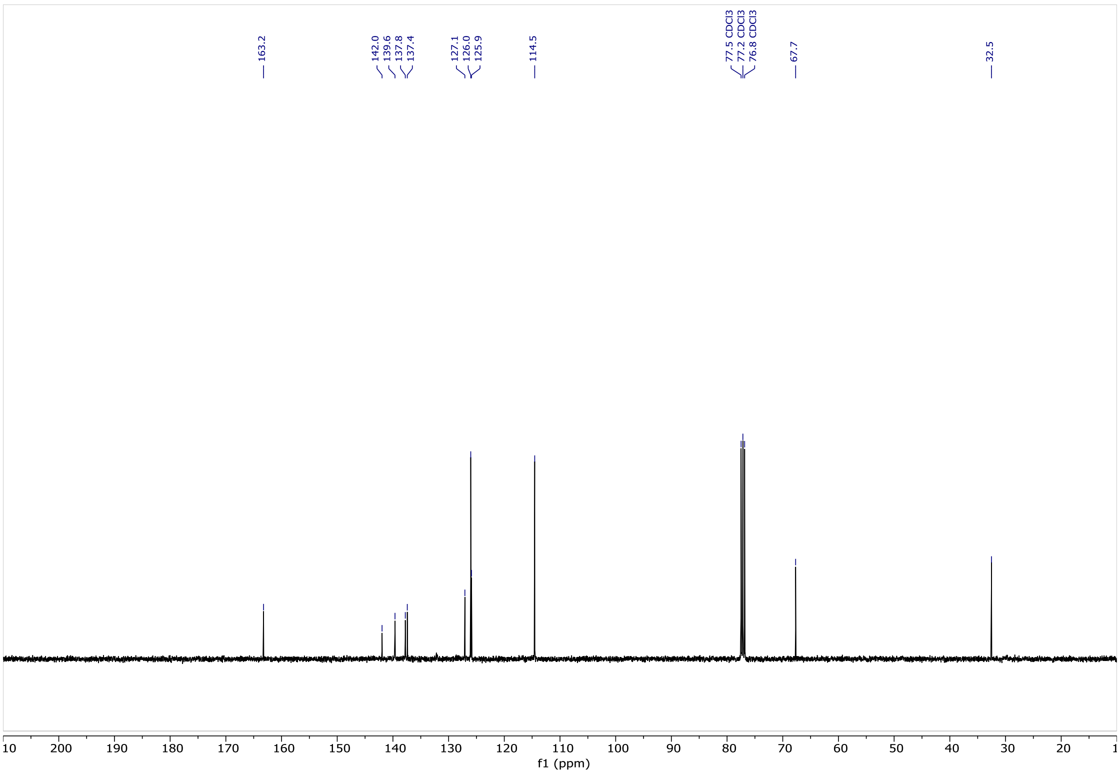

3-(2-(4-chloro-2,3-dimethylphenoxy)ethyl)pyridine (24a)

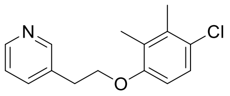

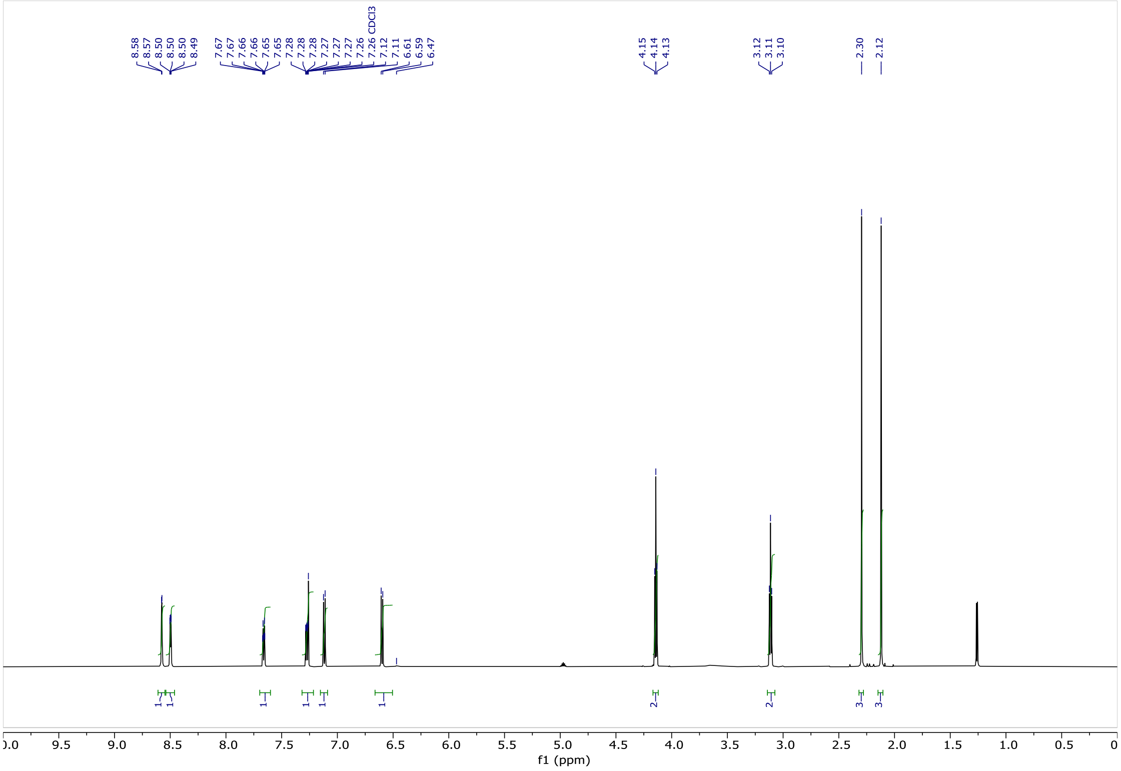

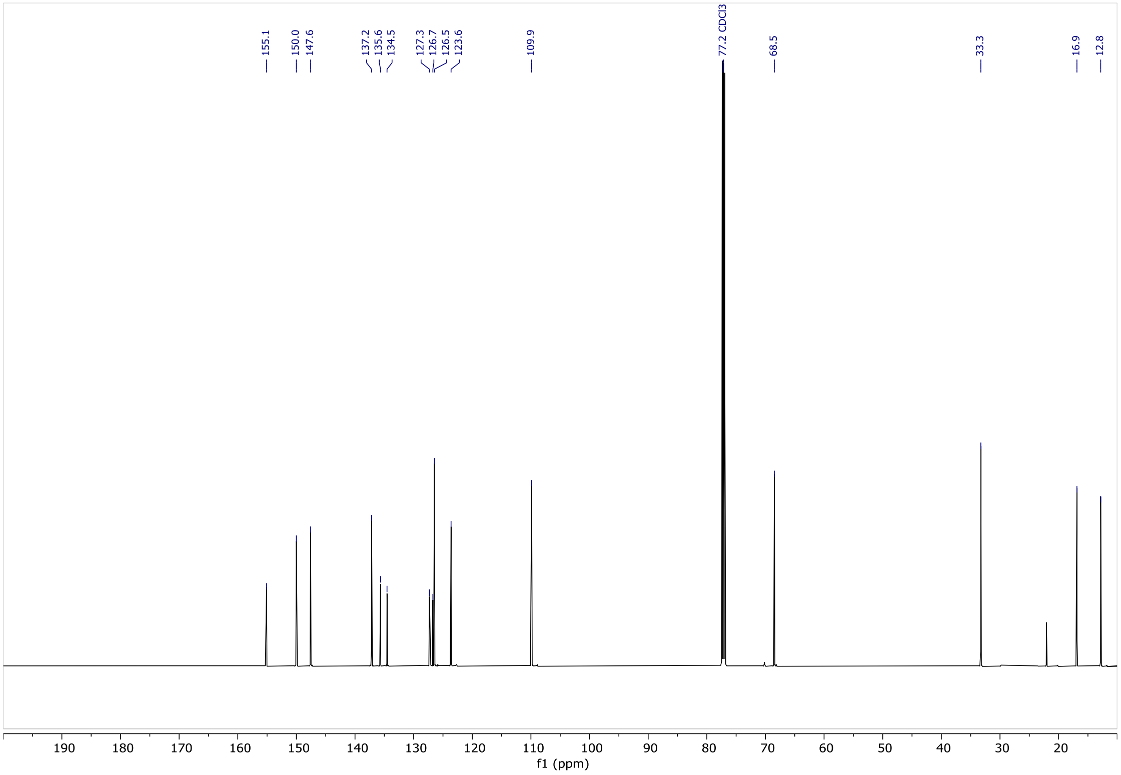

3-(2-(4-chloro-2,3-dimethylphenoxy)ethyl)pyridine 1-oxide (24)

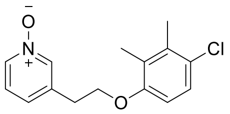

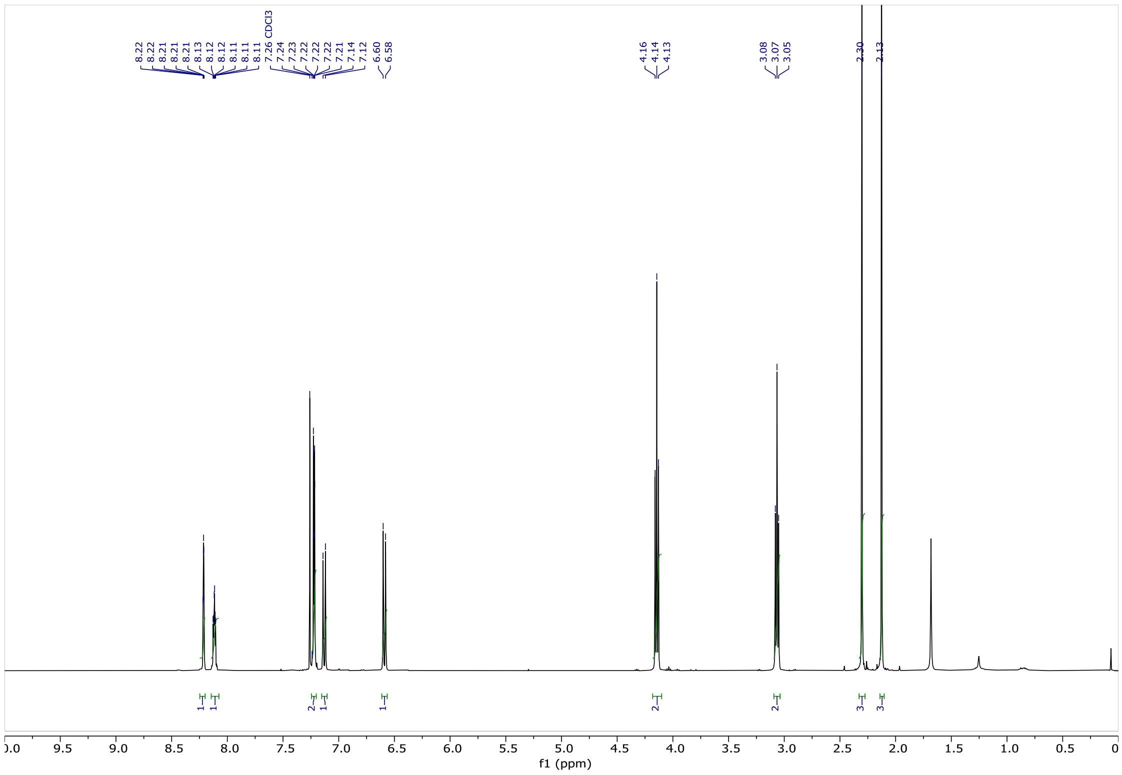

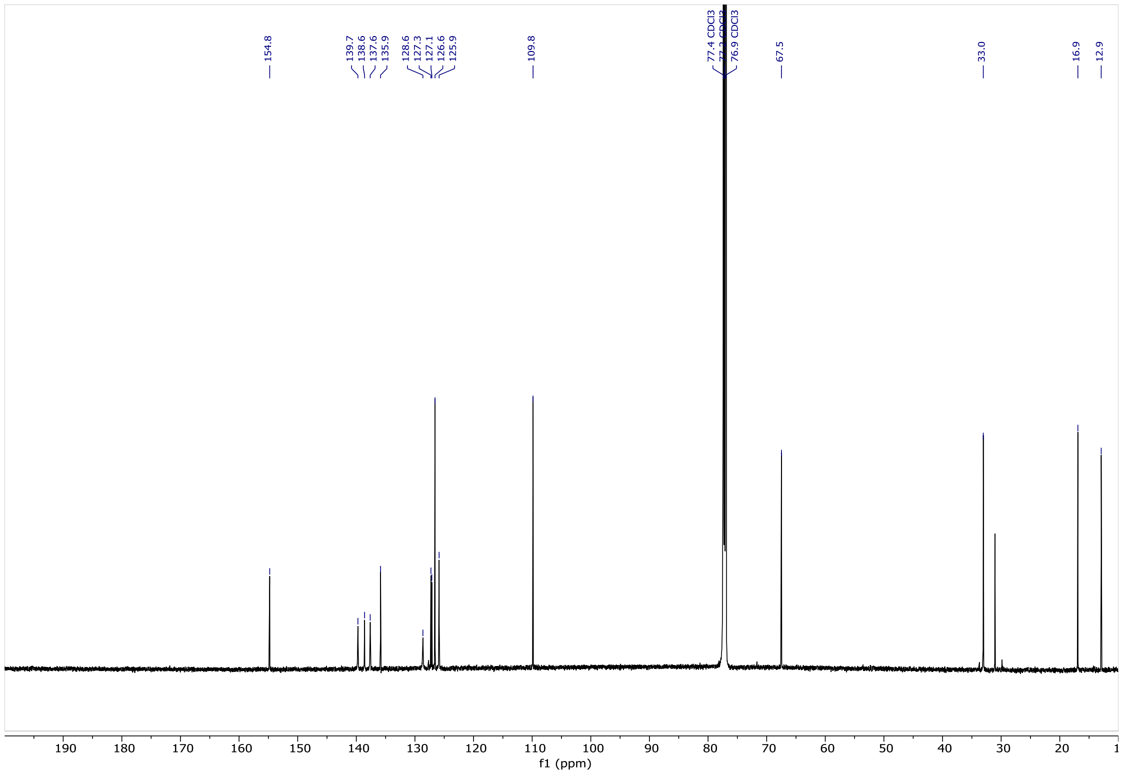

3-(2-(2,3-dichlorophenoxy)ethyl)pyridine (25a)

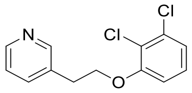

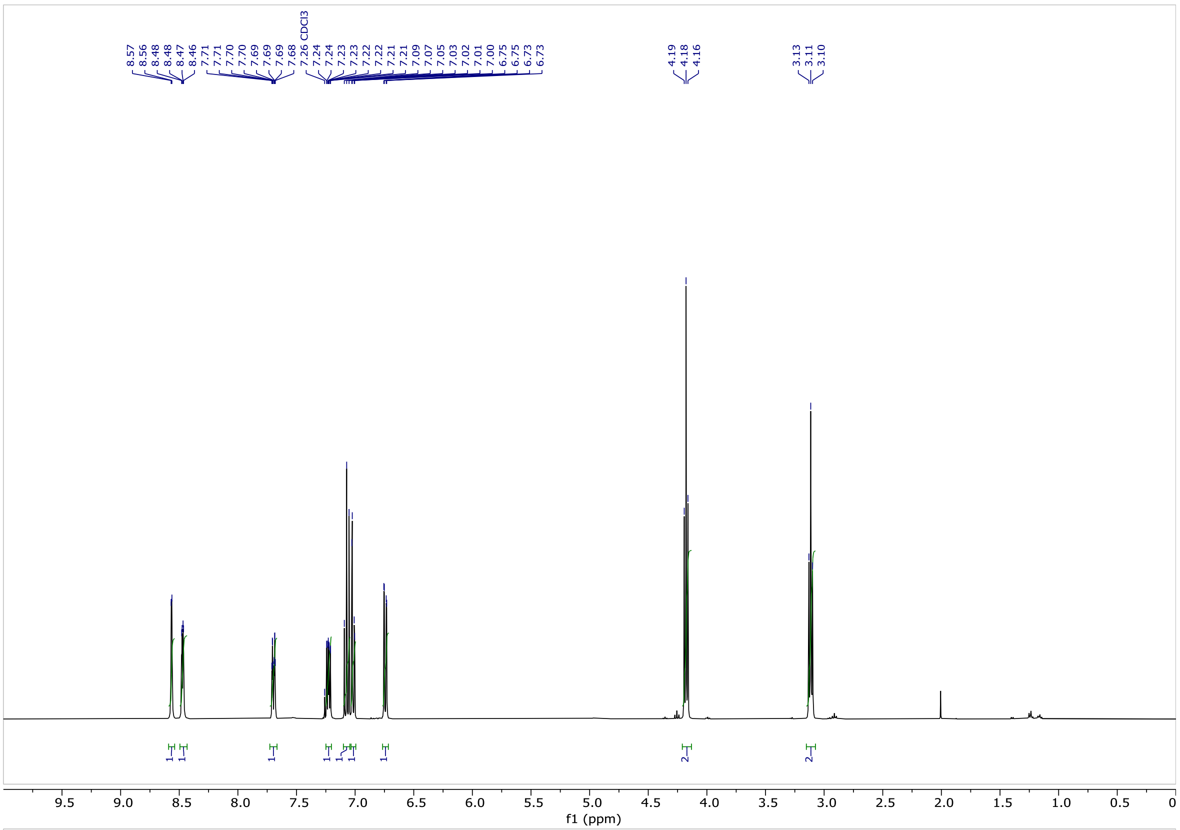

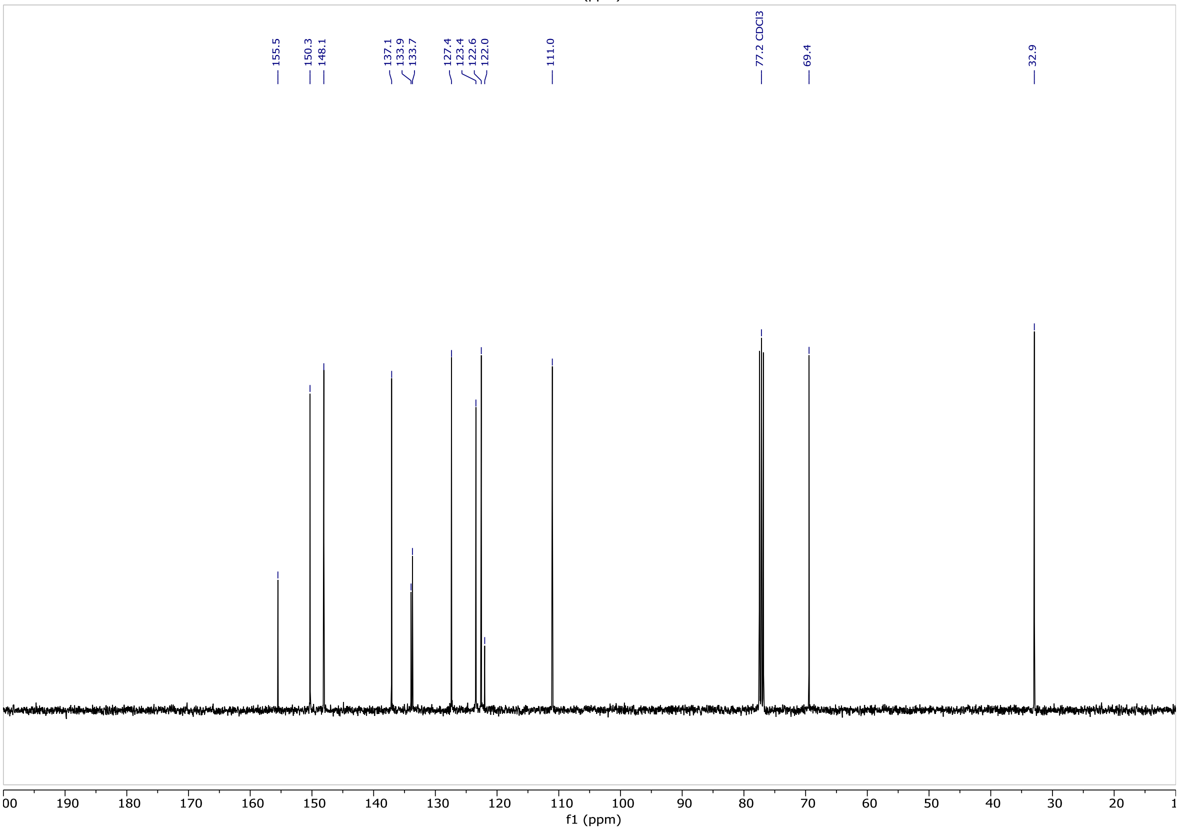

3-(2-(2,3-dichlorophenoxy)ethyl)pyridine 1-oxide (25)

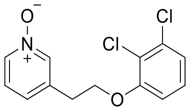

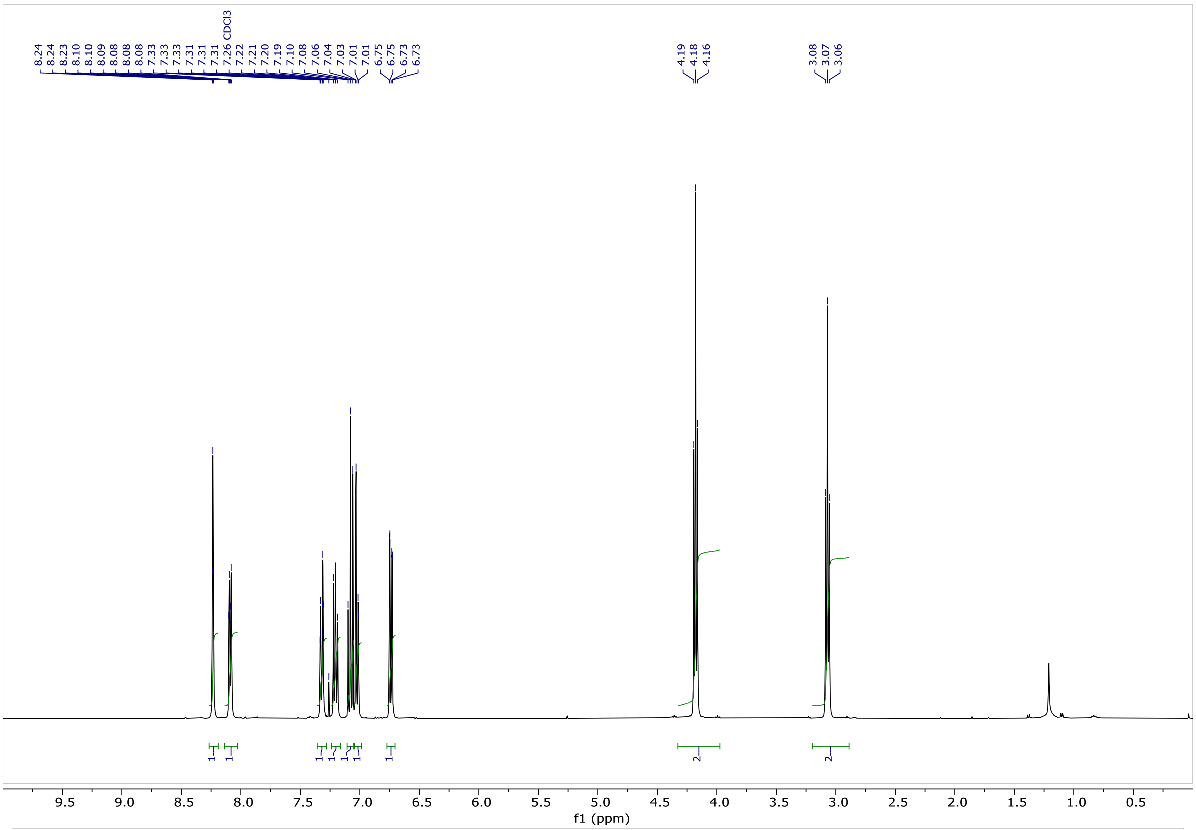

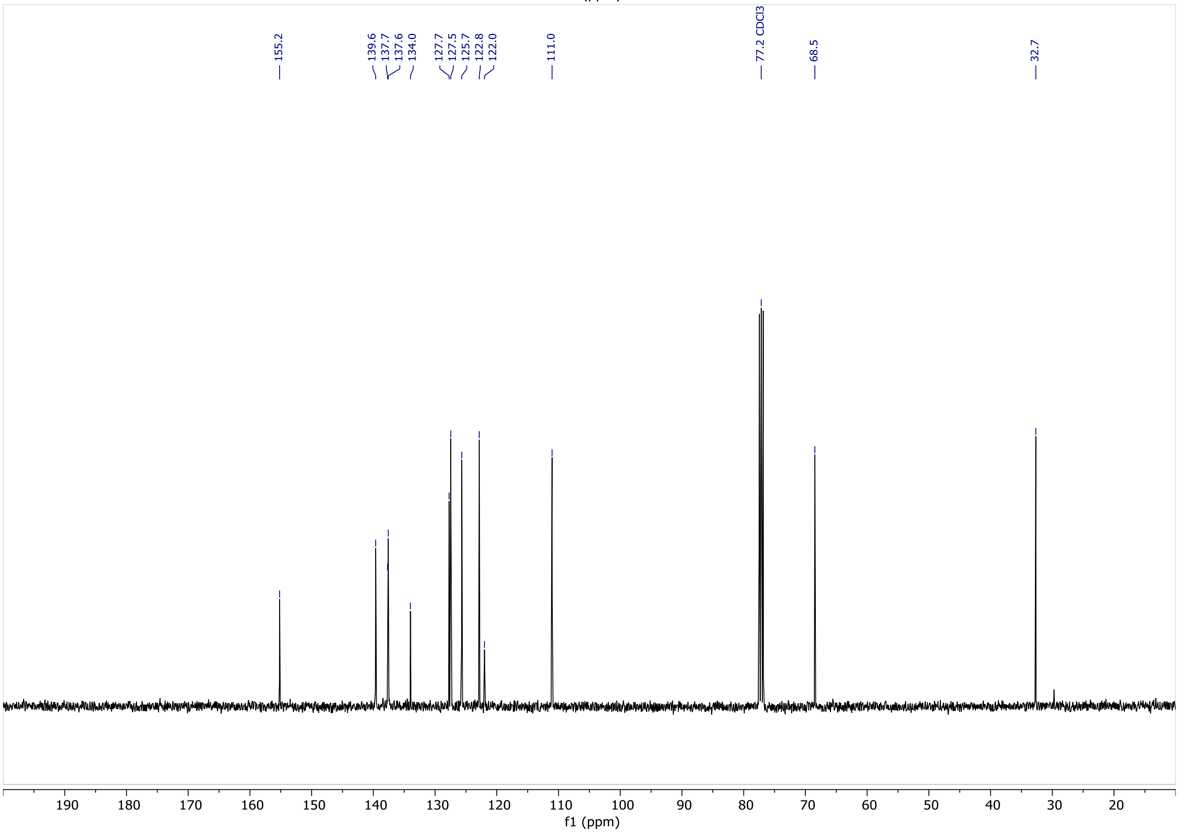

3-(2-(4-chloro-2-iodophenoxy)ethyl)pyridine (26a)

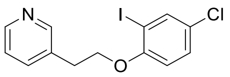

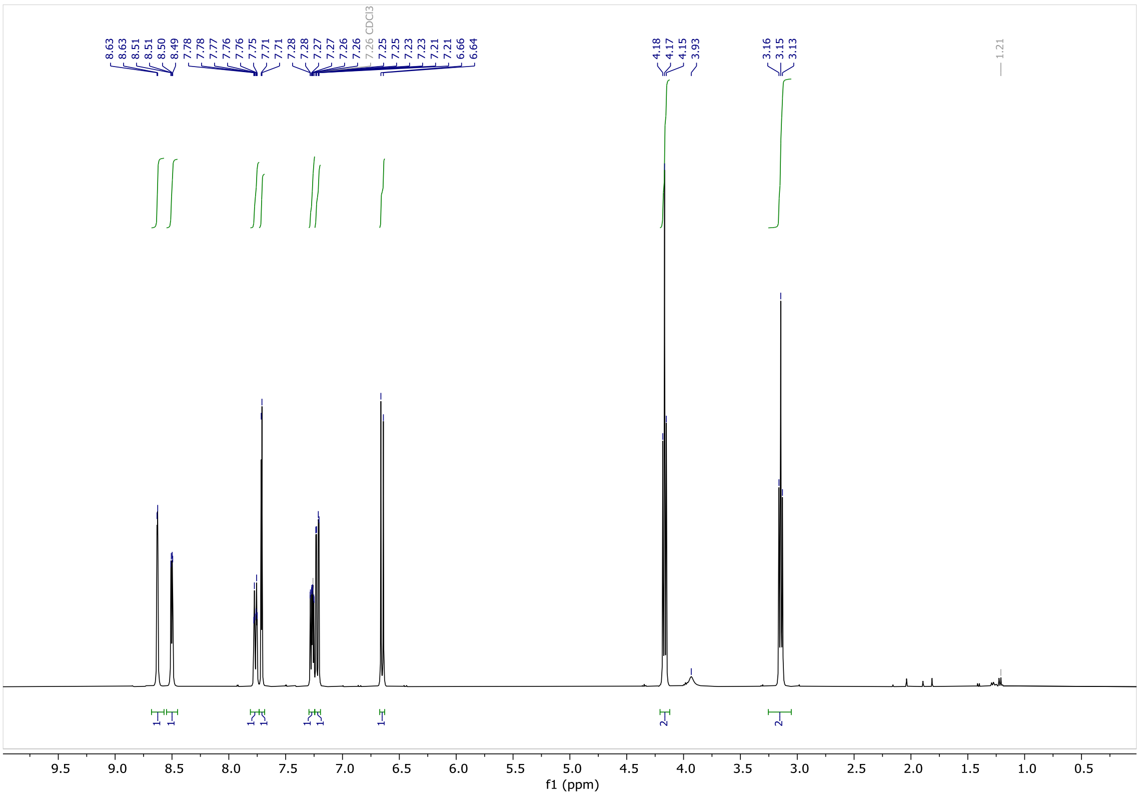

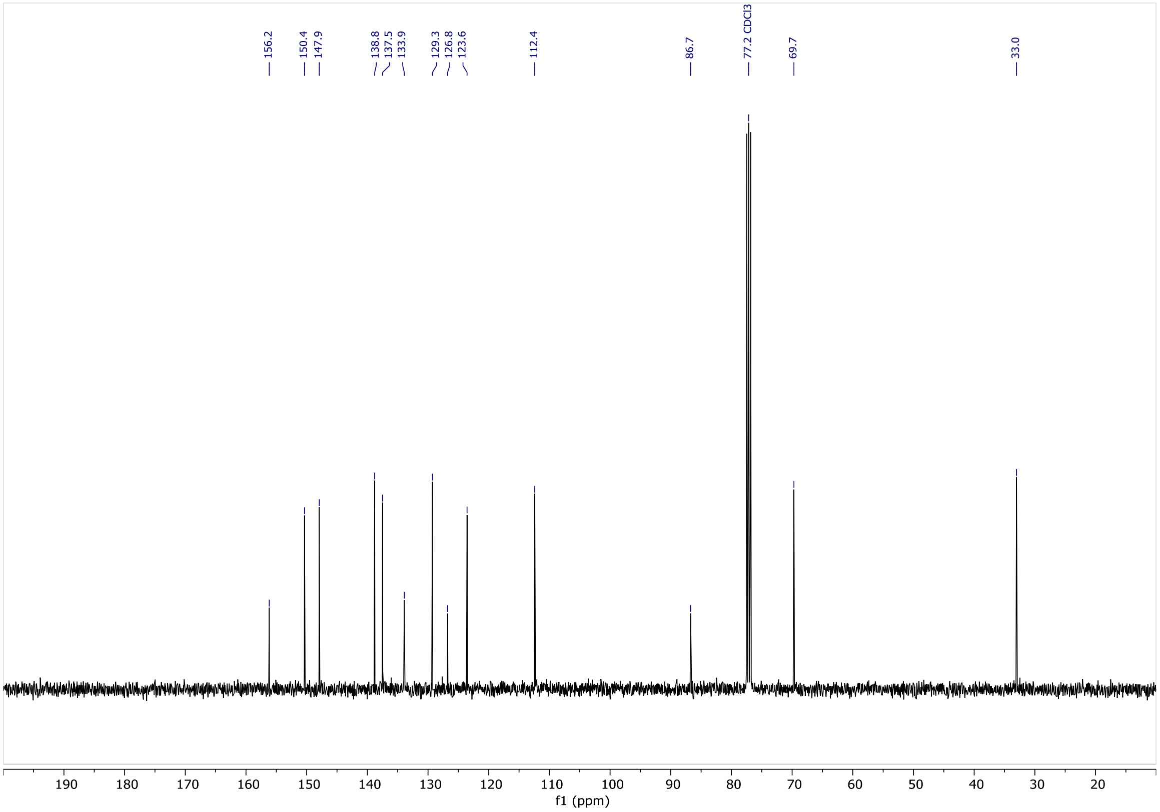

3-(2-(4-chloro-2-iodophenoxy)ethyl)pyridine 1-oxide (26)

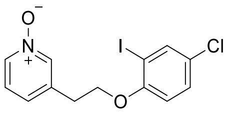

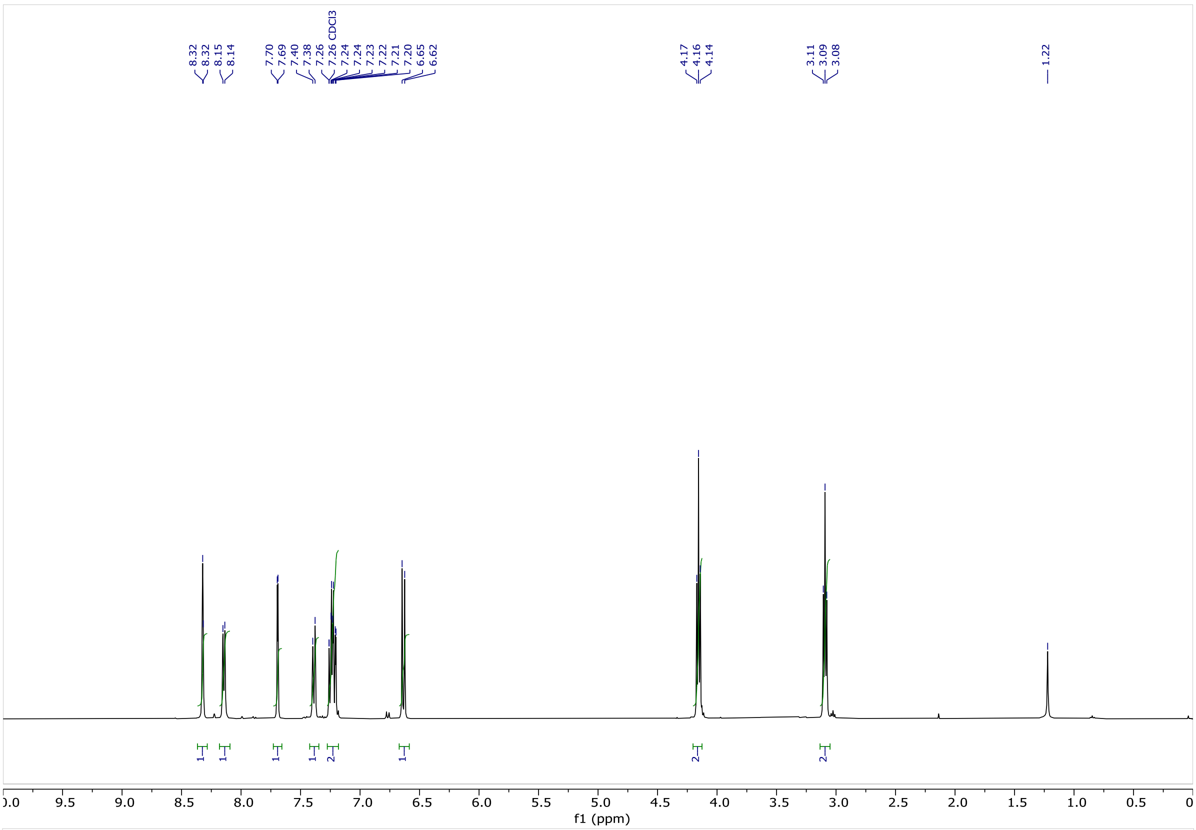

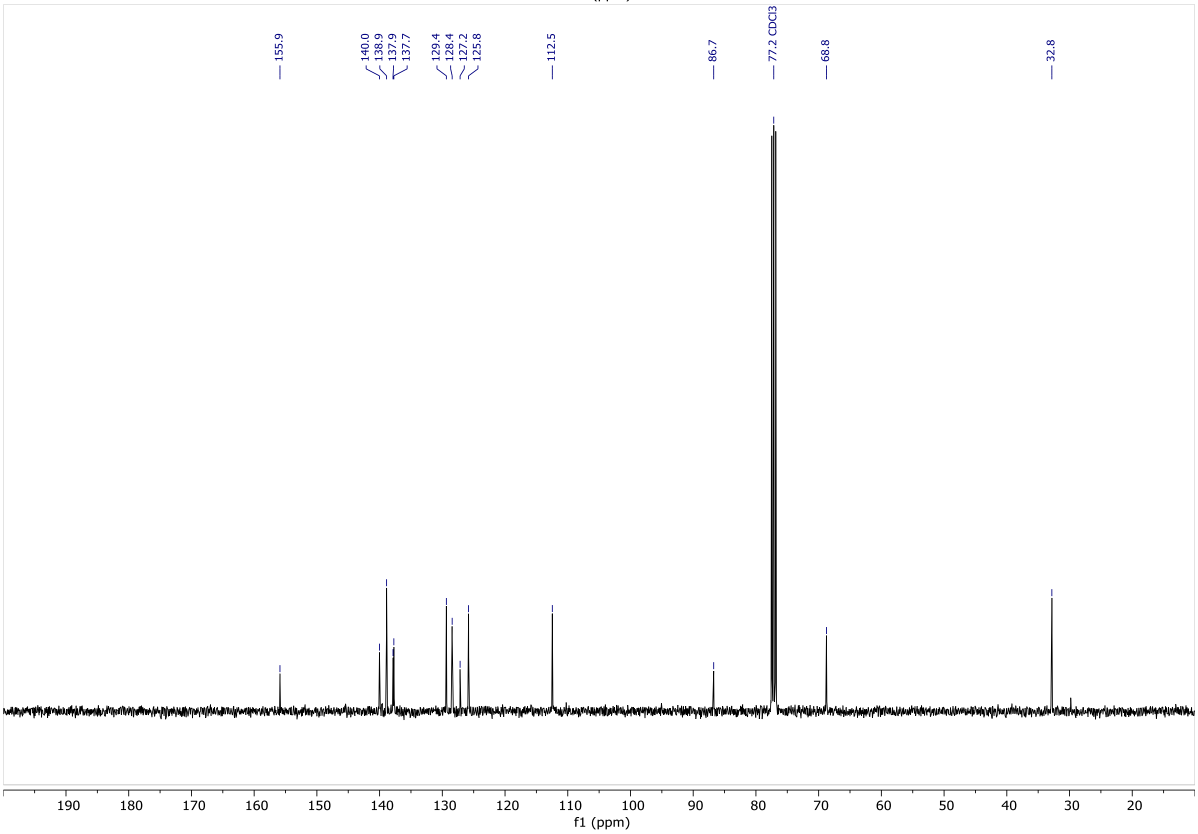

3-(2-(4-chloro-2-cyclohexylphenoxy)ethyl)pyridine 1-oxide (27)

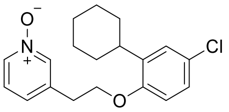

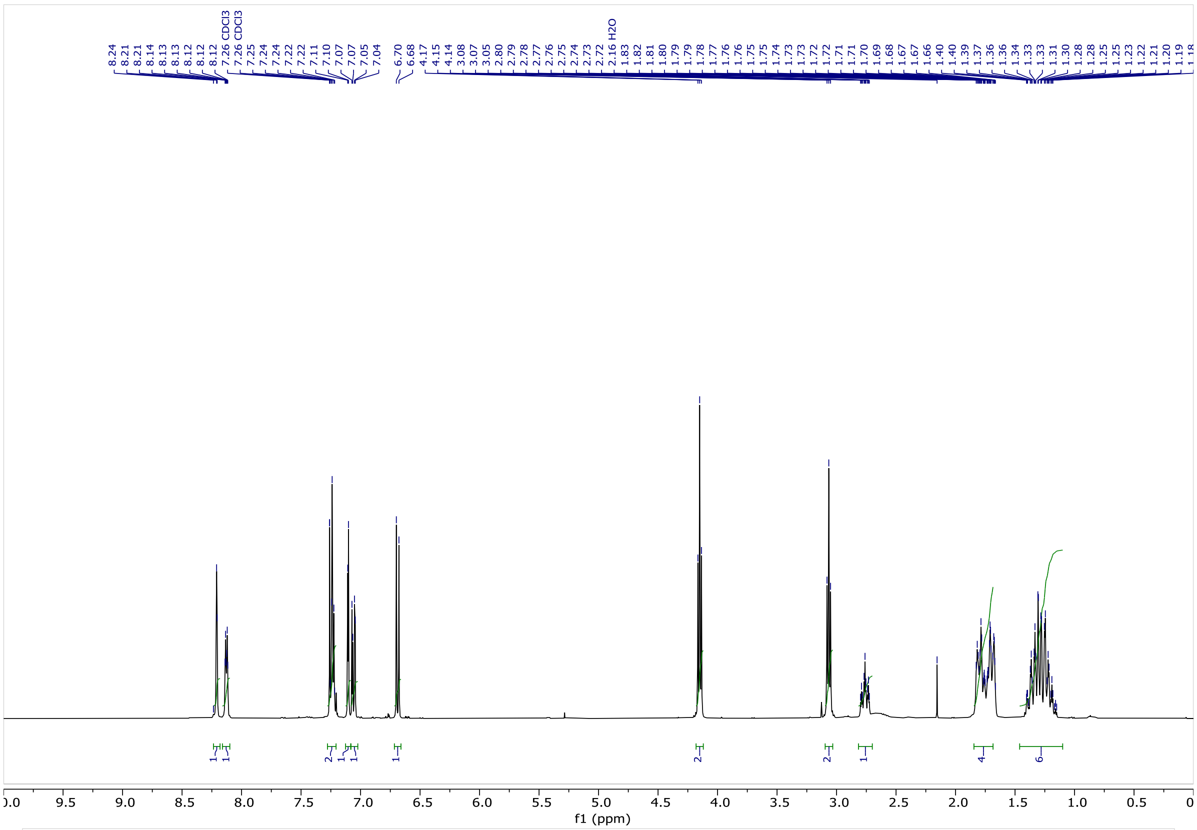

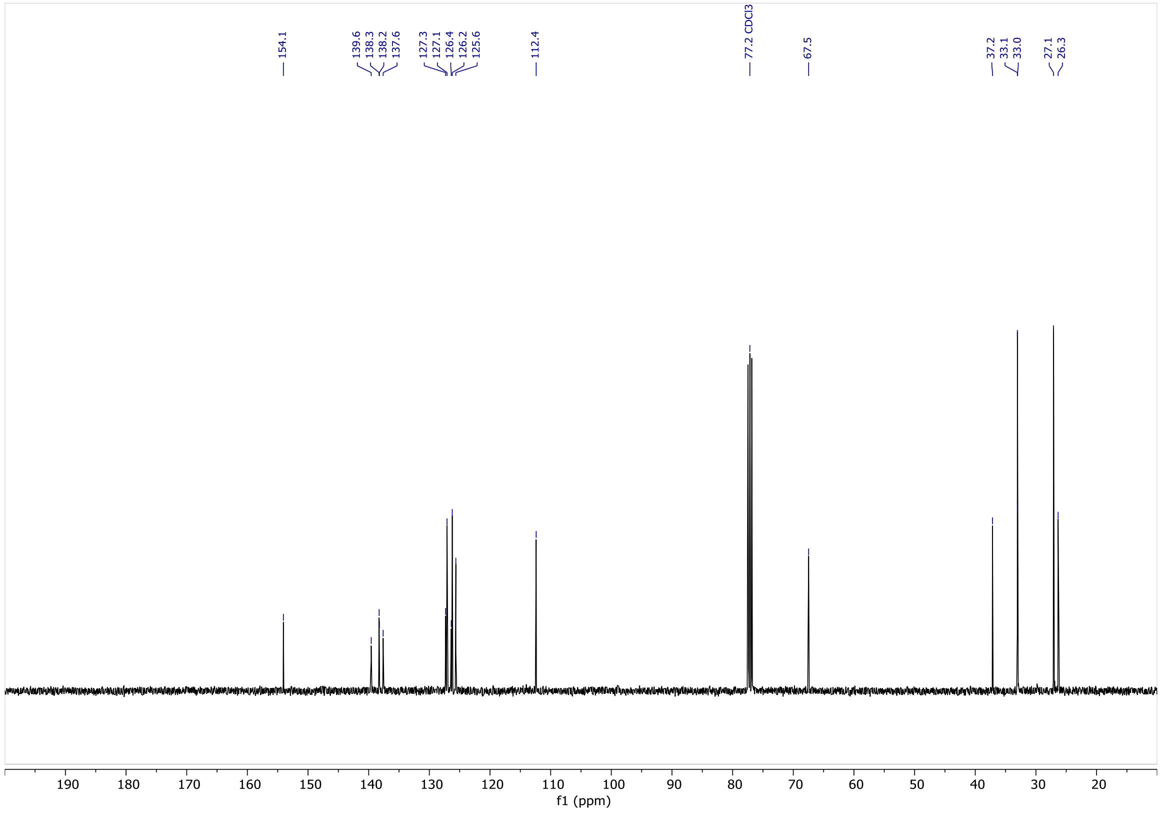

3-(2-((5-chloro-[1,1'-biphenyl]-2-yl)oxy)ethyl)pyridine (28a)

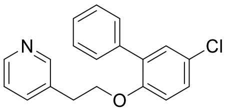

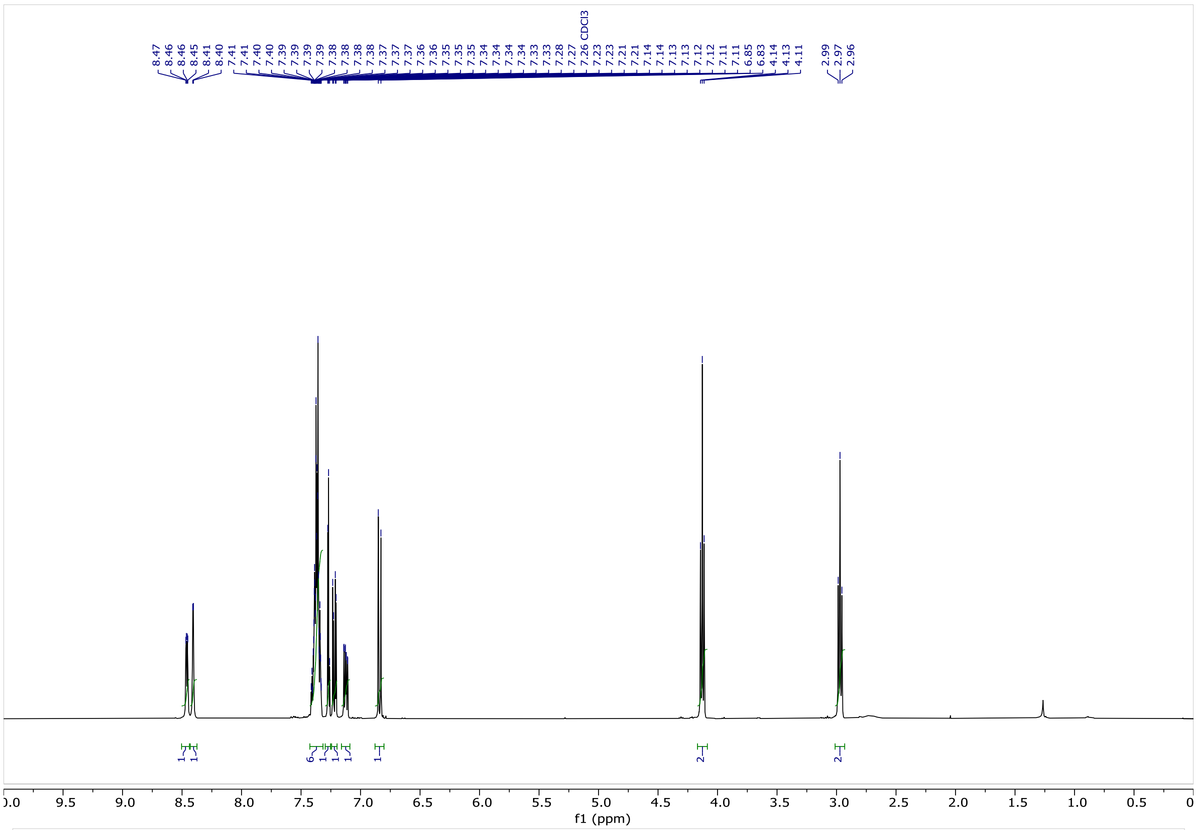

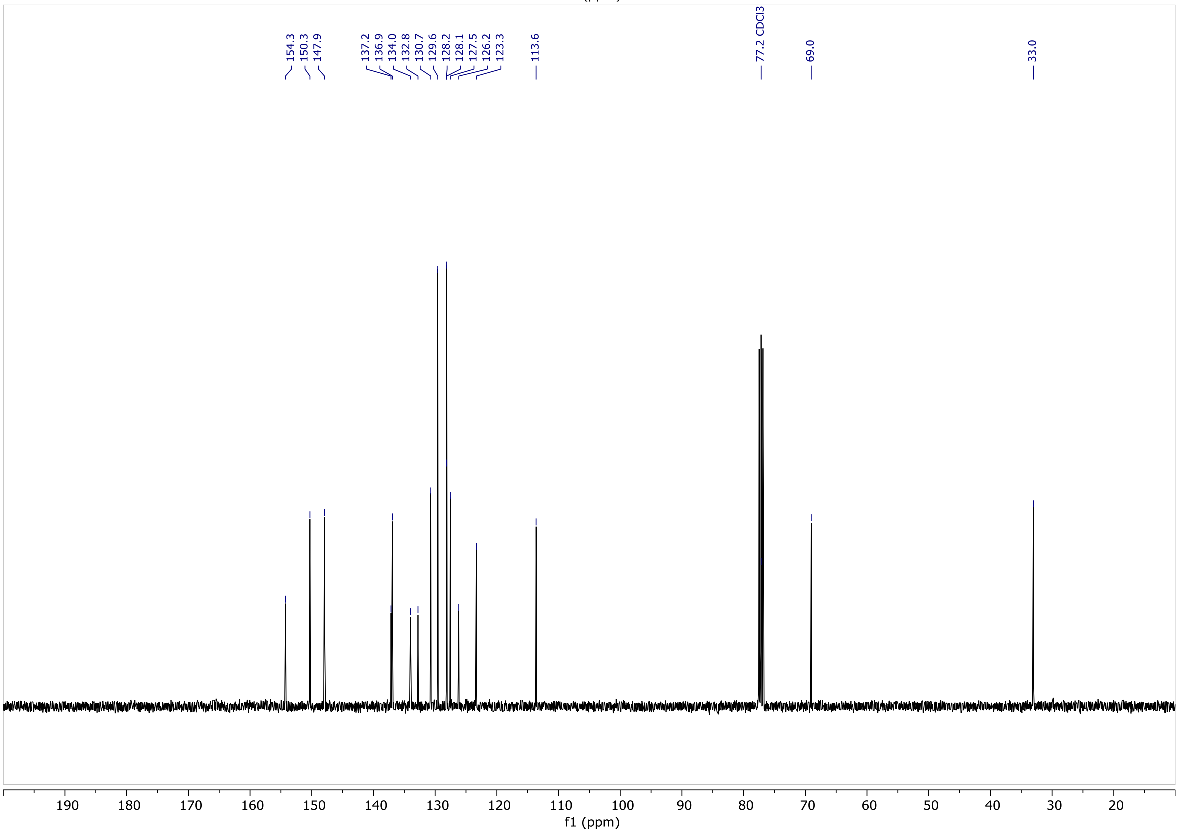

3-(2-((5-chloro-[1,1'-biphenyl]-2-yl)oxy)ethyl)pyridine 1-oxide (28)

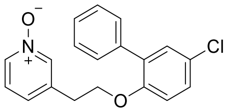

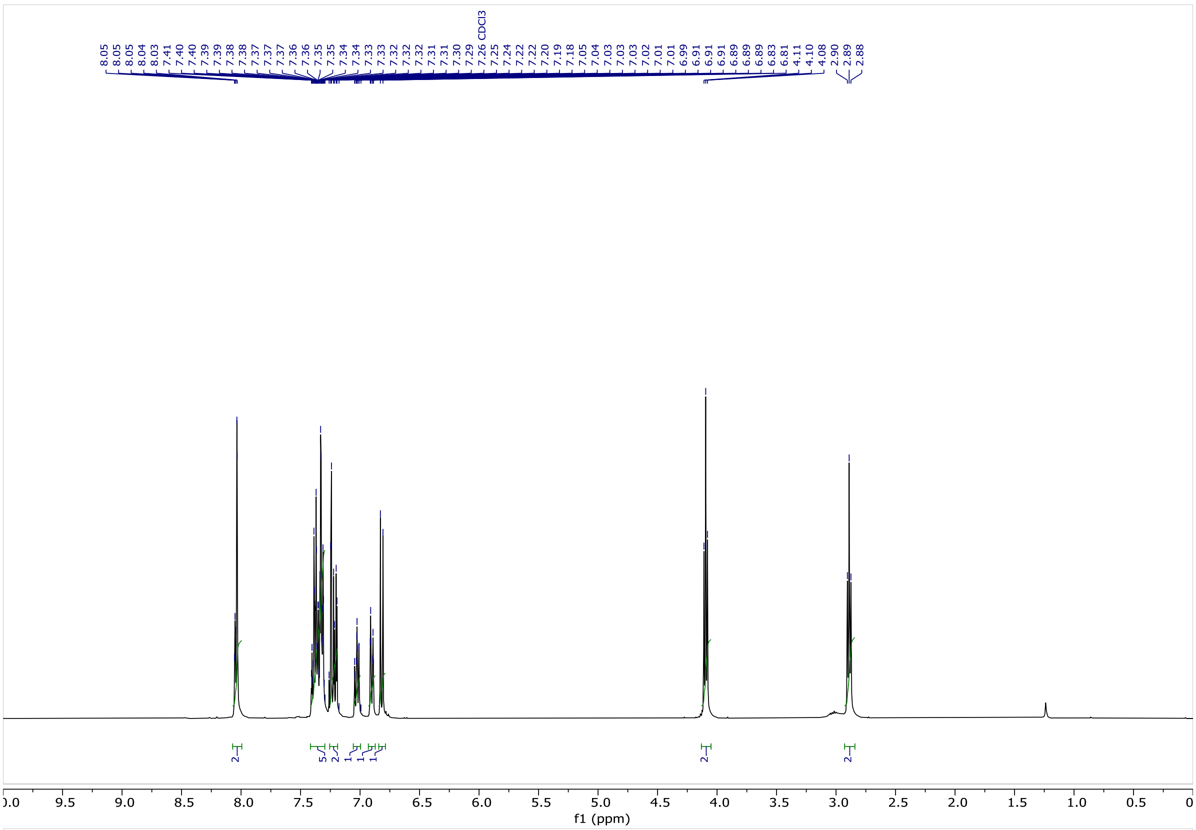

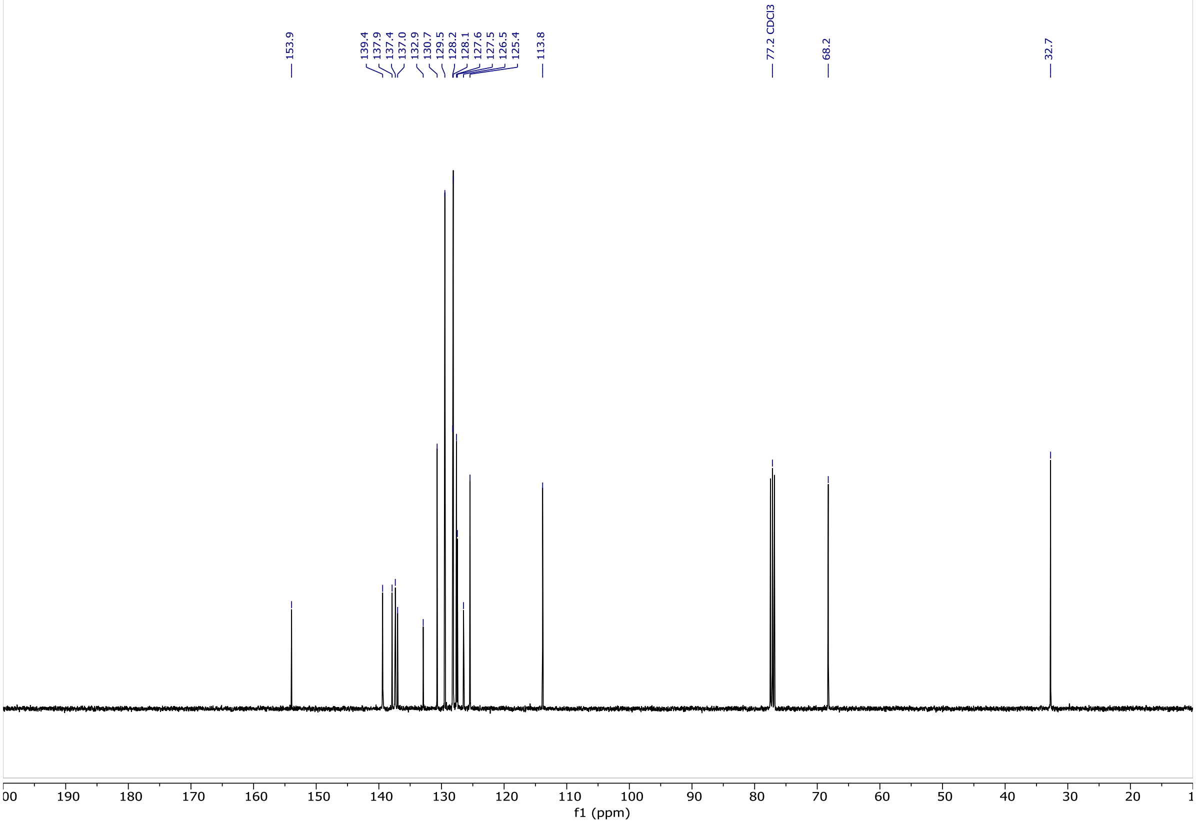

3-(2-(2,3,4-trichlorophenoxy)ethyl)pyridine (29a)

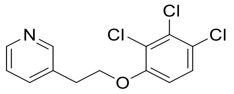

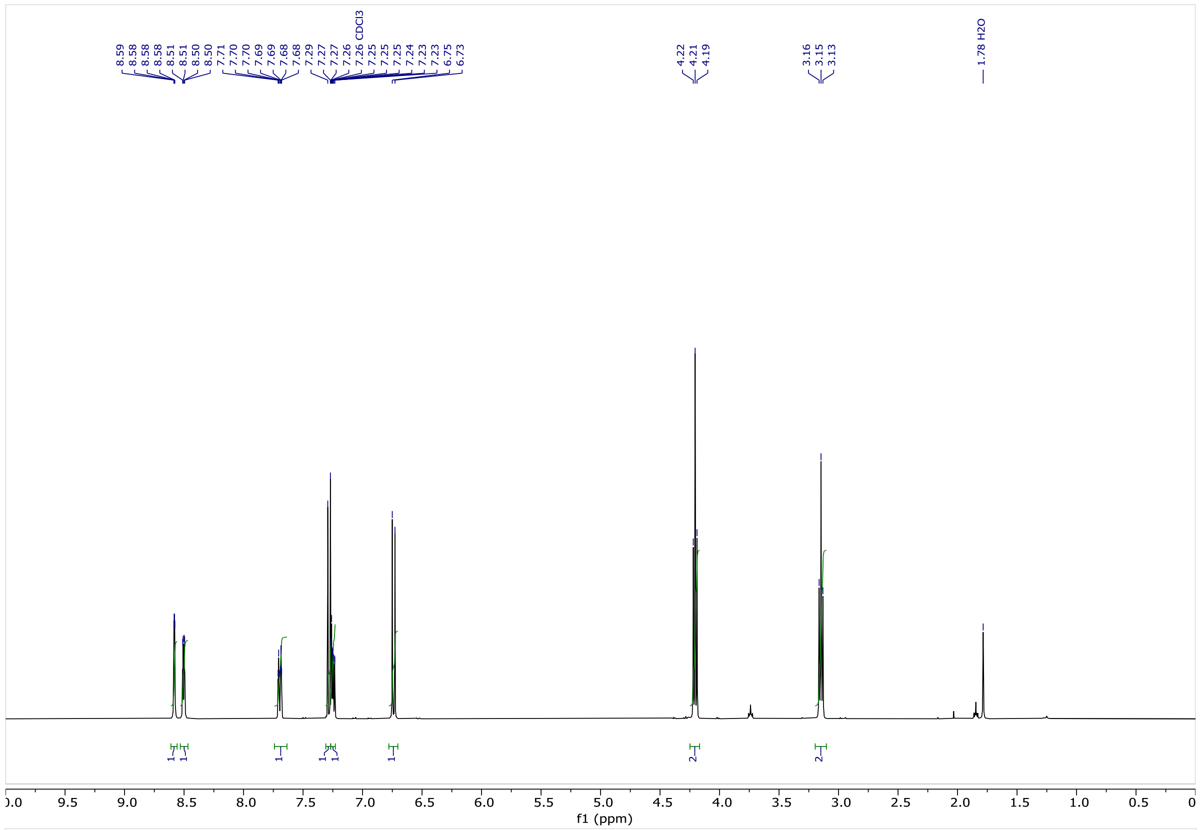

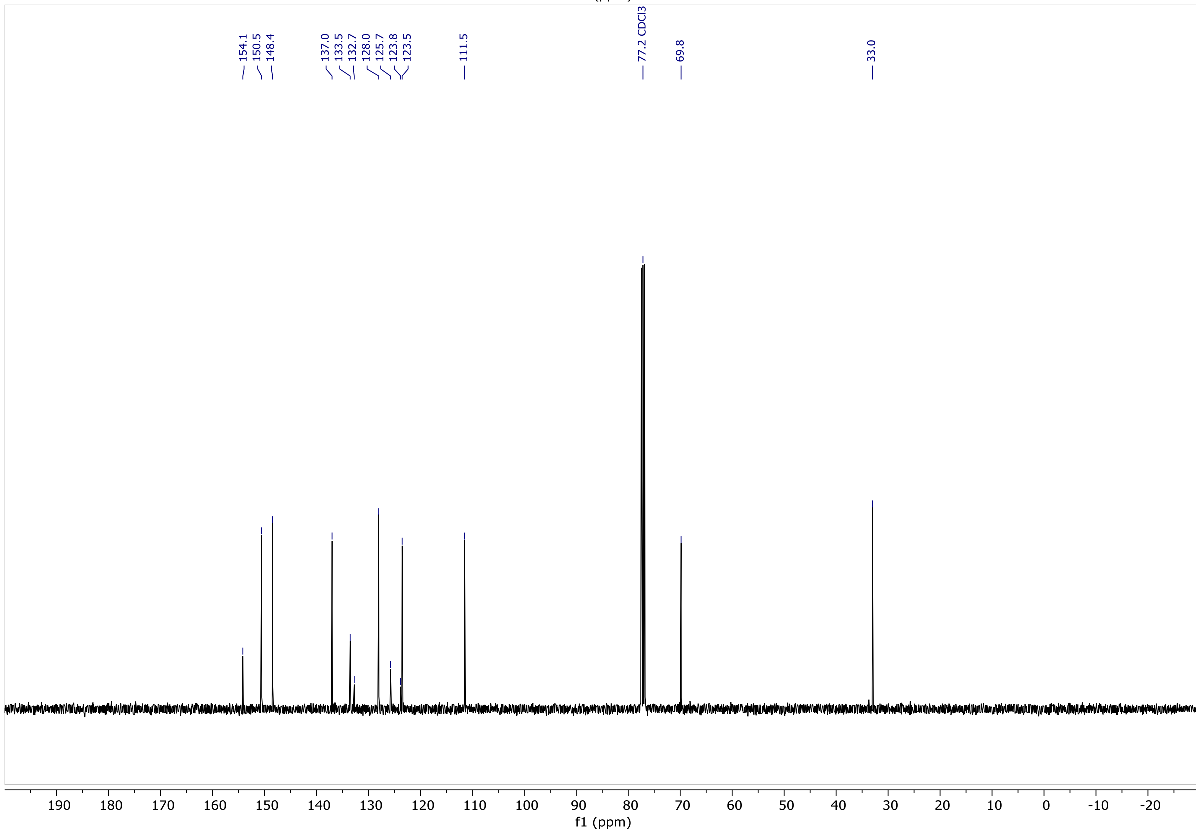

3-(2-(2,3,4-trichlorophenoxy)ethyl)pyridine 1-oxide (29)

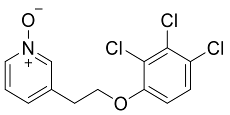

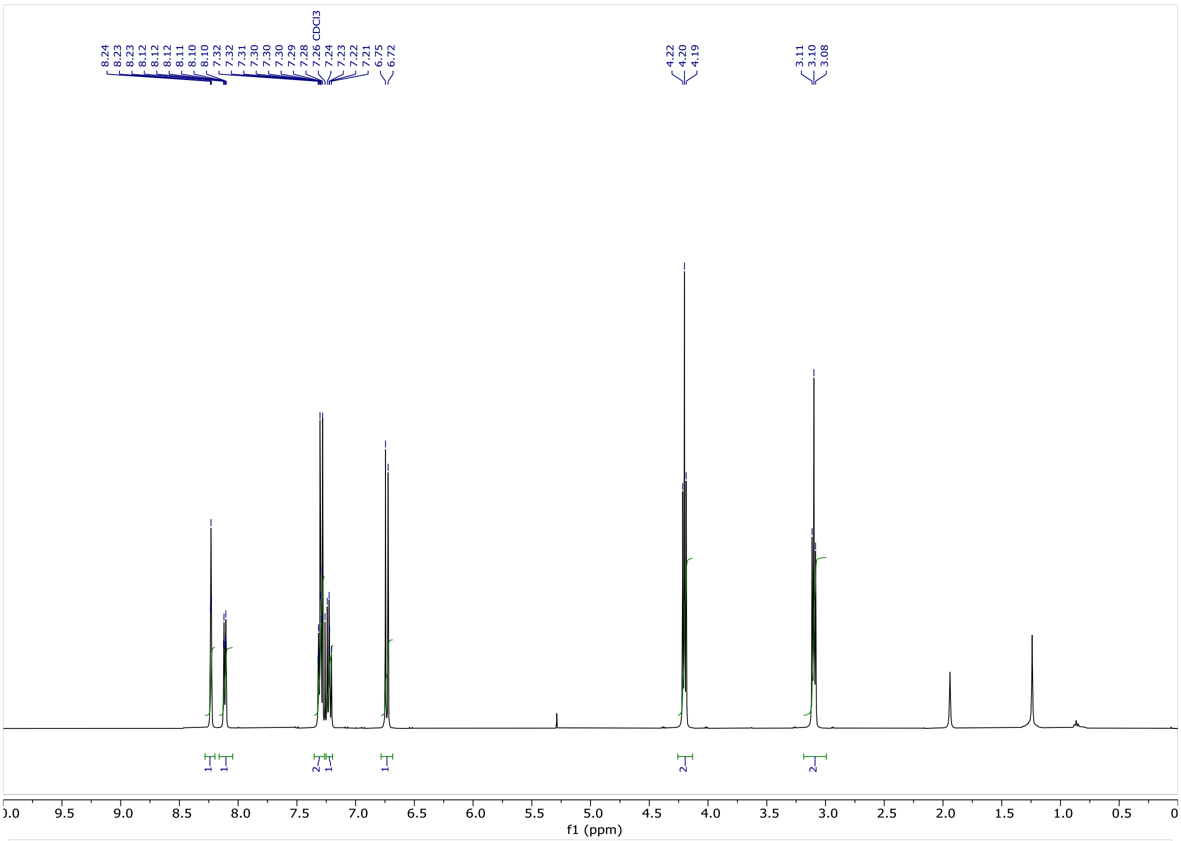

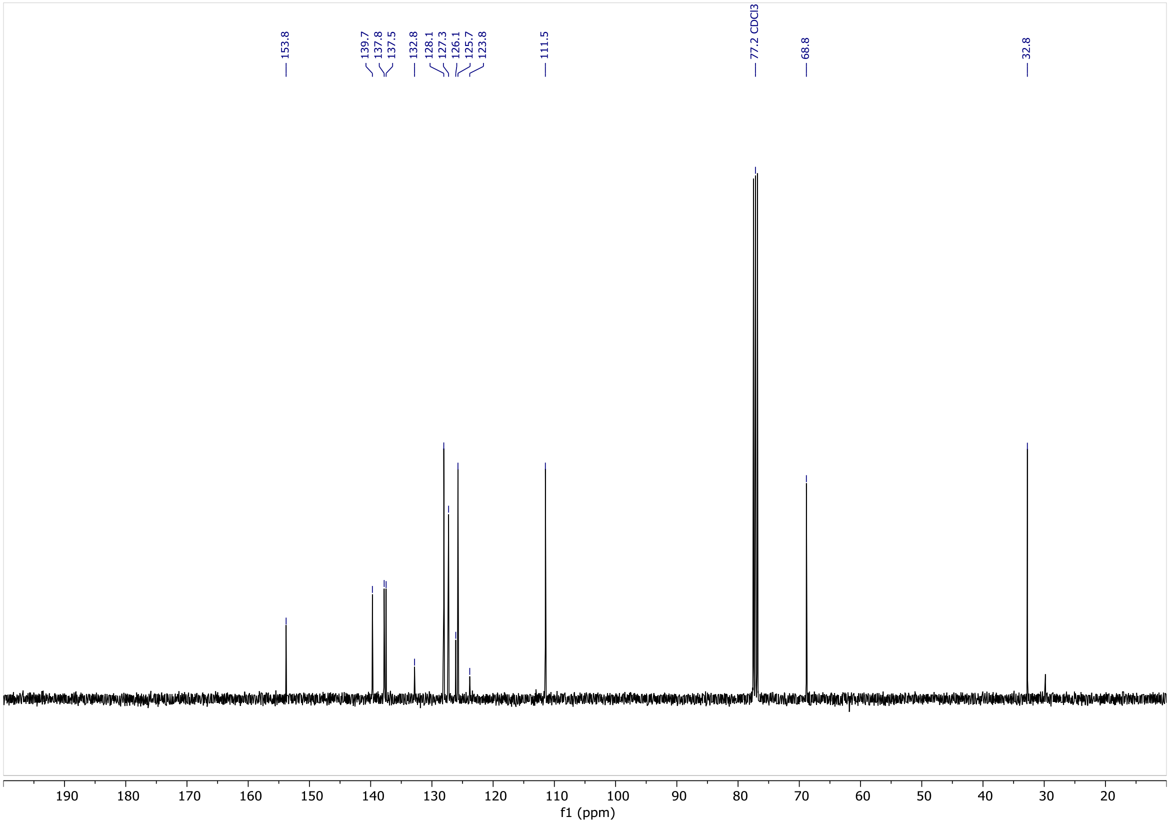

3-(2-(4-chloro-3,5-dimethylphenoxy)ethyl)pyridine (30a)

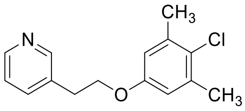

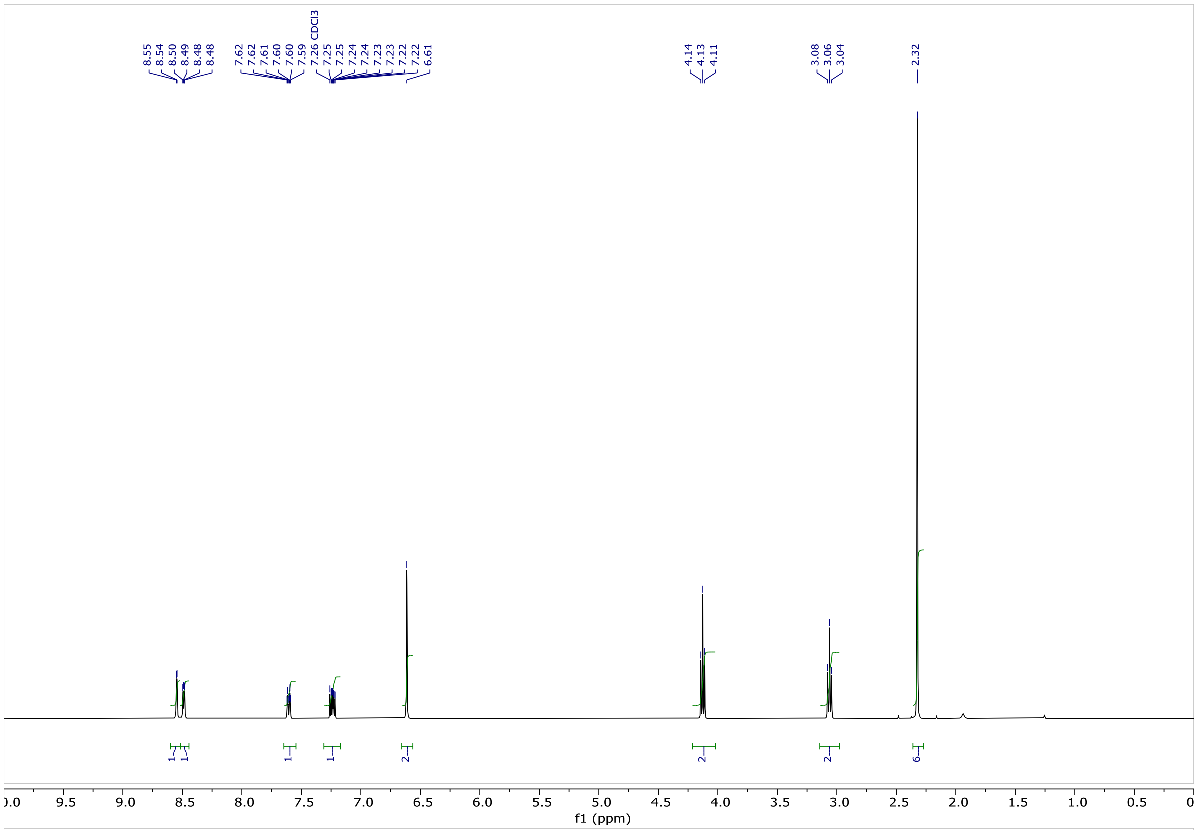

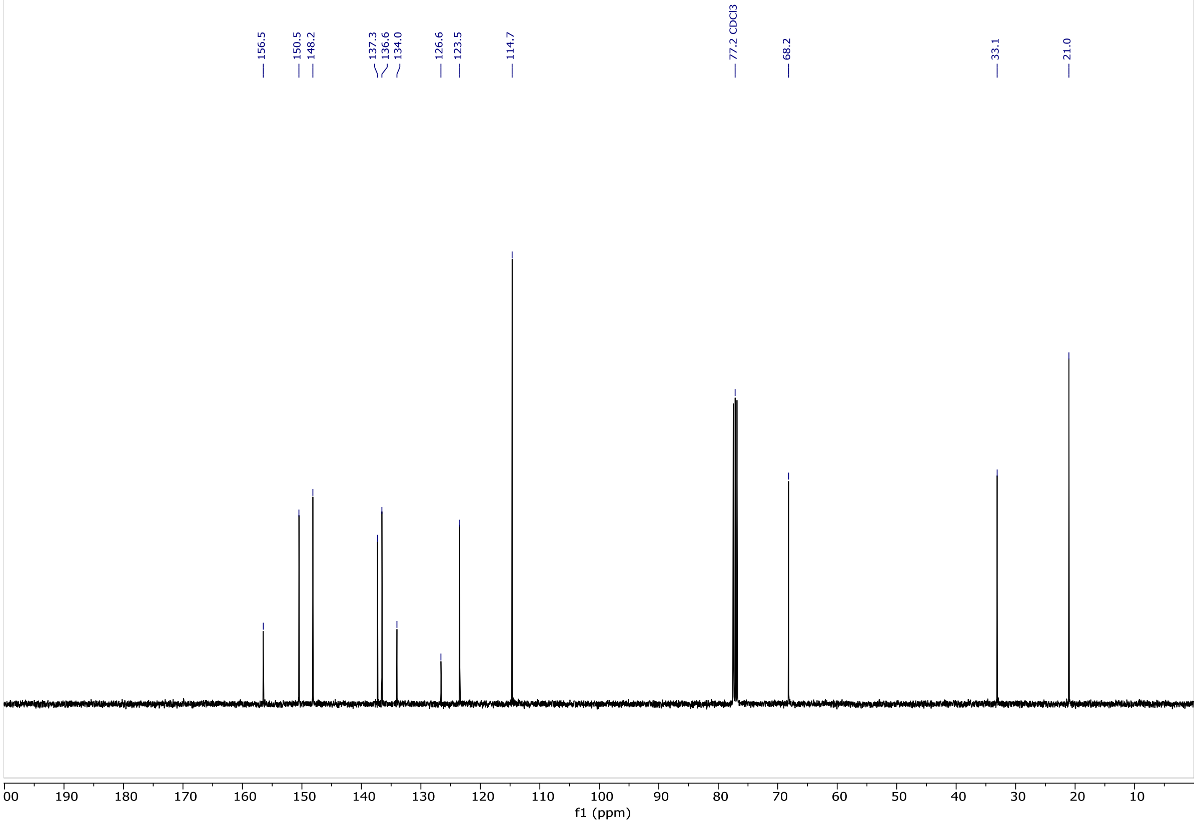

3-(2-(4-chloro-3,5-dimethylphenoxy)ethyl)pyridine 1-oxide (30)

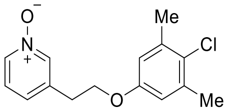

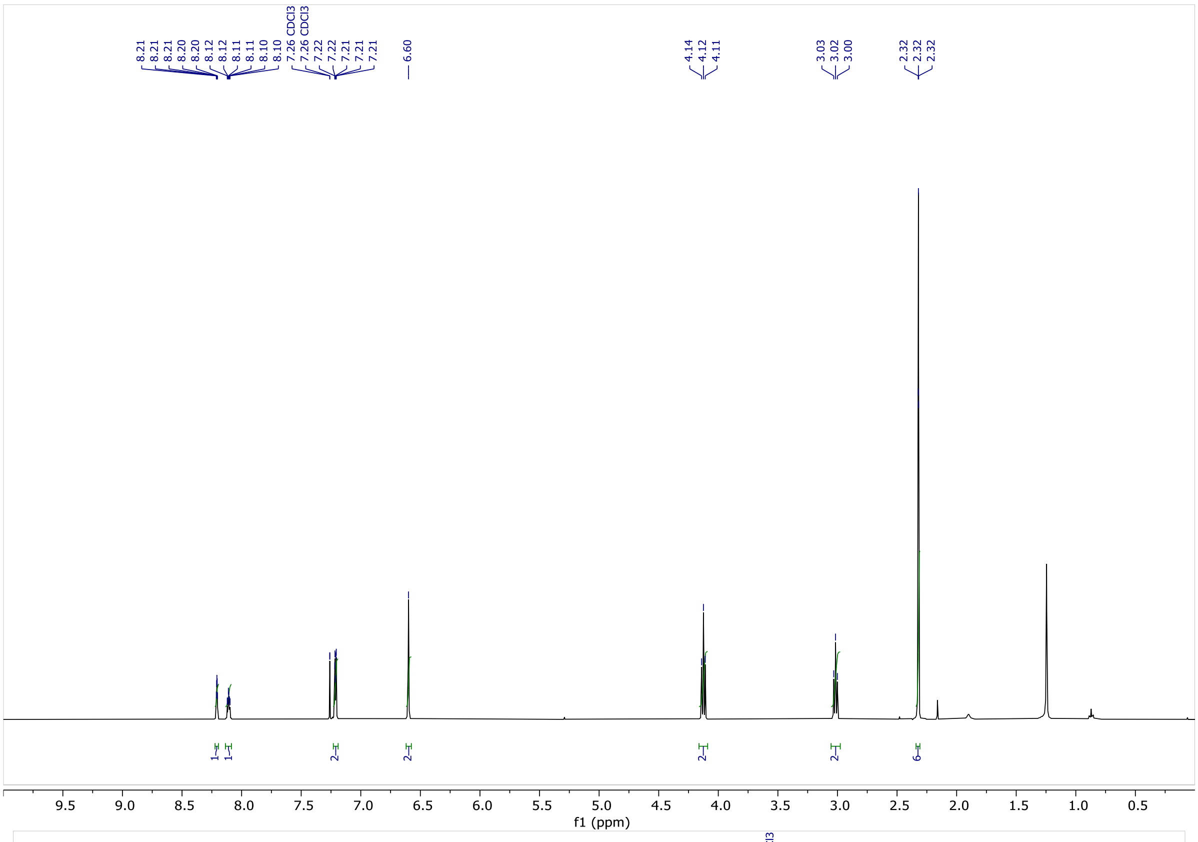

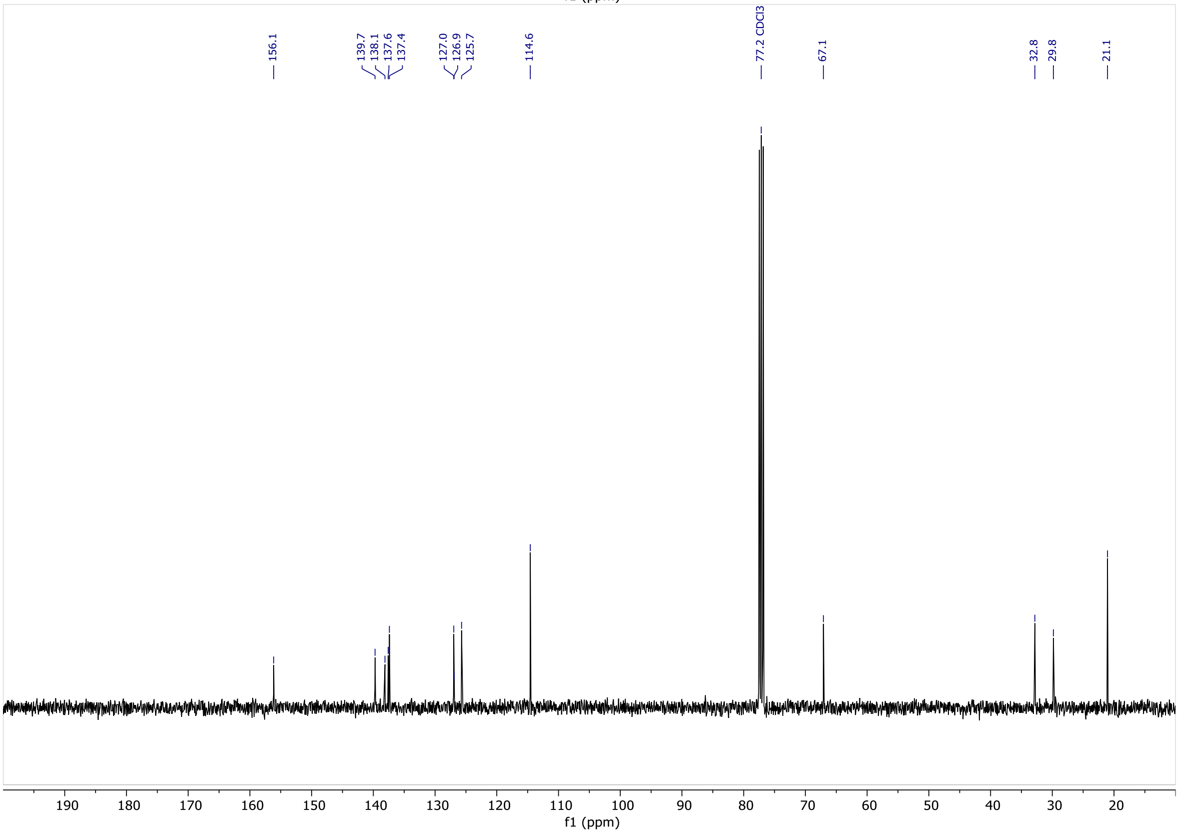

3-(2-(3,4,5-trimethylphenoxy)ethyl)pyridine (31a)

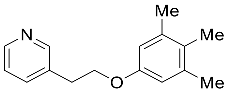

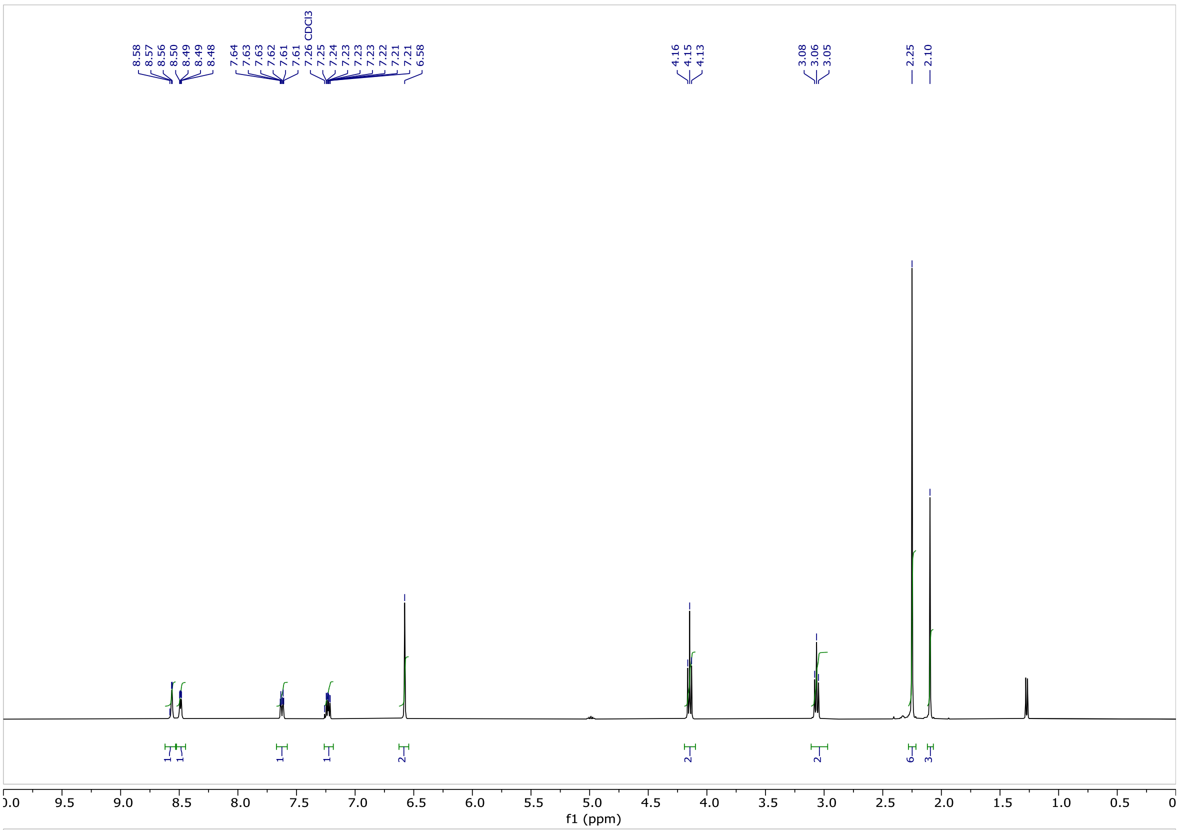

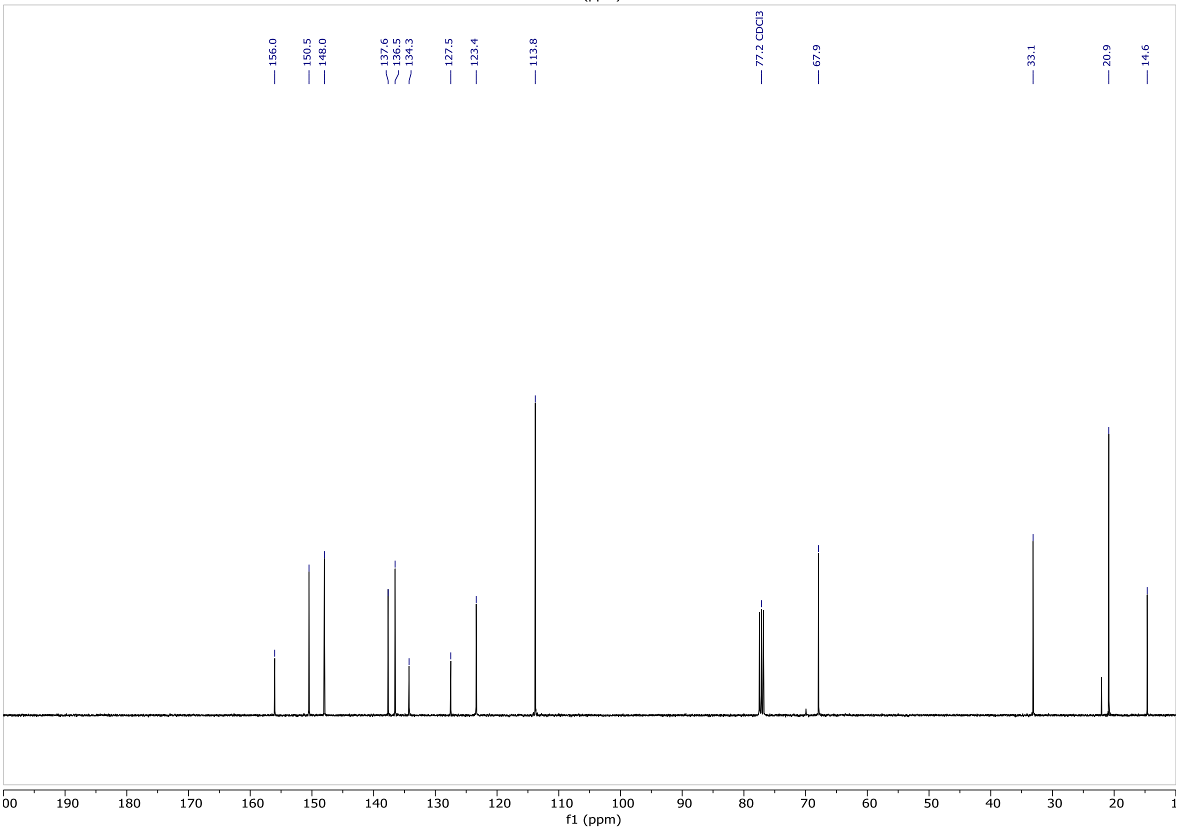

3-(2-(3,4,5-trimethylphenoxy)ethyl)pyridine 1-oxide (31)

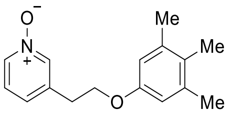

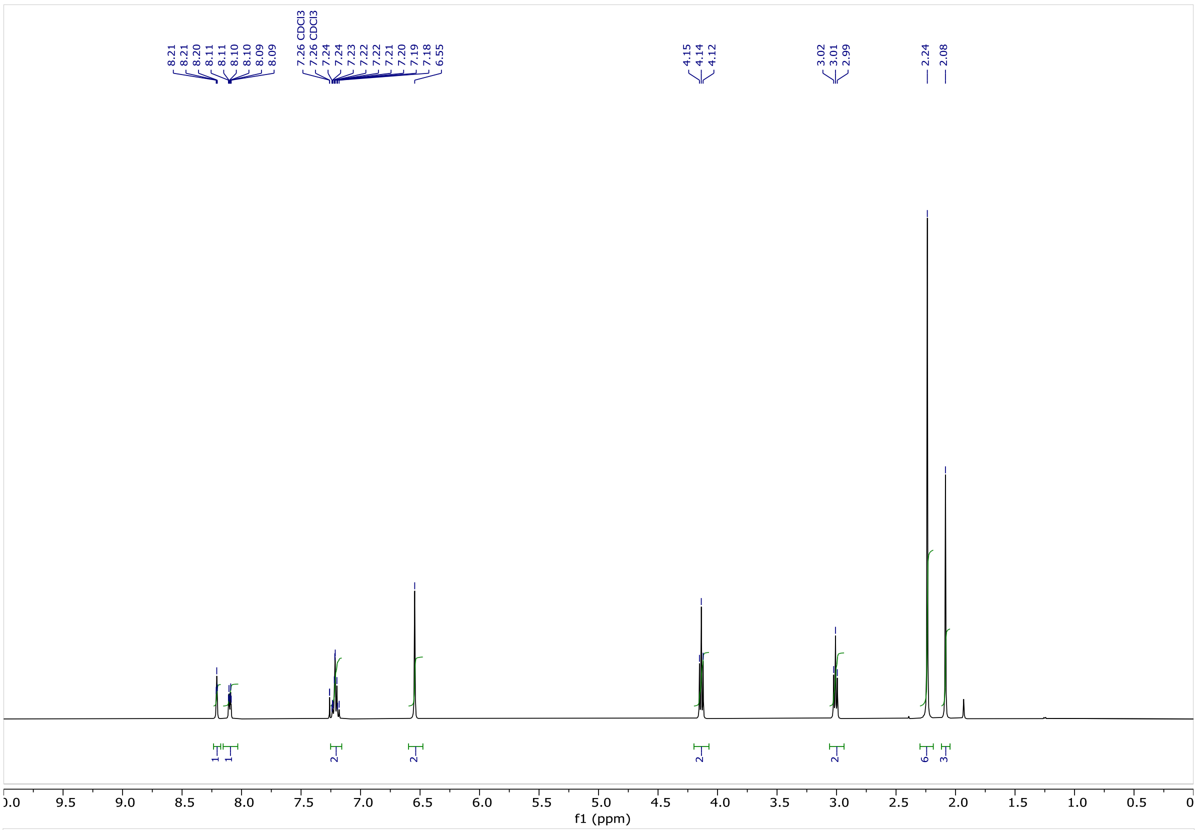

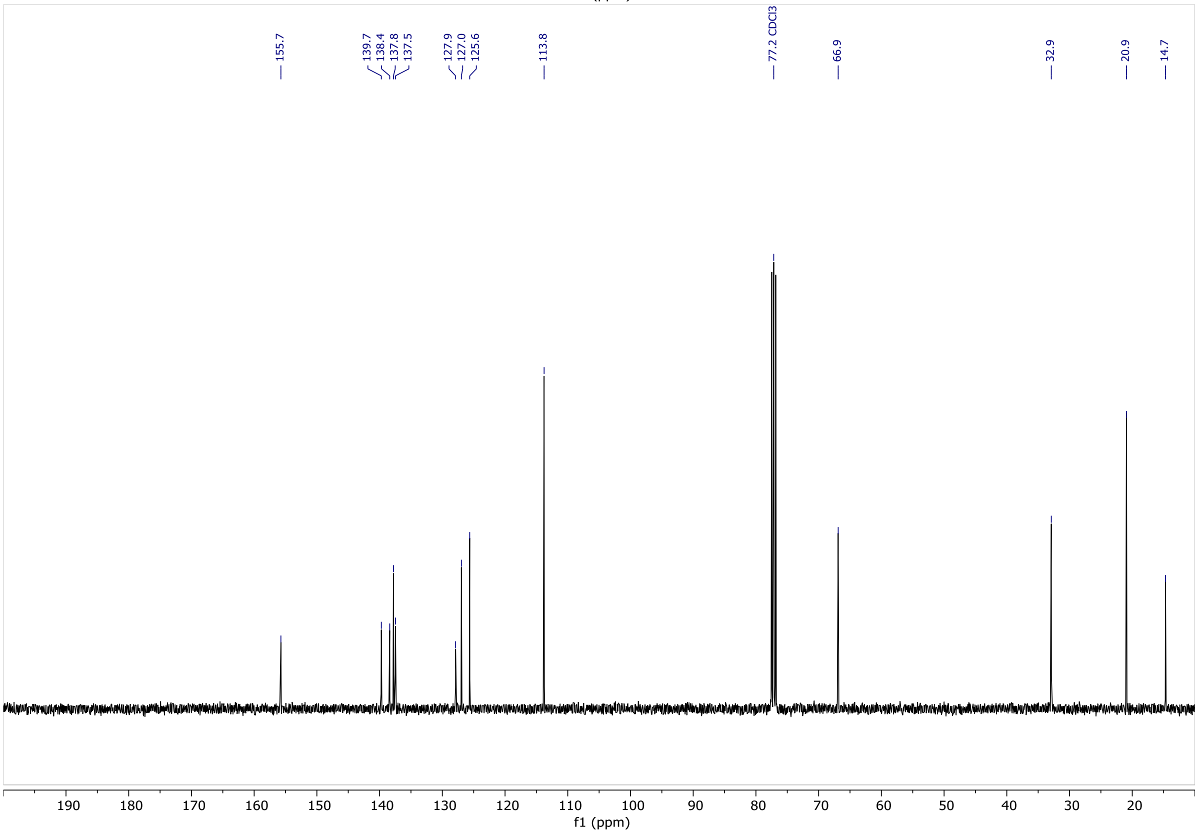

3-(2-(4-chloro-3-iodophenoxy)ethyl)pyridine (32a)

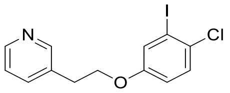

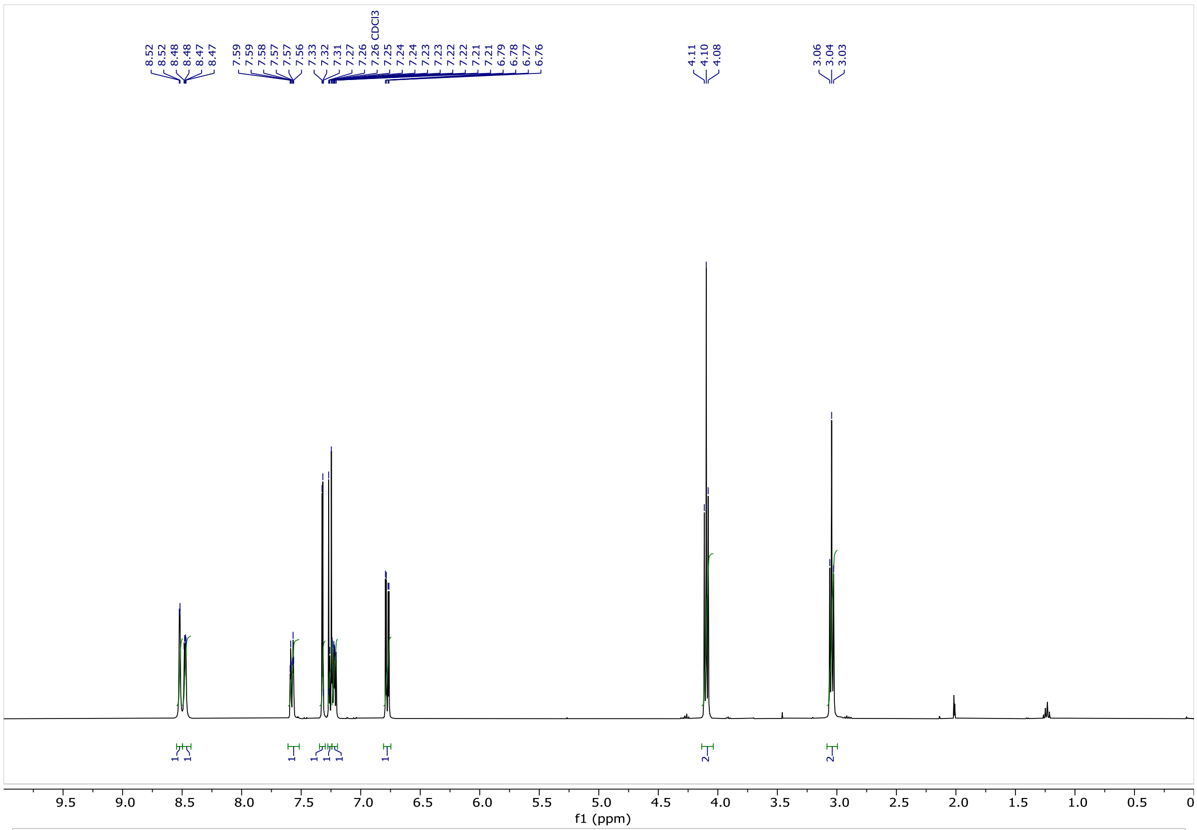

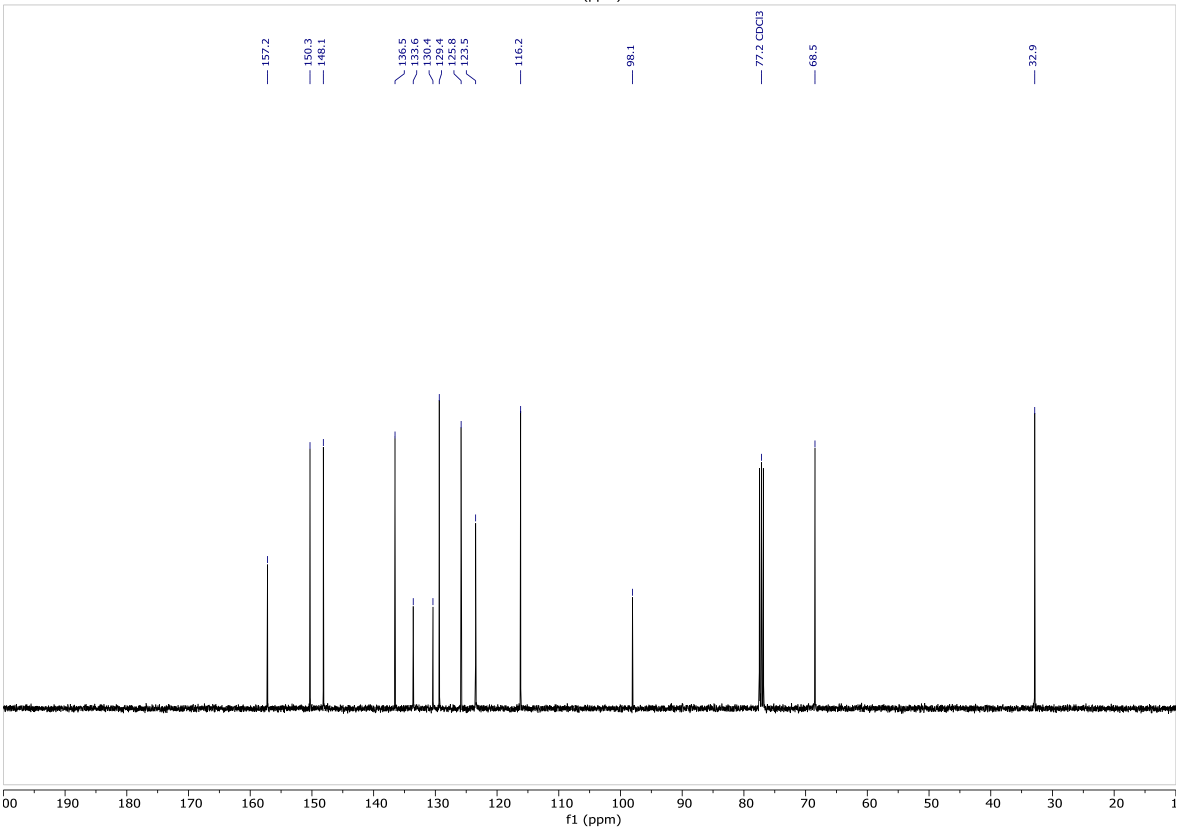

3-(2-(4-chloro-3-iodophenoxy)ethyl)pyridine 1-oxide (32)

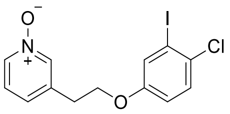

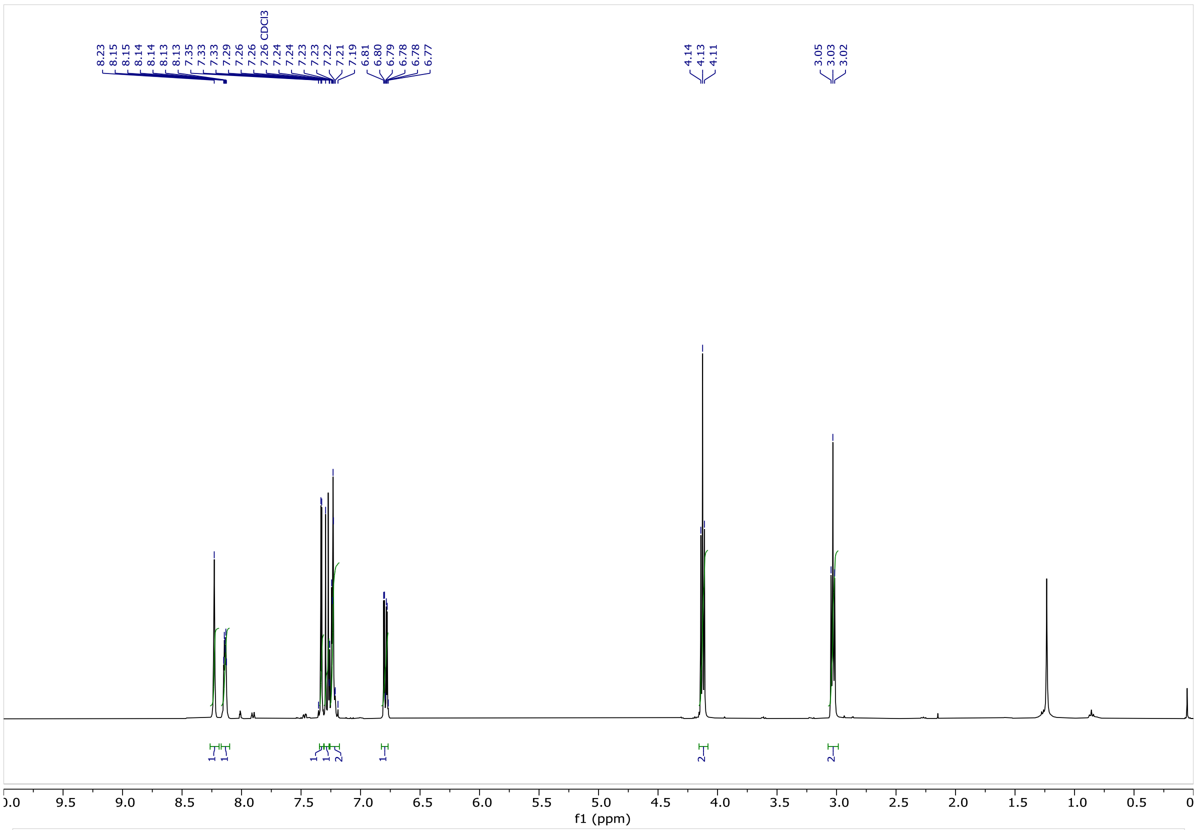

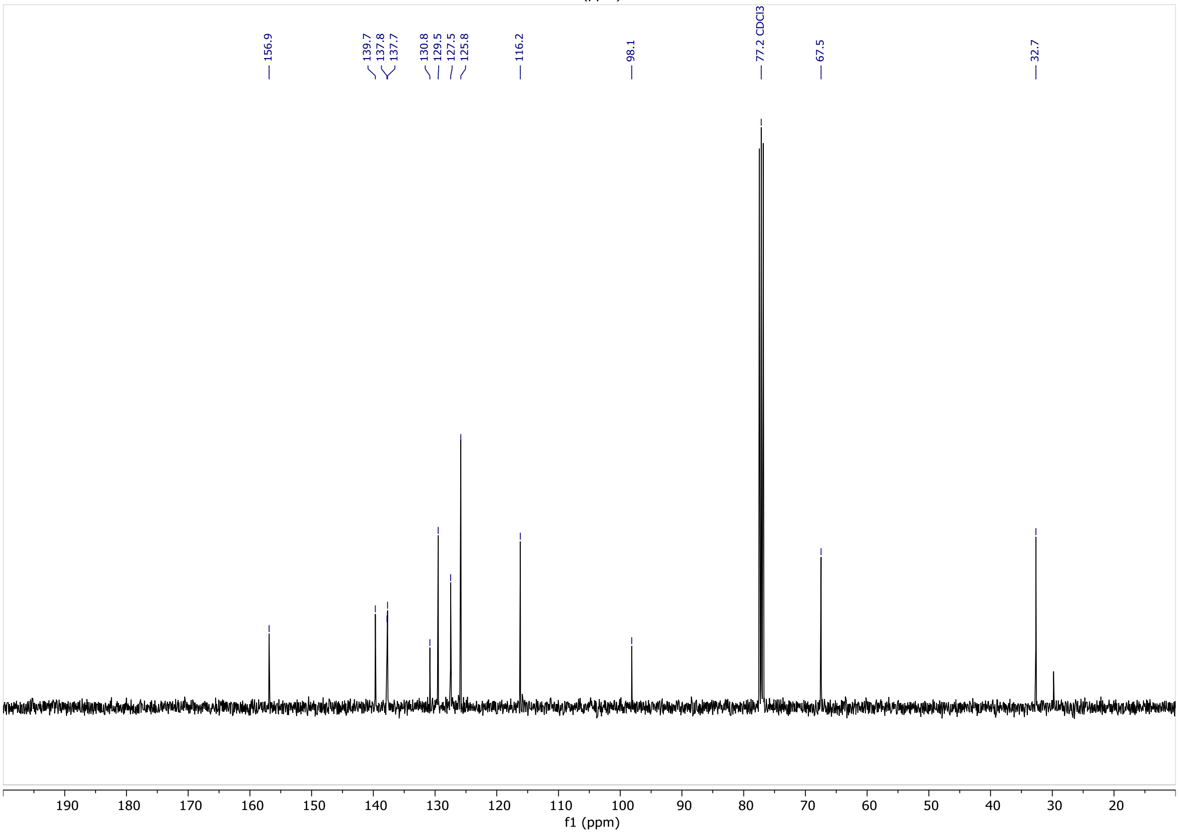

3-(2-(3-bromo-4-chlorophenoxy)ethyl)pyridine (33a)

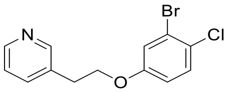

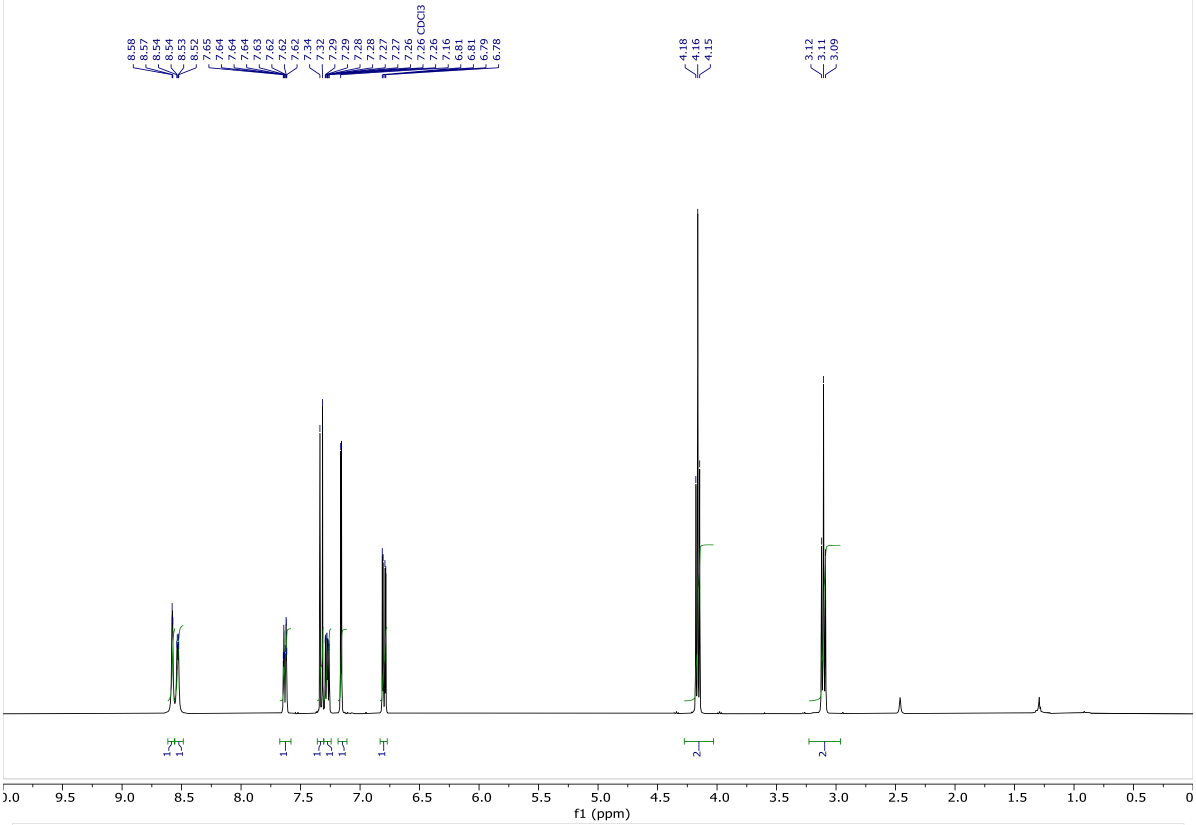

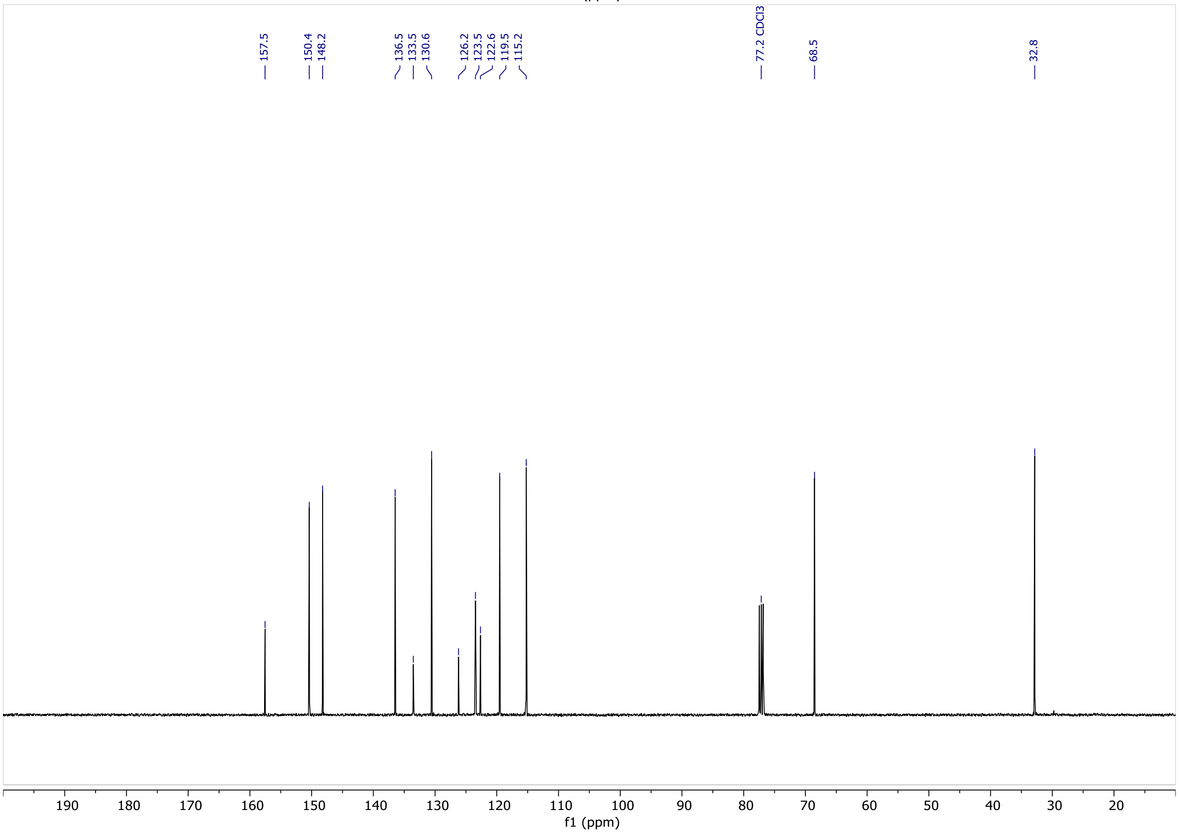

3-(2-(3-bromo-4-chlorophenoxy)ethyl)pyridine 1-oxide (33)

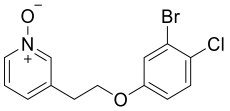

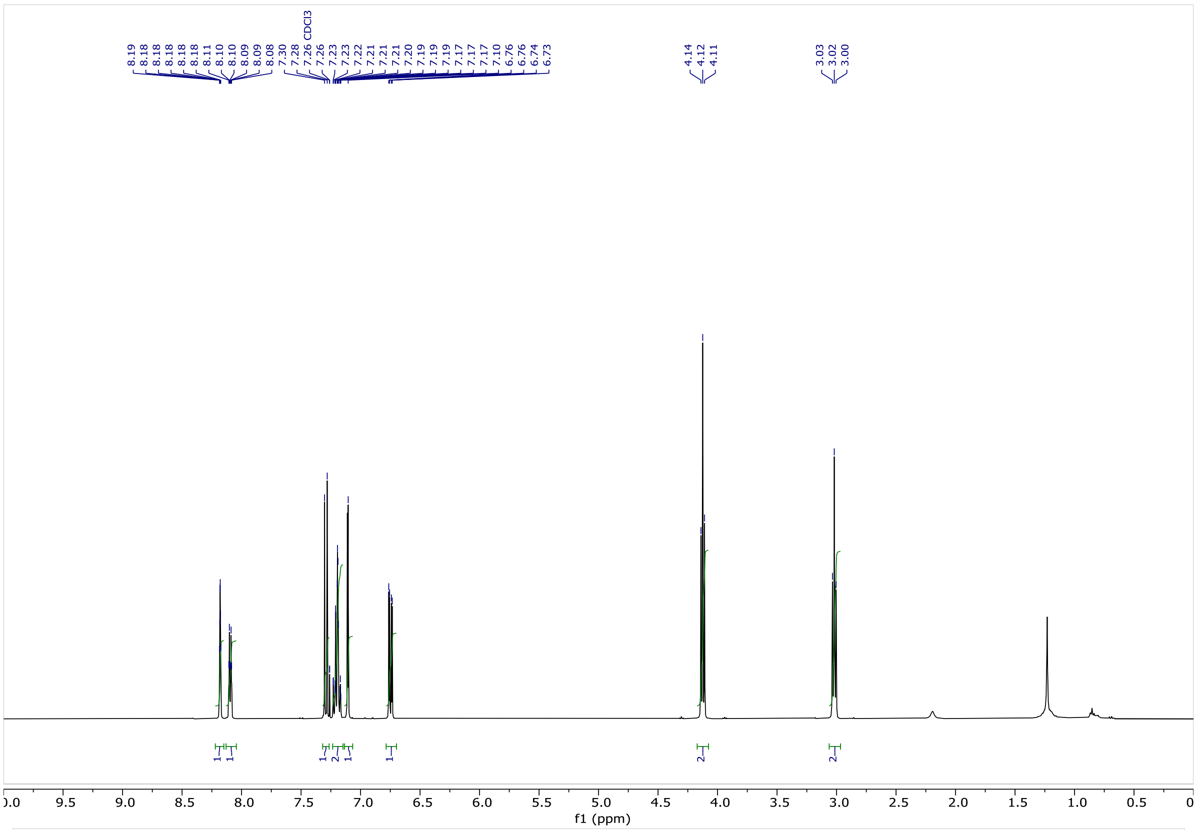

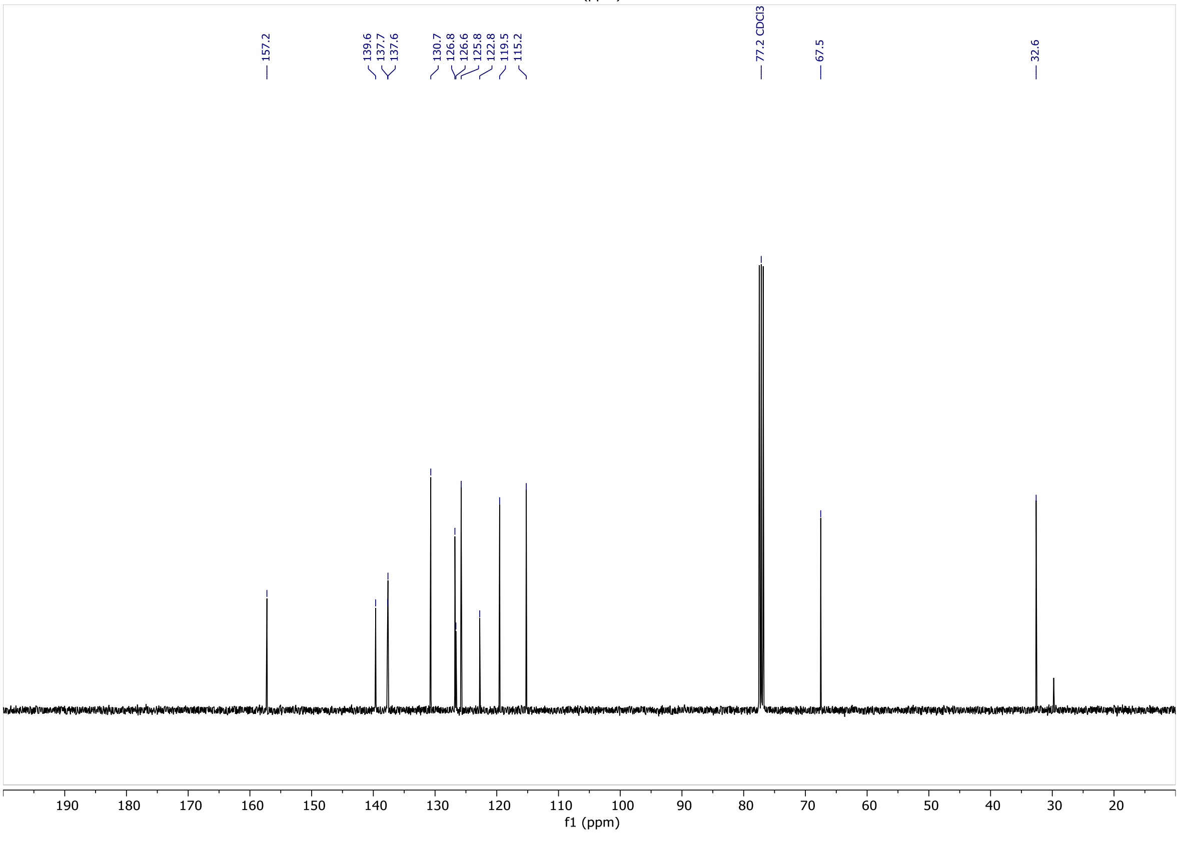

3-(2-(3,4-dichlorophenoxy)ethyl)pyridine (34a)

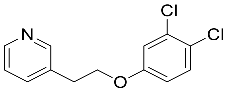

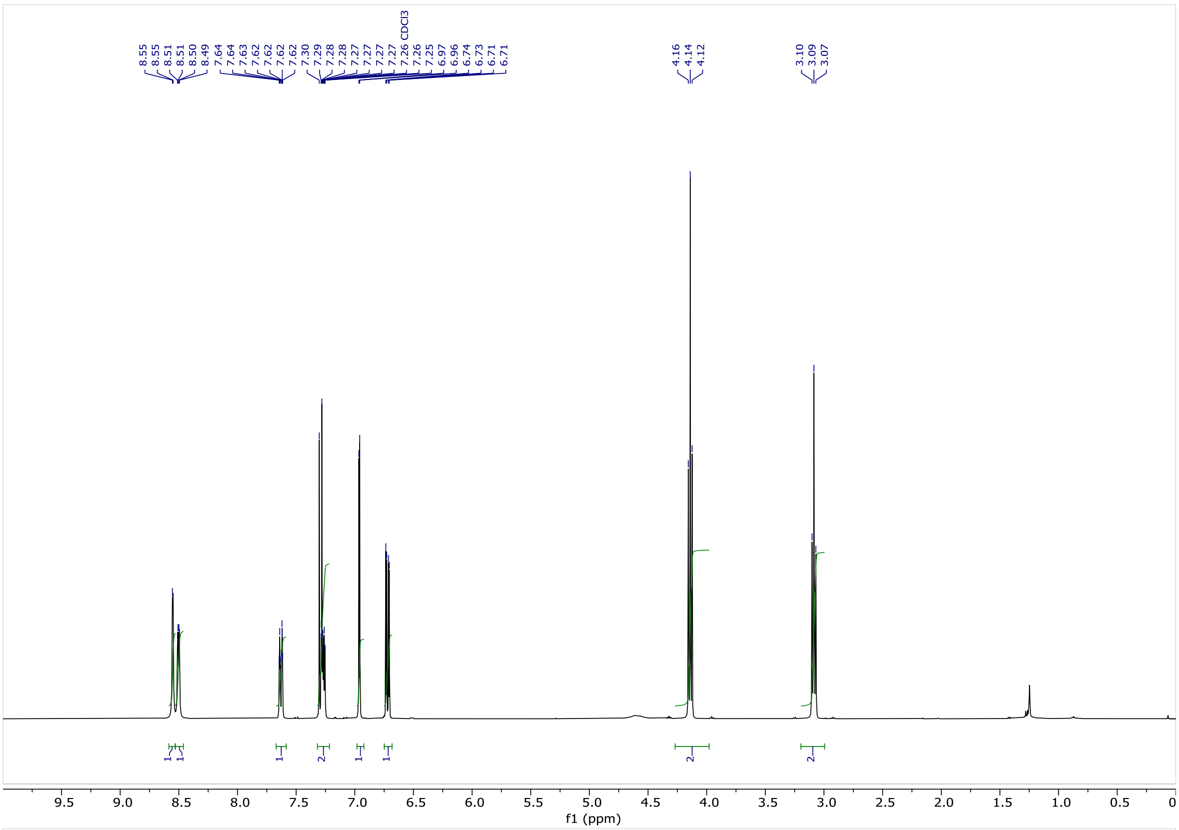

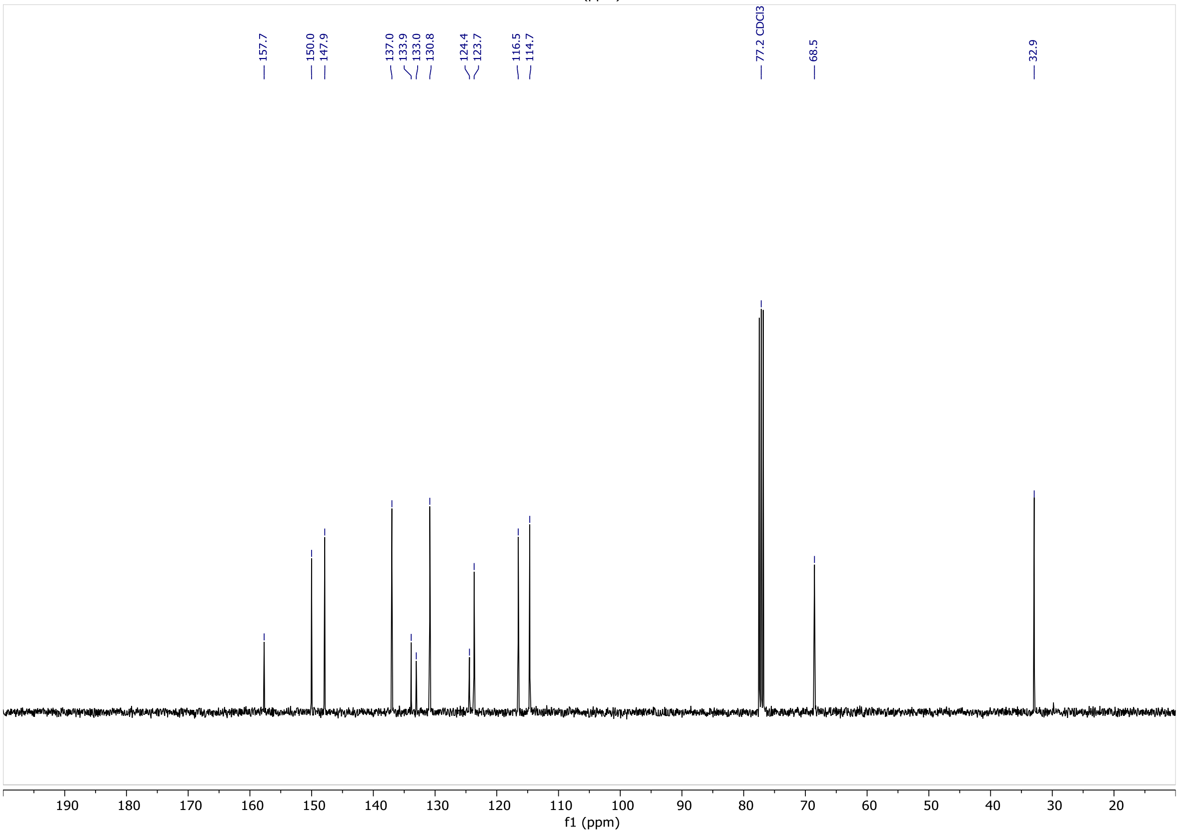

3-(2-(3,4-dichlorophenoxy)ethyl)pyridine 1-oxide (34)

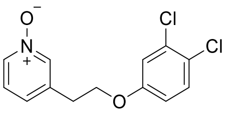

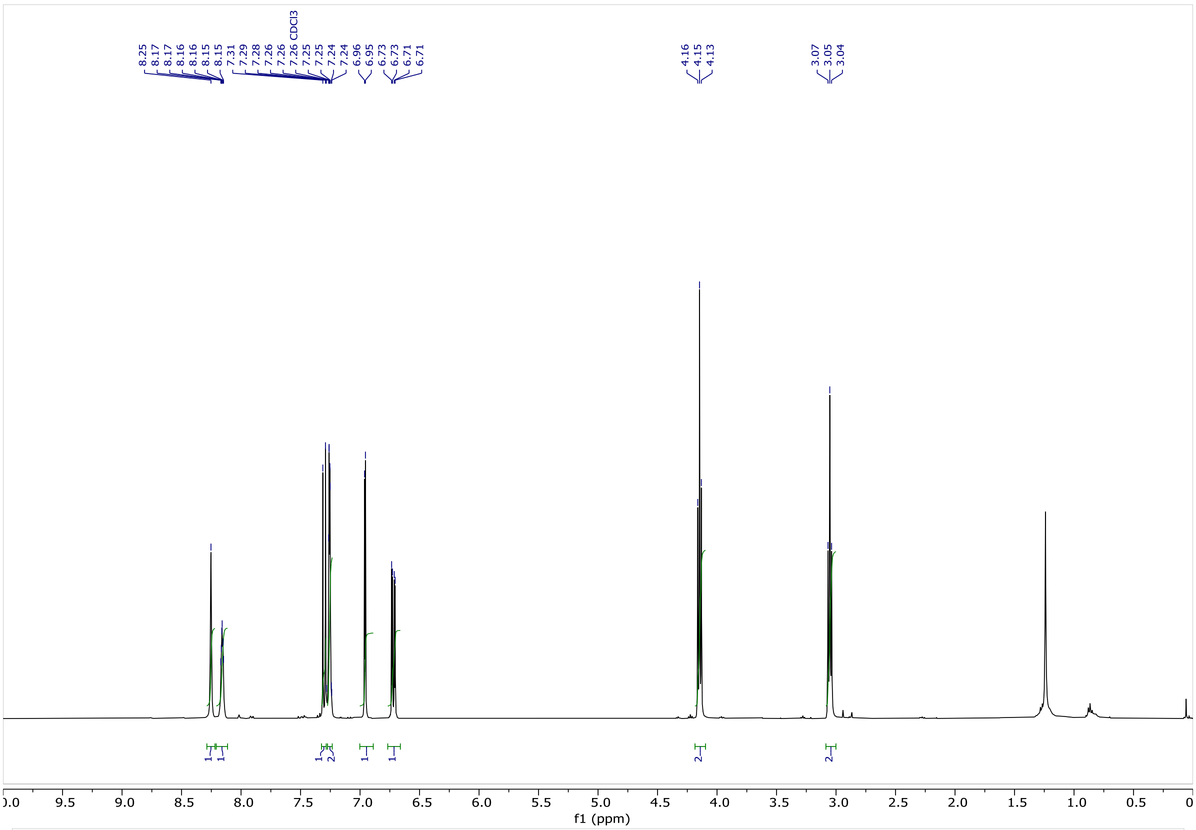

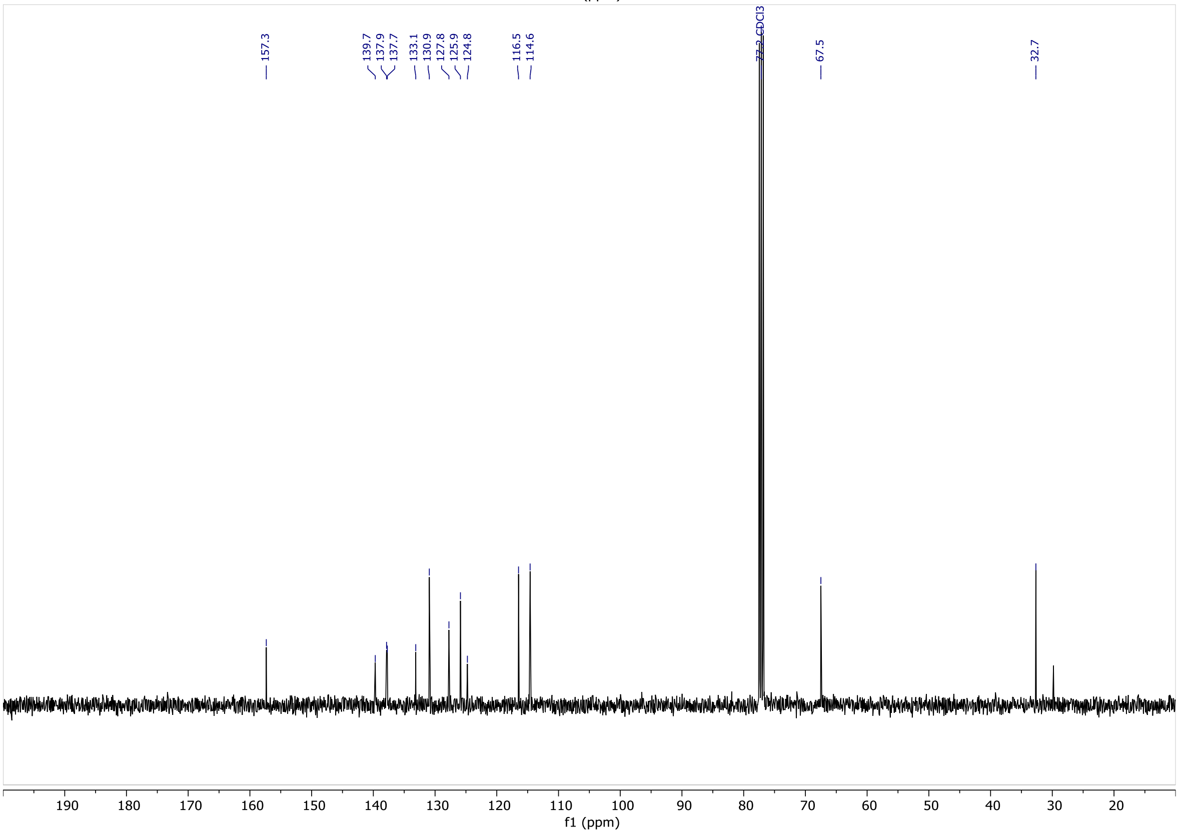

3-(2-(4-chloro-3-fluorophenoxy)ethyl)pyridine (35a)

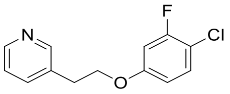

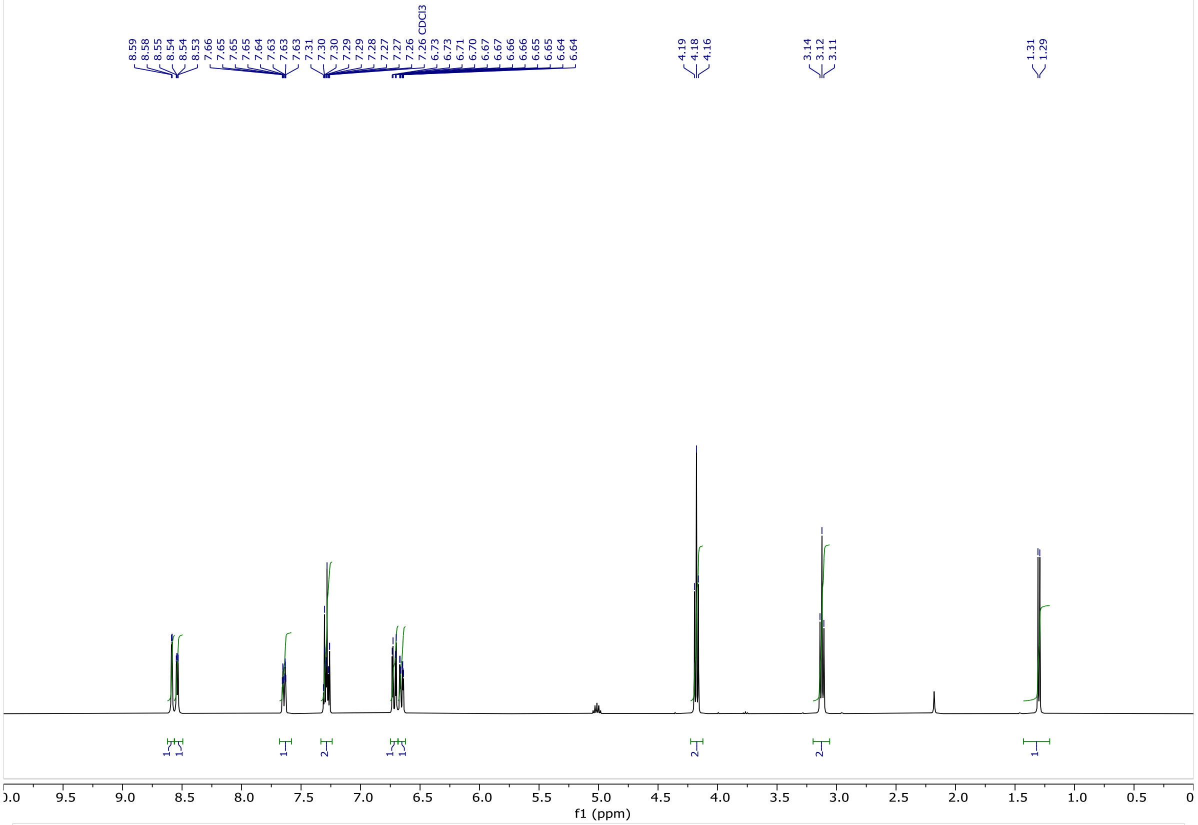

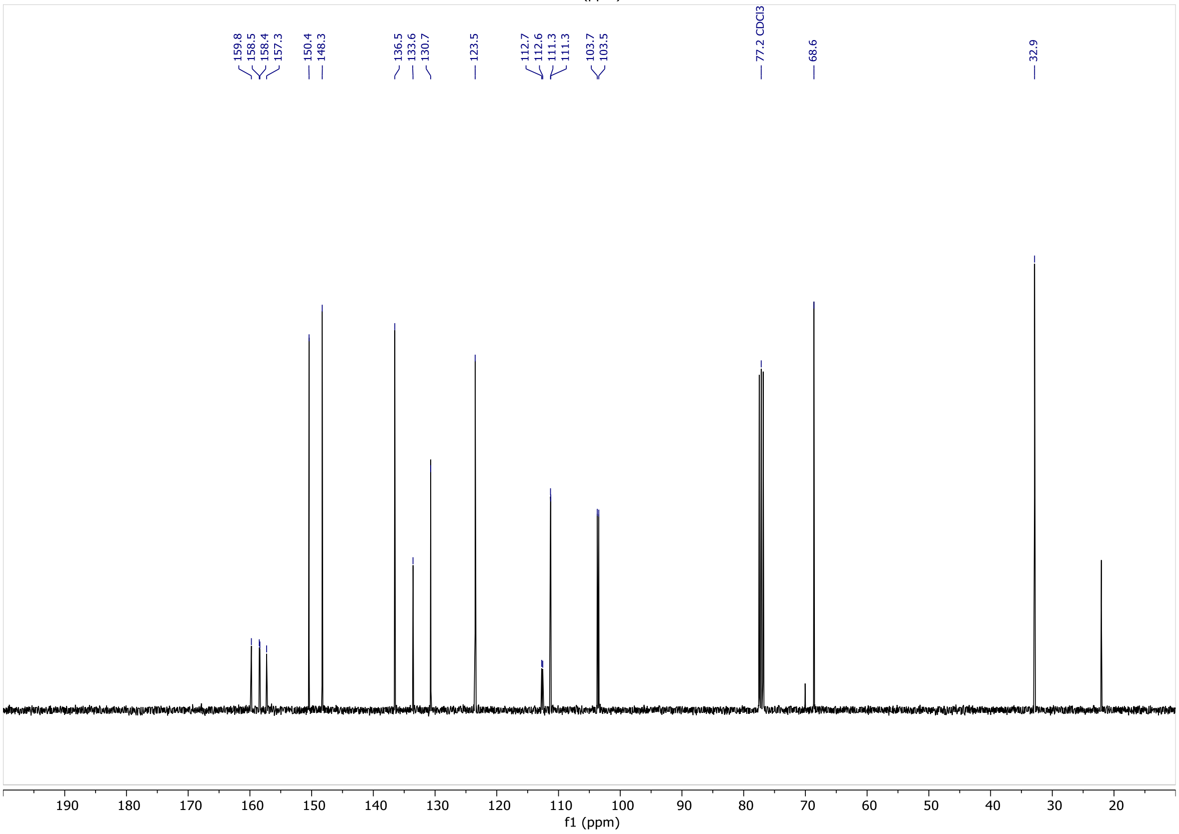

3-(2-(4-chloro-3-fluorophenoxy)ethyl)pyridine 1-oxide (35)

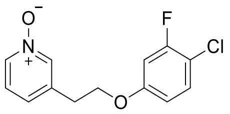

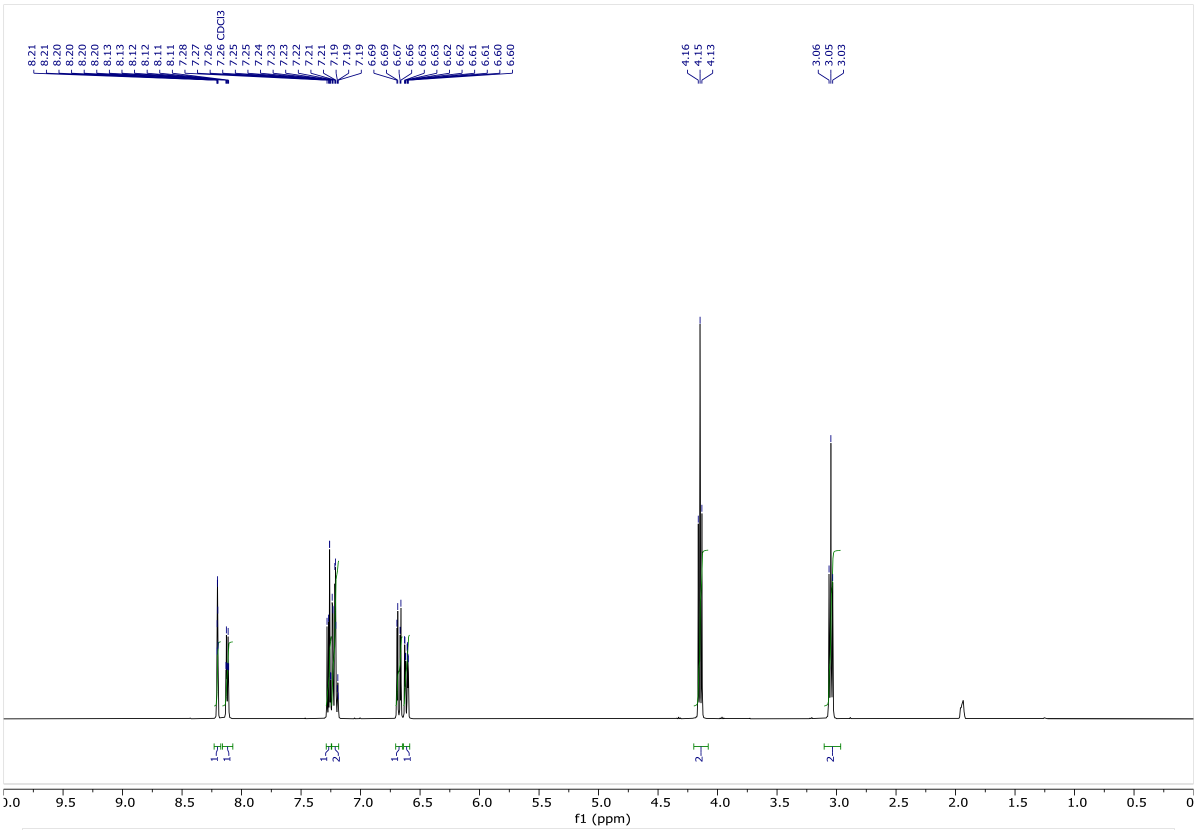

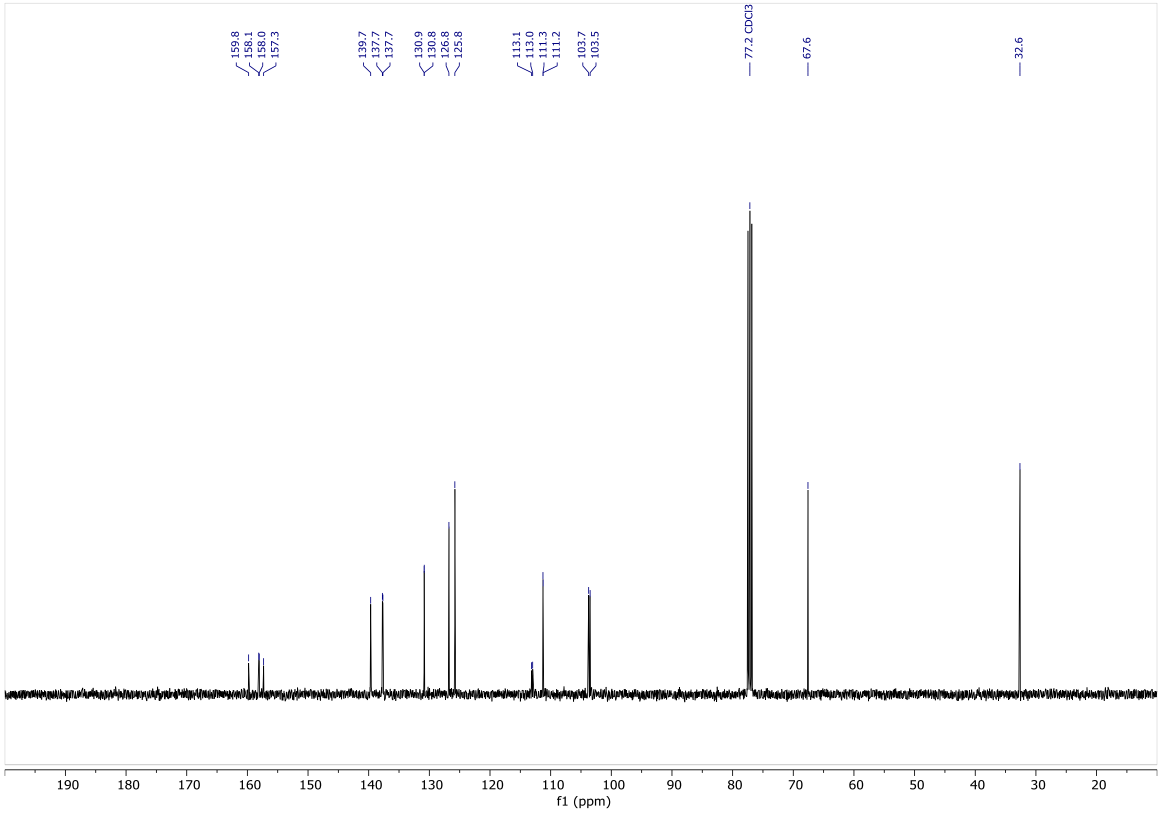

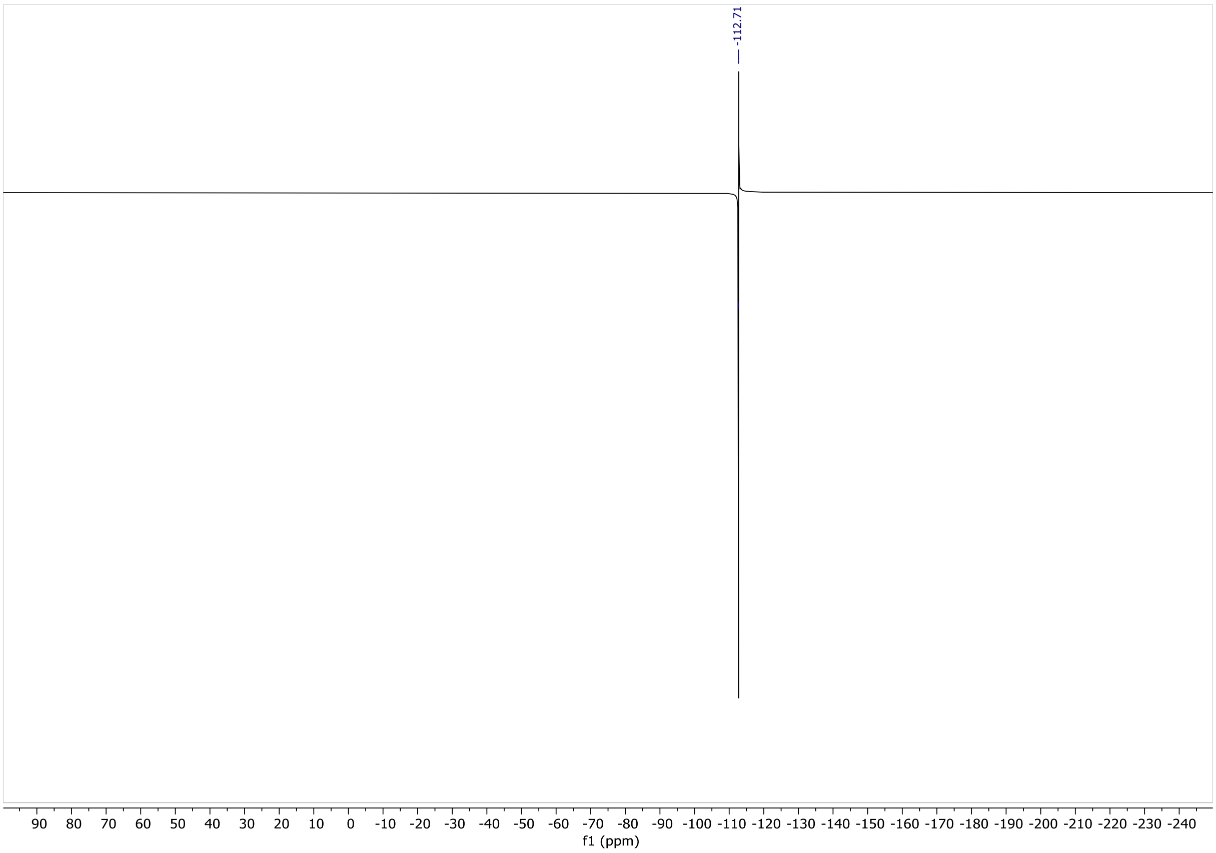
-112.71
90 80 70 60 50 40 30 20 10 0 -10 -20 -30 -40 -50 -60 -70 -80 -90 -100 -110 -120 -130 -140 -150 -160 -170 -180 -190 -200 -210 -220 -230 -240
f1 (ppm)

3-(2-((6-chloro-[1,1'-biphenyl]-3-yl)oxy)ethyl)pyridine (36a)

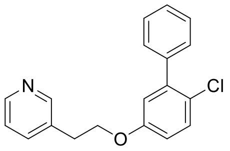

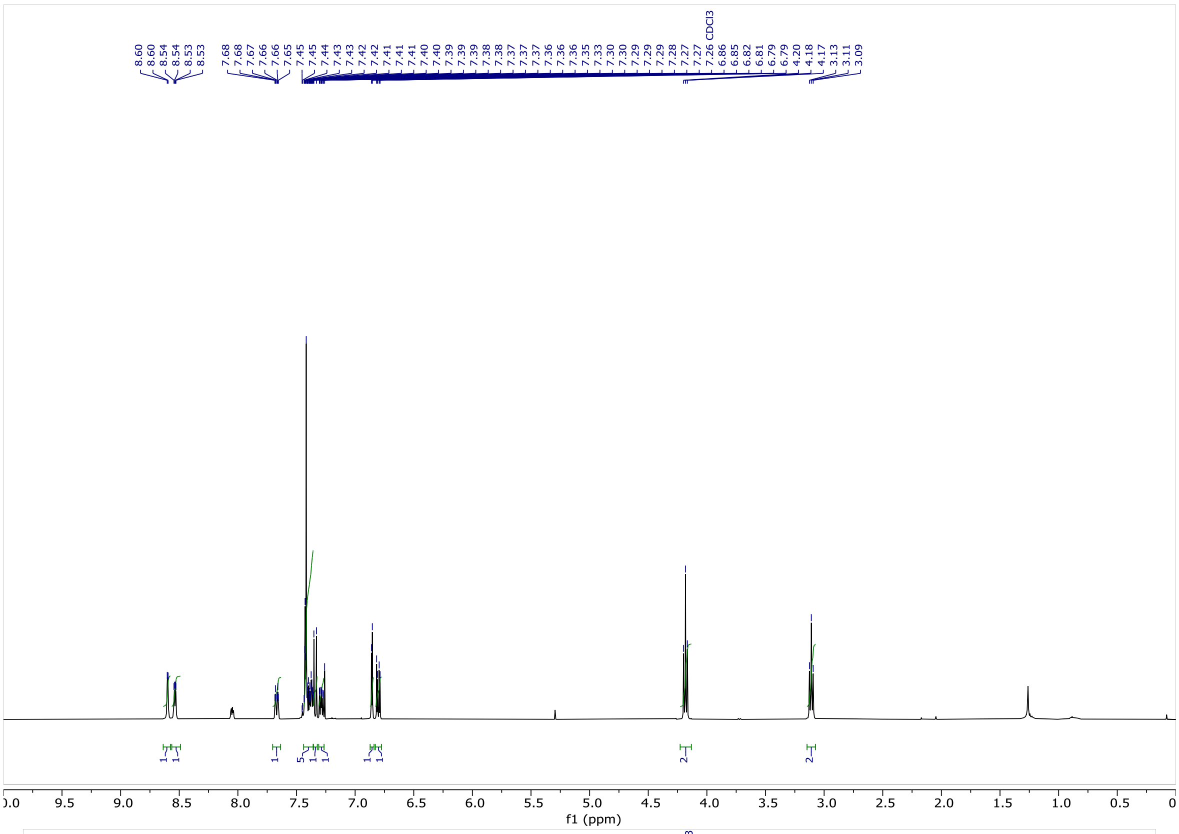

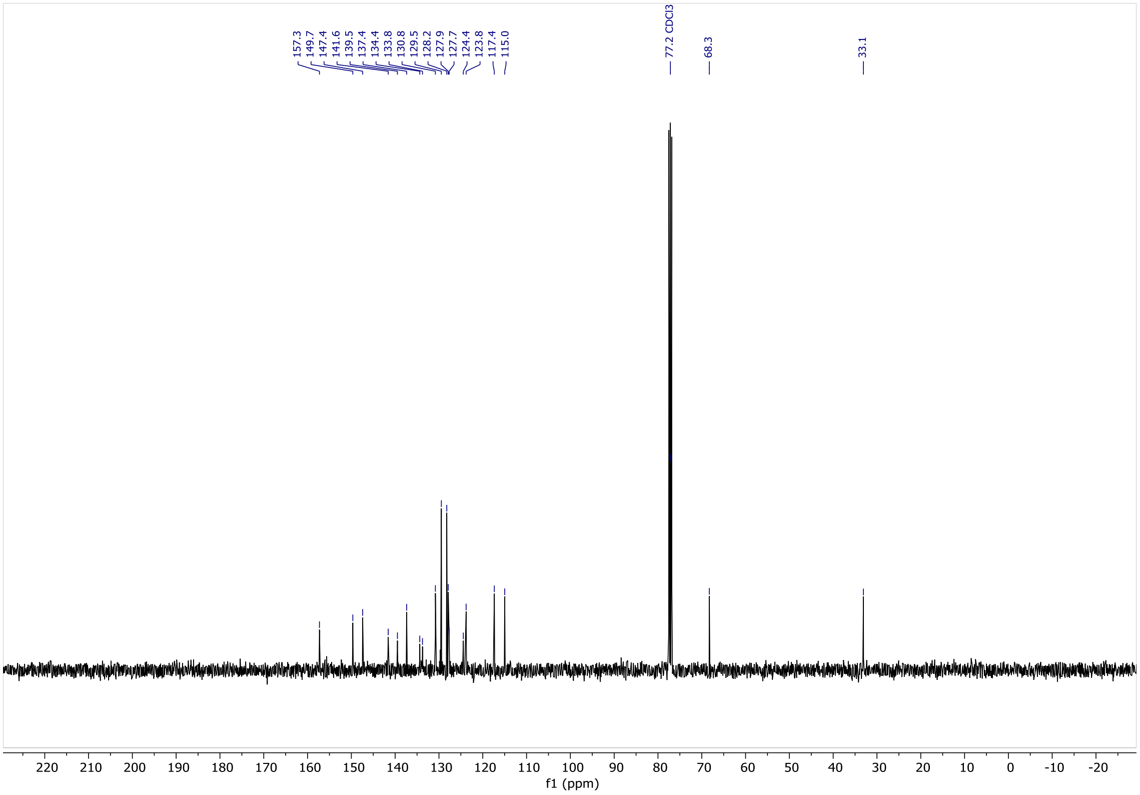

3-(2-((6-chloro-[1,1'-biphenyl]-3-yl)oxy)ethyl)pyridine 1-oxide (36)

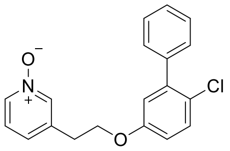

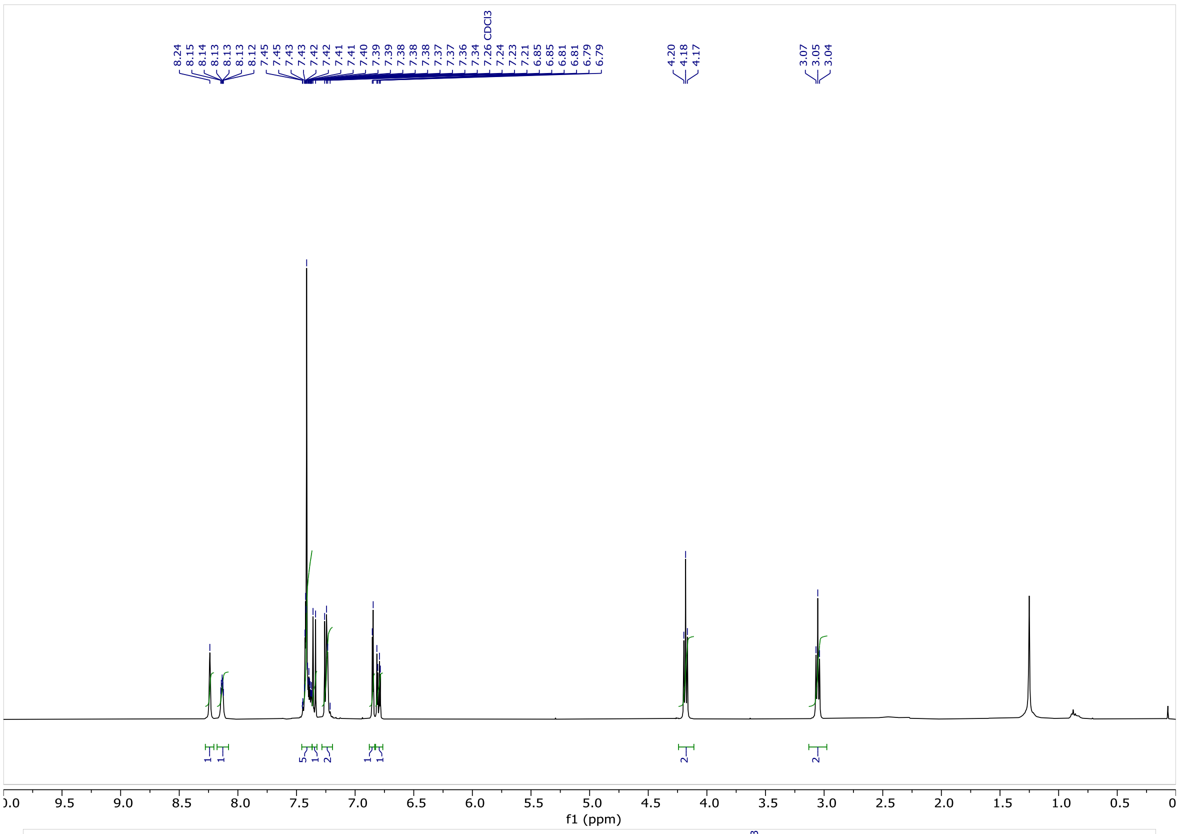

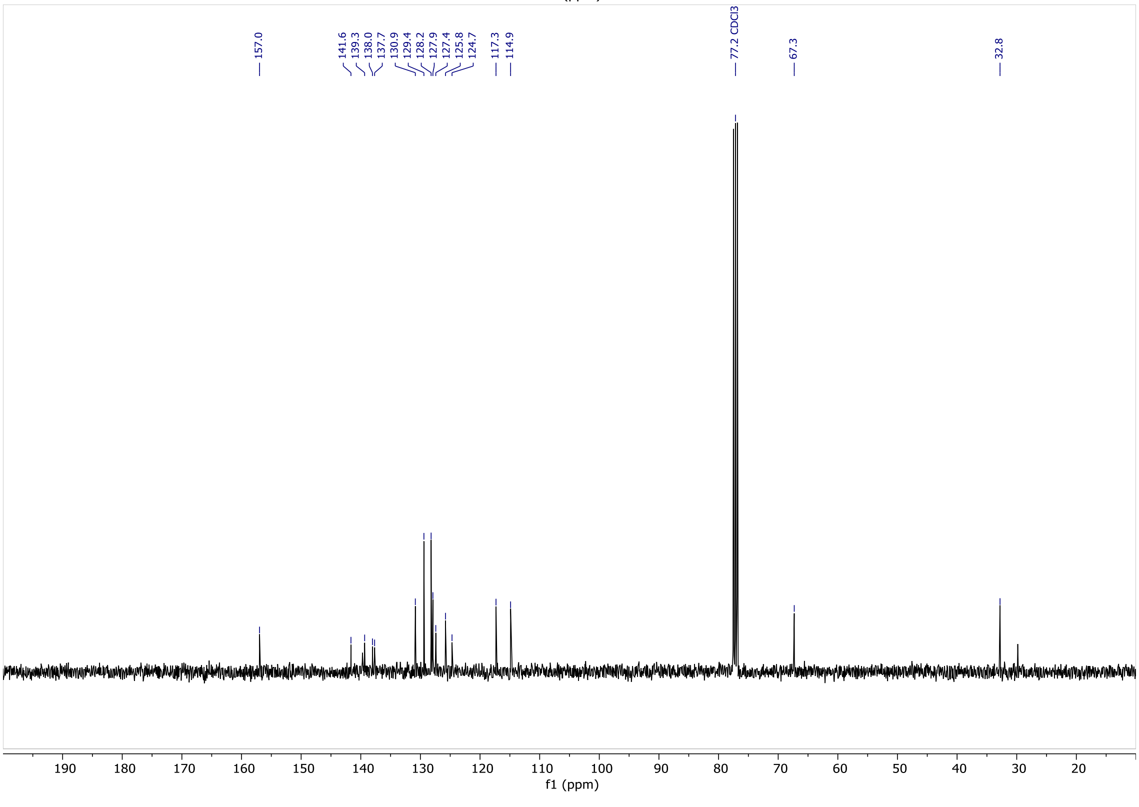

3-(2-(4-chloro-3-methylphenoxy)ethyl)pyridine (37a)

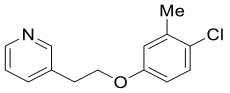

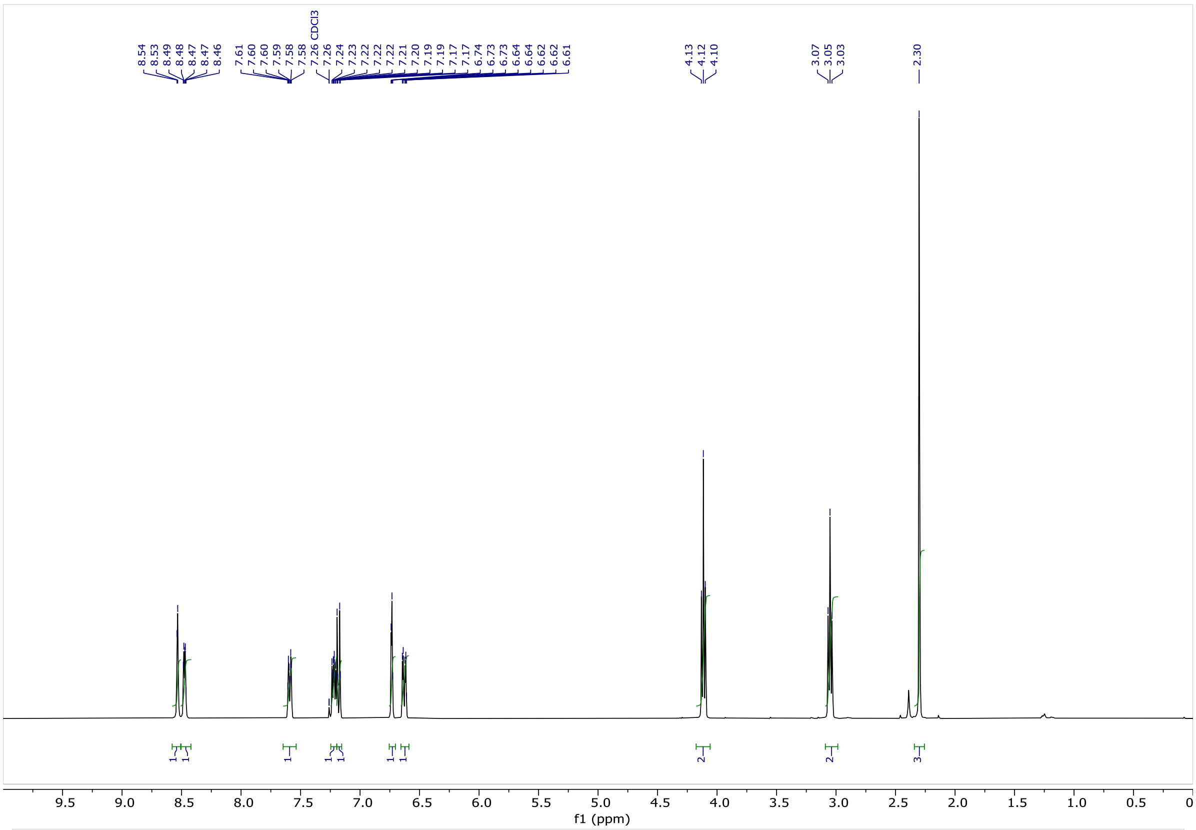

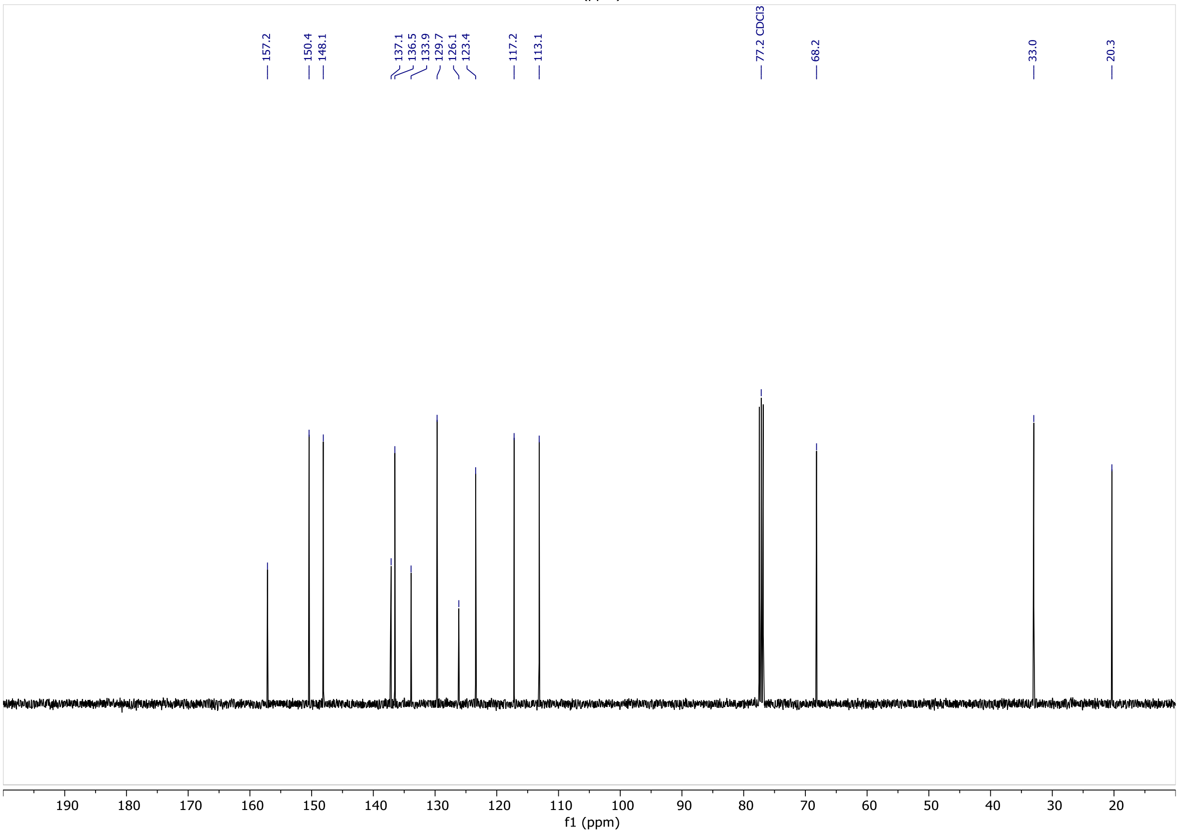

3-(2-(4-chloro-3-methylphenoxy)ethyl)pyridine 1-oxide (37)

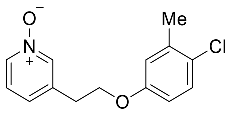

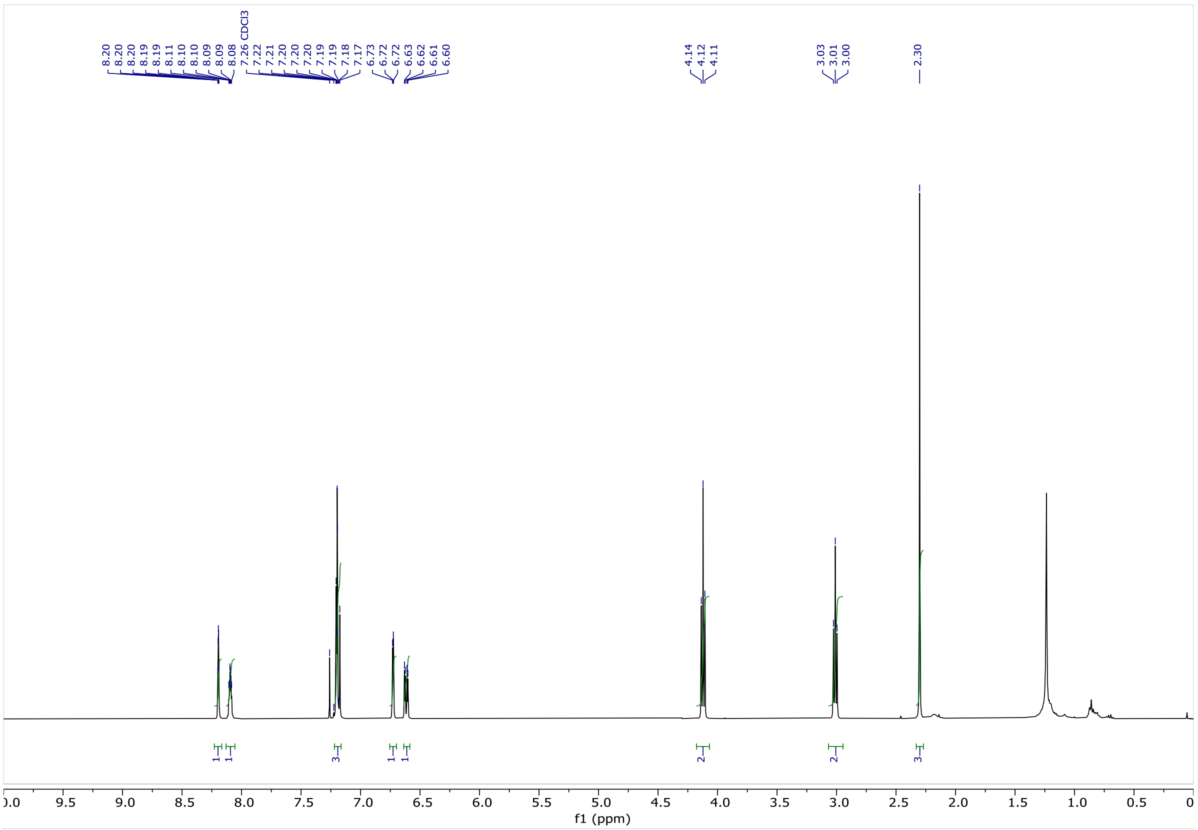

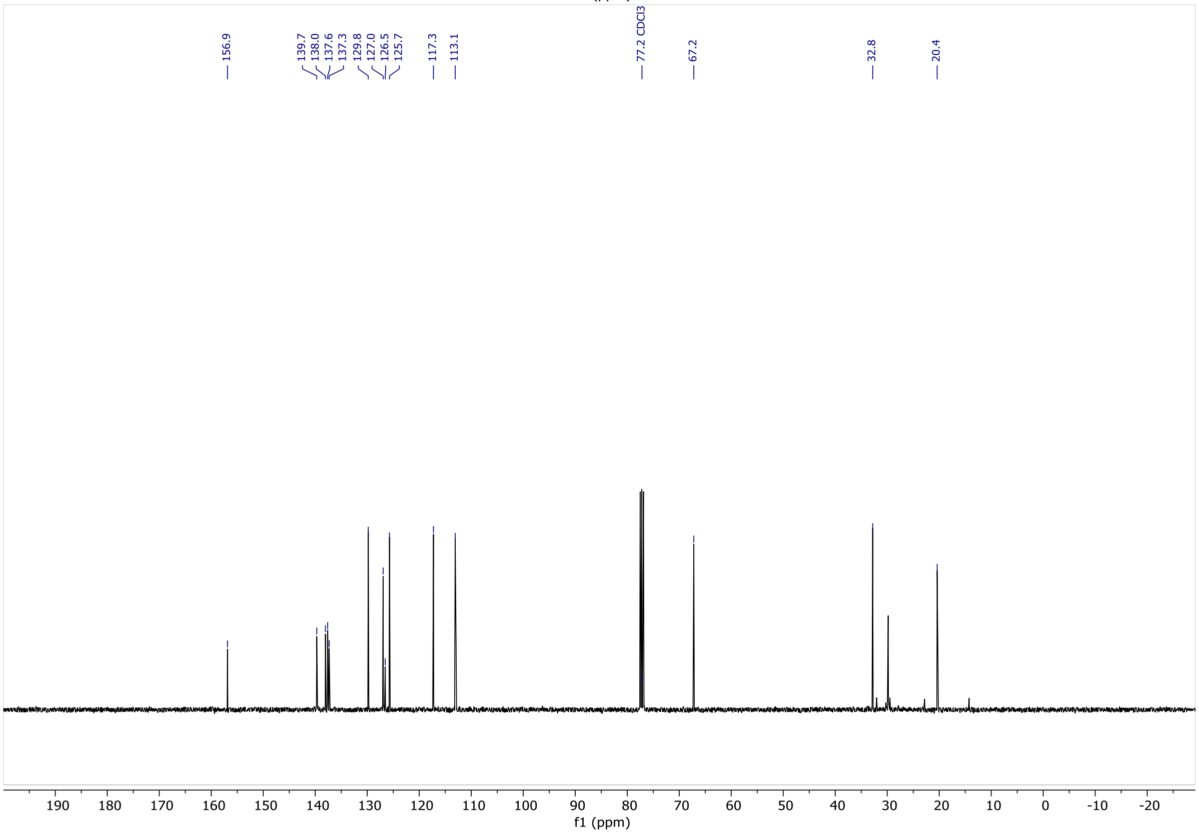

3-(2-(4-chloro-3-nitrophenoxy)ethyl)pyridine (38a)

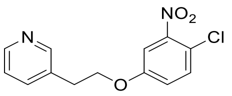

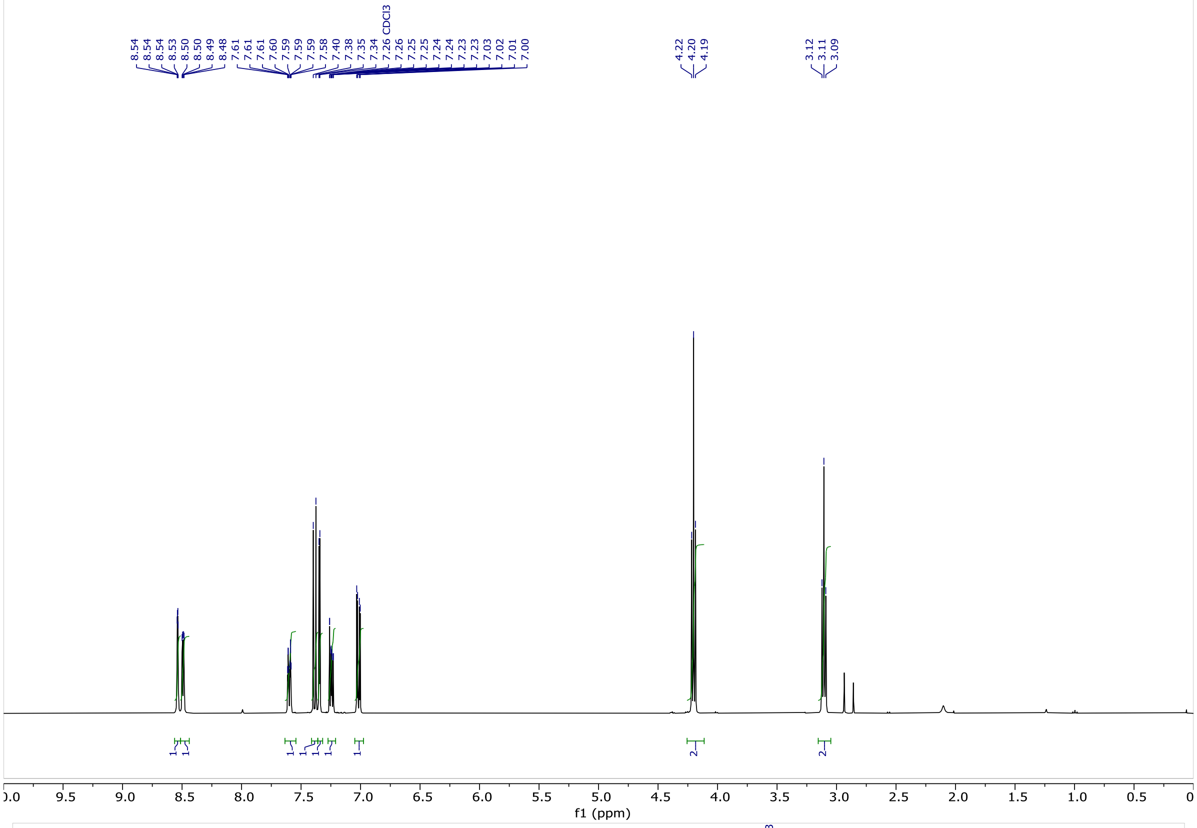

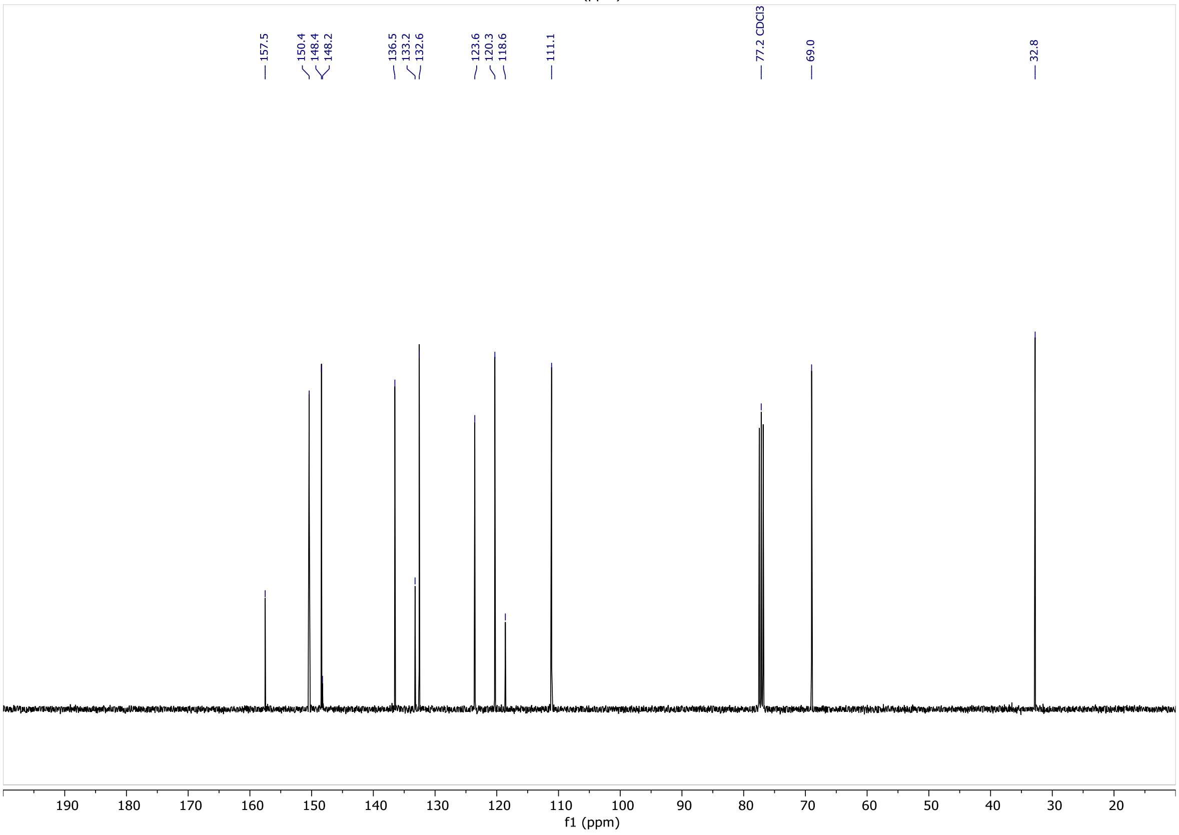

3-(2-(4-chloro-3-nitrophenoxy)ethyl)pyridine 1-oxide (38)

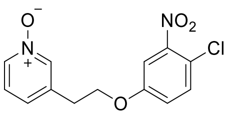

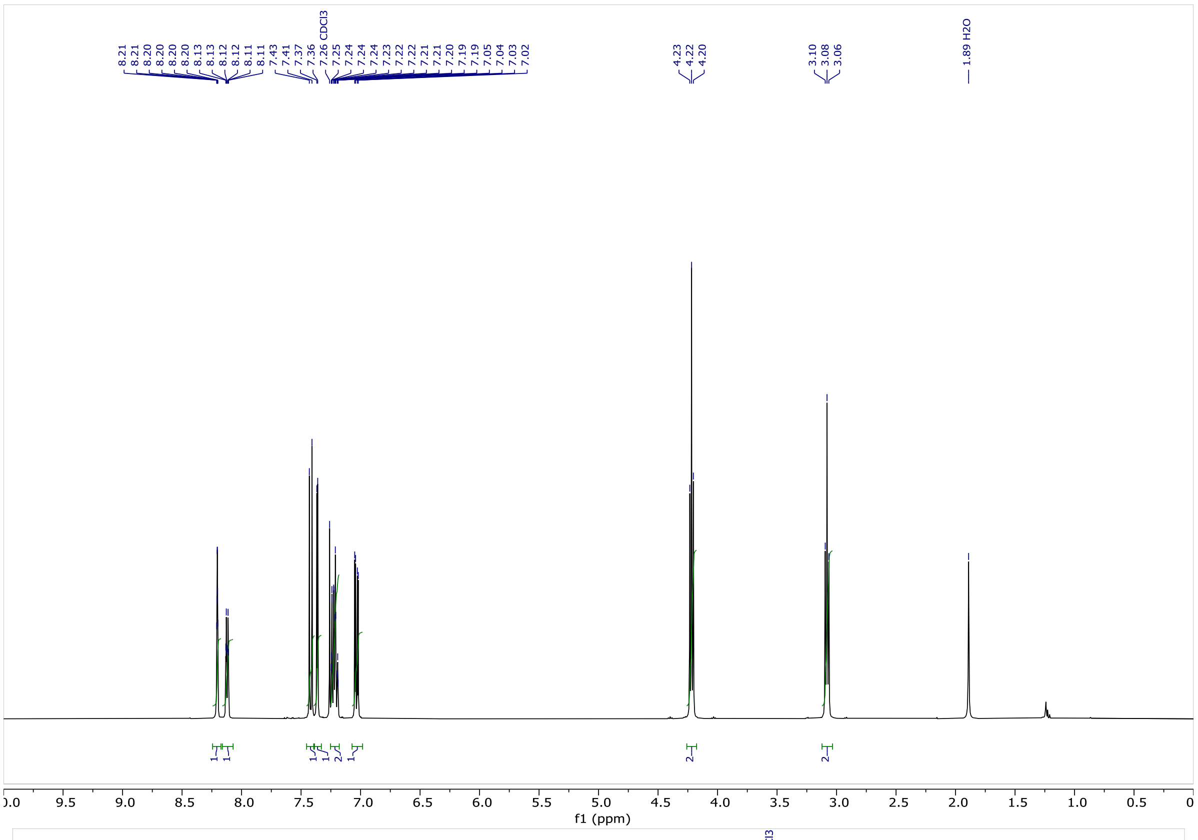

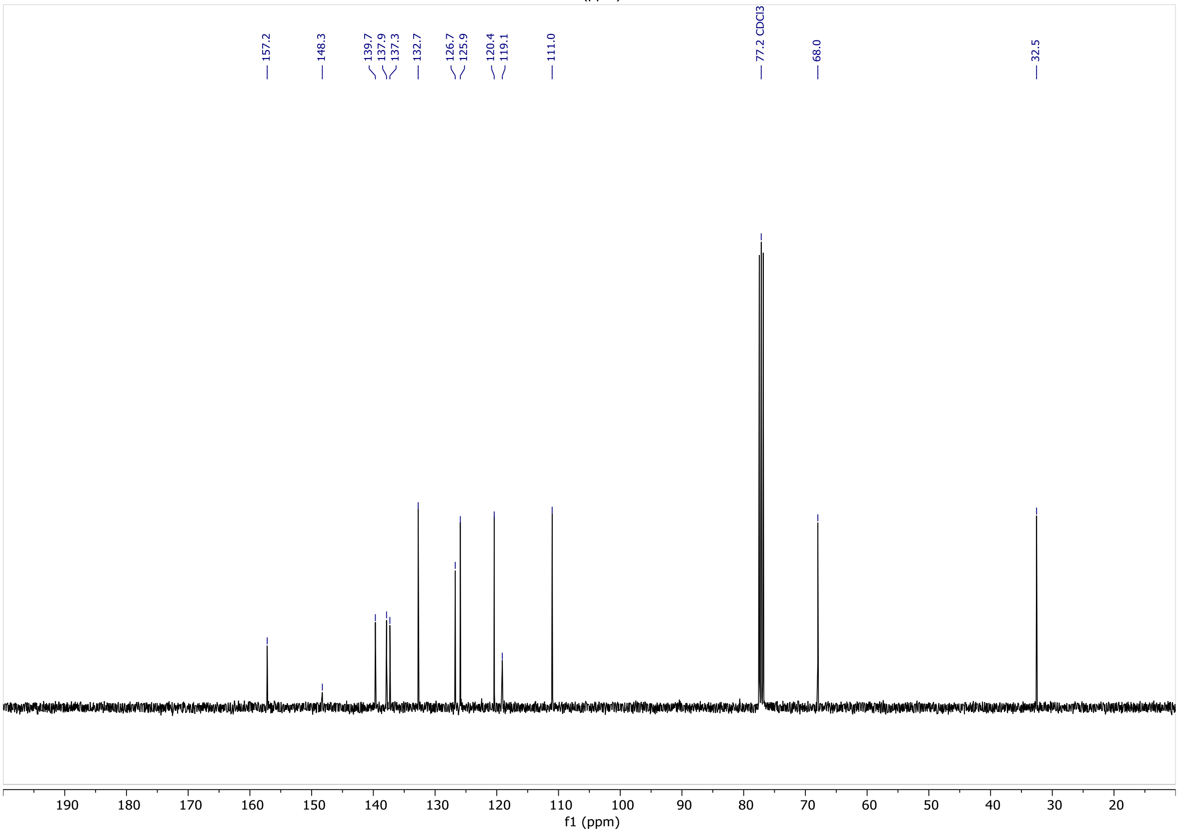

3-(2-(4-chloro-3-ethylphenoxy)ethyl)pyridine (39a)

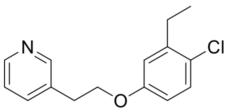

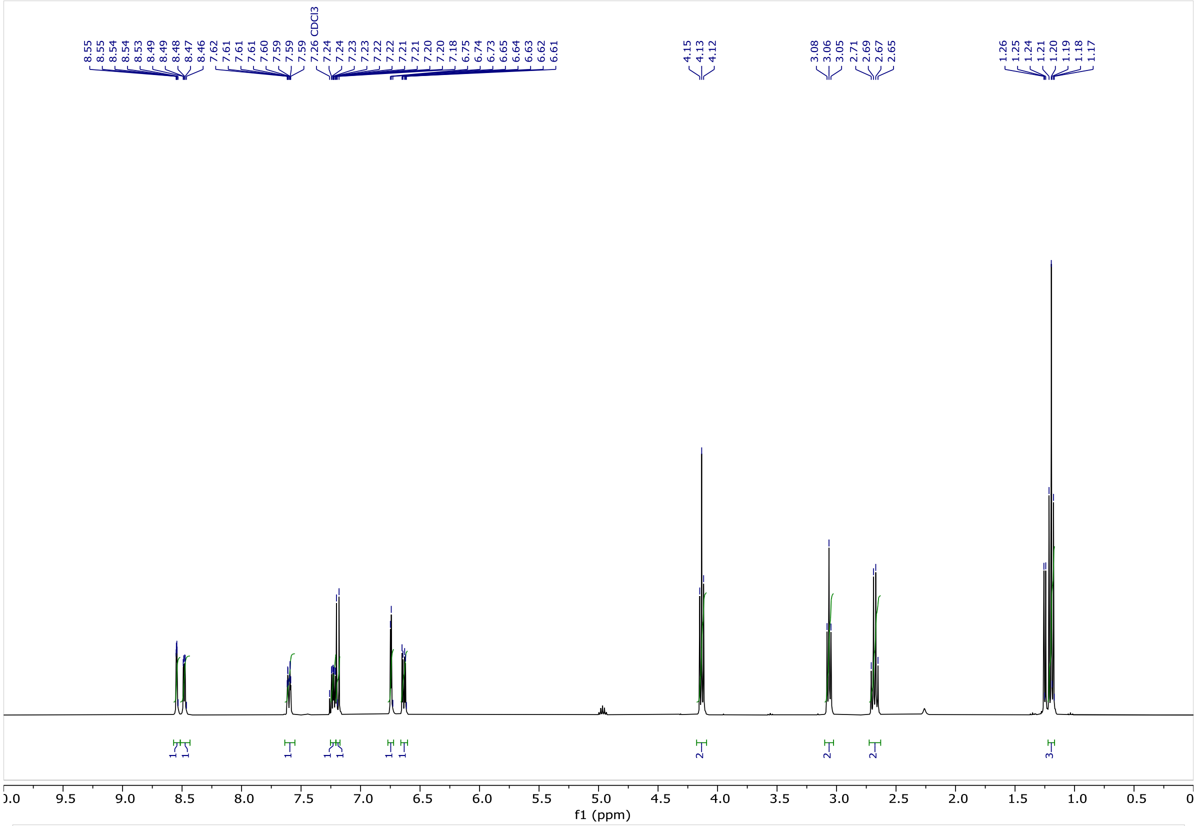

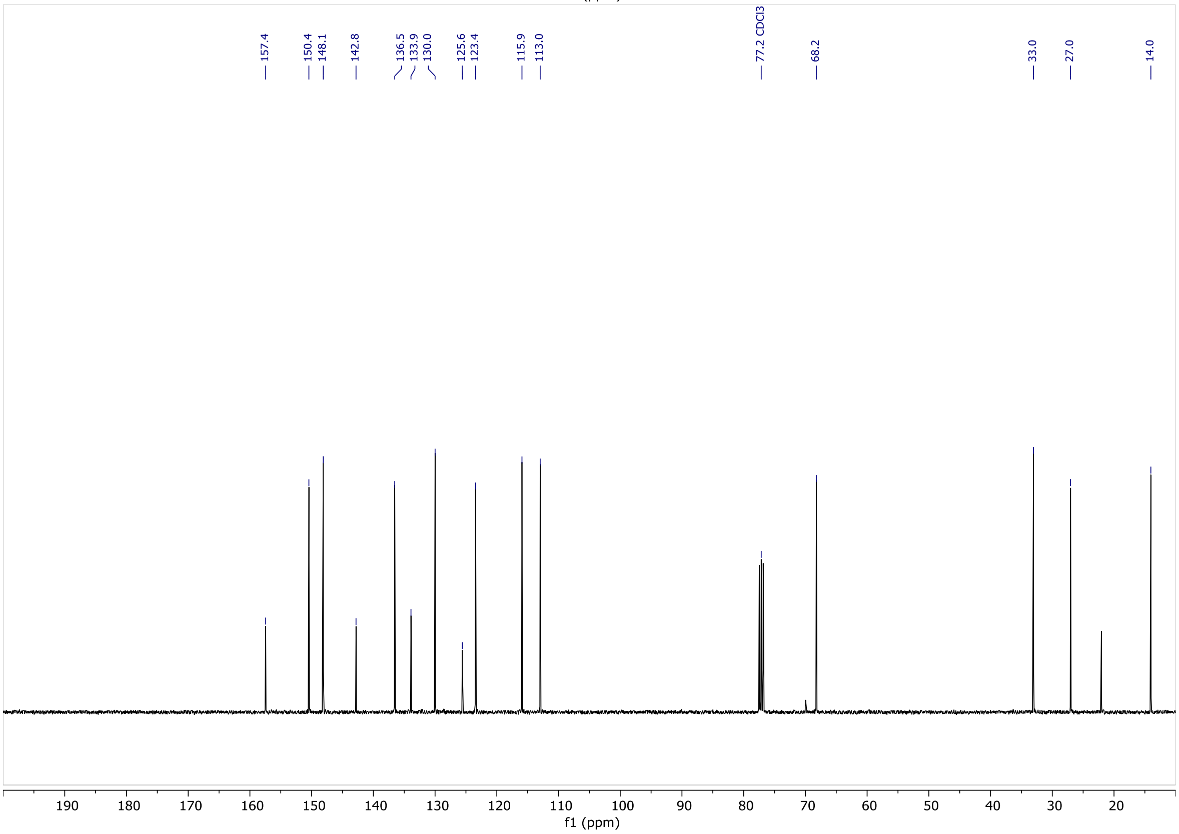

3-(2-(4-chloro-3-ethylphenoxy)ethyl)pyridine 1-oxide (39)

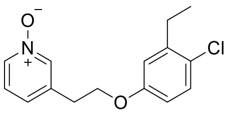

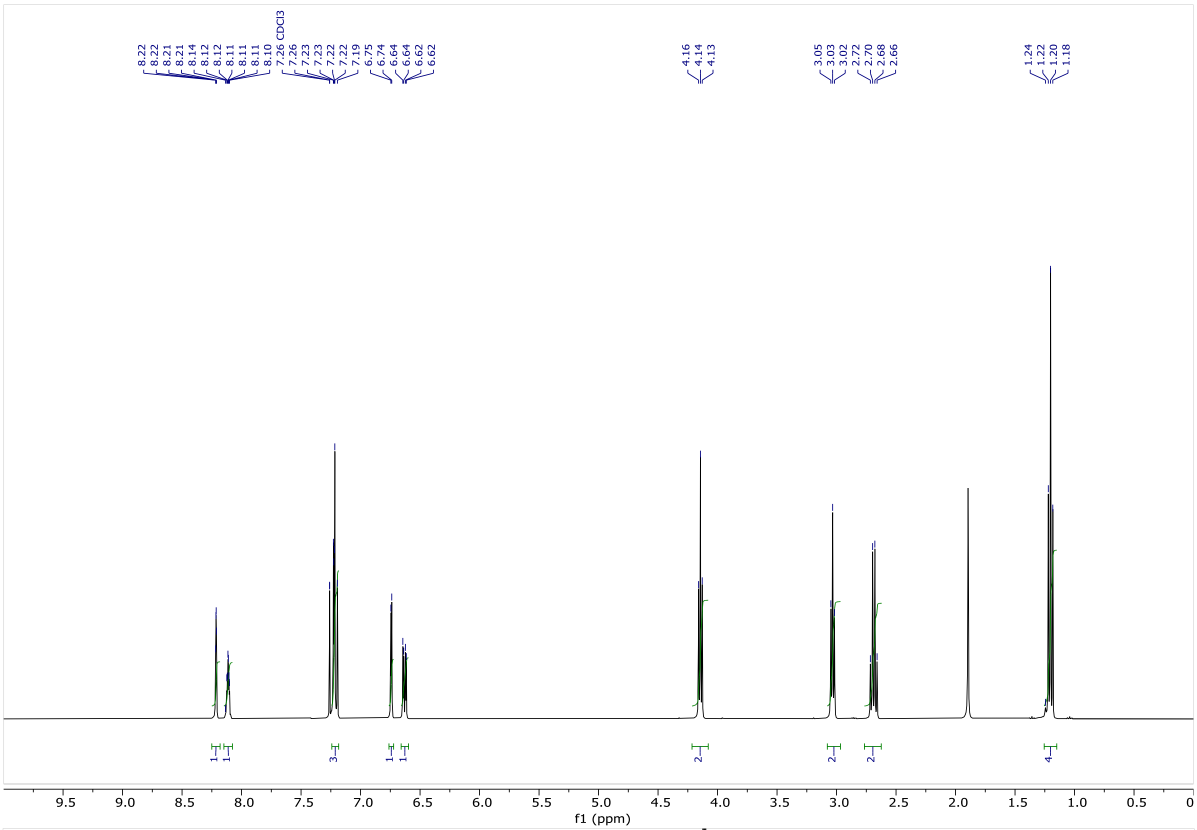

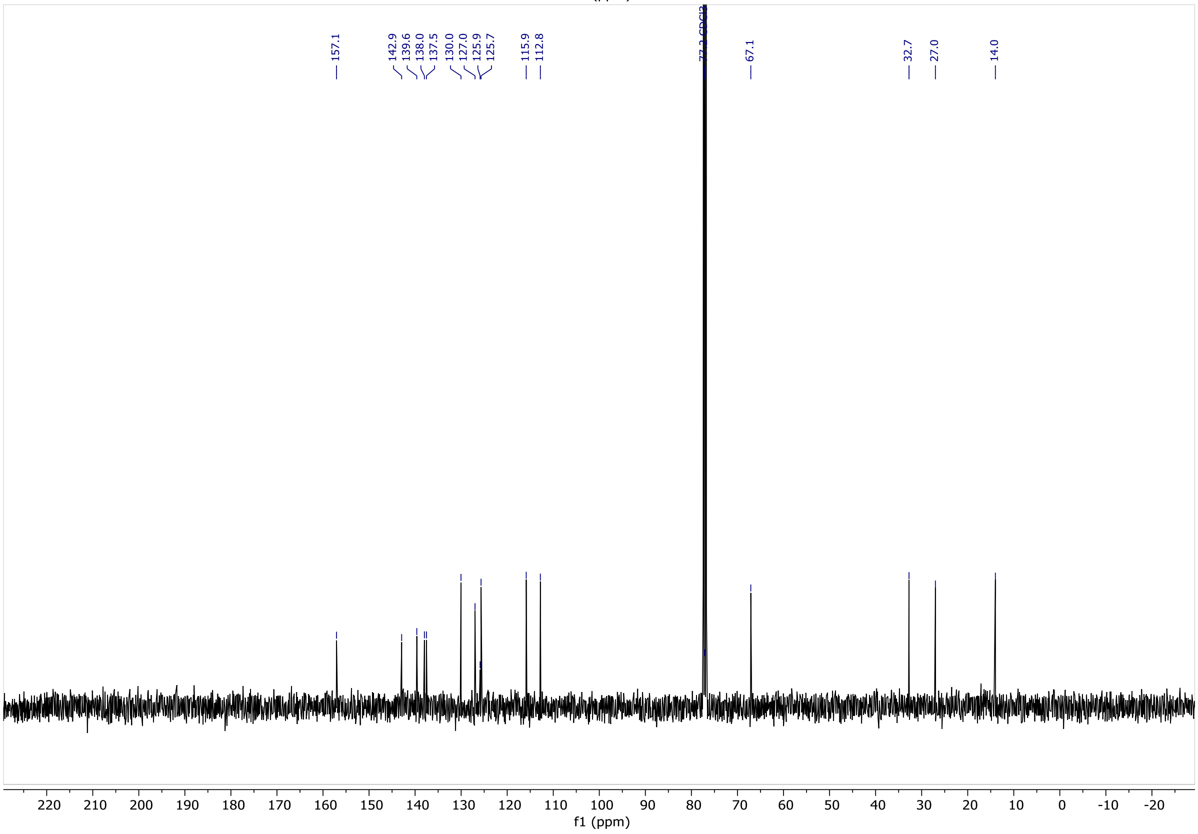

3-(2-(4-chloro-3-methoxyphenoxy)ethyl)pyridine (40a)

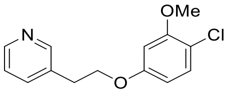

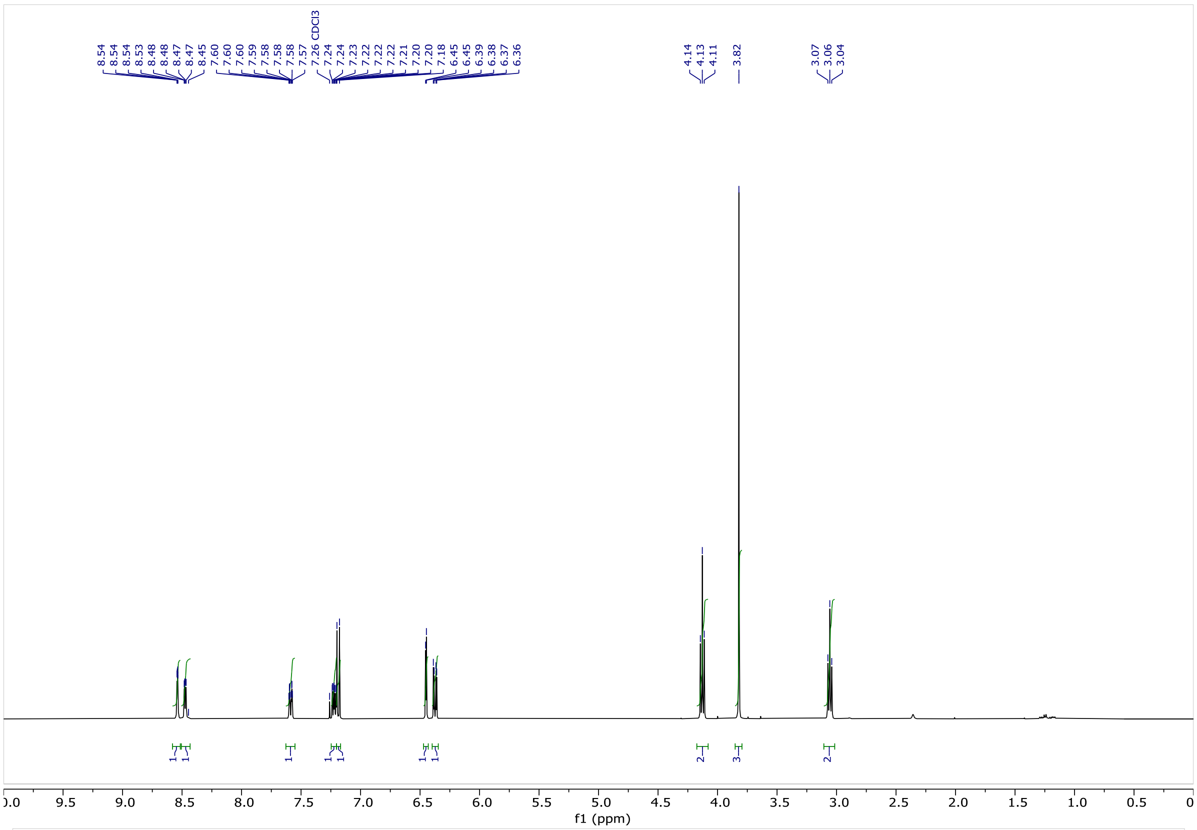

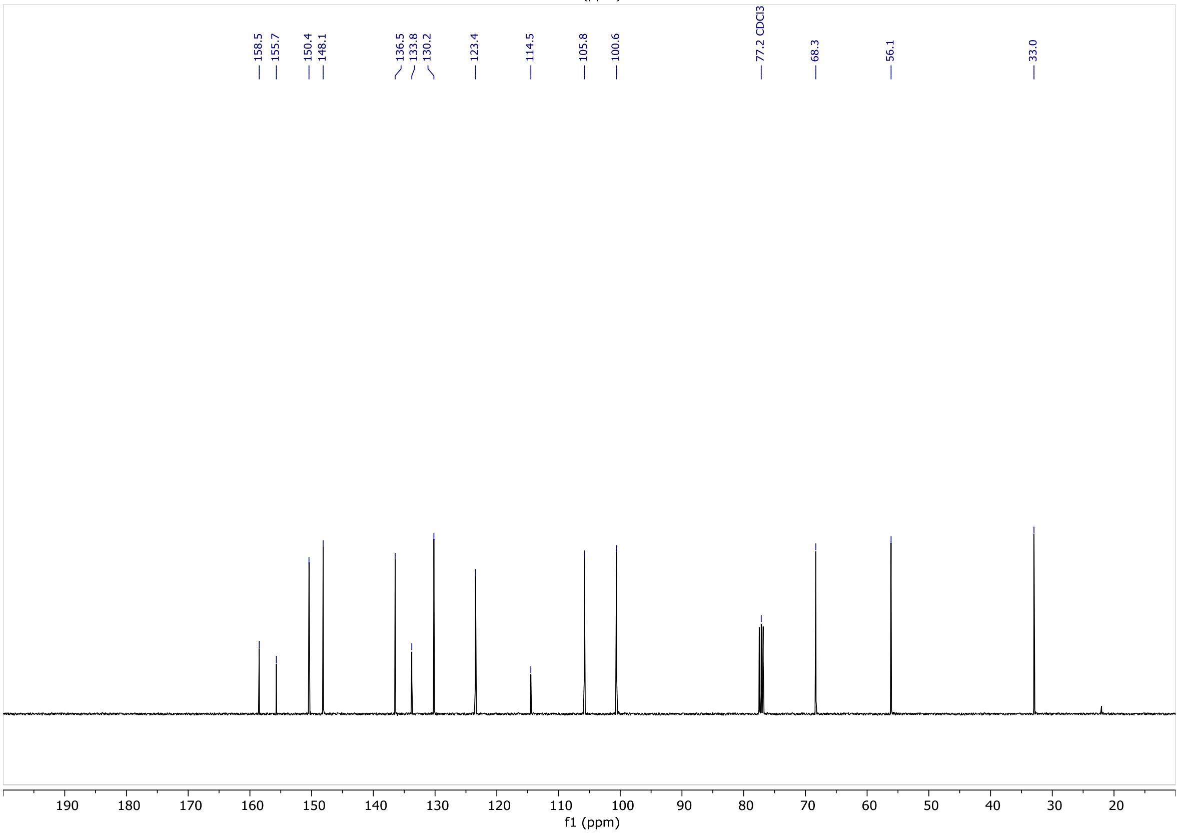

3-(2-(4-chloro-3-methoxyphenoxy)ethyl)pyridine 1-oxide (40)

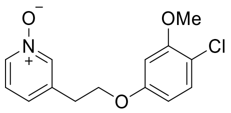

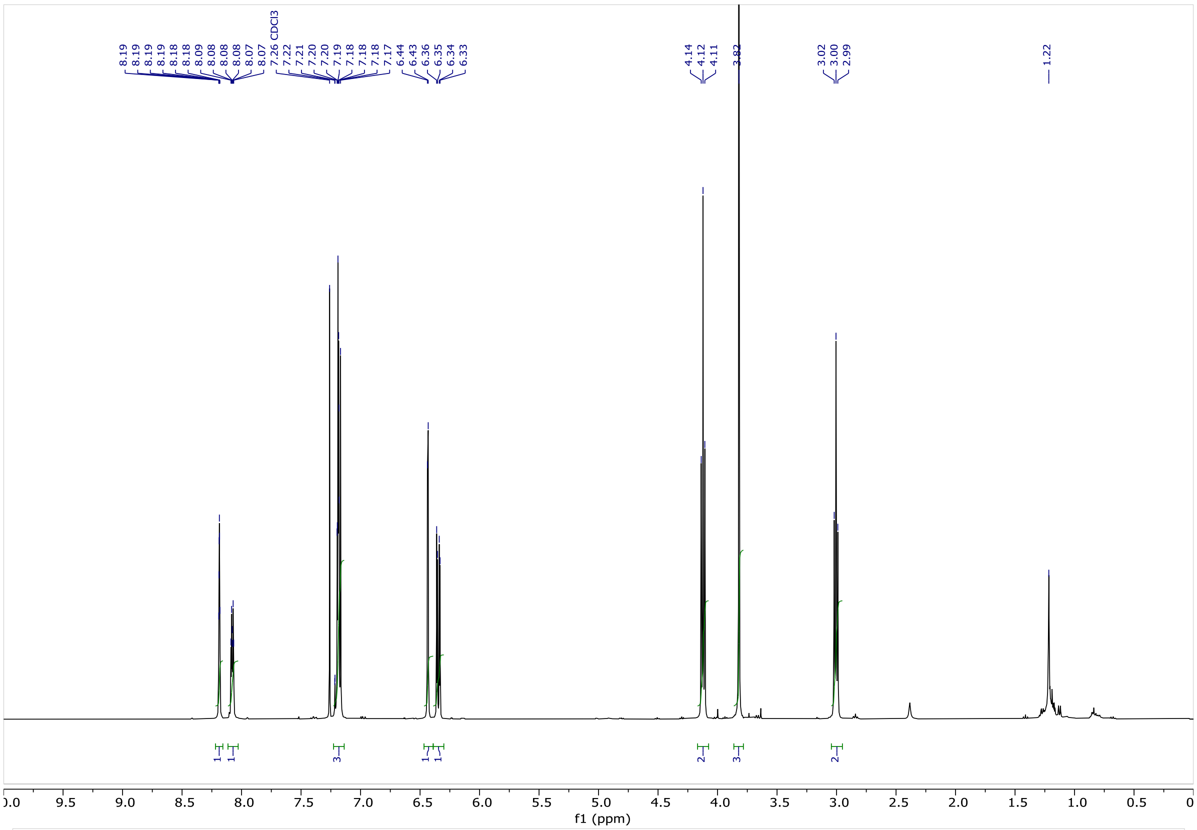

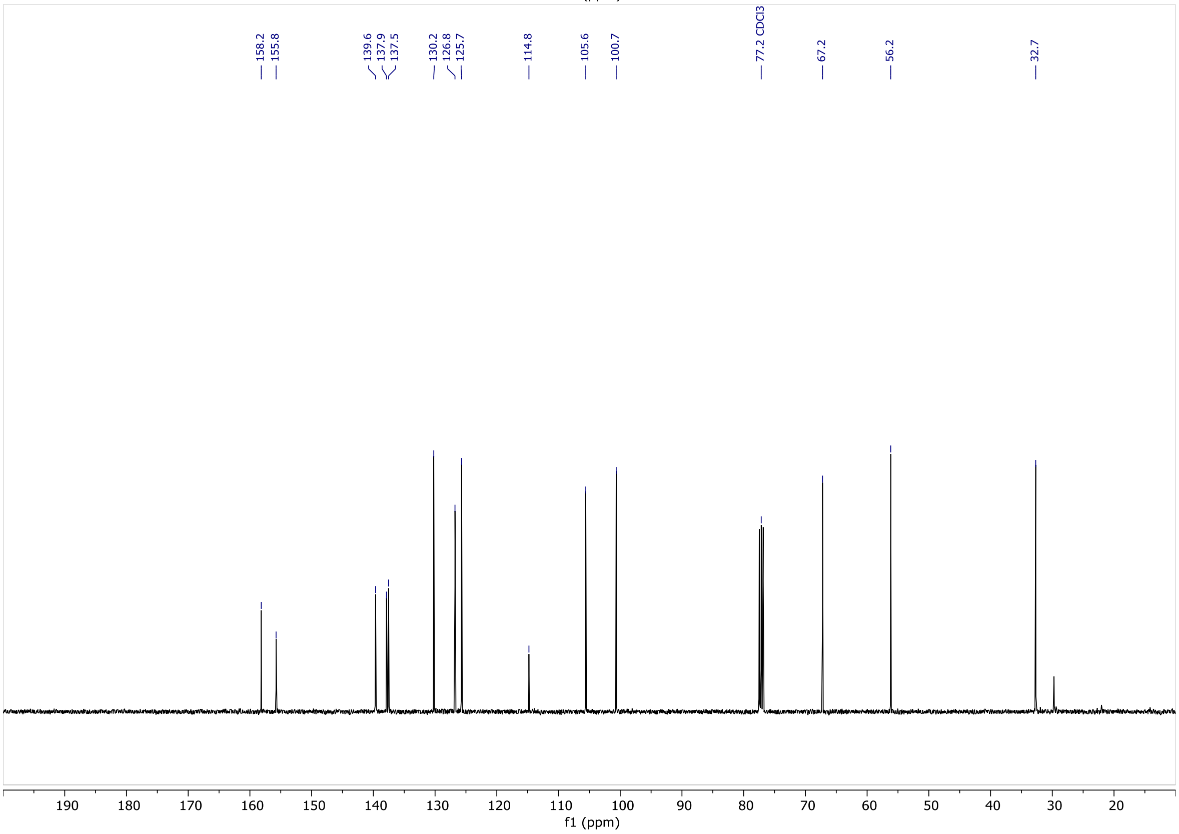

3-(2-(4-chloro-3-cyclopropylphenoxy)ethyl)pyridine (41a)

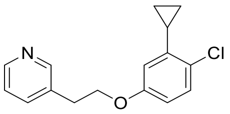

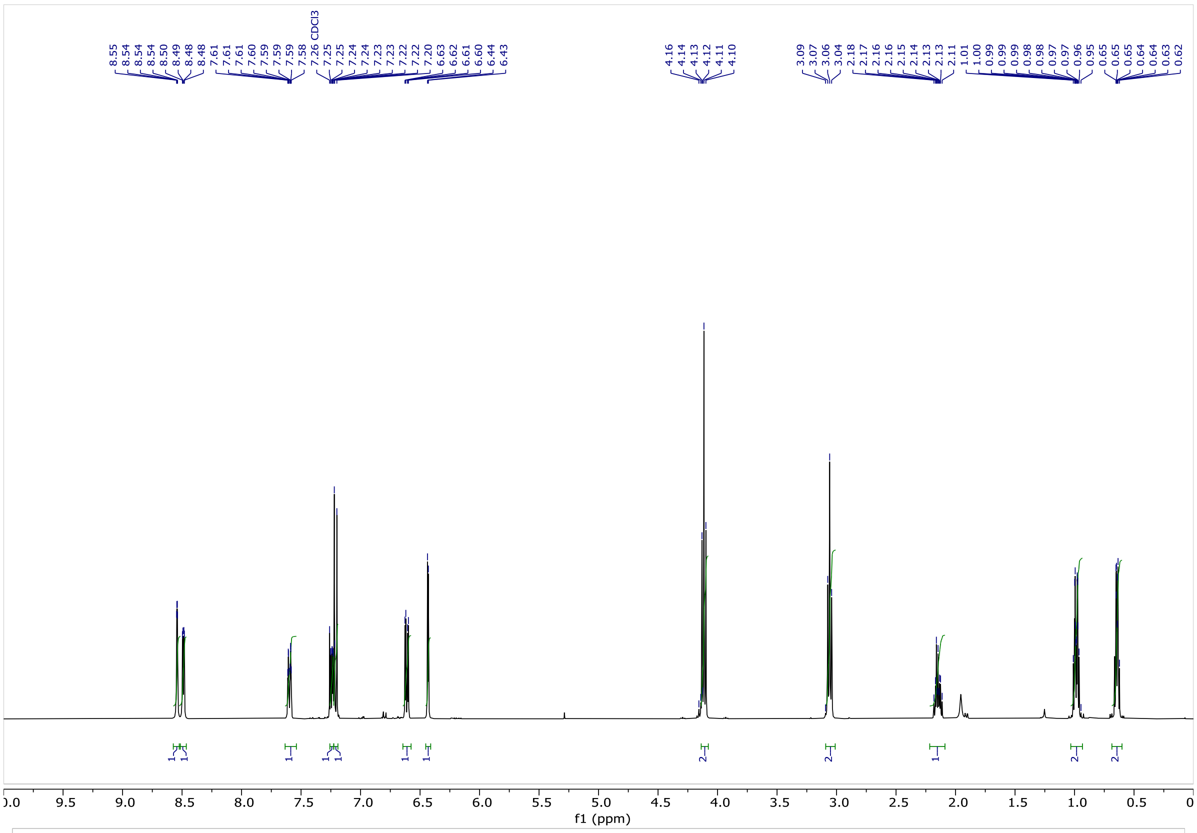

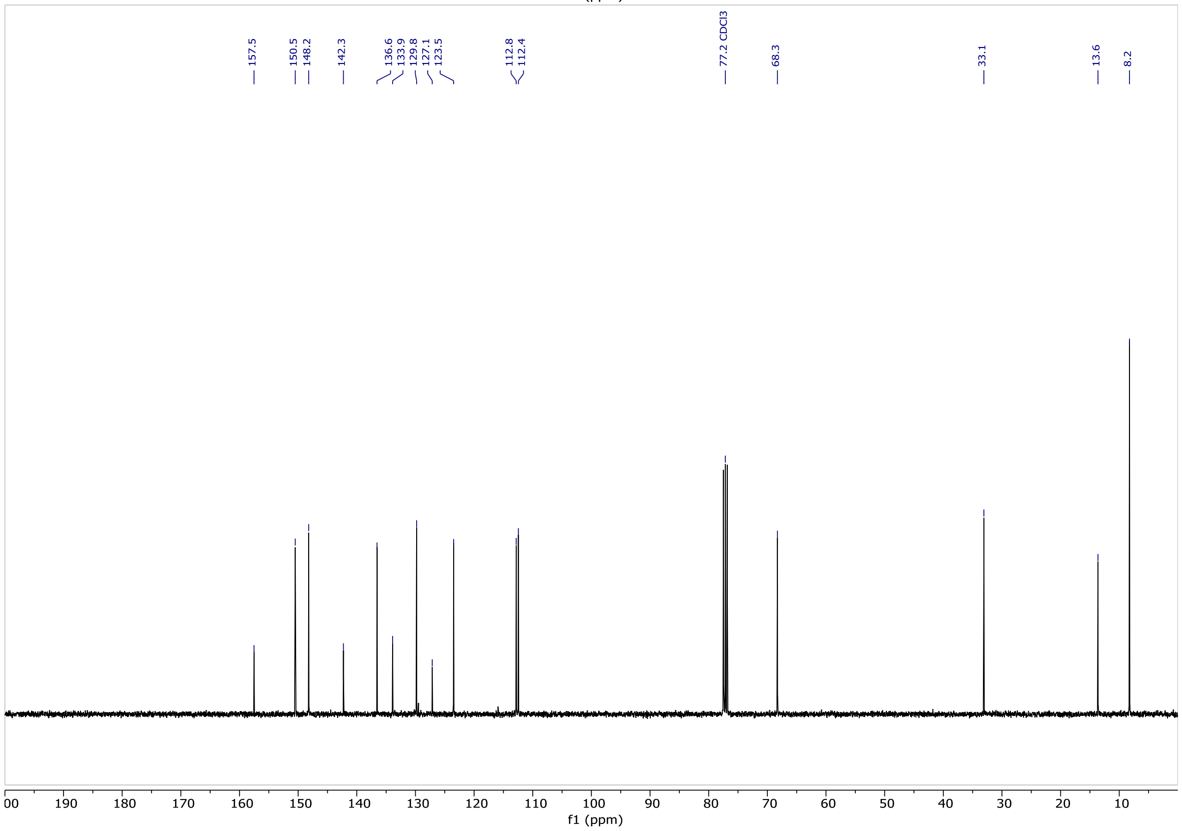

3-(2-(4-chloro-3-cyclopropylphenoxy)ethyl)pyridine 1-oxide (41)

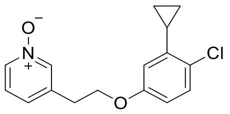

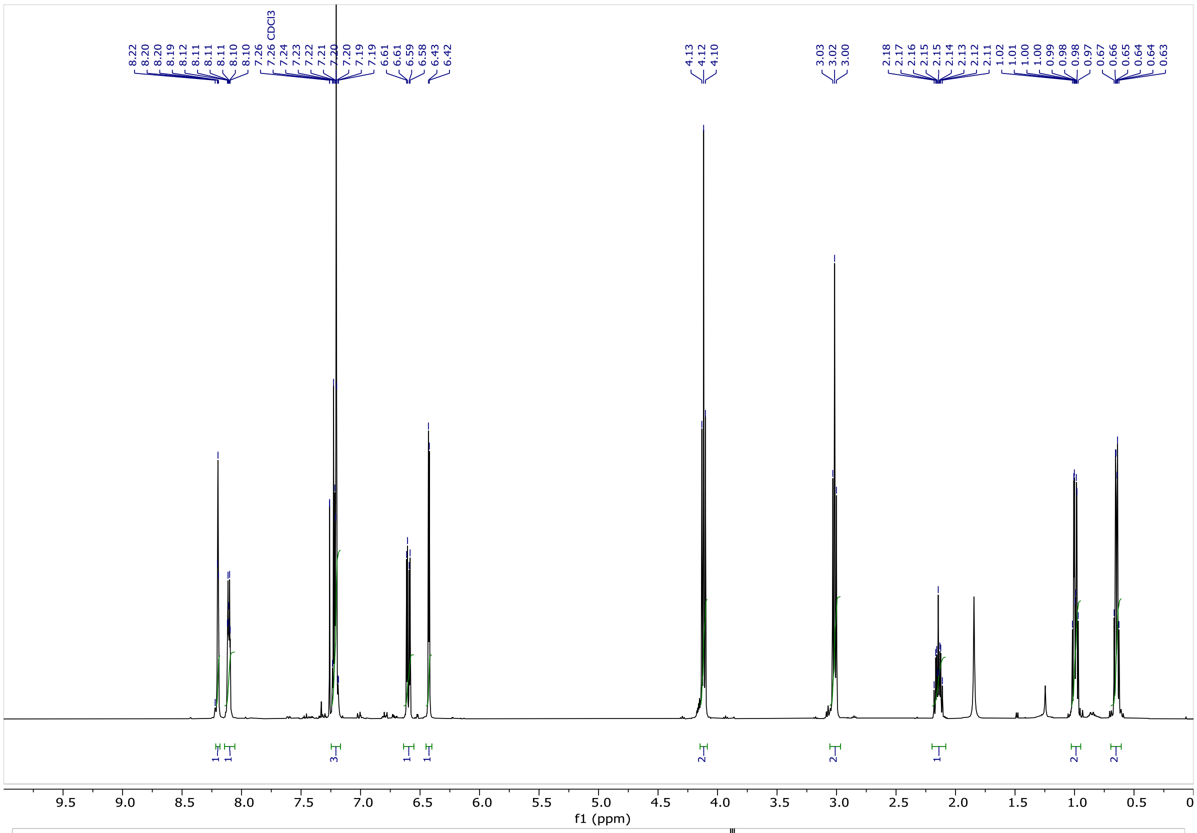

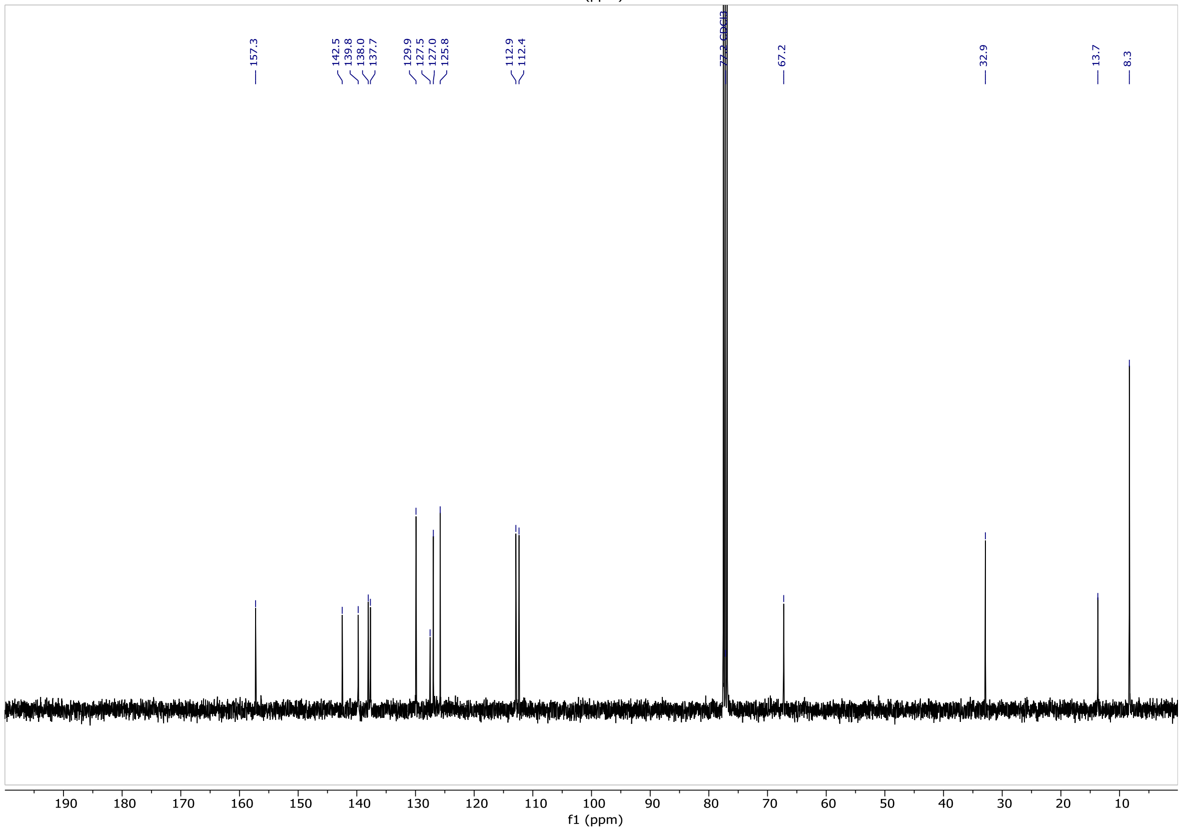

3-(2-(4-chloro-3-(trifluoromethyl)phenoxy)ethyl)pyridine (42a)

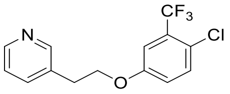

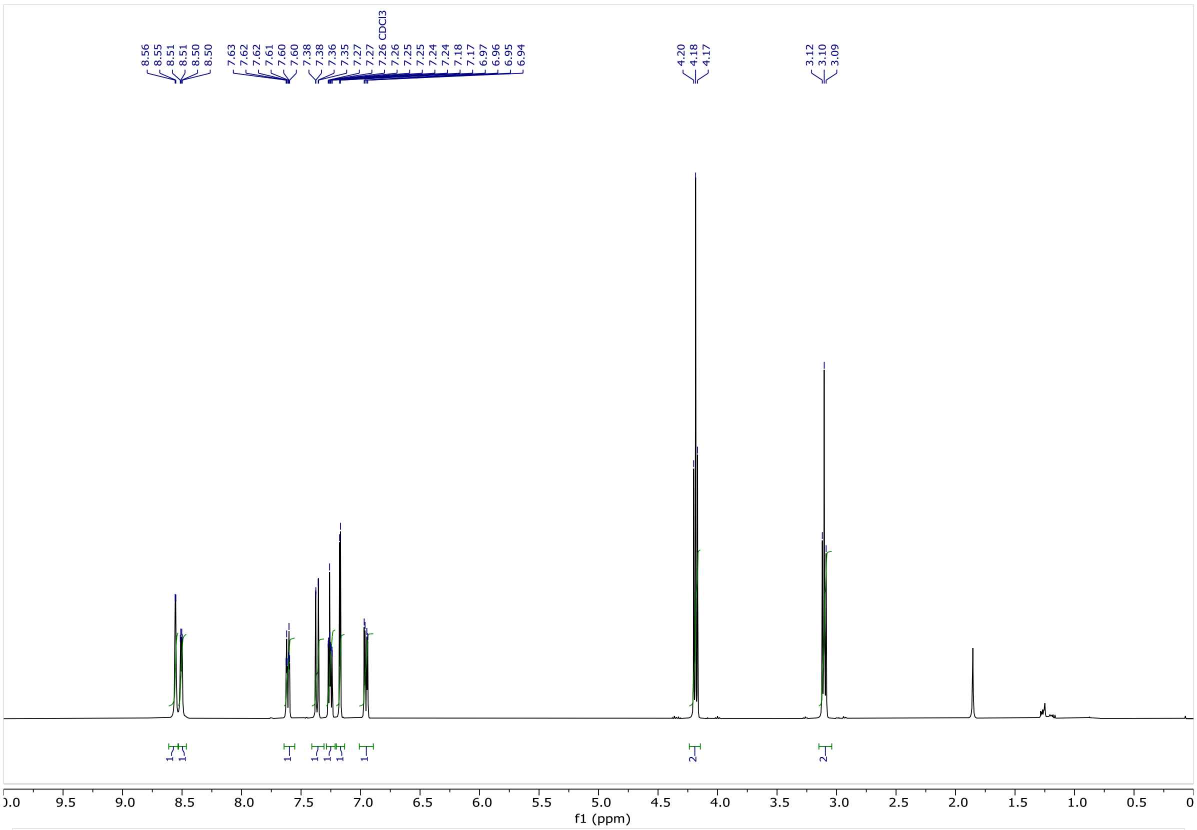

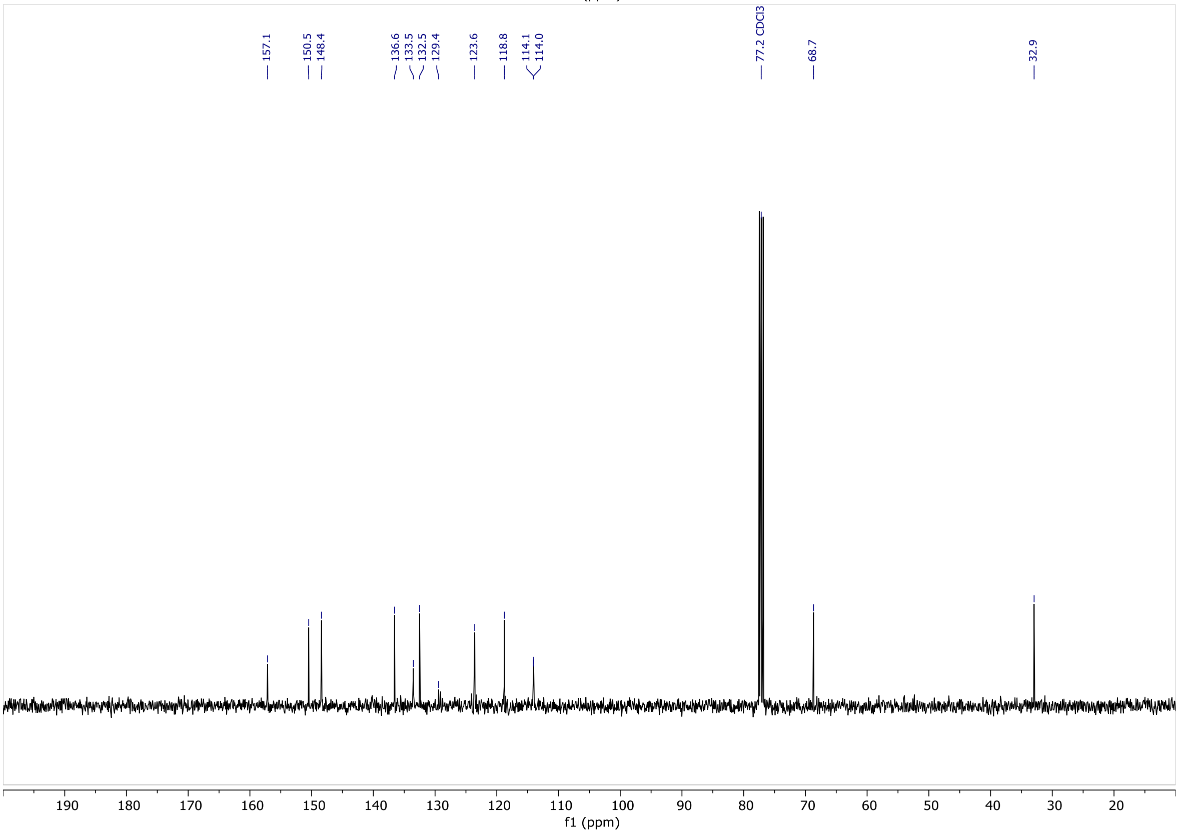

3-(2-(4-chloro-3-(trifluoromethyl)phenoxy)ethyl)pyridine 1-oxide (42)

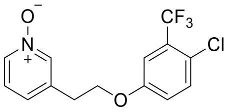

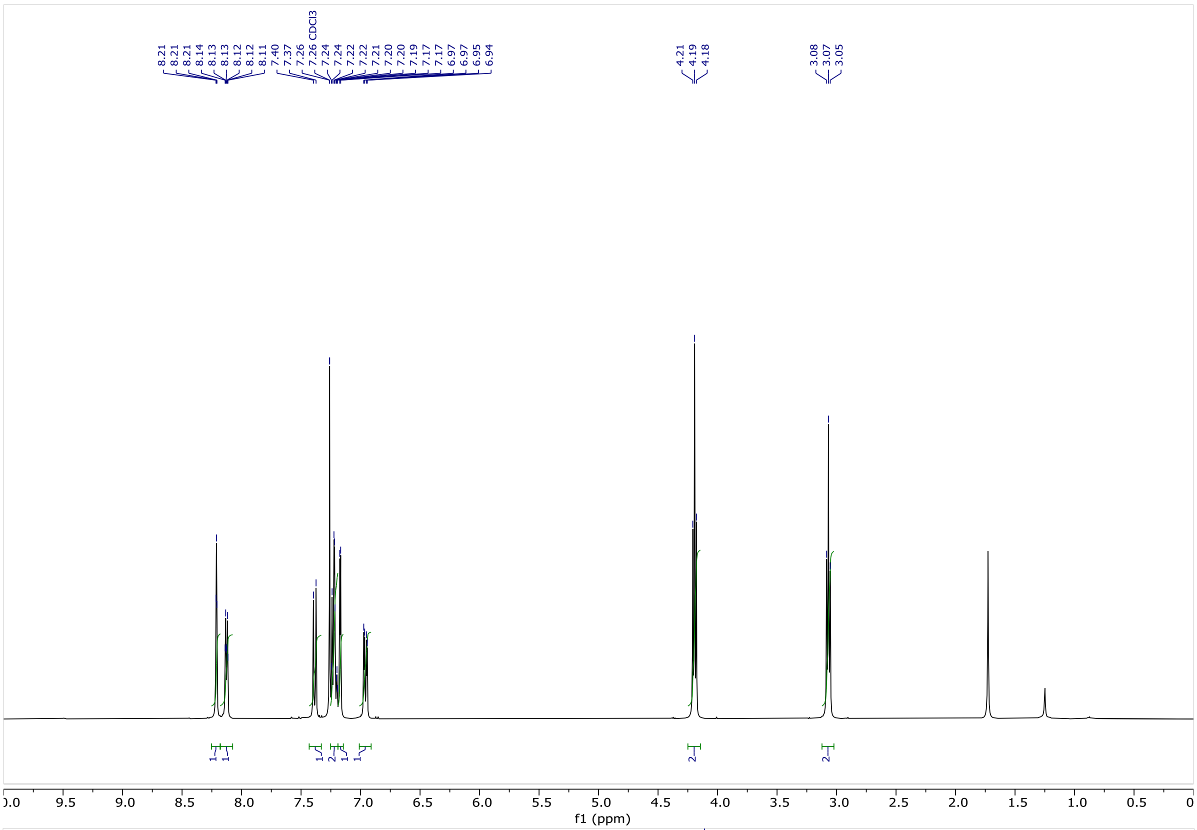

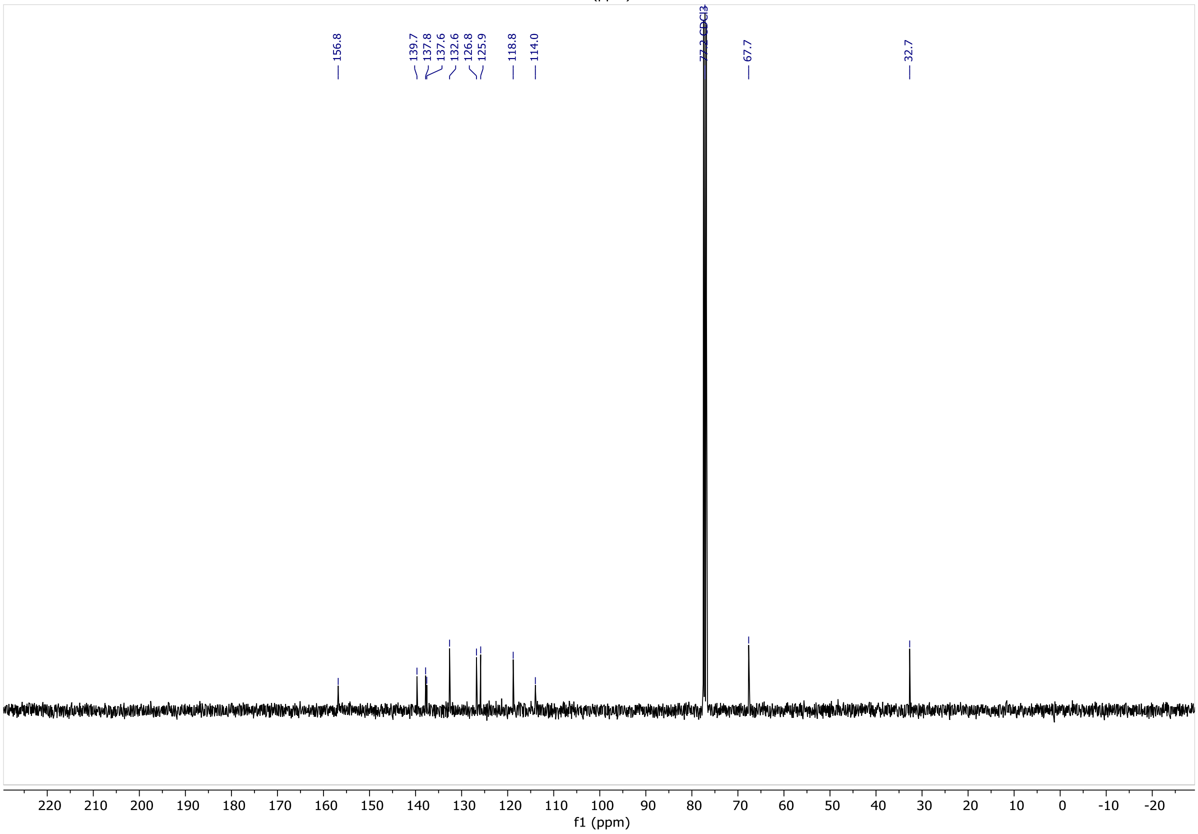

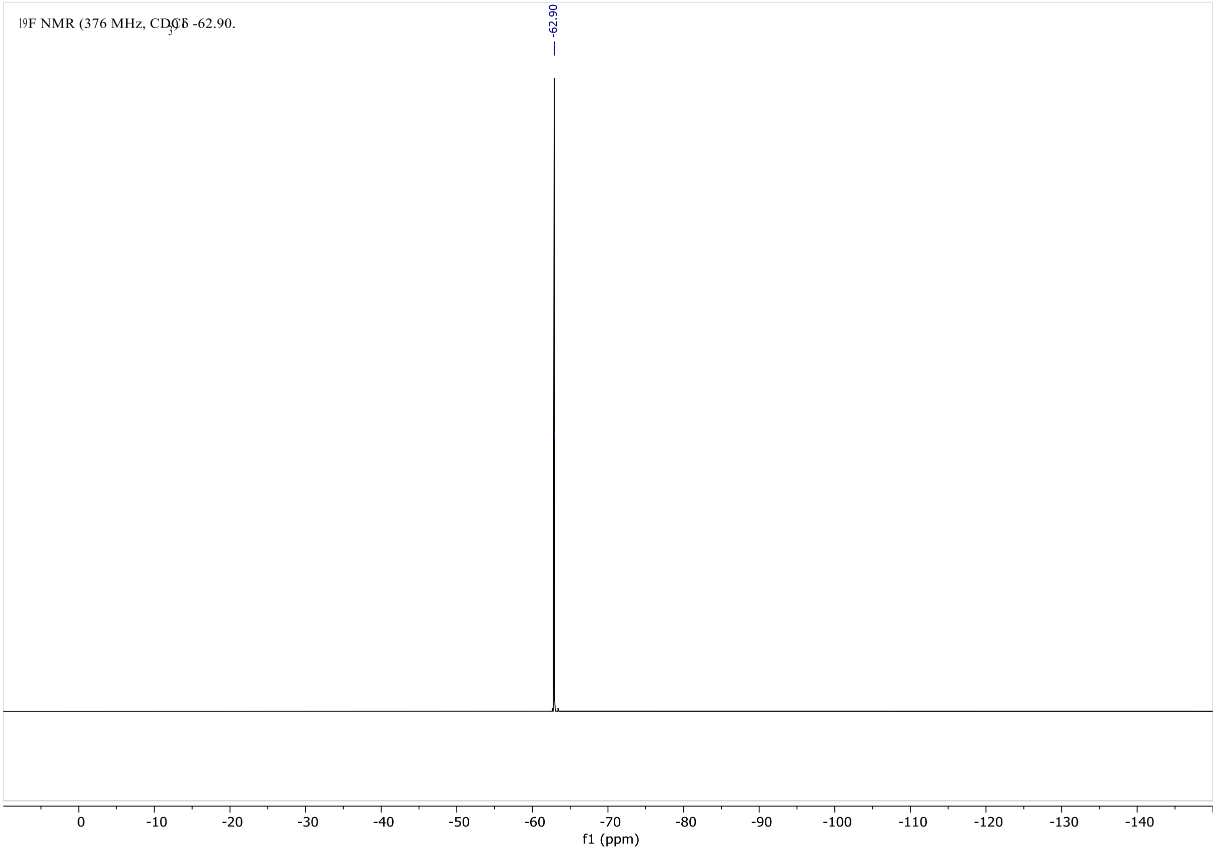
19F NMR (376 MHz, CDCl3) δ -62.90.
-62.90
0
-10
-20
-30
-40
-50
-60
-70
-80
-90
-100
-110
-120
-130
-140
f1 (ppm)

3-(2-(3,5-bis(trifluoromethyl)phenoxy)ethyl)pyridine (43a)

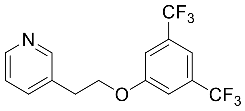

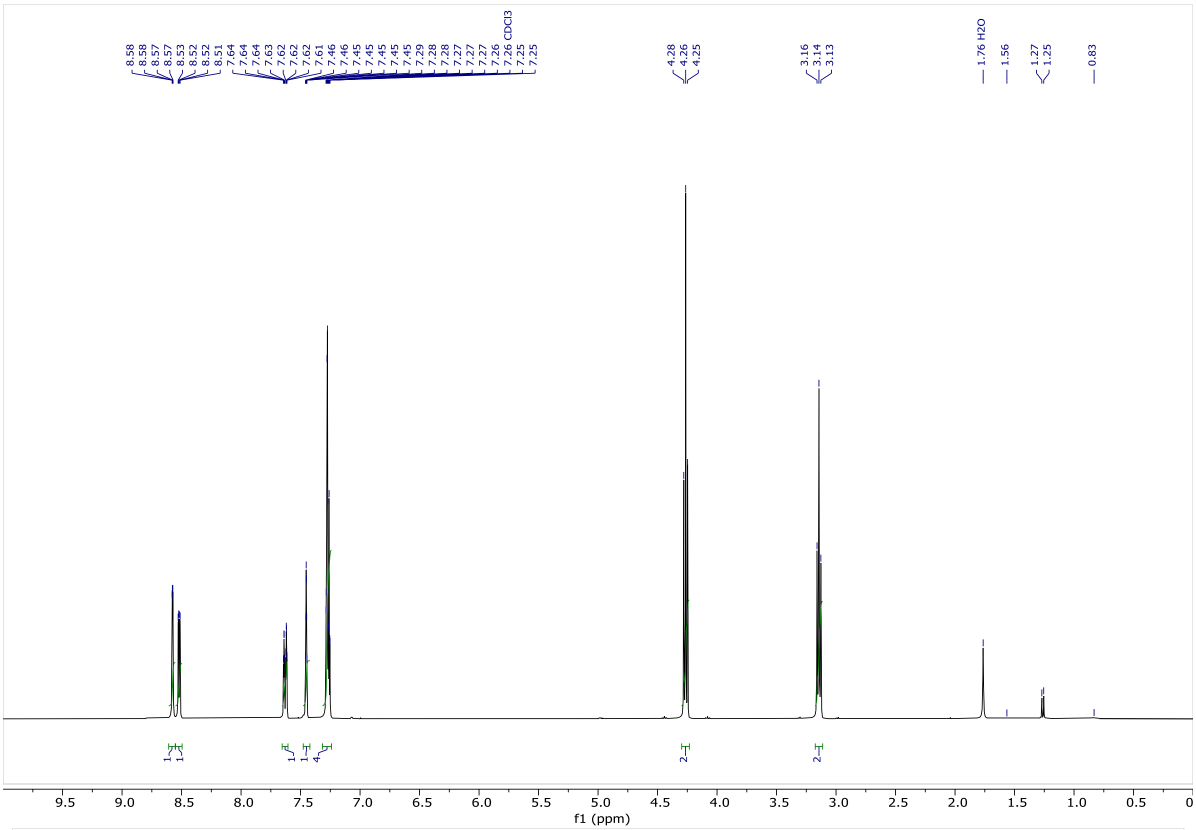

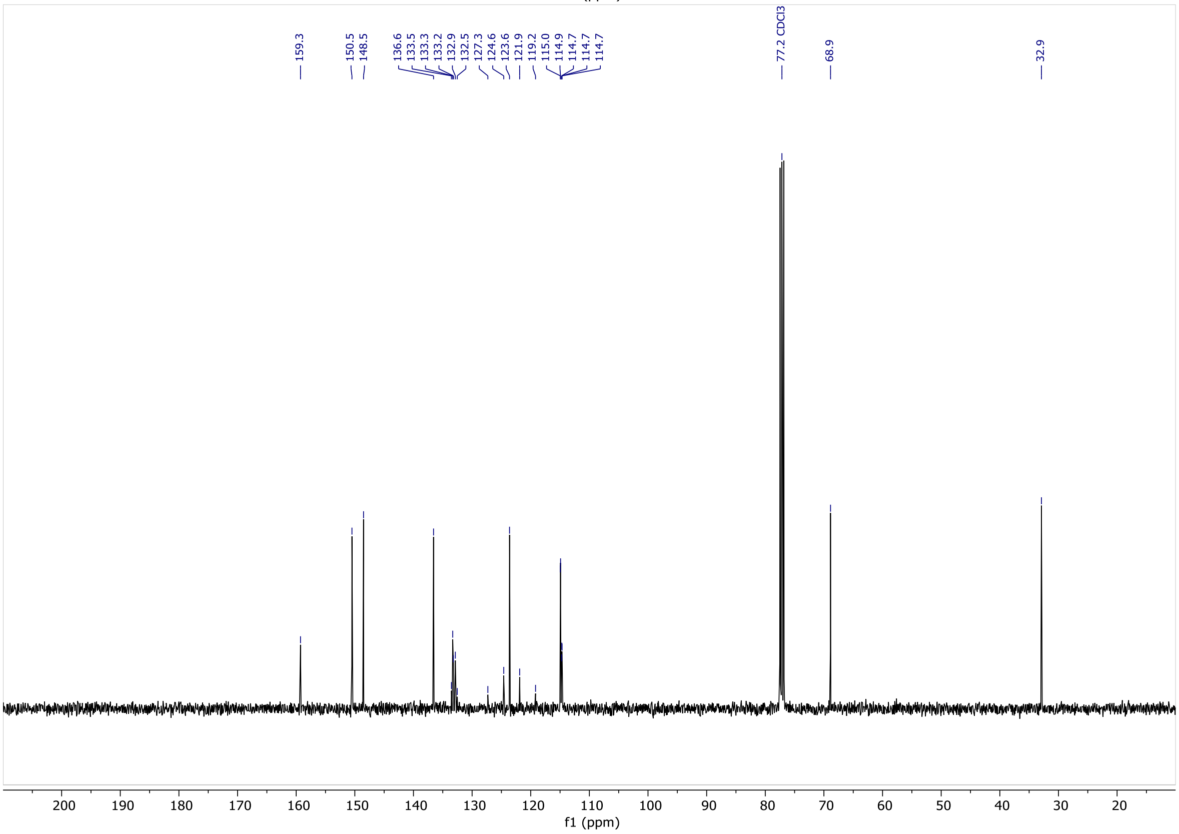

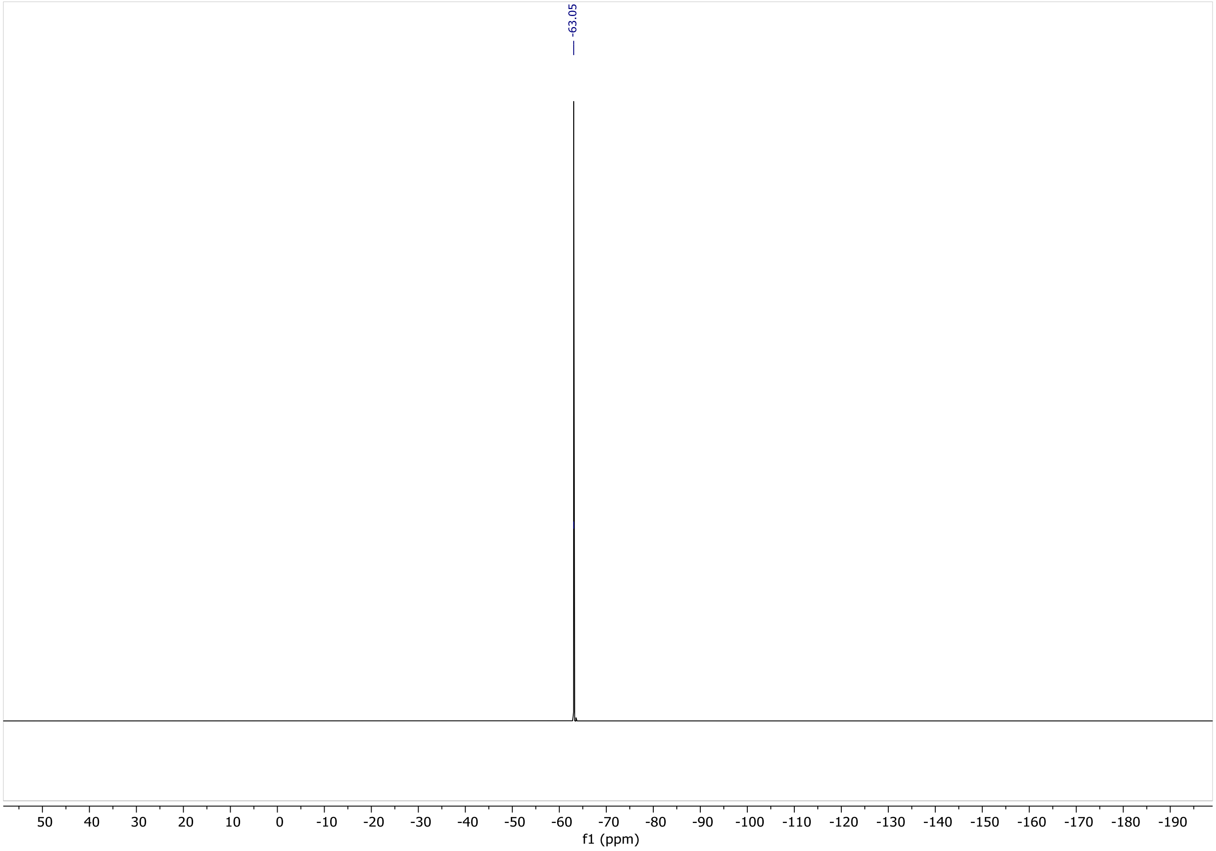
-63.05
50
40
30
20
10
0
-10
-20
-30
-40
-50
-60
-70
-80
-90
-100
-110
-120
-130
-140
-150
-160
-170
-180
-190
f1 (ppm)

3-(2-(3,5-bis(trifluoromethyl)phenoxy)ethyl)pyridine 1-oxide (43)

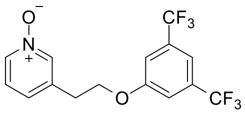

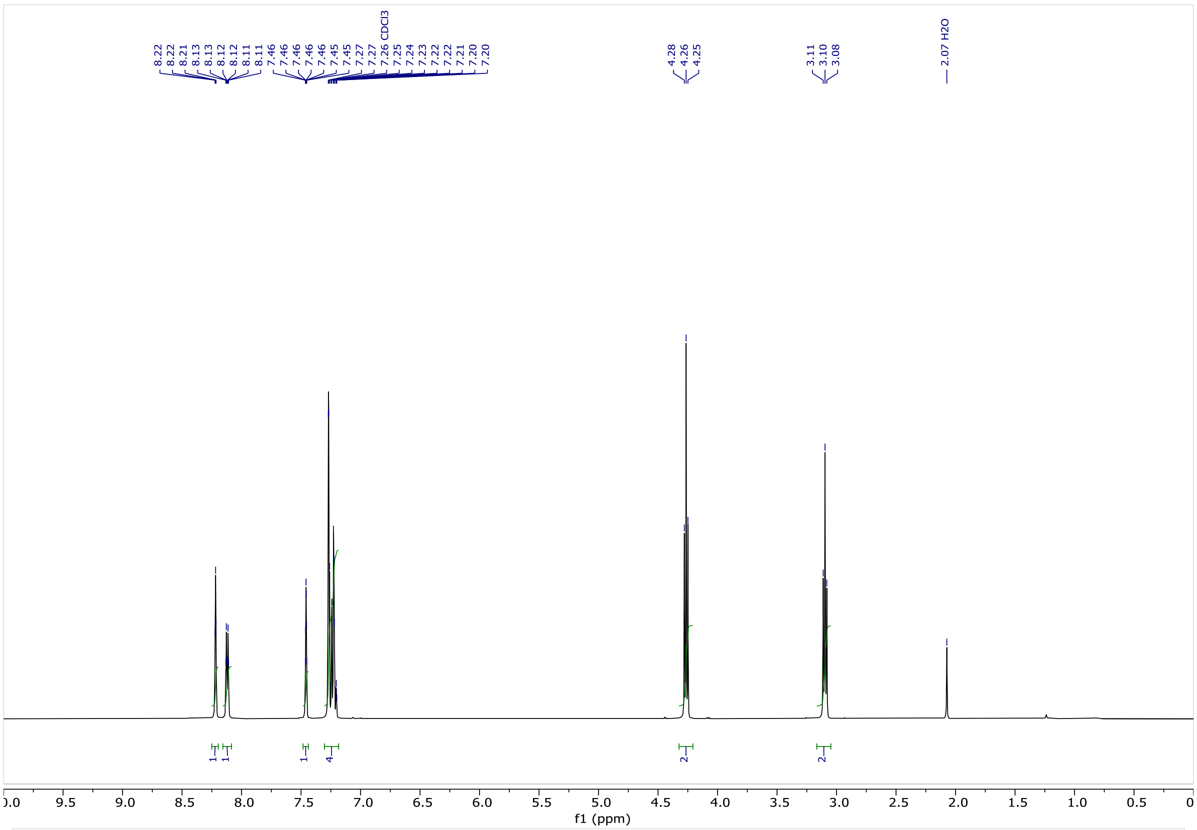

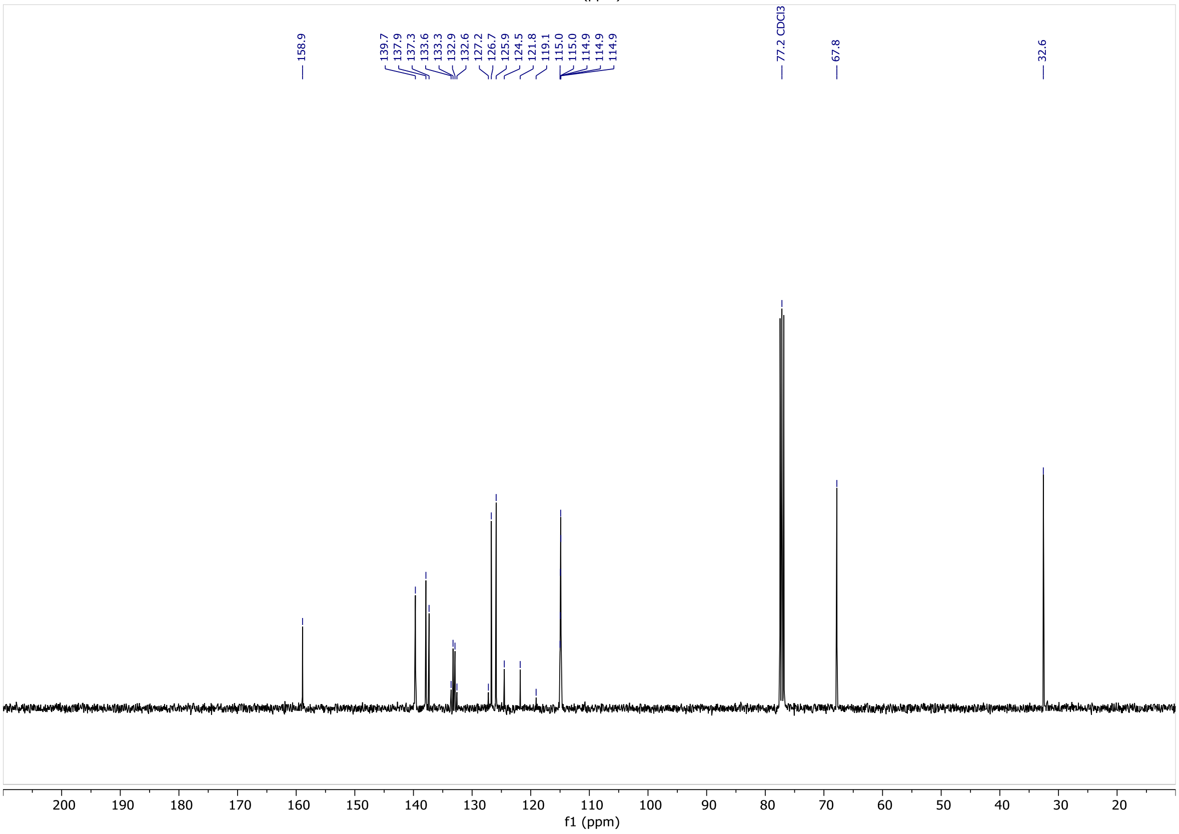

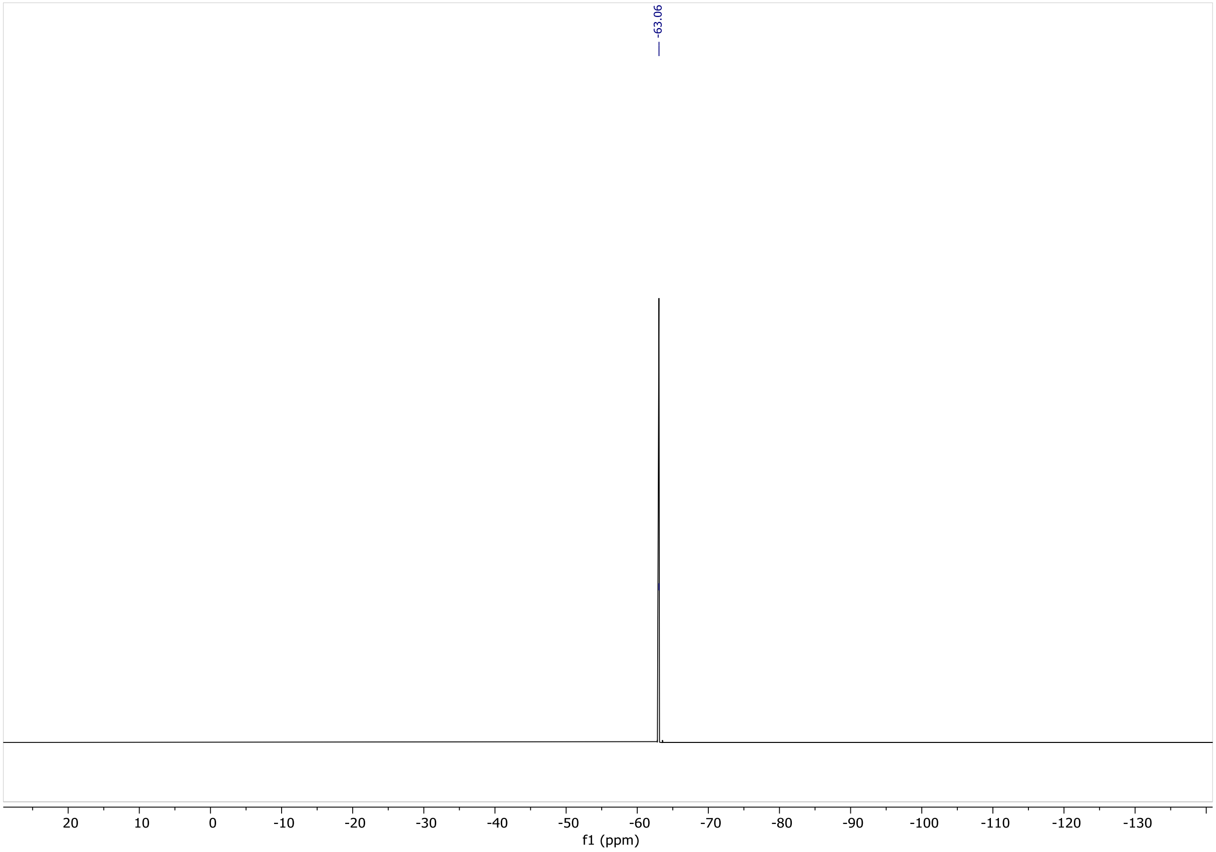
-63.06
20
10
0
-10
-20
-30
-40
-50
-60
-70
-80
-90
-100
-110
-120
-130
f1 (ppm)

3-(((4-chloronaphthalen-1-yl)oxy)methyl)pyridine (44a)

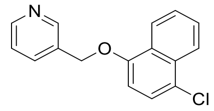

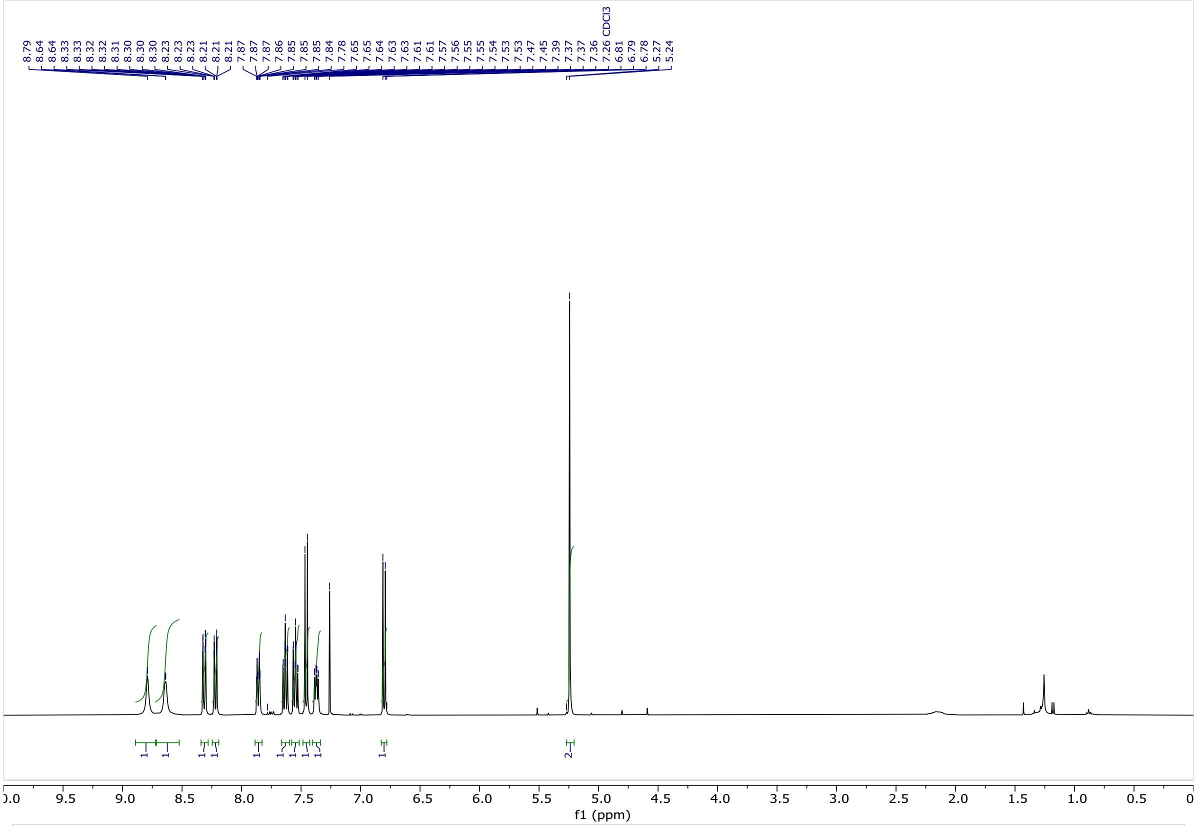

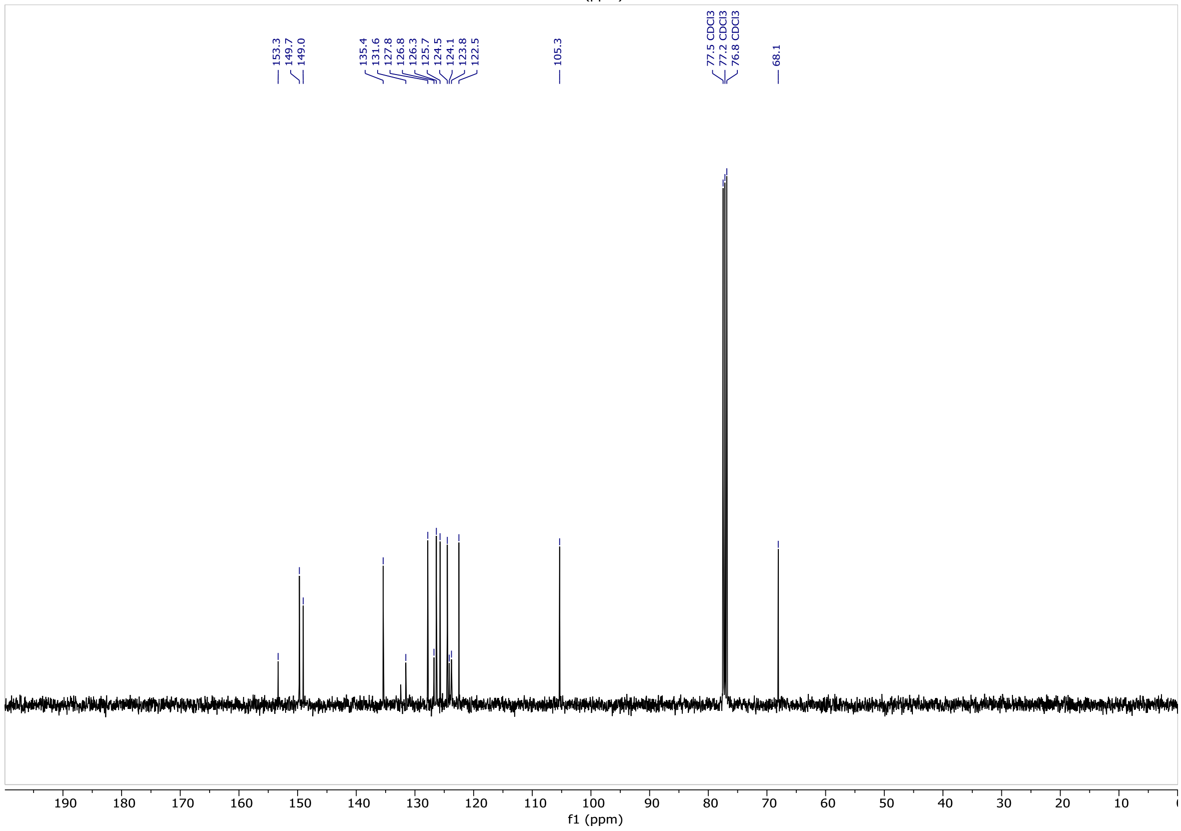

3-(((4-chloronaphthalen-1-yl)oxy)methyl)pyridine 1-oxide (44)

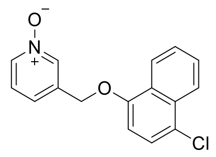

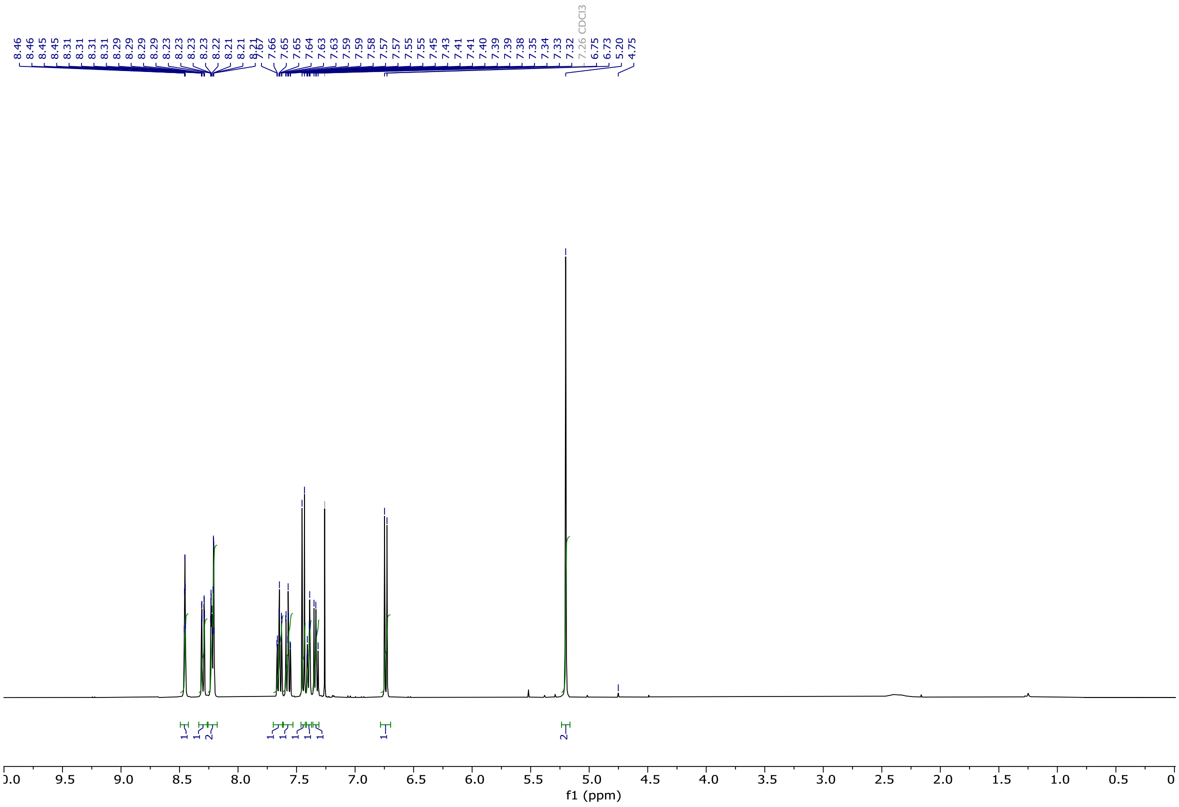

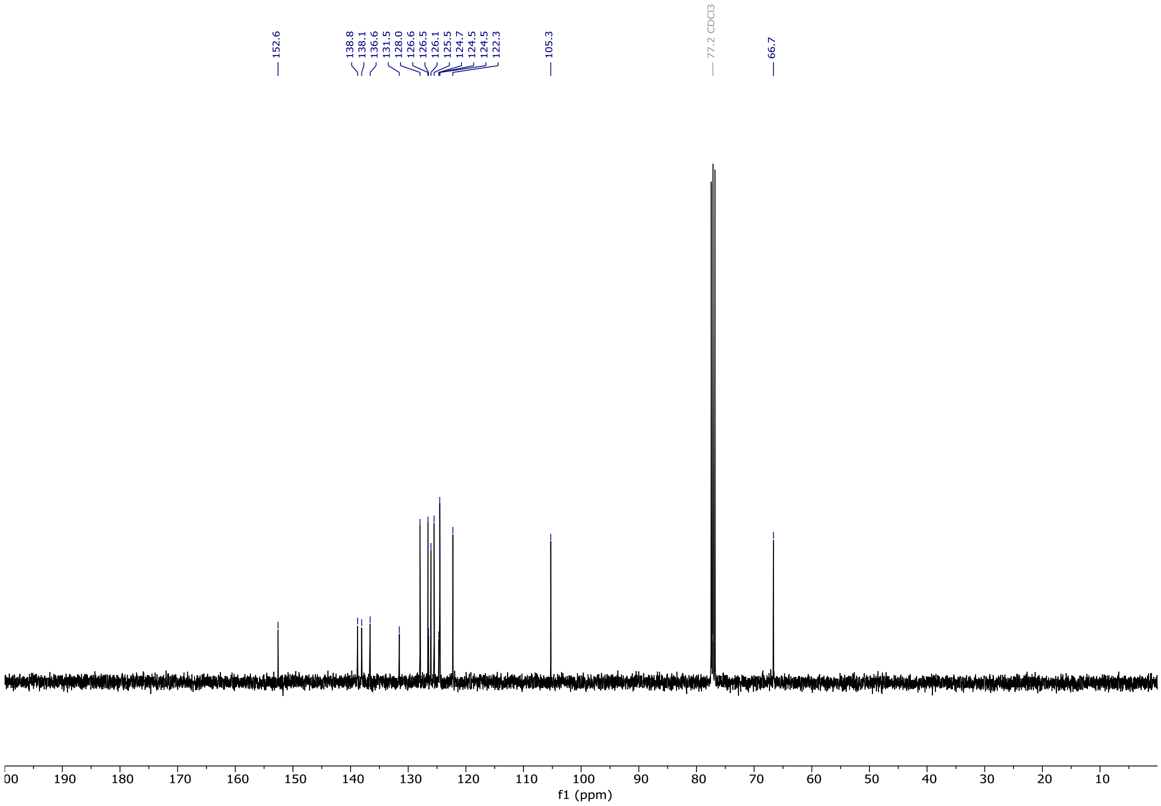

3-(3-((4-chloronaphthalen-1-yl)oxy)propyl)pyridine 1-oxide (45)

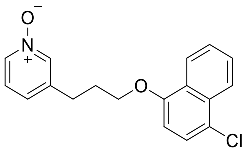

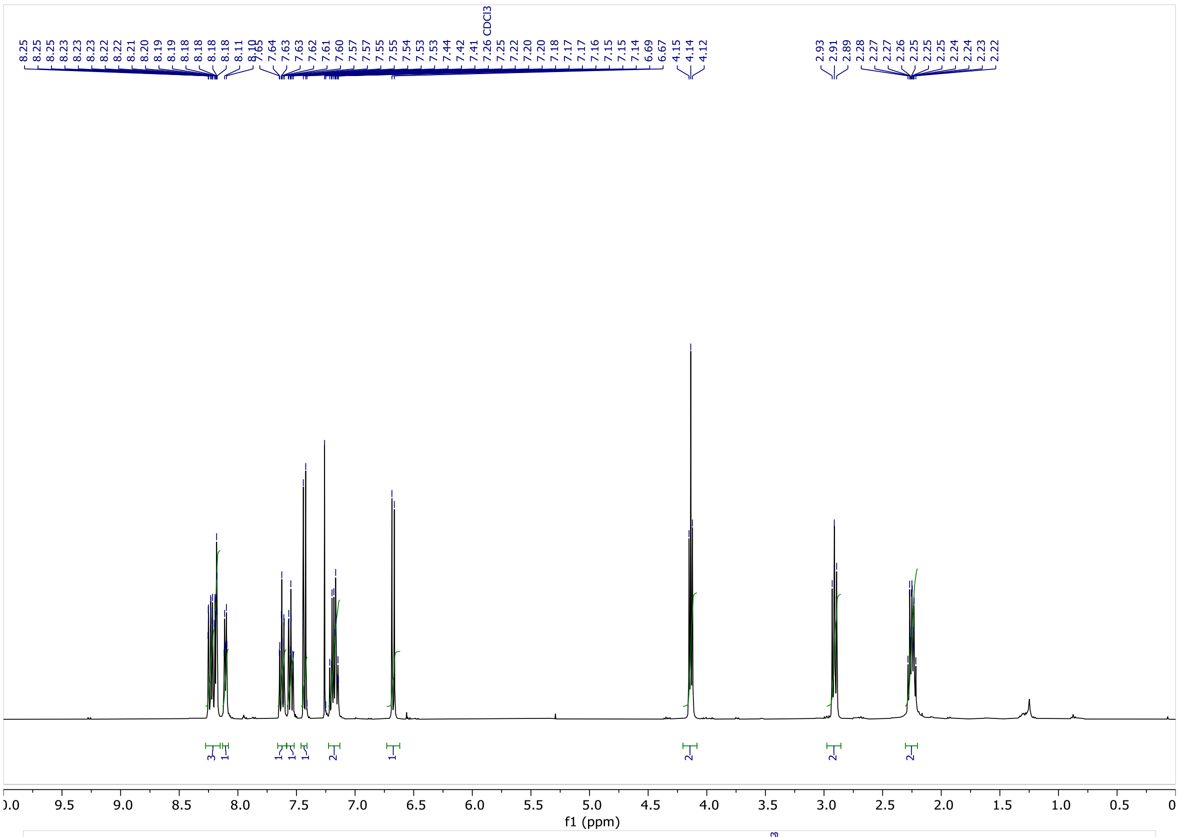

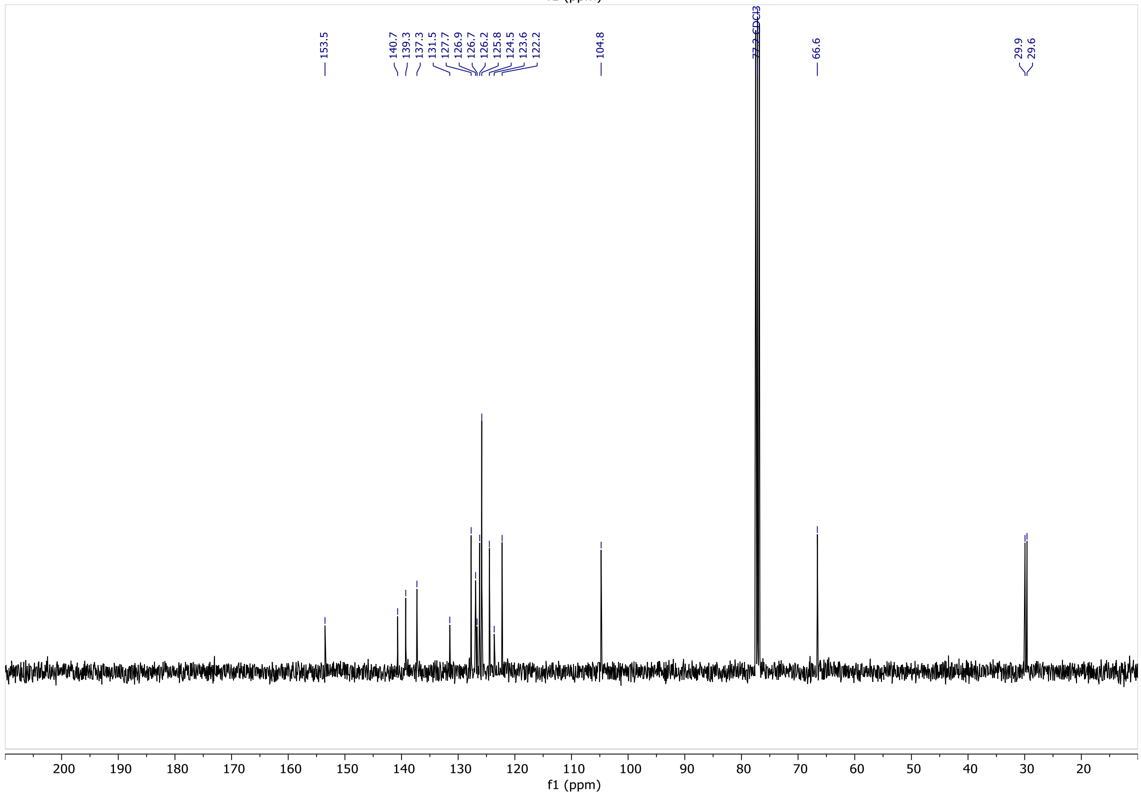

(*E*)-3-(naphthalen-1-yl)-1-(pyridin-3-yl)prop-2-en-1-one (46c)

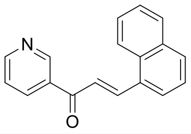

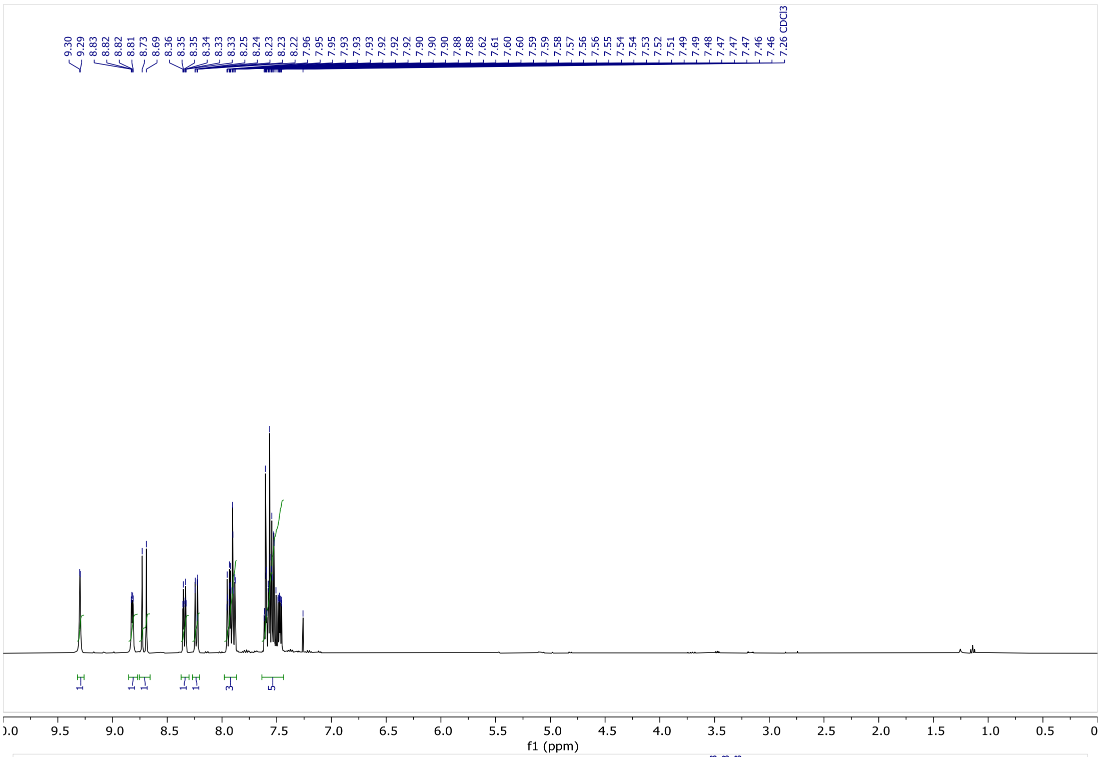

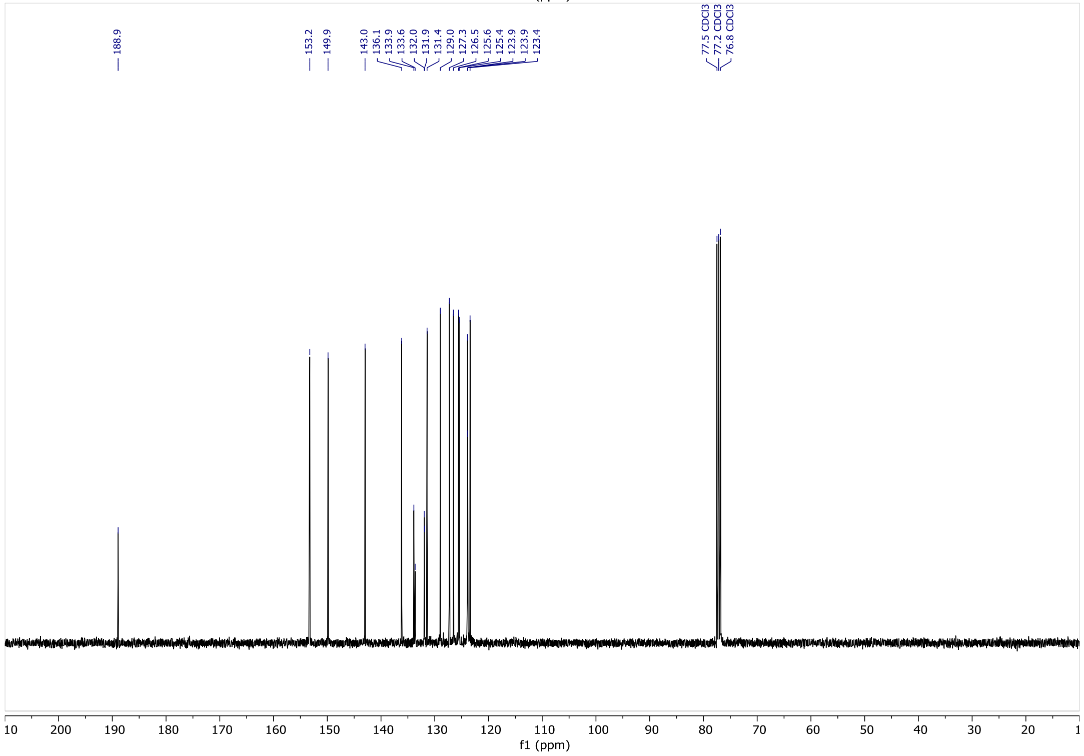

3-(naphthalen-1-yl)-1-(pyridin-3-yl)propan-1-one (46b)

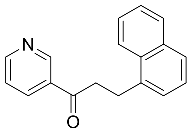

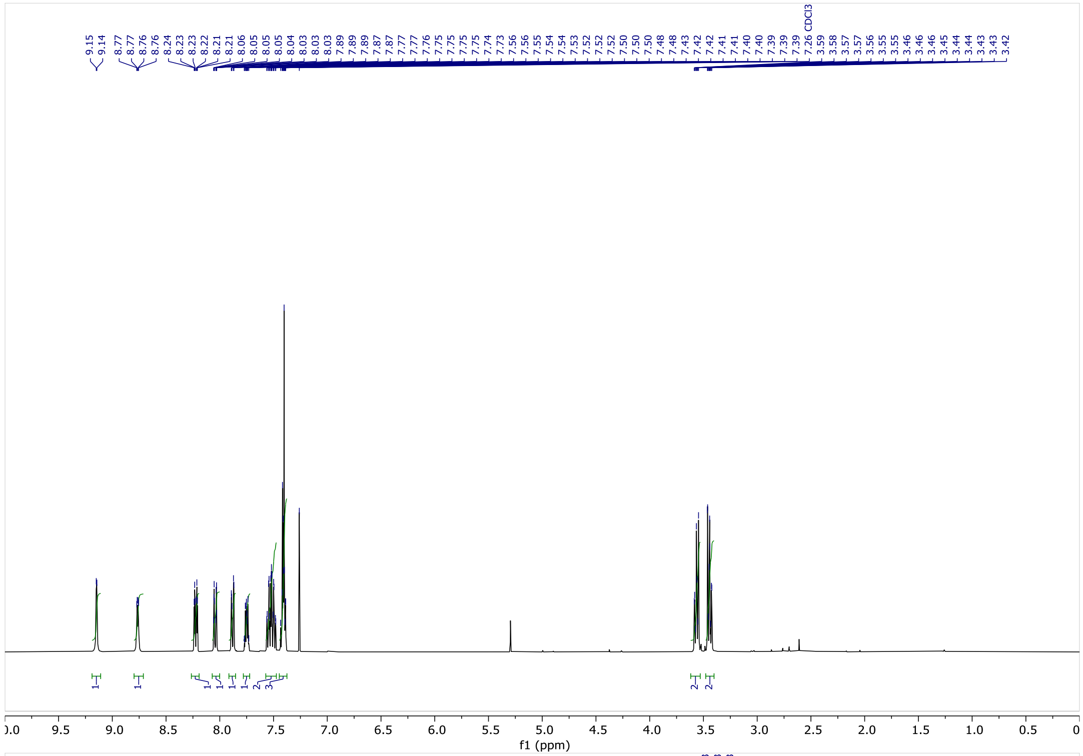

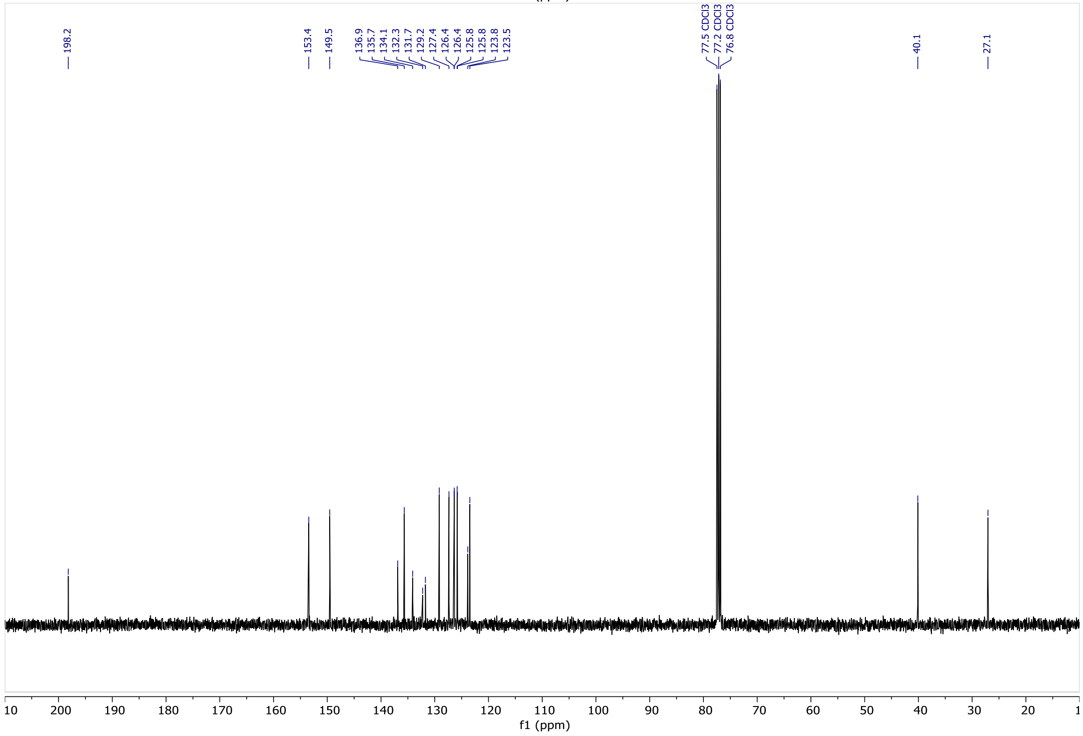

3-(3-(naphthalen-1-yl)propyl)pyridine (46a)

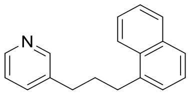

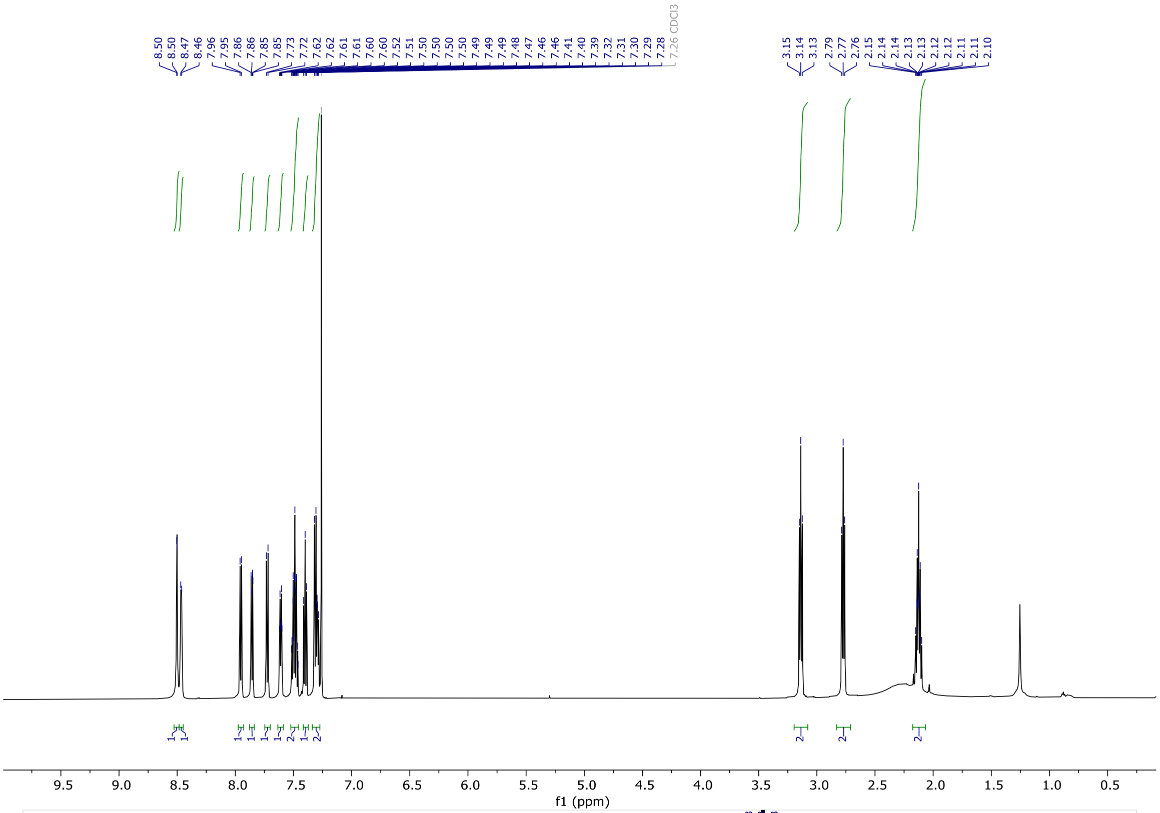

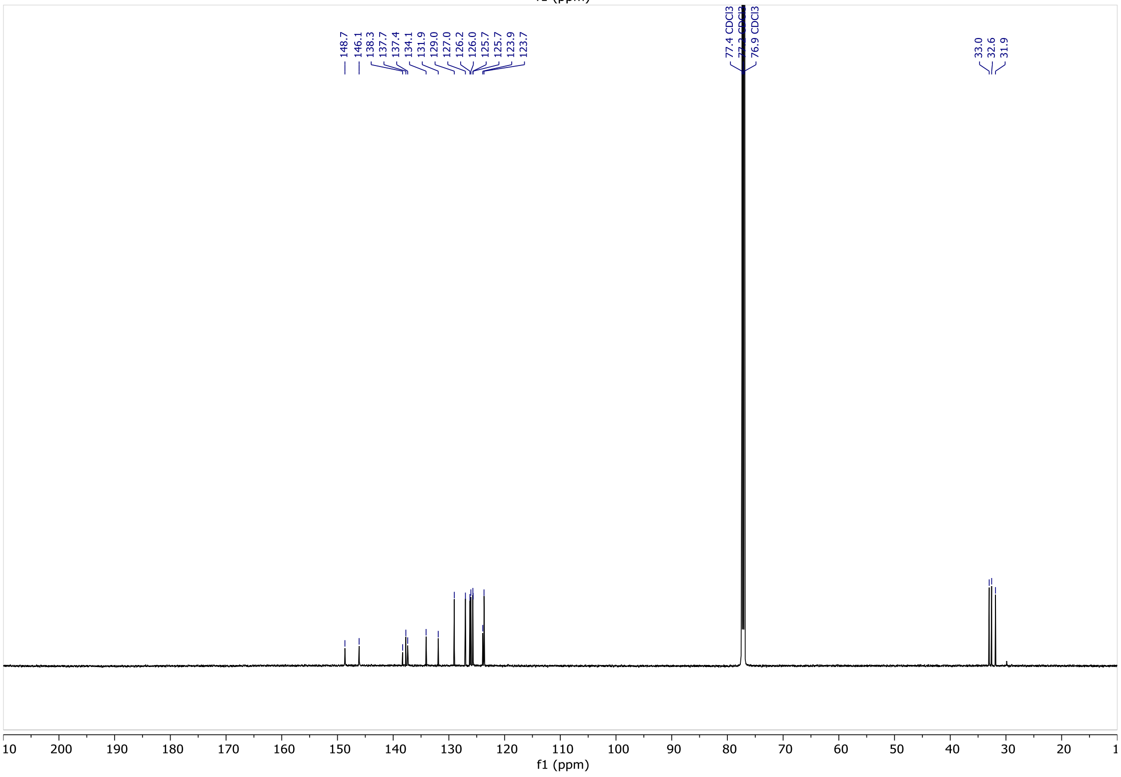

3-(3-(naphthalen-1-yl)propyl)pyridine 1-oxide (46)

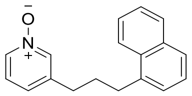

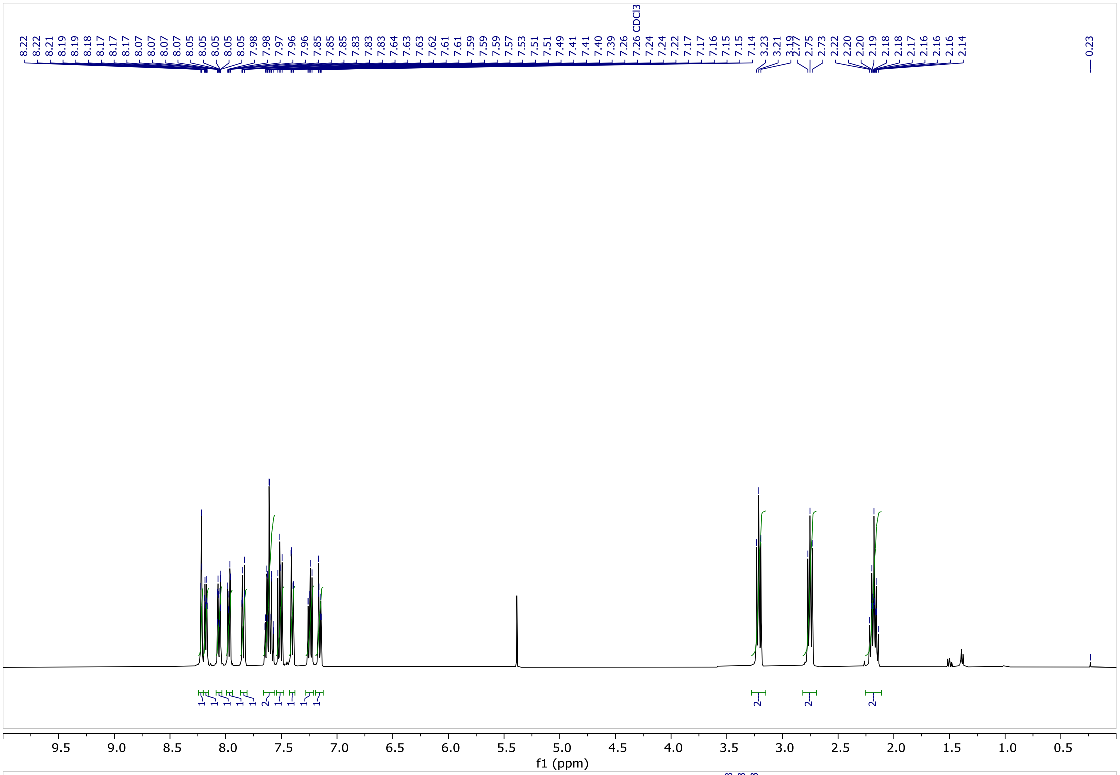

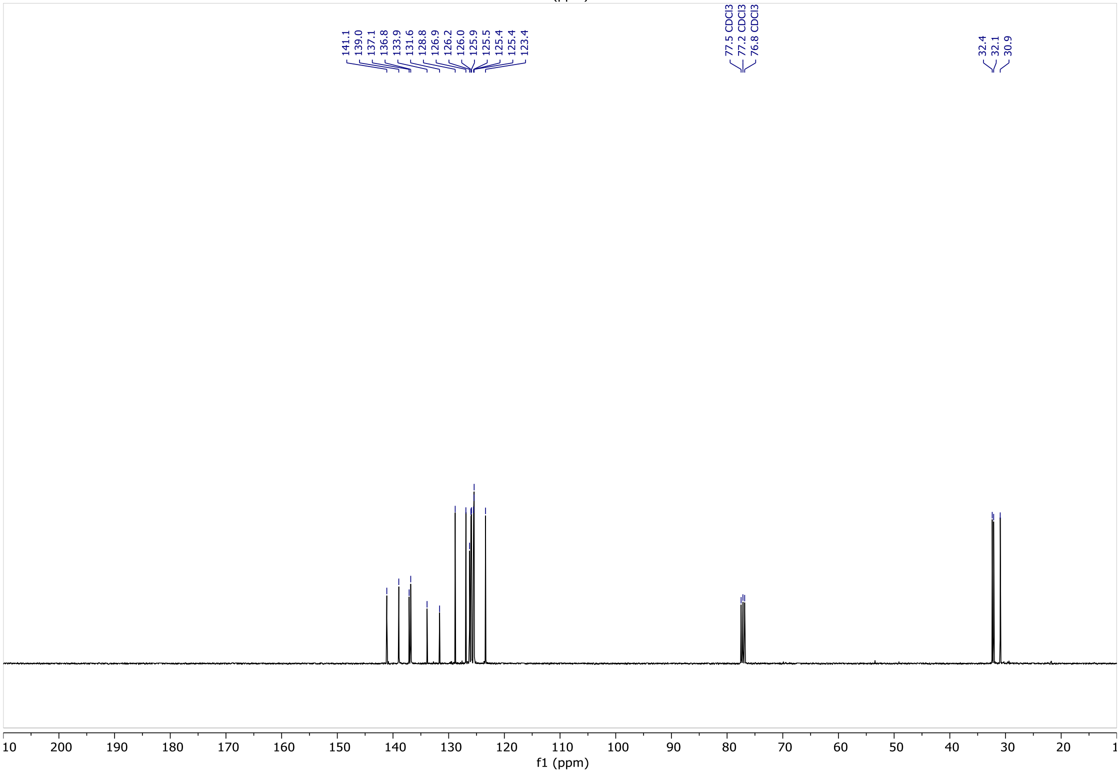

3-(3-(naphthalen-1-yl)propanoyl)pyridine 1-oxide (47)

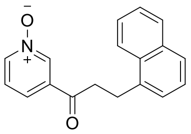

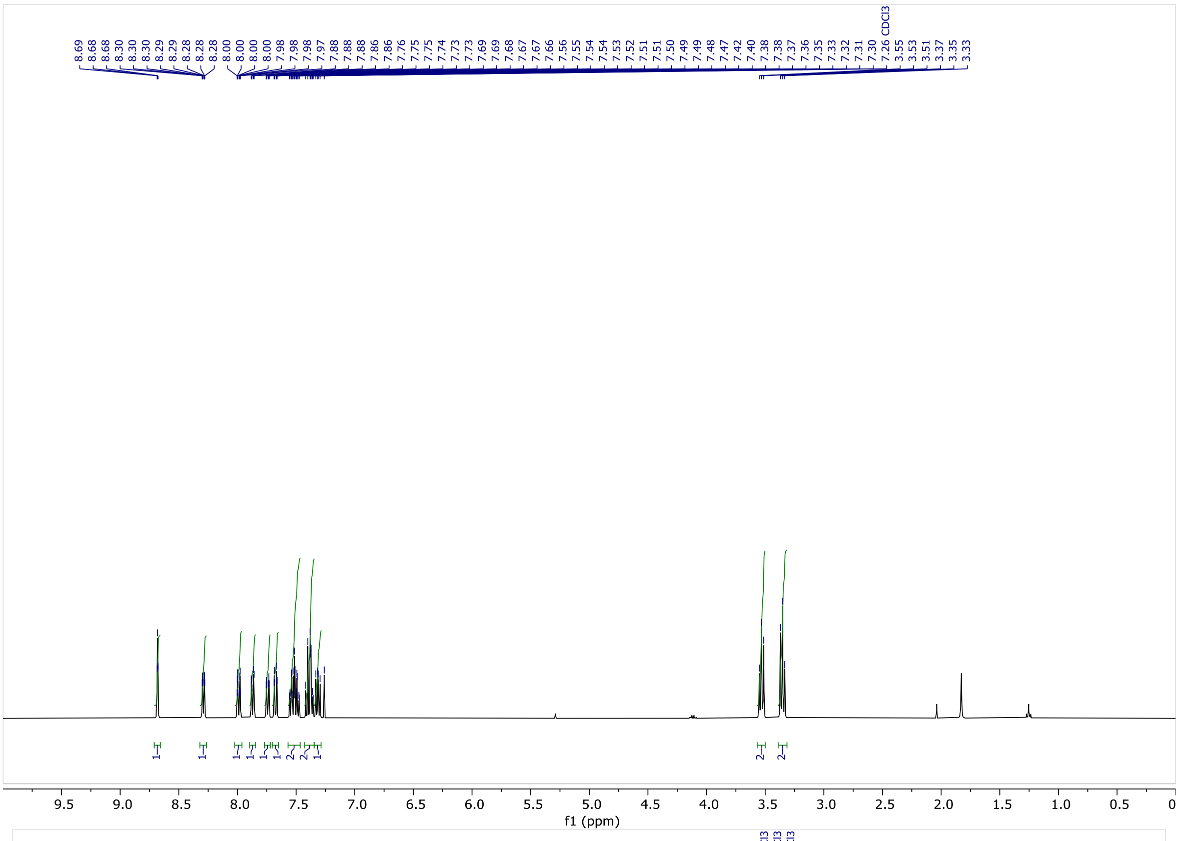

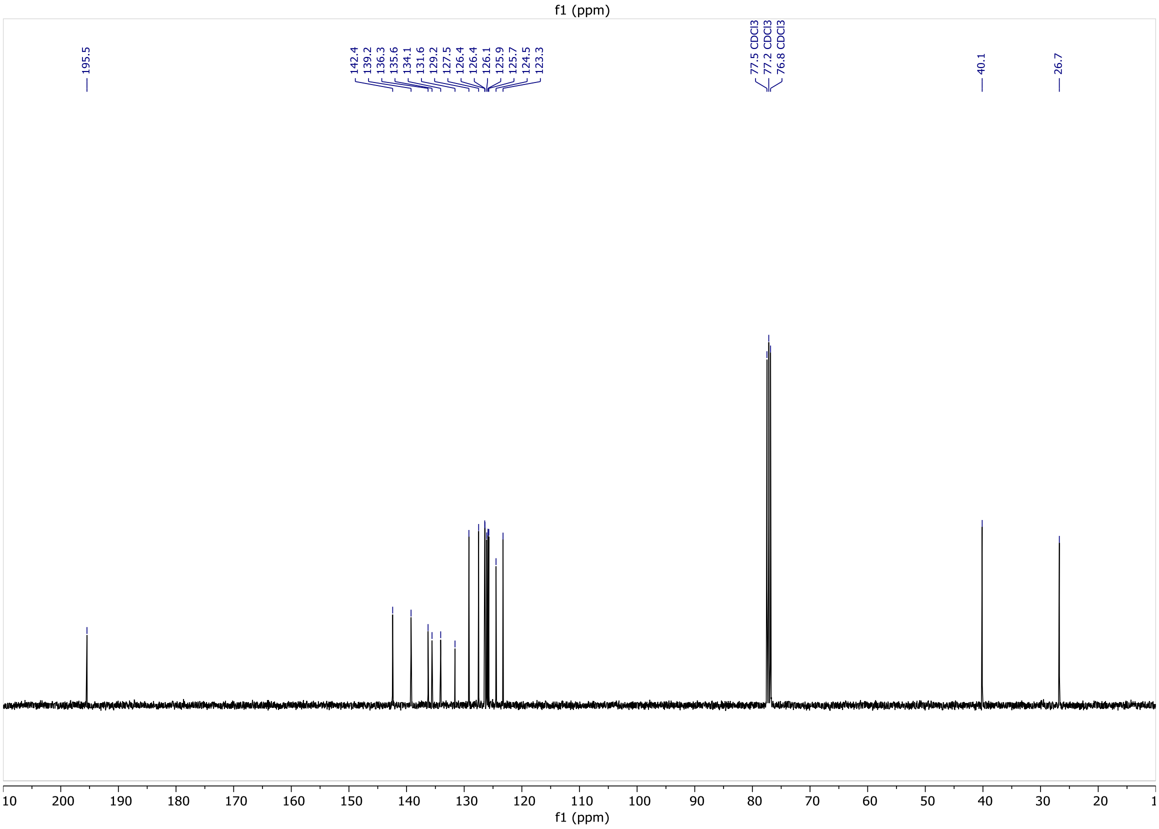

3-(4-chlorophenethoxy)pyridine (48a)

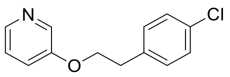

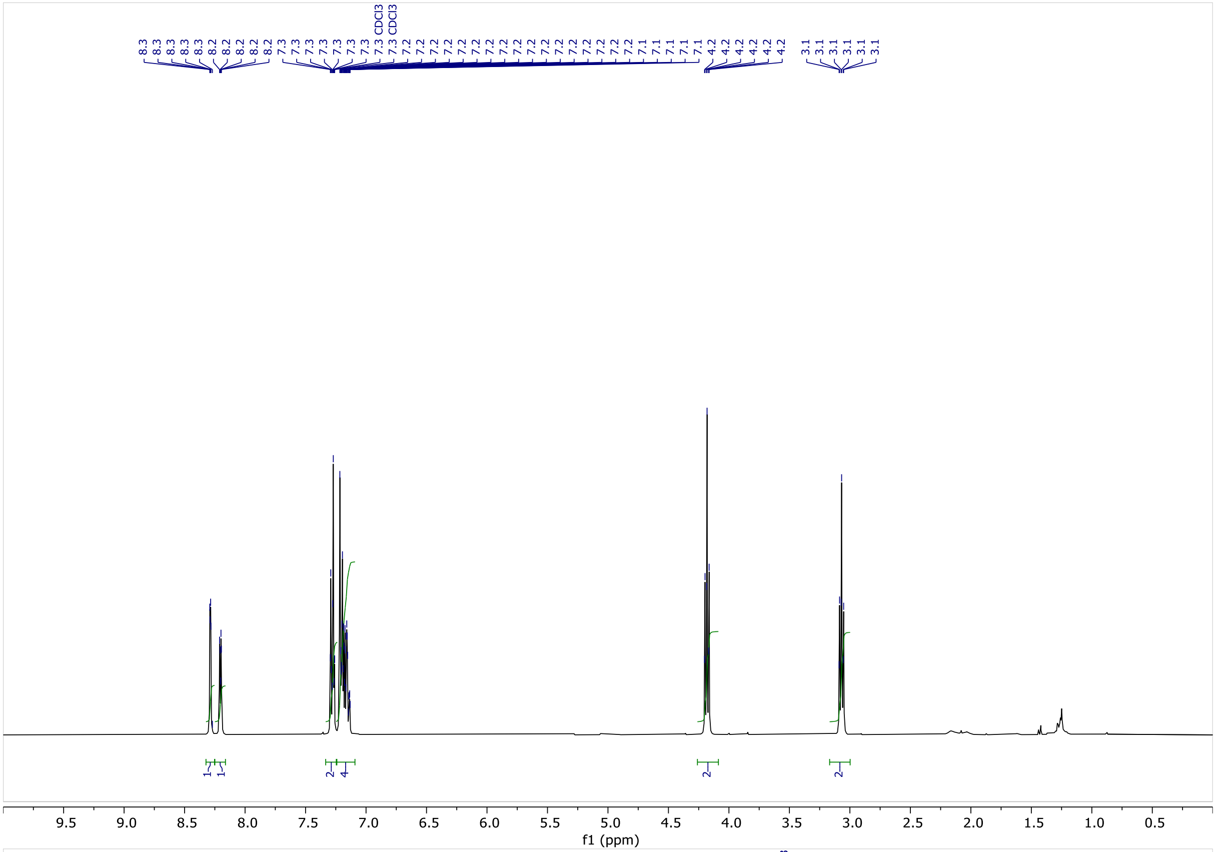

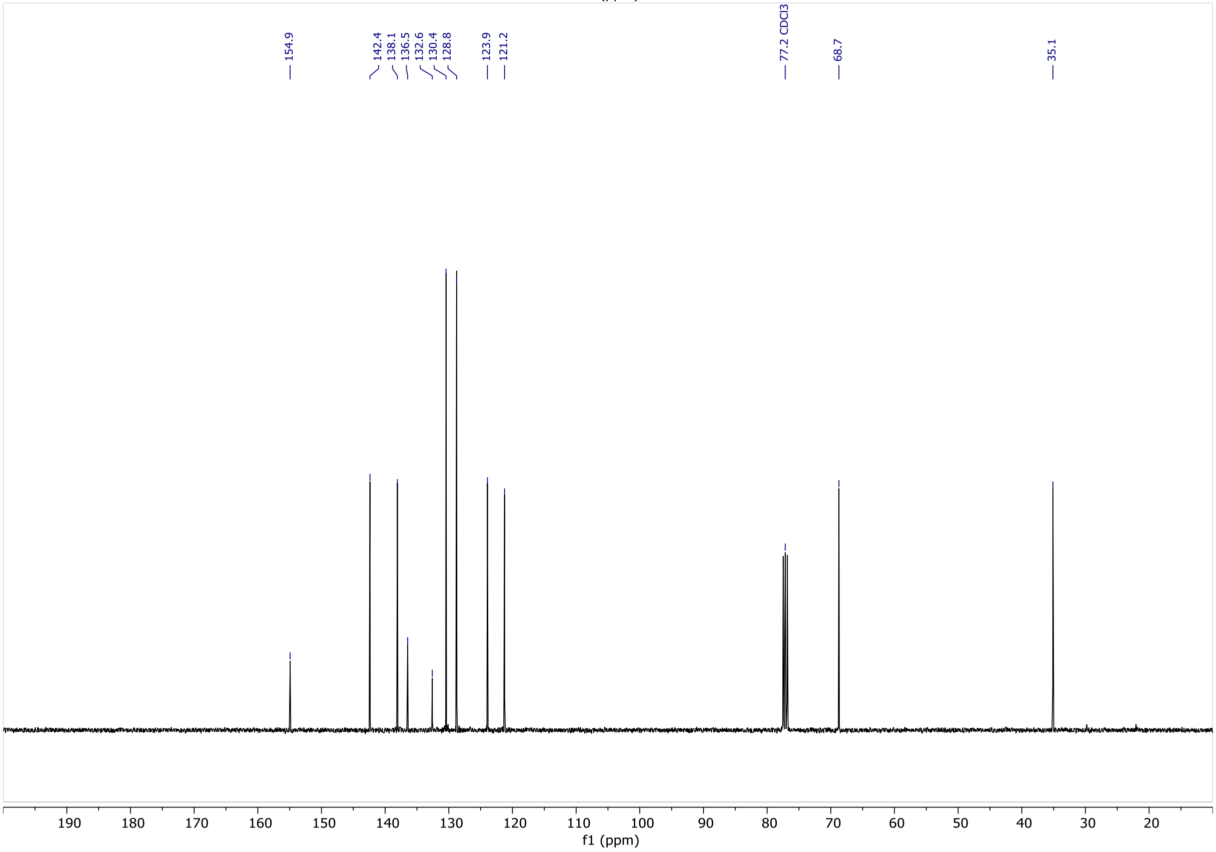

3-(4-chlorophenethoxy)pyridine 1-oxide (48)

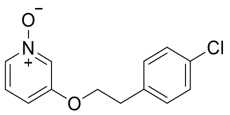

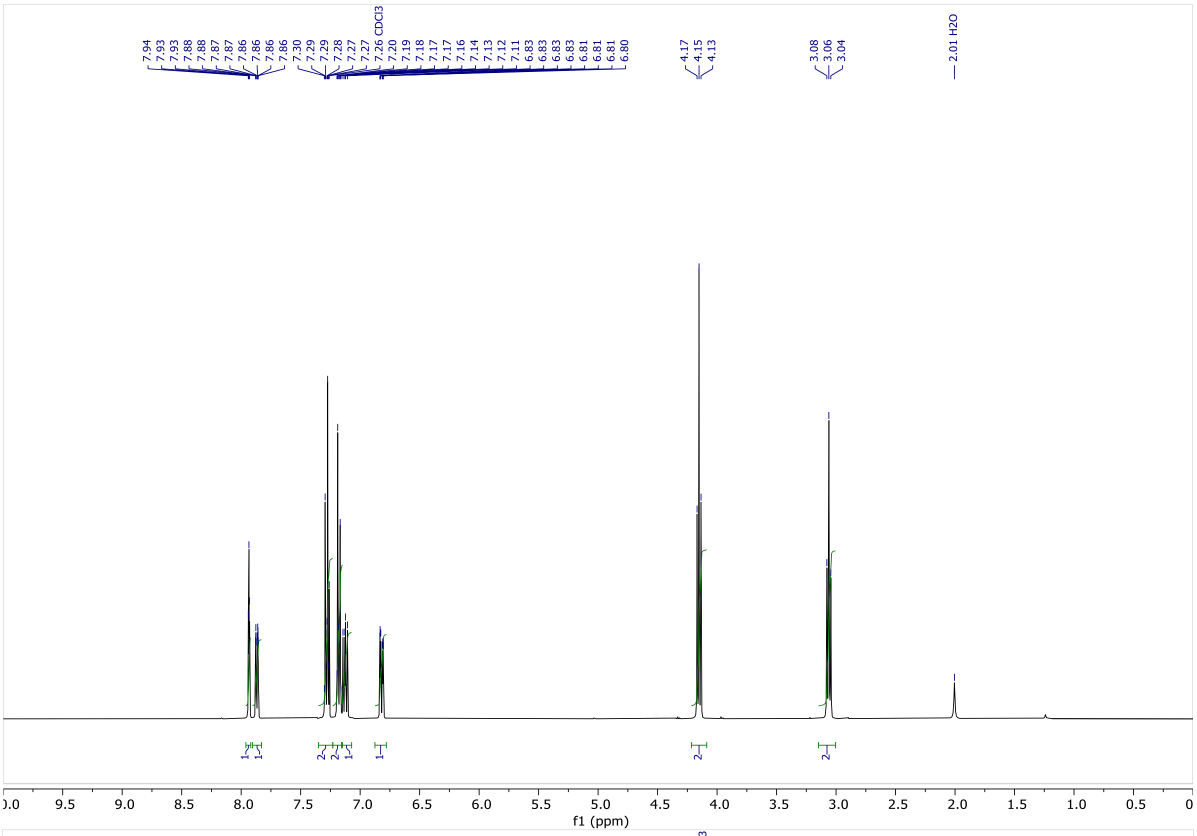

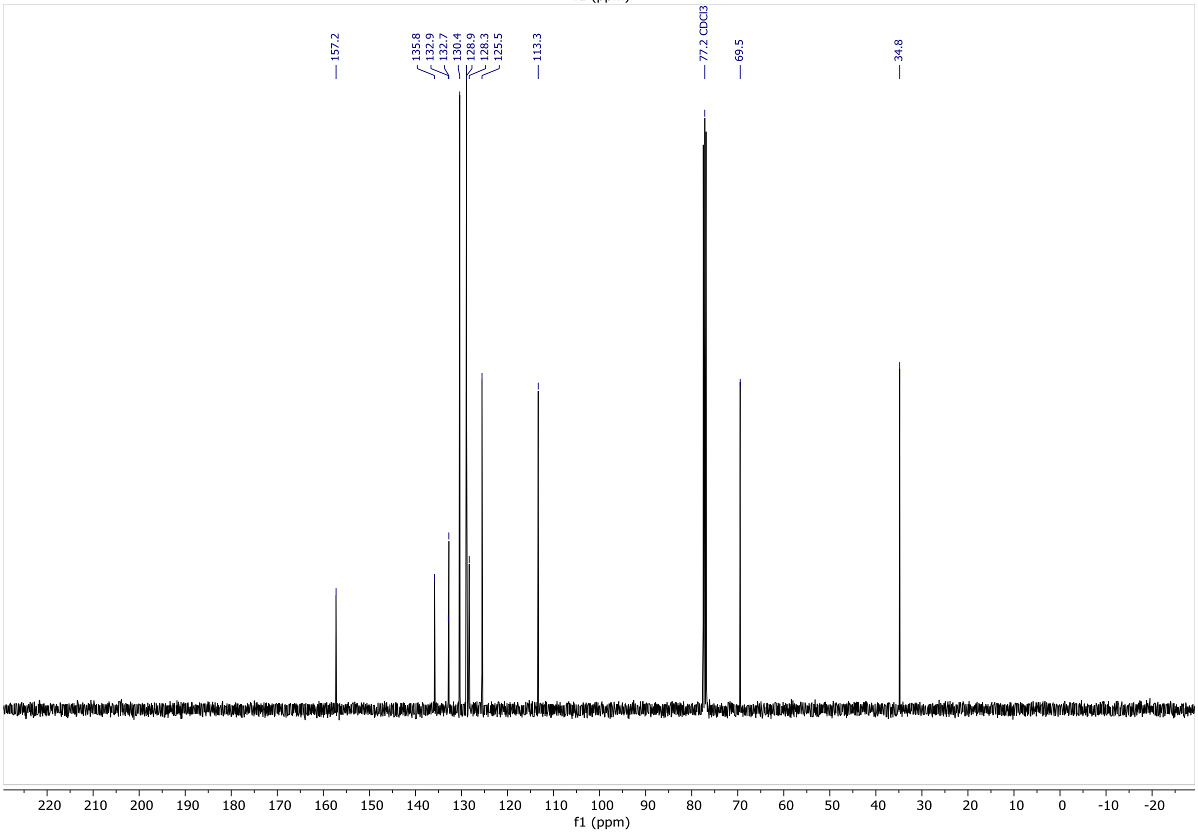

(*E*)-3-(4-chlorophenyl)-1-(pyridin-3-yl)prop-2-en-1-one (49c)

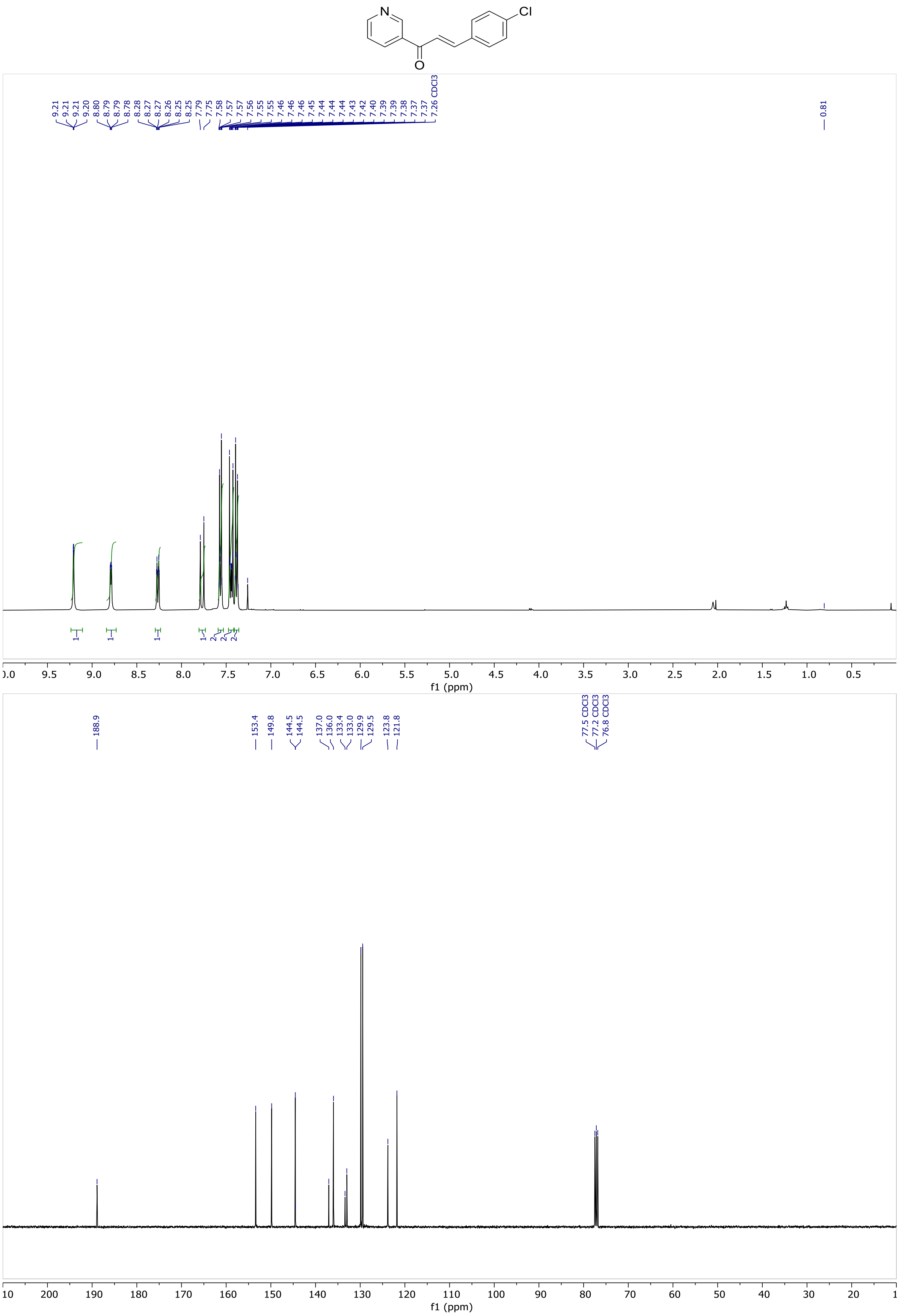

3-(4-chlorophenyl)-1-(pyridin-3-yl)propan-1-one (49b)

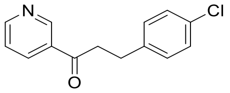

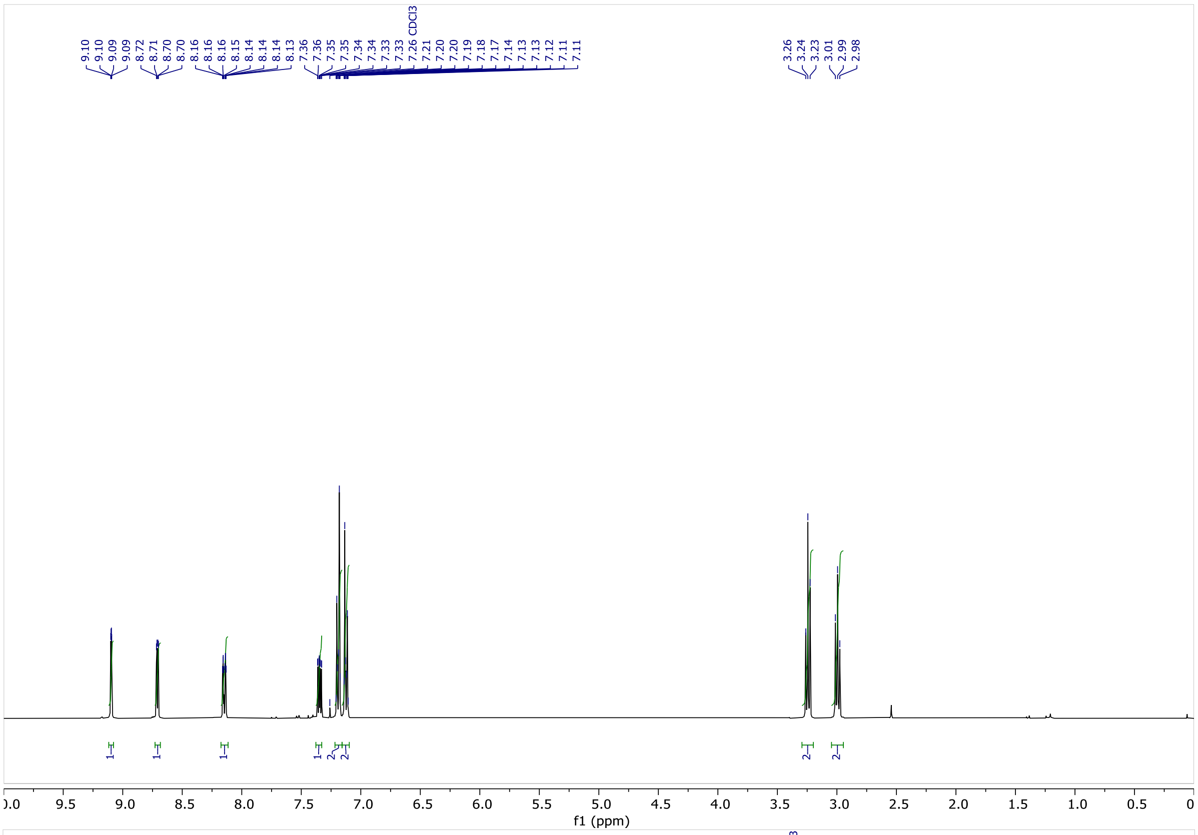

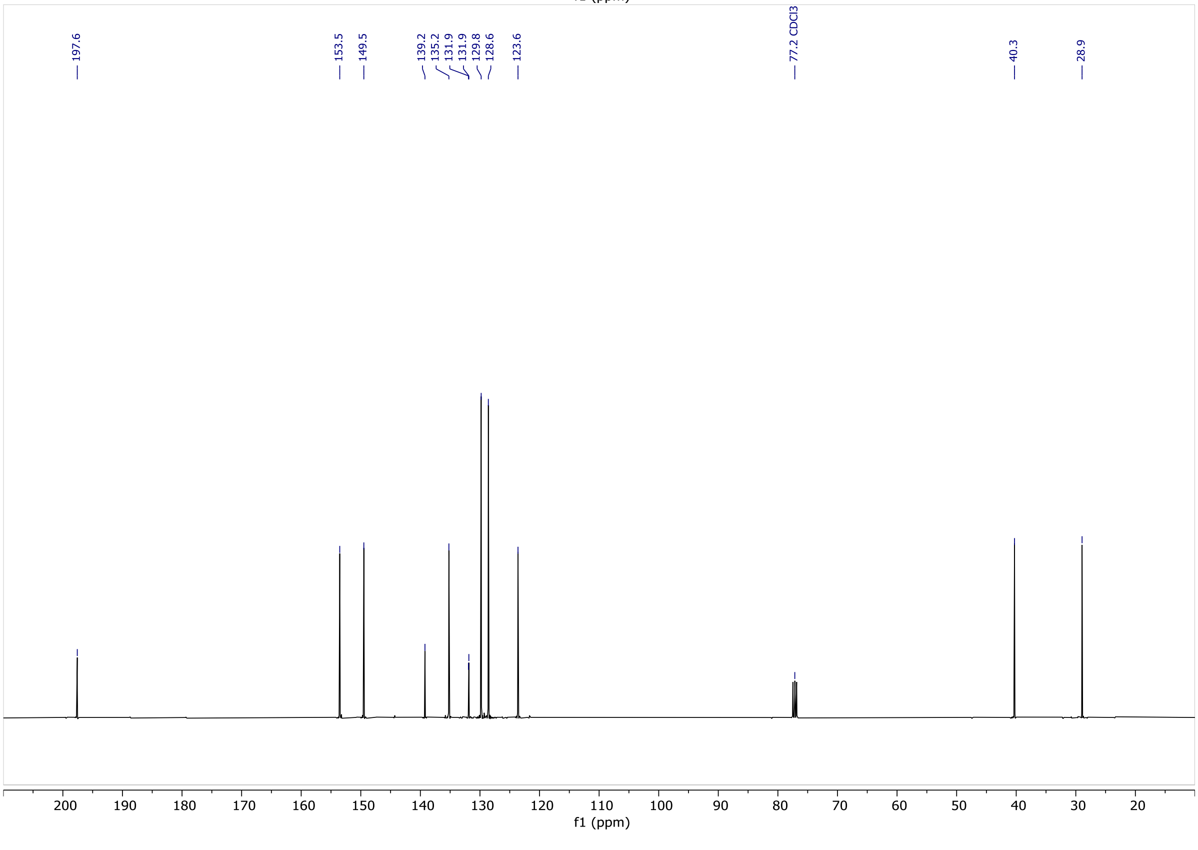

3-(3-(4-chlorophenyl)propyl)pyridine (49a)

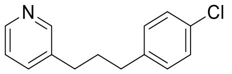

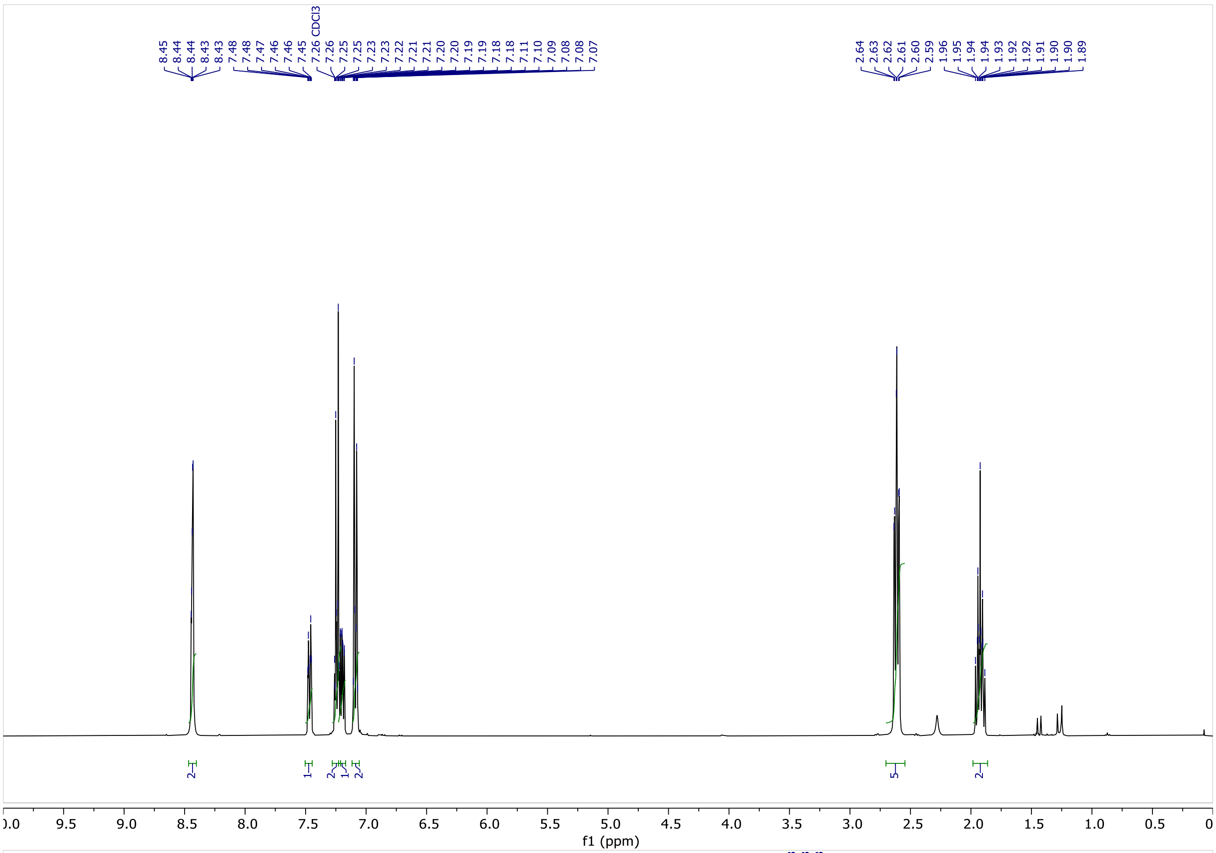

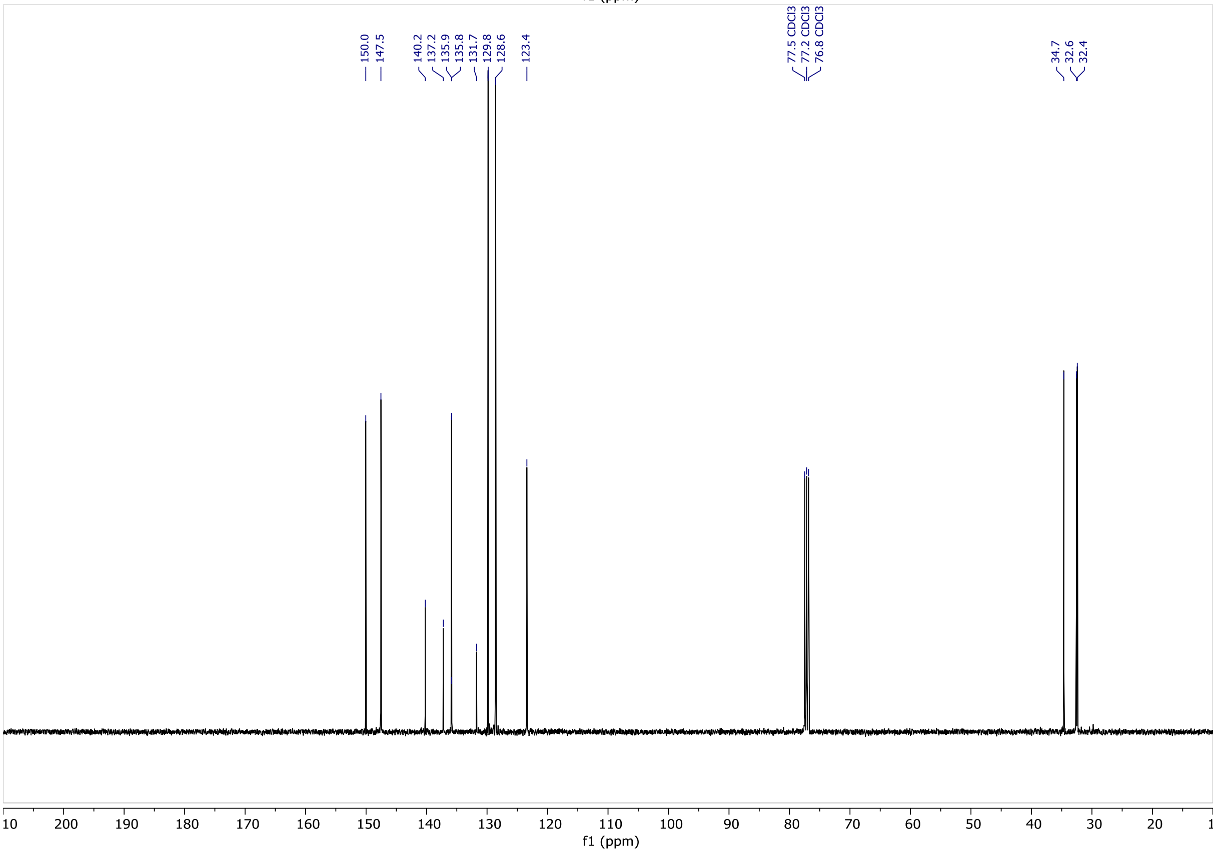

3-(3-(4-chlorophenyl)propyl)pyridine 1-oxide (49)

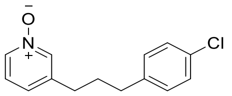

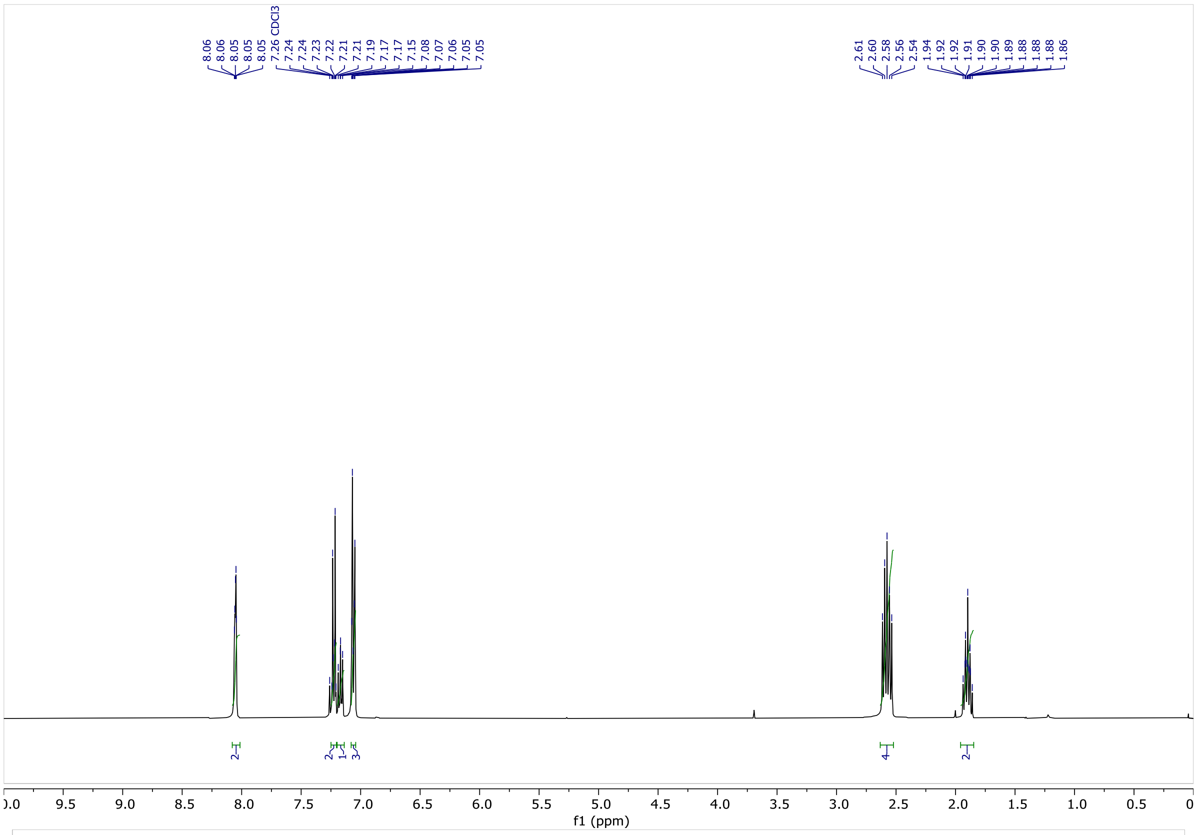

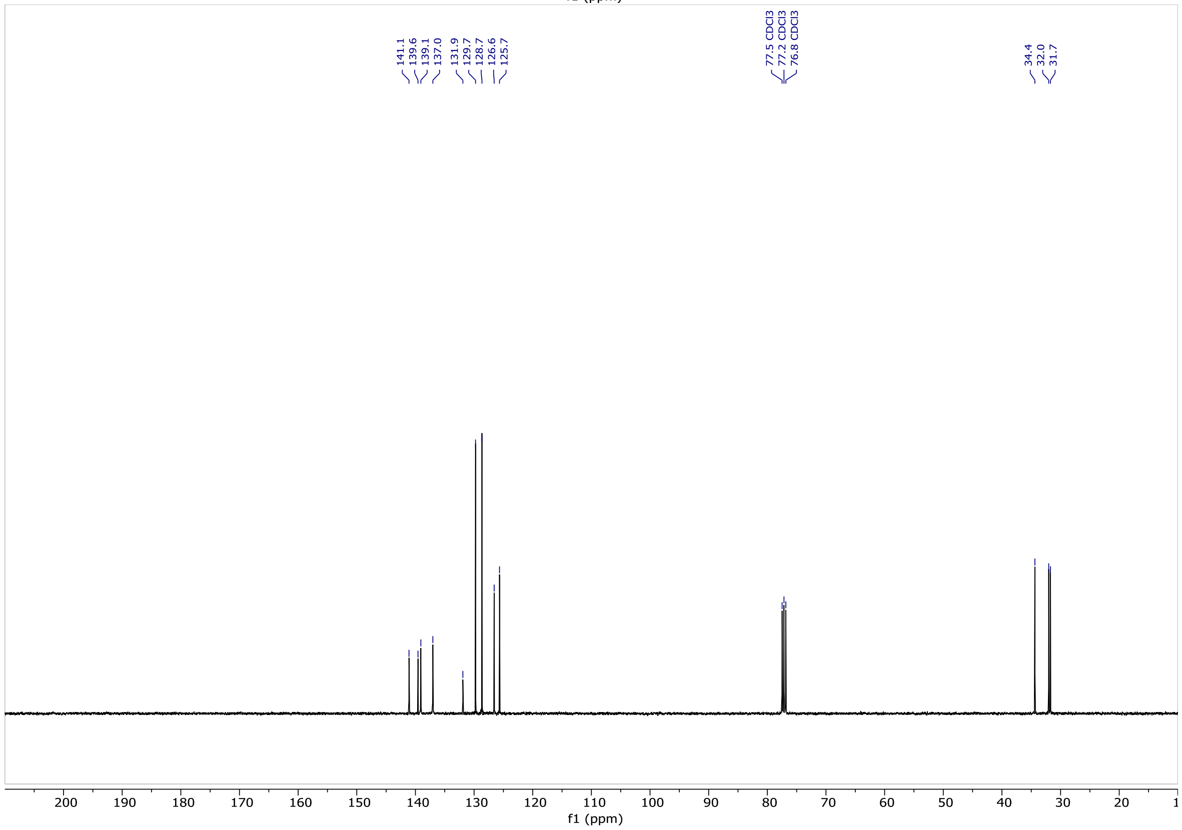

3-(3-(4-chlorophenyl)propanoyl)pyridine 1-oxide (50)

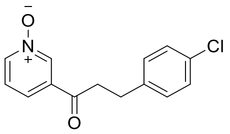

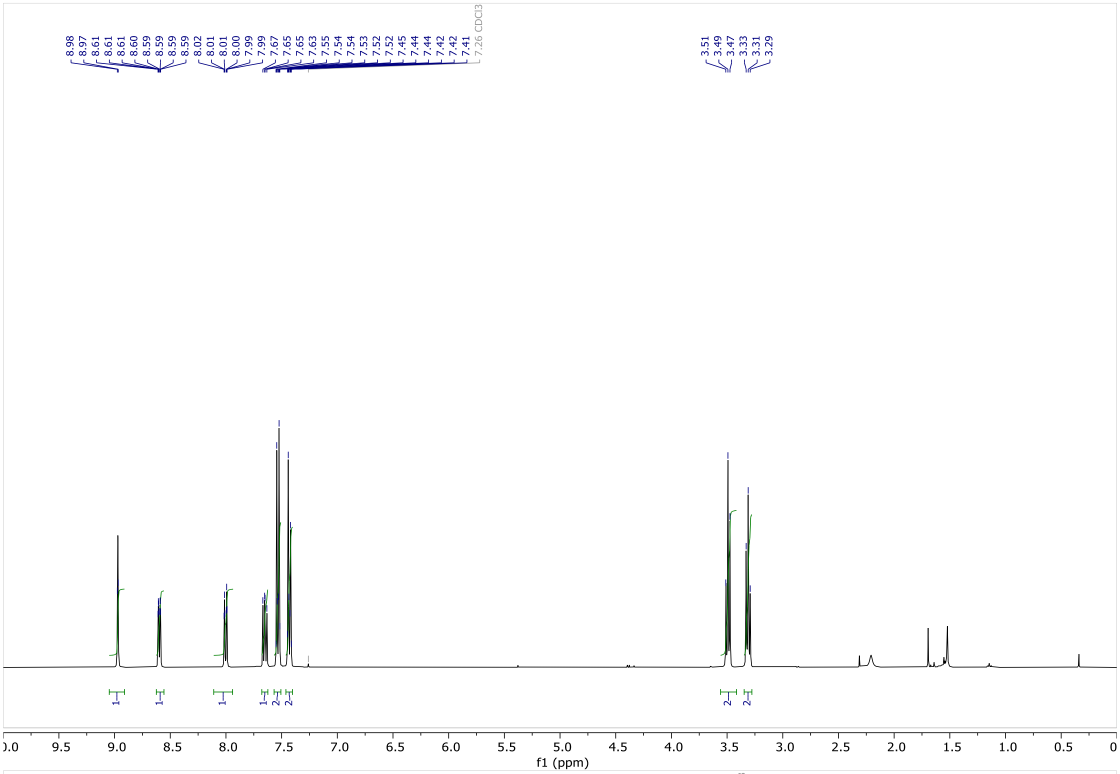

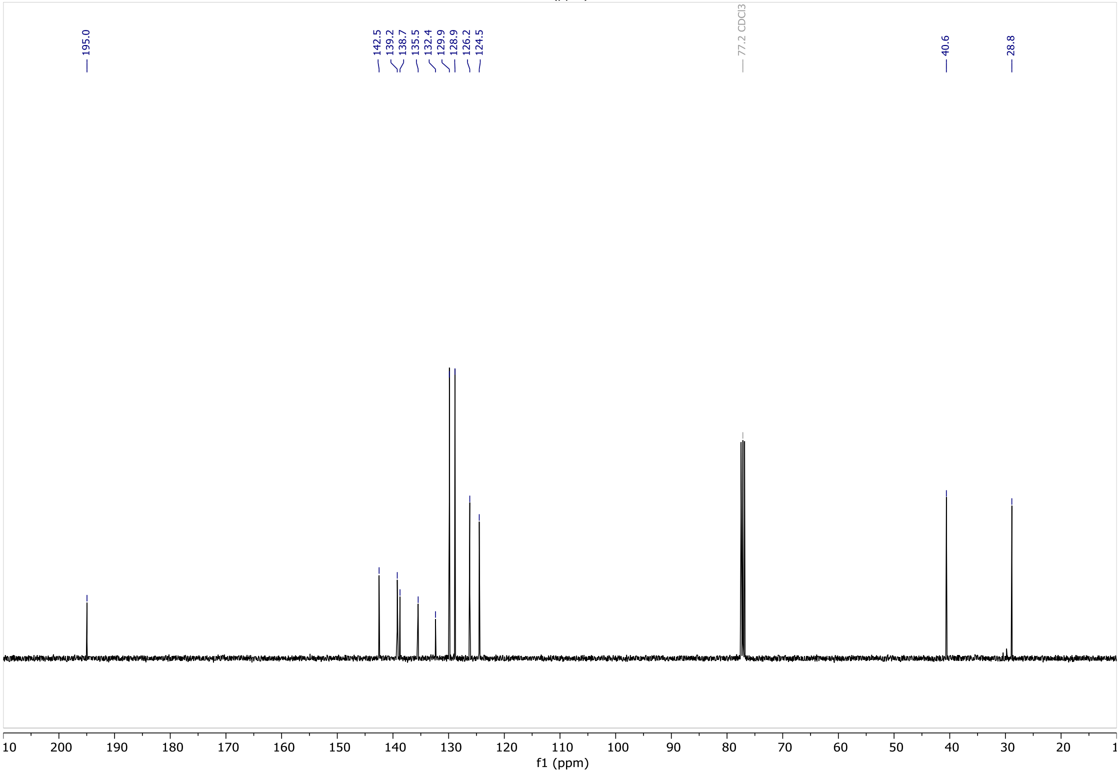

3-(2-((4-chlorophenyl)thio)ethyl)pyridine (51a)

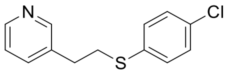

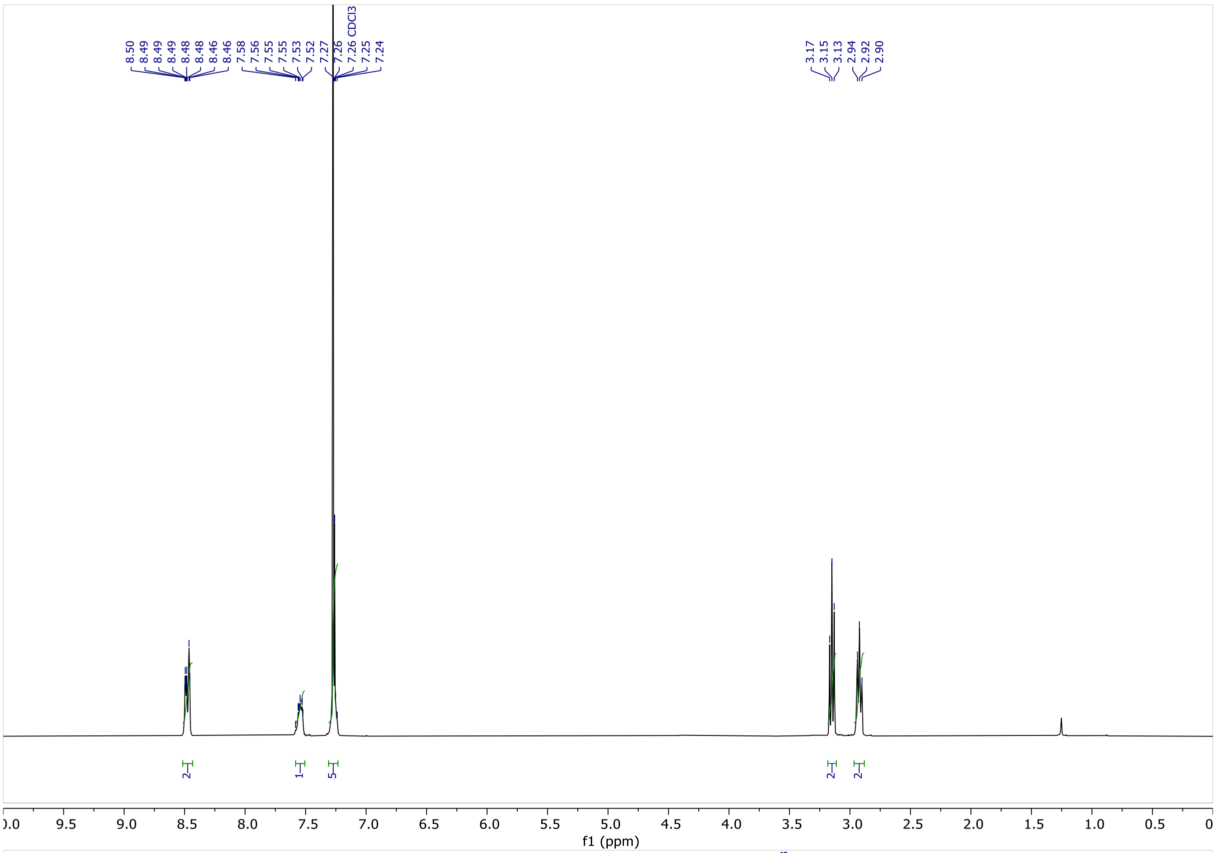

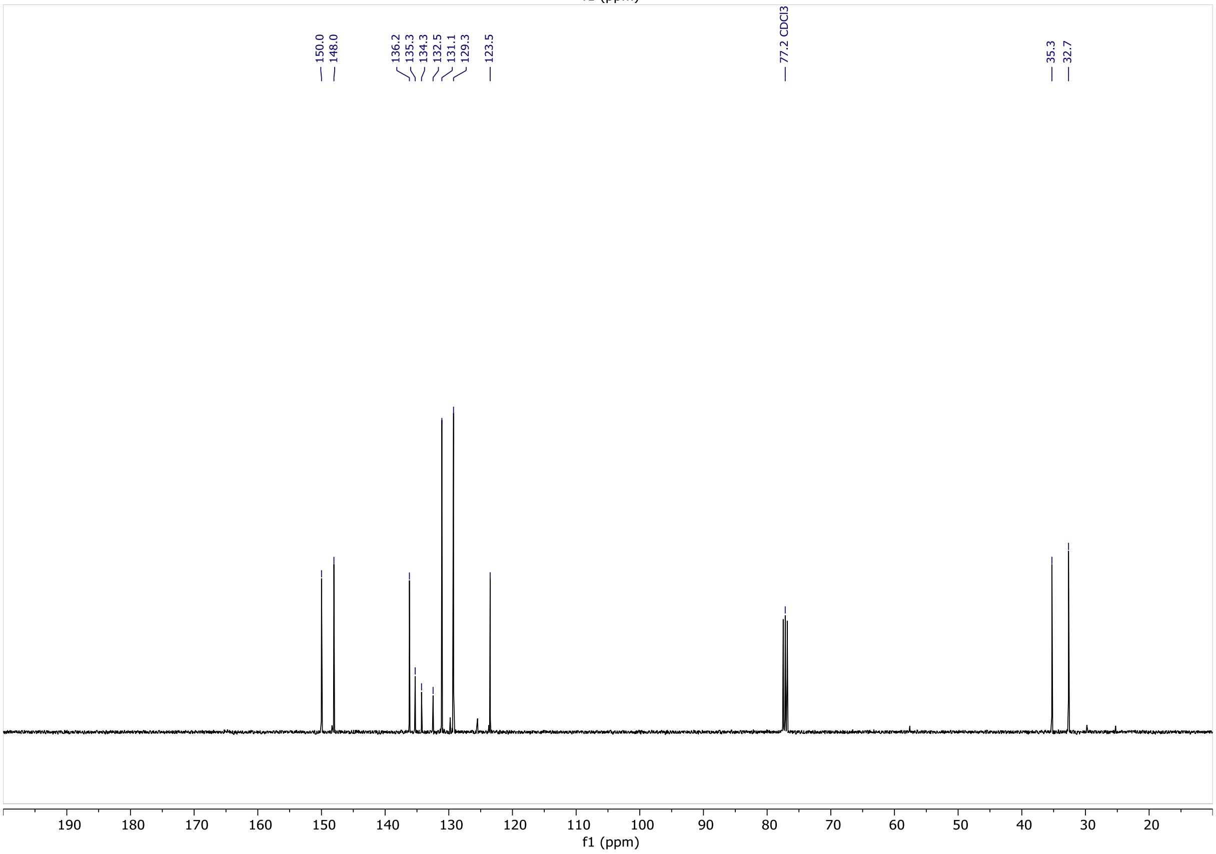

3-(2-((4-chlorophenyl)sulfonyl)ethyl)pyridine 1-oxide (51)

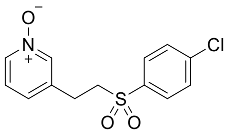

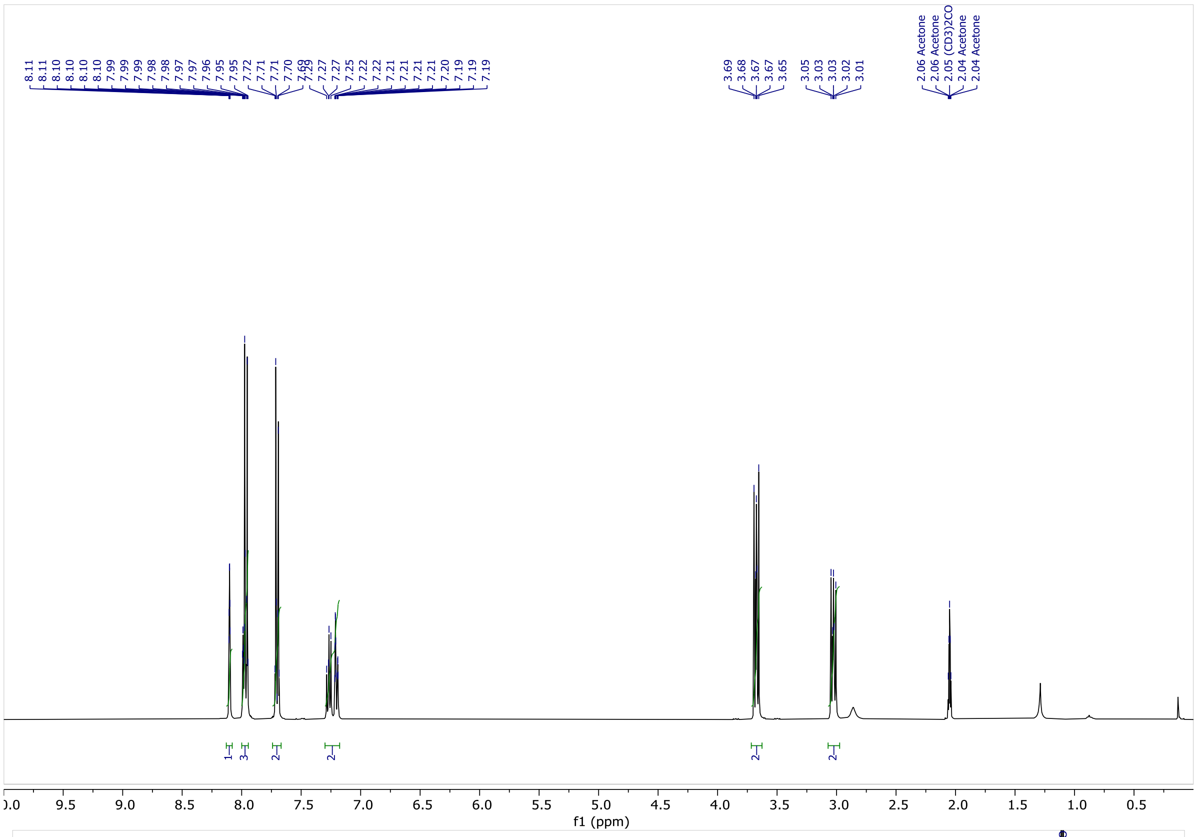

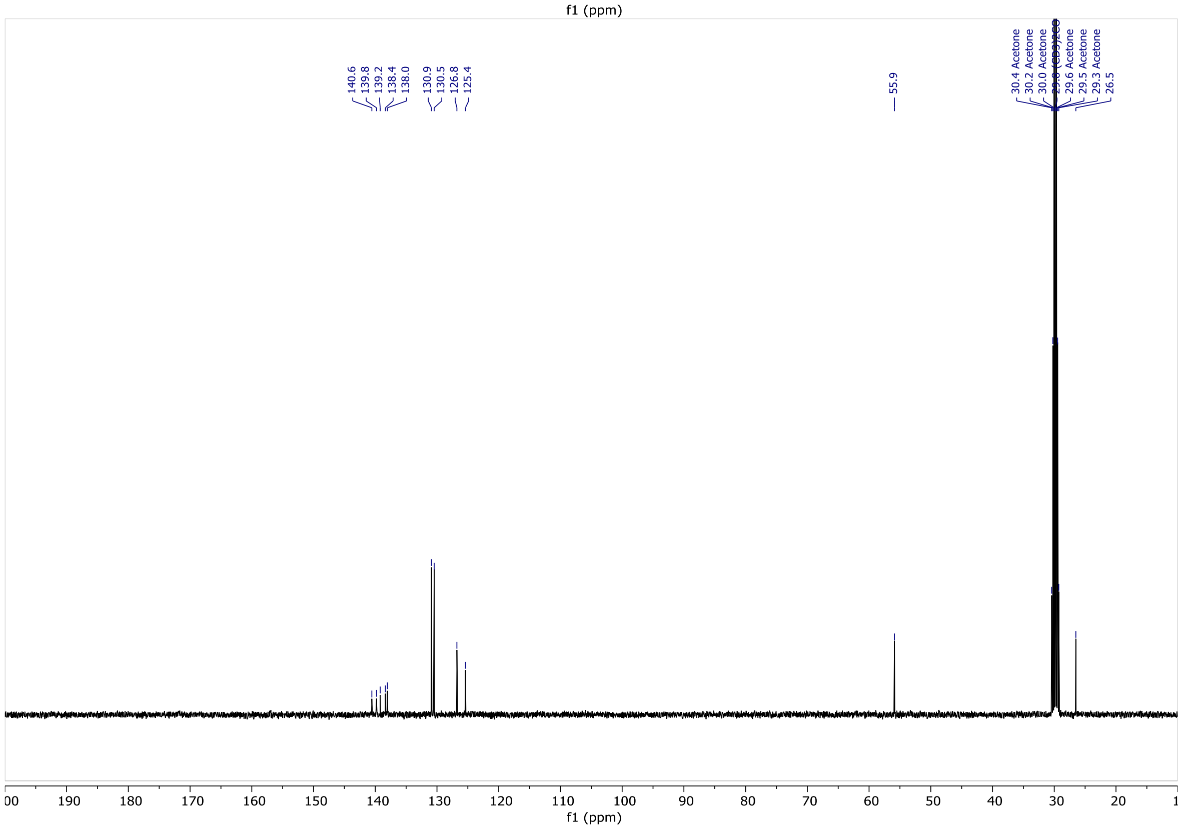

(*E*)-1-(4-chlorophenyl)-3-(pyridin-3-yl)prop-2-en-1-one (52d)

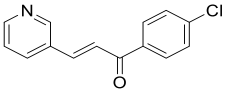

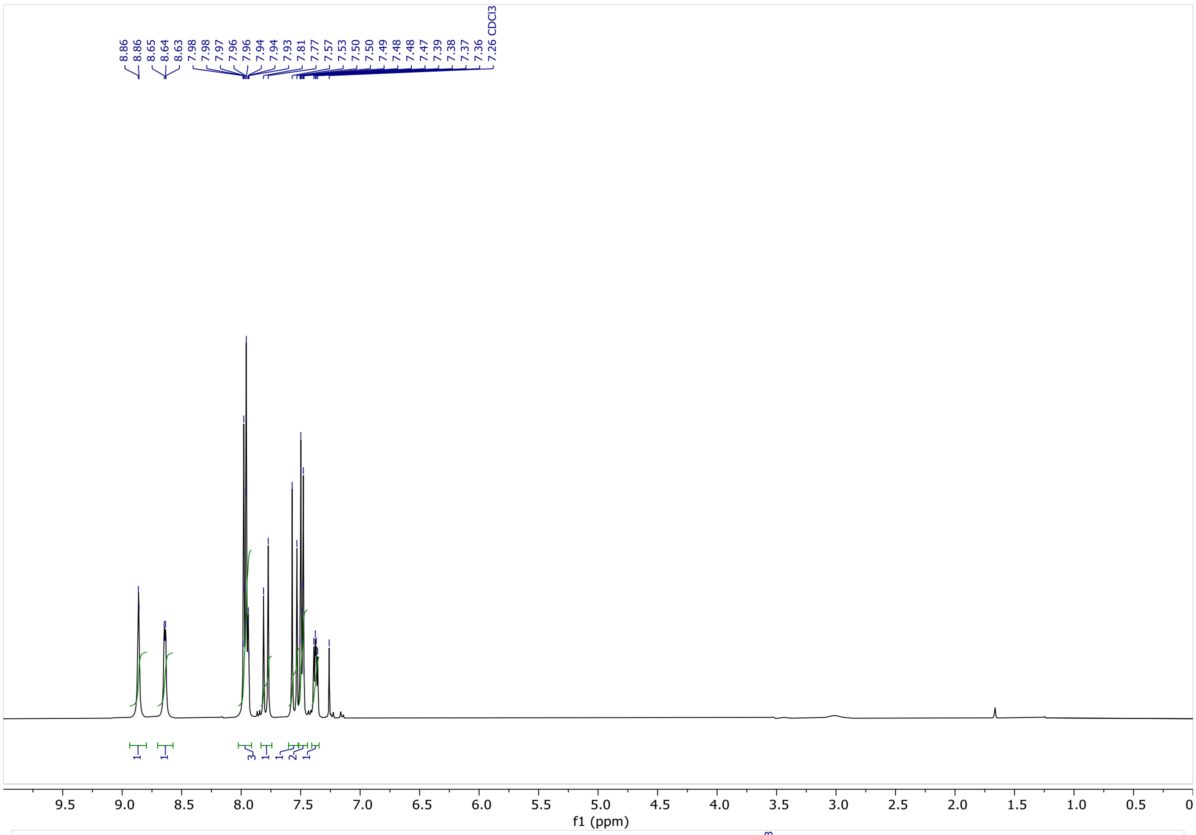

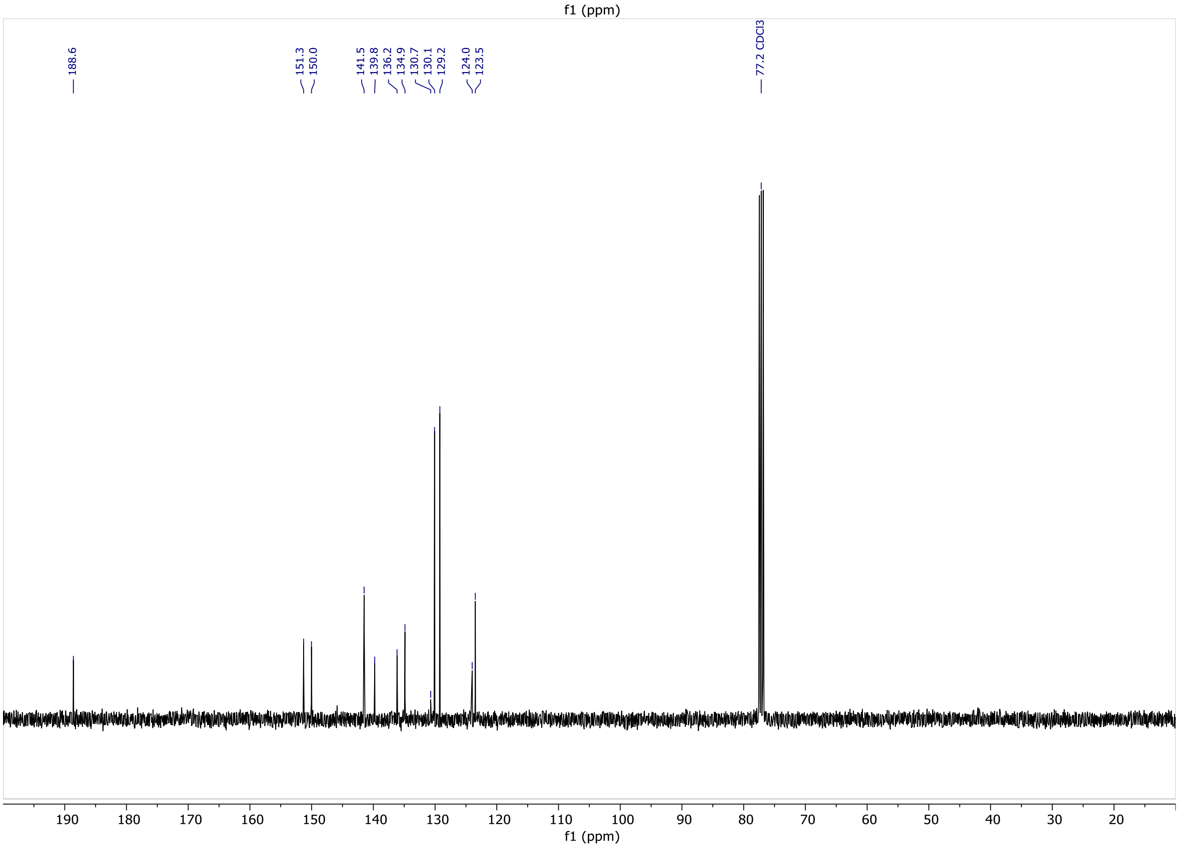

1-(4-chlorophenyl)-3-(pyridin-3-yl)propan-1-one (52c)

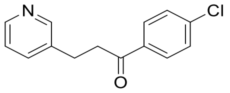

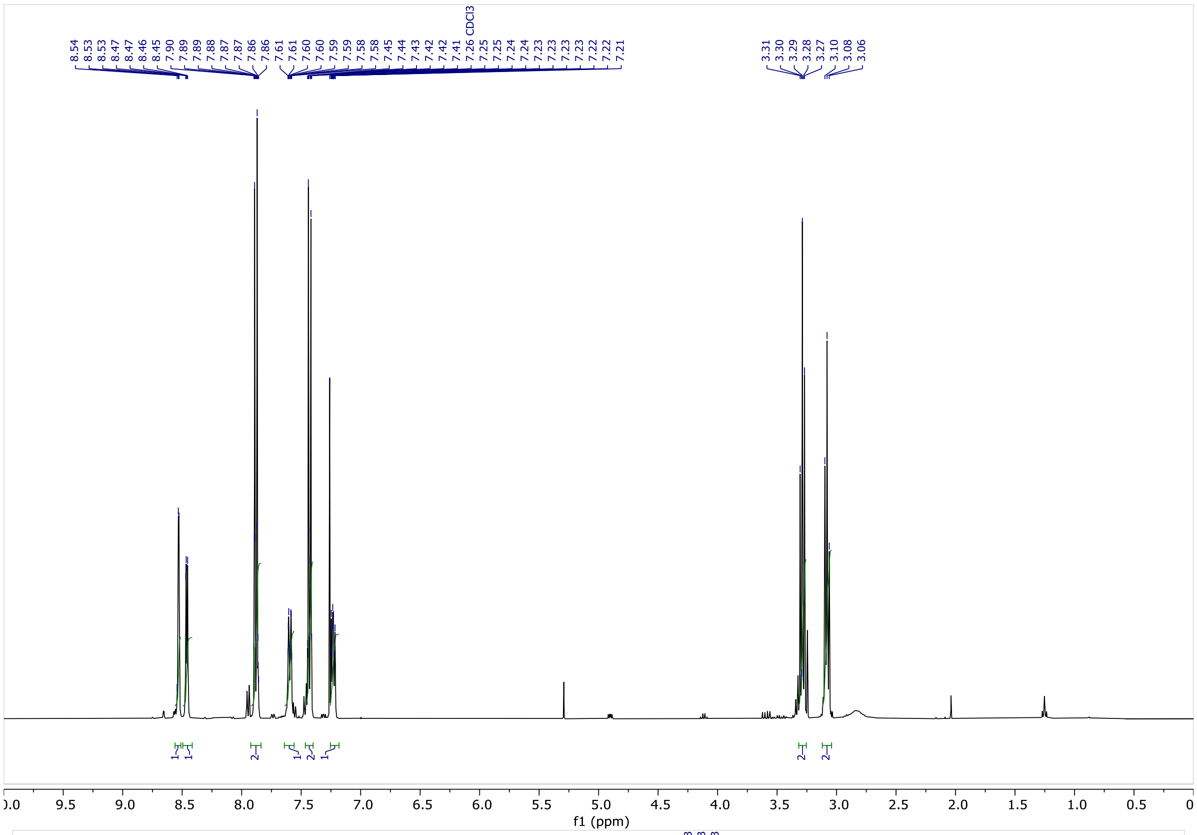

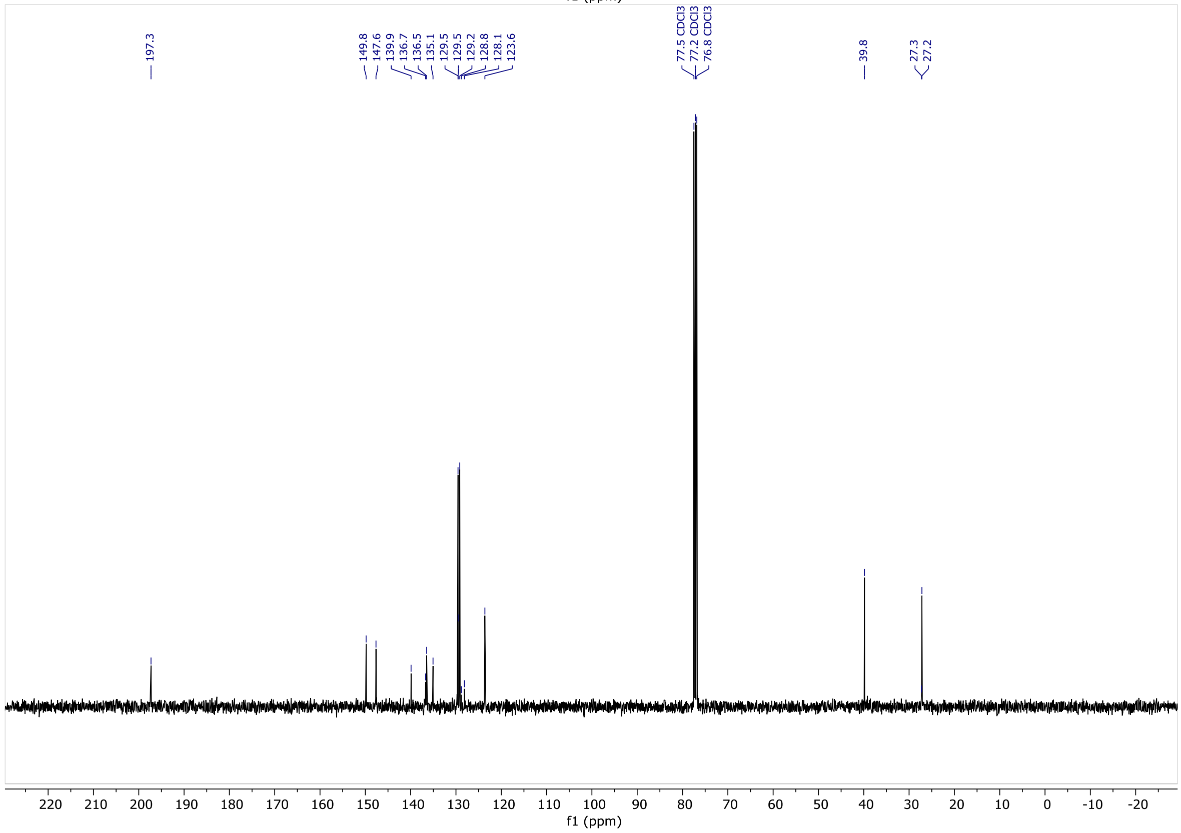

1-(4-chlorophenyl)-3-(pyridin-3-yl)propan-1-ol (52b)

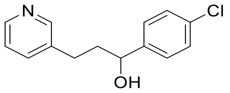

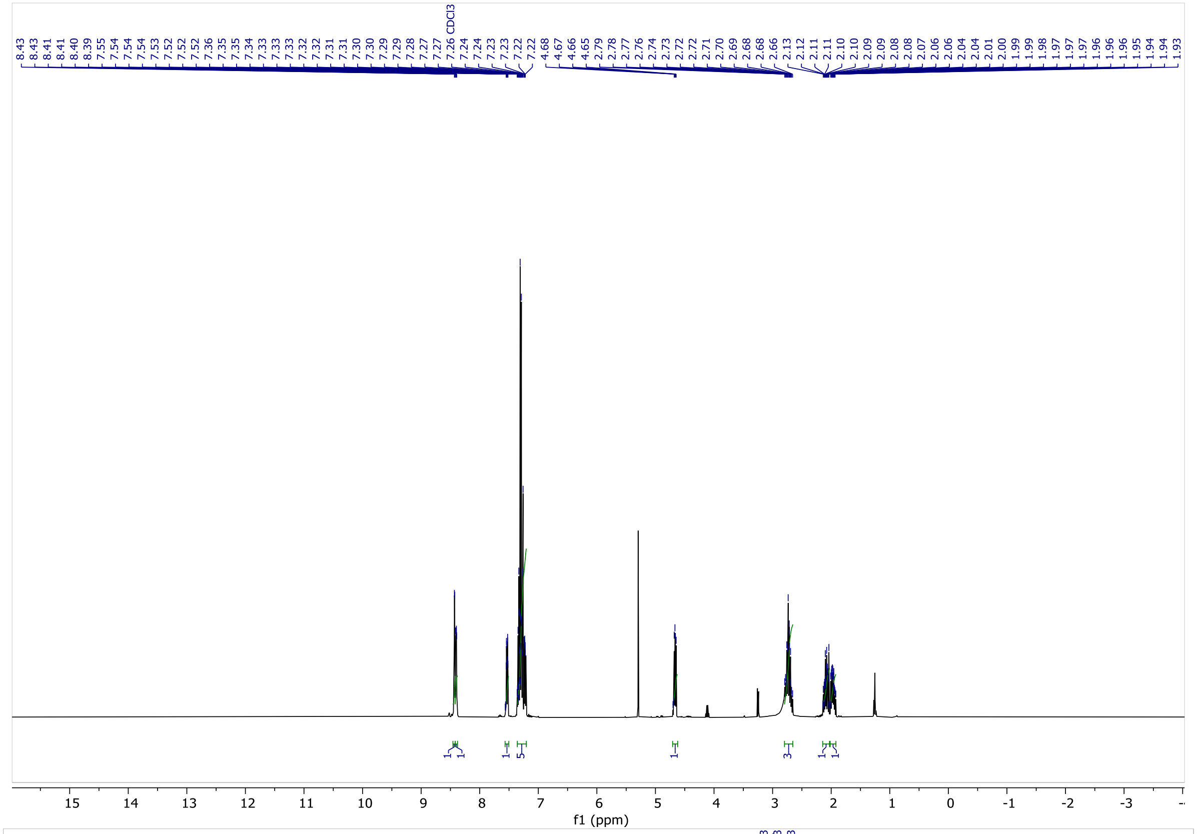

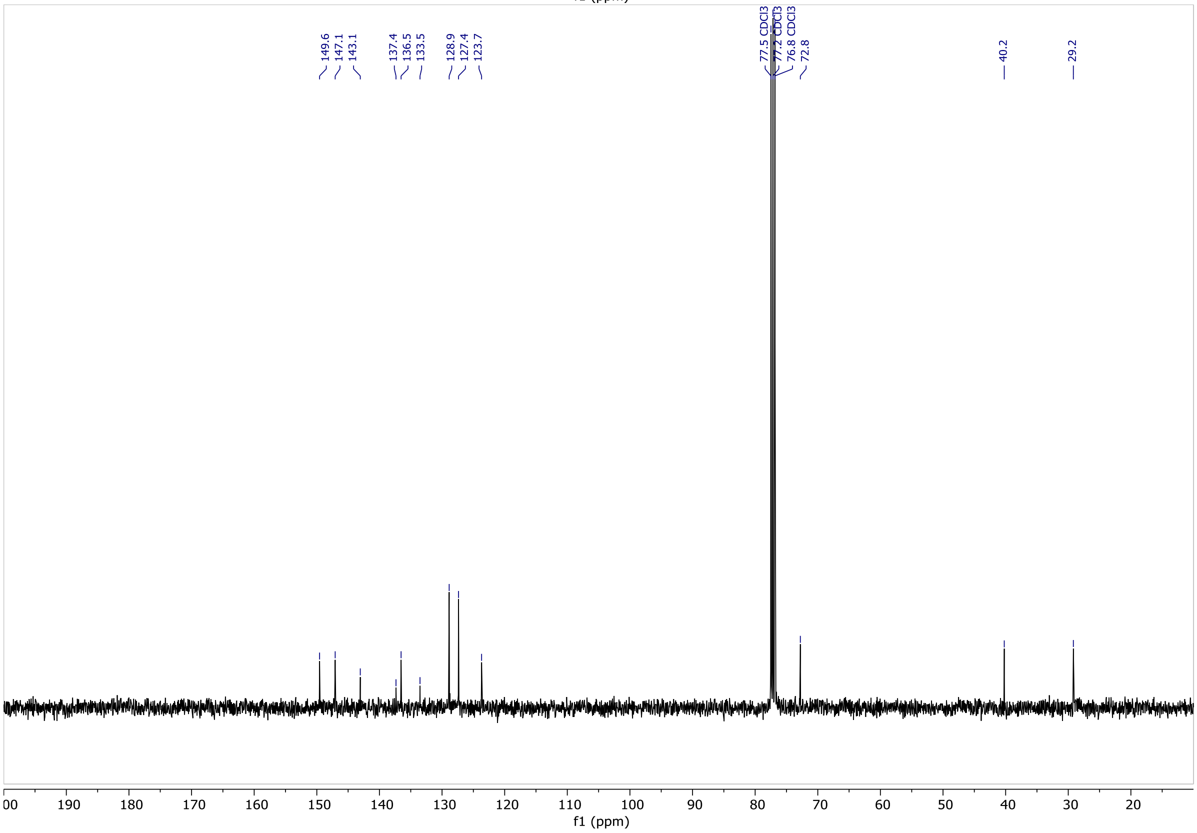

3-(3-(4-chlorophenyl)-3-fluoropropyl)pyridine (52a)

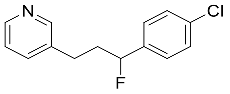

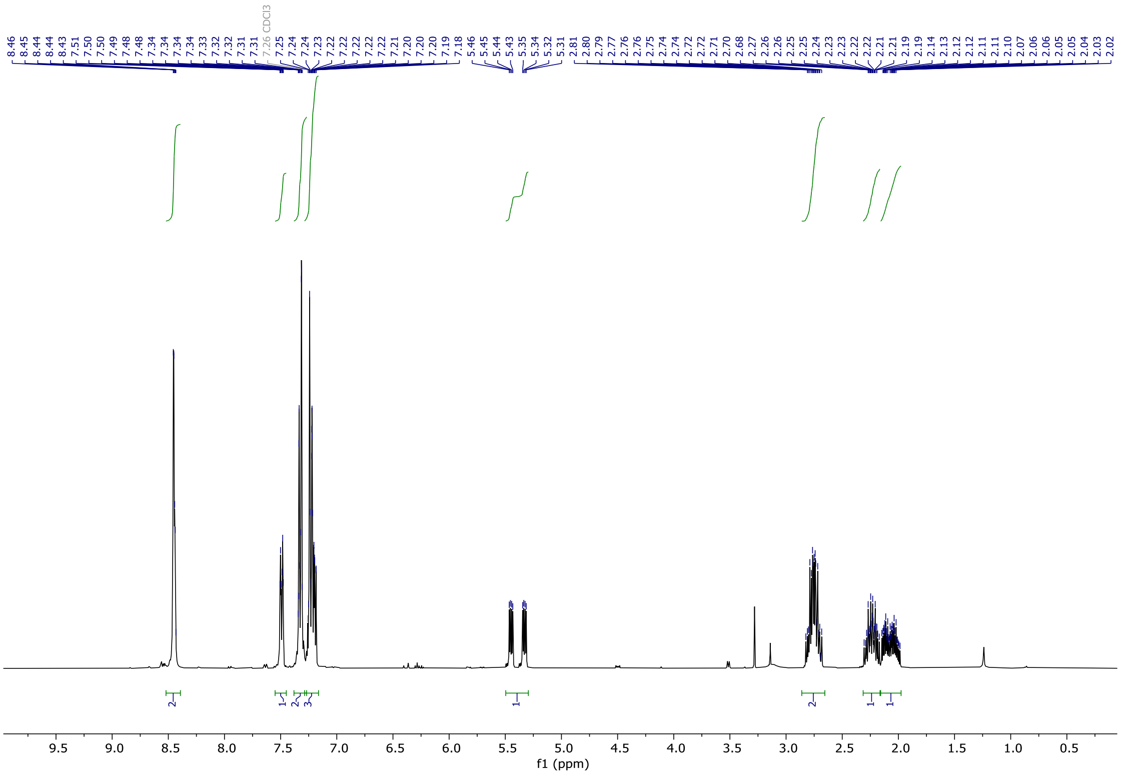

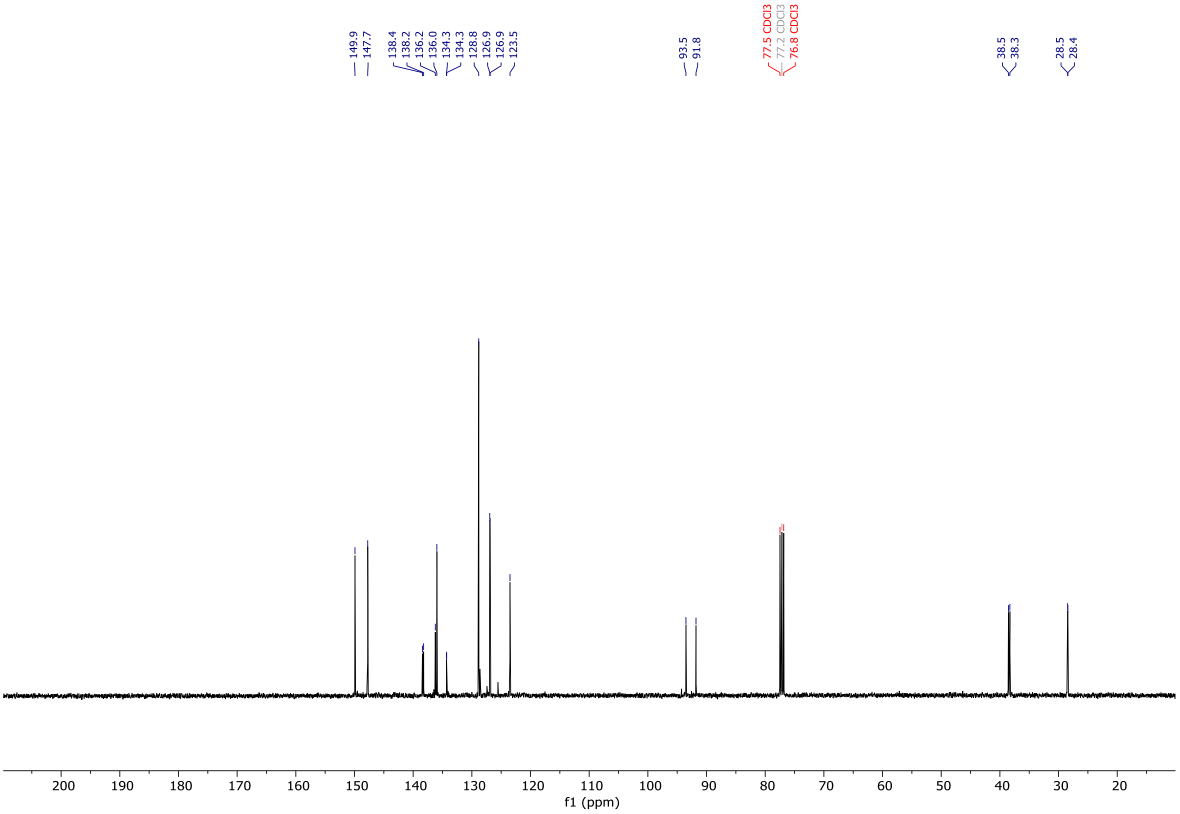

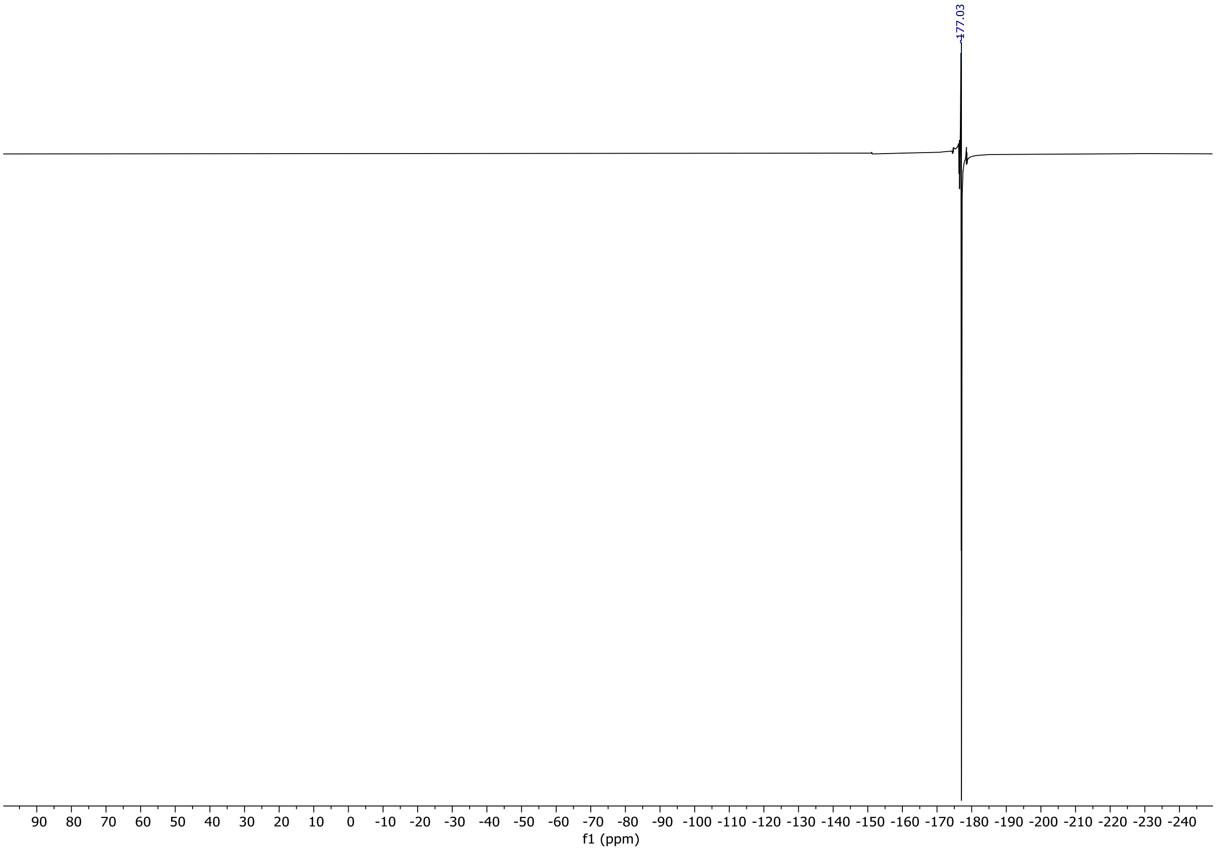
-177.03
90 80 70 60 50 40 30 20 10 0 -10 -20 -30 -40 -50 -60 -70 -80 -90 -100 -110 -120 -130 -140 -150 -160 -170 -180 -190 -200 -210 -220 -230 -240
f1 (ppm)

3-(3-(4-chlorophenyl)-3-fluoropropyl)pyridine 1-oxide (52)

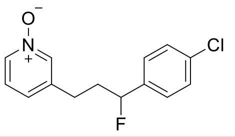
O
N
+
Cl
F

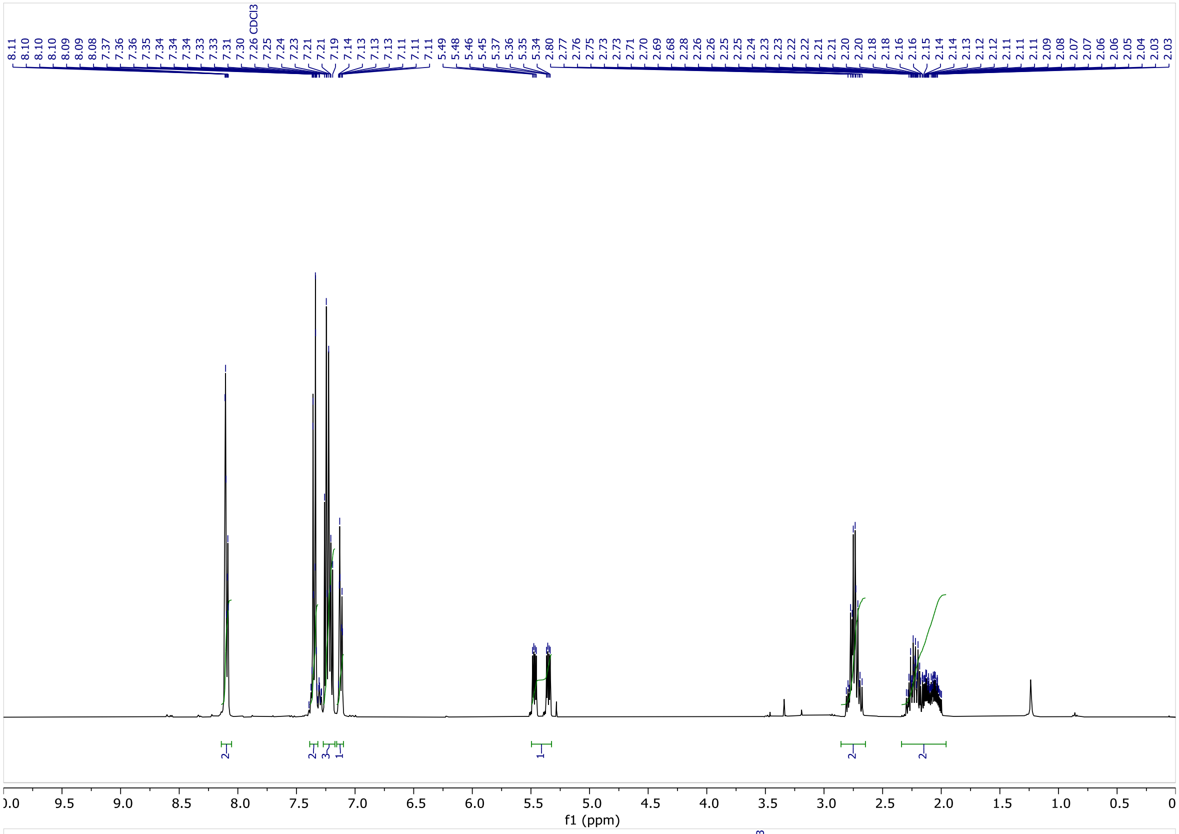
8.11 8.10 8.10 8.09 8.08 7.37 7.36 7.36 7.35 7.34 7.34 7.34 7.33 7.33 7.31 7.30 7.26 CDCl3 7.25 7.24 7.23 7.21 7.21 7.19 7.14 7.13 7.13 7.13 7.11 7.11 7.11 5.49 5.48 5.46 5.45 5.37 5.36 5.35 5.34 2.80 2.77 2.76 2.75 2.73 2.73 2.71 2.70 2.69 2.68 2.28 2.26 2.26 2.25 2.24 2.23 2.23 2.22 2.22 2.21 2.21 2.20 2.20 2.18 2.16 2.16 2.15 2.14 2.14 2.13 2.12 2.12 2.11 2.11 2.11 2.09 2.08 2.07 2.07 2.06 2.06 2.05 2.04 2.03 2.03
2 2 3 1 1 2 2
f1 (ppm)

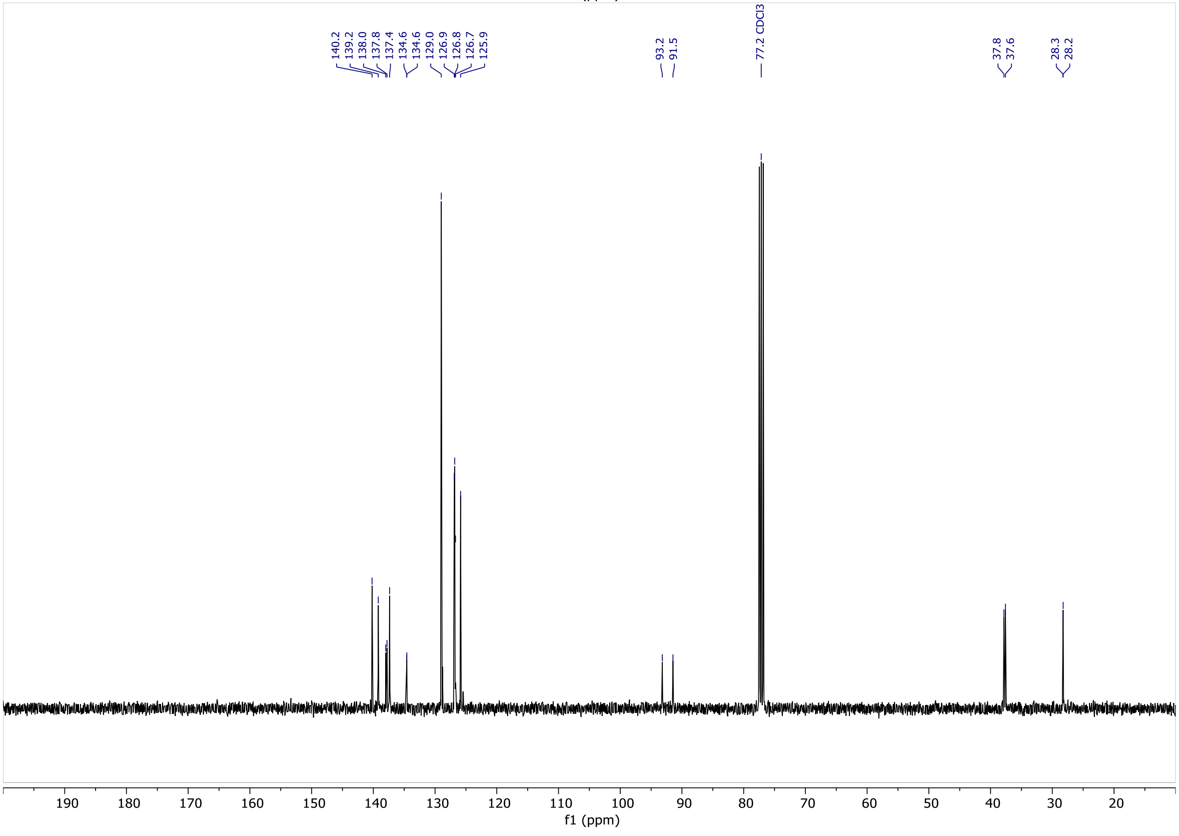
140.2 139.2 138.0 137.8 137.4 134.6 134.6 129.0 126.9 126.8 126.7 125.9
93.2 91.5
77.2 CDCl3
37.8 37.6
28.3 28.2
f1 (ppm)

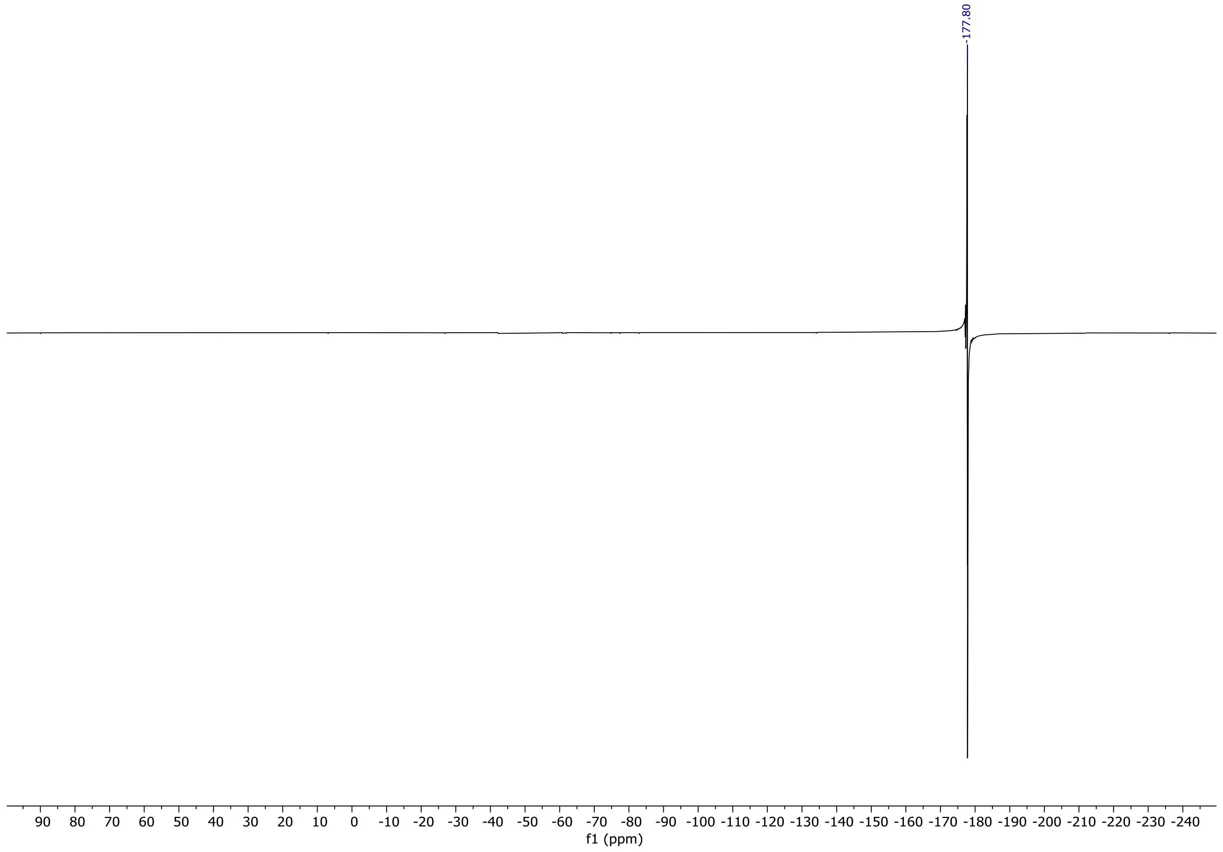
-177.80
90 80 70 60 50 40 30 20 10 0 -10 -20 -30 -40 -50 -60 -70 -80 -90 -100 -110 -120 -130 -140 -150 -160 -170 -180 -190 -200 -210 -220 -230 -240
f1 (ppm)

3-(3-(4-chlorophenyl)-3-hydroxypropyl)pyridine 1-oxide (53)

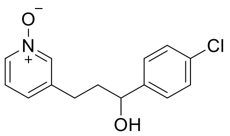

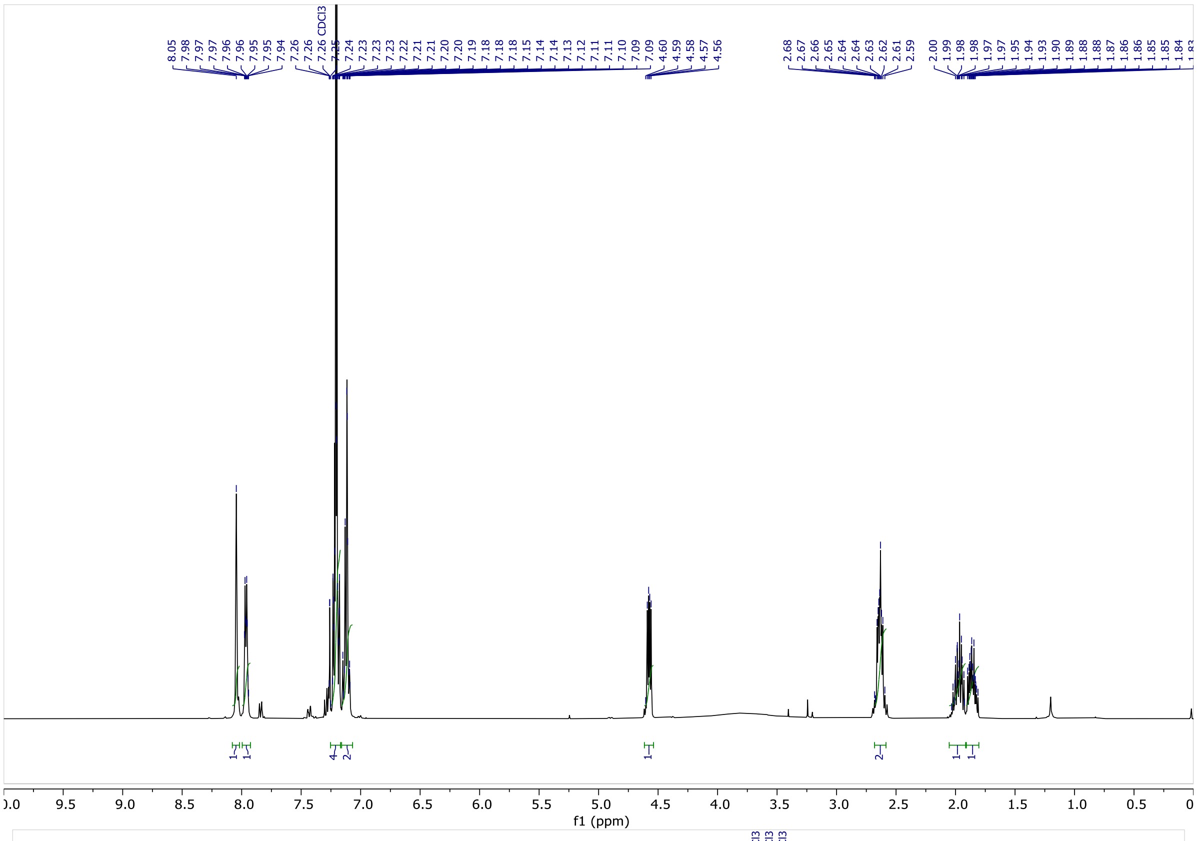

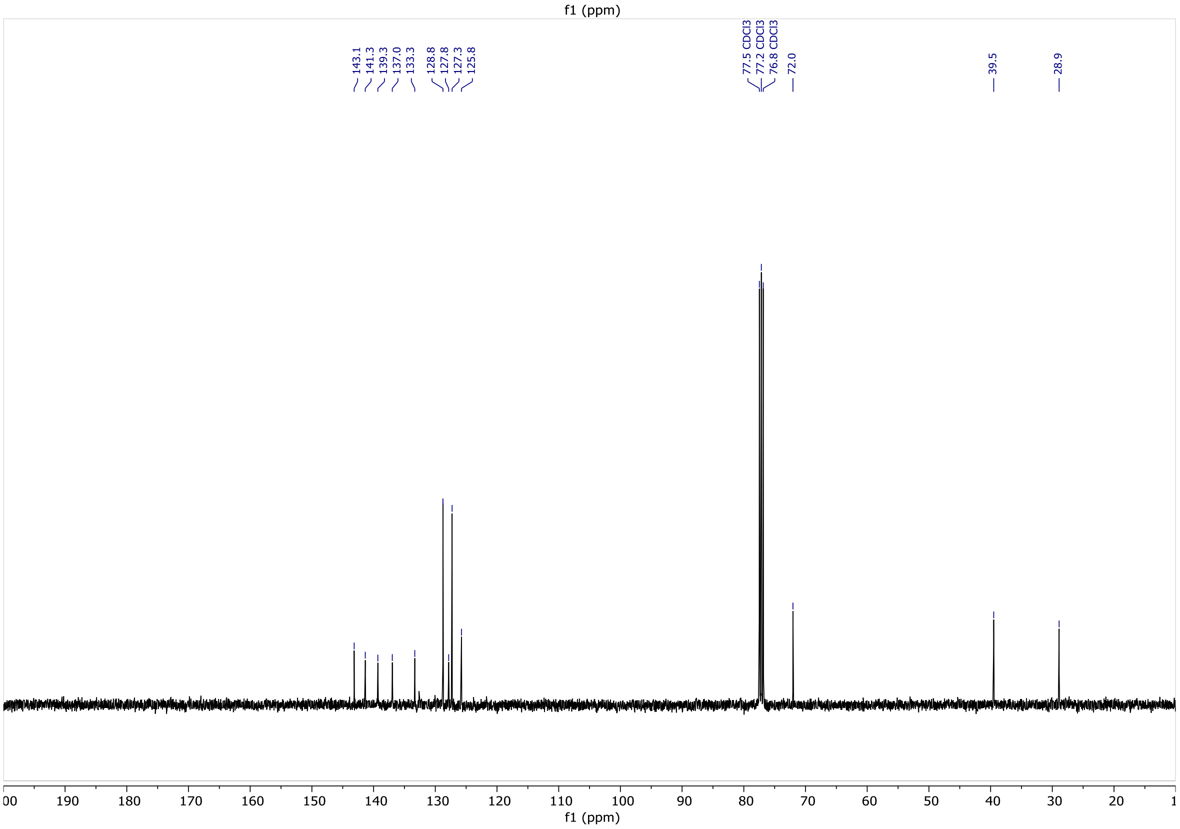

3-(3-(4-chlorophenyl)-3-oxopropyl)pyridine 1-oxide (54)

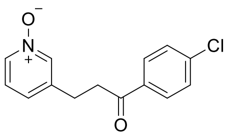

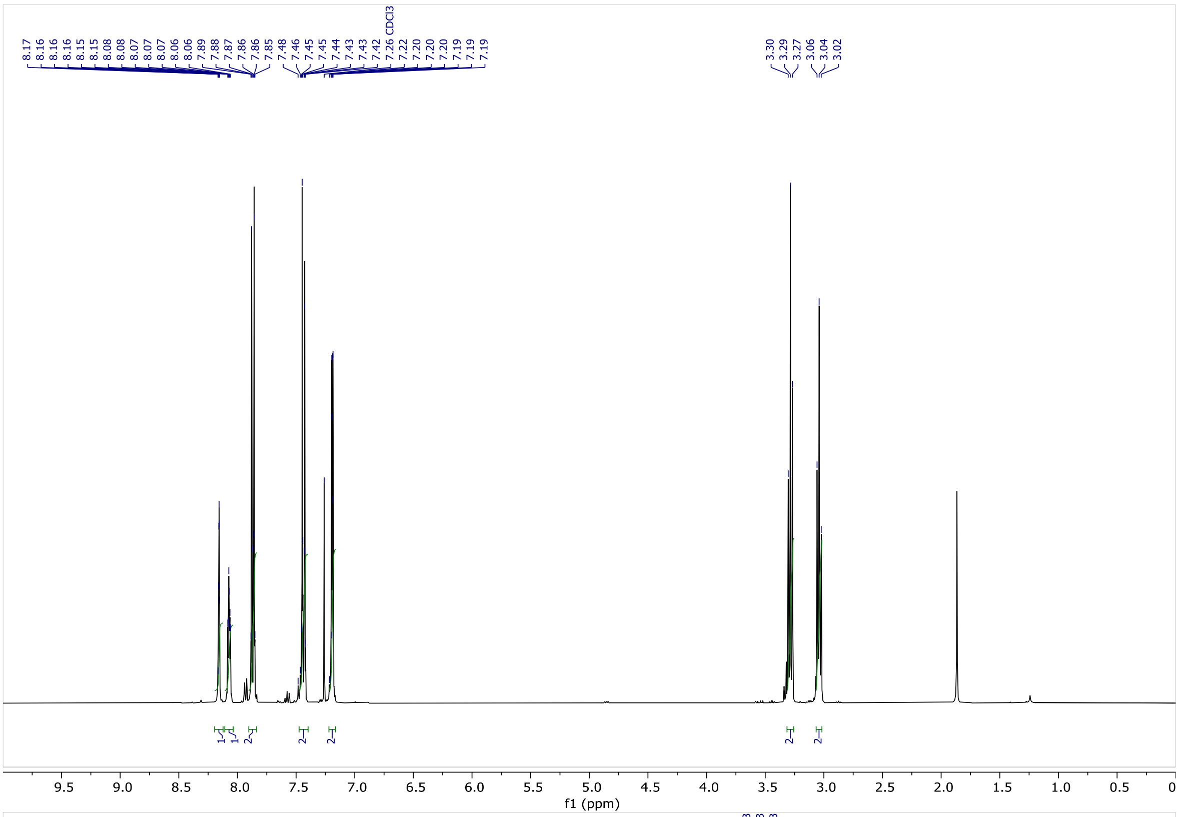

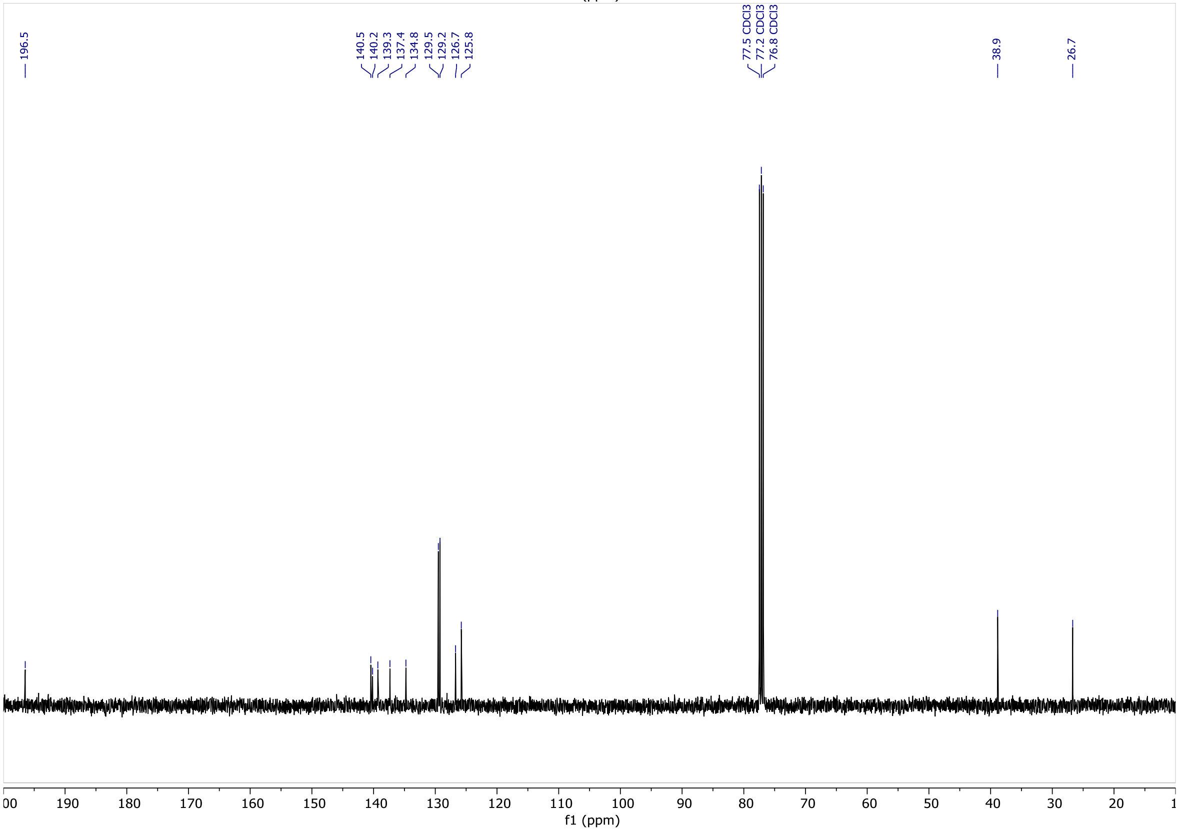

3-bromopyridine 1-oxide (55a)

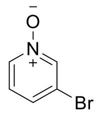

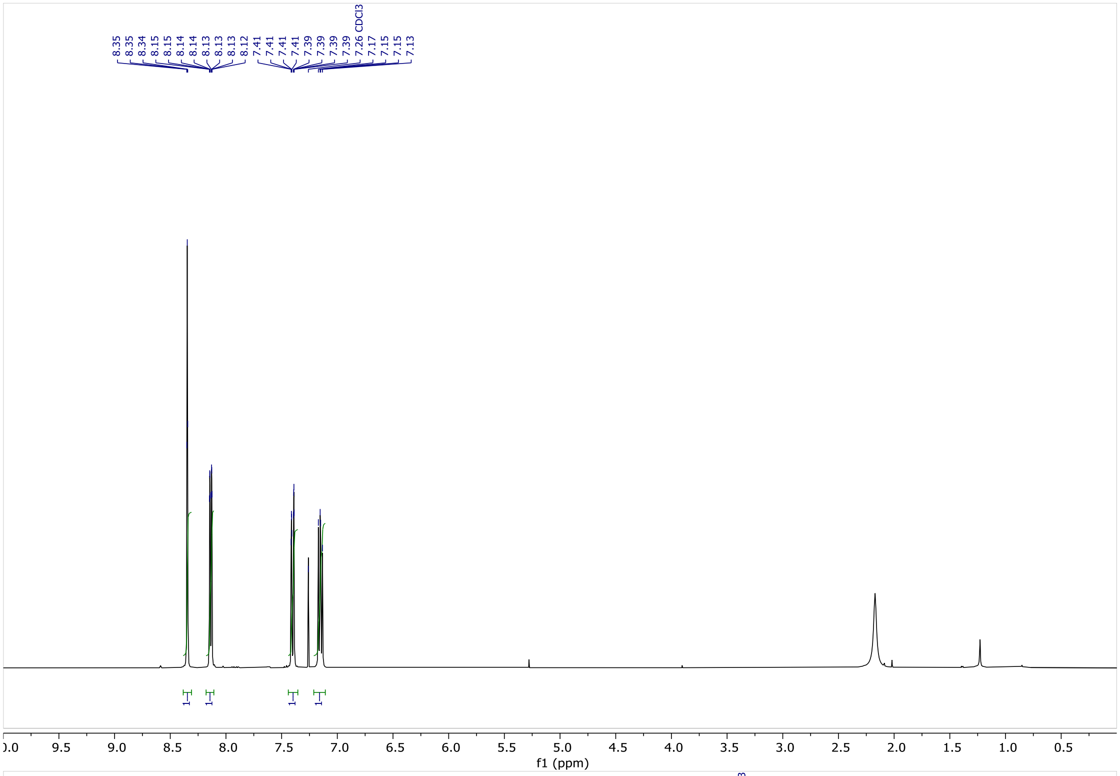

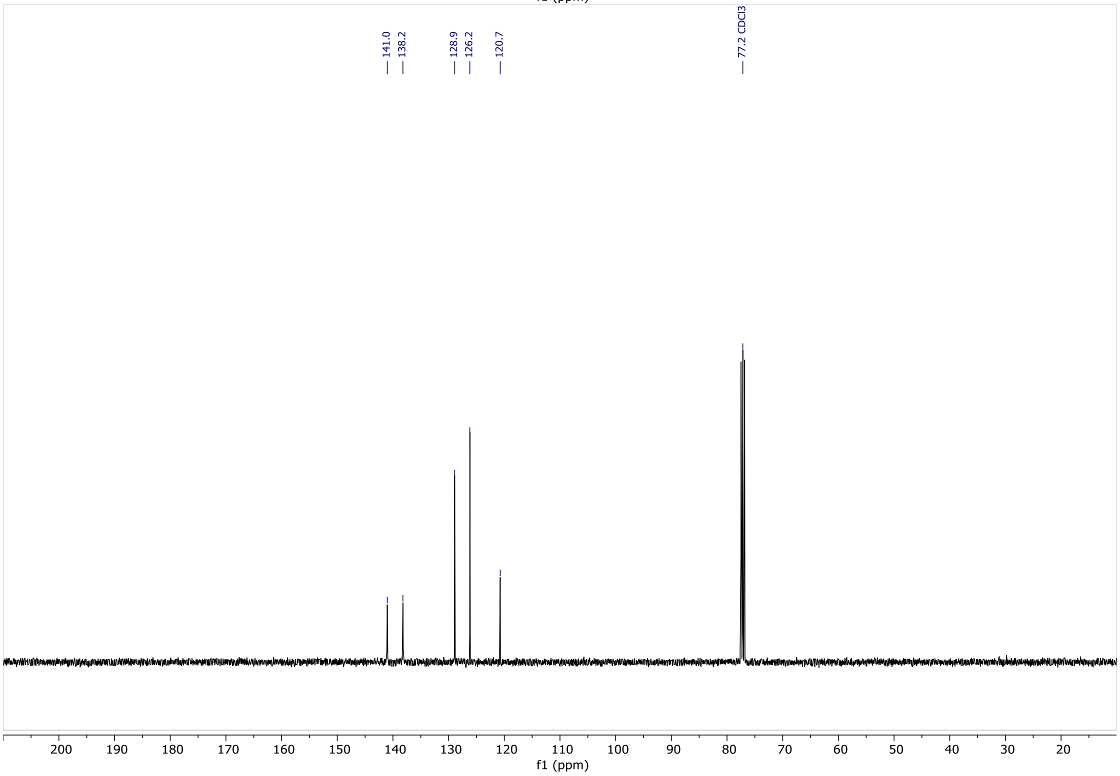

3-((4-chlorophenethyl)amino)pyridine 1-oxide (55)

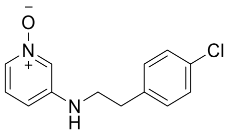

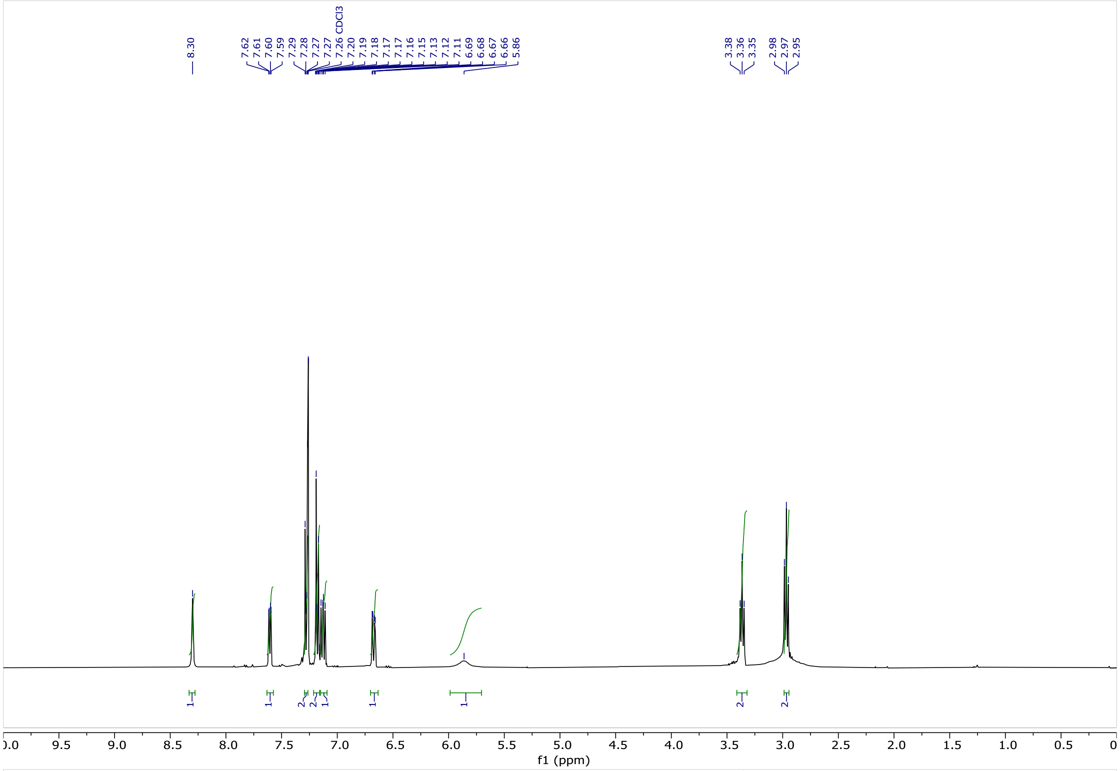

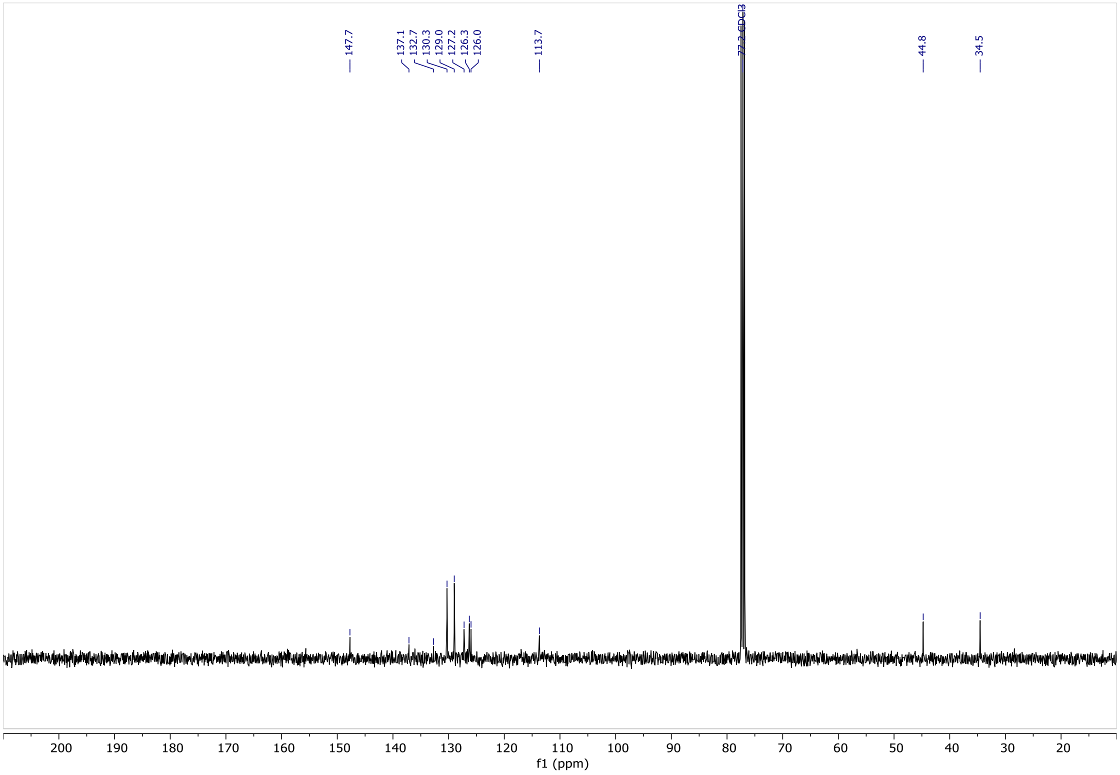

2-(6-methoxypyridin-2-yl)ethan-1-ol (56a)

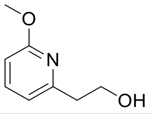

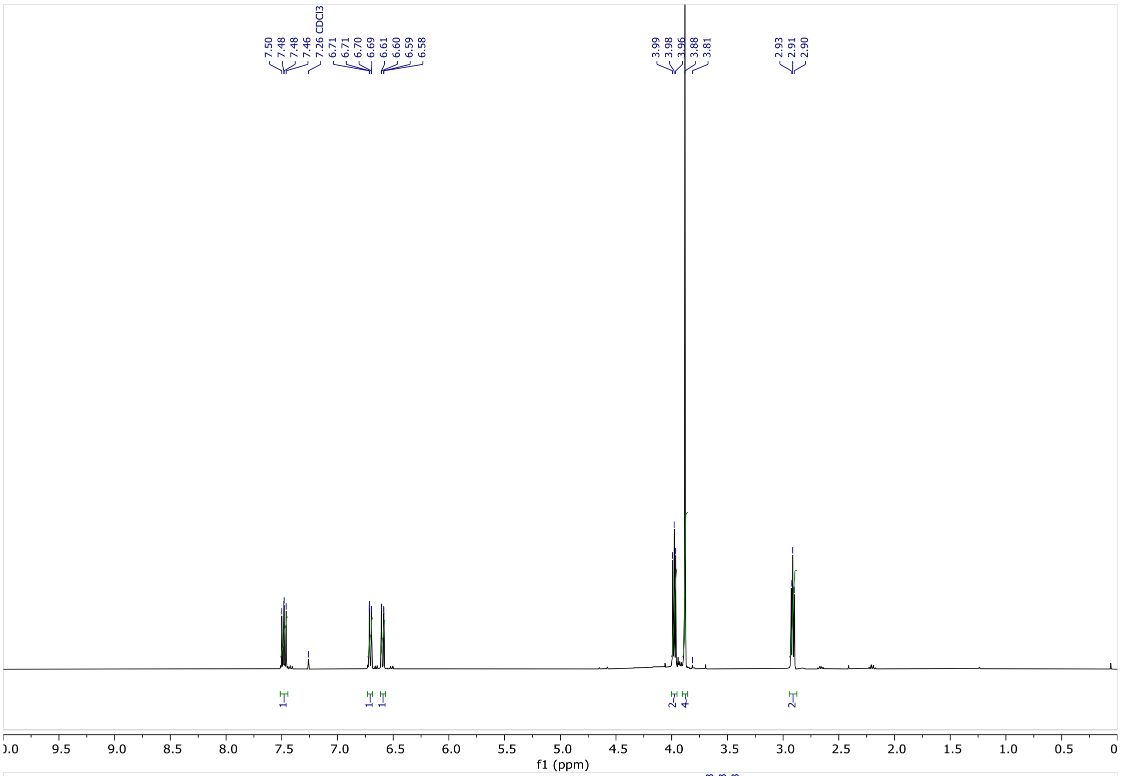

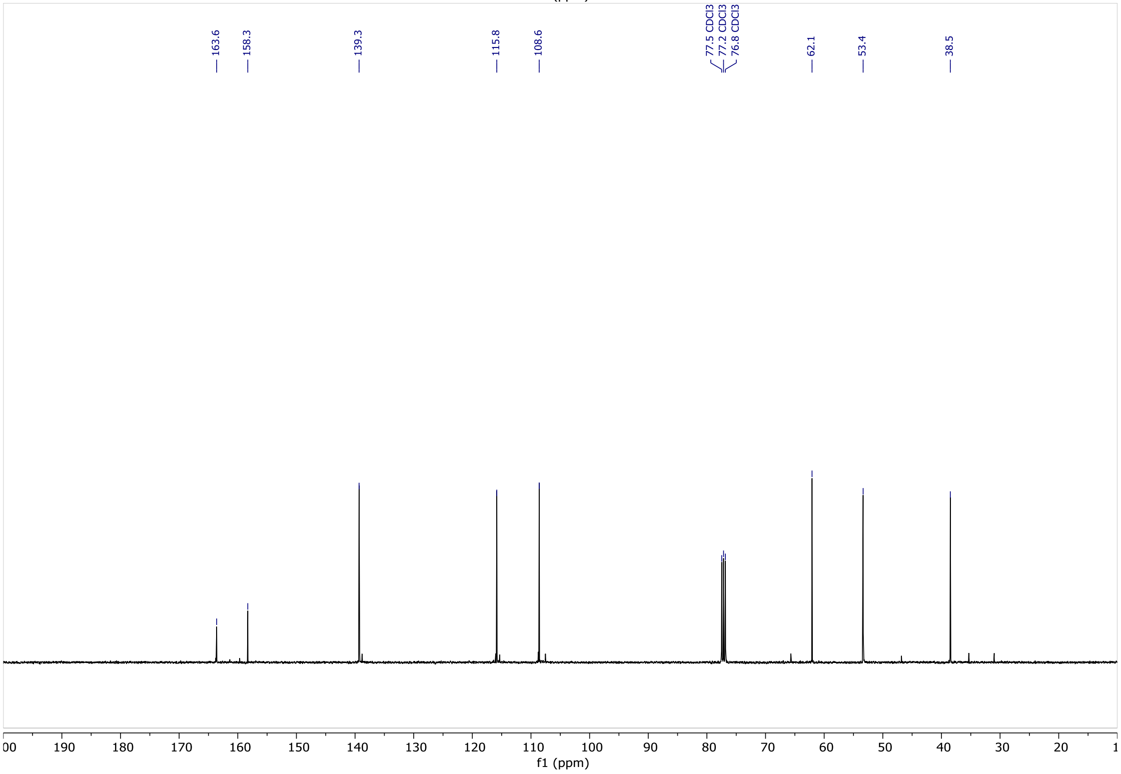

2-(2-((4-chloronaphthalen-1-yl)oxy)ethyl)-6-methoxypyridine (56)

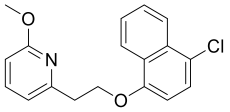

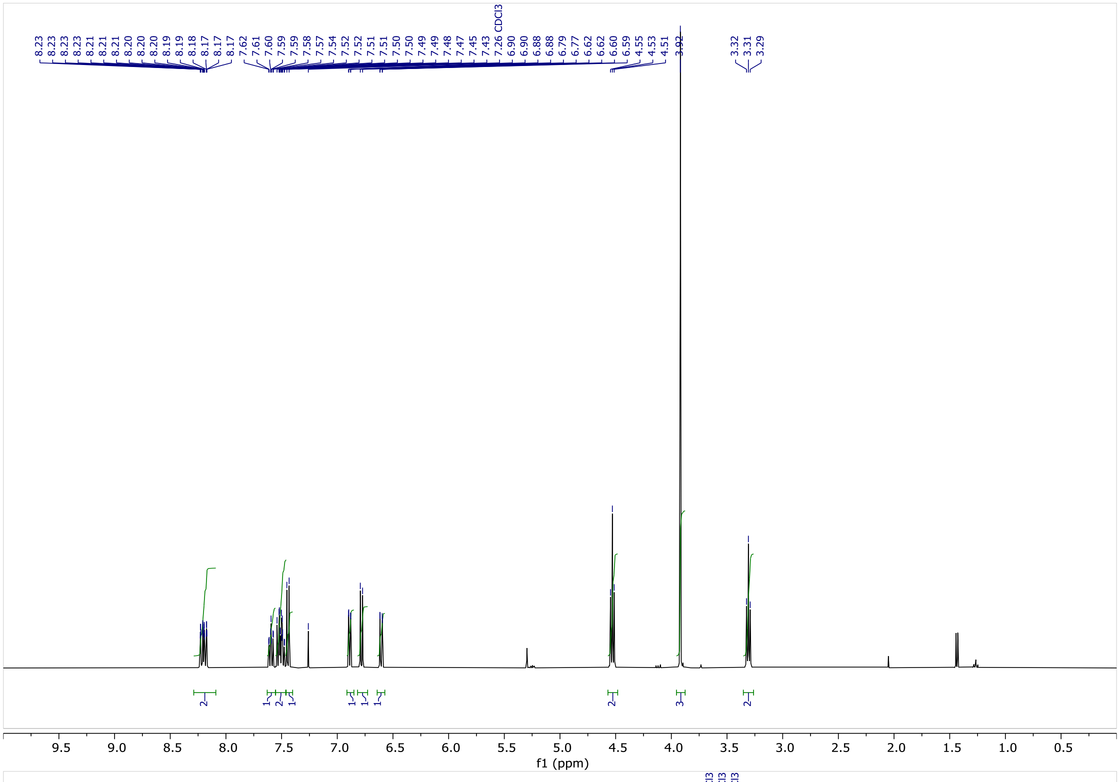

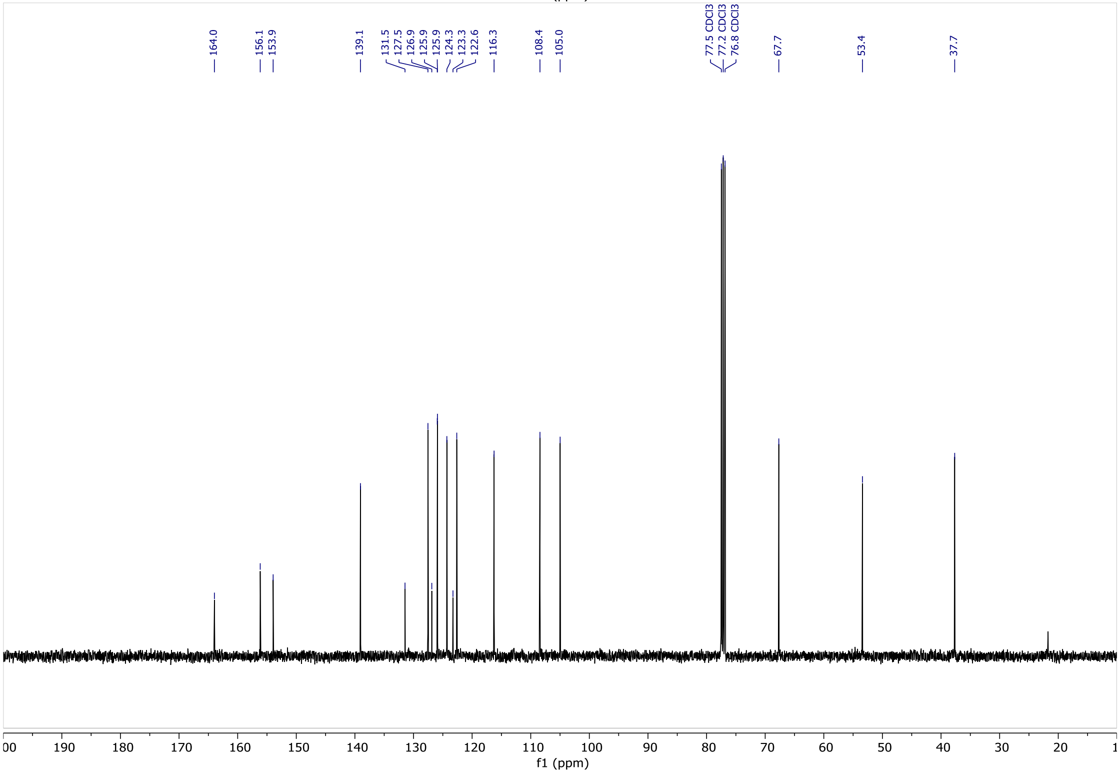

2-(2-methoxypyridin-4-yl)ethan-1-ol (57a)

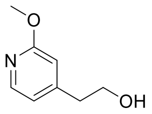

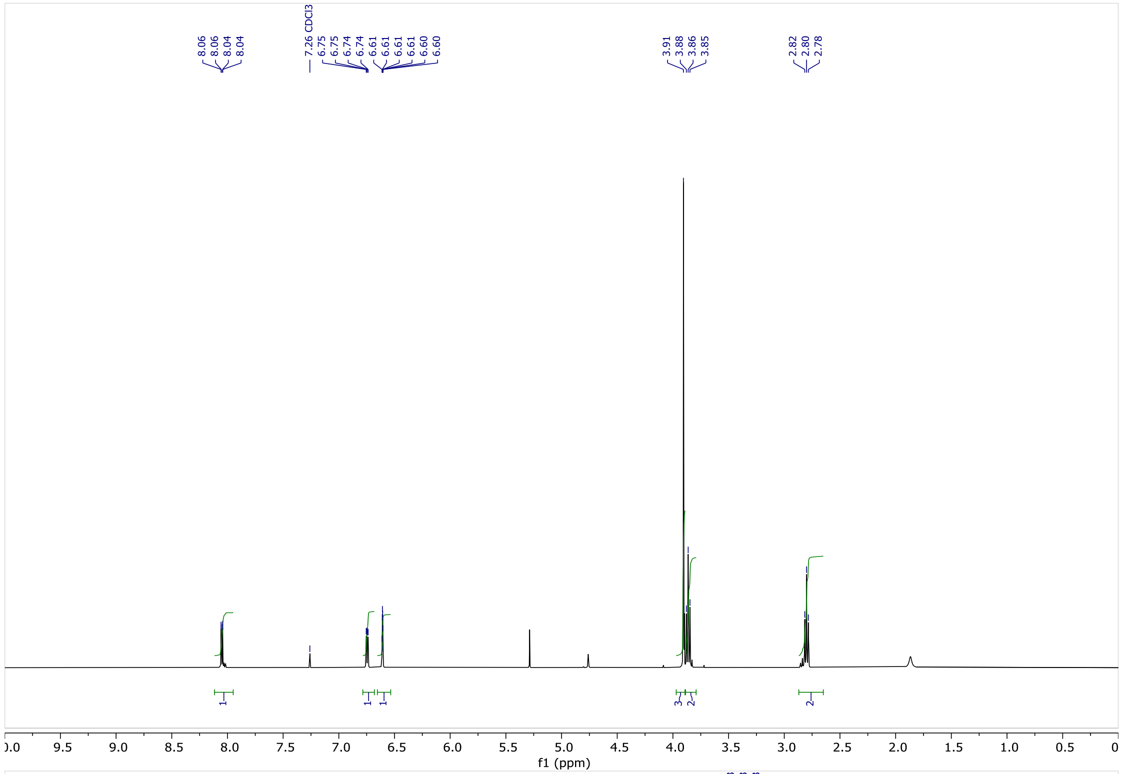

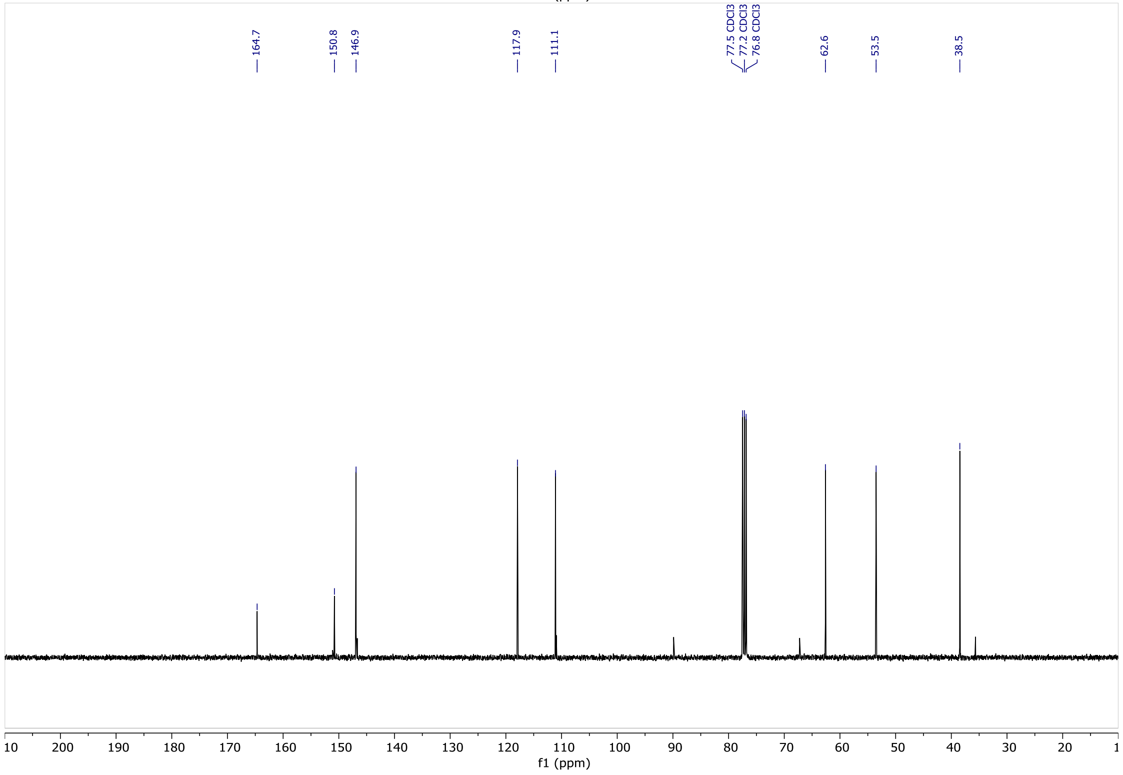

4-(2-((4-chloronaphthalen-1-yl)oxy)ethyl)-2-methoxypyridine (57)

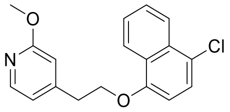

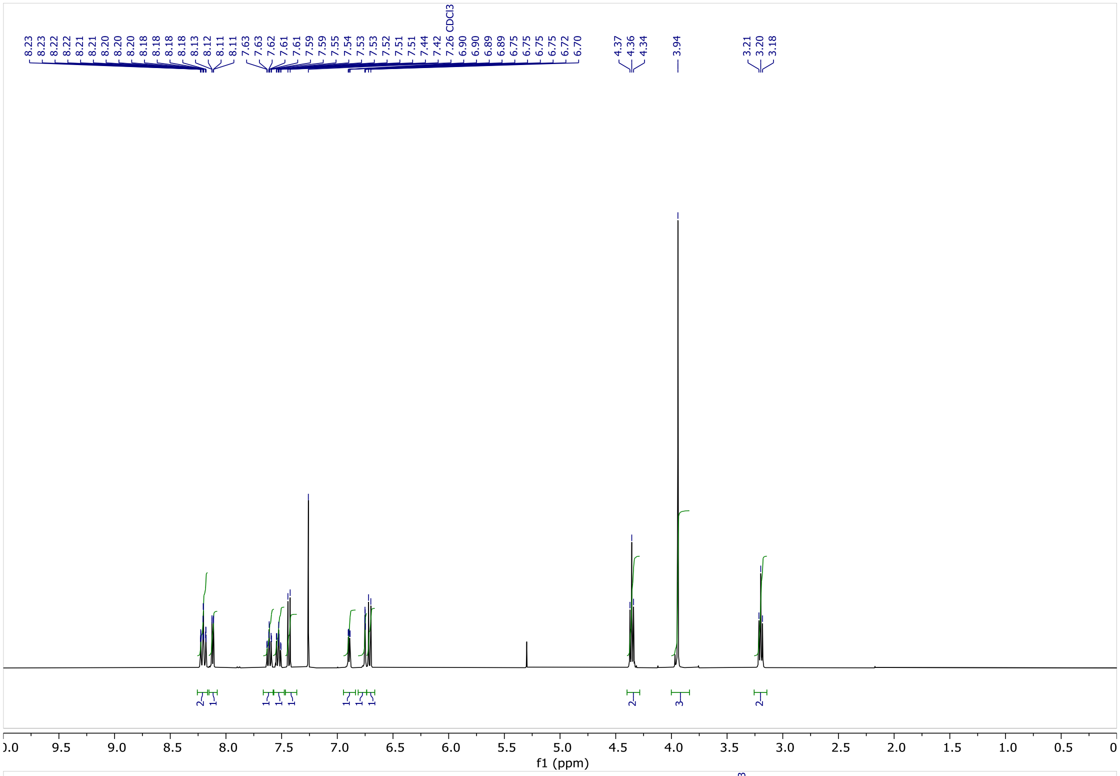

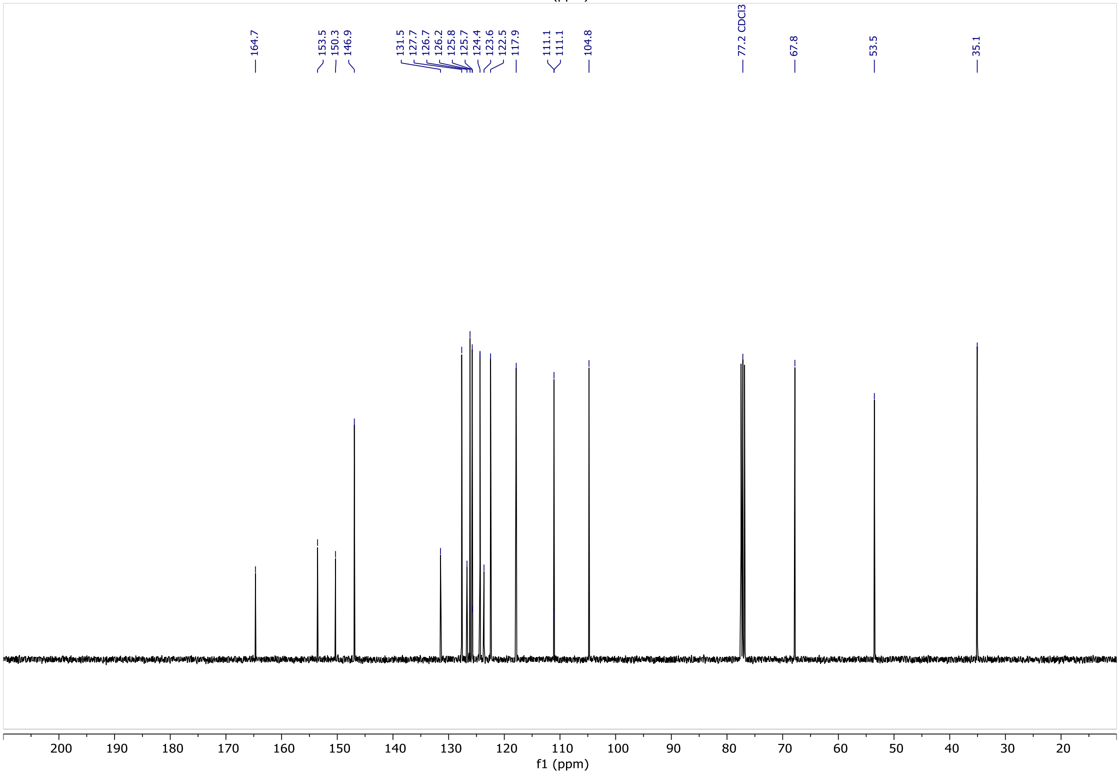

6-(2-((4-chloronaphthalen-1-yl)oxy)ethyl)pyridin-2-ol (58)

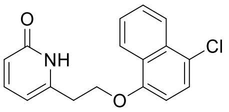

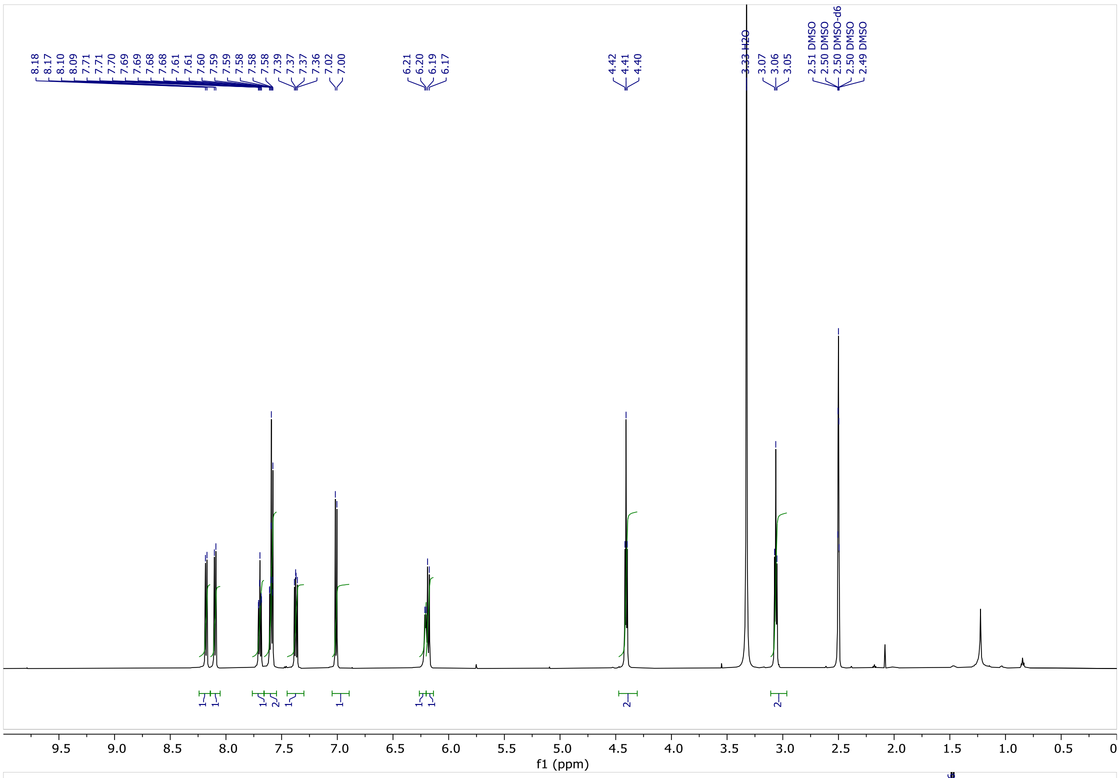

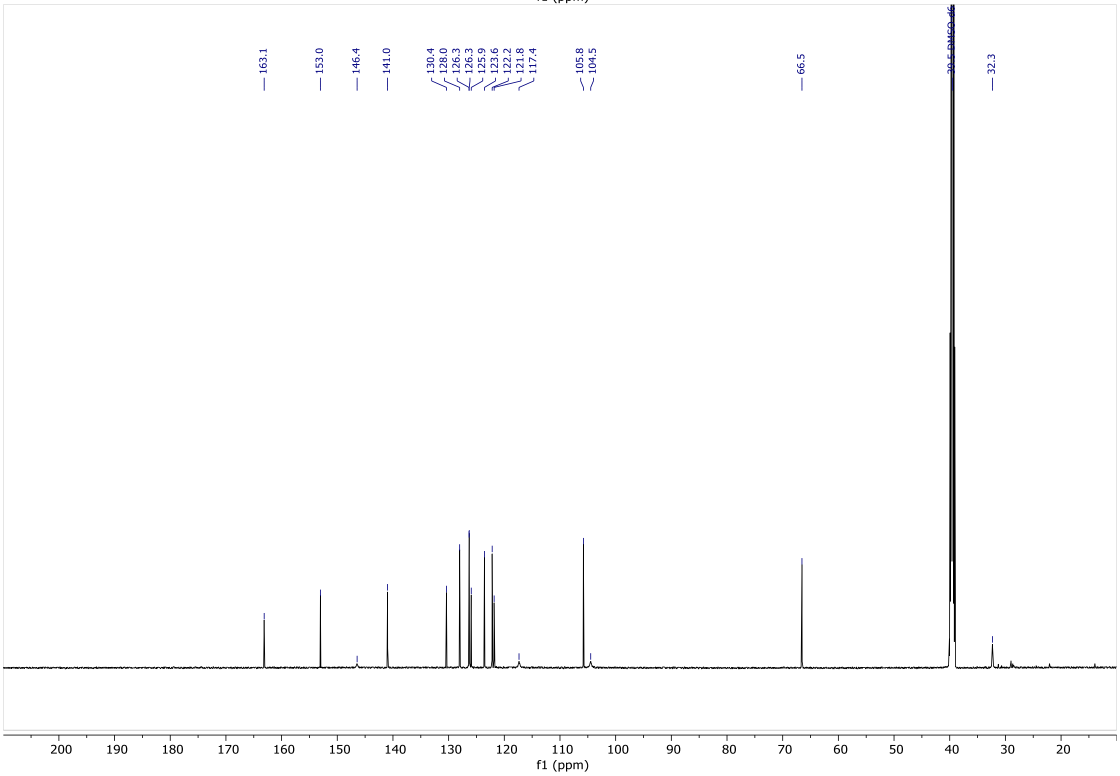

4-(2-((4-chloronaphthalen-1-yl)oxy)ethyl)pyridin-2-ol (59)

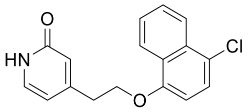

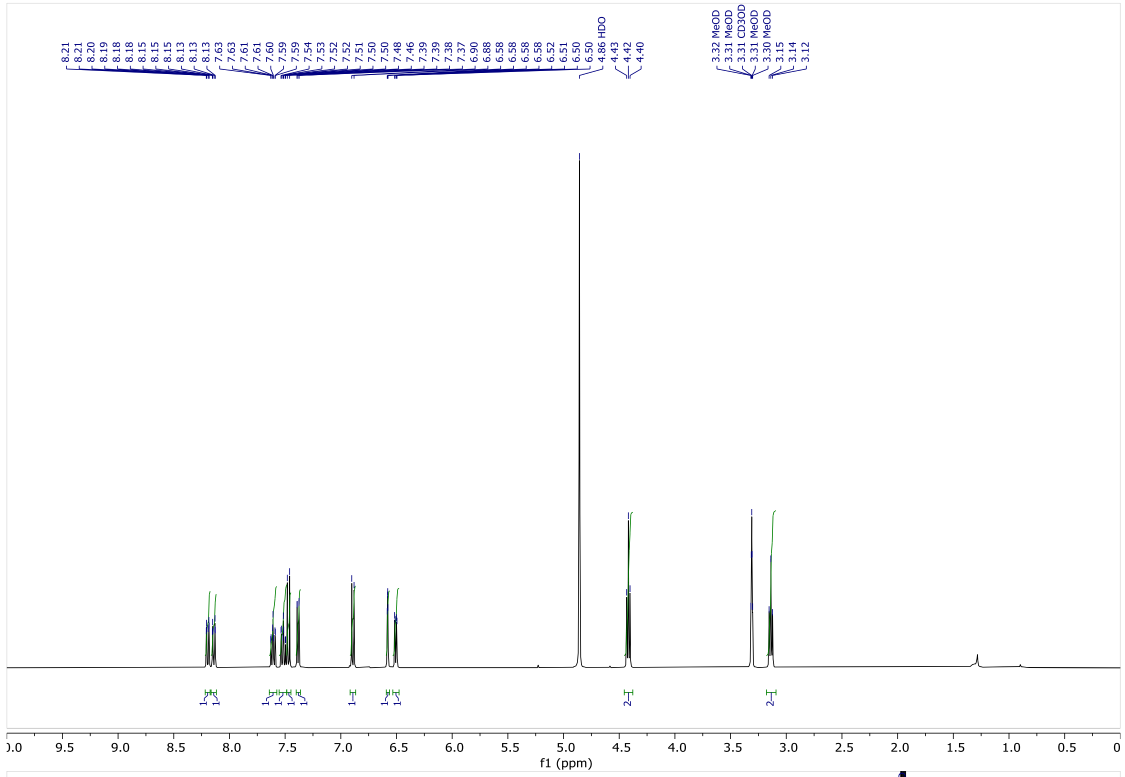

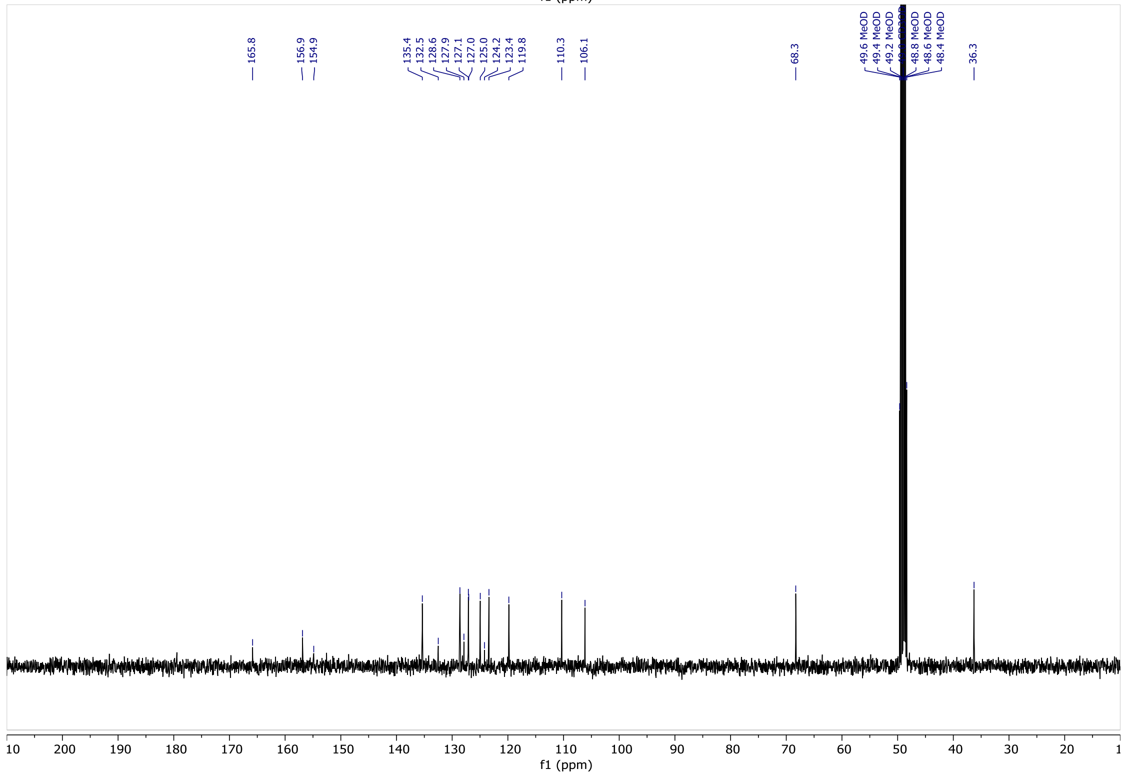

2-(2-(4-chlorophenoxy)ethyl)-6-methoxypyridine (60a)

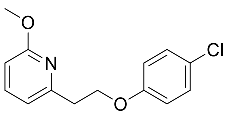

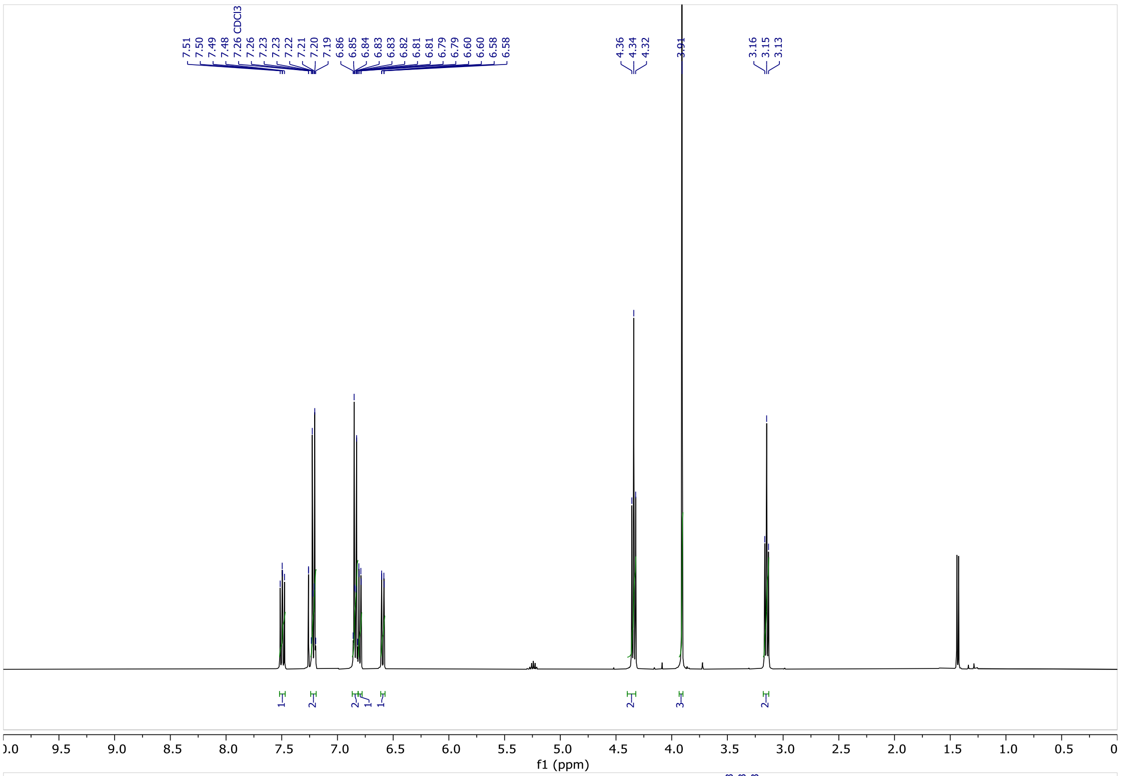

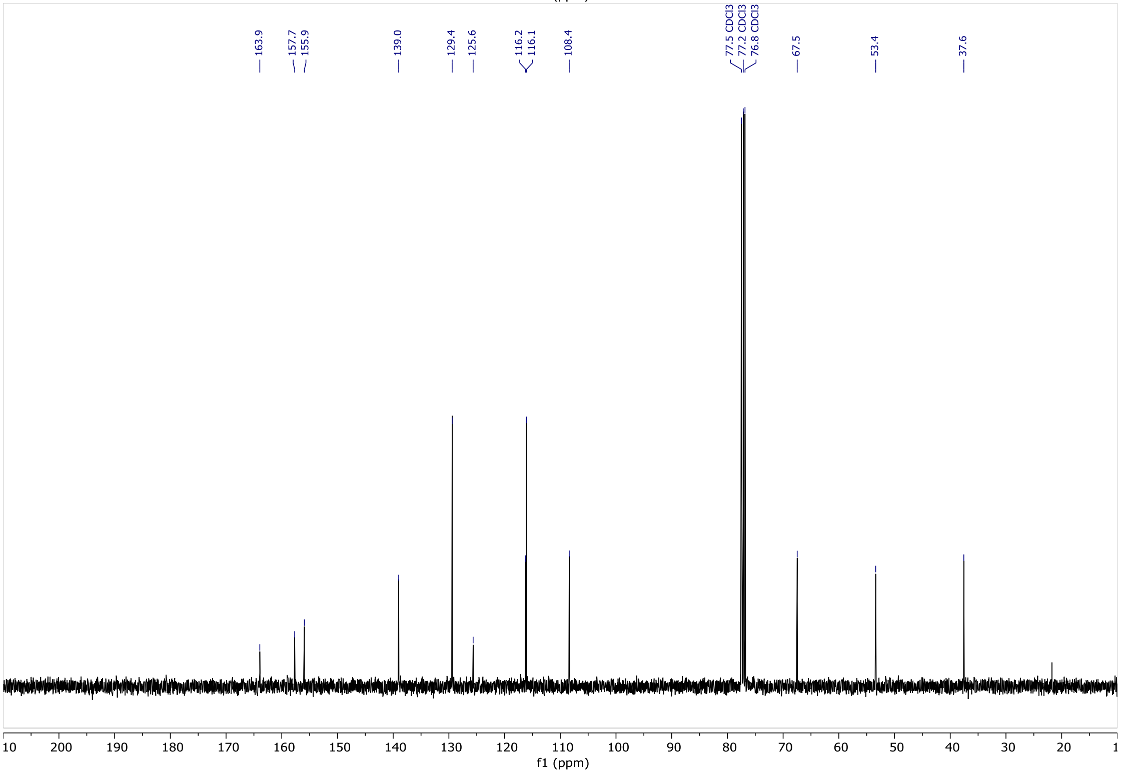

6-(2-(4-chlorophenoxy)ethyl)pyridin-2-ol (60)

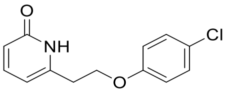

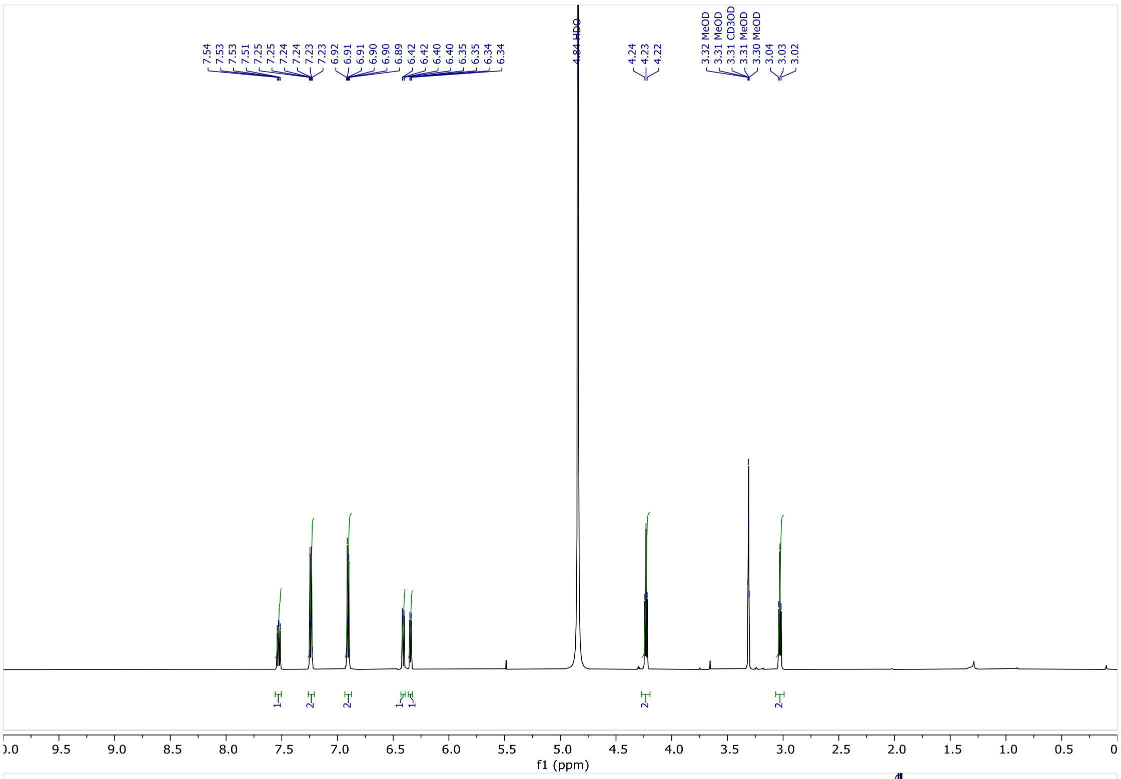

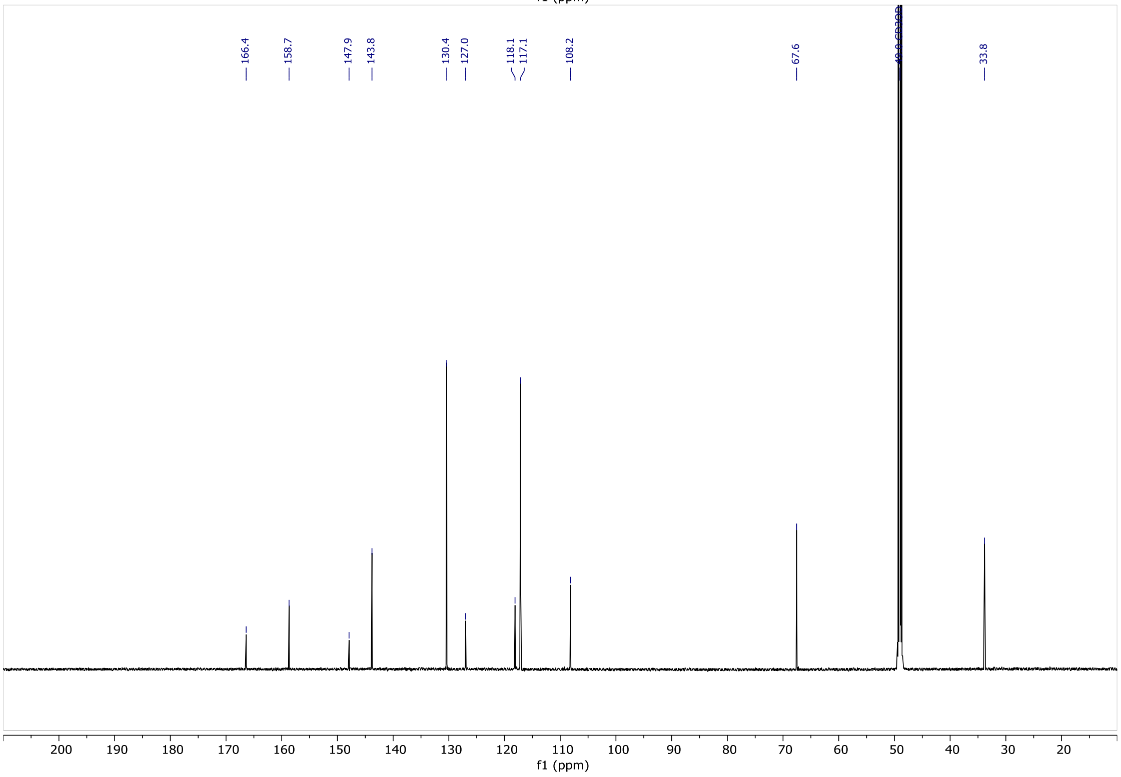

5-(2-((4-chloronaphthalen-1-yl)oxy)ethyl)-2-methylpyridine (61a)

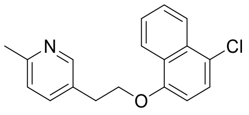

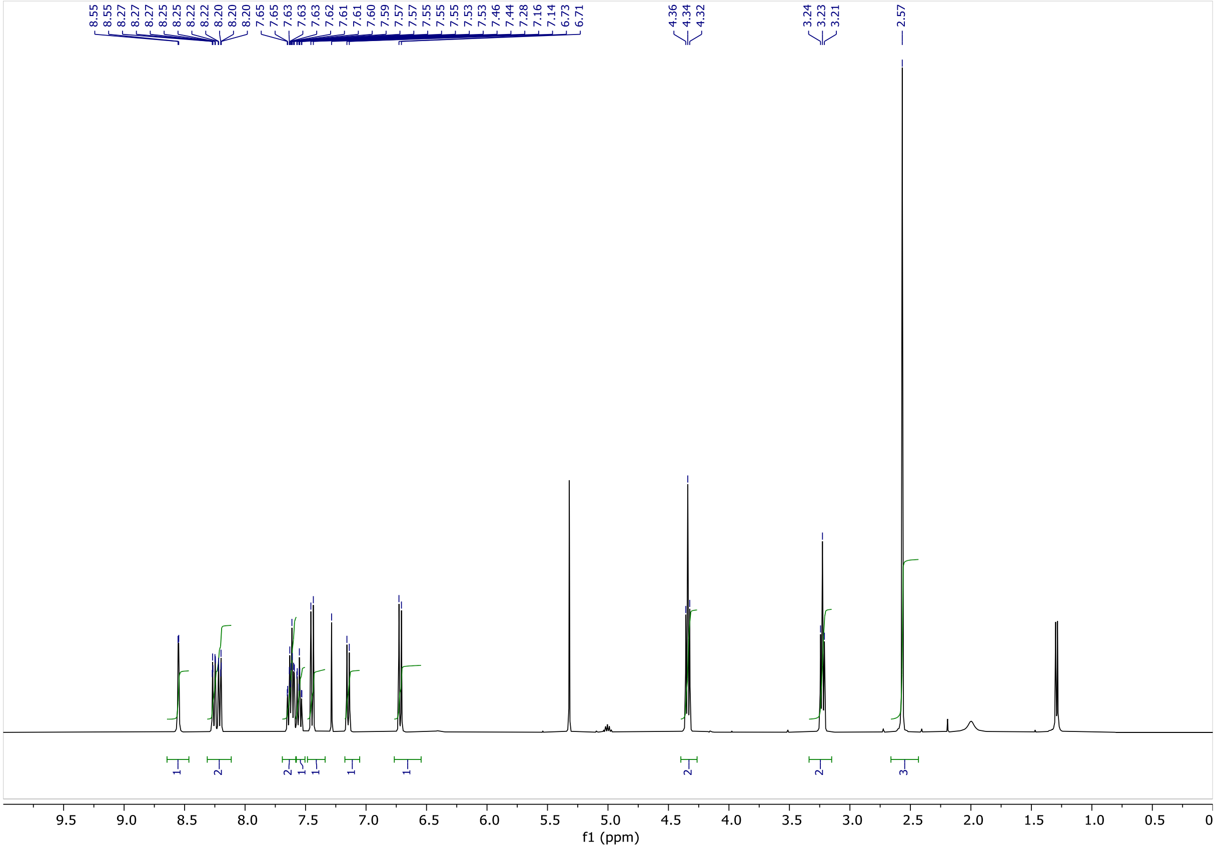

5-(2-((4-chloronaphthalen-1-yl)oxy)ethyl)-2-methylpyridine 1-oxide (61)

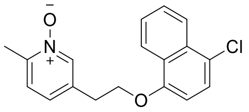

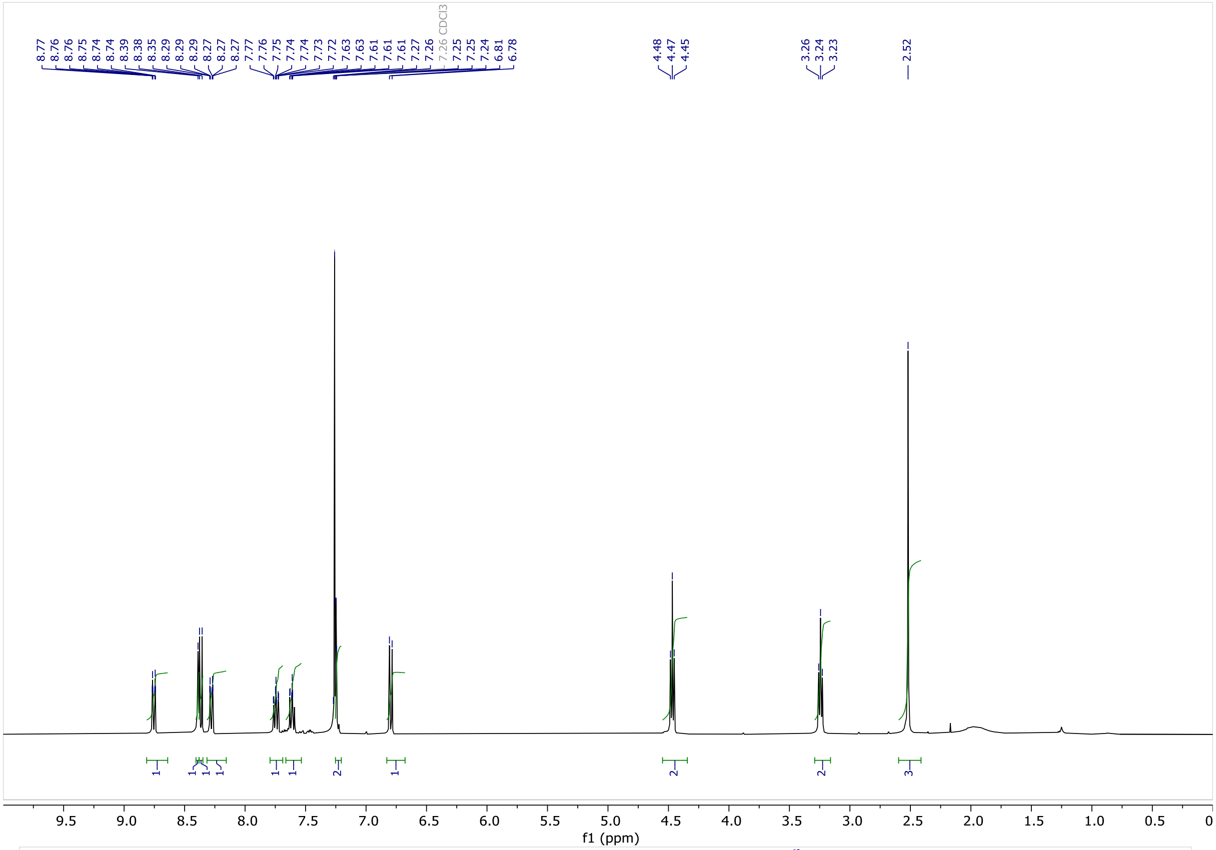

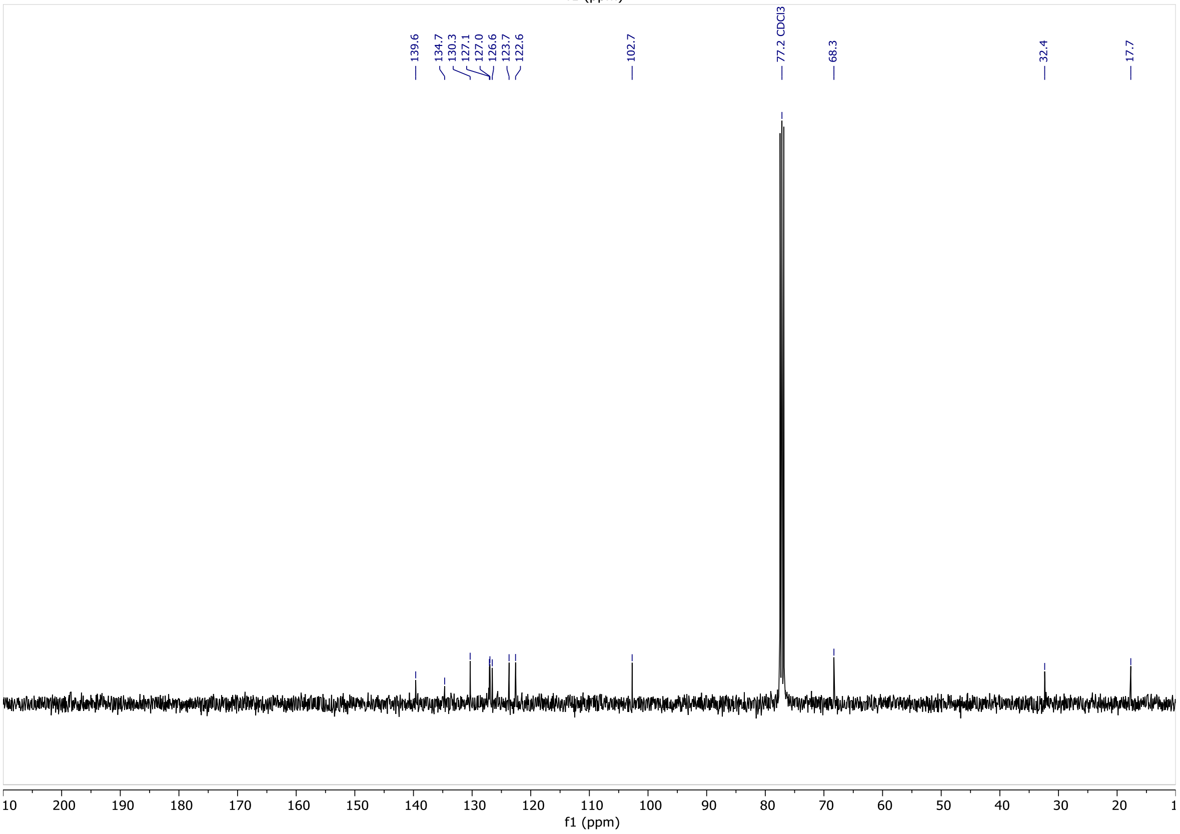

5-(2-((4-chloronaphthalen-1-yl)oxy)ethyl)-2-vinylpyridine (62b)

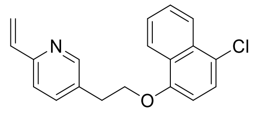

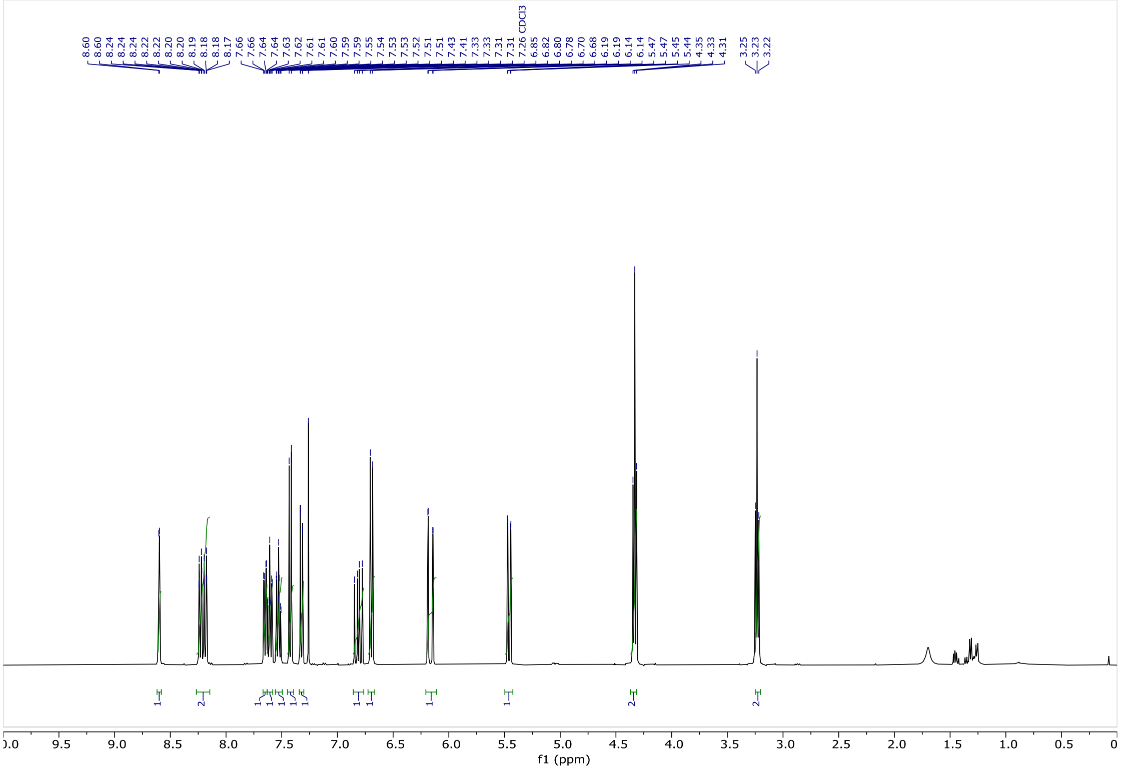

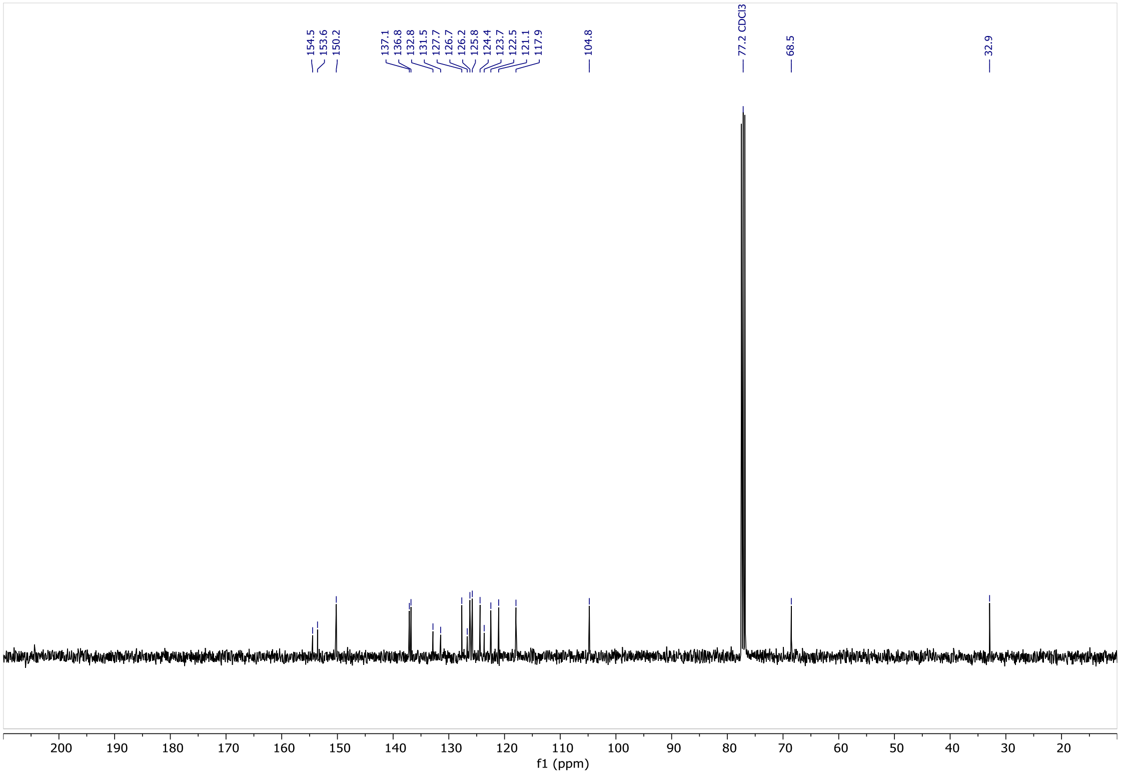

5-(2-((4-chloronaphthalen-1-yl)oxy)ethyl)-2-ethylpyridine (62a)

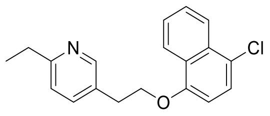

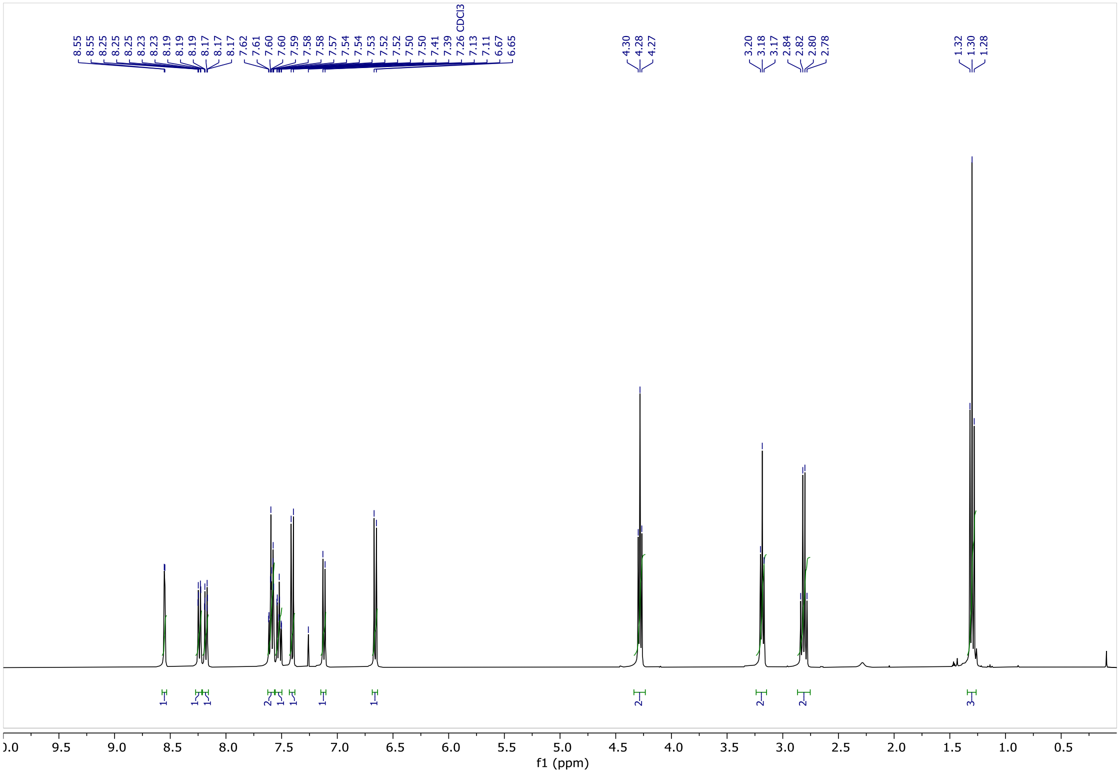

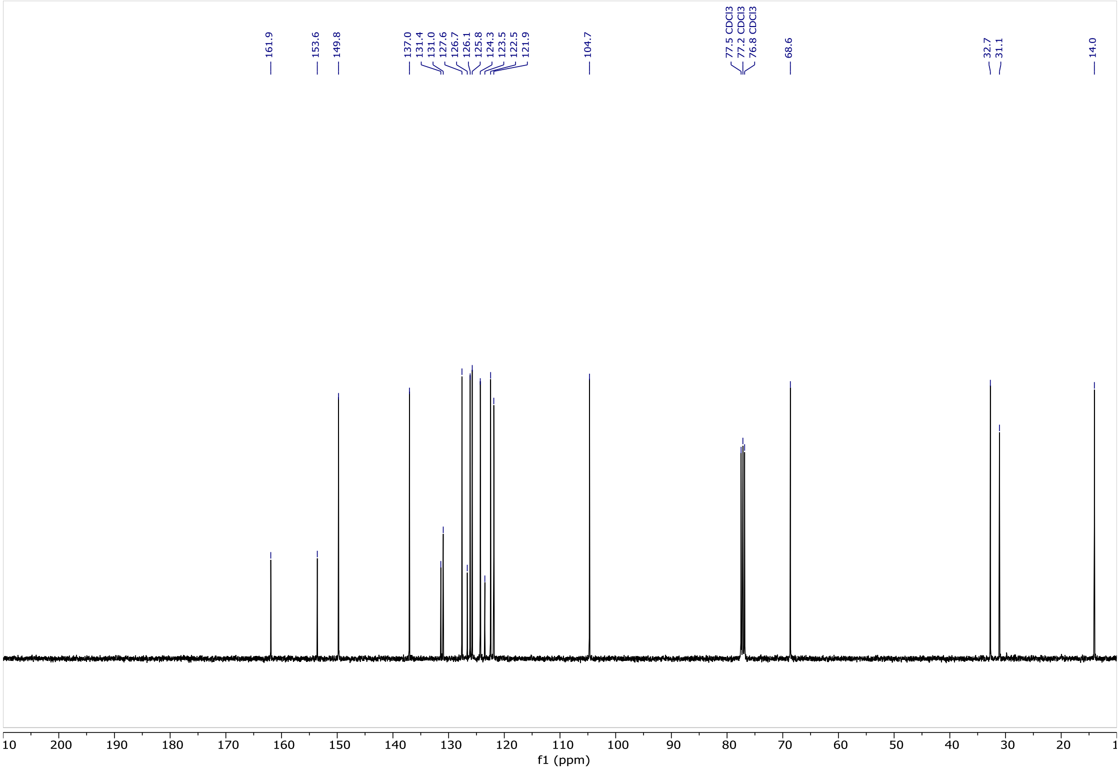

5-(2-((4-chloronaphthalen-1-yl)oxy)ethyl)-2-vinylpyridine (62)

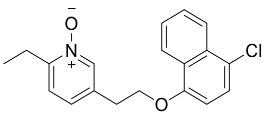

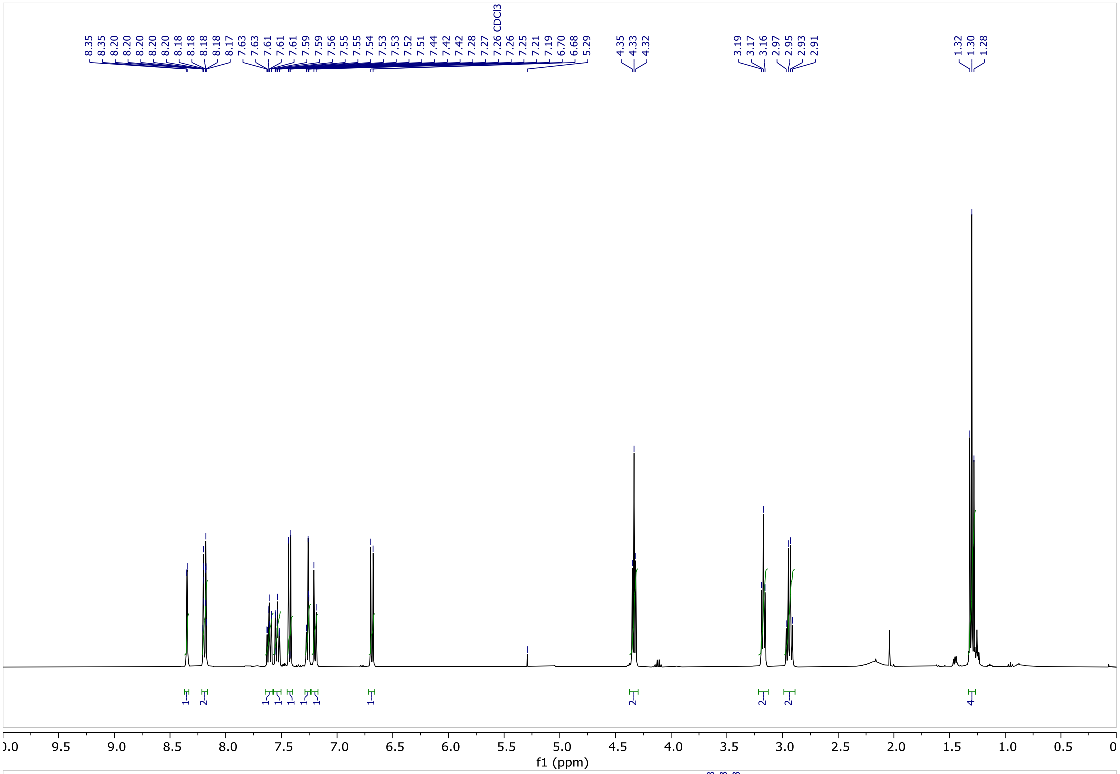

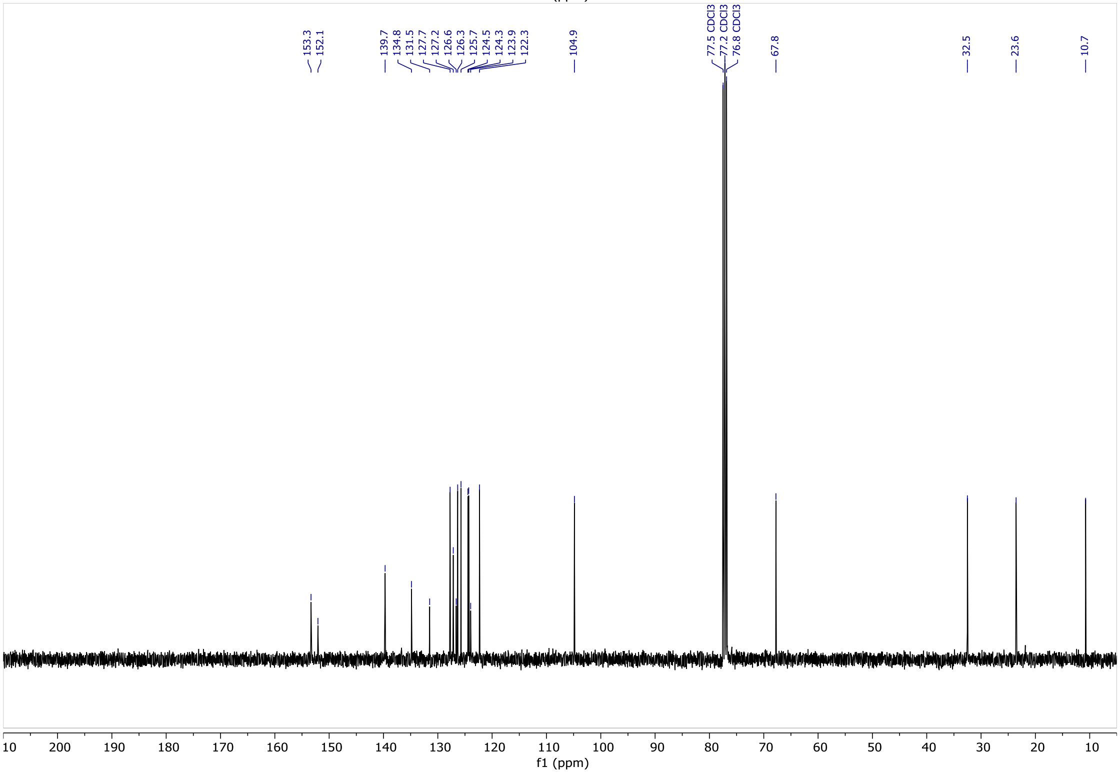

3-(2-((4-chloronaphthalen-1-yl)oxy)ethyl)-2,6-dimethylpyridine (63a)

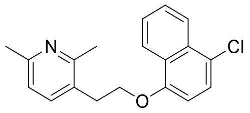

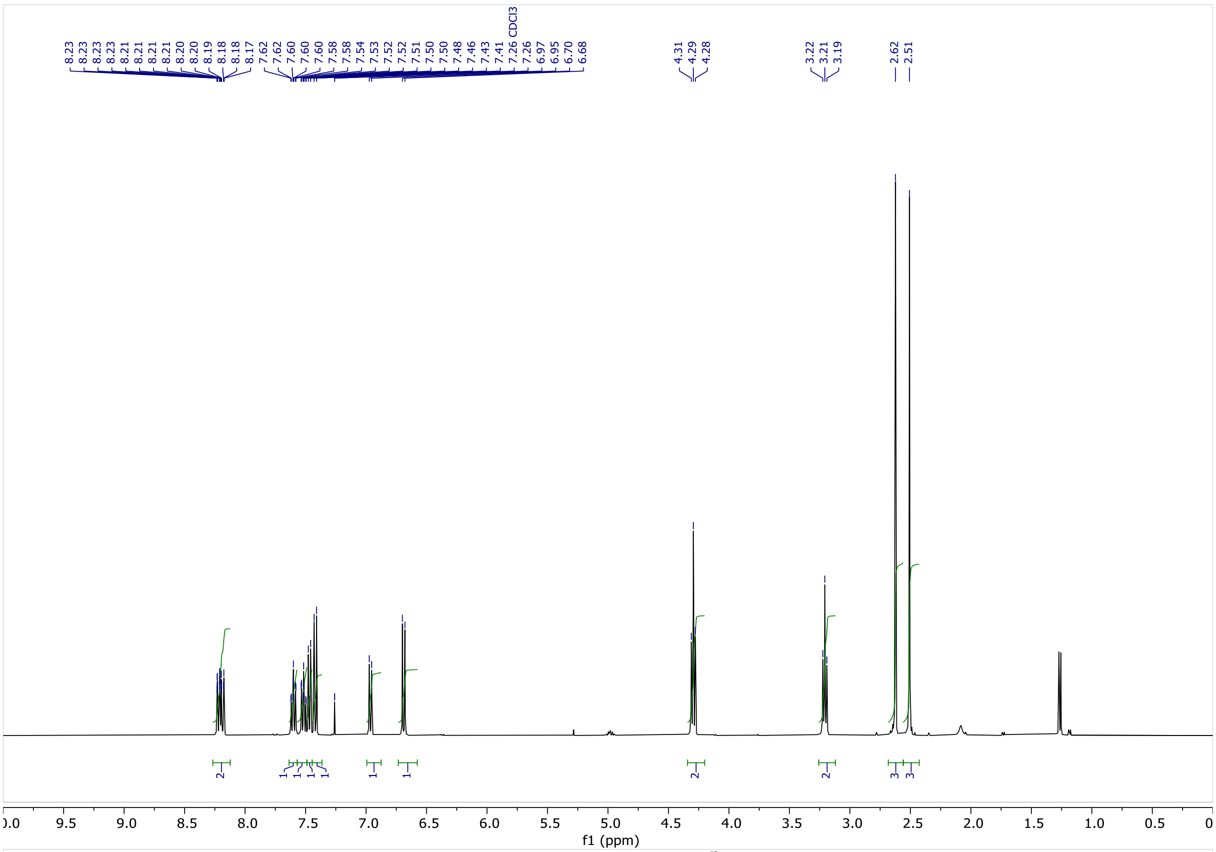

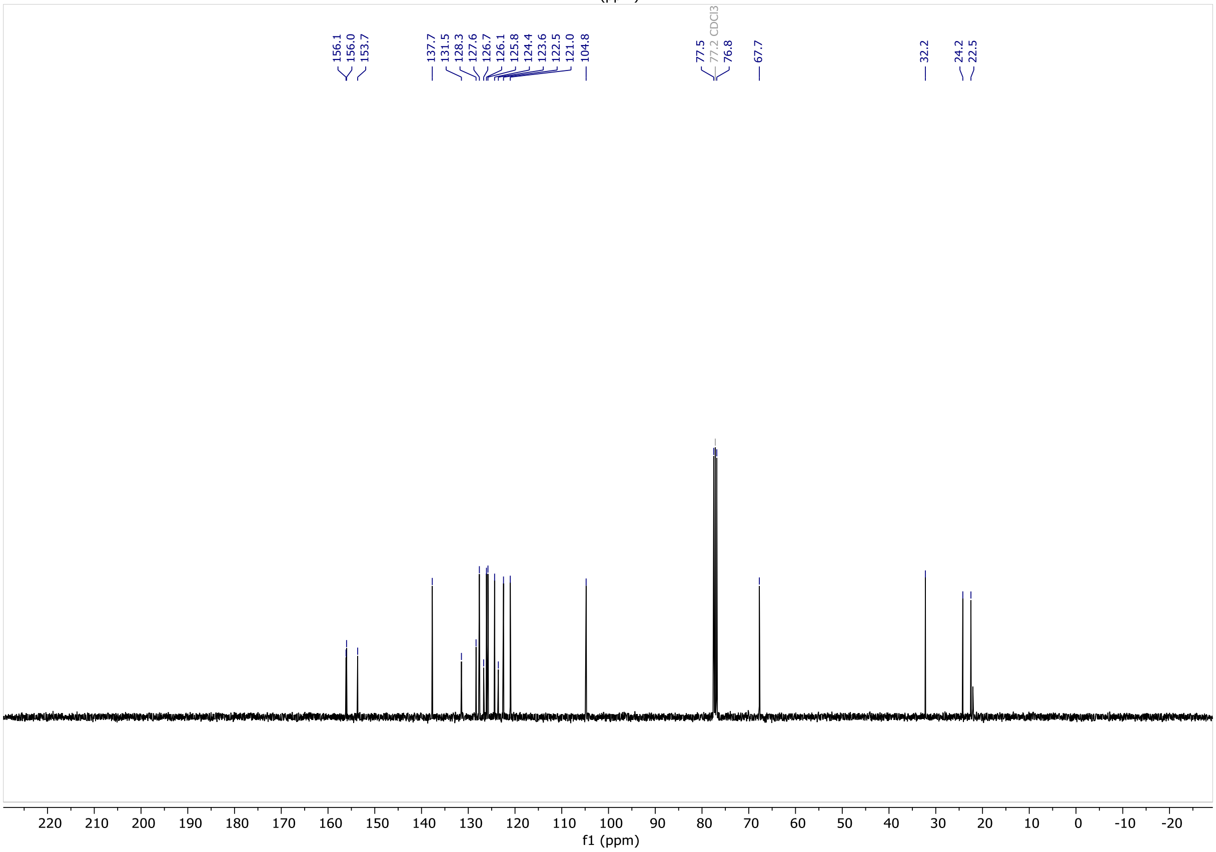

3-(2-((4-chloronaphthalen-1-yl)oxy)ethyl)-2,6-dimethylpyridine 1-oxide (63)

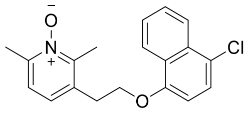

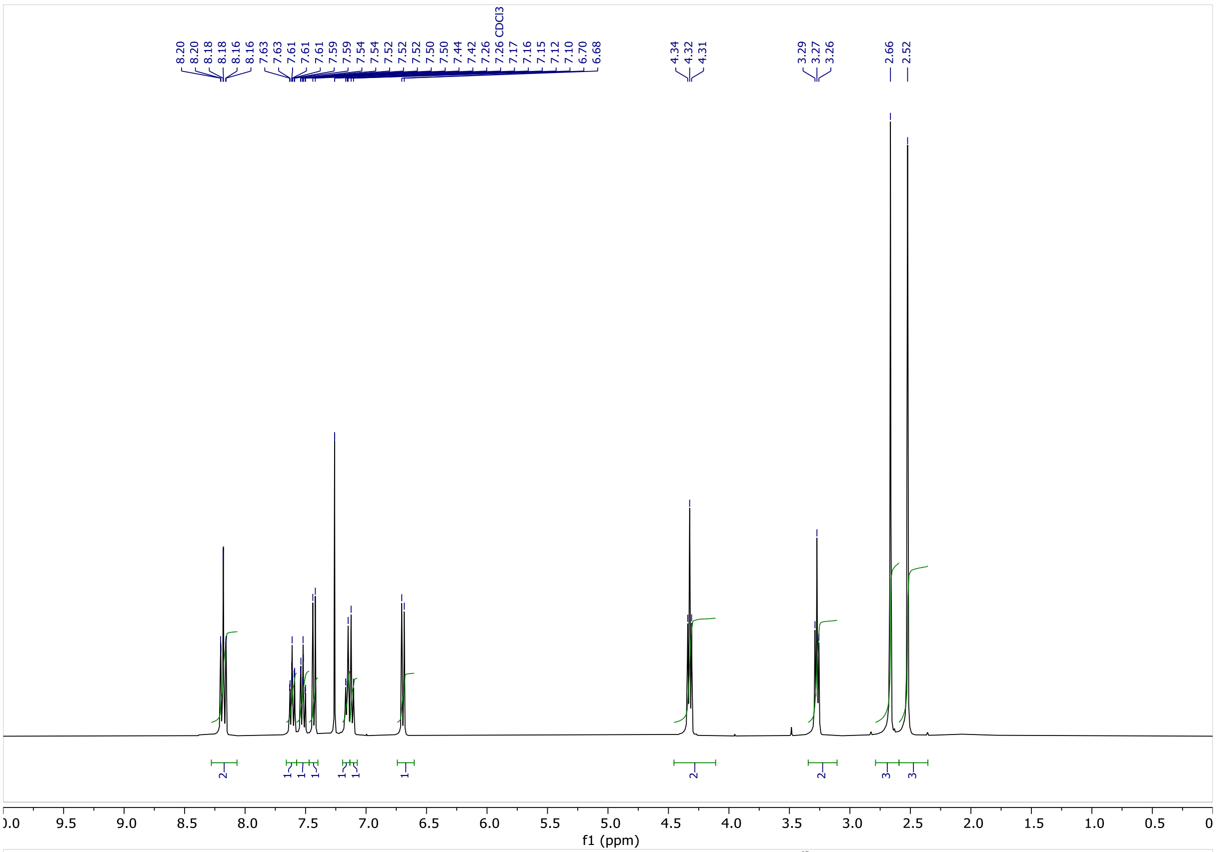

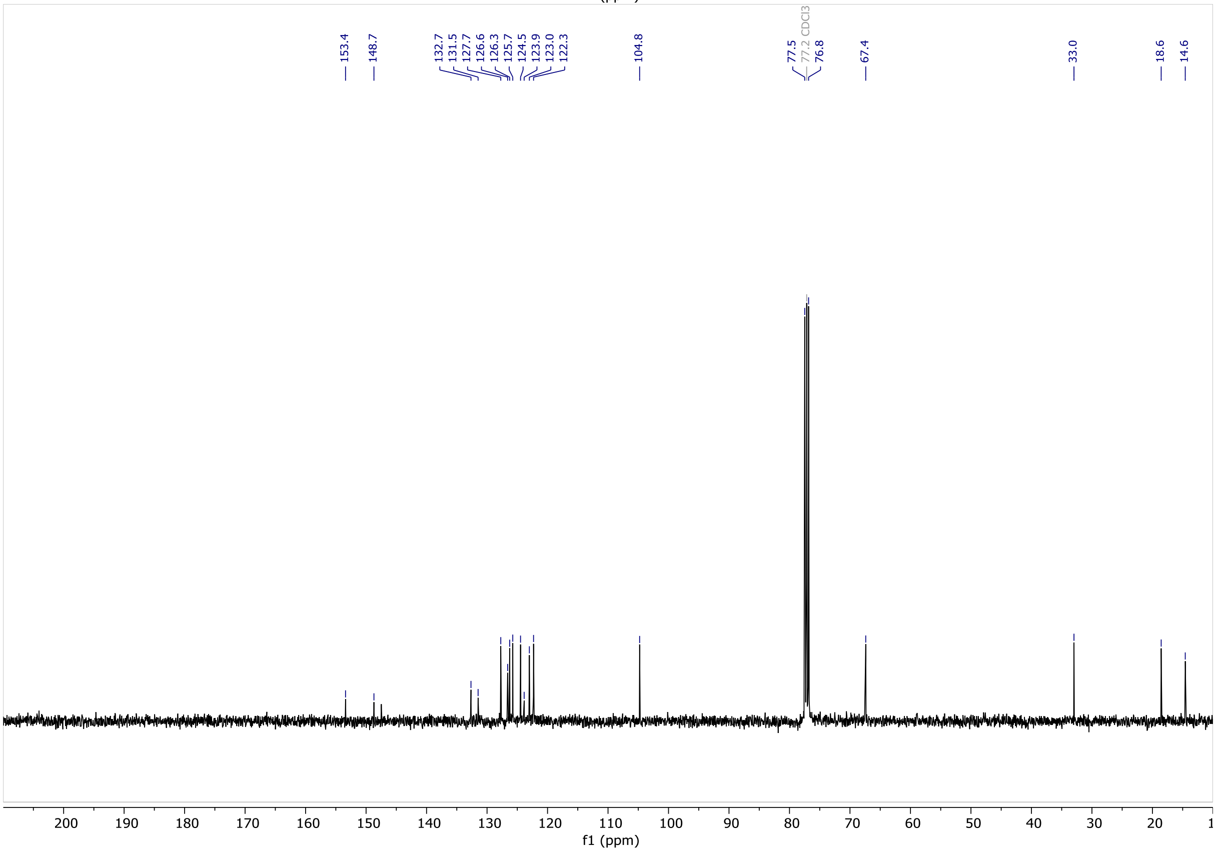

(*E*)-1-(4-chloronaphthalen-1-yl)-3-(pyridin-3-yl)prop-2-en-1-one (64c)

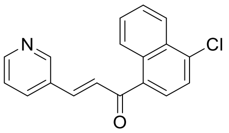

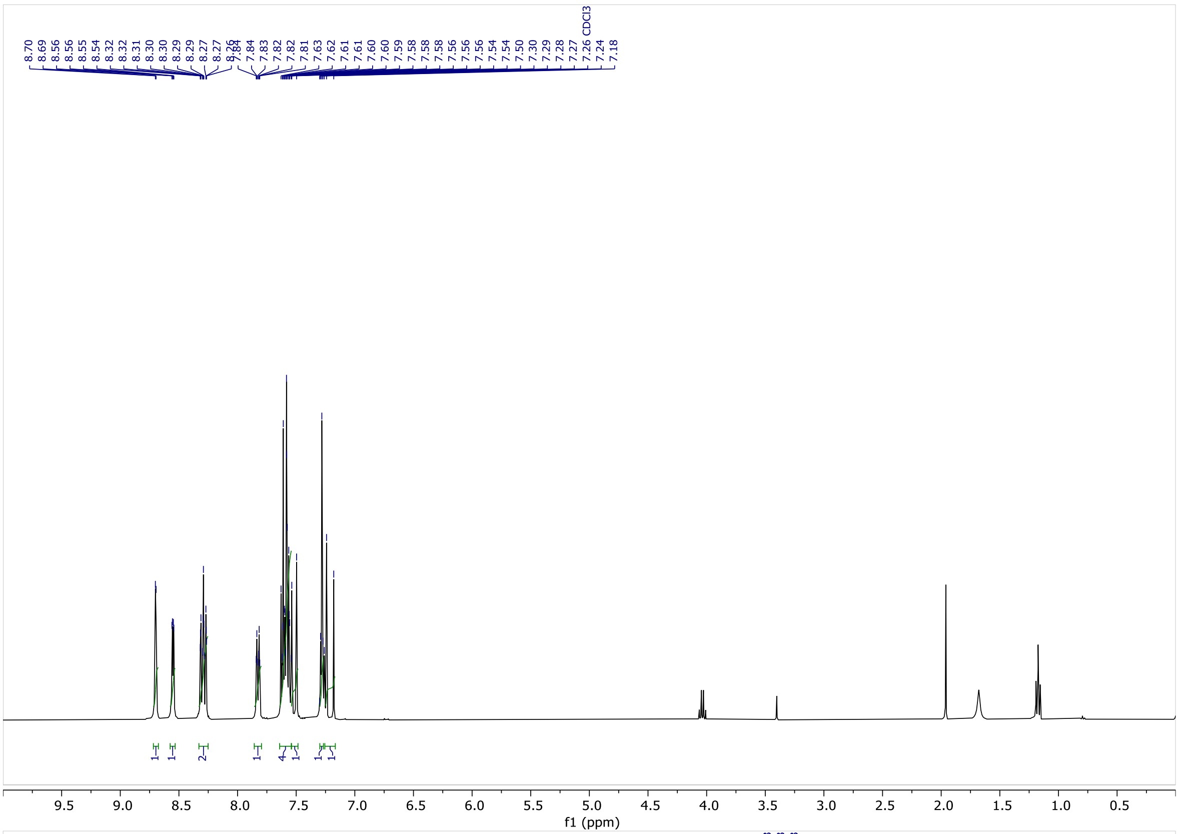

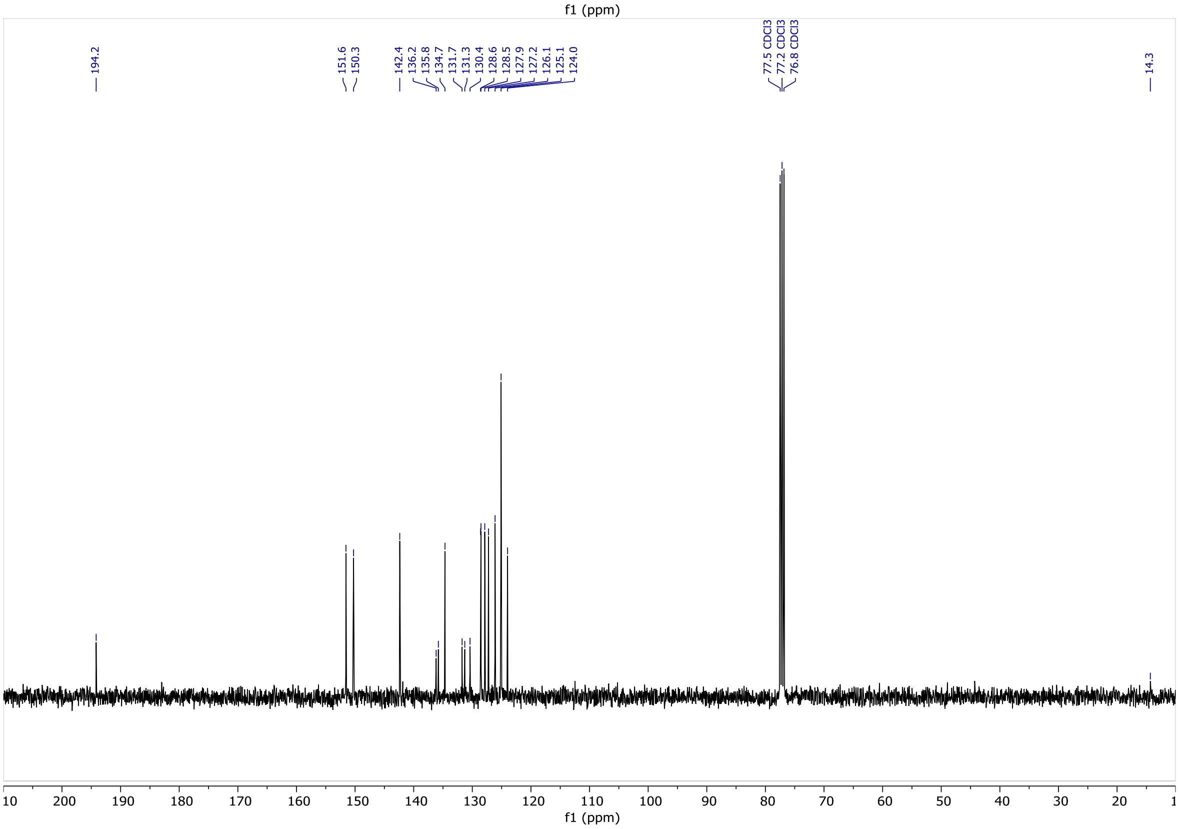

3-(3-(4-chloronaphthalen-1-yl)propyl)pyridine (64a)

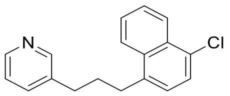

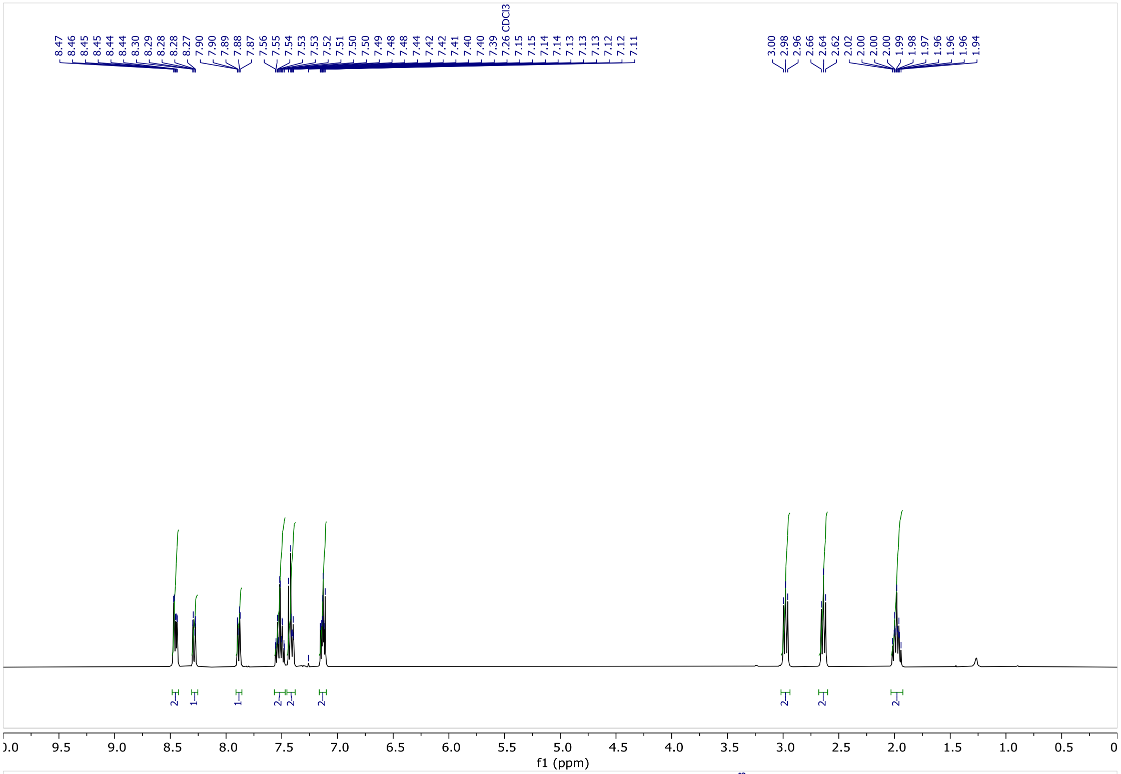

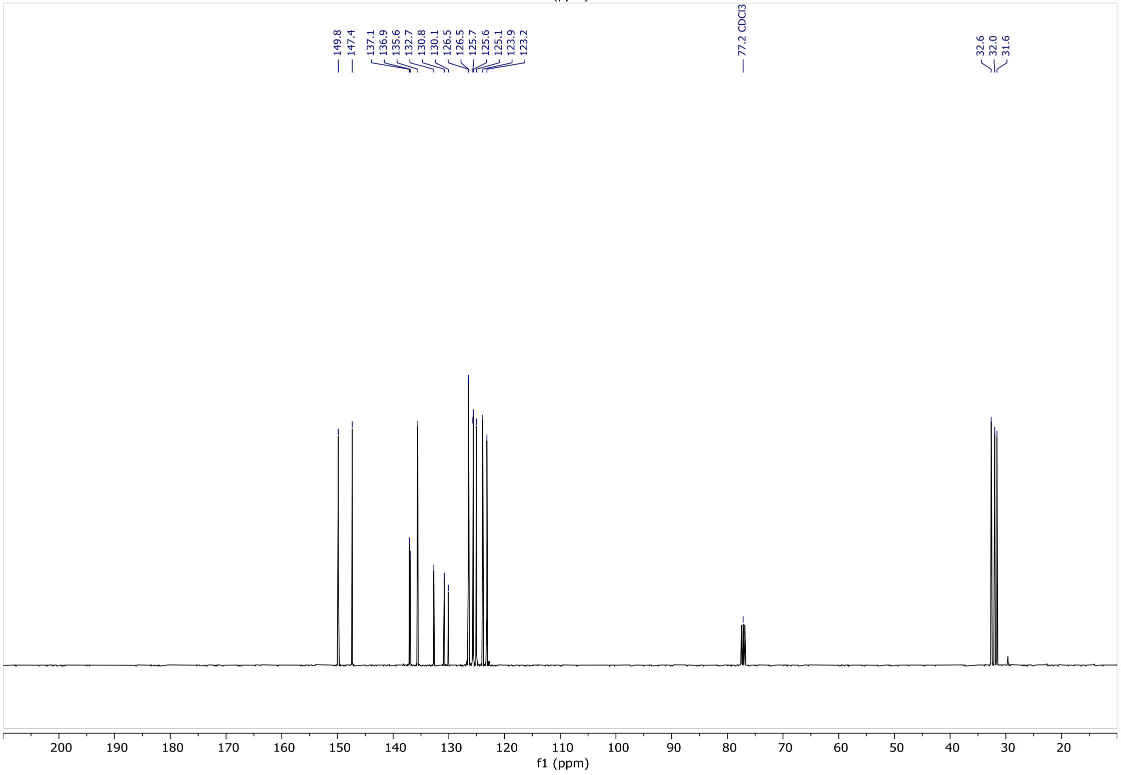

3-(3-(4-chloronaphthalen-1-yl)propyl)pyridine 1-oxide (64)

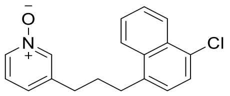

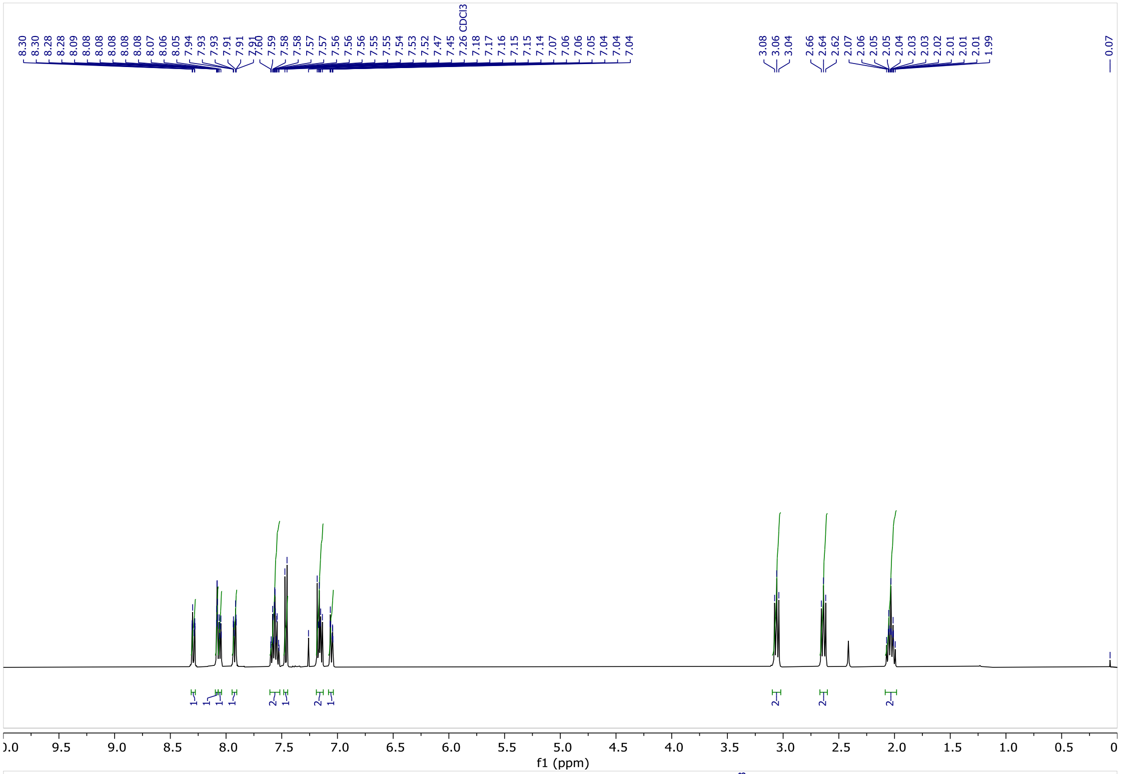

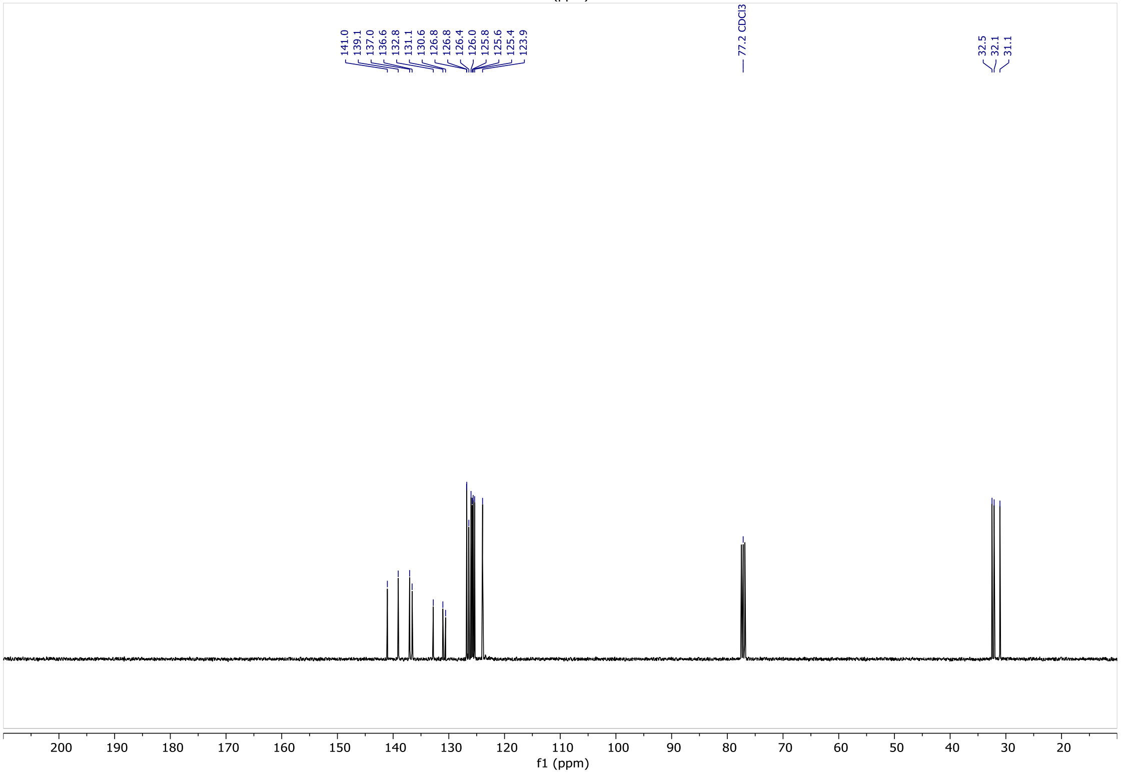

1-(4-chloronaphthalen-1-yl)ethan-1-one (65d)

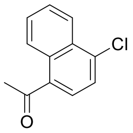

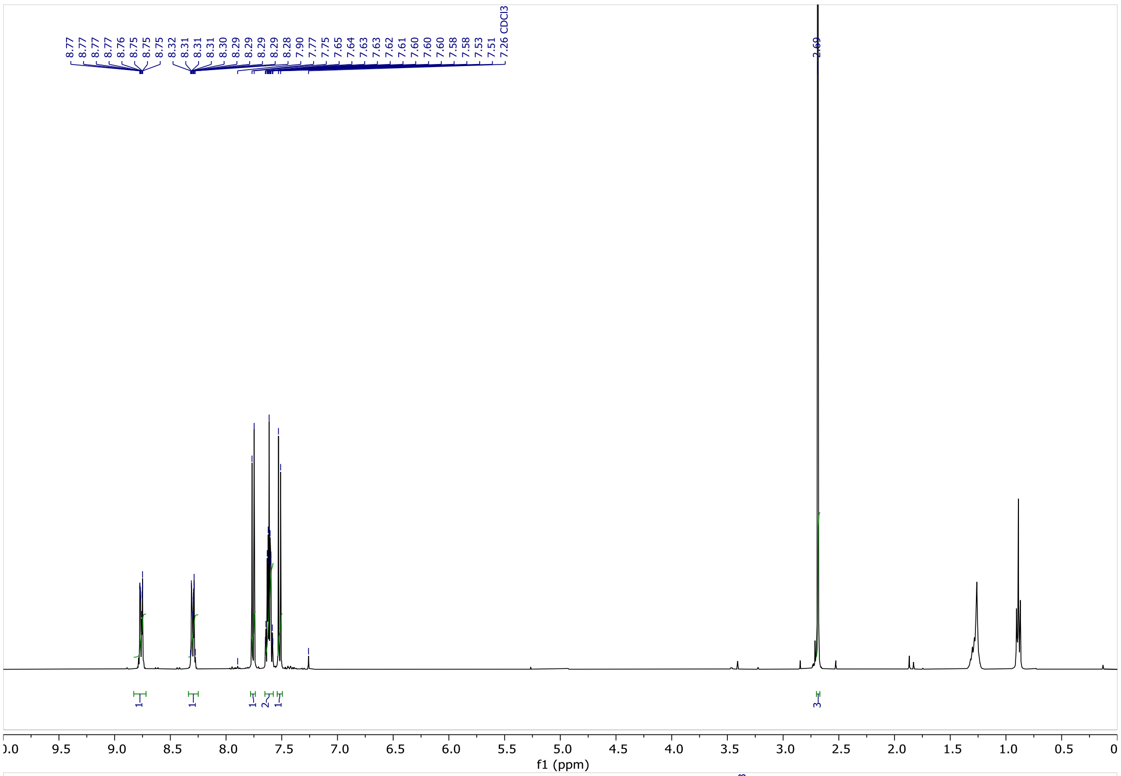

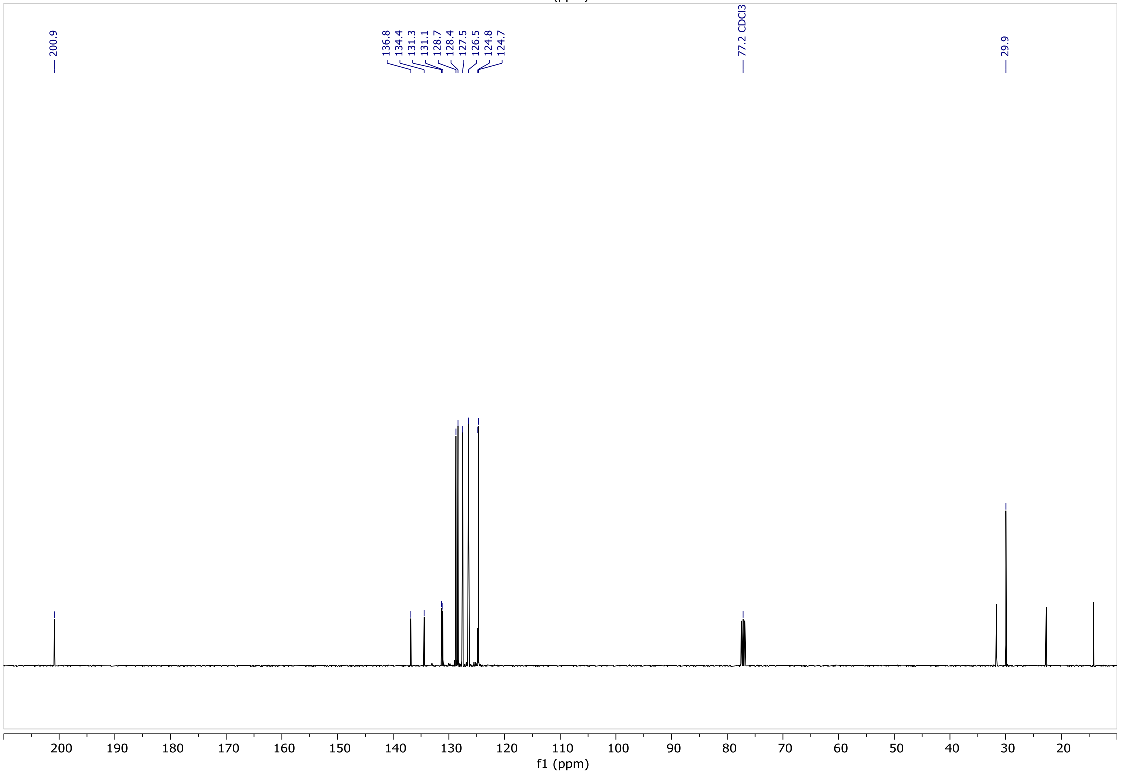

(*E*)-1-(4-chloronaphthalen-1-yl)-3-(5-methoxypyridin-3-yl)prop-2-en-1-one (65c)

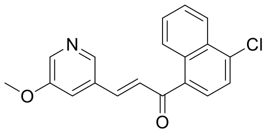

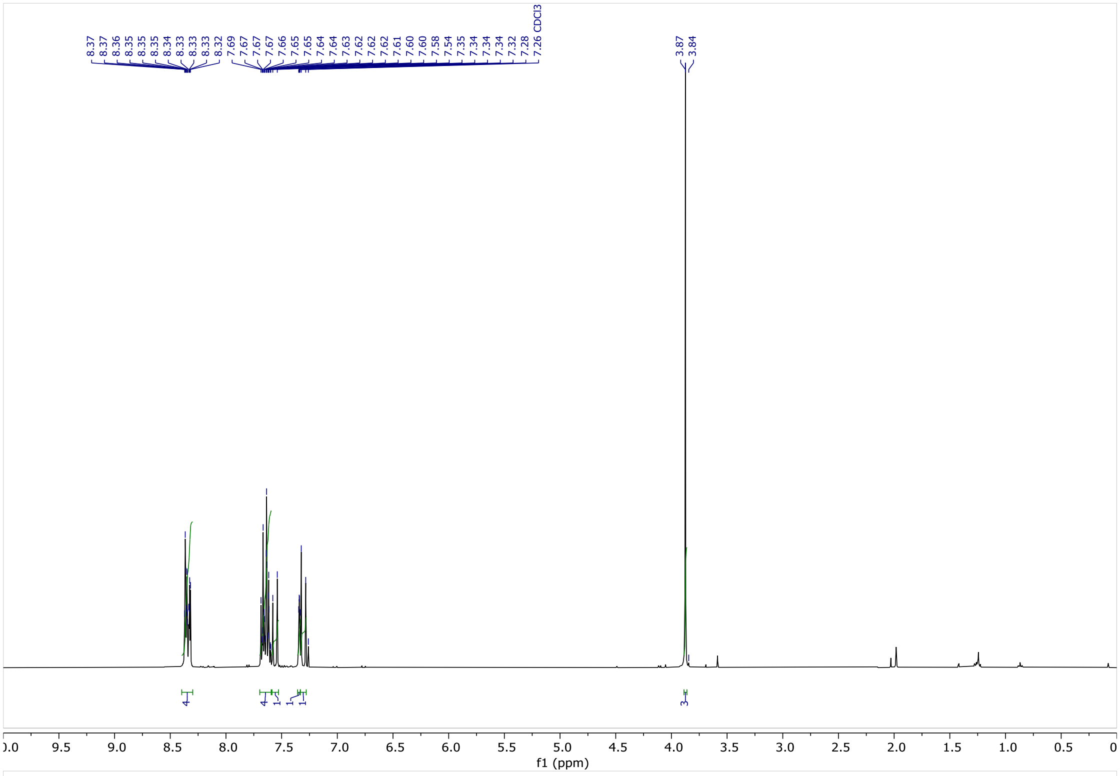

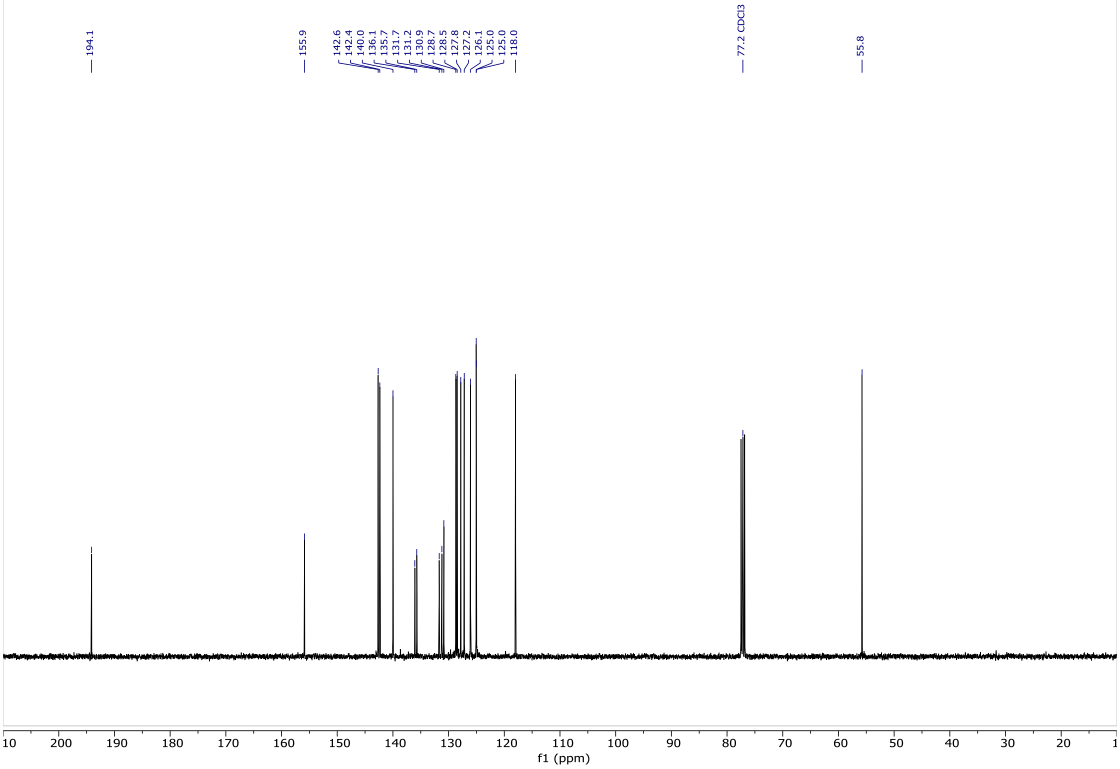

(*E*)-3-(3-(4-chloronaphthalen-1-yl)prop-1-en-1-yl)-5-methoxypyridine (65b)

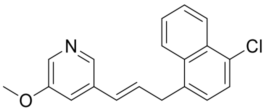

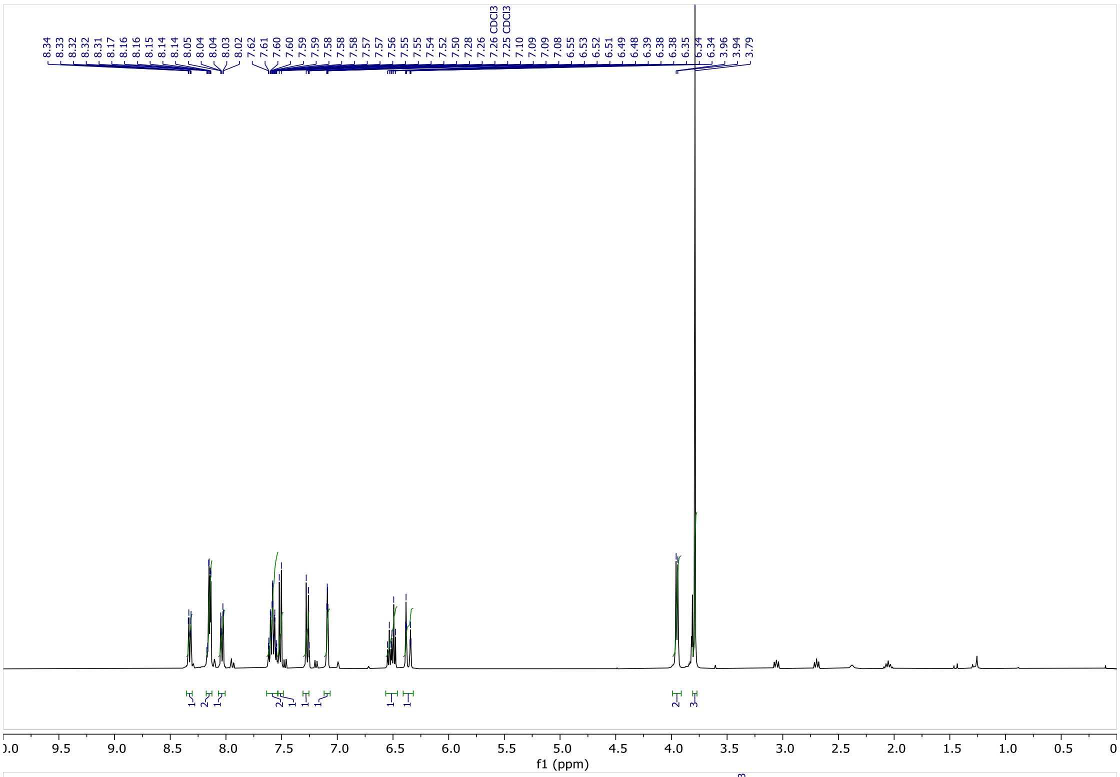

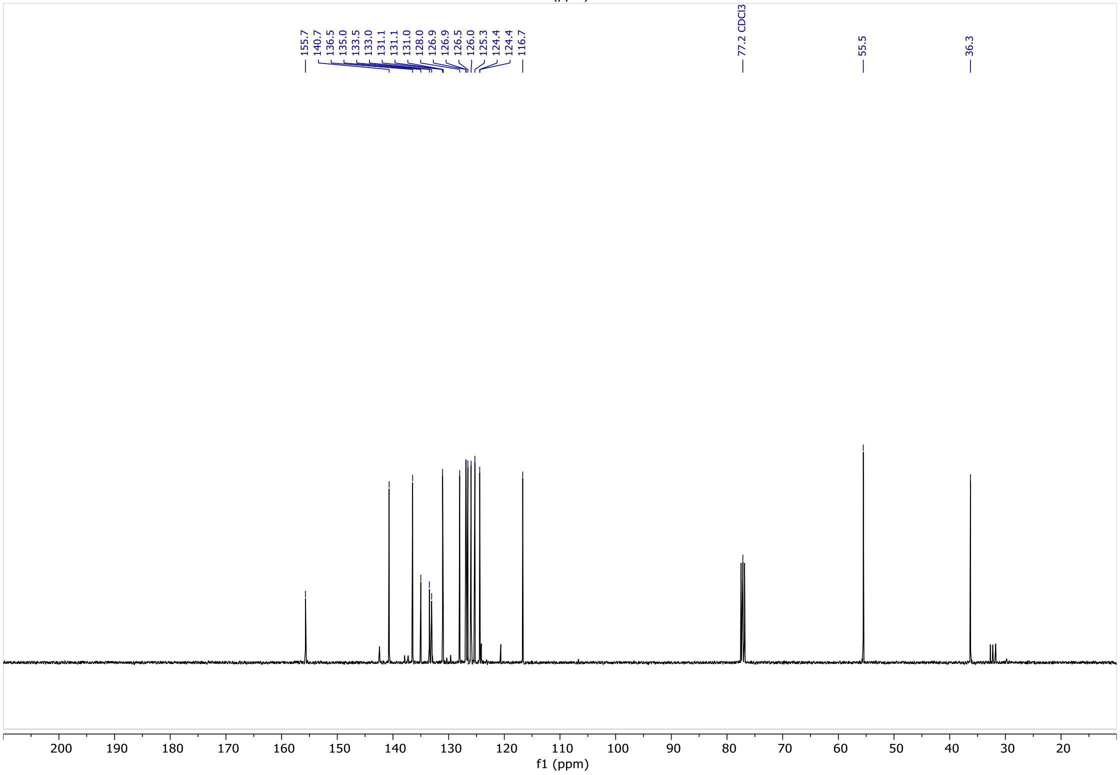

3-(3-(4-chloronaphthalen-1-yl)propyl)-5-methoxypyridine (65a)

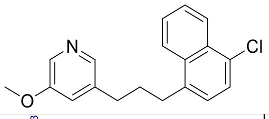

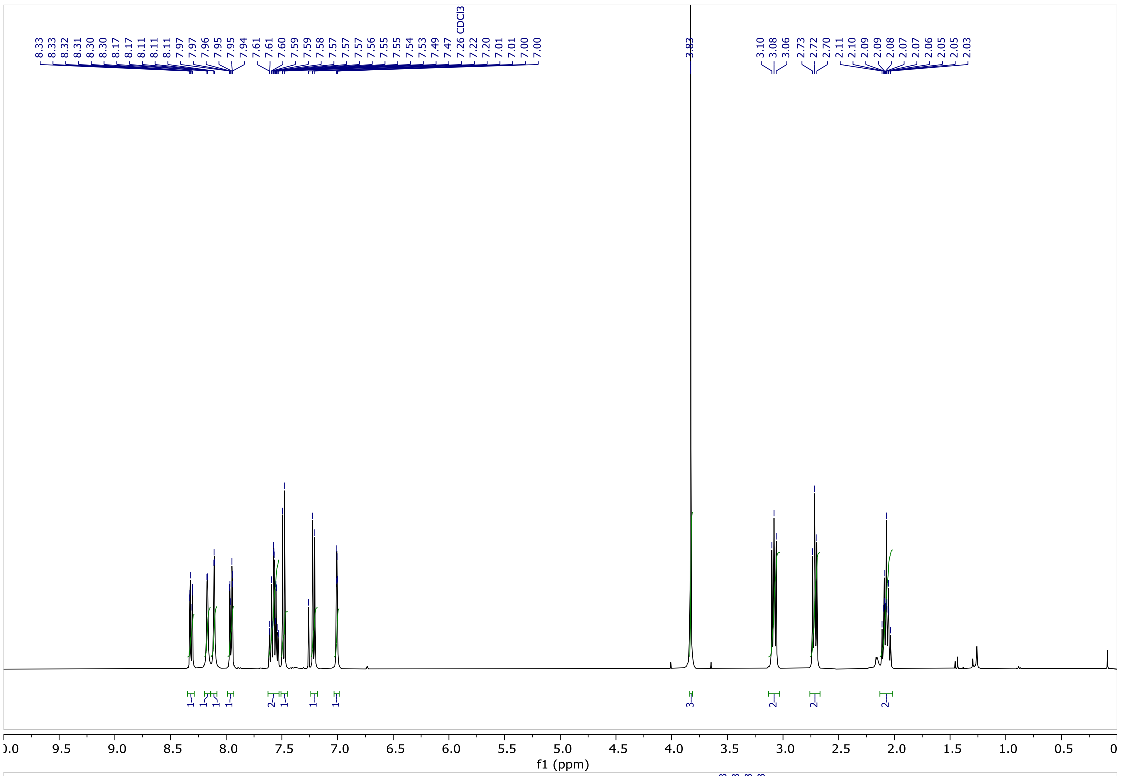

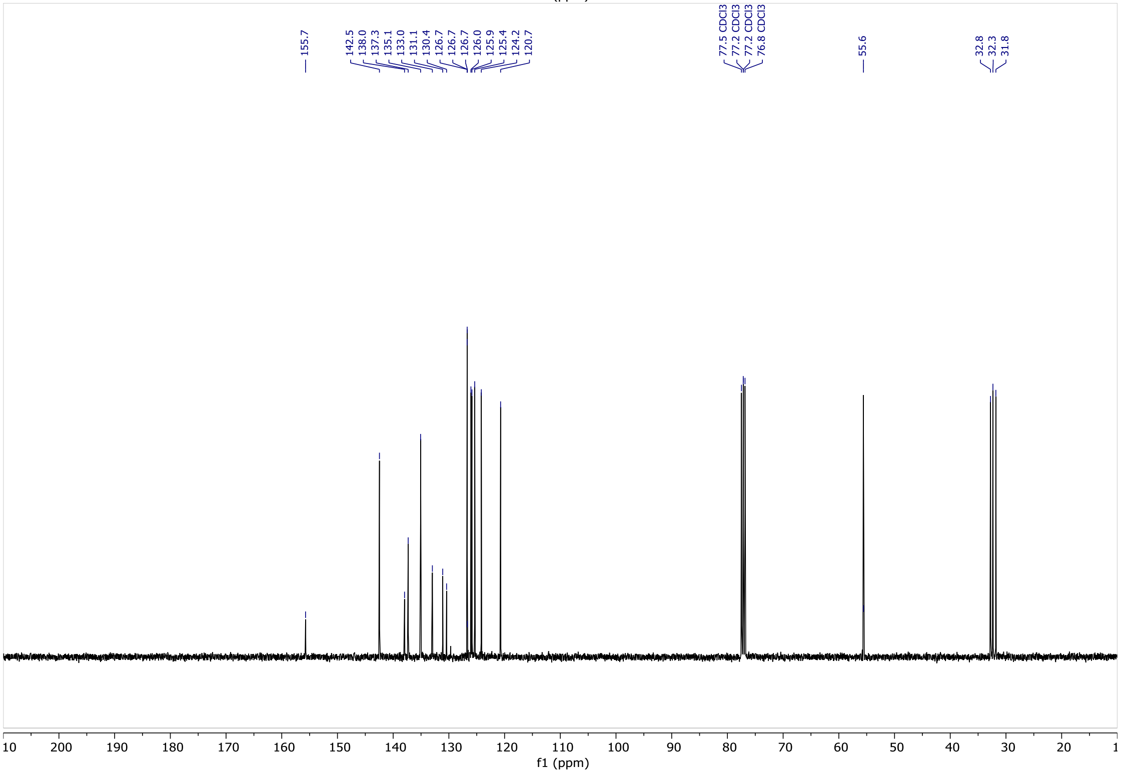

3-(3-(4-chloronaphthalen-1-yl)propyl)-5-methoxypyridine 1-oxide (65)

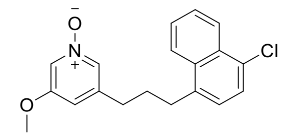

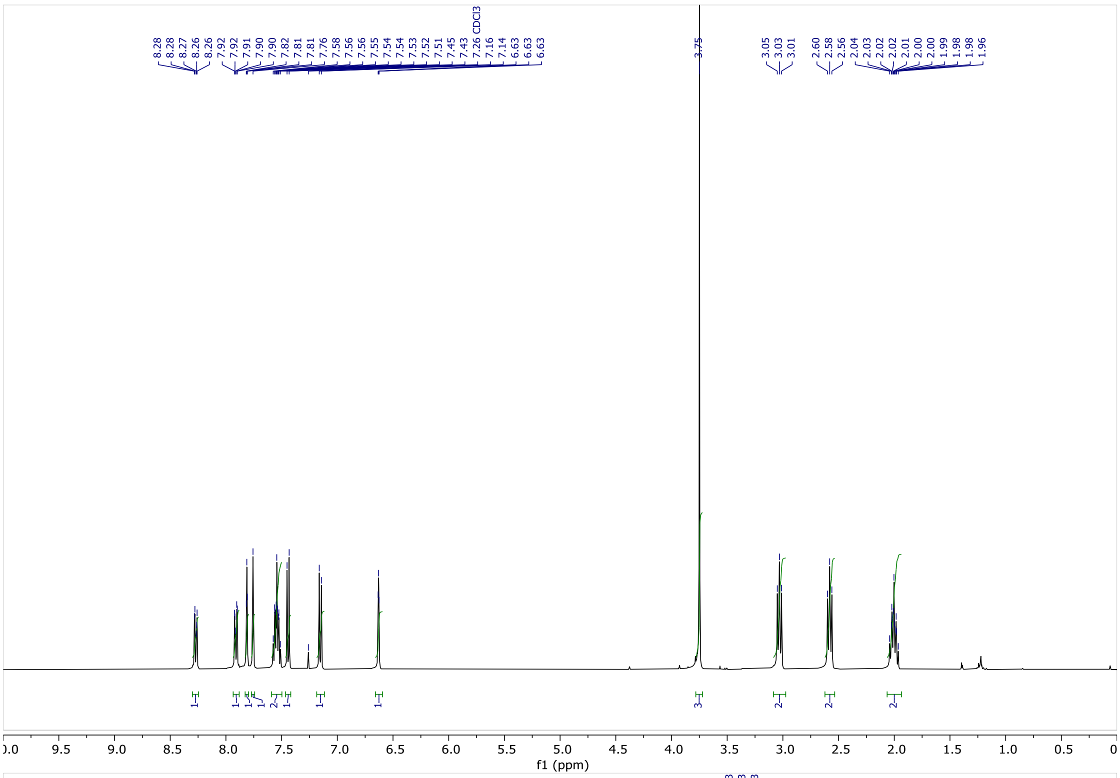

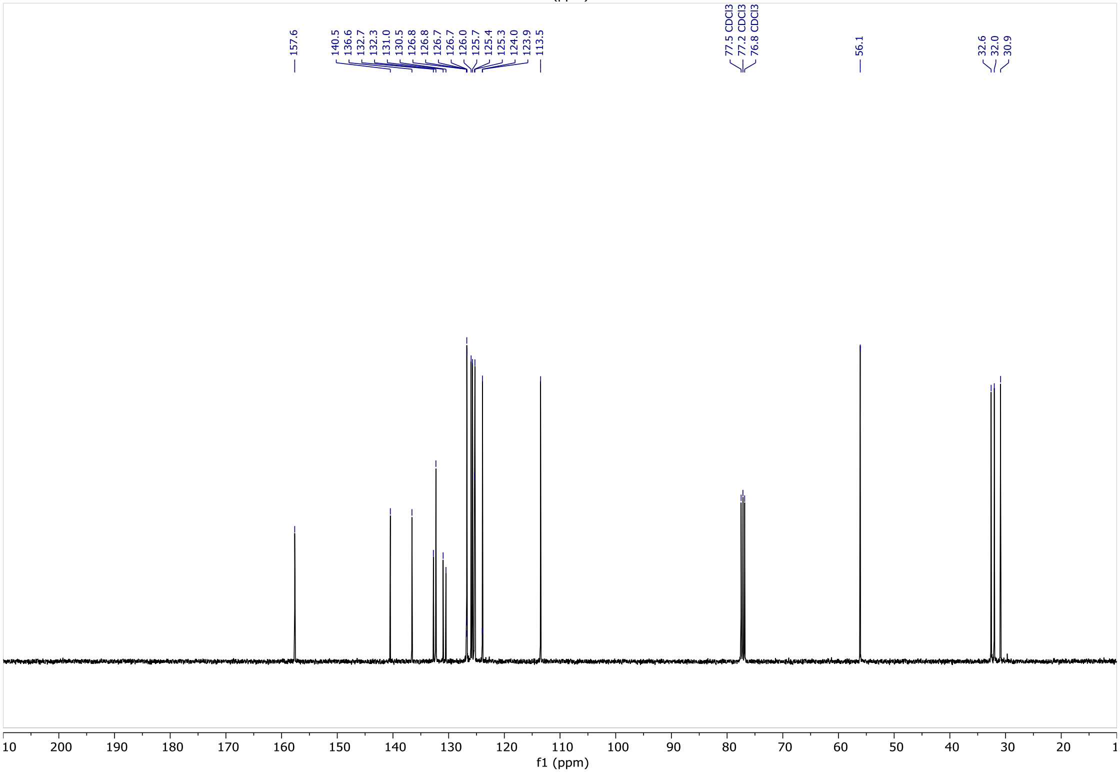

5-(3-(4-chloronaphthalen-1-yl)propyl)pyridin-3-ol (66a)

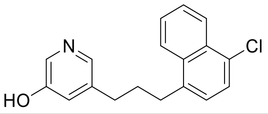

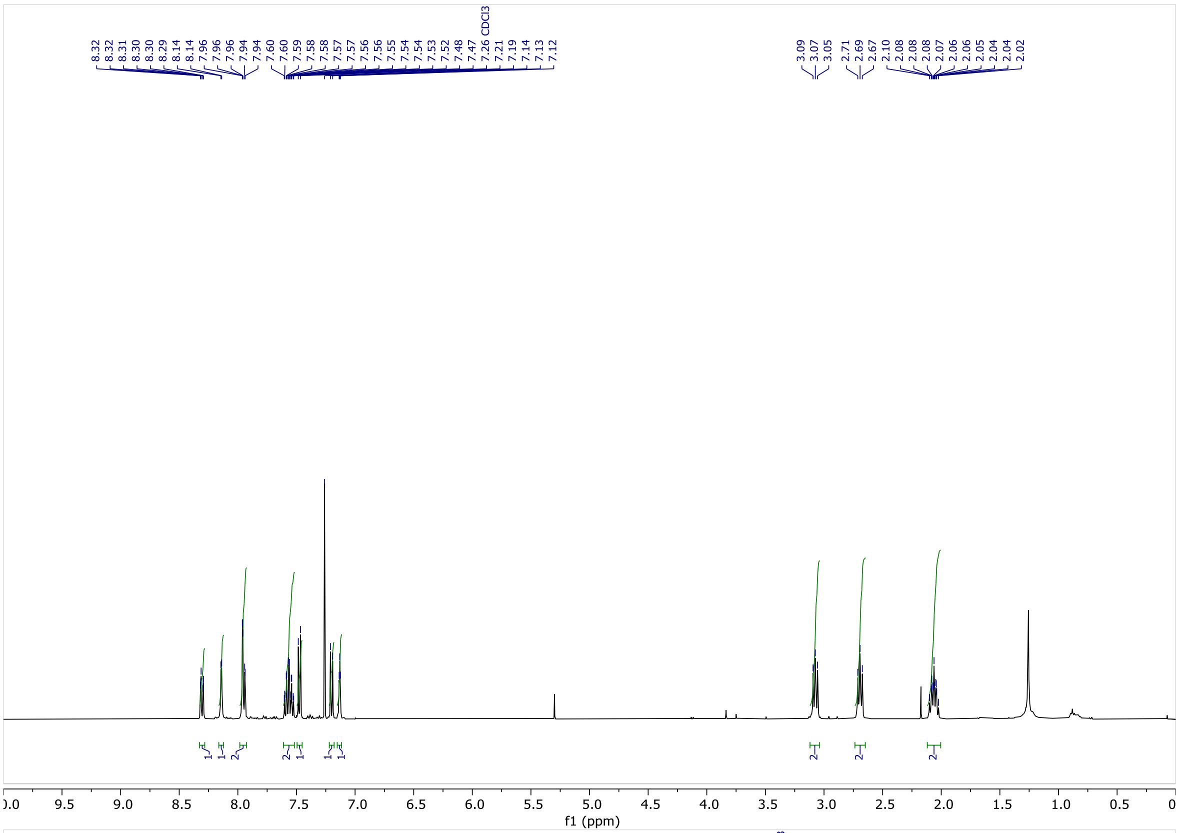

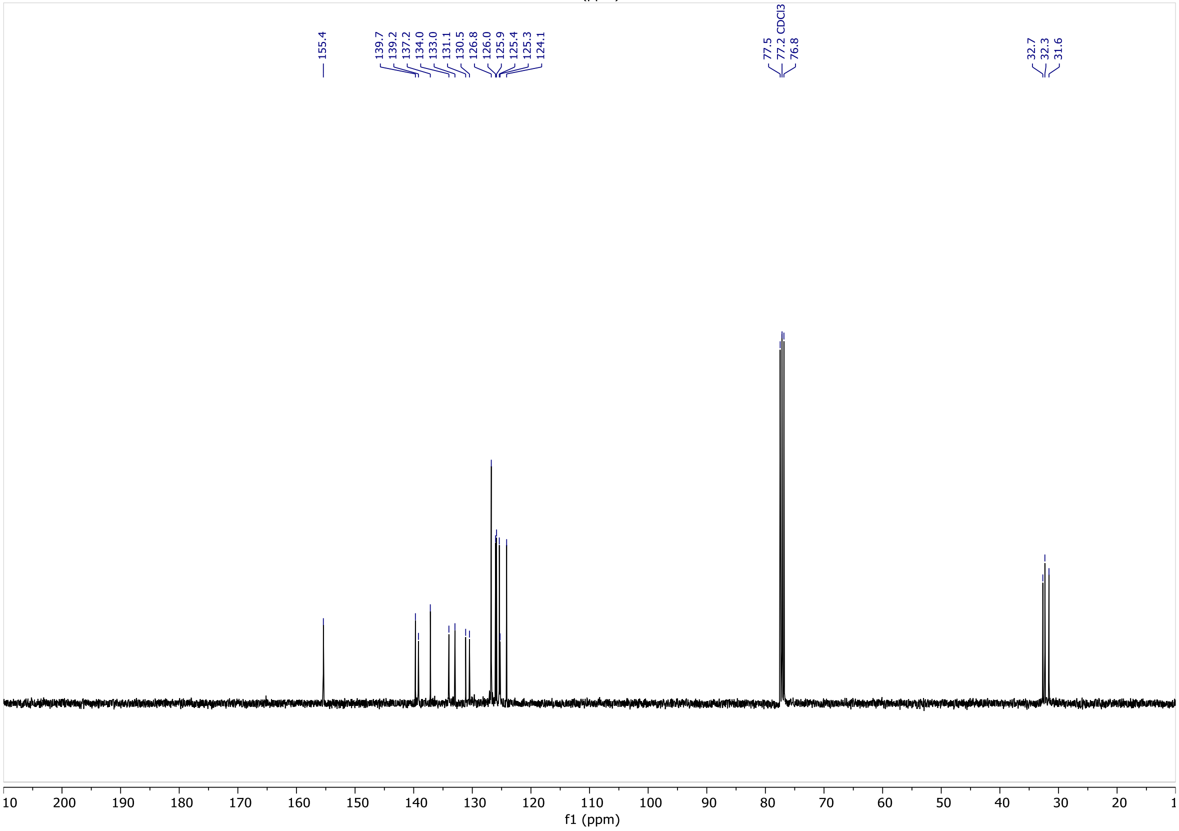

3-(3-(4-chloronaphthalen-1-yl)propyl)-5-hydroxypyridine 1-oxide (66)

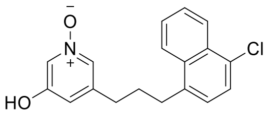

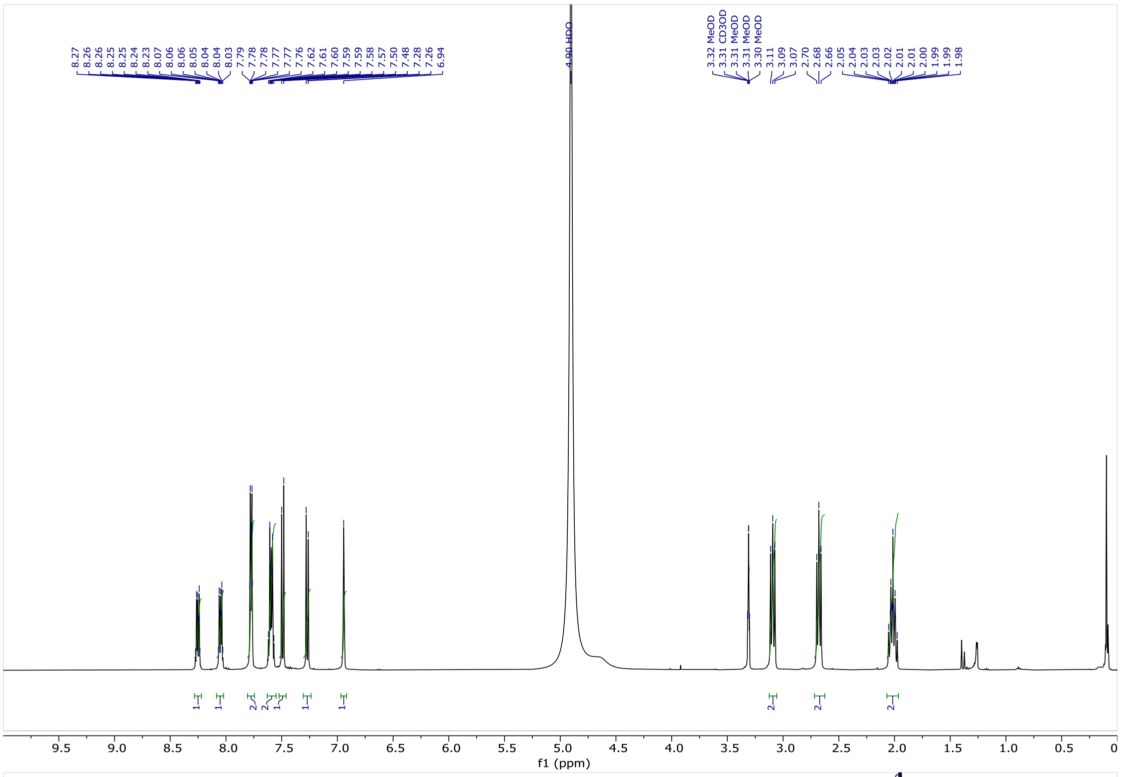

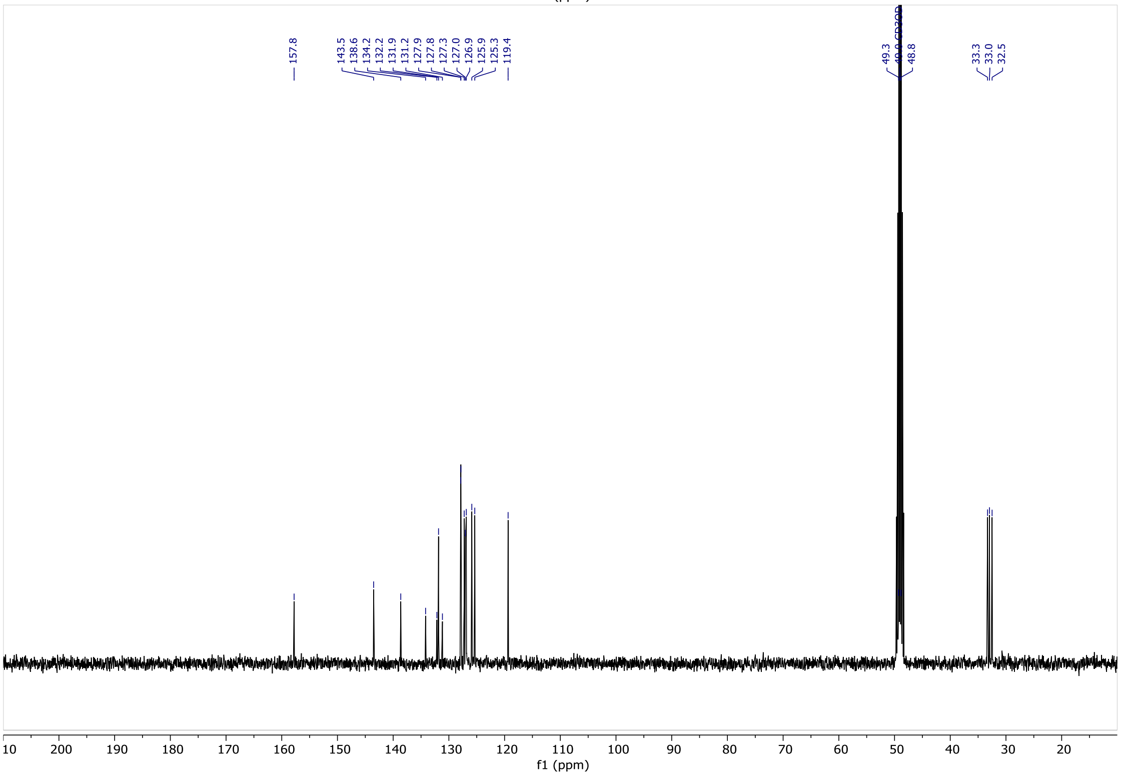

3-(2-((4-chloronaphthalen-1-yl)oxy)ethyl)-5-fluoropyridine (67a)

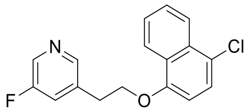

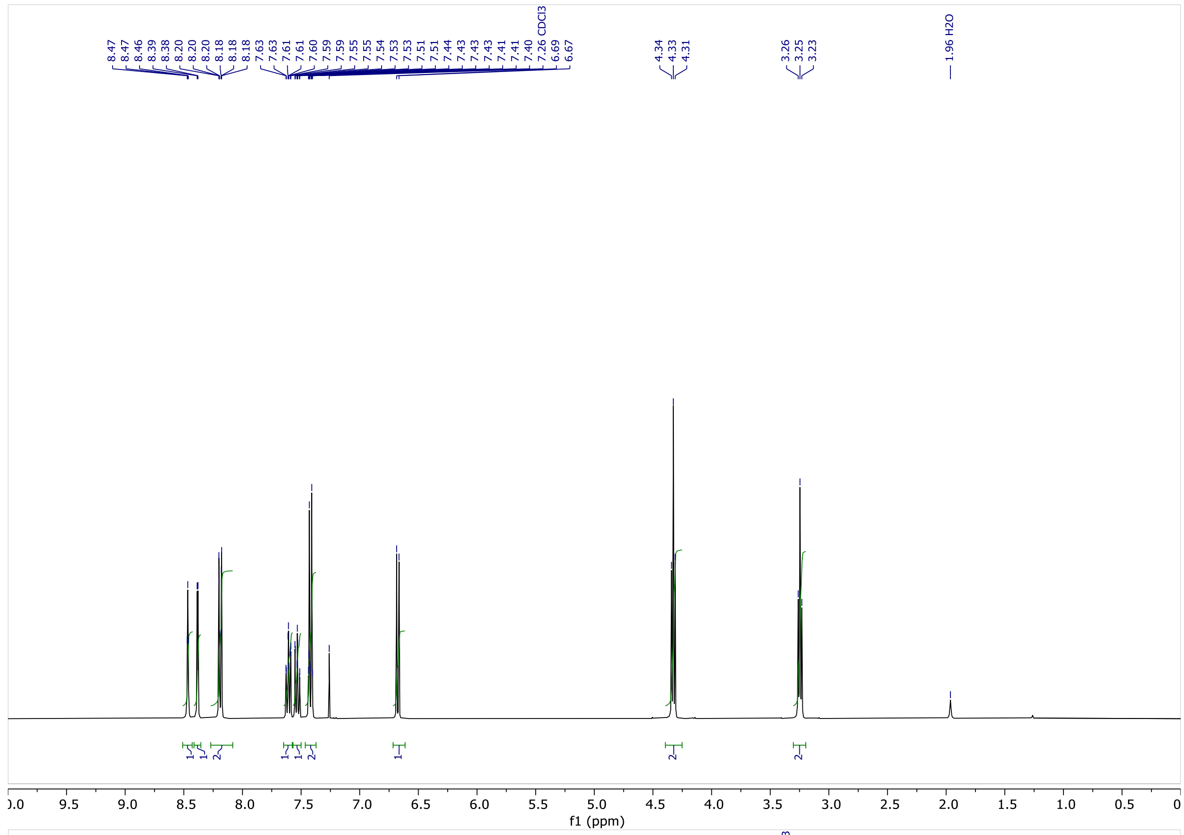

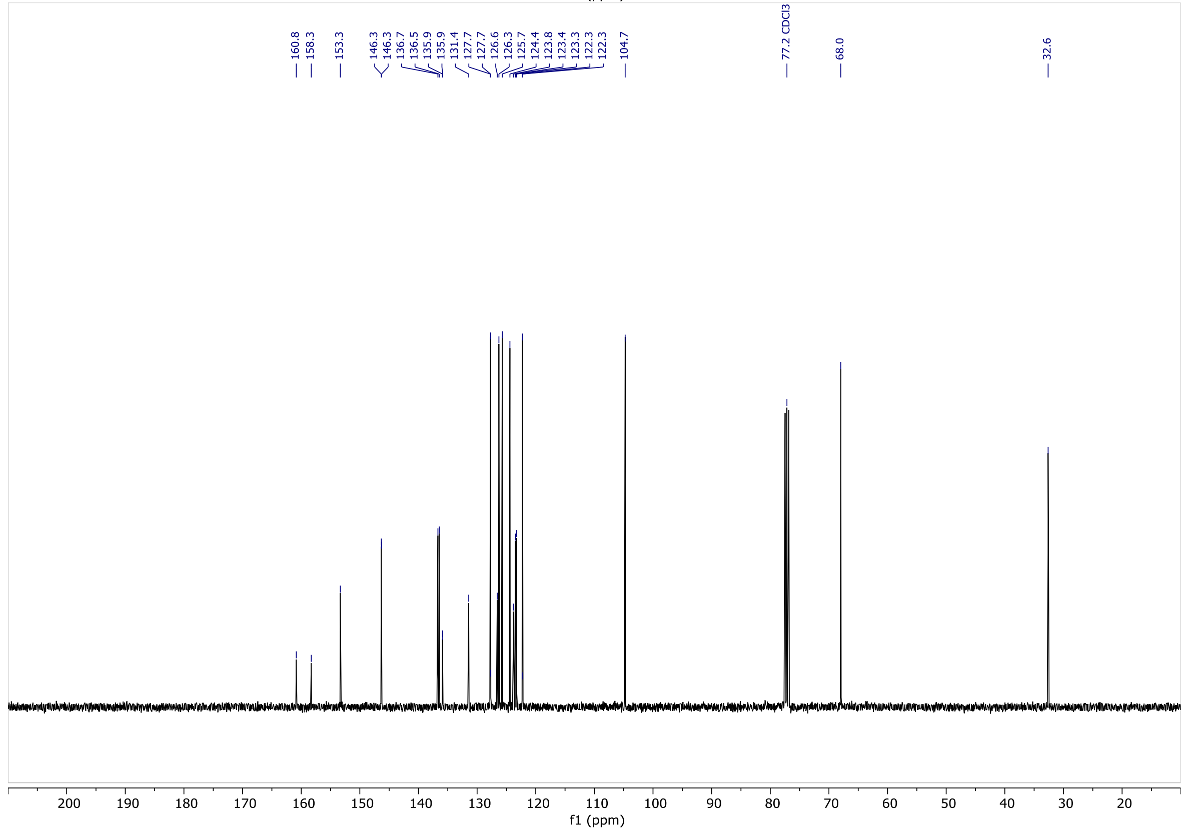

3-(2-((4-chloronaphthalen-1-yl)oxy)ethyl)-5-fluoropyridine 1-oxide (67)

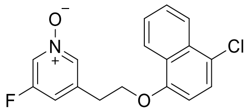

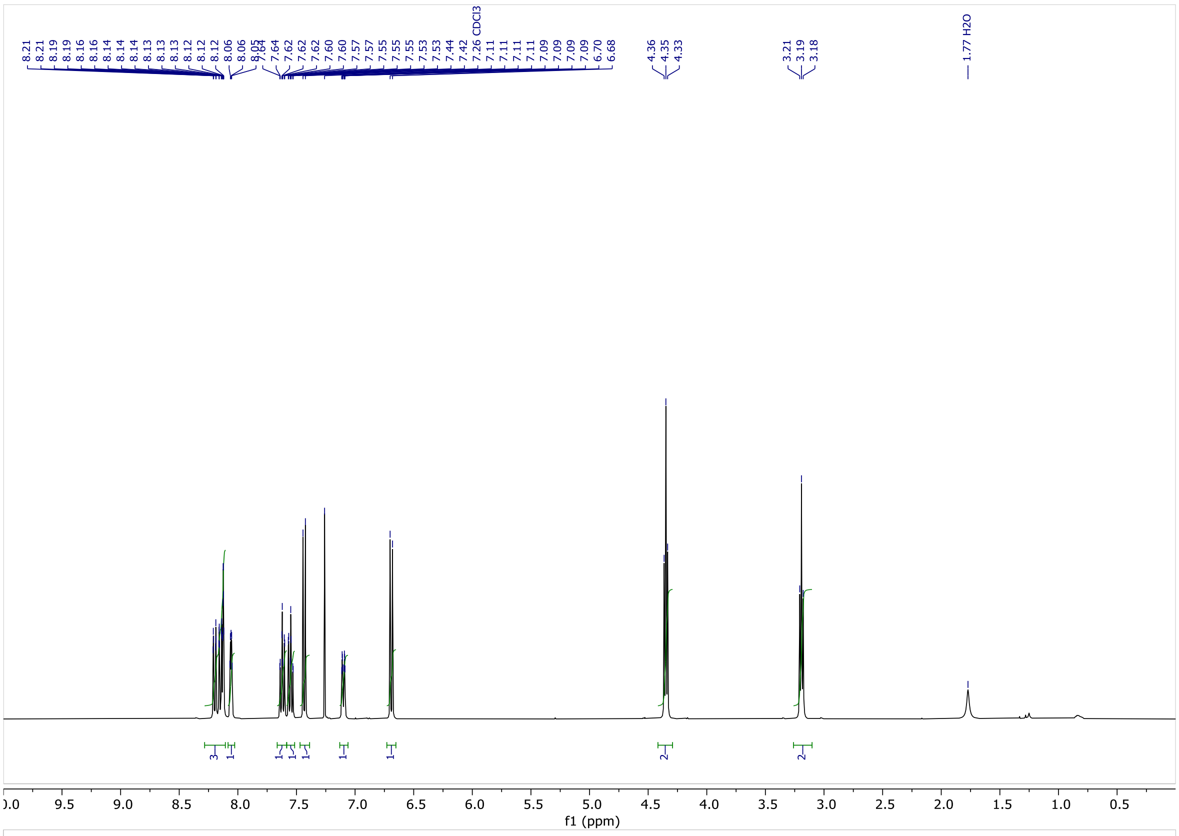

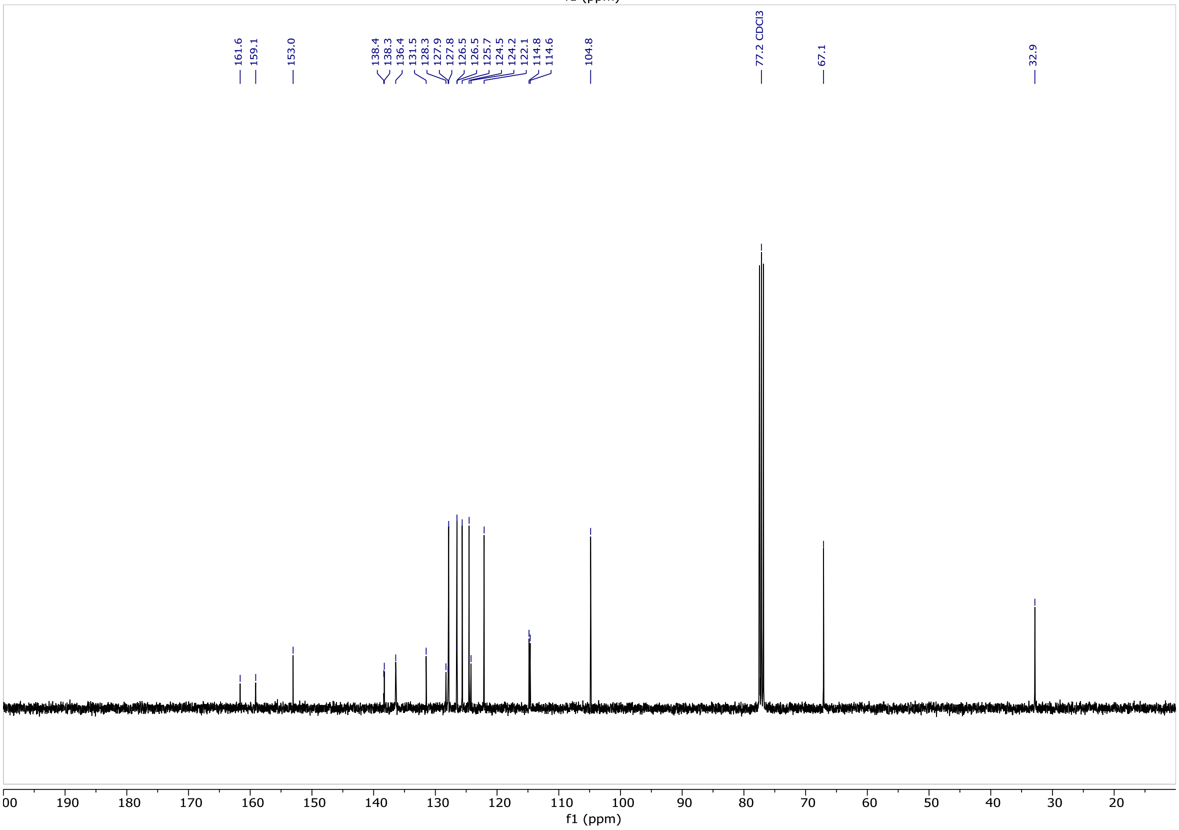

(*E*)-1-(3,5-bis(trifluoromethyl)phenyl)-3-(5-methoxypyridin-3-yl)prop-2-en-1-one (68d)

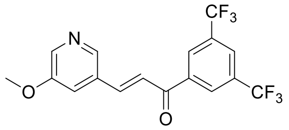

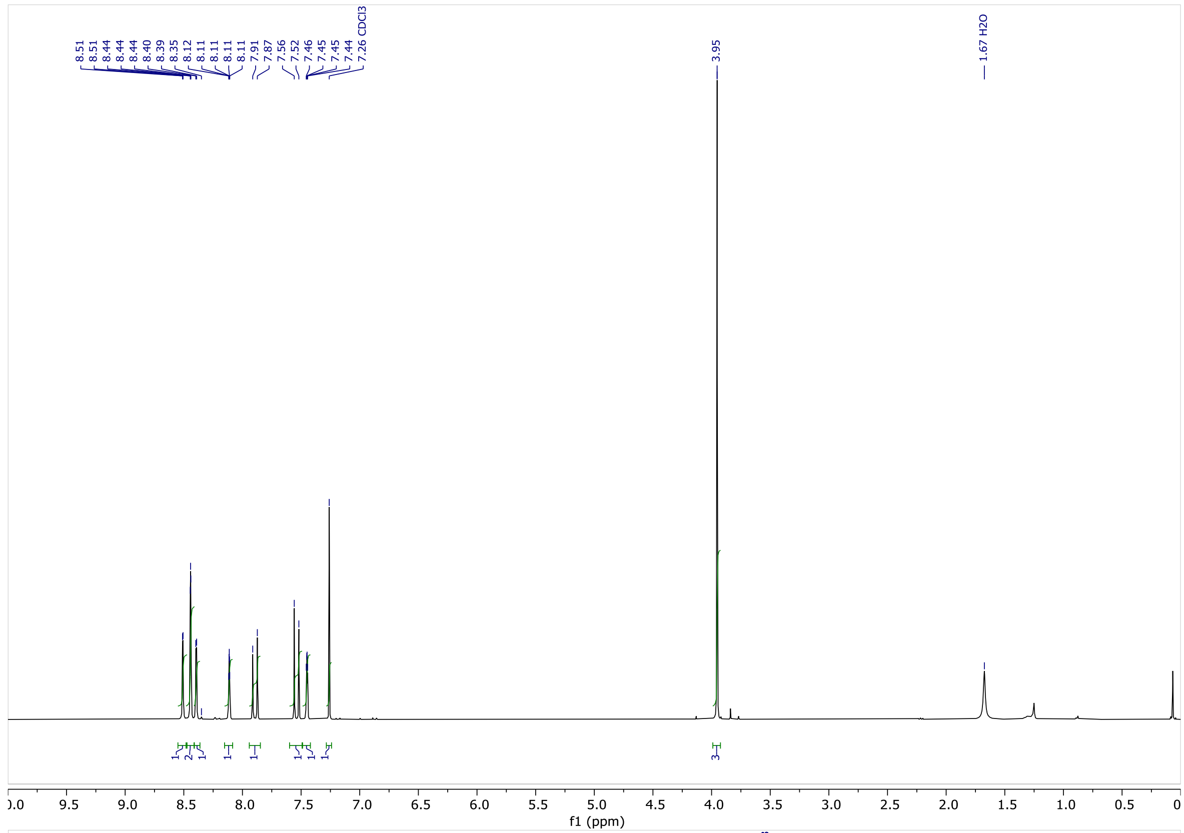

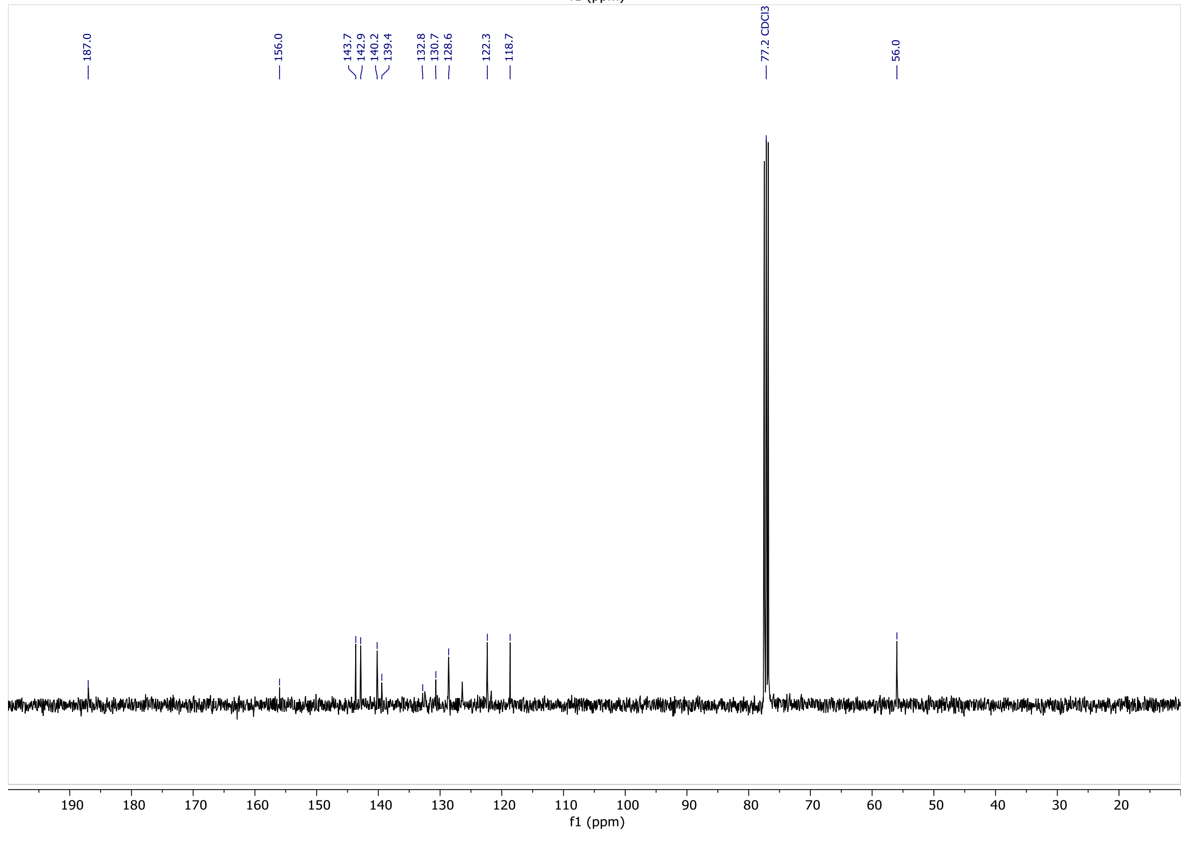

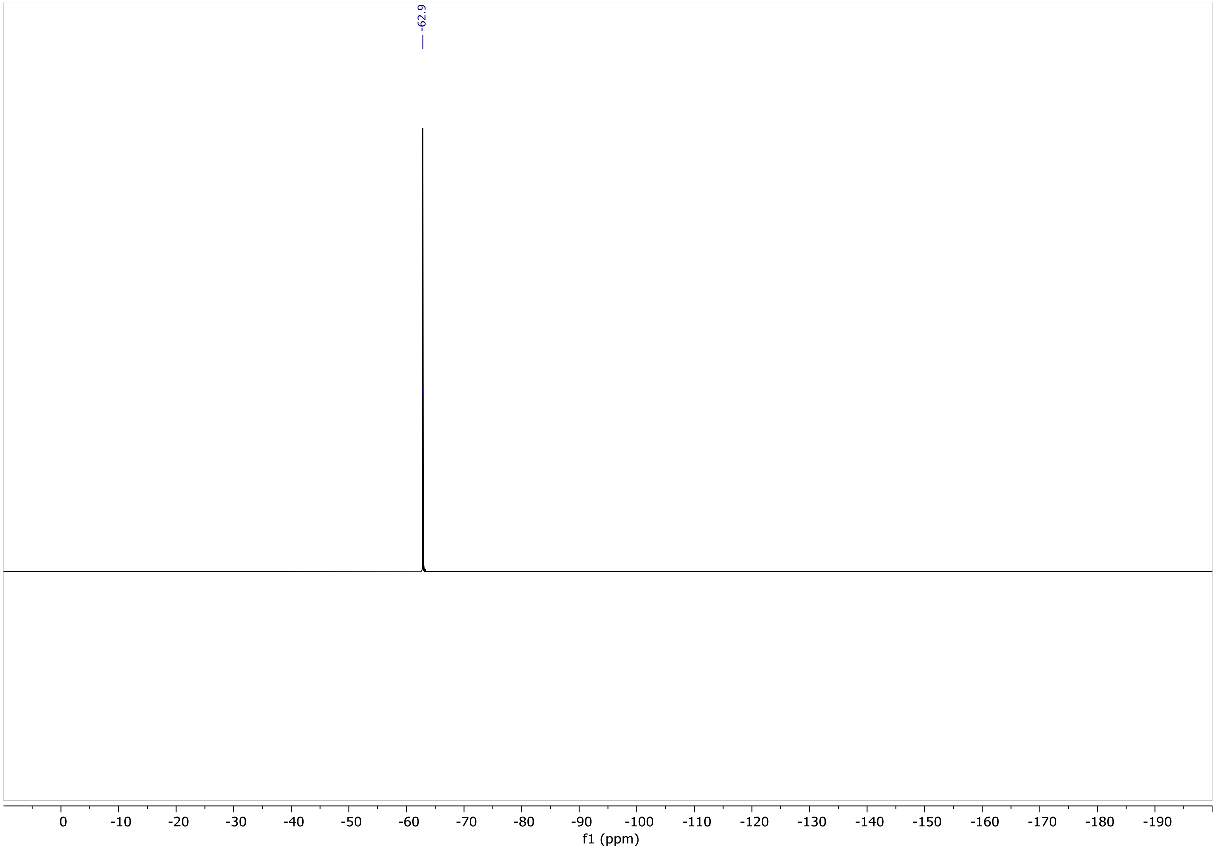
-62.9
0
-10
-20
-30
-40
-50
-60
-70
-80
-90
-100
-110
-120
-130
-140
-150
-160
-170
-180
-190
f1 (ppm)

(*E*)-3-(3-(3,5-bis(trifluoromethyl)phenyl)prop-1-en-1-yl)-5-methoxypyridine (68c)

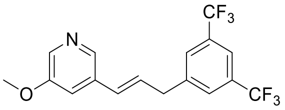

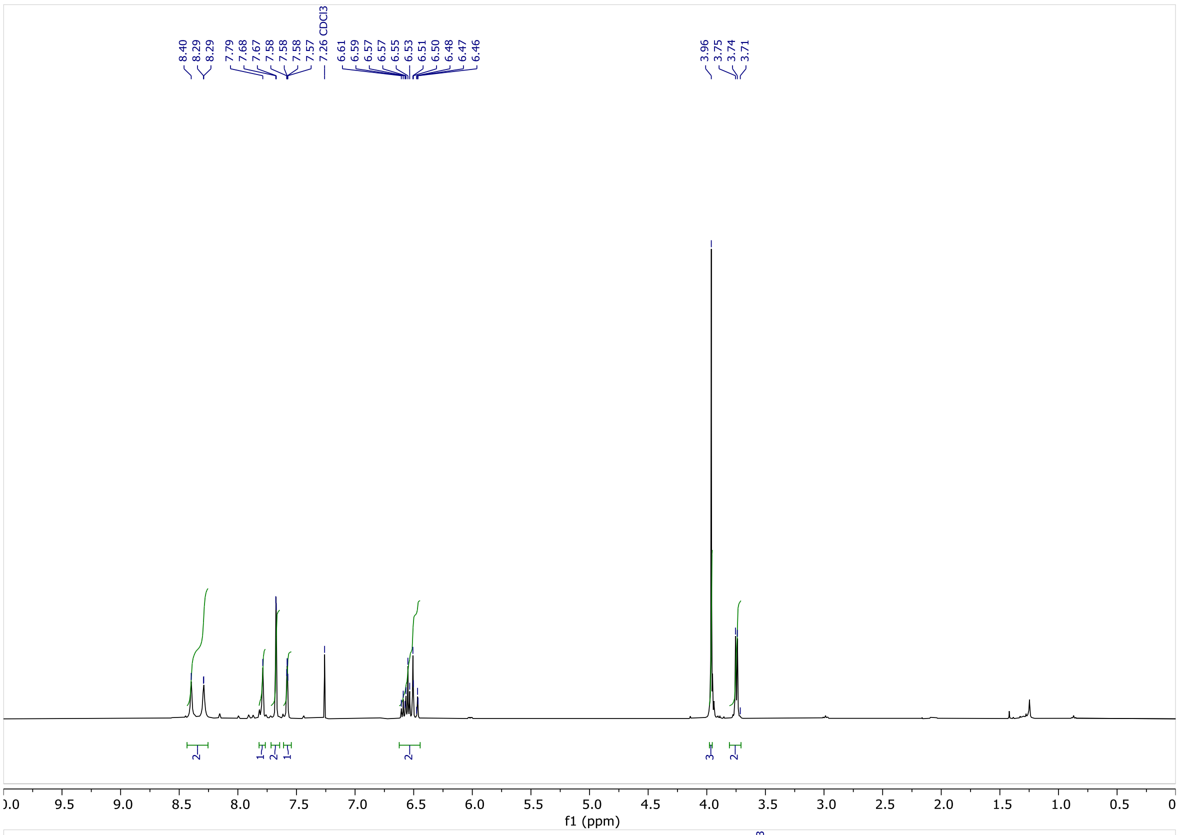

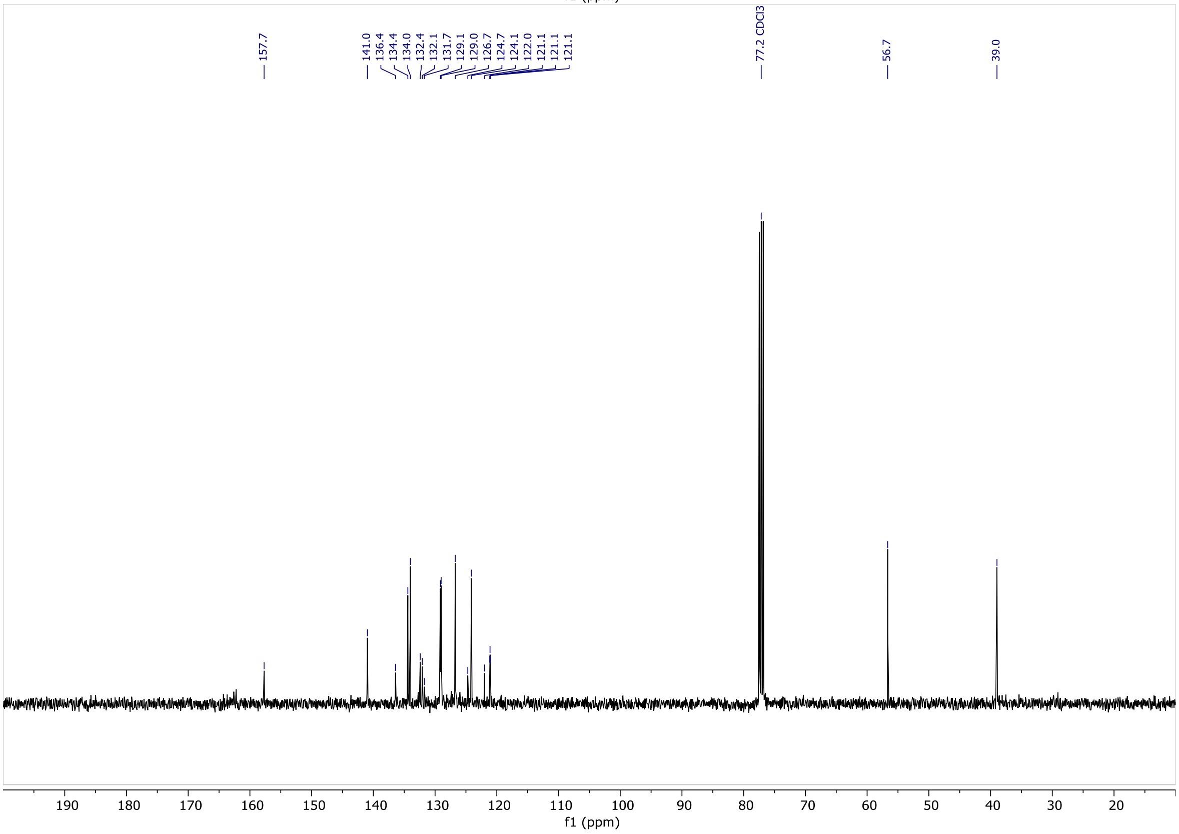

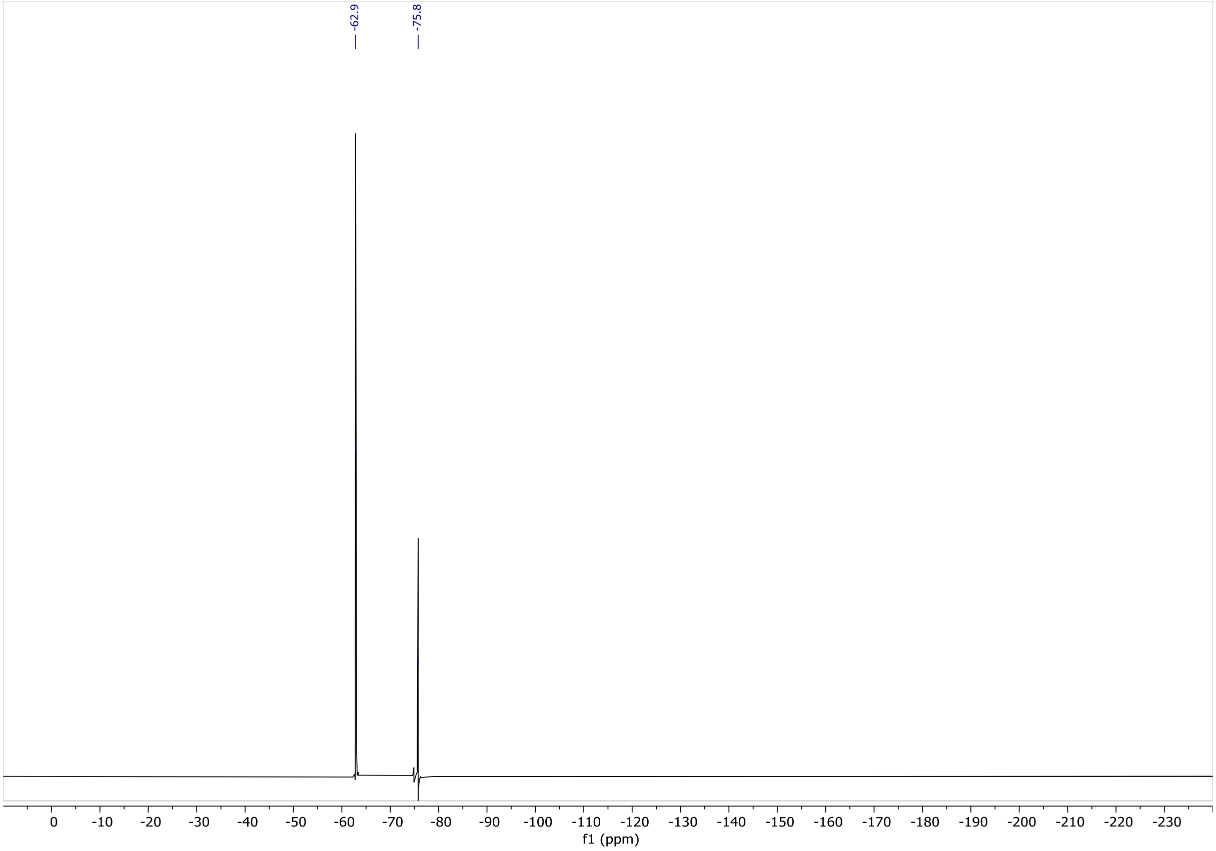
-62.9
-75.8
0 -10 -20 -30 -40 -50 -60 -70 -80 -90 -100 -110 -120 -130 -140 -150 -160 -170 -180 -190 -200 -210 -220 -230
f1 (ppm)

3-(3-(3,5-bis(trifluoromethyl)phenyl)propyl)-5-methoxypyridine (68b)

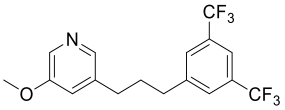

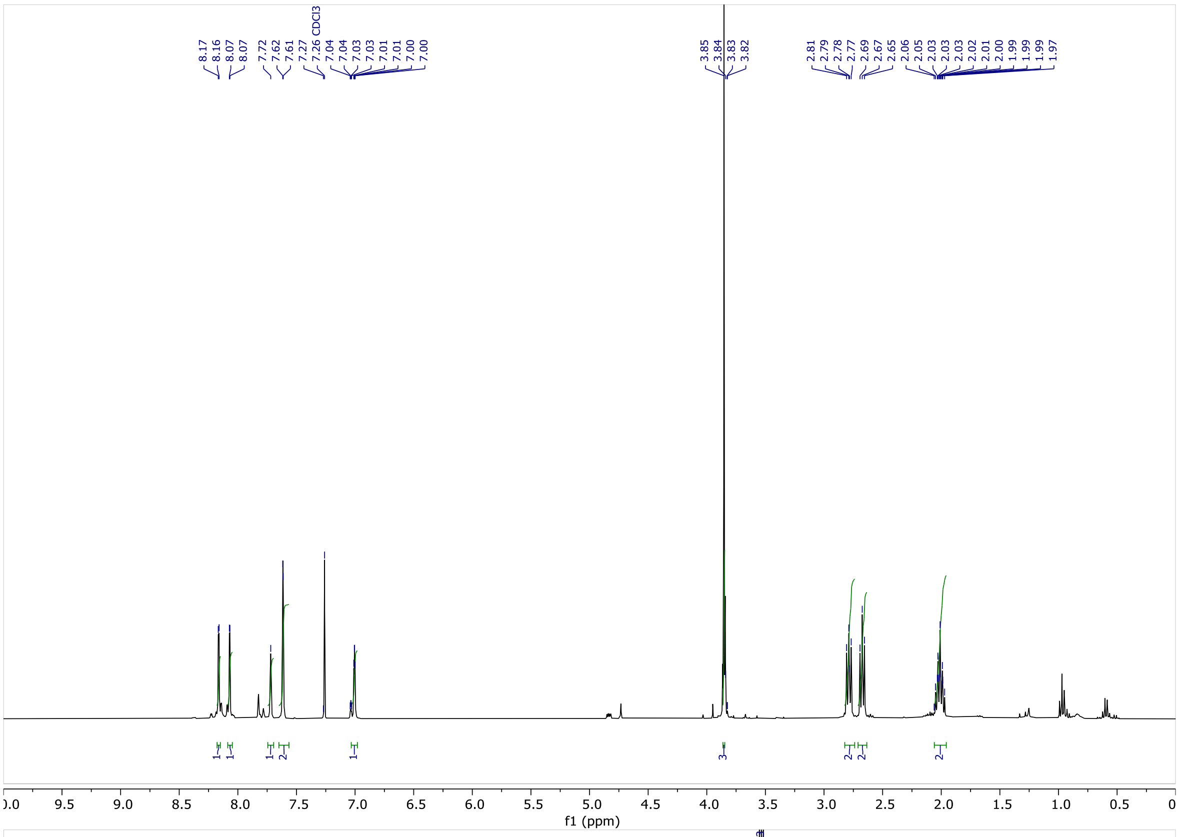

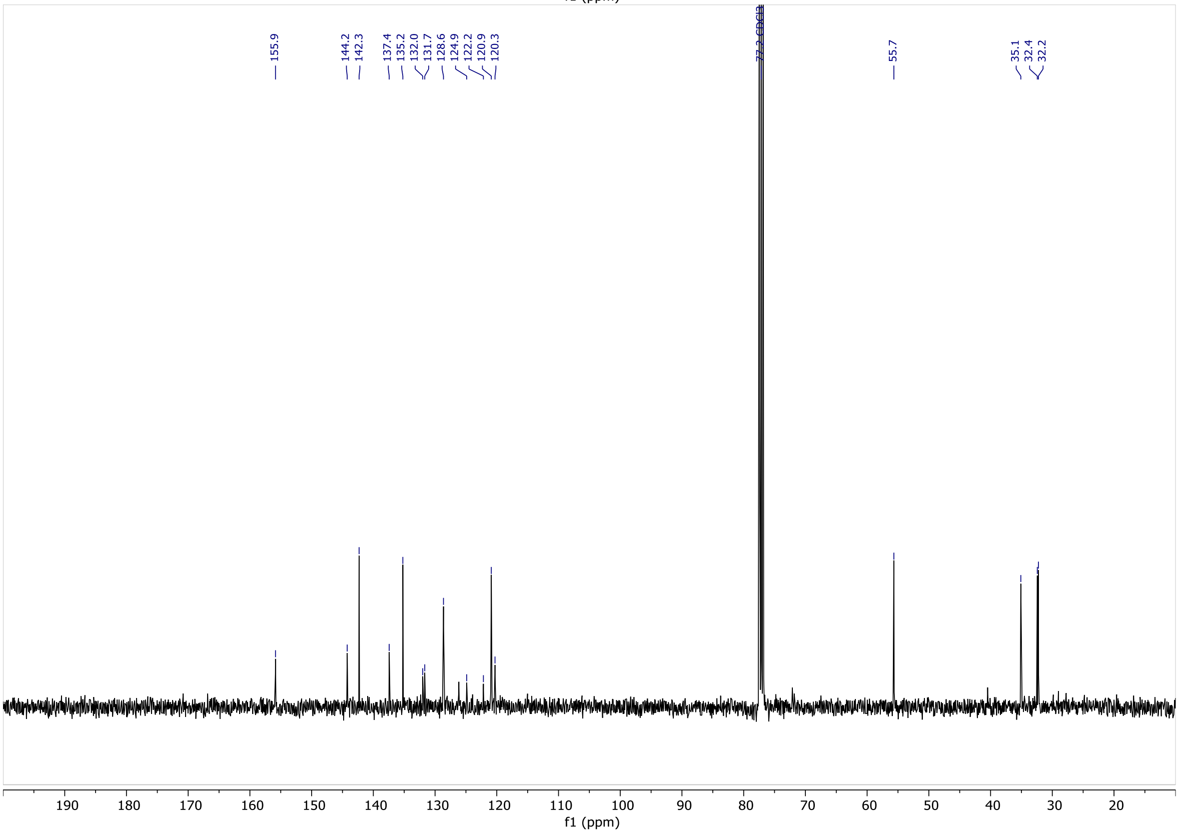

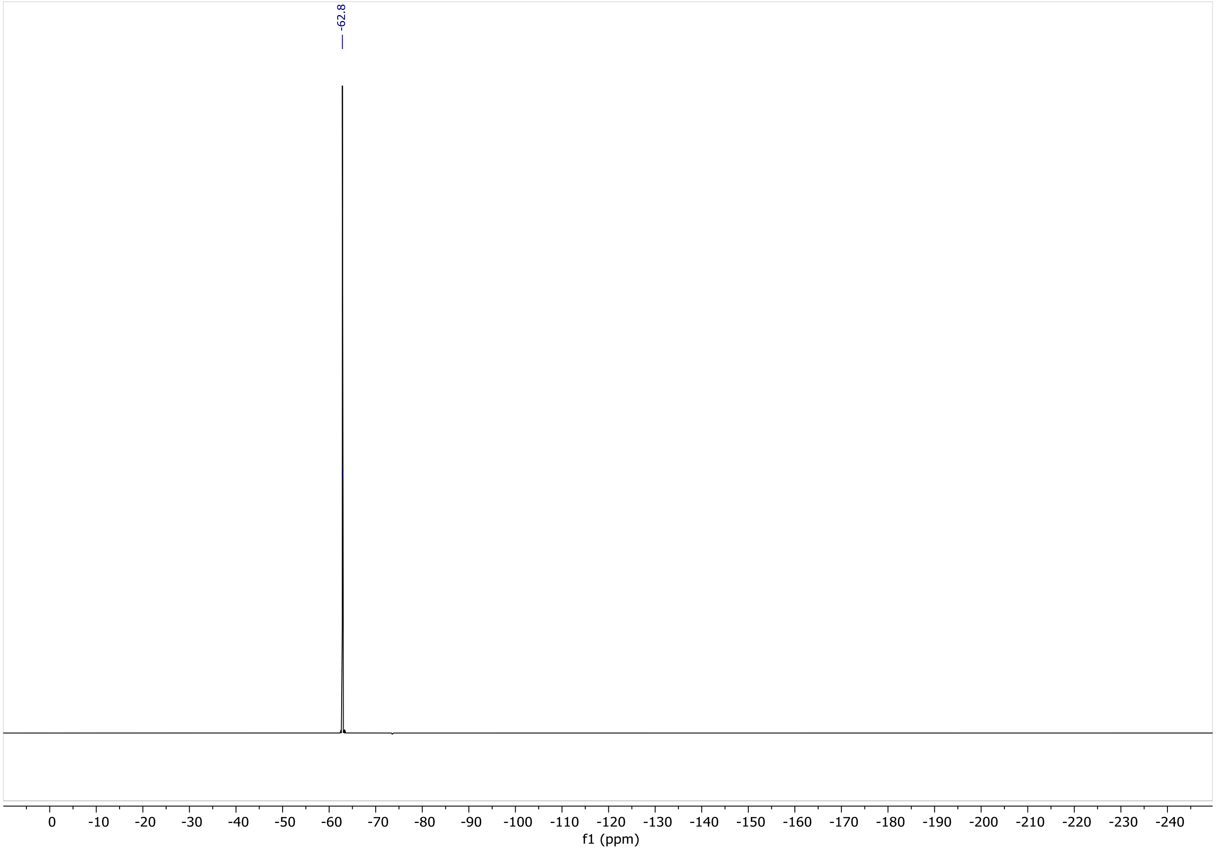

-62.8
0
-10
-20
-30
-40
-50
-60
-70
-80
-90
-100
-110
-120
-130
-140
-150
-160
-170
-180
-190
-200
-210
-220
-230
-240
f1 (ppm)

5-(3-(3,5-bis(trifluoromethyl)phenyl)propyl)pyridin-3-ol (68a)

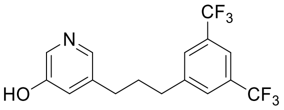

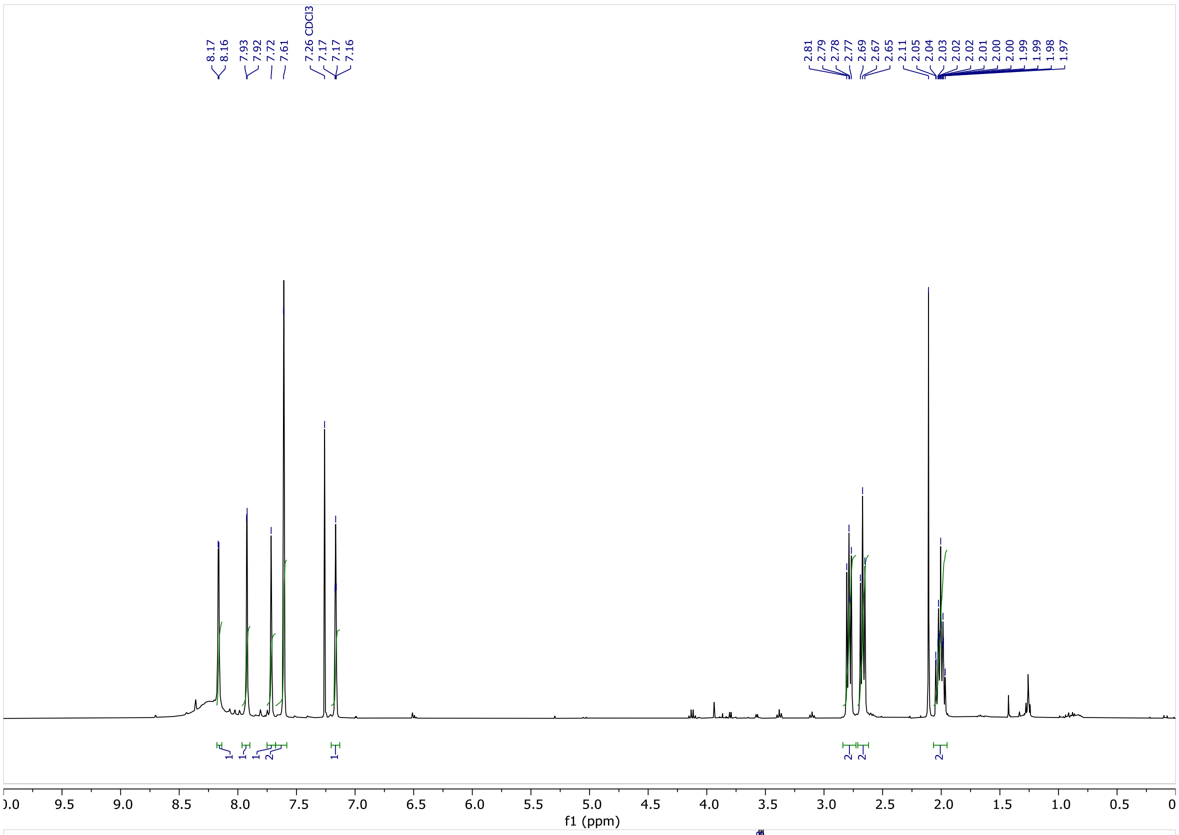

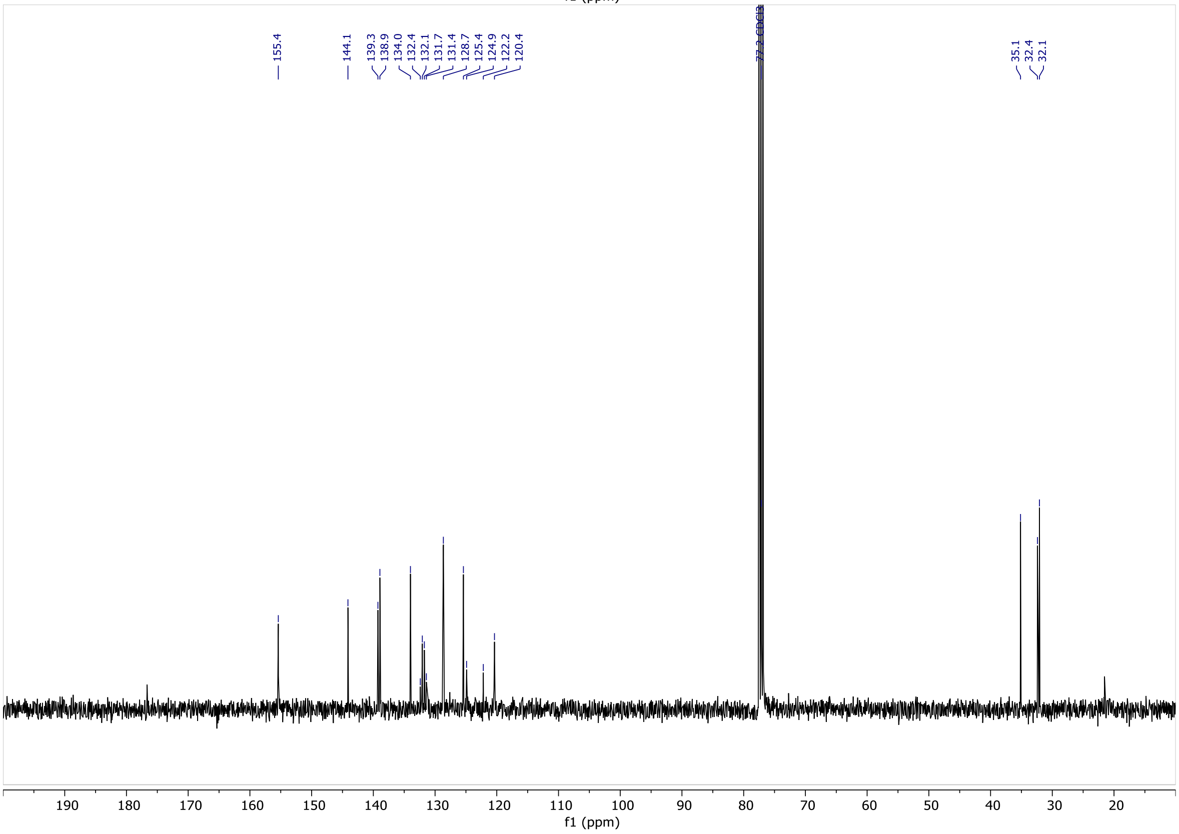

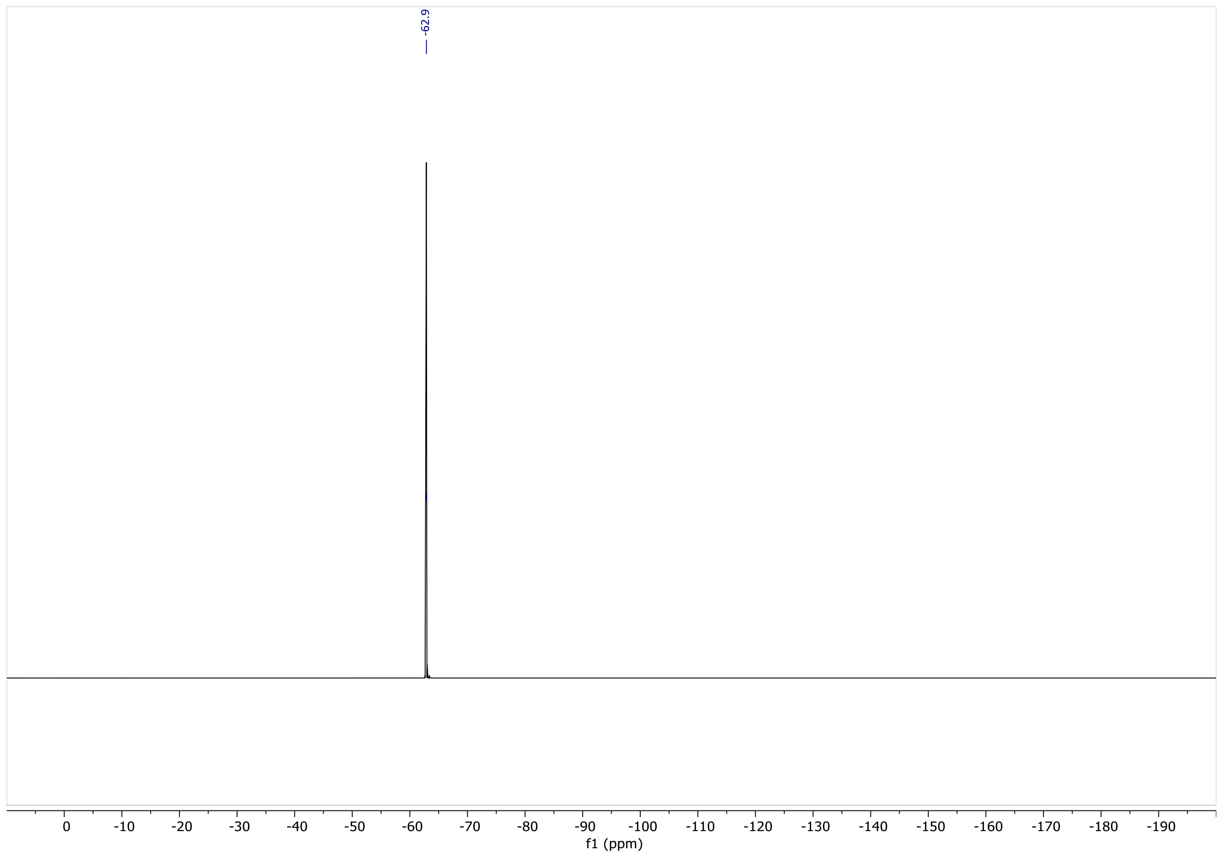
-62.9
0
-10
-20
-30
-40
-50
-60
-70
-80
-90
-100
-110
-120
-130
-140
-150
-160
-170
-180
-190
f1 (ppm)

3-(3-(3,5-bis(trifluoromethyl)phenyl)propyl)-5-hydroxypyridine 1-oxide (68)

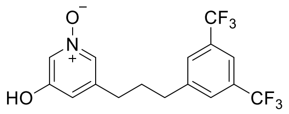

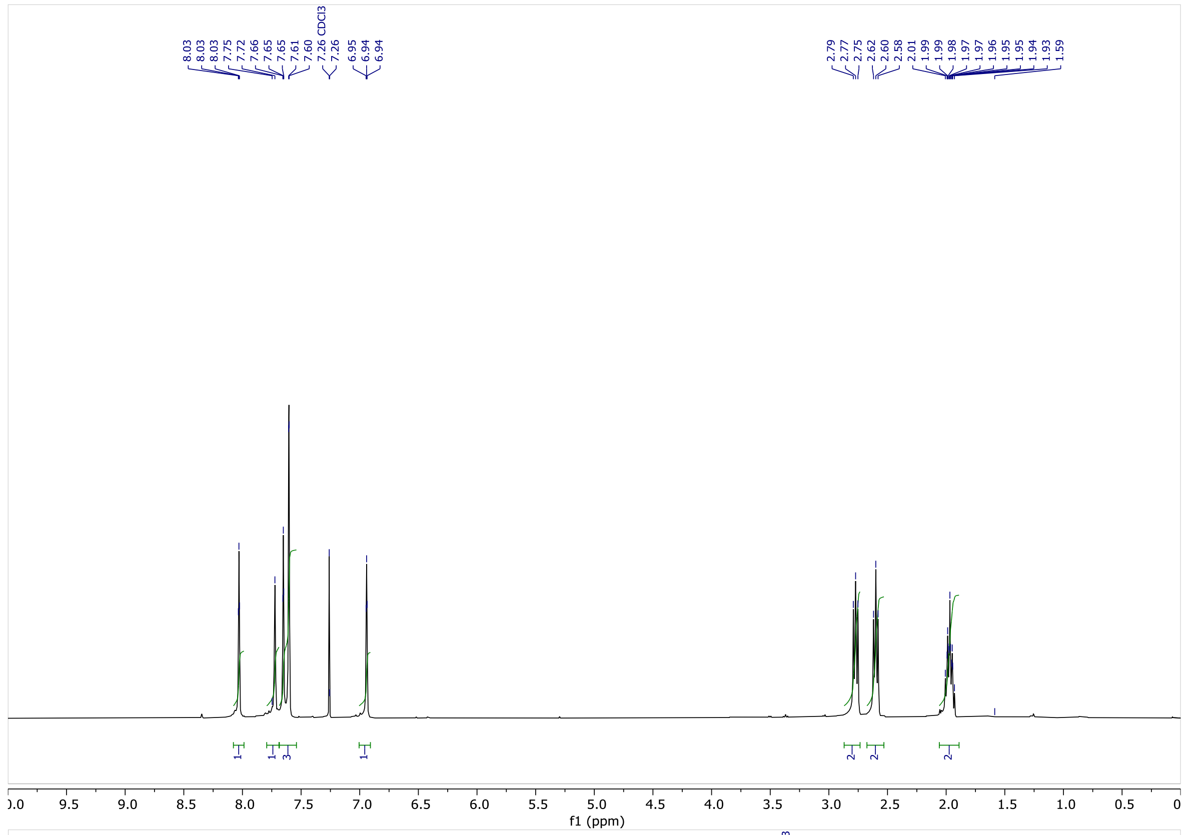

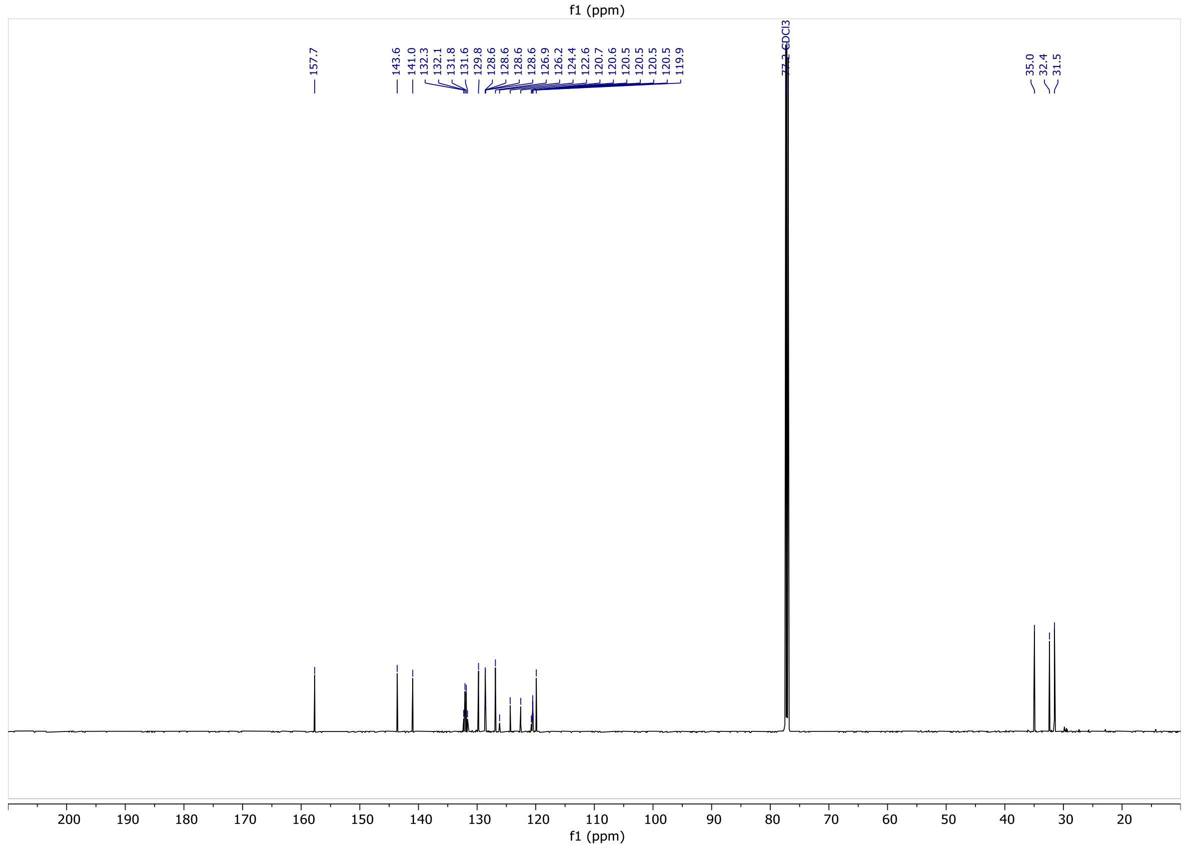

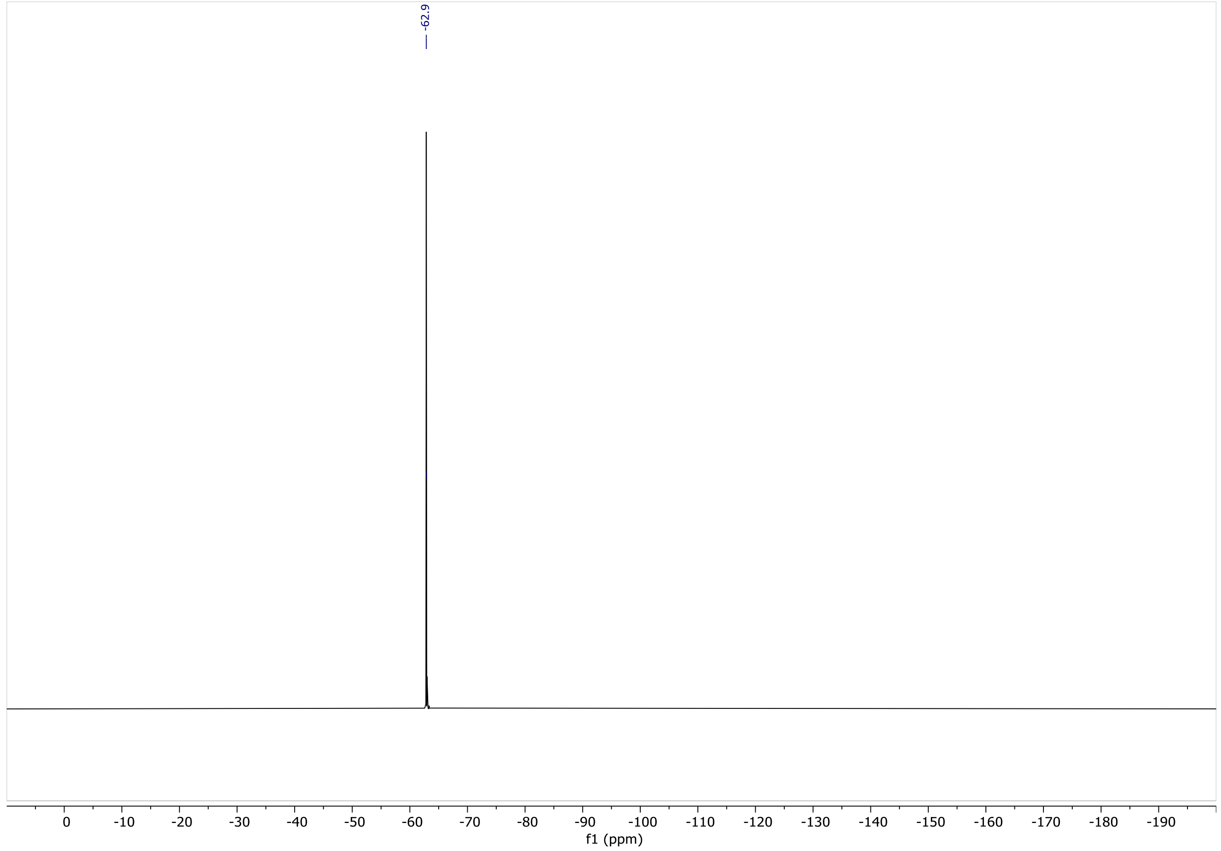
-62.9
0
-10
-20
-30
-40
-50
-60
-70
-80
-90
-100
-110
-120
-130
-140
-150
-160
-170
-180
-190
f1 (ppm)

(*E*)-1-(4-chloro-3-(trifluoromethyl)phenyl)-3-(5-methoxypyridin-3-yl)prop-2-en-1-one

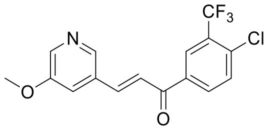

(69d)

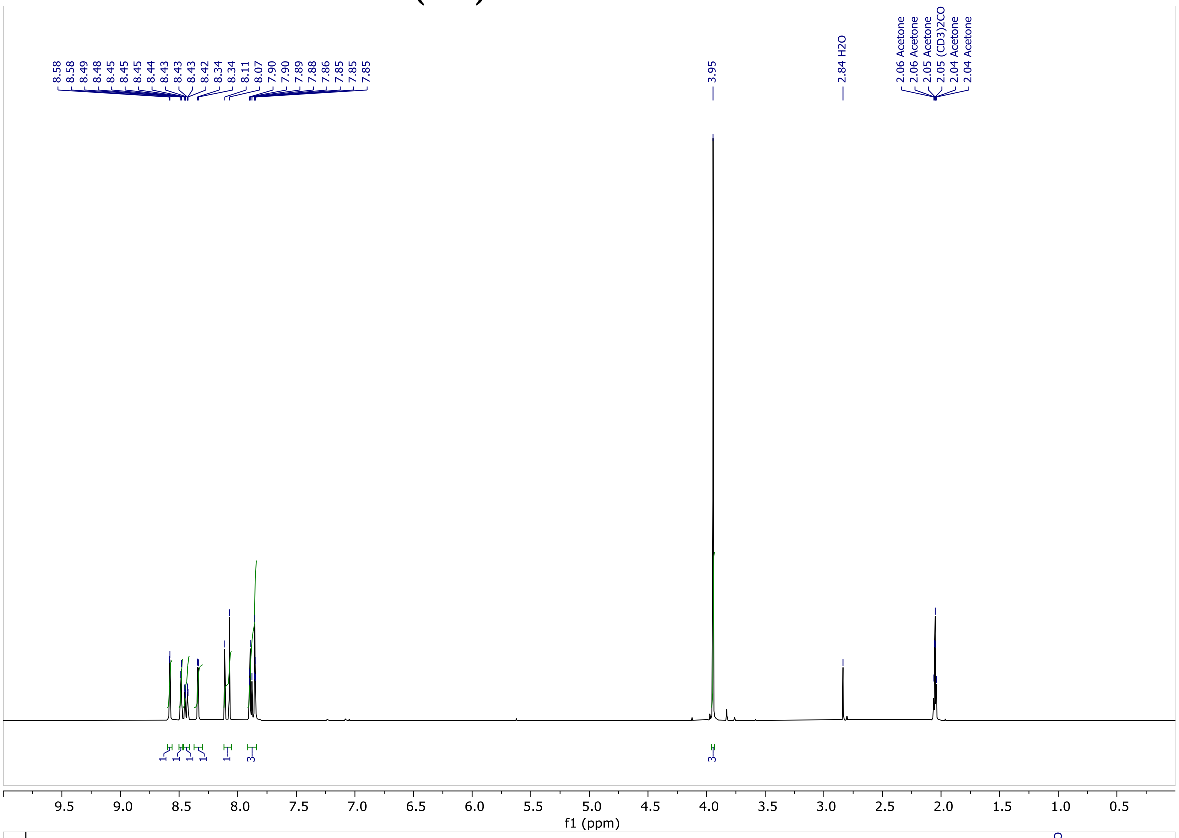

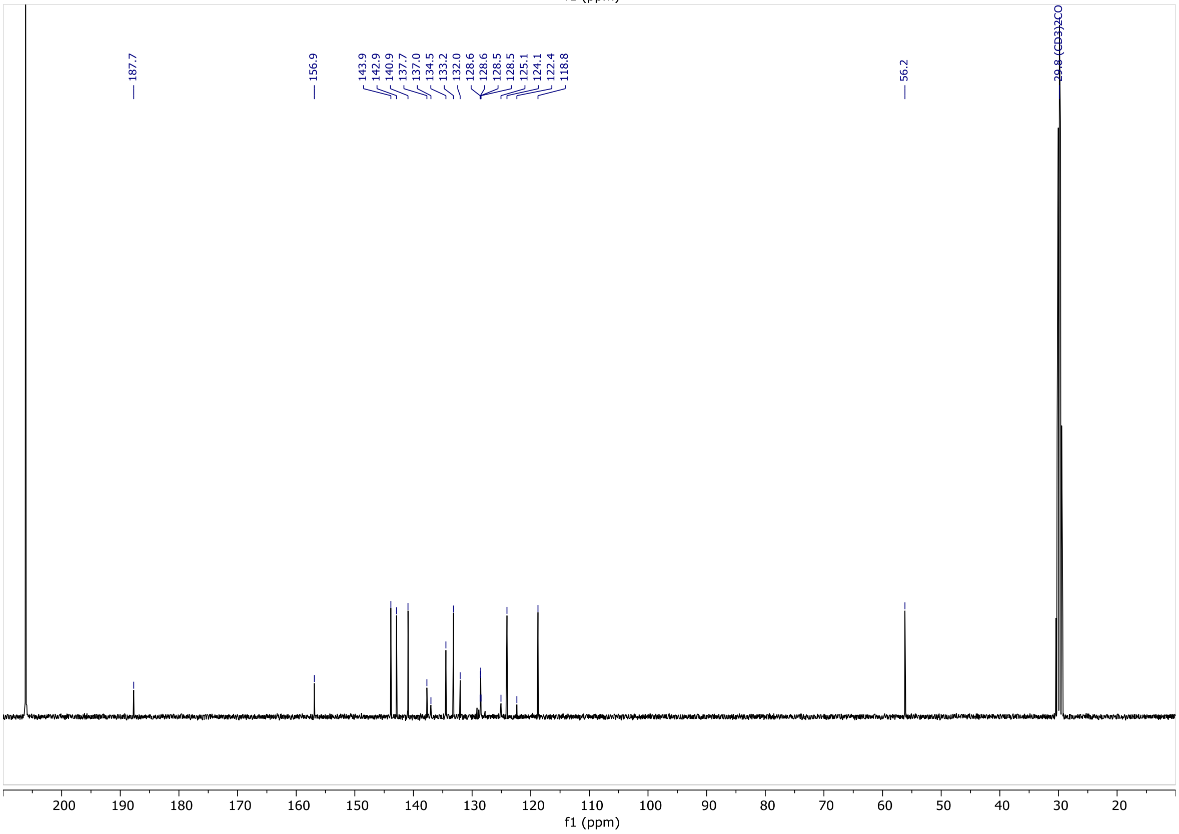

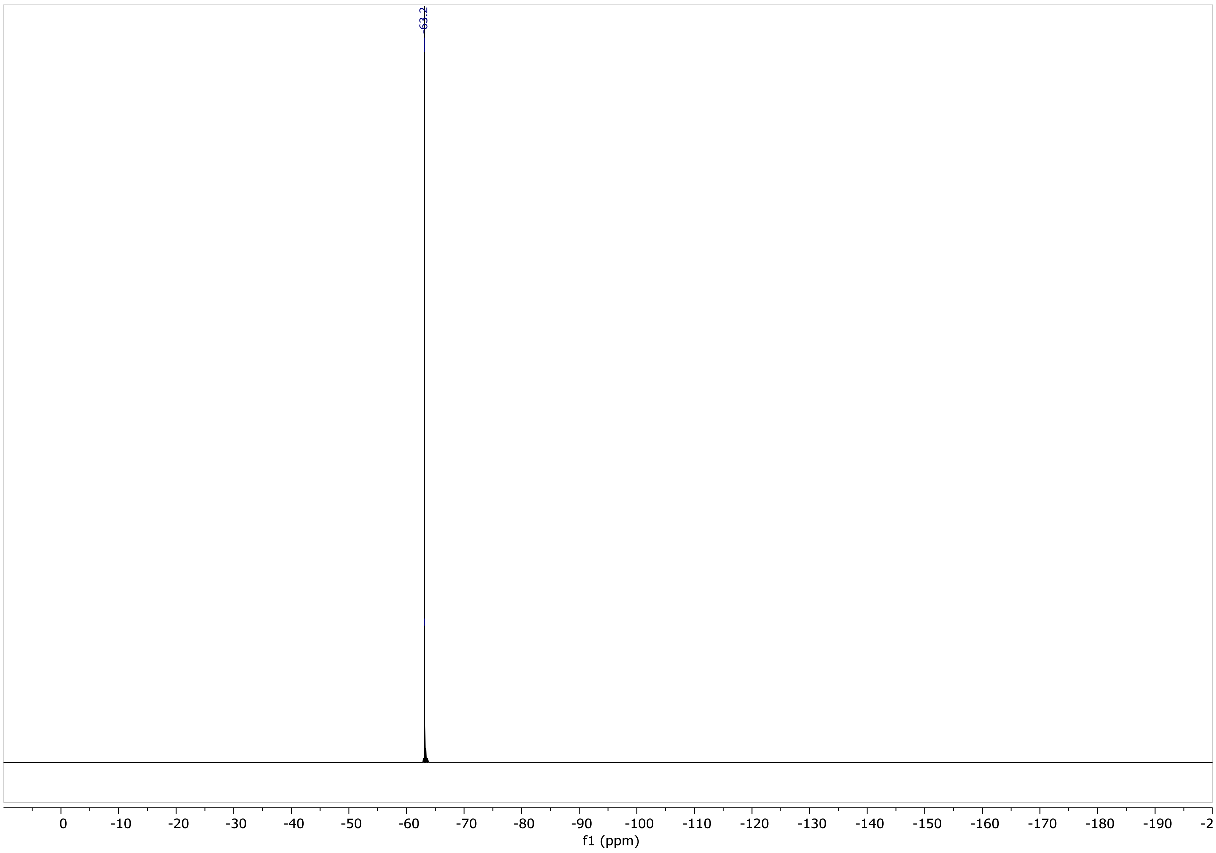
-63.2
0 -10 -20 -30 -40 -50 -60 -70 -80 -90 -100 -110 -120 -130 -140 -150 -160 -170 -180 -190
f1 (ppm)

(*E*)-3-(3-(4-chloro-3-(trifluoromethyl)phenyl)prop-1-en-1-yl)-5-methoxypyridine (69c)

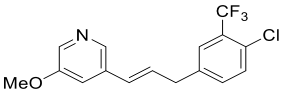

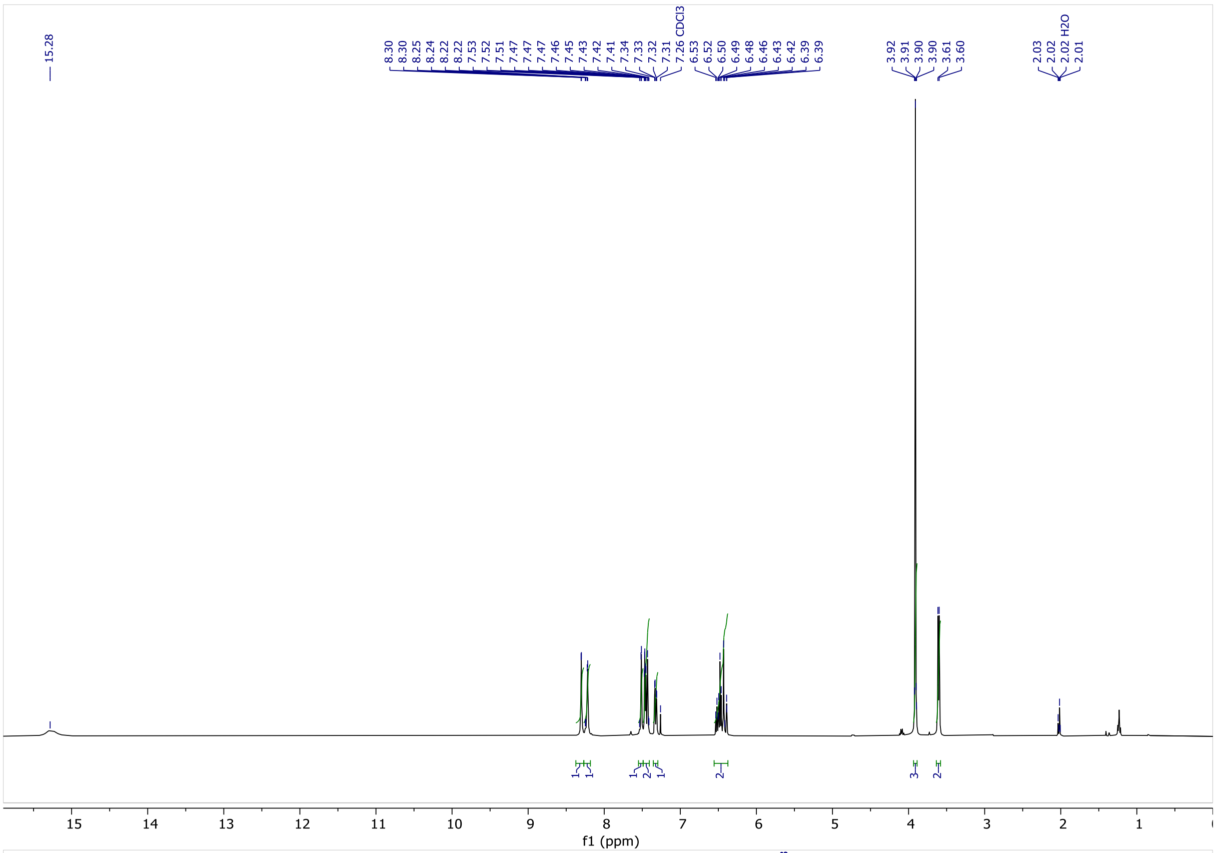

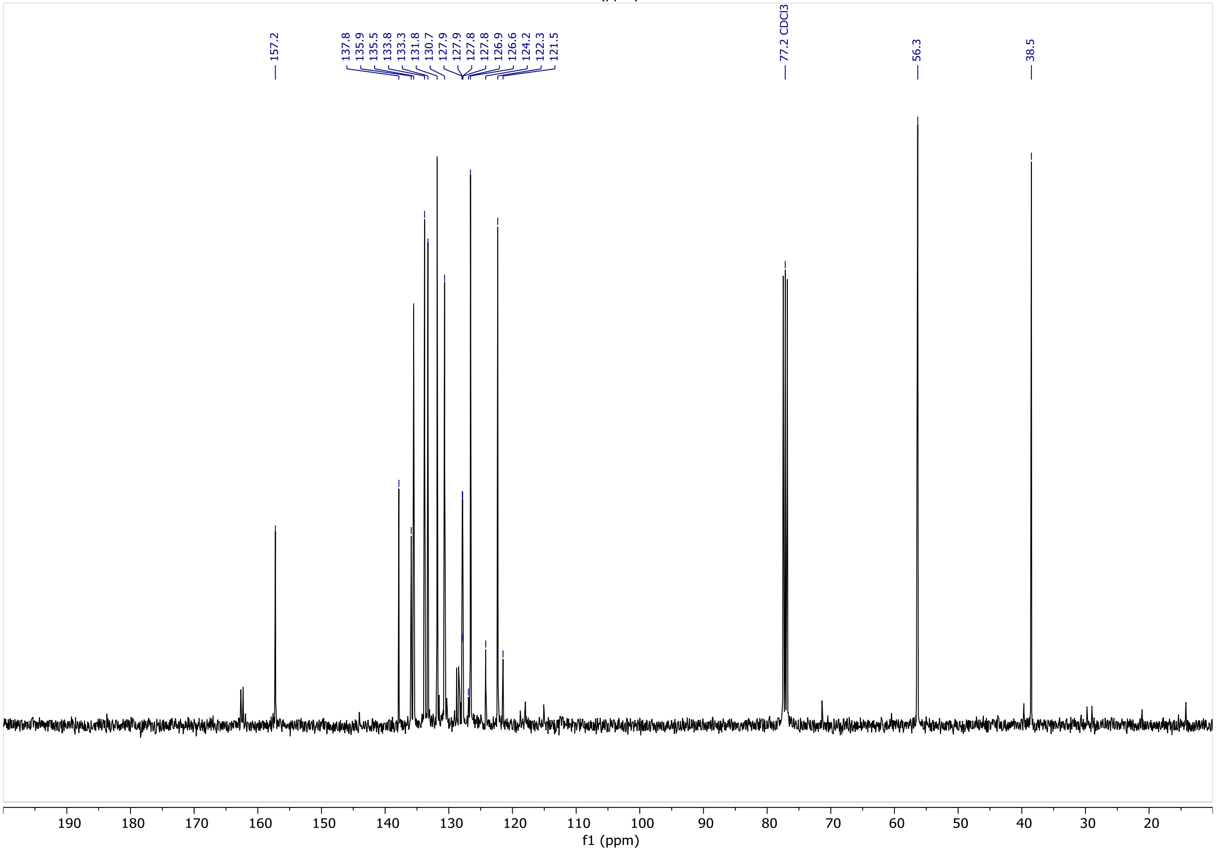

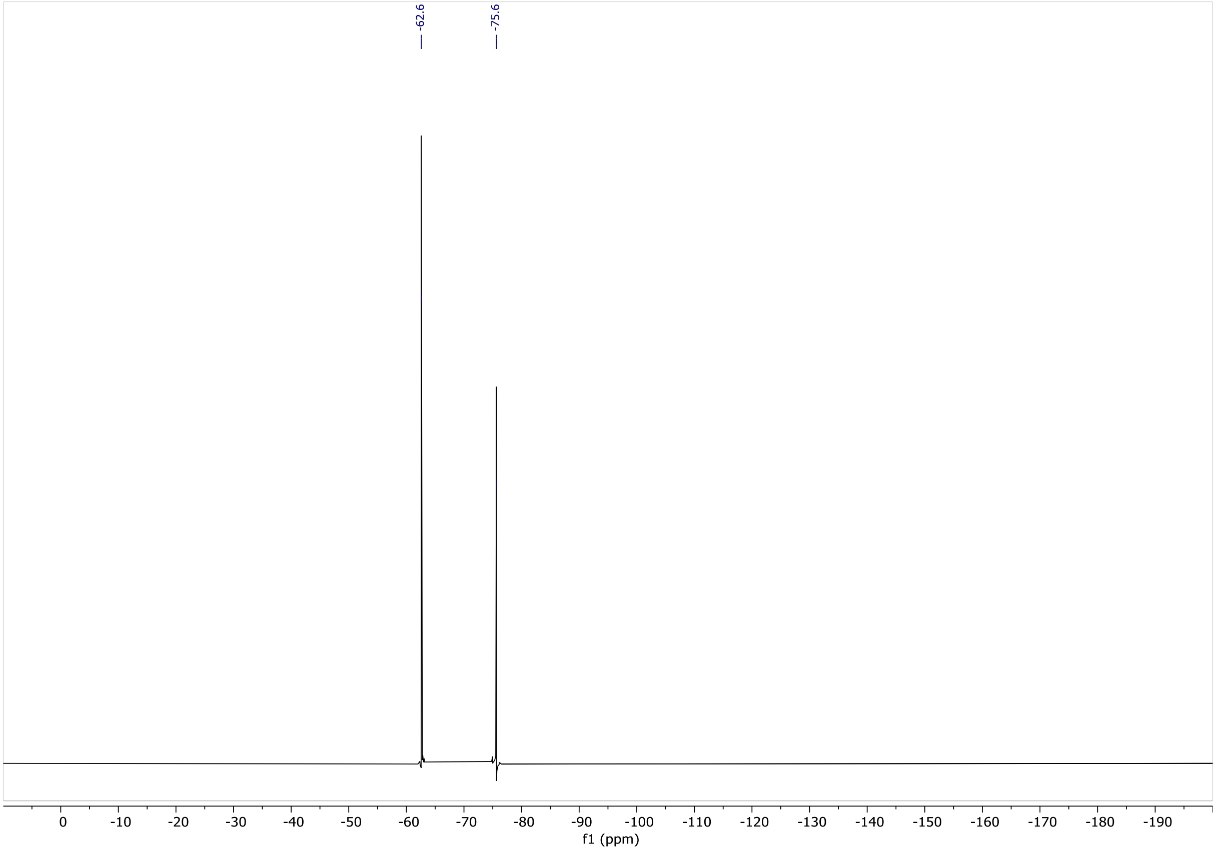
-62.6
-75.6
0 -10 -20 -30 -40 -50 -60 -70 -80 -90 -100 -110 -120 -130 -140 -150 -160 -170 -180 -190
f1 (ppm)

3-(3-(4-chloro-3-(trifluoromethyl)phenyl)propyl)-5-methoxypyridine (69b)

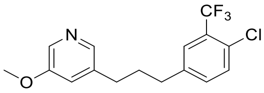

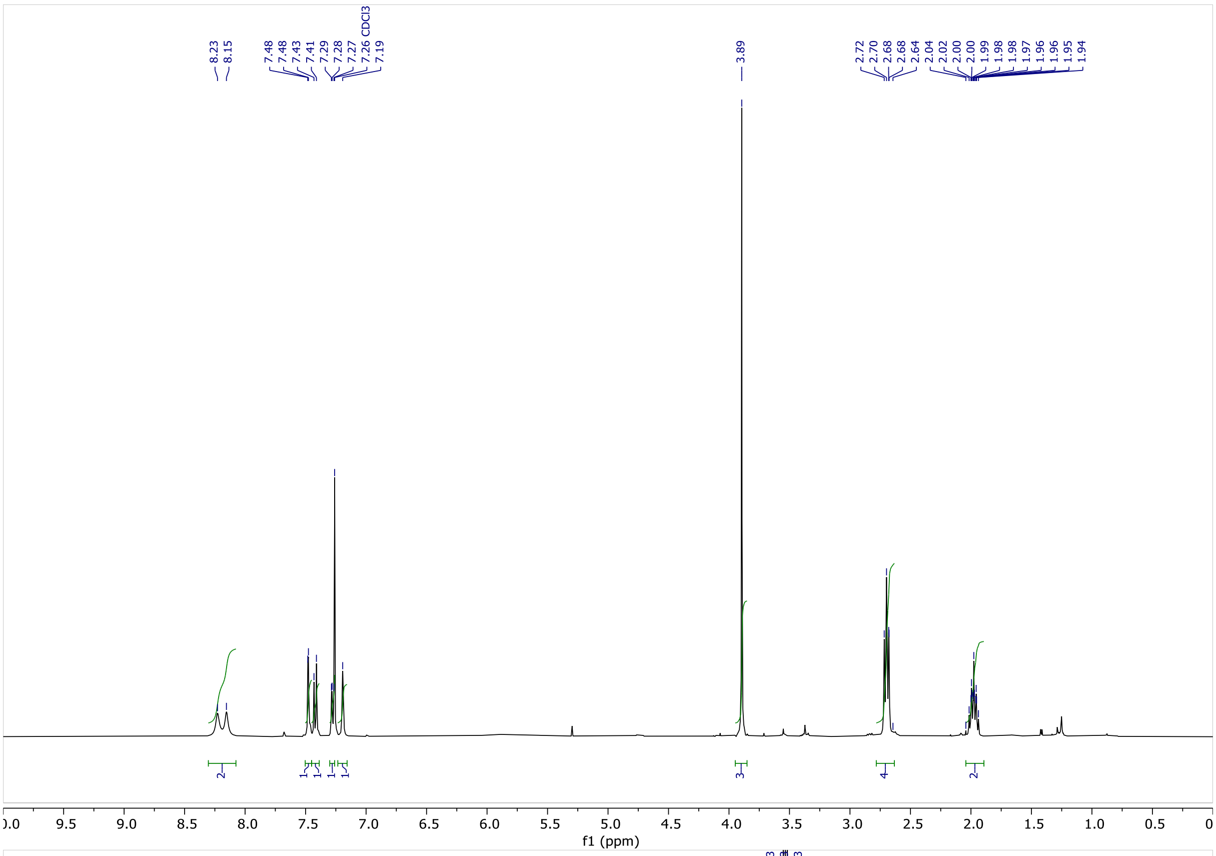

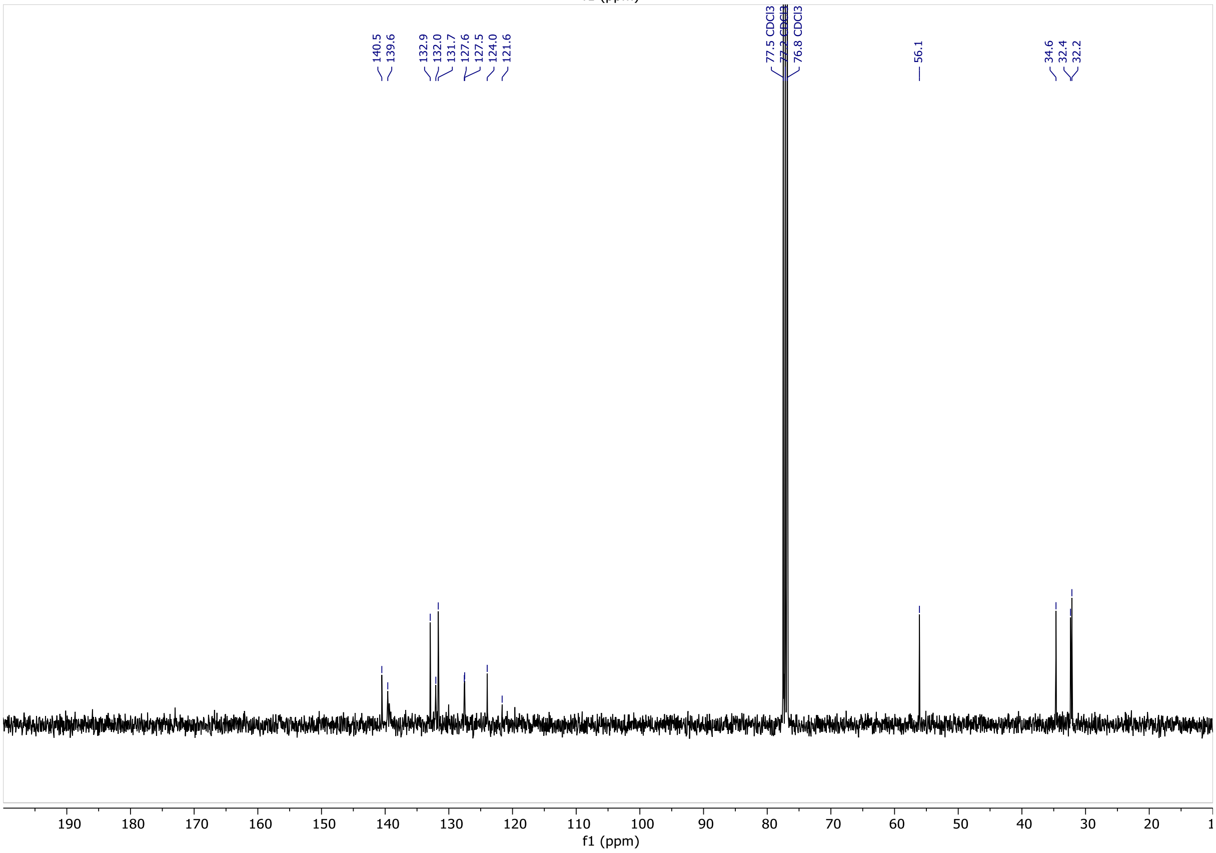

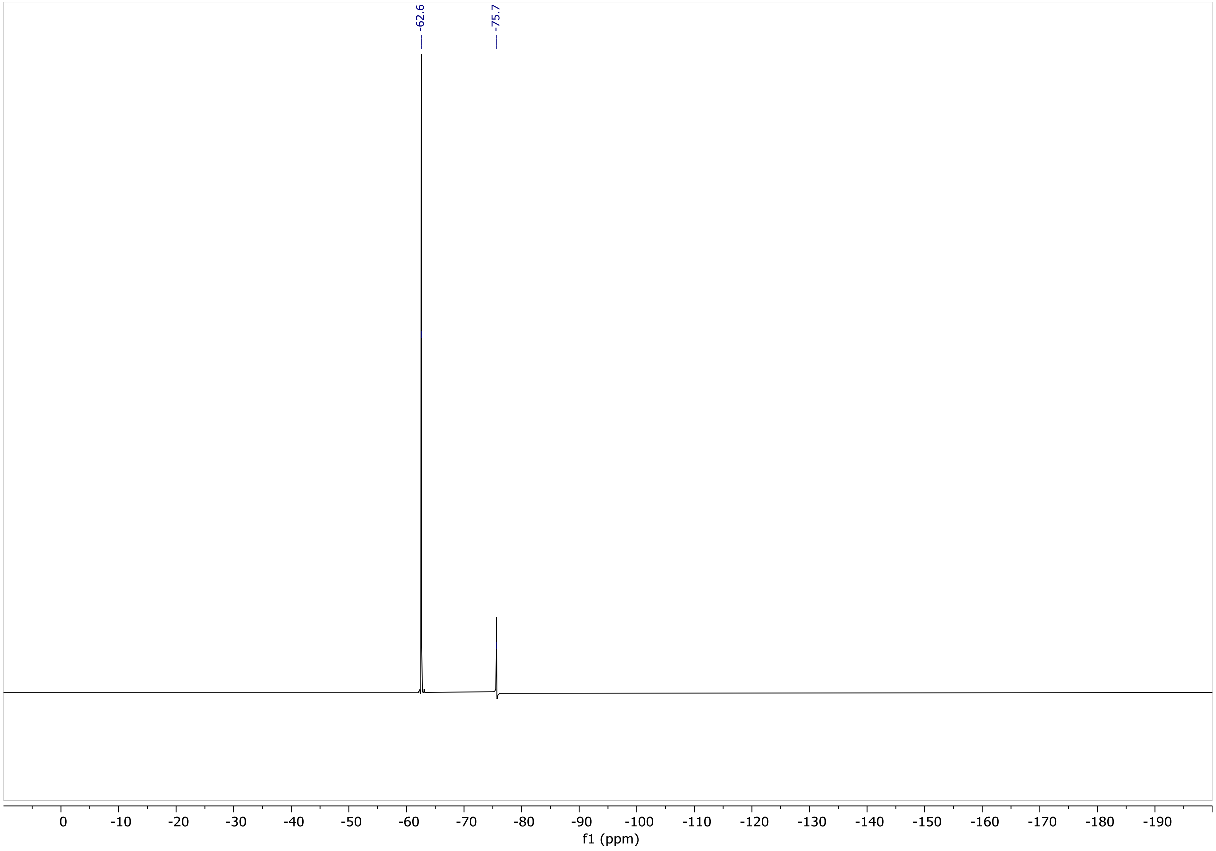
-62.6
-75.7
0
-10
-20
-30
-40
-50
-60
-70
-80
-90
-100
-110
-120
-130
-140
-150
-160
-170
-180
-190
f1 (ppm)

5-(3-(4-chloro-3-(trifluoromethyl)phenyl)propyl)pyridin-3-ol (69a)

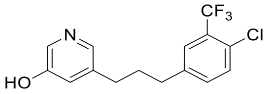

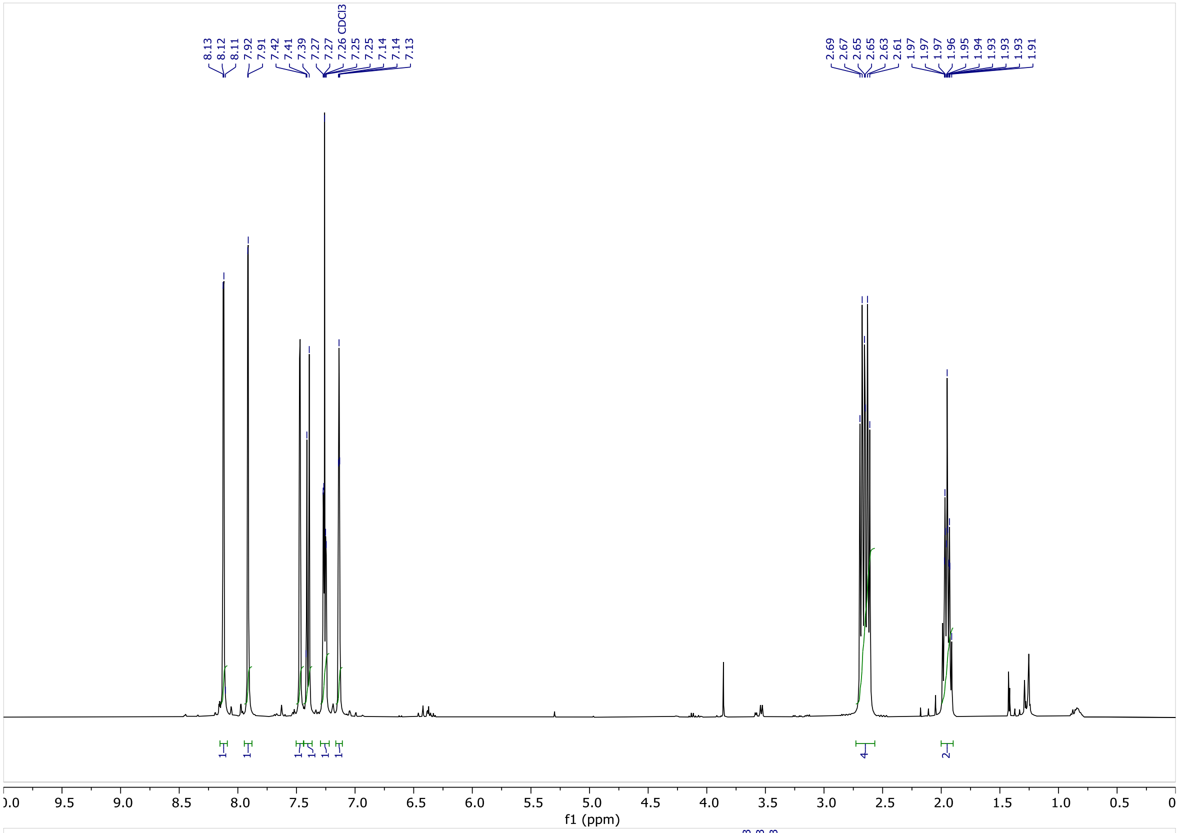

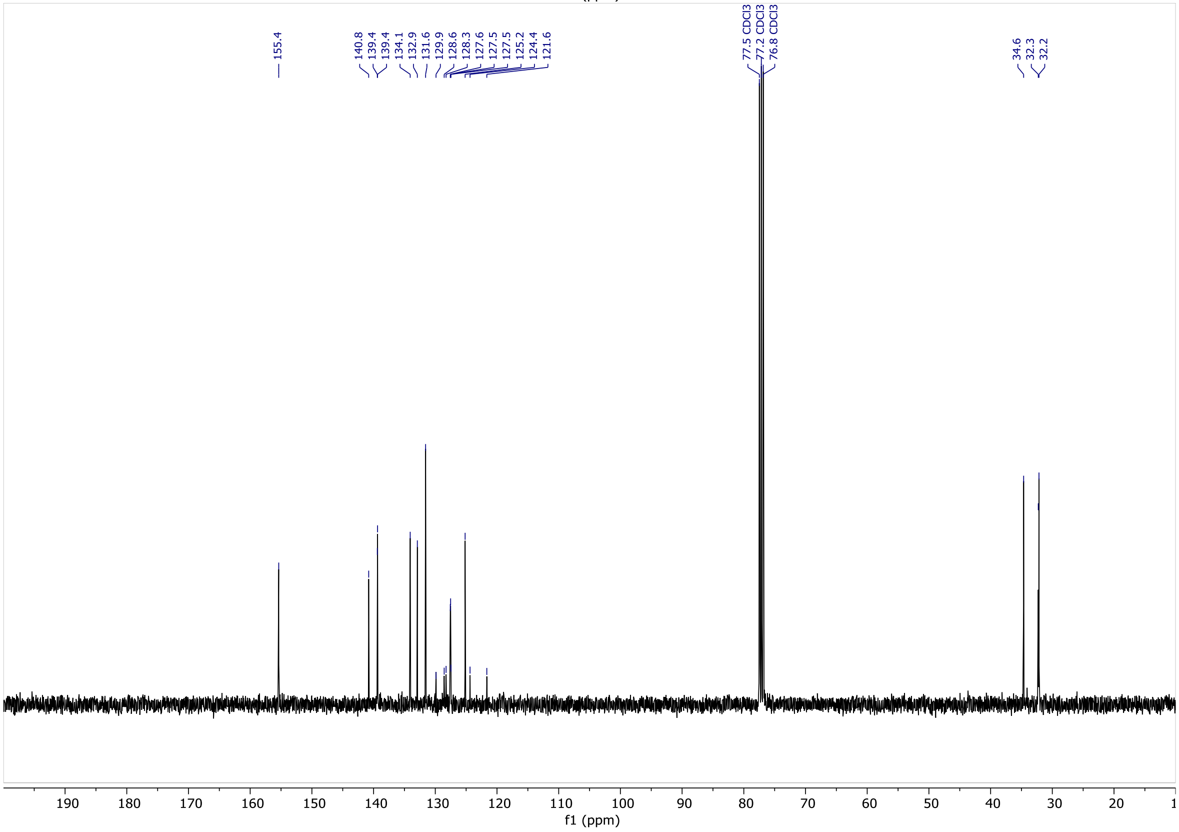

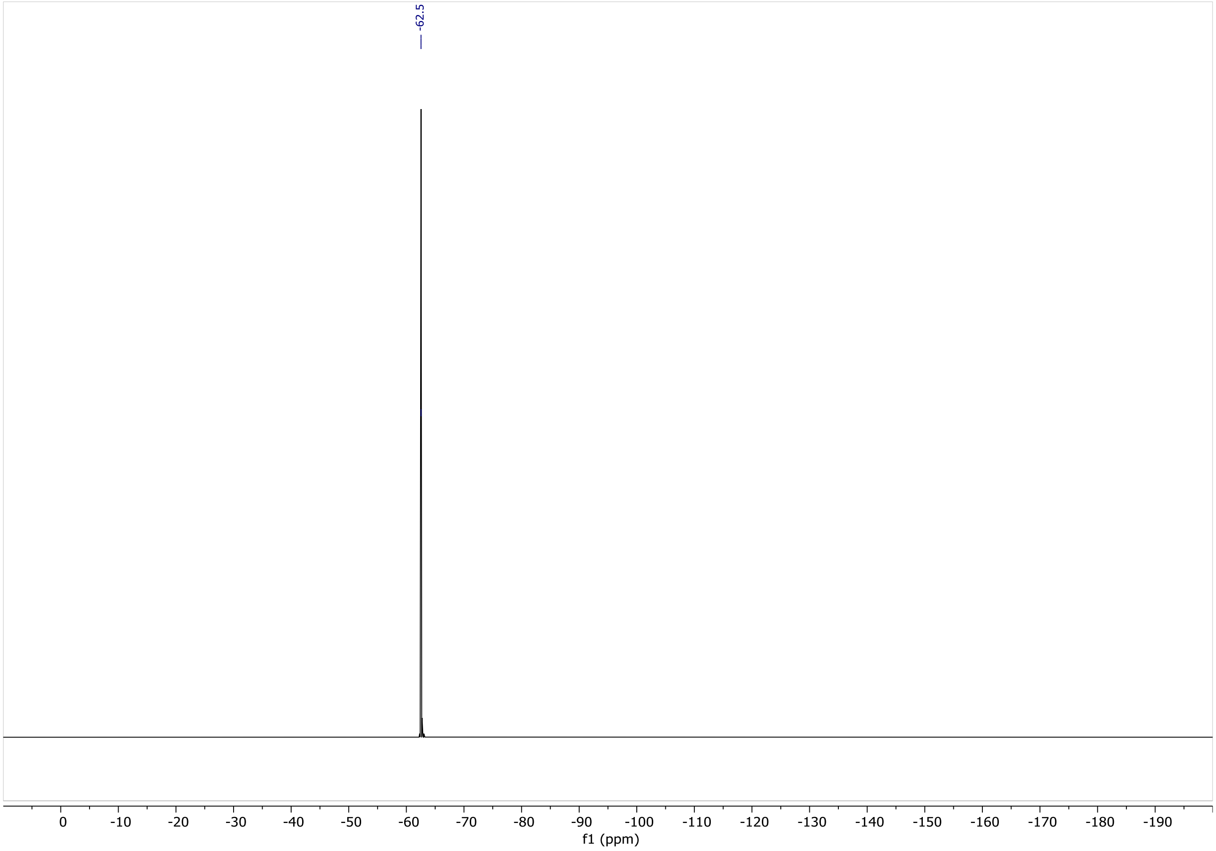
-62.5
0
-10
-20
-30
-40
-50
-60
-70
-80
-90
-100
-110
-120
-130
-140
-150
-160
-170
-180
-190
f1 (ppm)

3-(3-(4-chloro-3-(trifluoromethyl)phenyl)propyl)-5-hydroxypyridine 1-oxide (69)

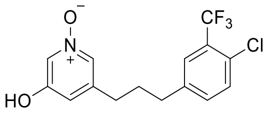

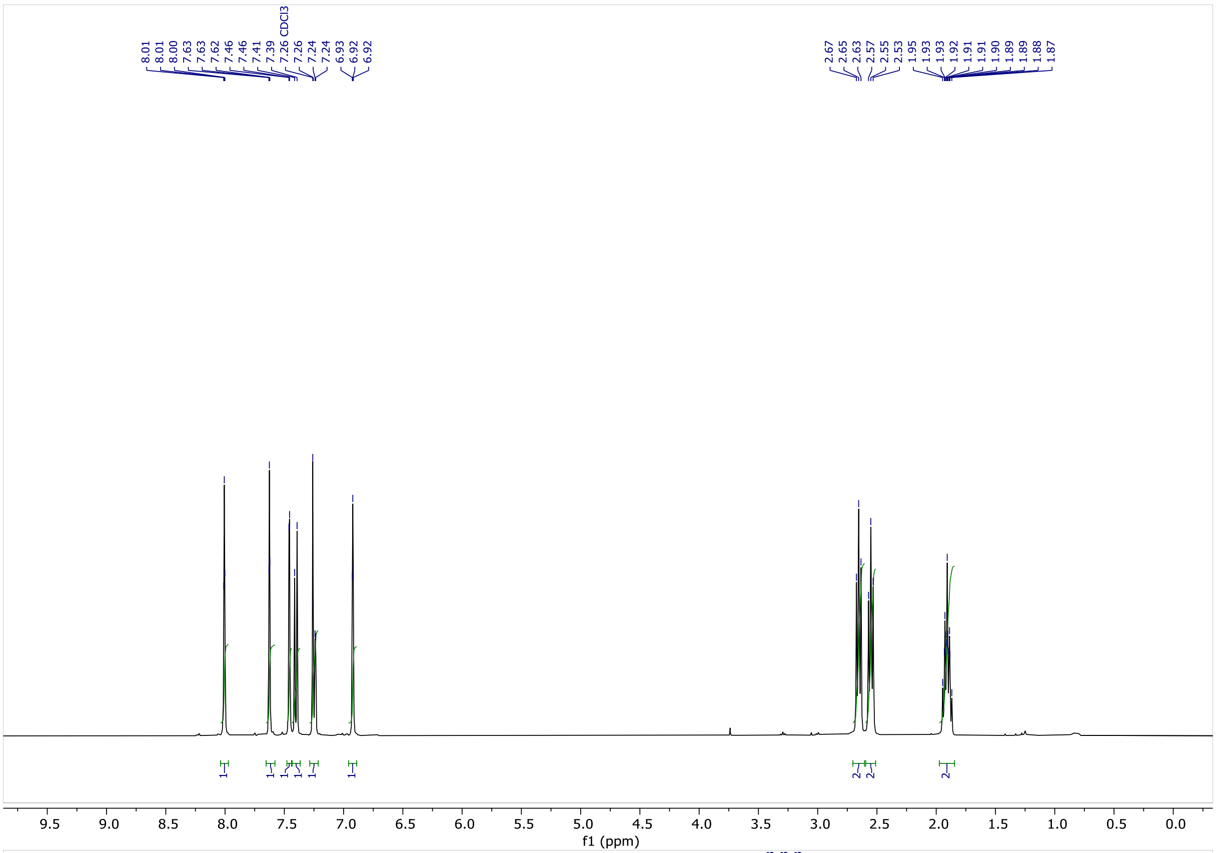

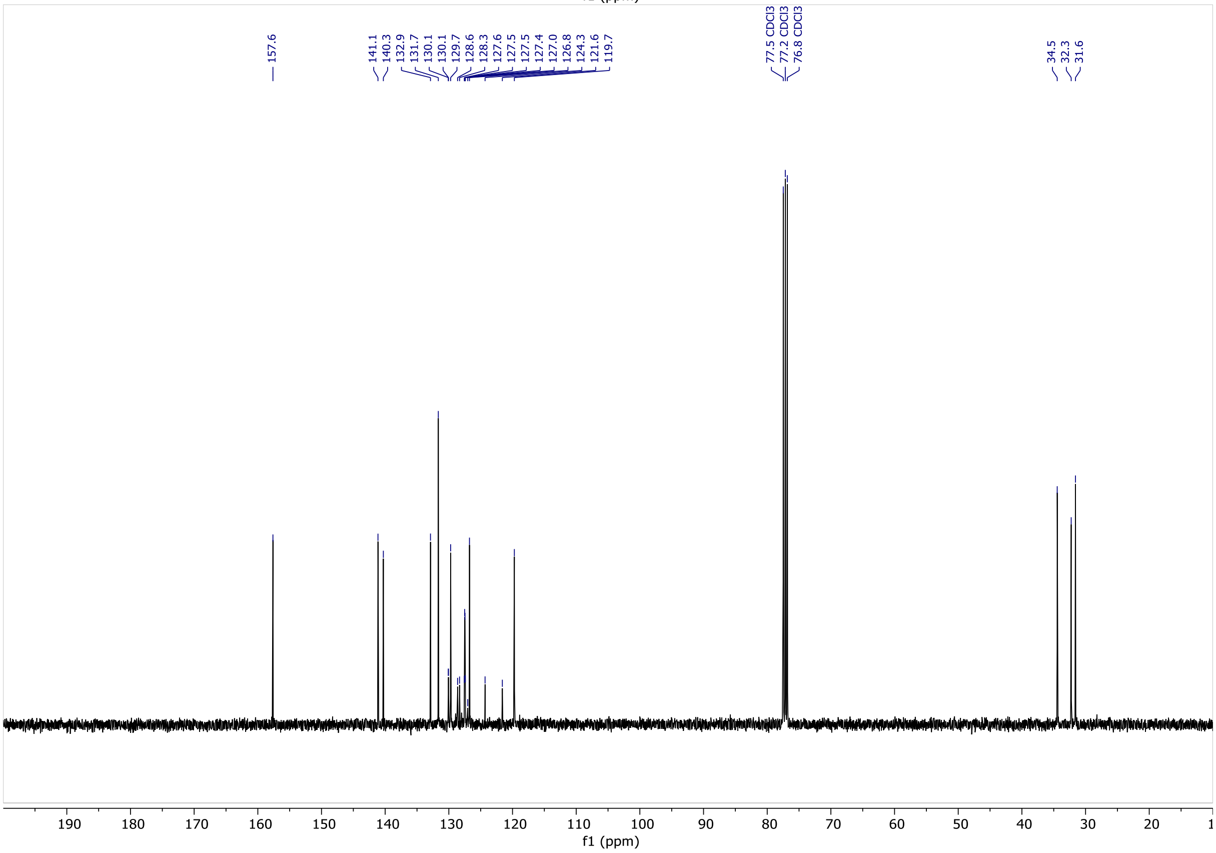

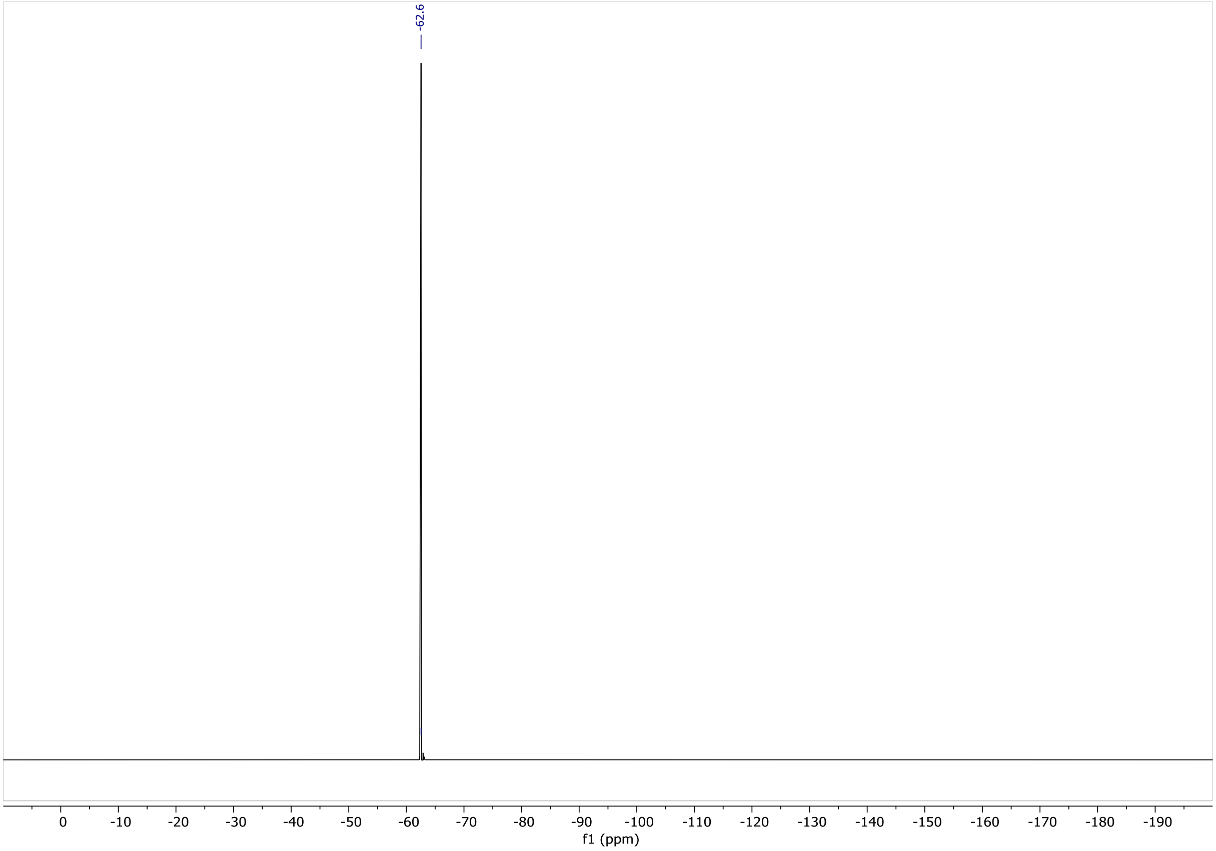

-62.6
0 -10 -20 -30 -40 -50 -60 -70 -80 -90 -100 -110 -120 -130 -140 -150 -160 -170 -180 -190
f1 (ppm)

HPLC traces for final products

3-(2-((4-bromonaphthalen-1-yl)oxy)ethyl)pyridine 1-oxide (12)

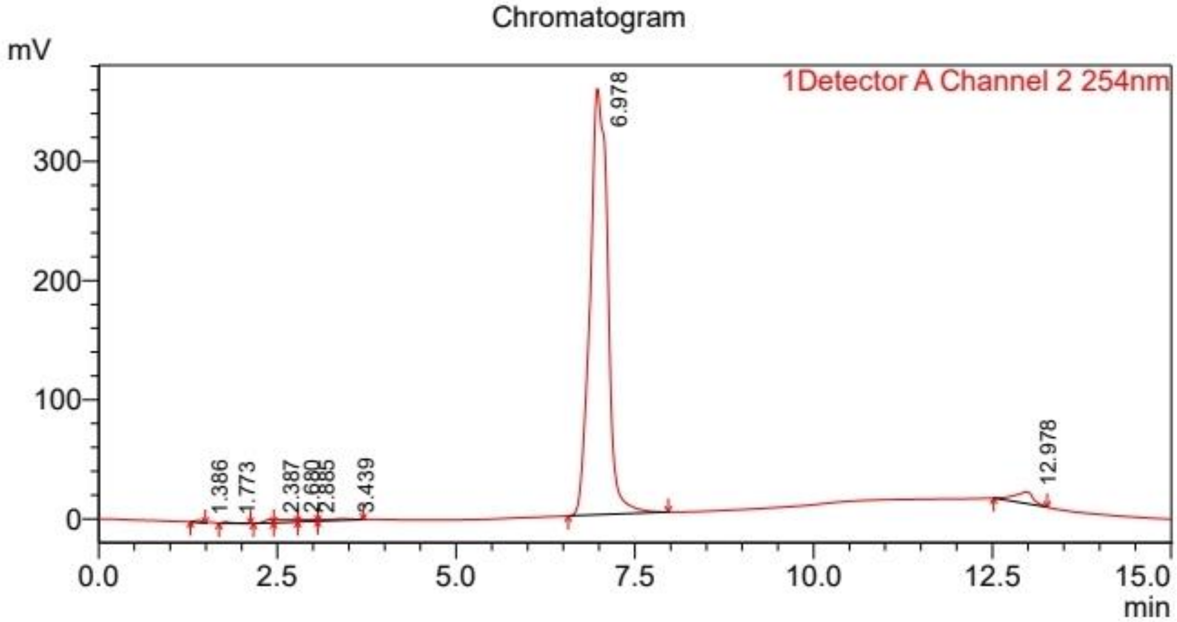

Detector A Channel 2 254nm

Peak#	Ret. Time (min)	Area%
1	1.386	0.189
2	1.773	0.151
3	2.387	0.369
4	2.680	0.697
5	2.885	0.419
6	3.439	0.510
7	6.978	94.895
8	12.978	2.771
Total		100.000

3-(2-((4-fluoronaphthalen-1-yl)oxy)ethyl)pyridine 1-oxide (13)

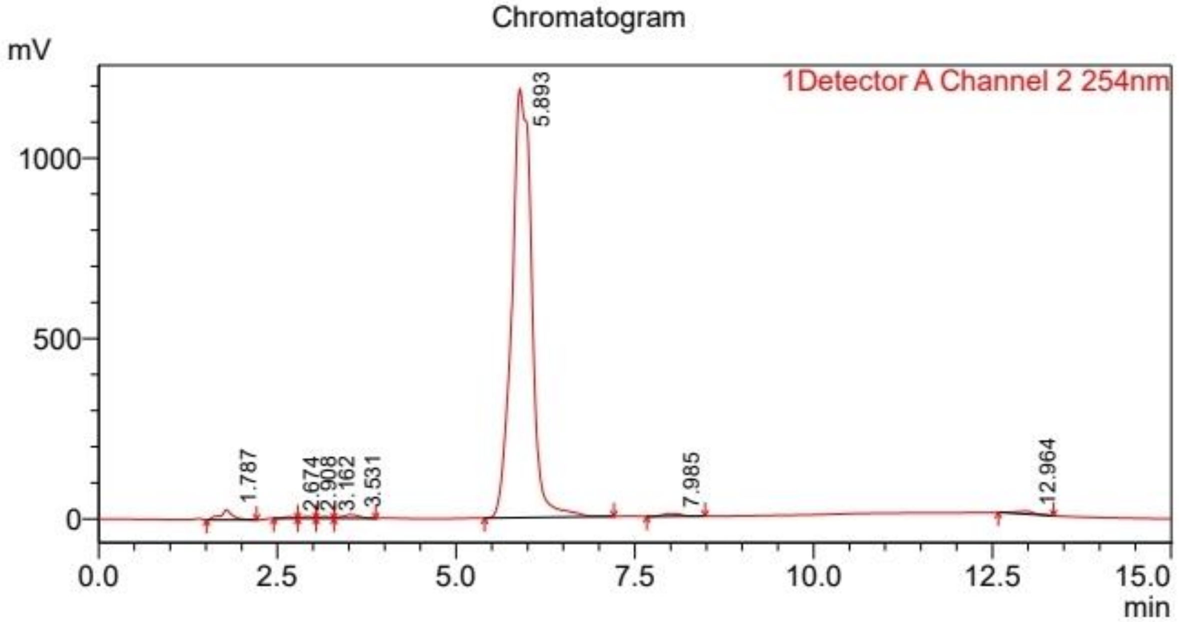

Detector A Channel 2 254nm

Peak#	Ret. Time (min)	Area%
1	1.787	1.387
2	2.674	0.205
3	2.908	0.239
4	3.162	0.228
5	3.531	0.676
6	5.893	96.070
7	7.985	0.585
8	12.964	0.611
Total		100.000

3-(2-((4-methoxynaphthalen-1-yl)oxy)ethyl)pyridine 1-oxide (14)

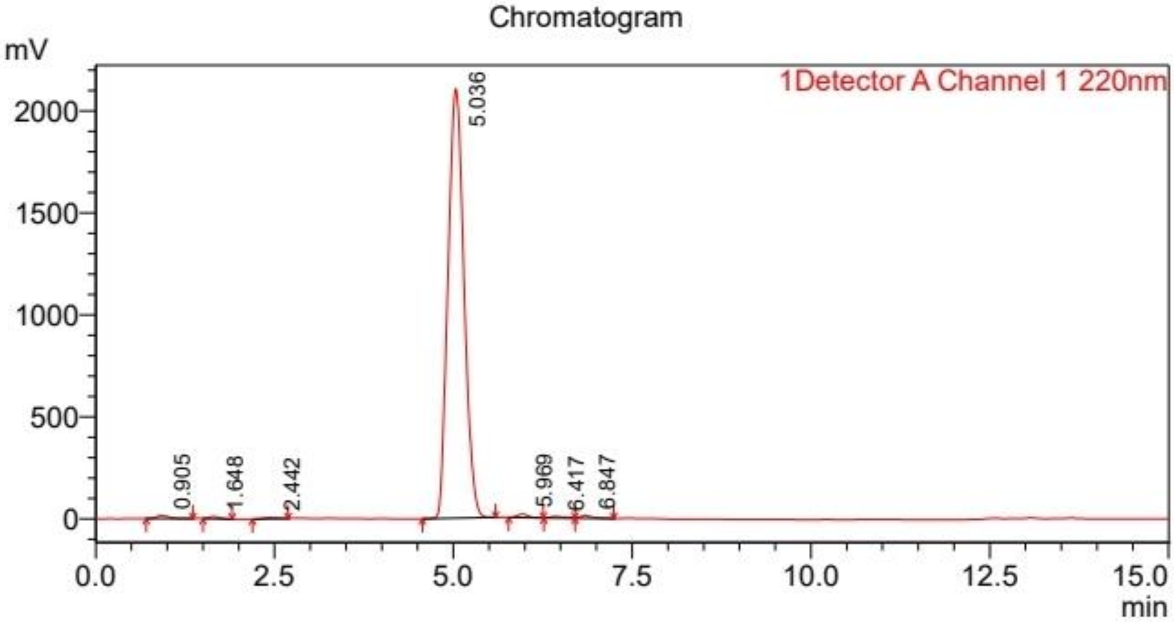

Detector A Channel 1 220nm

Peak#	Ret. Time (min)	Area%
1	0.905	0.535
2	1.648	0.299
3	2.442	0.241
4	5.036	97.681
5	5.969	0.506
6	6.417	0.310
7	6.847	0.430
Total		100.000

3-(2-(naphthalen-1-yloxy)ethyl)pyridine 1-oxide (15)

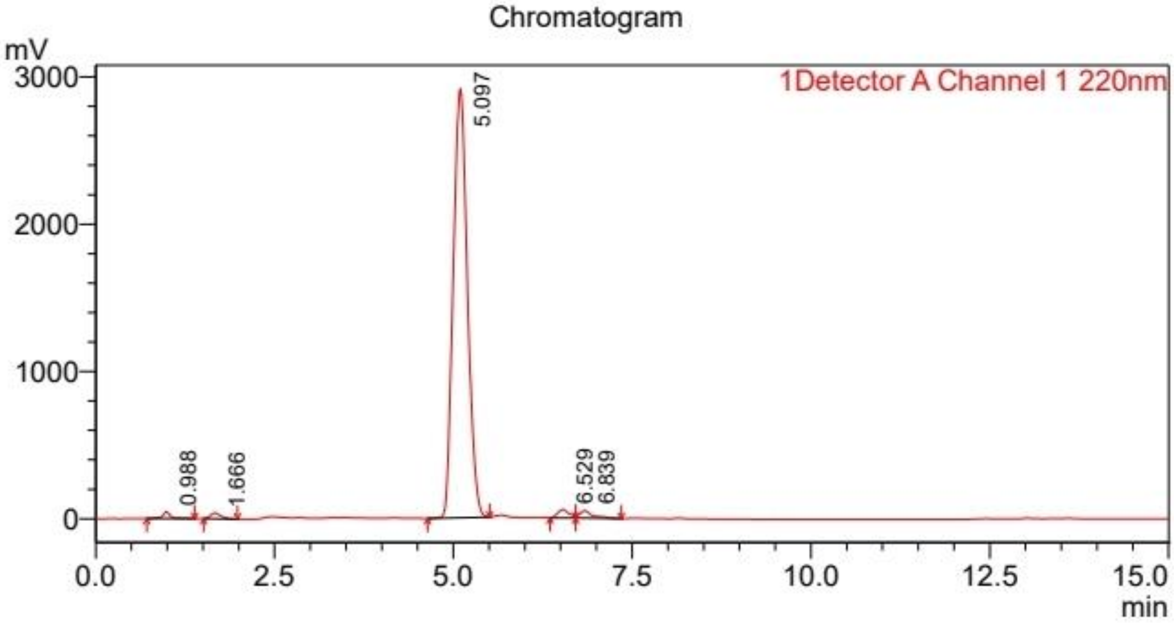

Detector A Channel 1 220nm

Peak#	Ret. Time (min)	Area%
1	0.988	0.897
2	1.666	0.904
3	5.097	95.150
4	6.529	1.507
5	6.839	1.542
Total		100.000

3-(2-((4-chloro-5,6,7,8-tetrahydronaphthalen-1-yl)oxy)ethyl)pyridine 1-oxide (16)

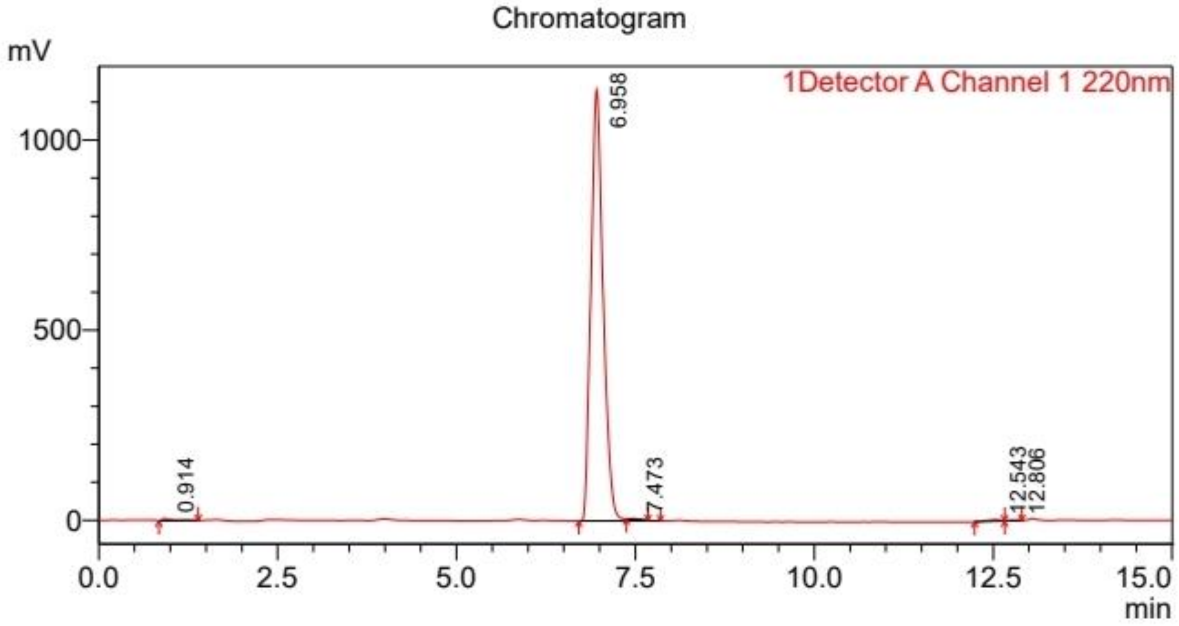

Detector A Channel 1 220nm

Peak#	Ret. Time (min)	Area%
1	0.914	0.410
2	6.958	98.941
3	7.473	0.138
4	12.543	0.428
5	12.806	0.083
Total		100.000

3-(2-((2,3-dihydro-1H-inden-1-yl)oxy)ethyl)pyridine 1-oxide (18)

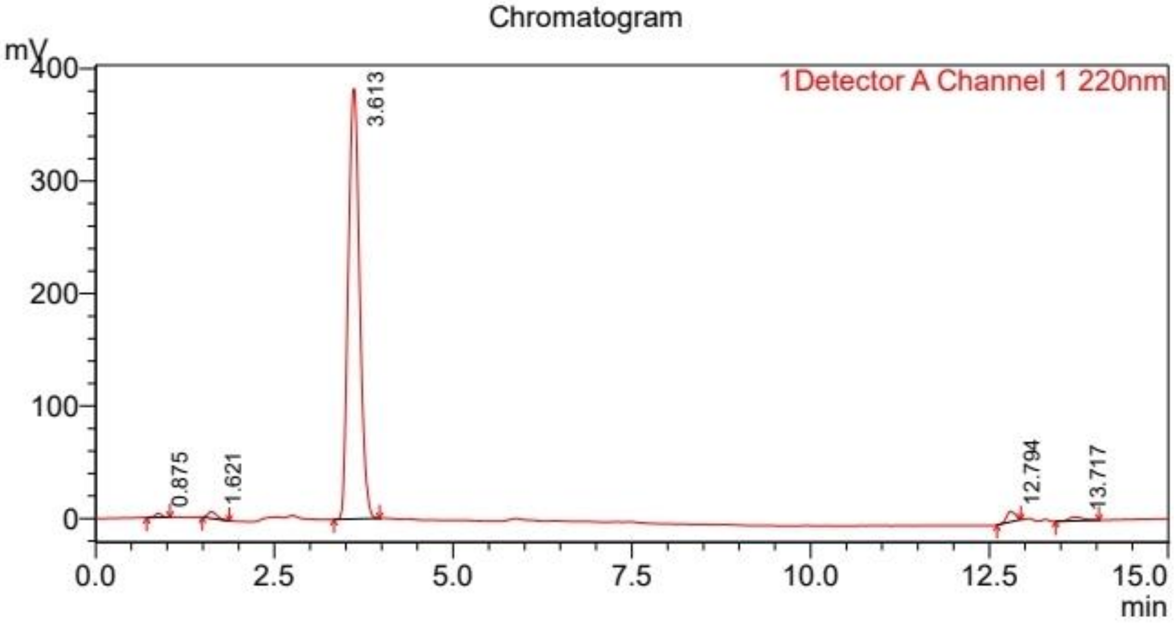

Detector A Channel 1 220nm

Peak#	Ret. Time (min)	Area%
1	0.875	0.572
2	1.621	1.149
3	3.613	95.390
4	12.794	1.834
5	13.717	1.055
Total		100.000

3-(2-(4-chlorophenoxy)ethyl)pyridine 1-oxide (19)

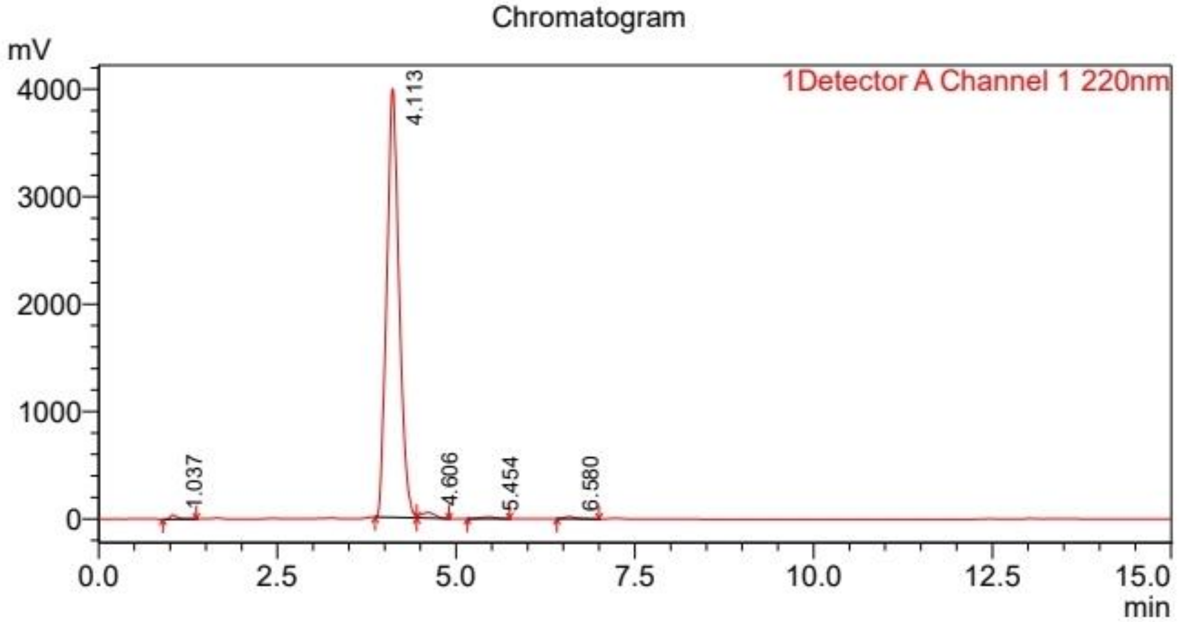

Detector A Channel 1 220nm

Peak#	Ret. Time (min)	Area%
1	1.037	0.545
2	4.113	97.102
3	4.606	1.426
4	5.454	0.489
5	6.580	0.438
Total		100.000

3-(2-(4-bromophenoxy)ethyl)pyridine 1-oxide (20)

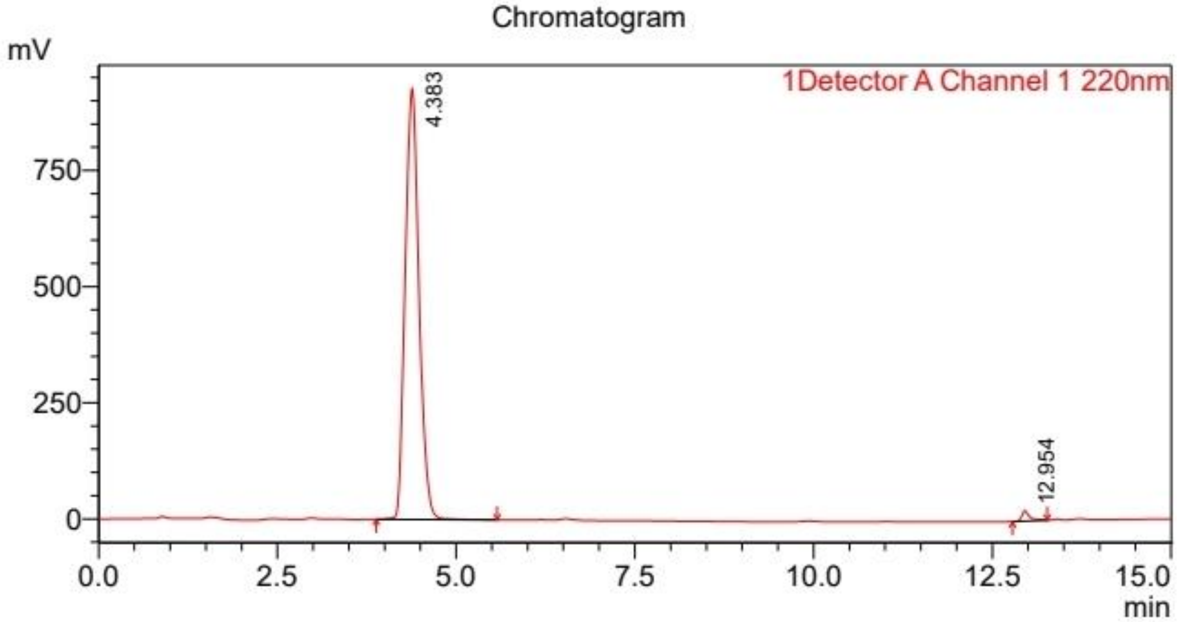

Detector A Channel 1 220nm

Peak#	Ret. Time (min)	Area%
1	4.383	98.664
2	12.954	1.336
Total		100.000

3-(2-(4-cyanophenoxy)ethyl)pyridine 1-oxide (21)

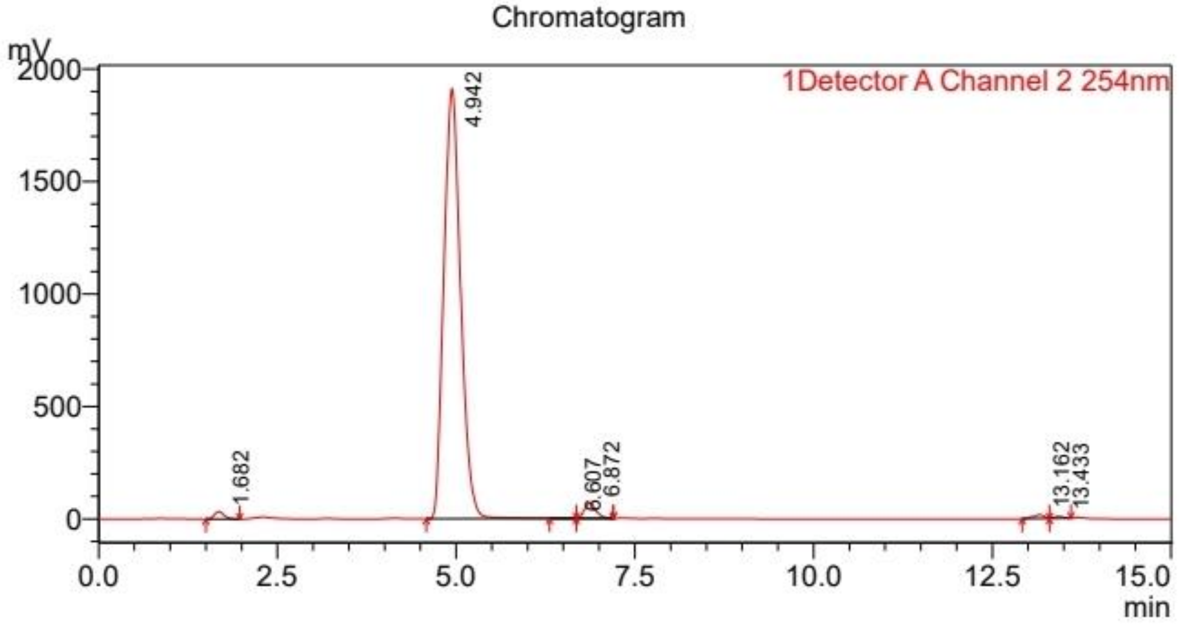

Detector A Channel 2 254nm

Peak#	Ret. Time (min)	Area%
1	1.682	0.895
2	4.942	95.906
3	6.607	0.035
4	6.872	2.337
5	13.162	0.587
6	13.433	0.240
Total		100.000

3-(2-(4-cyanophenoxy)ethyl)pyridine 1-oxide (22)

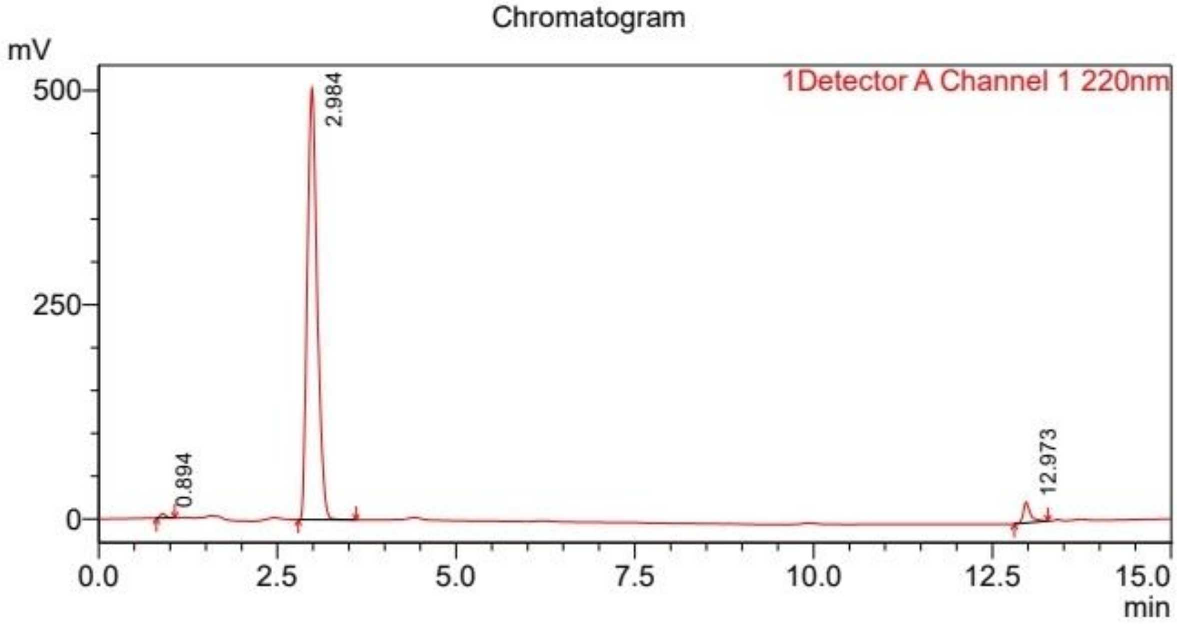

Detector A Channel 1 220nm

Peak#	Ret. Time (min)	Area%
1	0.894	0.553
2	2.984	96.062
3	12.973	3.385
Total		100.000

3-(2-(4-nitrophenoxy)ethyl)pyridine 1-oxide (23)

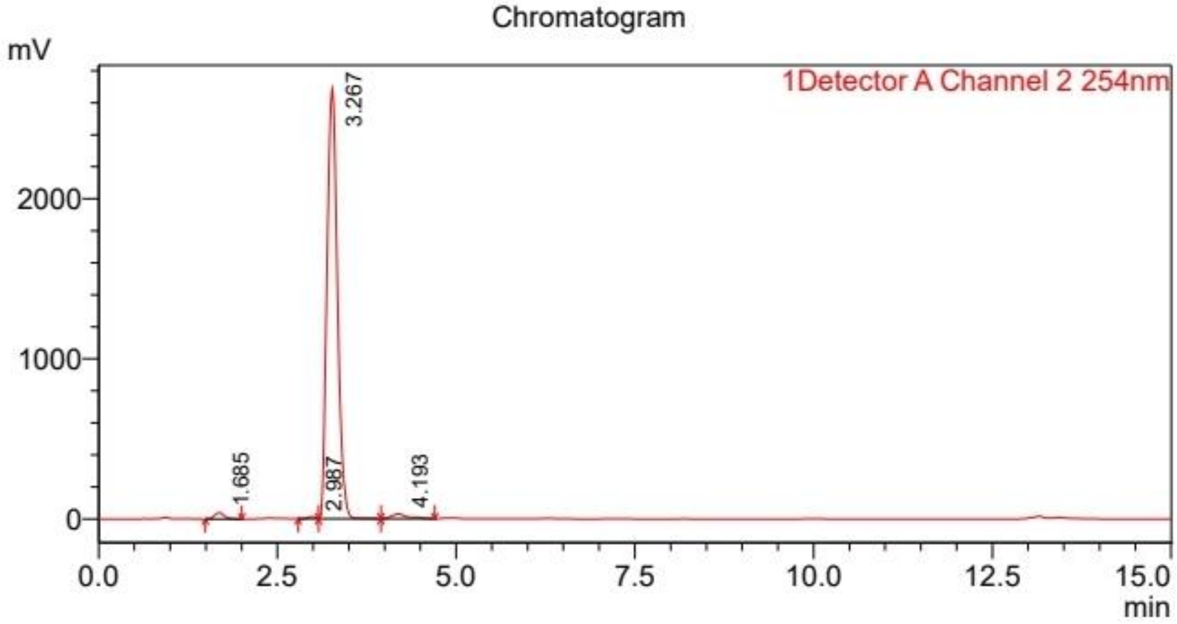

Detector A Channel 2 254nm

Peak#	Ret. Time (min)	Area%
1	1.685	1.251
2	2.987	0.389
3	3.267	96.647
4	4.193	1.713
Total		100.000

3-(2-(4-chloro-2,3-dimethylphenoxy)ethyl)pyridine 1-oxide (24)

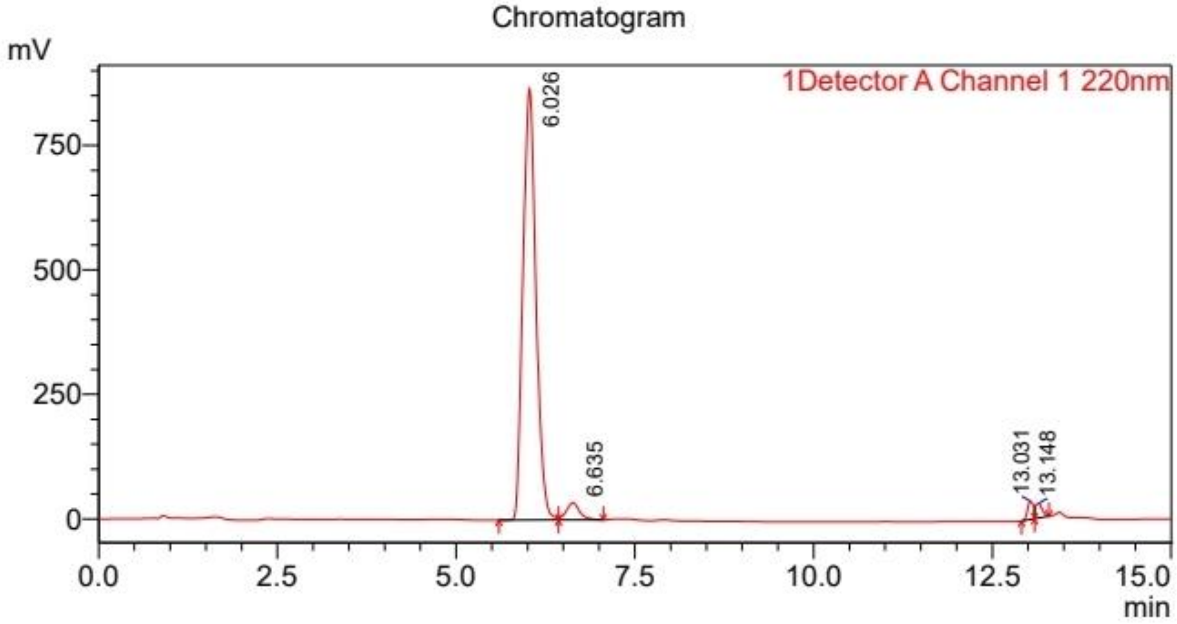

Detector A Channel 1 220nm

Peak#	Ret. Time (min)	Area%
1	6.026	92.735
2	6.635	3.816
3	13.031	1.832
4	13.148	1.617
Total		100.000

3-(2-(2,3-dichlorophenoxy)ethyl)pyridine 1-oxide (25)

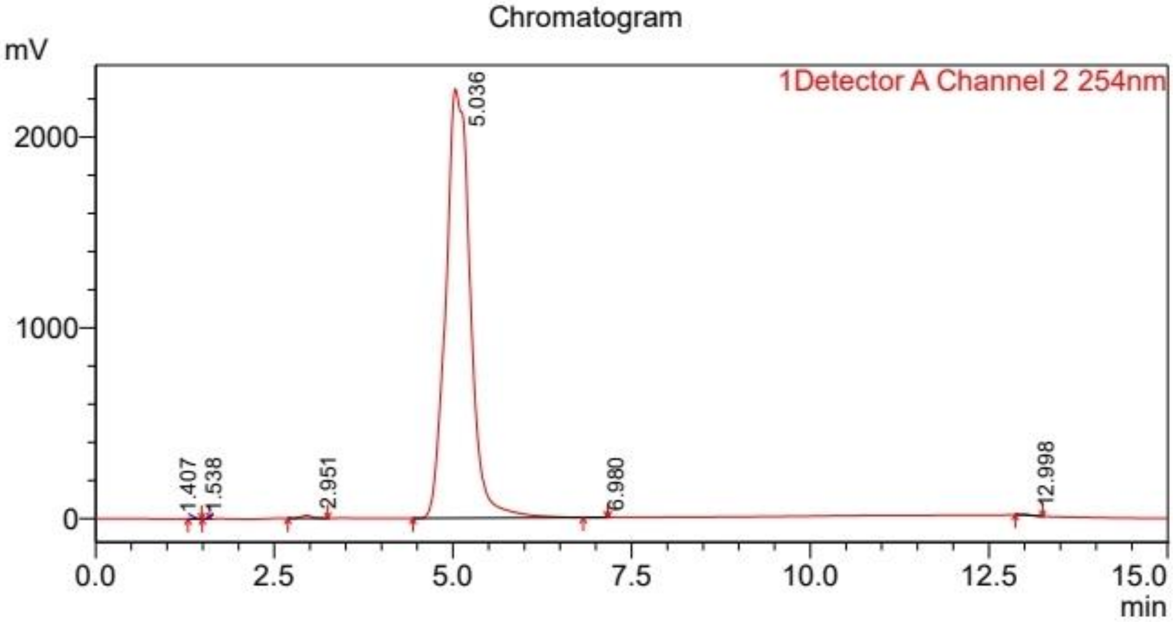

Detector A Channel 2 254nm

Peak#	Ret. Time (min)	Area%
1	1.407	0.031
2	1.538	0.004
3	2.951	0.299
4	5.036	99.574
5	6.980	0.004
6	12.998	0.087
Total		100.000

3-(2-(4-chloro-2-iodophenoxy)ethyl)pyridine 1-oxide (26)

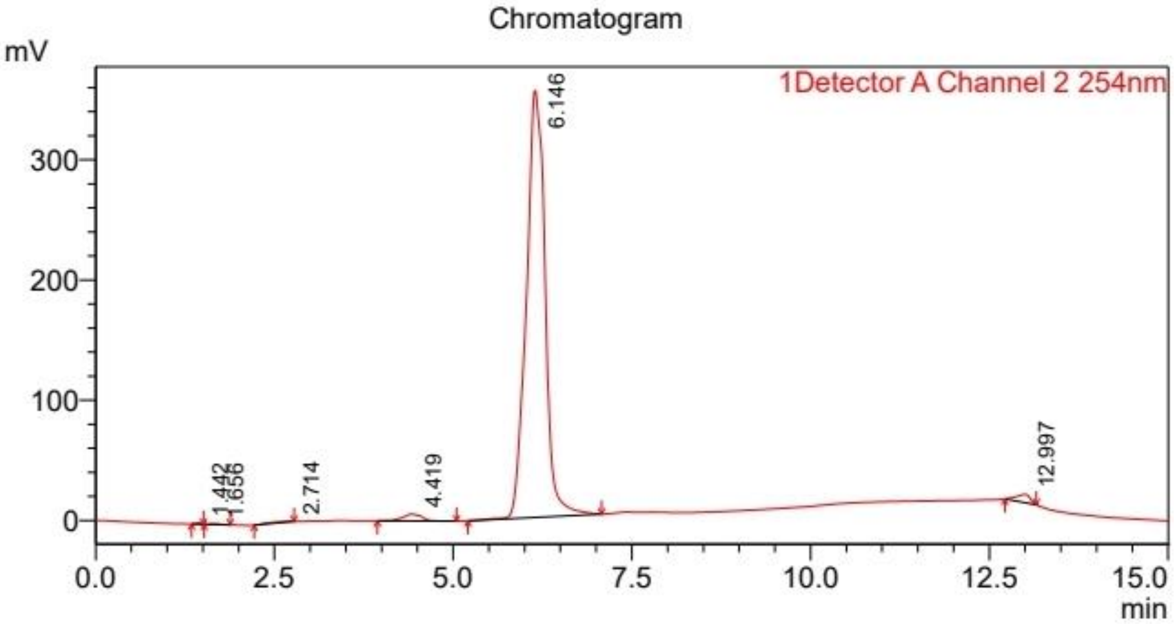

Detector A Channel 2 254nm

Peak#	Ret. Time (min)	Area%
1	1.442	0.078
2	1.656	0.122
3	2.714	0.318
4	4.419	1.520
5	6.146	96.619
6	12.997	1.343
Total		100.000

3-(2-(4-chloro-2-cyclohexylphenoxy)ethyl)pyridine 1-oxide (27)

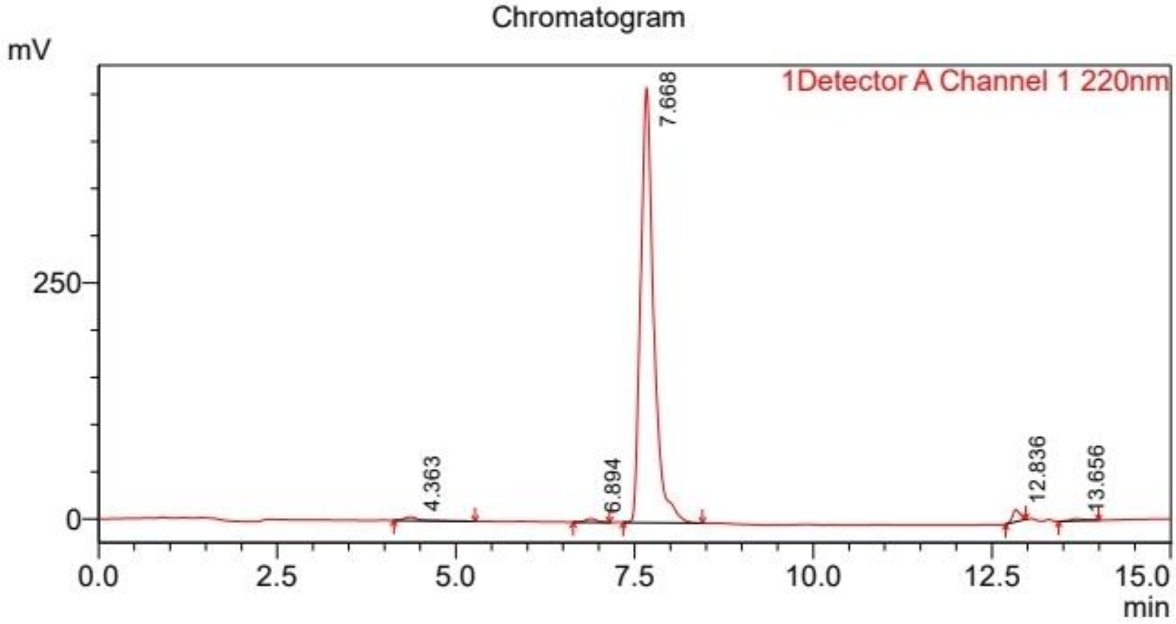

Detector A Channel 1 220nm

Peak#	Ret. Time (min)	Area%
1	4.363	0.882
2	6.894	0.687
3	7.668	96.463
4	12.836	1.495
5	13.656	0.473
Total		100.000

3-(2-((5-chloro-[1,1'-biphenyl]-2-yl)oxy)ethyl)pyridine 1-oxide (28)

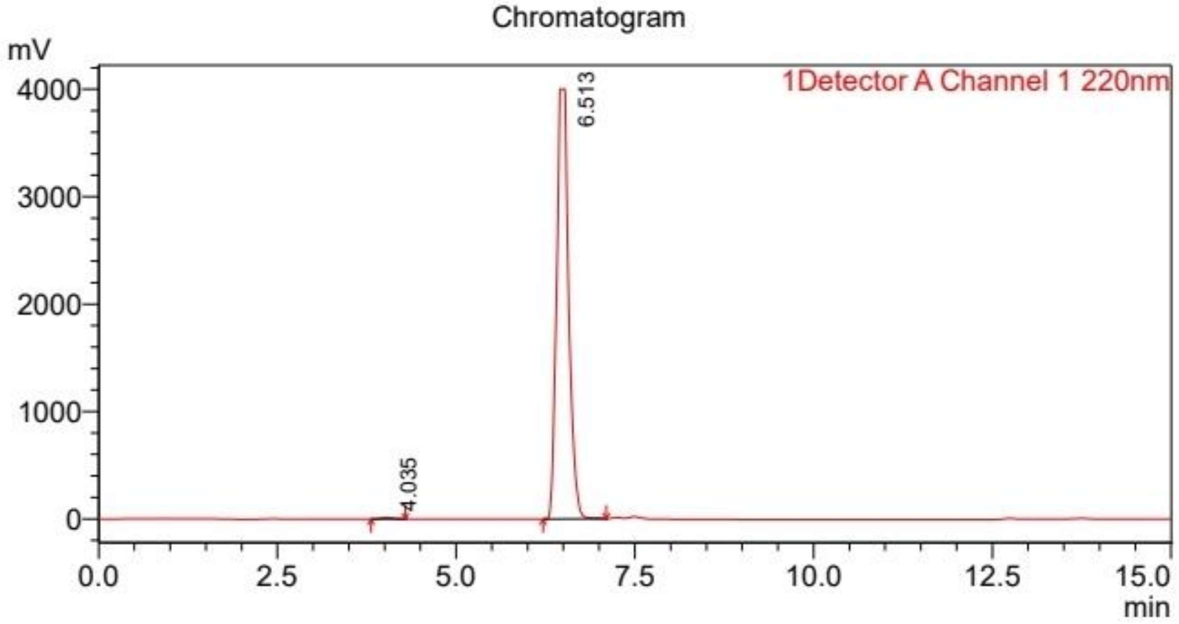

Detector A Channel 1 220nm

Peak#	Ret. Time (min)	Area%
1	4.035	0.319
2	6.513	99.681
Total		100.000

3-(2-(2,3,4-trichlorophenoxy)ethyl)pyridine 1-oxide (29)

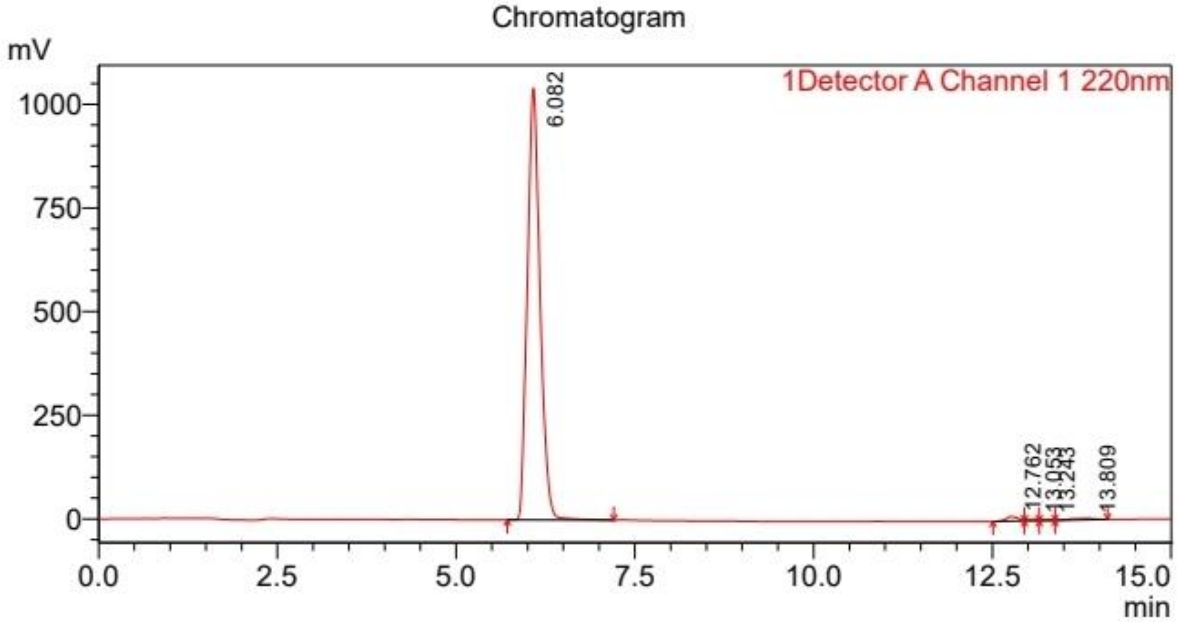

Detector A Channel 1 220nm

Peak#	Ret. Time (min)	Area%
1	6.082	97.786
2	12.762	1.006
3	13.053	0.345
4	13.243	0.220
5	13.809	0.643
Total		100.000

3-(2-(4-chloro-3,5-dimethylphenoxy)ethyl)pyridine 1-oxide (30)

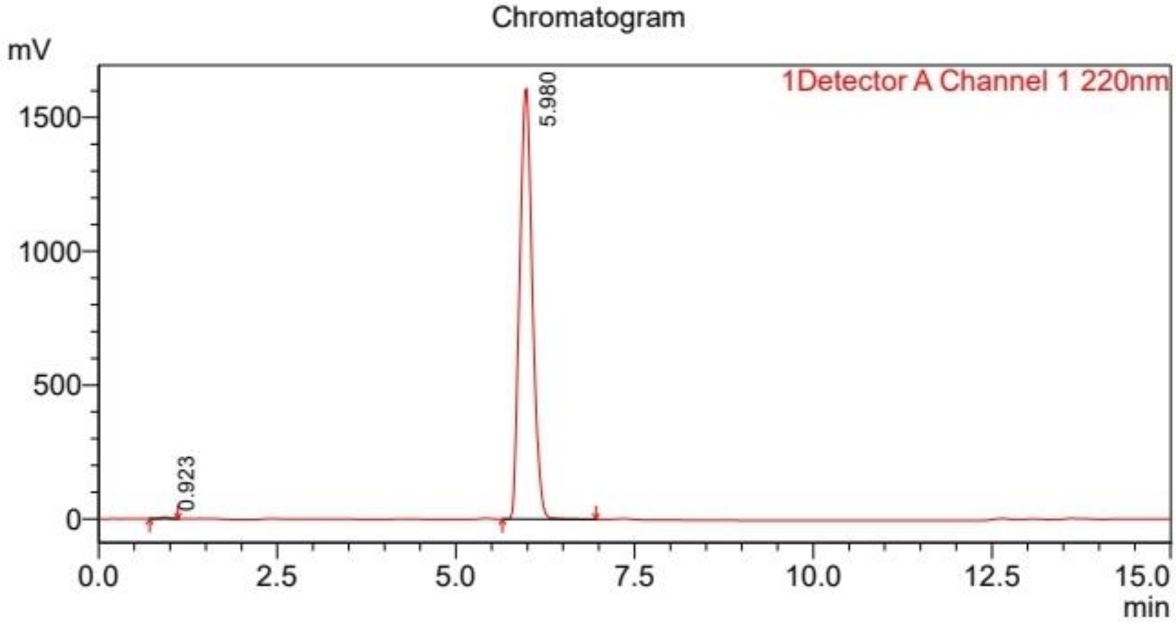

Detector A Channel 1 220nm

Peak#	Ret. Time (min)	Area%
1	0.923	0.219
2	5.980	99.781
Total		100.000

3-(2-(3,4,5-trimethylphenoxy)ethyl)pyridine 1-oxide (31)

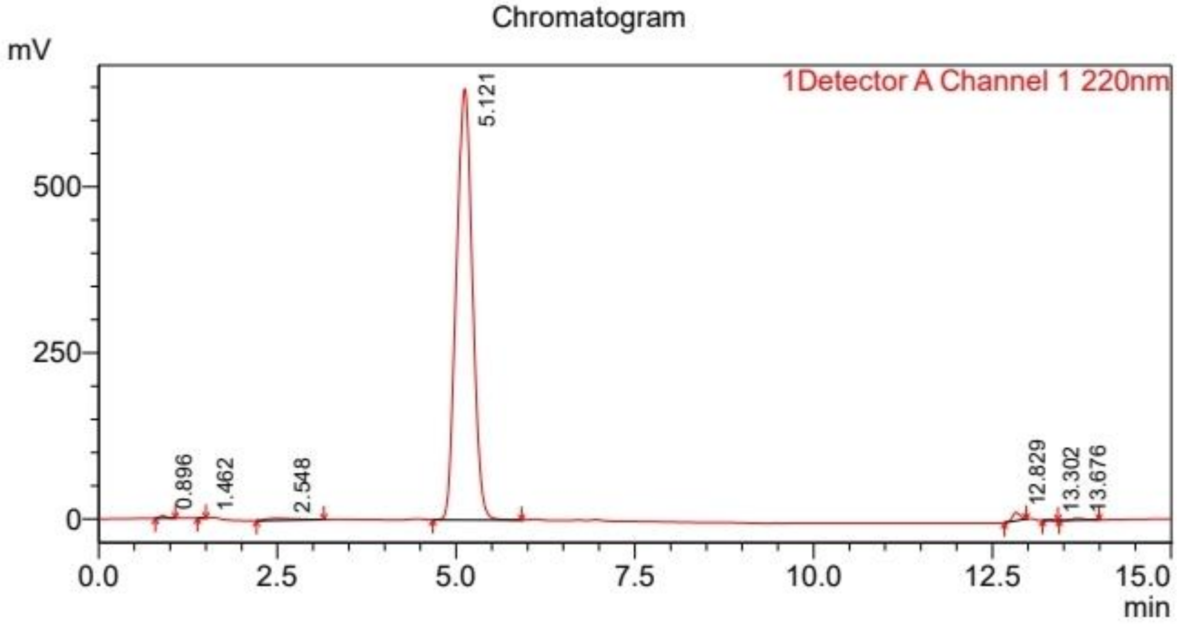

Detector A Channel 1 220nm

Peak#	Ret. Time (min)	Area%
1	0.896	0.246
2	1.462	0.006
3	2.548	0.856
4	5.121	97.606
5	12.829	0.866
6	13.302	0.087
7	13.676	0.333
Total		100.000

3-(2-(4-chloro-3-iodophenoxy)ethyl)pyridine 1-oxide (32)

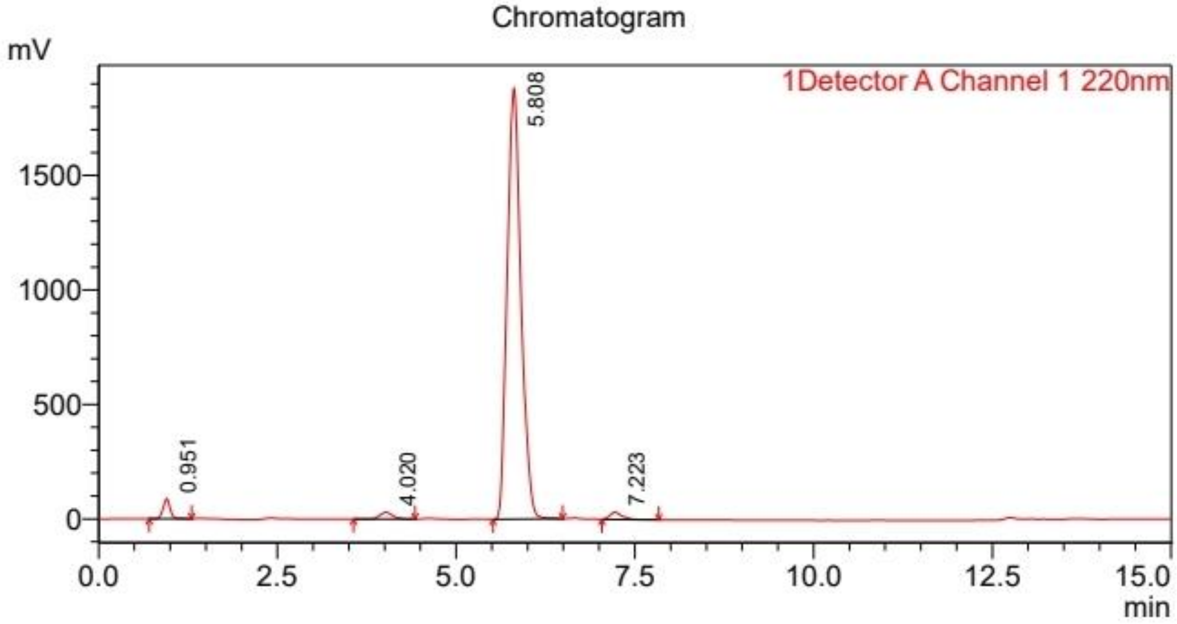

Detector A Channel 1 220nm

Peak#	Ret. Time (min)	Area%
1	0.951	2.204
2	4.020	1.363
3	5.808	95.107
4	7.223	1.326
Total		100.000

3-(2-(3-bromo-4-chlorophenoxy)ethyl)pyridine 1-oxide (33)

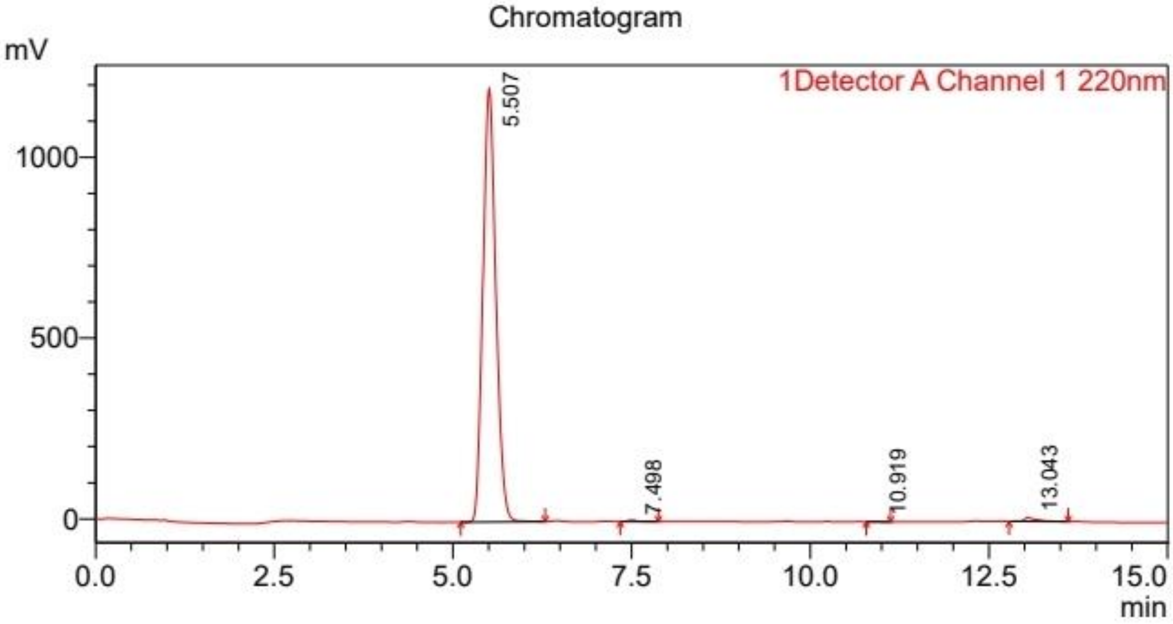

Detector A Channel 1 220nm

Peak#	Ret. Time (min)	Area%
1	5.507	98.858
2	7.498	0.250
3	10.919	0.072
4	13.043	0.819
Total		100.000

3-(2-(3,4-dichlorophenoxy)ethyl)pyridine 1-oxide (34)

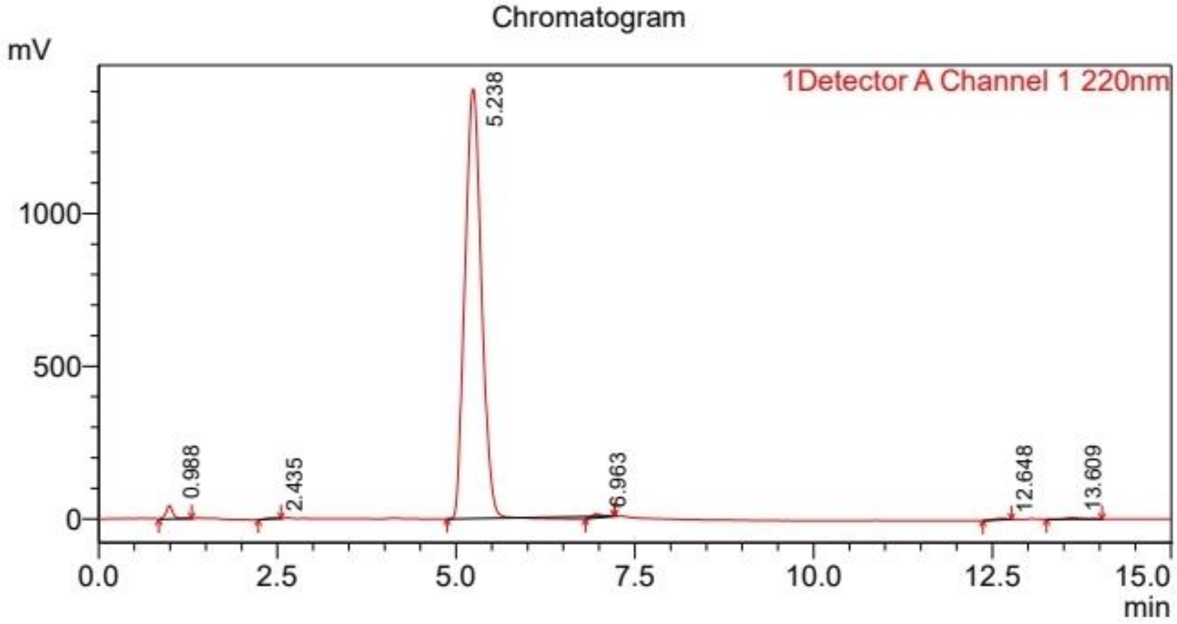

Detector A Channel 1 220nm

Peak#	Ret. Time (min)	Area%
1	0.988	1.275
2	2.435	0.132
3	5.238	97.381
4	6.963	0.655
5	12.648	0.249
6	13.609	0.309
Total		100.000

3-(2-(4-chloro-3-fluorophenoxy)ethyl)pyridine 1-oxide (35)

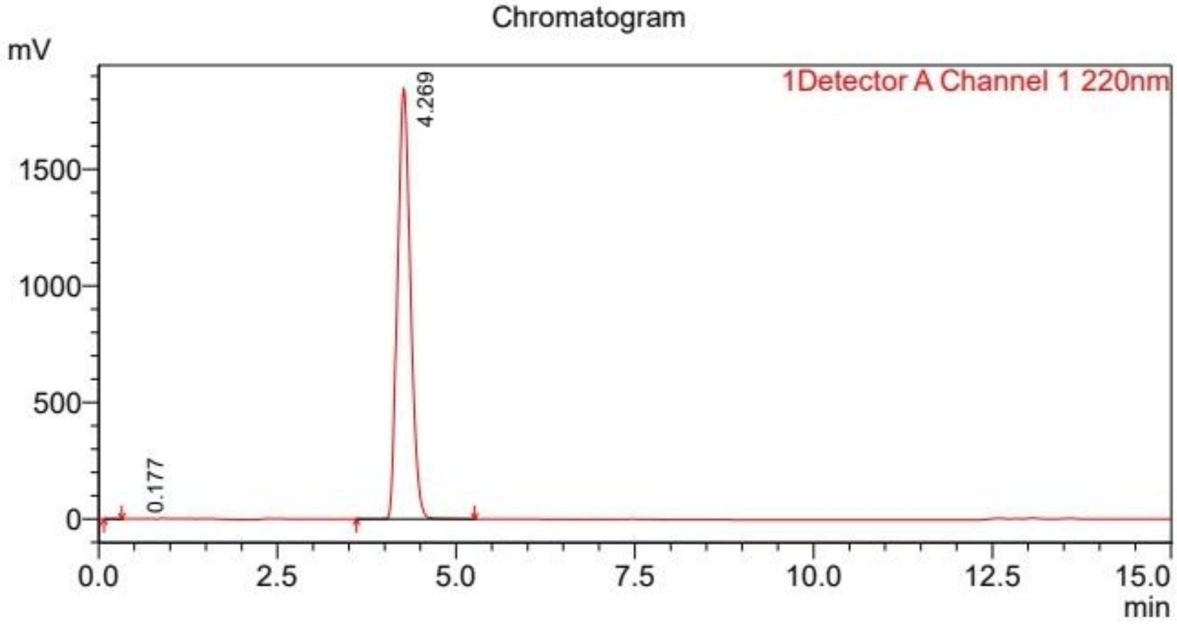

Detector A Channel 1 220nm

Peak#	Ret. Time (min)	Area%
1	0.177	0.005
2	4.269	99.995
Total		100.000

3-(2-((6-chloro-[1,1'-biphenyl]-3-yl)oxy)ethyl)pyridine 1-oxide (36)

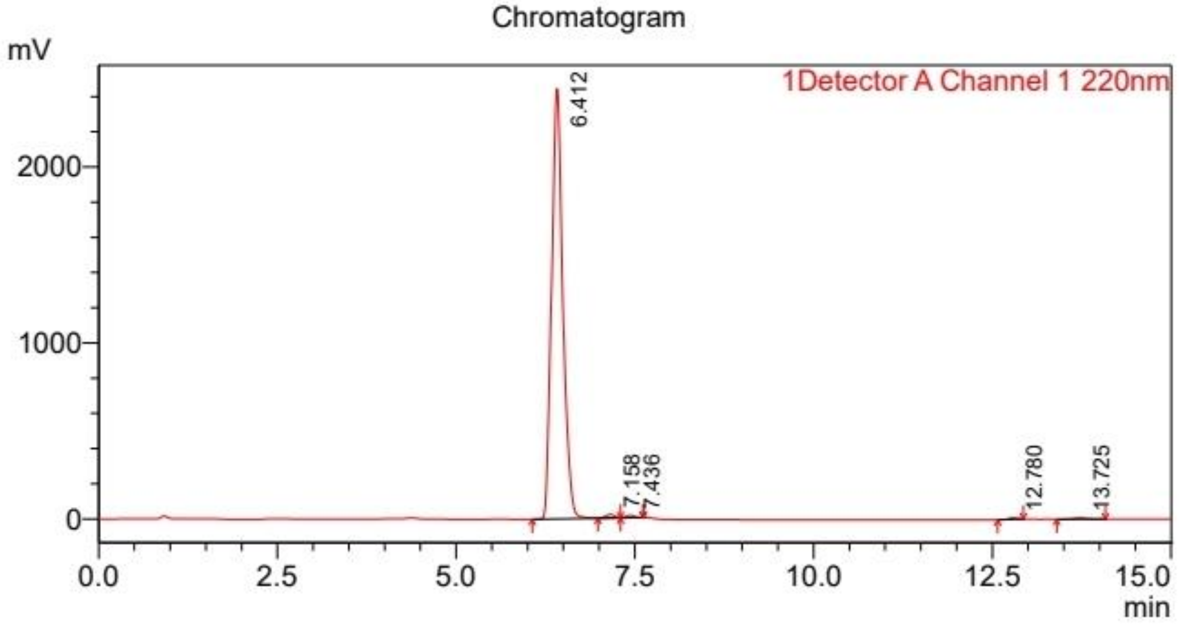

Detector A Channel 1 220nm

Peak#	Ret. Time (min)	Area%
1	6.412	98.311
2	7.158	0.657
3	7.436	0.422
4	12.780	0.292
5	13.725	0.318
Total		100.000

3-(2-(4-chloro-3-methylphenoxy)ethyl)pyridine 1-oxide (37)

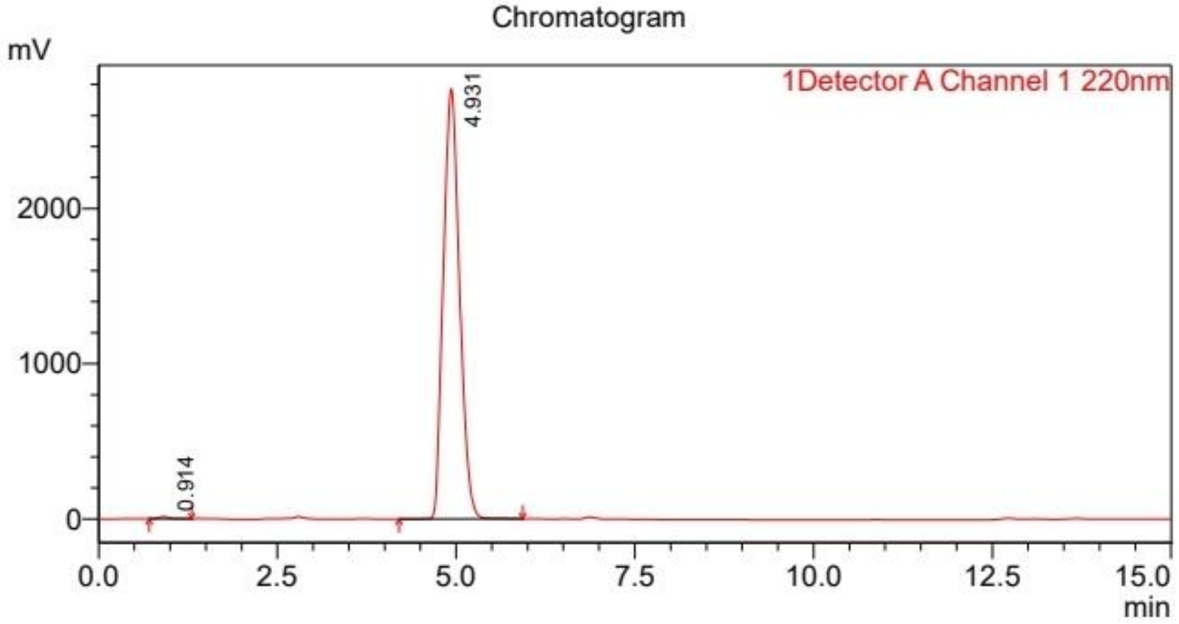

Detector A Channel 1 220nm

Peak#	Ret. Time (min)	Area%
1	0.914	0.267
2	4.931	99.733
Total		100.000

3-(2-(4-chloro-3-nitrophenoxy)ethyl)pyridine 1-oxide (38)

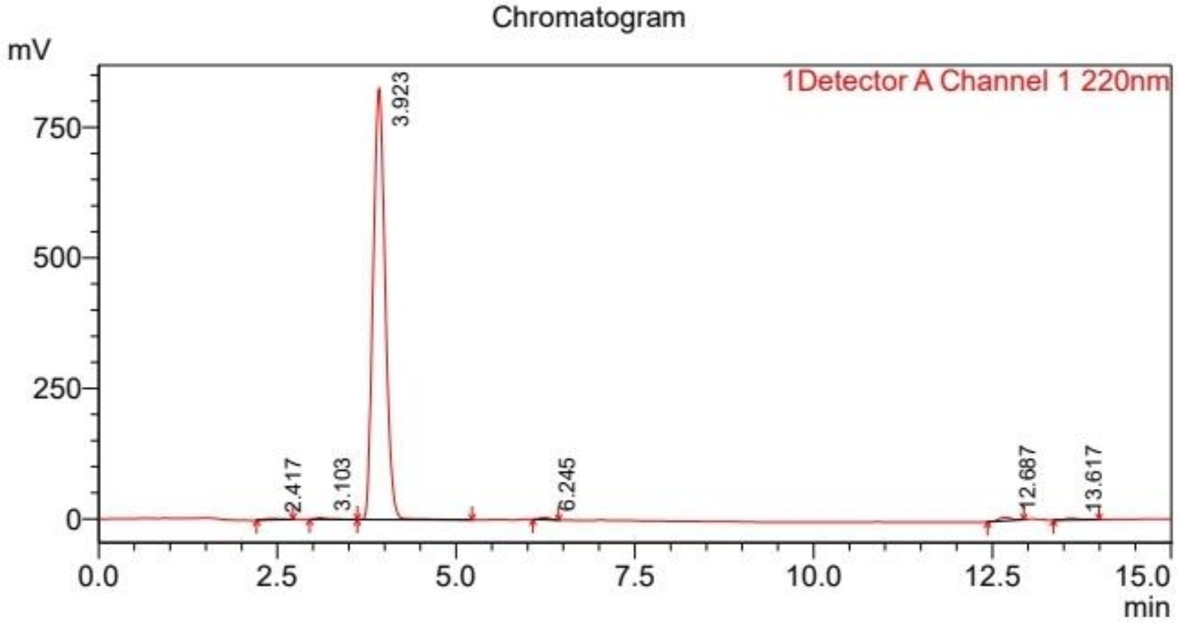

Detector A Channel 1 220nm

Peak#	Ret. Time (min)	Area%
1	2.417	0.372
2	3.103	0.384
3	3.923	97.618
4	6.245	0.391
5	12.687	0.842
6	13.617	0.394
Total		100.000

3-(2-(4-chloro-3-ethylphenoxy)ethyl)pyridine 1-oxide (39)

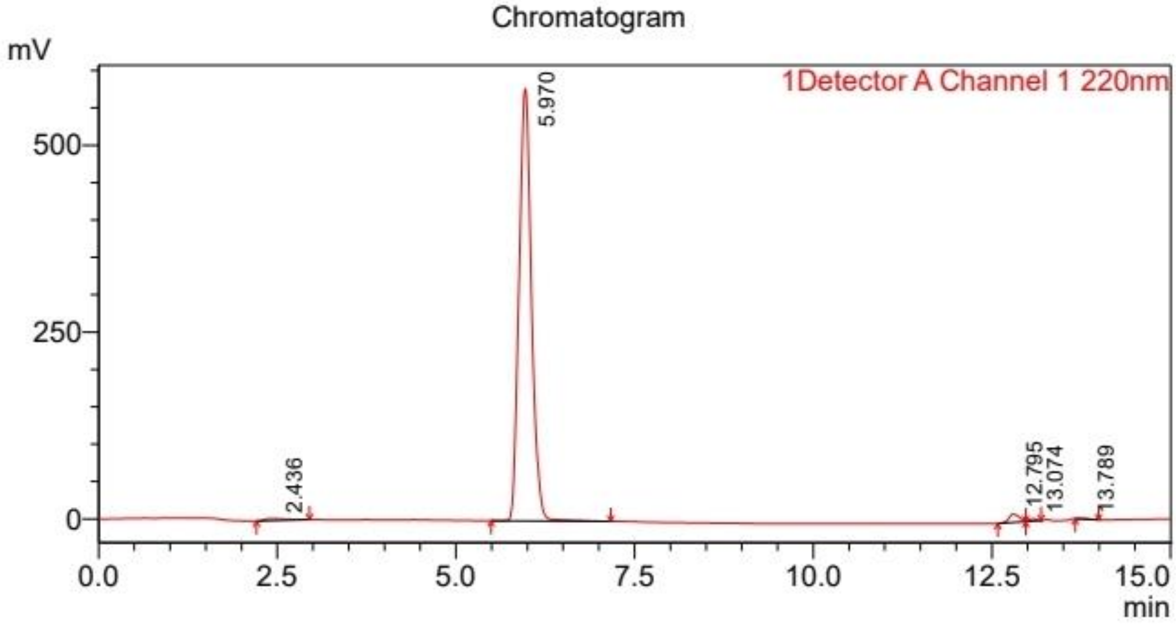

Detector A Channel 1 220nm

Peak#	Ret. Time (min)	Area%
1	2.436	0.786
2	5.970	97.135
3	12.795	1.612
4	13.074	0.352
5	13.789	0.115
Total		100.000

3-(2-(4-chloro-3-methoxyphenoxy)ethyl)pyridine 1-oxide (40)

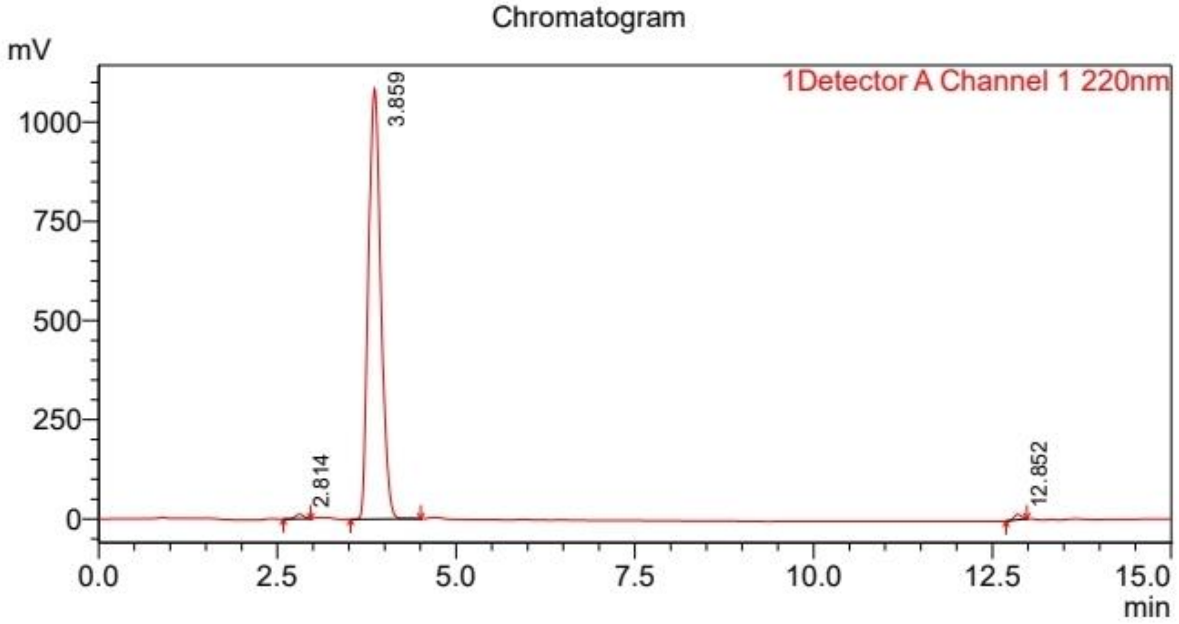

Detector A Channel 1 220nm

Peak#	Ret. Time (min)	Area%
1	2.814	0.663
2	3.859	98.598
3	12.852	0.739
Total		100.000

3-(2-(4-chloro-3-cyclopropylphenoxy)ethyl)pyridine 1-oxide (41)

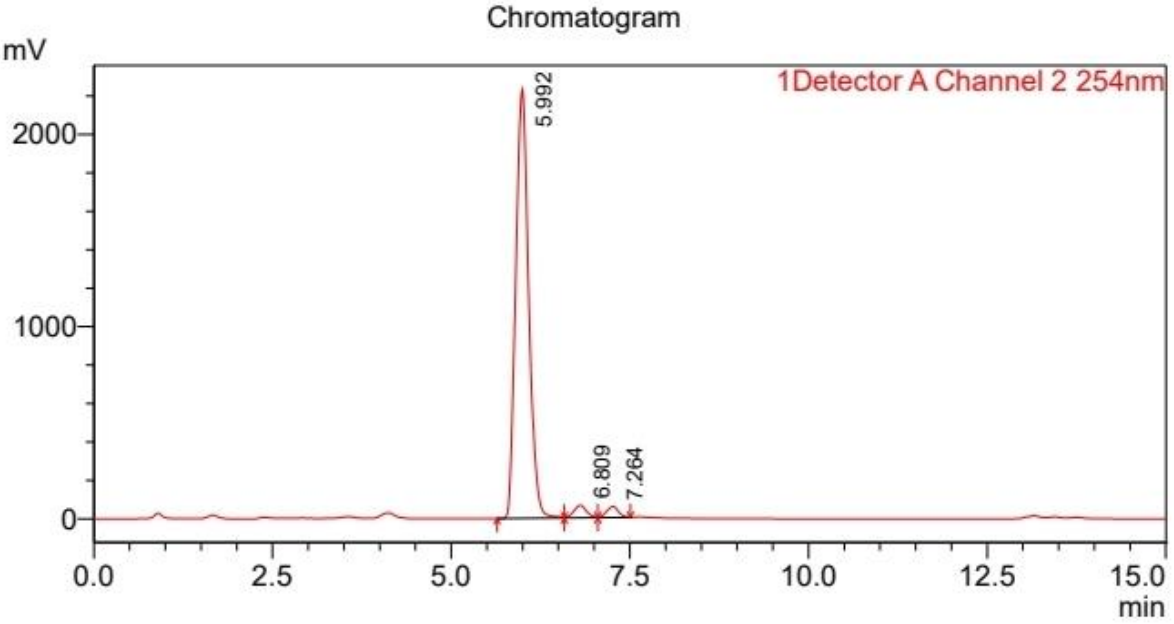

Detector A Channel 2 254nm

Peak#	Ret. Time (min)	Area%
1	5.992	95.032
2	6.809	2.741
3	7.264	2.227
Total		100.000

3-(2-(4-chloro-3-(trifluoromethyl)phenoxy)ethyl)pyridine 1-oxide (42)

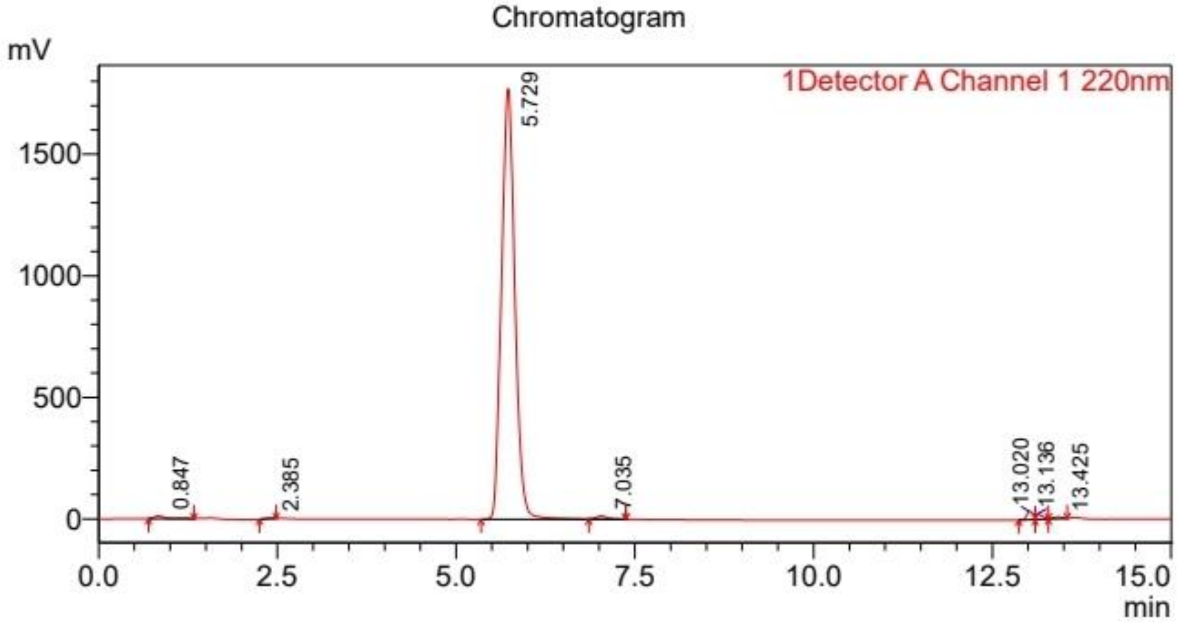

Detector A Channel 1 220nm

Peak#	Ret. Time (min)	Area%
1	0.847	0.438
2	2.385	0.102
3	5.729	97.051
4	7.035	0.569
5	13.020	0.947
6	13.136	0.614
7	13.425	0.279
Total		100.000

3-(2-(3,5-bis(trifluoromethyl)phenoxy)ethyl)pyridine 1-oxide (43)

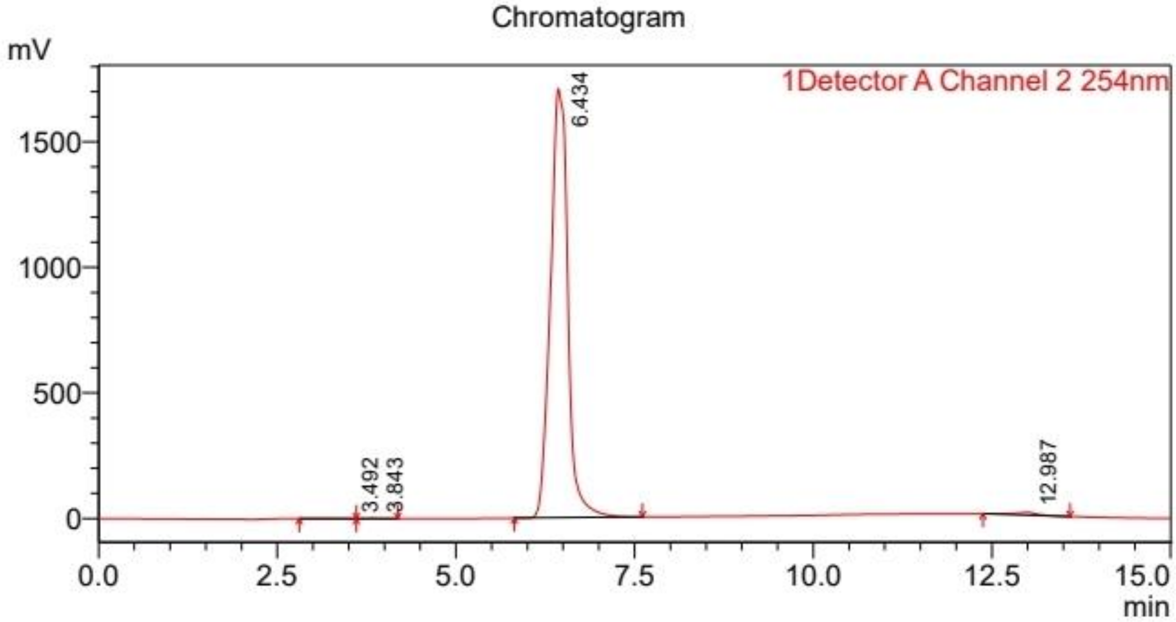

Detector A Channel 2 254nm

Peak#	Ret. Time (min)	Area%
1	3.492	0.027
2	3.843	0.029
3	6.434	99.081
4	12.987	0.863
Total		100.000

3-(((4-chloronaphthalen-1-yl)oxy)methyl)pyridine 1-oxide (44)

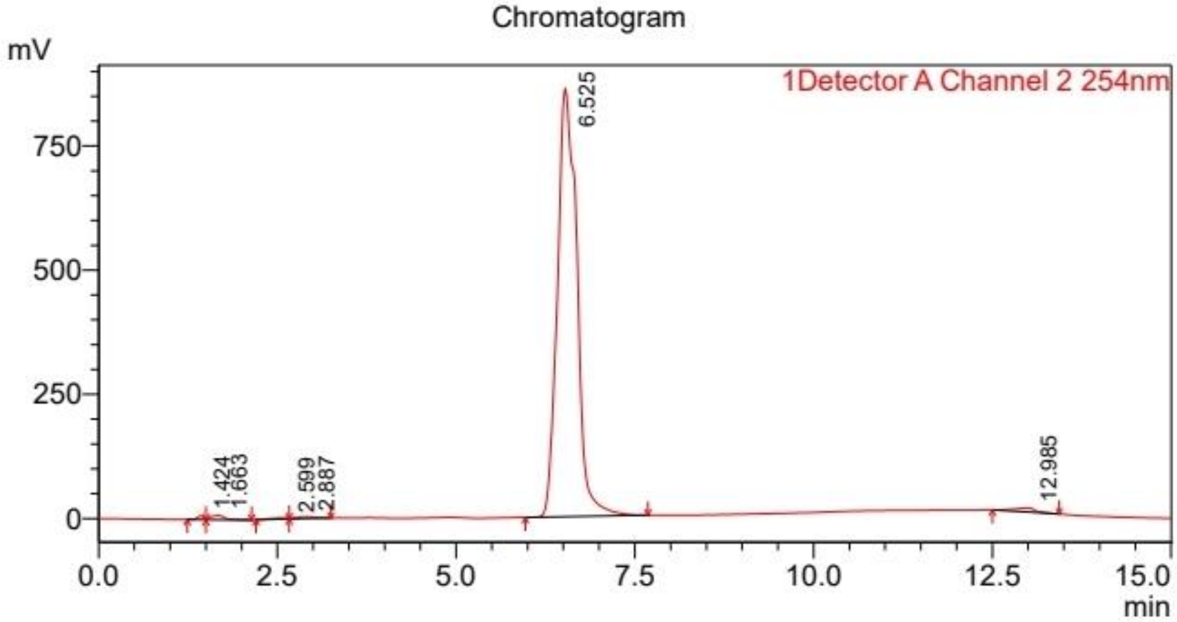

Detector A Channel 2 254nm

Peak#	Ret. Time (min)	Area%
1	1.424	0.245
2	1.663	0.689
3	2.599	0.272
4	2.887	0.423
5	6.525	97.334
6	12.985	1.037
Total		100.000

3-(3-((4-chloronaphthalen-1-yl)oxy)propyl)pyridine 1-oxide (45)

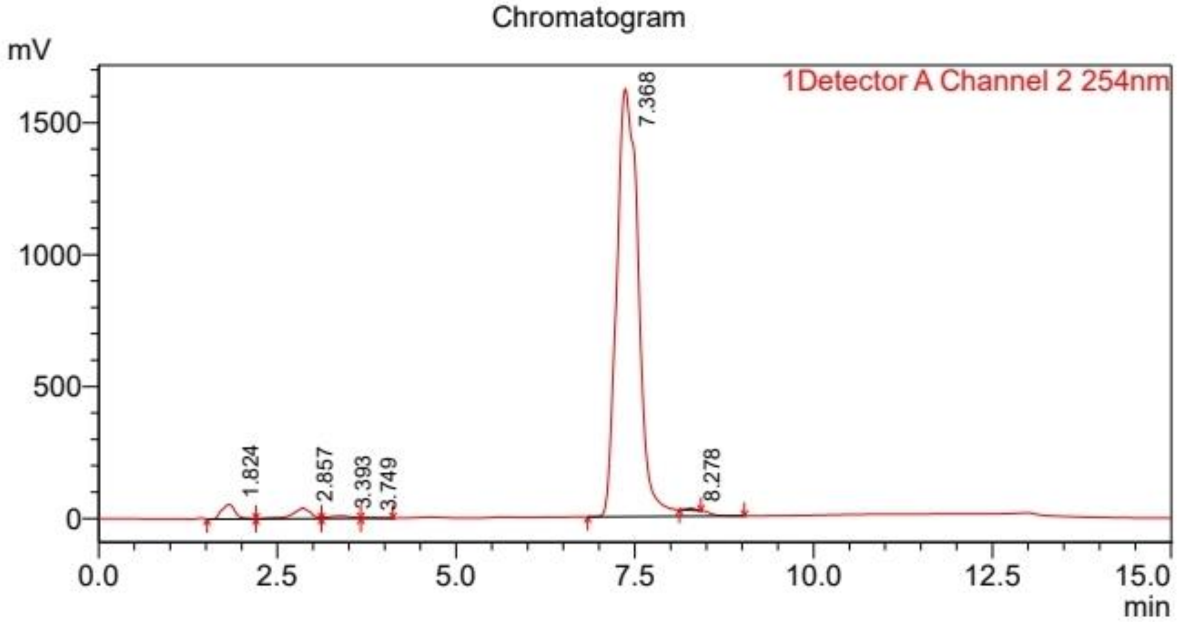

Detector A Channel 2 254nm

Peak#	Ret. Time (min)	Area%
1	1.824	1.980
2	2.857	1.929
3	3.393	0.566
4	3.749	0.093
5	7.368	95.247
6	8.278	0.186
Total		100.000

3-(3-(naphthalen-1-yl)propyl)pyridine 1-oxide (46)

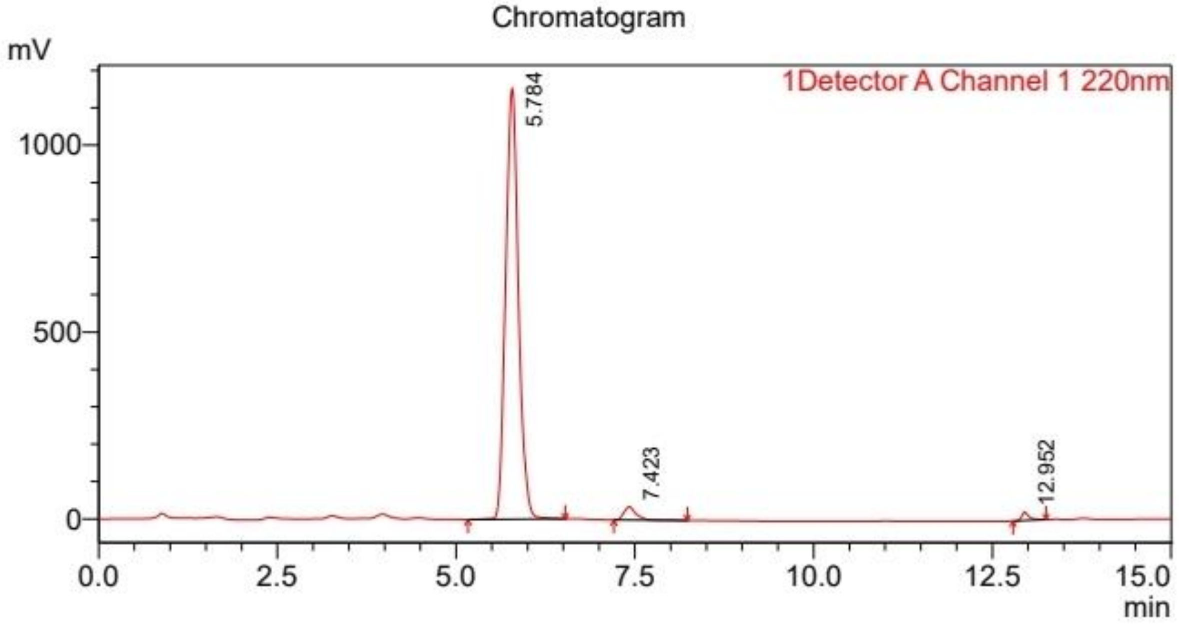

Detector A Channel 1 220nm

Peak#	Ret. Time (min)	Area%
1	5.784	96.115
2	7.423	2.741
3	12.952	1.144
Total		100.000

3-(3-(naphthalen-1-yl)propanoyl)pyridine 1-oxide (47)

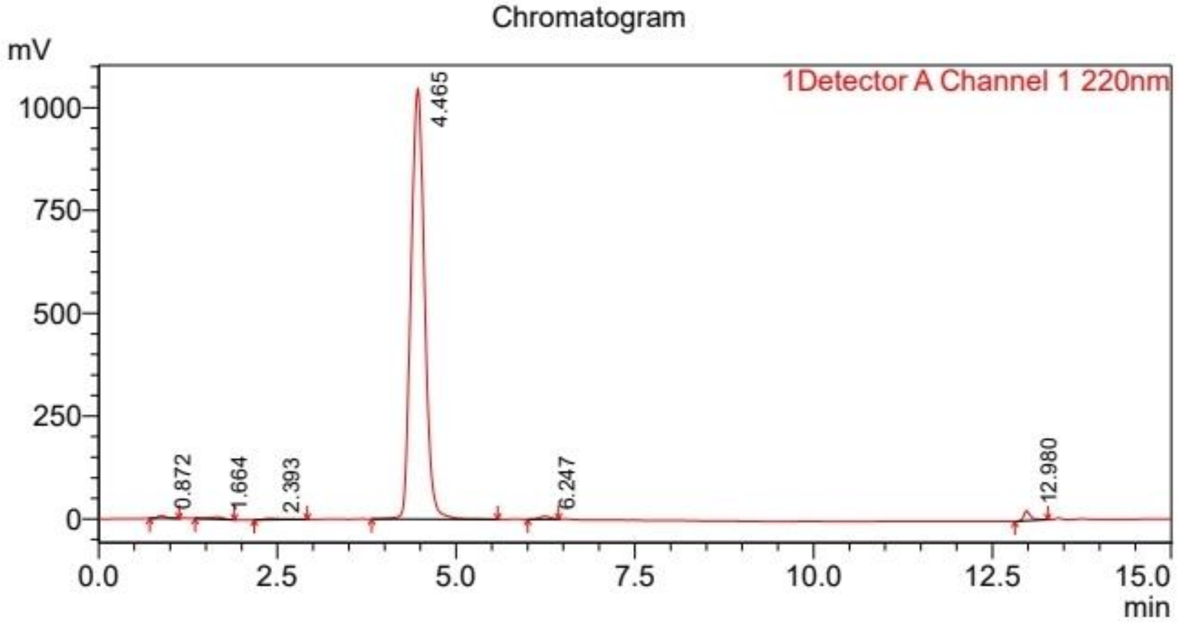

Detector A Channel 1 220nm

Peak#	Ret. Time (min)	Area%
1	0.872	0.412
2	1.664	0.472
3	2.393	0.410
4	4.465	97.050
5	6.247	0.481
6	12.980	1.175
Total		100.000

3-(4-chlorophenethoxy)pyridine 1-oxide (48)

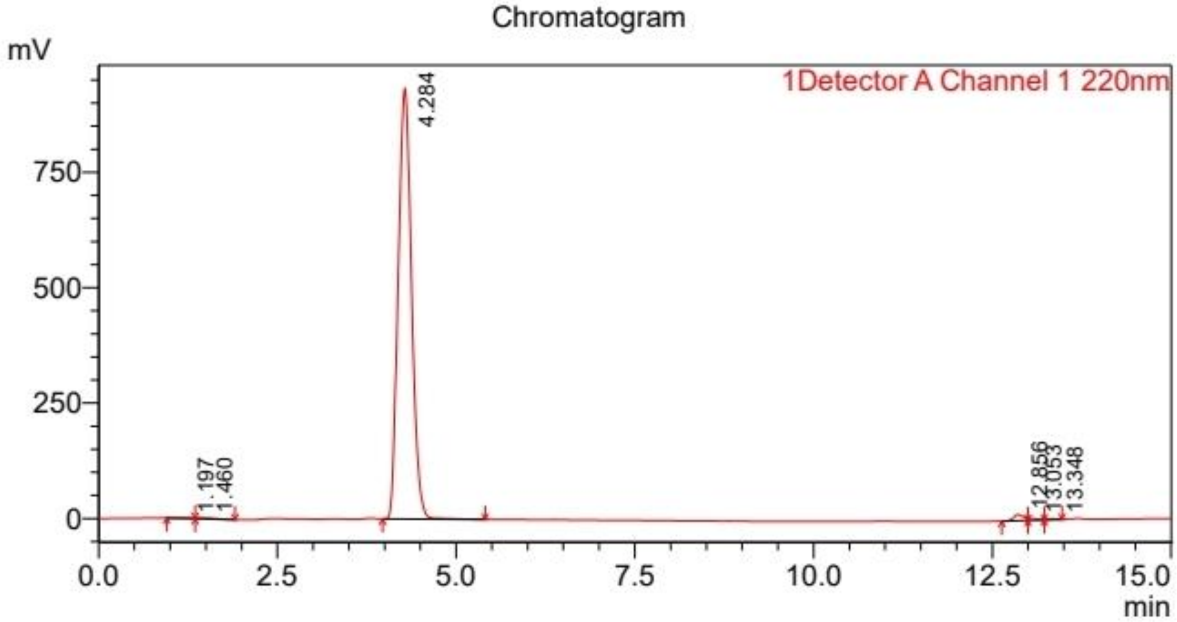

Detector A Channel 1 220nm

Peak#	Ret. Time (min)	Area%
1	1.197	0.171
2	1.460	0.312
3	4.284	98.031
4	12.856	1.066
5	13.053	0.289
6	13.348	0.131
Total		100.000

3-(3-(4-chlorophenyl)propyl)pyridine 1-oxide (49)

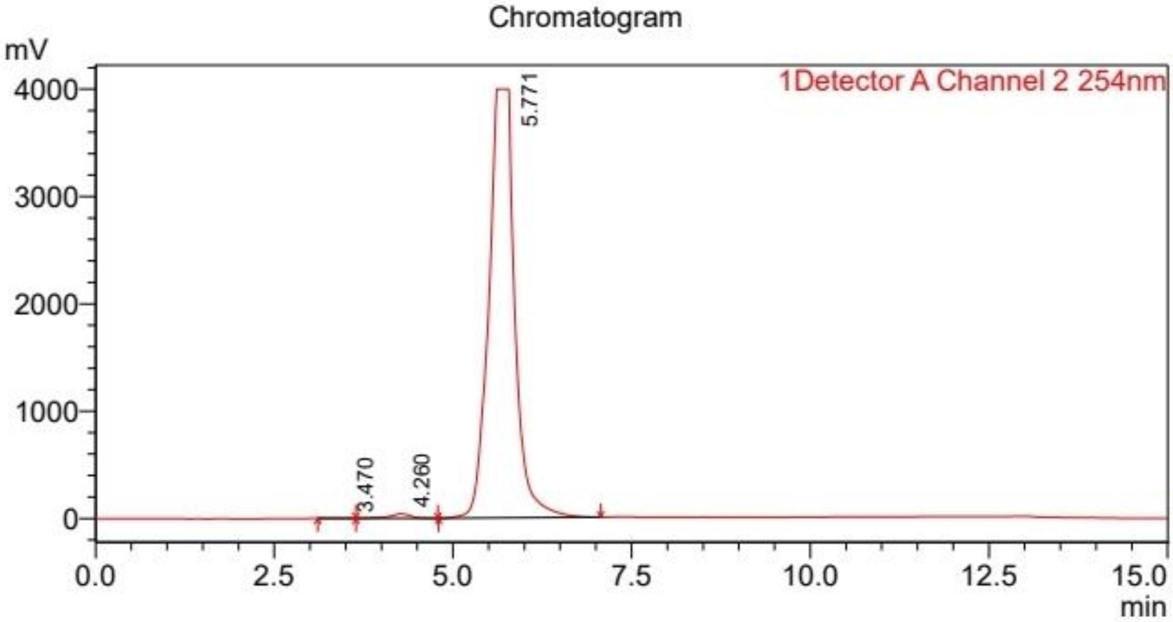

Detector A Channel 2 254nm

Peak#	Ret. Time (min)	Area%
1	3.470	0.014
2	4.260	0.841
3	5.771	99.145
Total		100.000

3-(3-(4-chlorophenyl)propanoyl)pyridine 1-oxide (50)

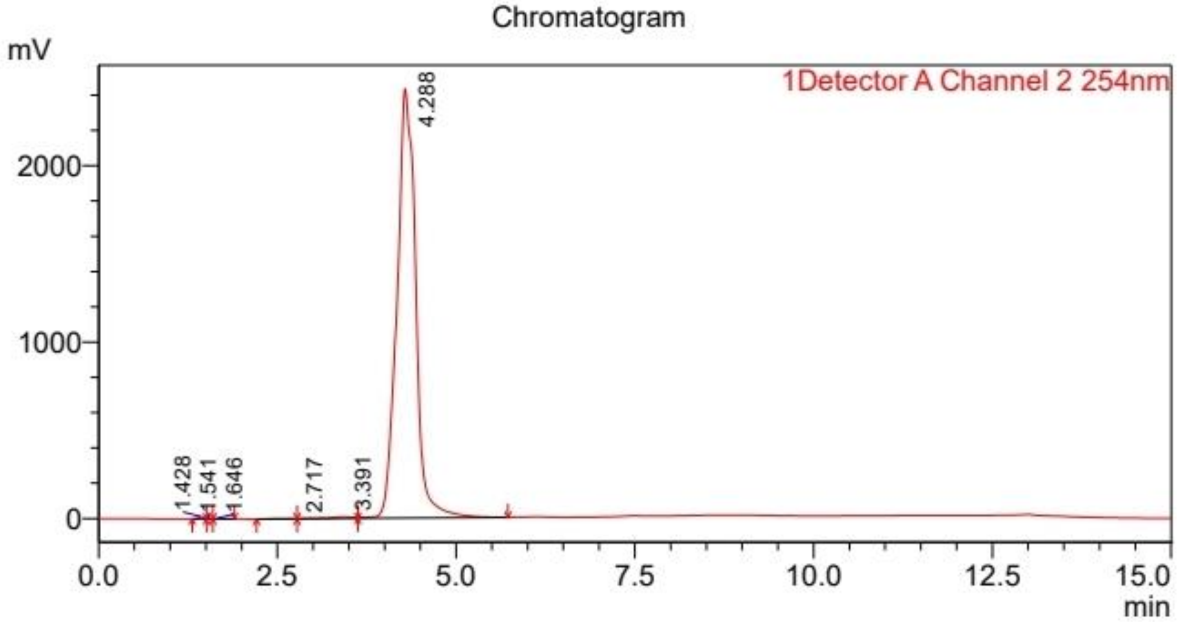

Detector A Channel 2 254nm

Peak#	Ret. Time (min)	Area%
1	1.428	0.160
2	1.541	0.021
3	1.646	0.023
4	2.717	0.120
5	3.391	0.507
6	4.288	99.169
Total		100.000

3-(2-((4-chlorophenyl)sulfonyl)ethyl)pyridine 1-oxide (51)

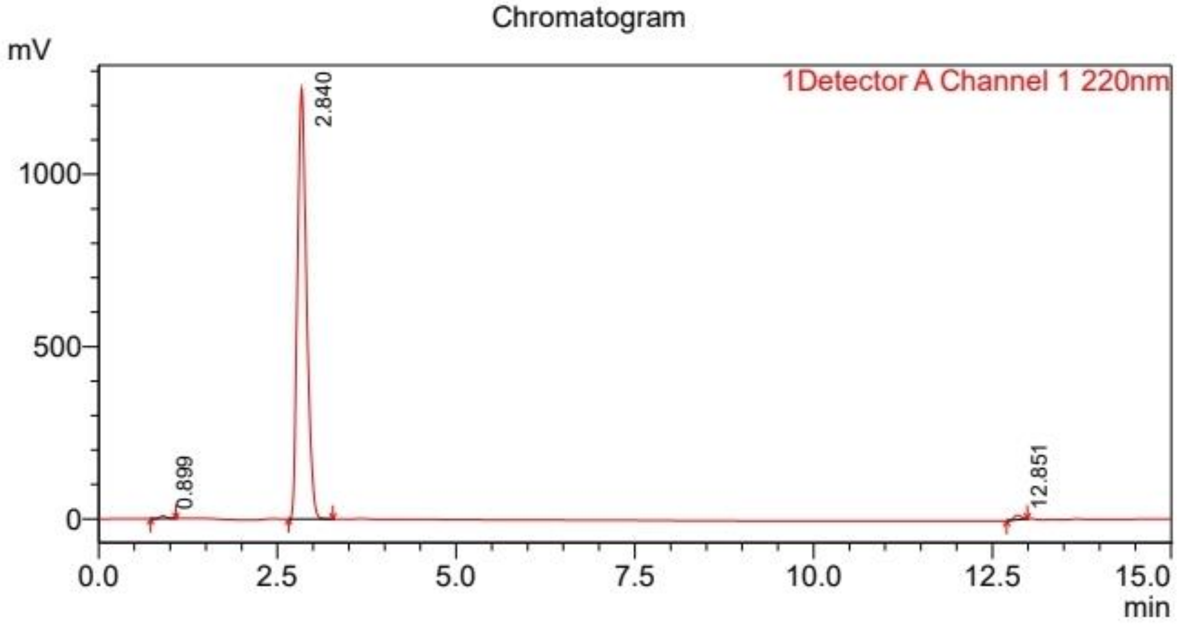

Detector A Channel 1 220nm

Peak#	Ret. Time (min)	Area%
1	0.899	0.433
2	2.840	98.702
3	12.851	0.865
Total		100.000

3-(3-(4-chlorophenyl)-3-fluoropropyl)pyridine 1-oxide (52)

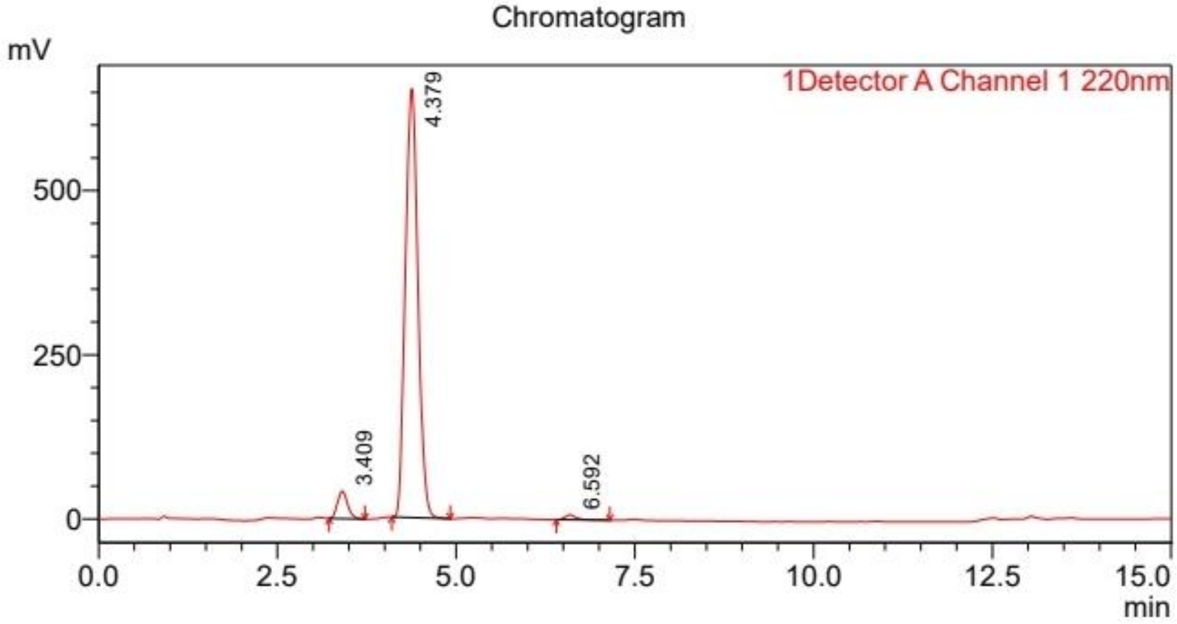

Detector A Channel 1 220nm

Peak#	Ret. Time (min)	Area%
1	3.409	4.861
2	4.379	94.189
3	6.592	0.950
Total		100.000

3-(3-(4-chlorophenyl)-3-hydroxypropyl)pyridine 1-oxide (53)

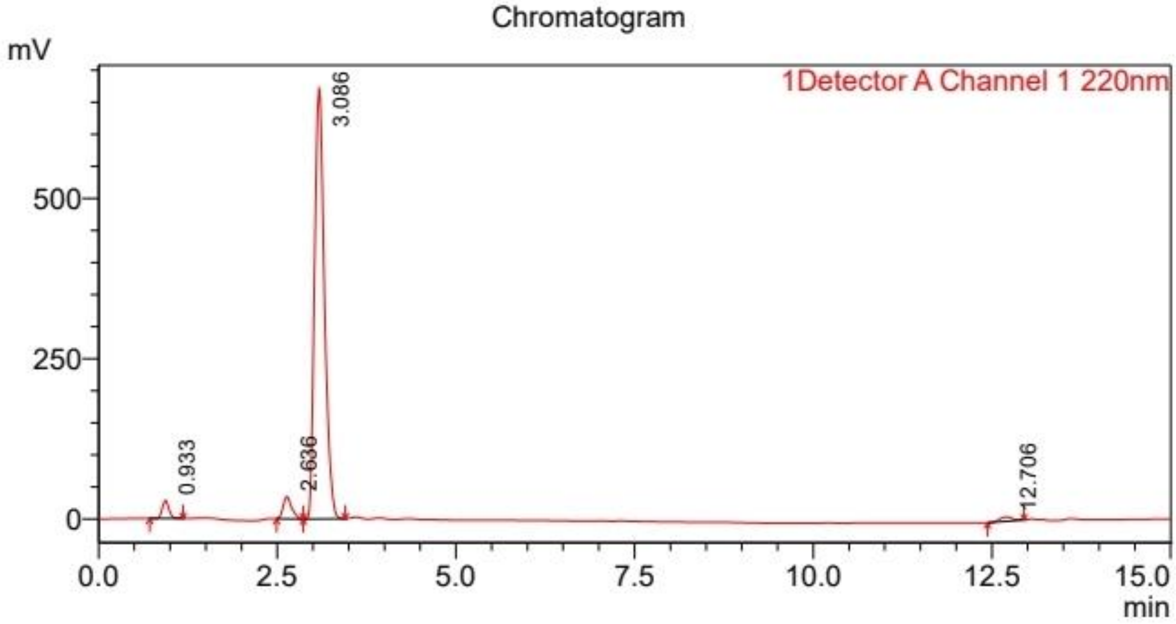

Detector A Channel 1 220nm

Peak#	Ret. Time (min)	Area%
1	0.933	2.572
2	2.636	4.124
3	3.086	92.096
4	12.706	1.208
Total		100.000

3-(3-(4-chlorophenyl)-3-oxopropyl)pyridine 1-oxide (54)

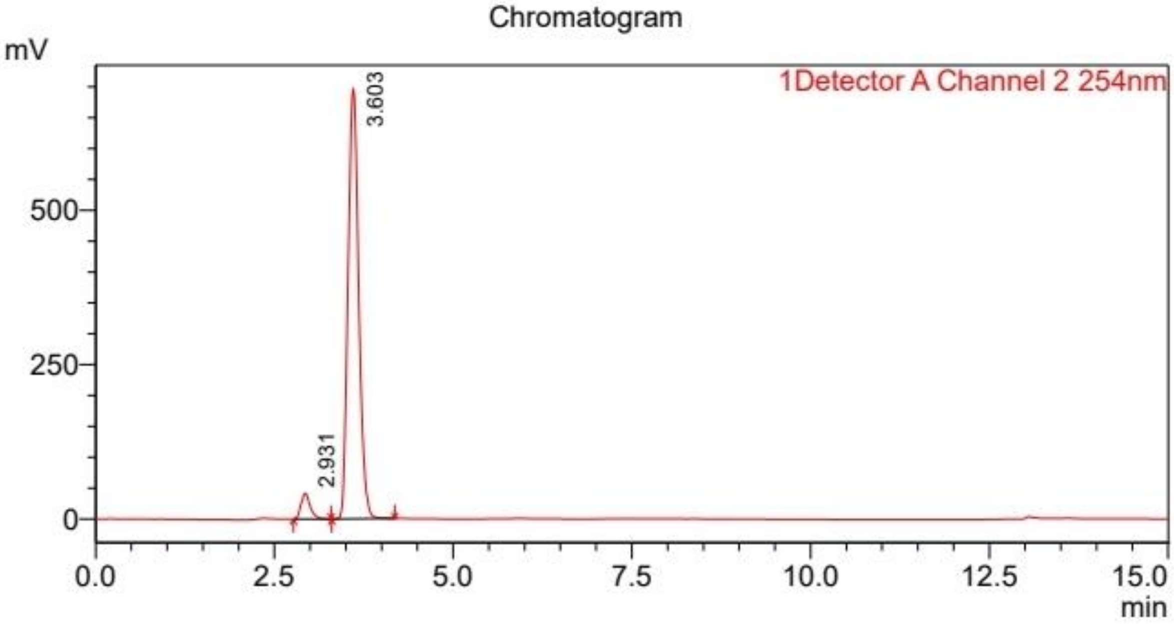

Detector A Channel 2 254nm

Peak#	Ret. Time (min)	Area%
1	2.931	4.855
2	3.603	95.145
Total		100.000

3-((4-chlorophenethyl)amino)pyridine 1-oxide (55)

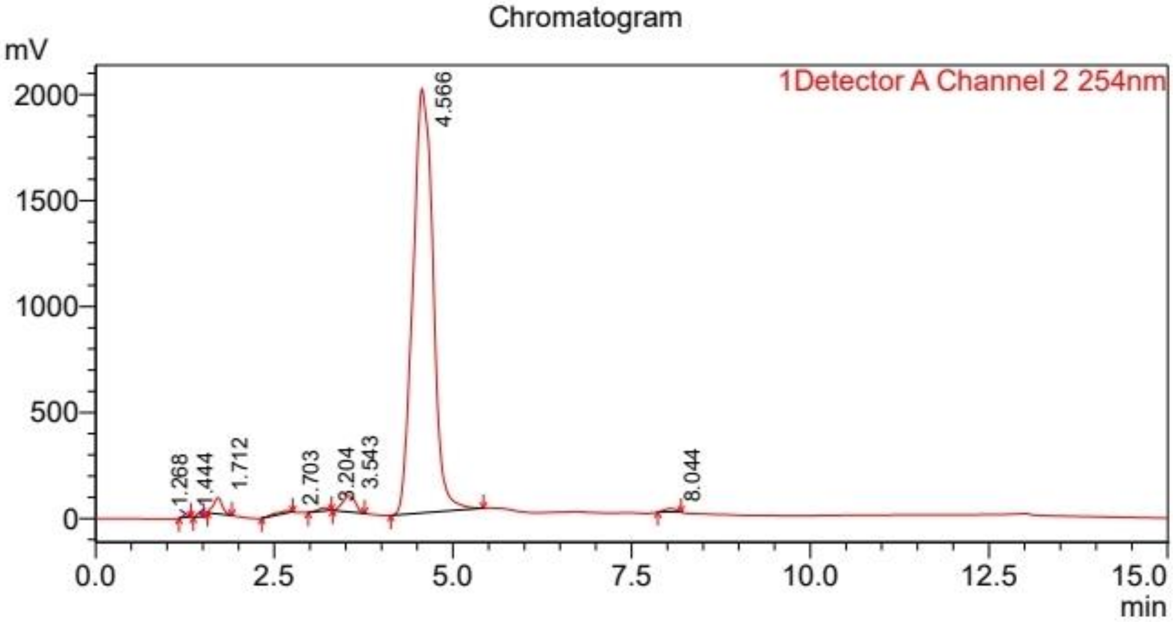

Detector A Channel 2 254nm

Peak#	Ret. Time (min)	Area%
1	1.268	0.184
2	1.444	0.170
3	1.712	1.597
4	2.703	0.459
5	3.204	0.326
6	3.543	2.268
7	4.566	94.552
8	8.044	0.445
Total		100.000

2-(2-((4-chloronaphthalen-1-yl)oxy)ethyl)-6-methoxypyridine (56)

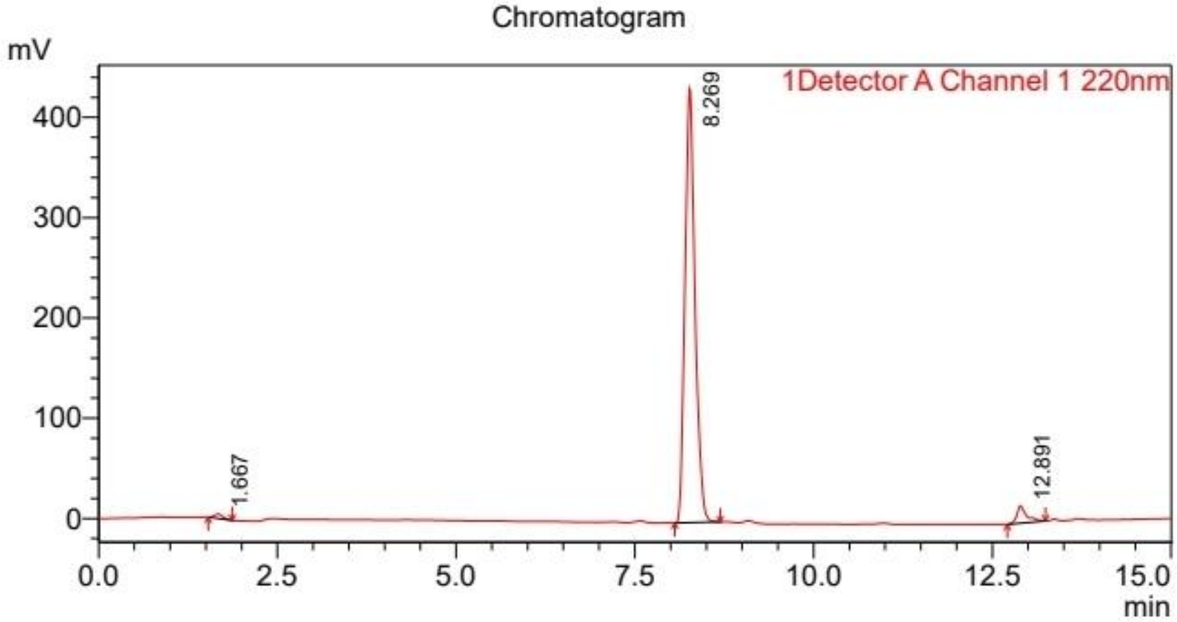

Detector A Channel 1 220nm

Peak#	Ret. Time (min)	Area%
1	1.667	0.841
2	8.269	95.481
3	12.891	3.678
Total		100.000

4-(2-((4-chloronaphthalen-1-yl)oxy)ethyl)-2-methoxypyridine (57)

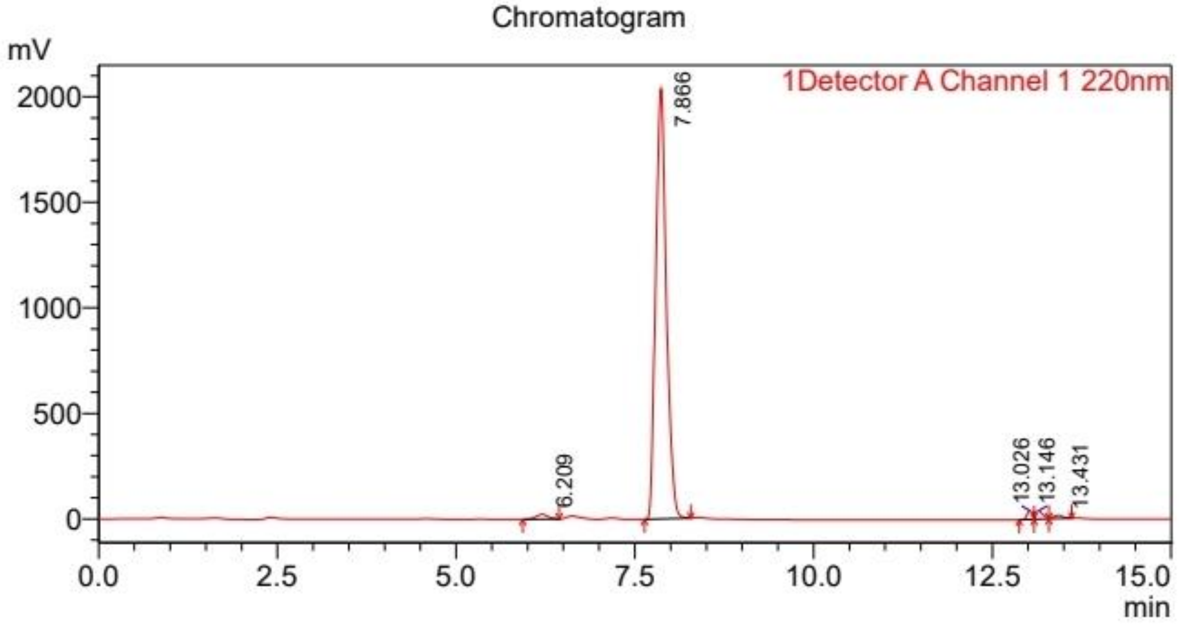

Detector A Channel 1 220nm

Peak#	Ret. Time (min)	Area%
1	6.209	1.135
2	7.866	95.677
3	13.026	1.097
4	13.146	1.384
5	13.431	0.708
Total		100.000

6-(2-((4-chloronaphthalen-1-yl)oxy)ethyl)pyridin-2-ol (58)

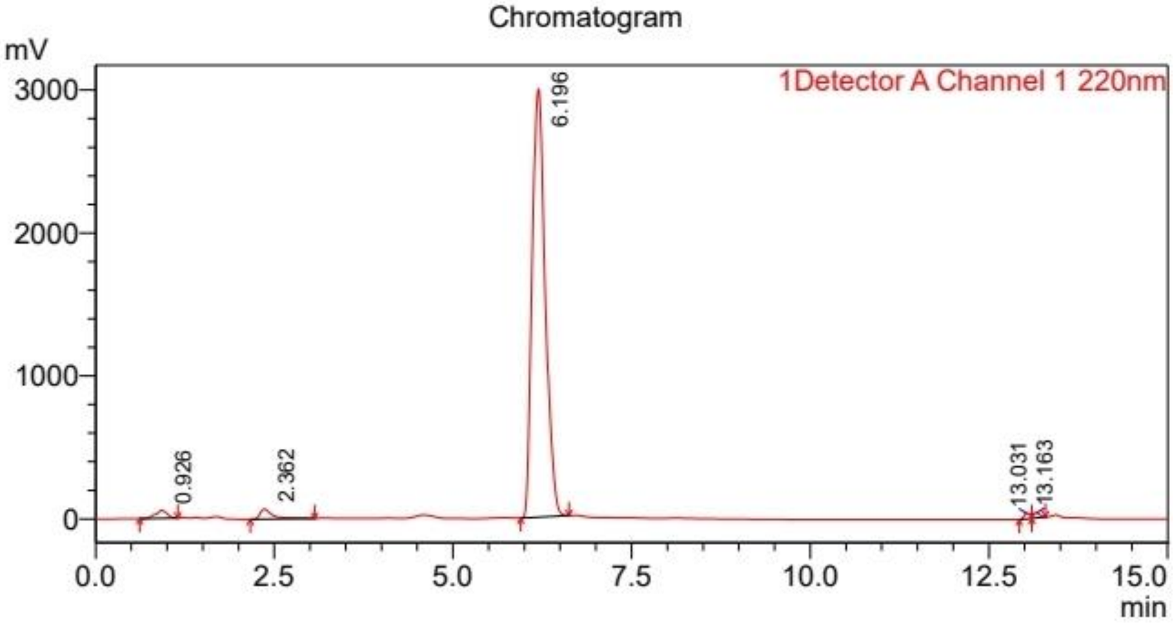

Detector A Channel 1 220nm

Peak#	Ret. Time (min)	Area%
1	0.926	1.586
2	2.362	2.033
3	6.196	95.203
4	13.031	0.600
5	13.163	0.578
Total		100.000

4-(2-((4-chloronaphthalen-1-yl)oxy)ethyl)pyridin-2-ol (59)

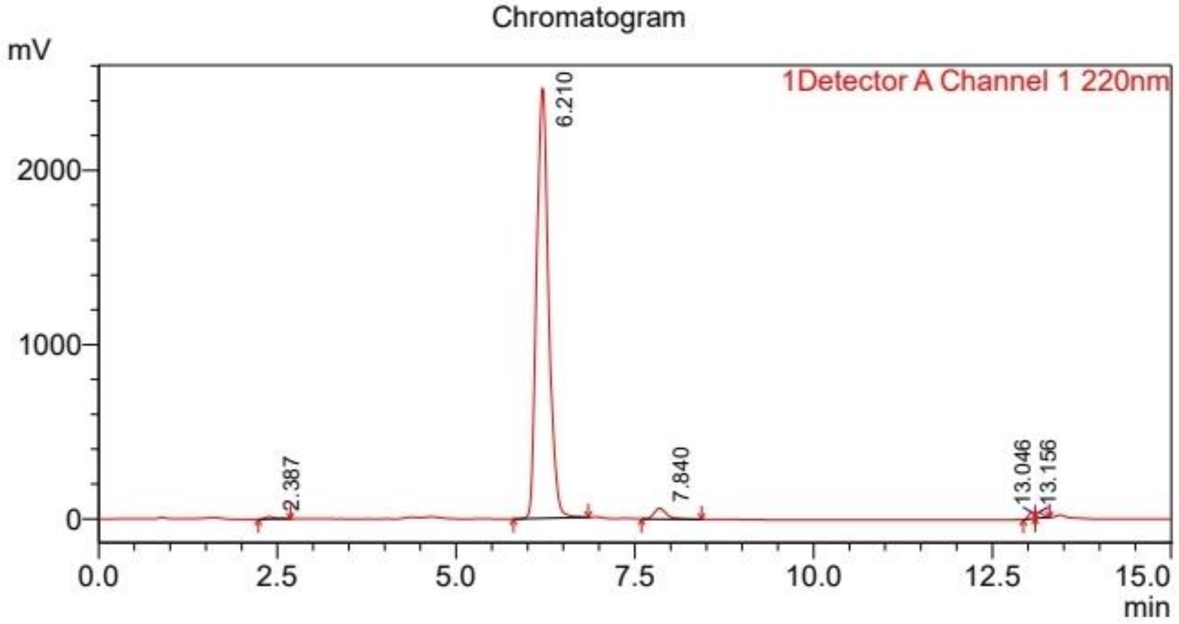

Detector A Channel 1 220nm

Peak#	Ret. Time (min)	Area%
1	2.387	0.440
2	6.210	95.663
3	7.840	2.394
4	13.046	0.744
5	13.156	0.760
Total		100.000

6-(2-(4-chlorophenoxy)ethyl)pyridin-2-ol (60)

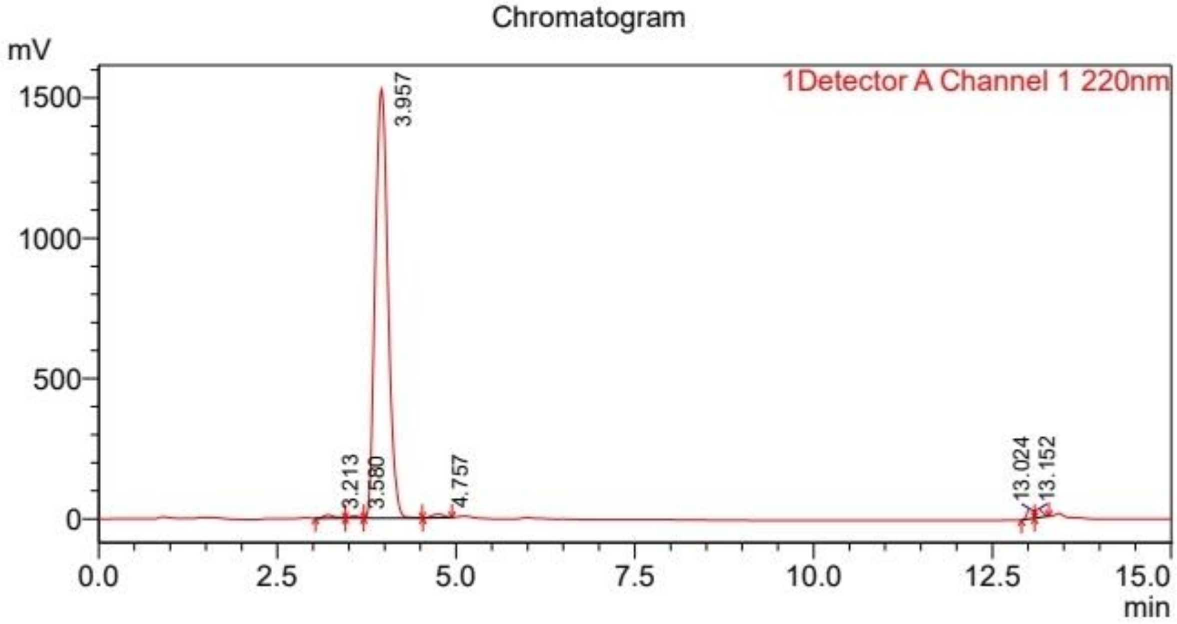

Detector A Channel 1 220nm

Peak#	Ret. Time (min)	Area%
1	3.213	0.690
2	3.580	0.380
3	3.957	96.014
4	4.757	0.760
5	13.024	1.132
6	13.152	1.022
Total		100.000

5-(2-((4-chloronaphthalen-1-yl)oxy)ethyl)-2-methylpyridine 1-oxide (61)

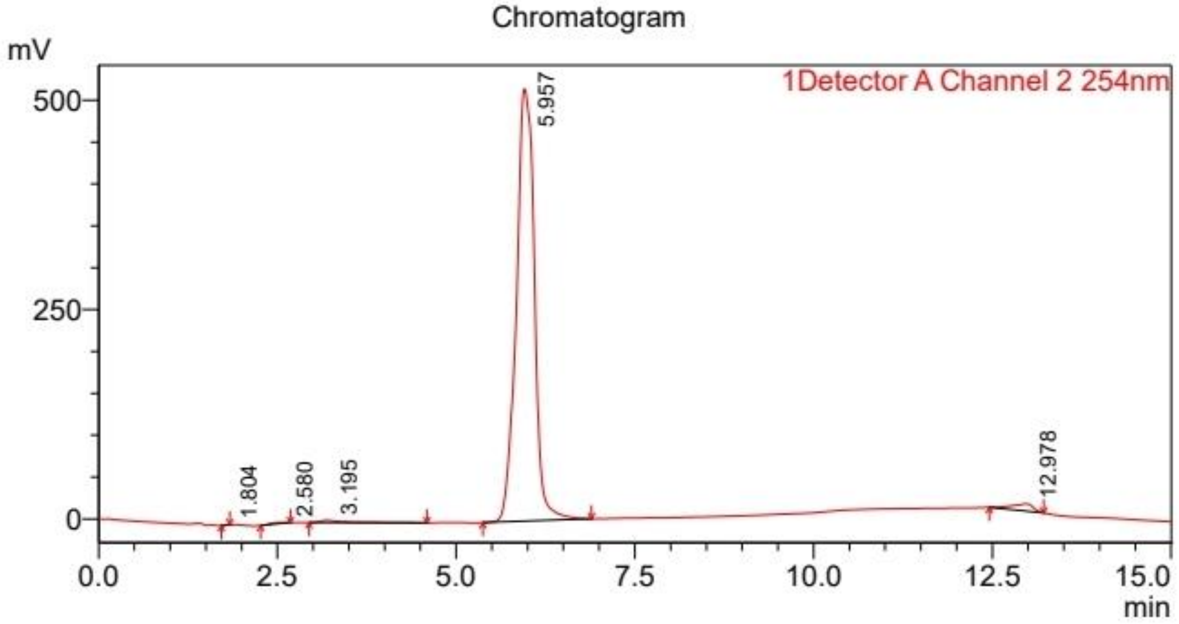

Detector A Channel 2 254nm

Peak#	Ret. Time (min)	Area%
1	1.804	0.017
2	2.580	0.234
3	3.195	0.663
4	5.957	97.182
5	12.978	1.904
Total		100.000

5-(2-((4-chloronaphthalen-1-yl)oxy)ethyl)-2-ethylpyridine 1-oxide (62)

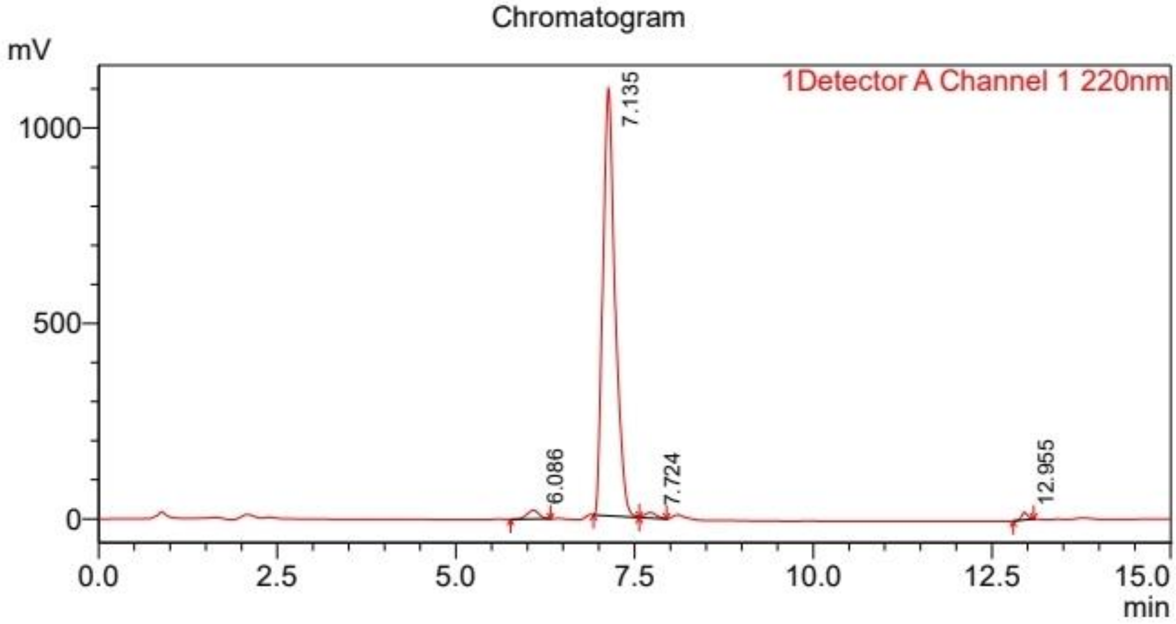

Detector A Channel 1 220nm

Peak#	Ret. Time (min)	Area%
1	6.086	1.944
2	7.135	96.051
3	7.724	1.148
4	12.955	0.857
Total		100.000

3-(2-((4-chloronaphthalen-1-yl)oxy)ethyl)-2,6-dimethylpyridine 1-oxide (63)

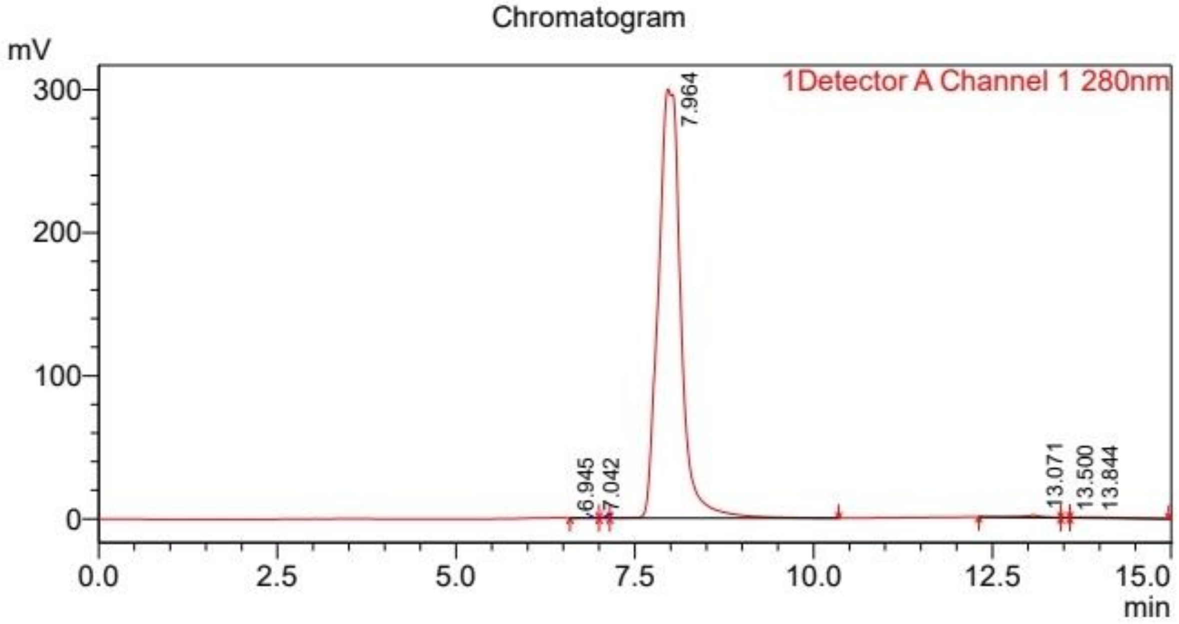

Detector A Channel 1 280nm

Peak#	Ret. Time (min)	Area%
1	6.945	0.045
2	7.042	0.026
3	7.964	98.960
4	13.071	0.535
5	13.500	0.038
6	13.844	0.397
Total		100.000

3-(3-(4-chloronaphthalen-1-yl)propyl)-5-methoxypyridine 1-oxide (64)

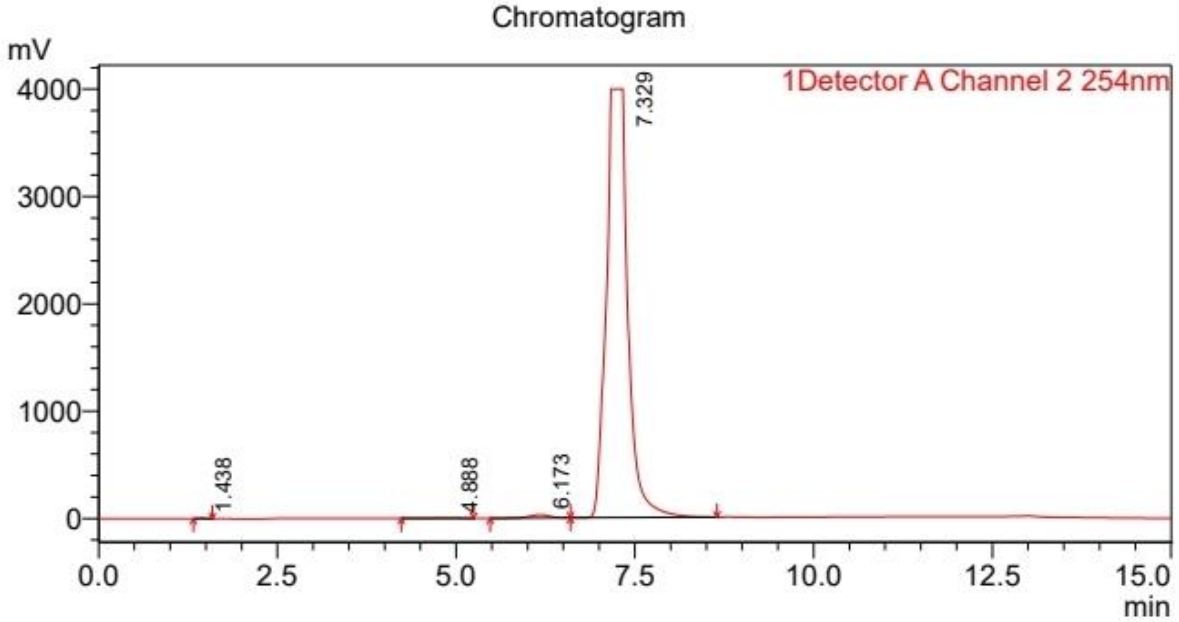

Detector A Channel 2 254nm

Peak#	Ret. Time (min)	Area%
1	1.438	0.034
2	4.888	0.116
3	6.173	0.745
4	7.329	99.105
Total		100.000

3-(3-(4-chloronaphthalen-1-yl)propyl)-5-methoxypyridine 1-oxide (65)

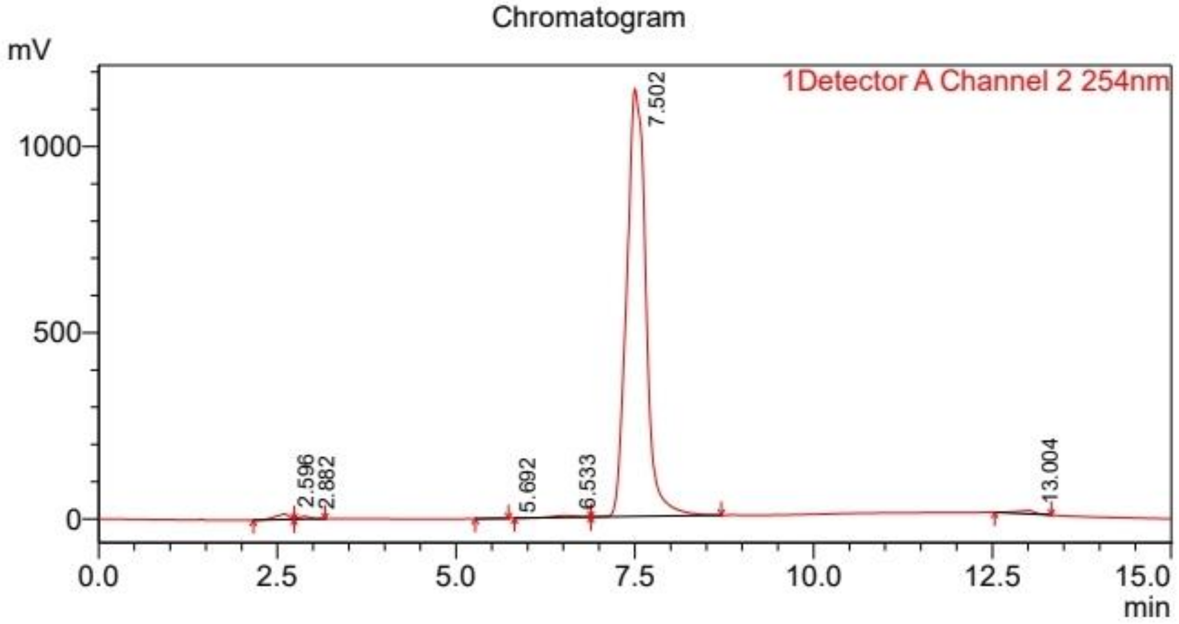

Detector A Channel 2 254nm

Peak#	Ret. Time (min)	Area%
1	2.596	0.941
2	2.882	0.393
3	5.692	0.016
4	6.533	0.547
5	7.502	97.339
6	13.004	0.764
Total		100.000

3-(3-(4-chloronaphthalen-1-yl)propyl)-5-hydroxypyridine 1-oxide (66)

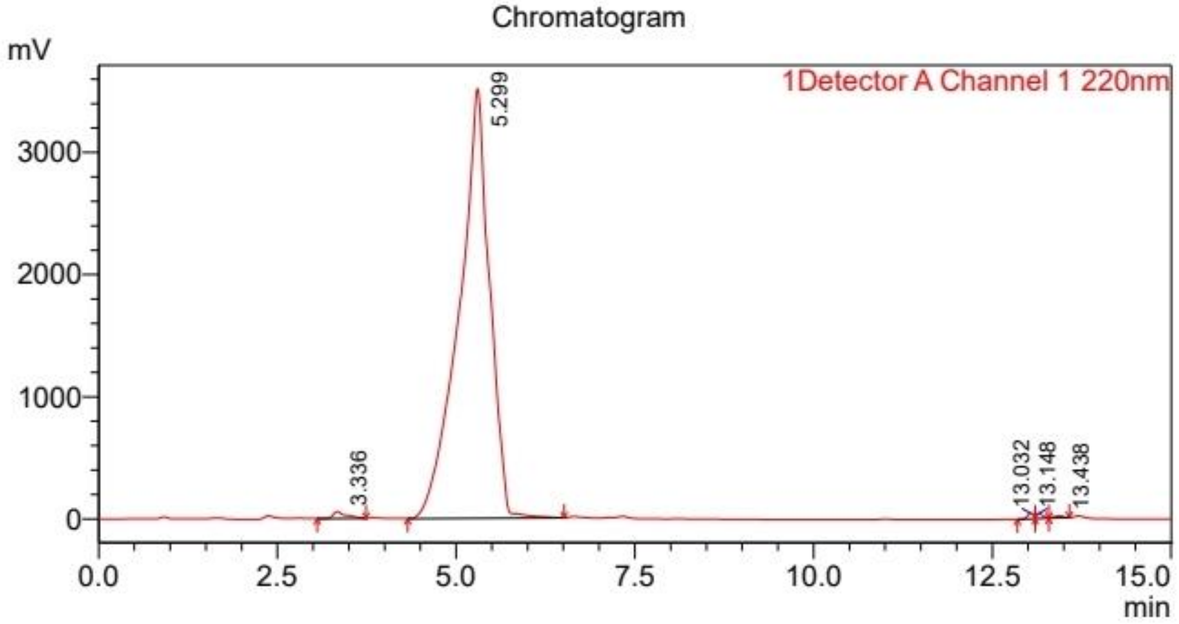

Detector A Channel 1 220nm

Peak#	Ret. Time (min)	Area%
1	3.336	0.676
2	5.299	98.626
3	13.032	0.278
4	13.148	0.267
5	13.438	0.153
Total		100.000

3-(2-((4-chloronaphthalen-1-yl)oxy)ethyl)-5-fluoropyridine 1-oxide (67)

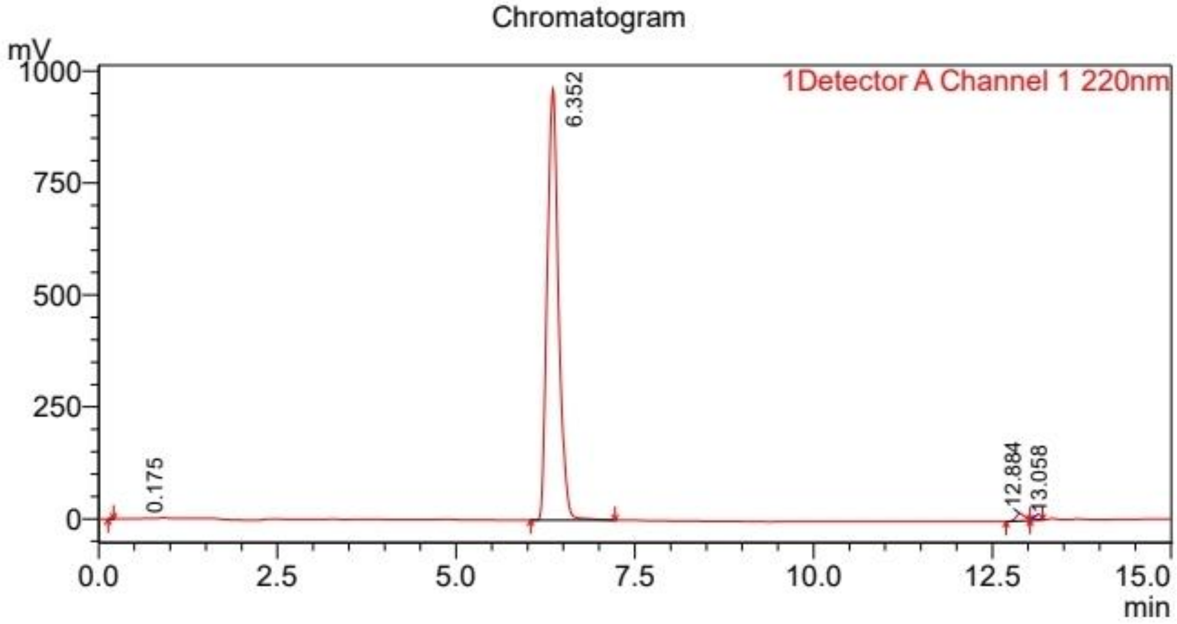

Detector A Channel 1 220nm

Peak#	Ret. Time (min)	Area%
1	0.175	0.002
2	6.352	98.484
3	12.884	1.314
4	13.058	0.200
Total		100.000

3-(3-(3,5-bis(trifluoromethyl)phenyl)propyl)-5-hydroxypyridine 1-oxide (68)

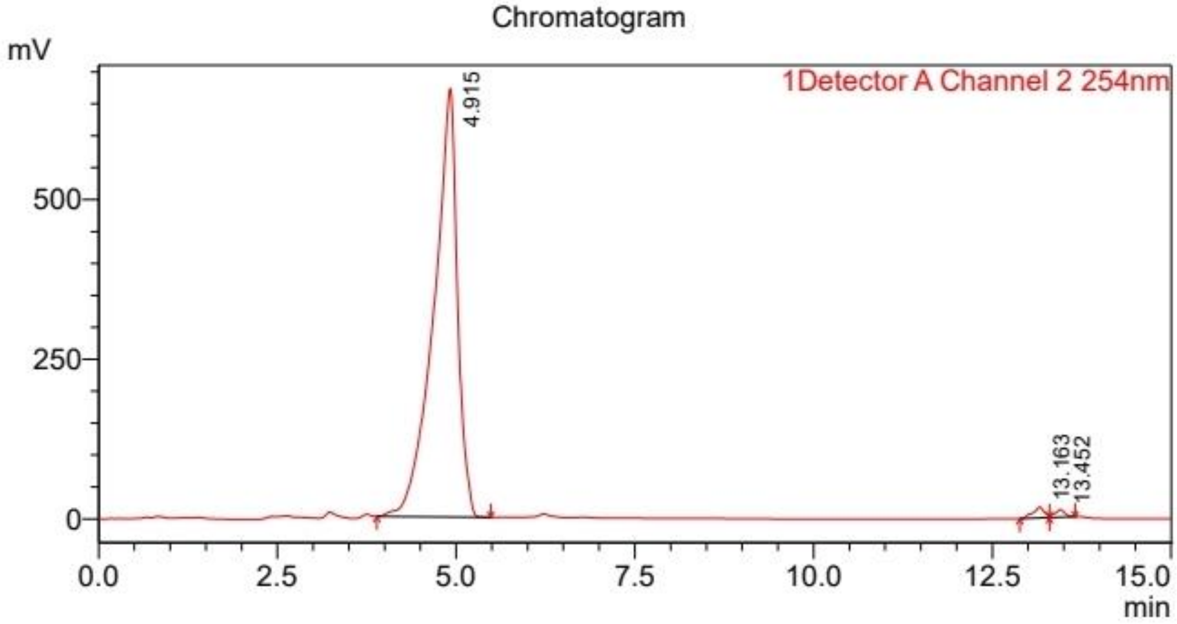

Detector A Channel 2 254nm

Peak#	Ret. Time (min)	Area%
1	4.915	98.098
2	13.163	1.185
3	13.452	0.717
Total		100.000

3-(3-(4-chloro-3-(trifluoromethyl)phenyl)propyl)-5-hydroxypyridine 1-oxide (69)

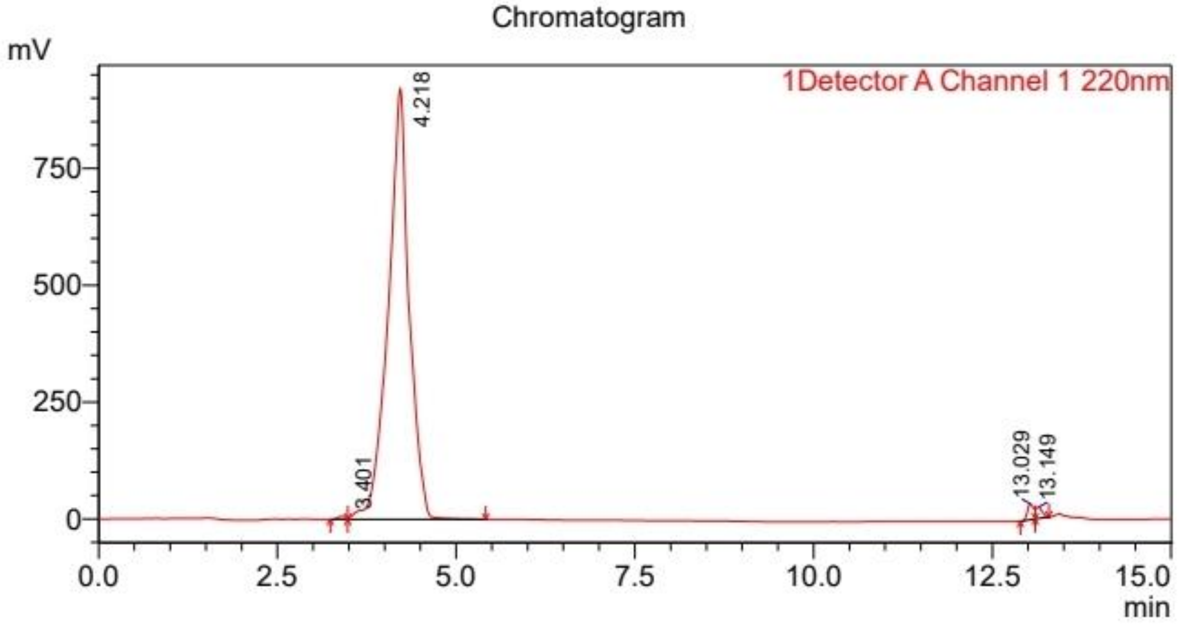

Detector A Channel 1 220nm

Peak#	Ret. Time (min)	Area%
1	3.401	0.264
2	4.218	97.869
3	13.029	1.096
4	13.149	0.770
Total		100.000

HRMS spectra for final products

3-(2-((4-bromonaphthalen-1-yl)oxy)ethyl)pyridine 1-oxide (12)

Expanded Spectrum RT 0.11, NL 6638895, Peak [1], Target Mass 344.0281

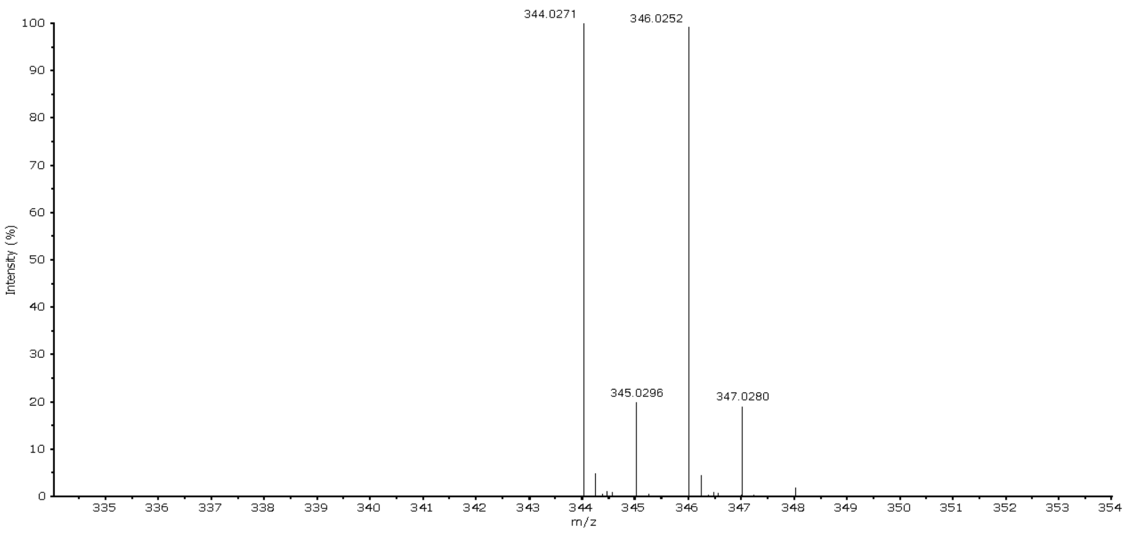

Theoretical Spectrum for C17H15NO2Br, Minimum Abundance 0.01%

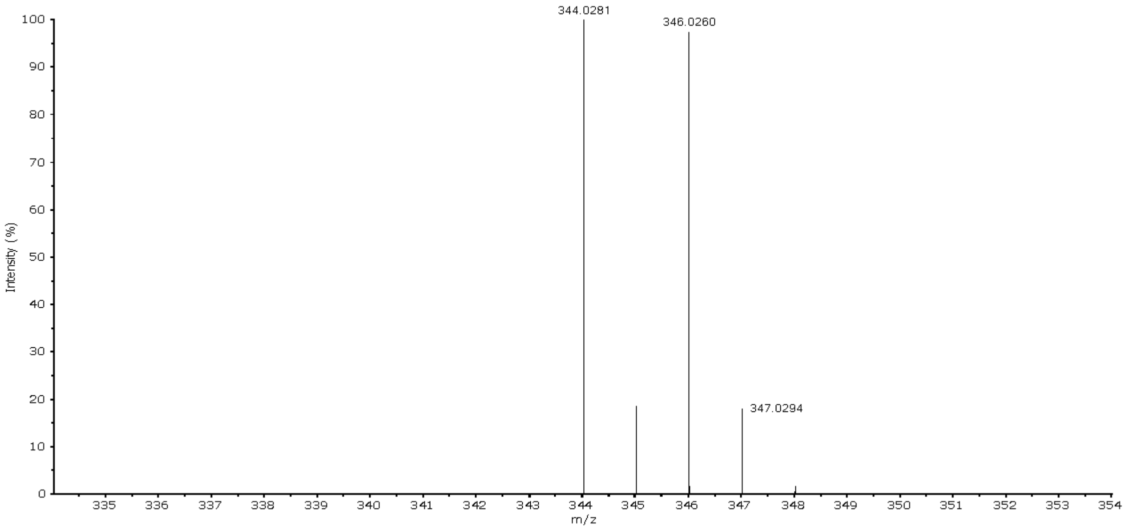

3-(2-((4-fluoronaphthalen-1-yl)oxy)ethyl)pyridine 1-oxide (13)

Expanded Spectrum RT 0.12, NL 2658101, Peak [1], Target Mass 284.1081

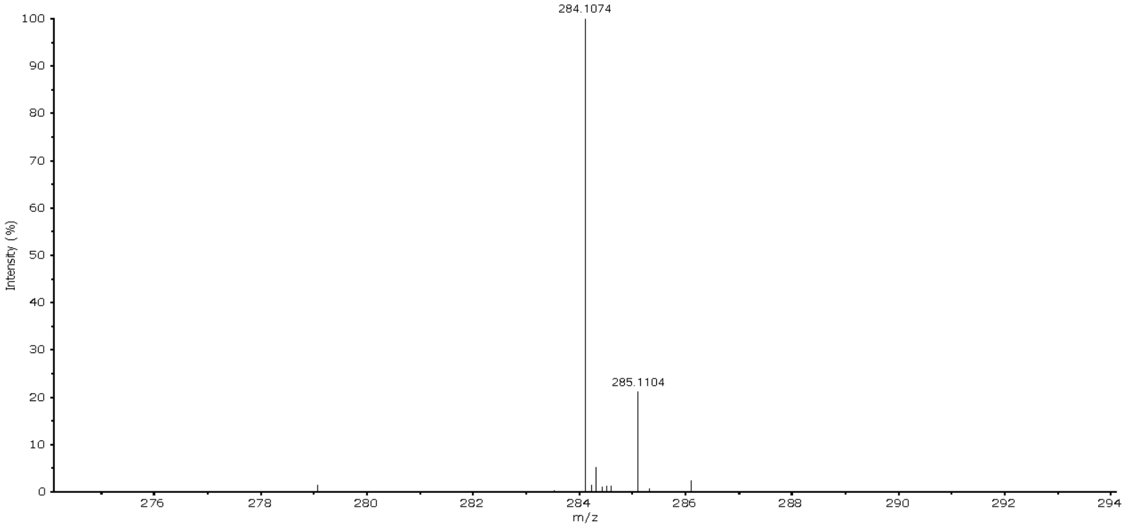

Theoretical Spectrum for C17H15NO2F, Minimum Abundance 0.01%

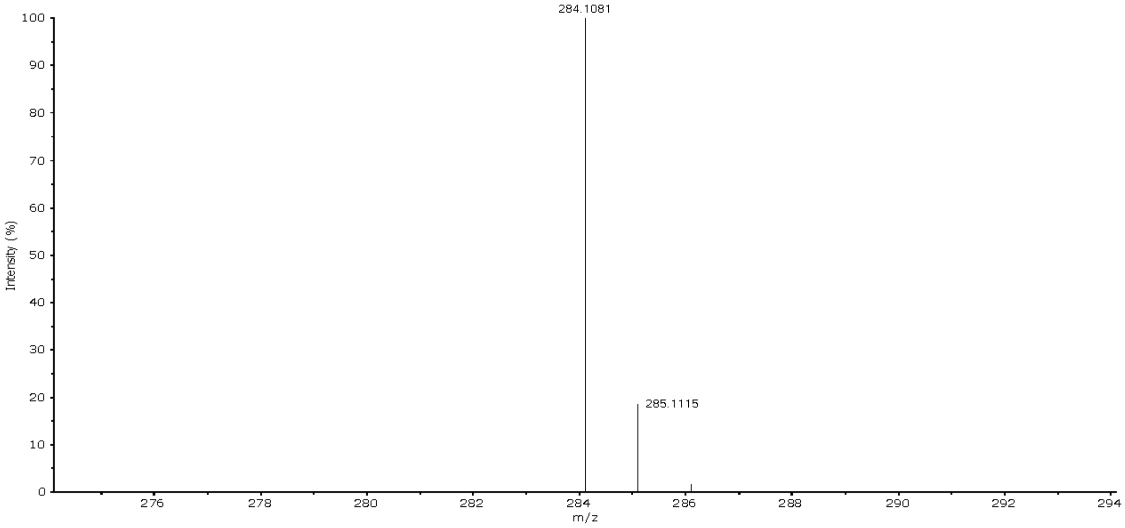

3-(2-((4-methoxynaphthalen-1-yl)oxy)ethyl)pyridine 1-oxide (14)

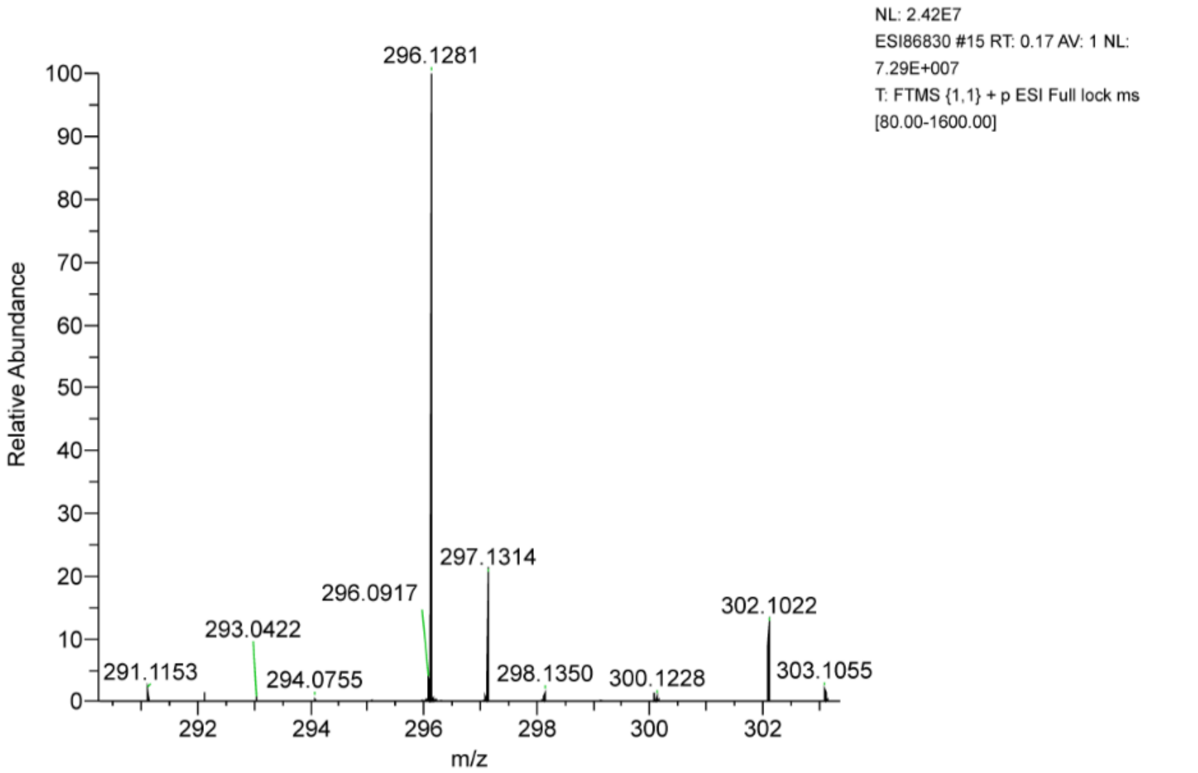

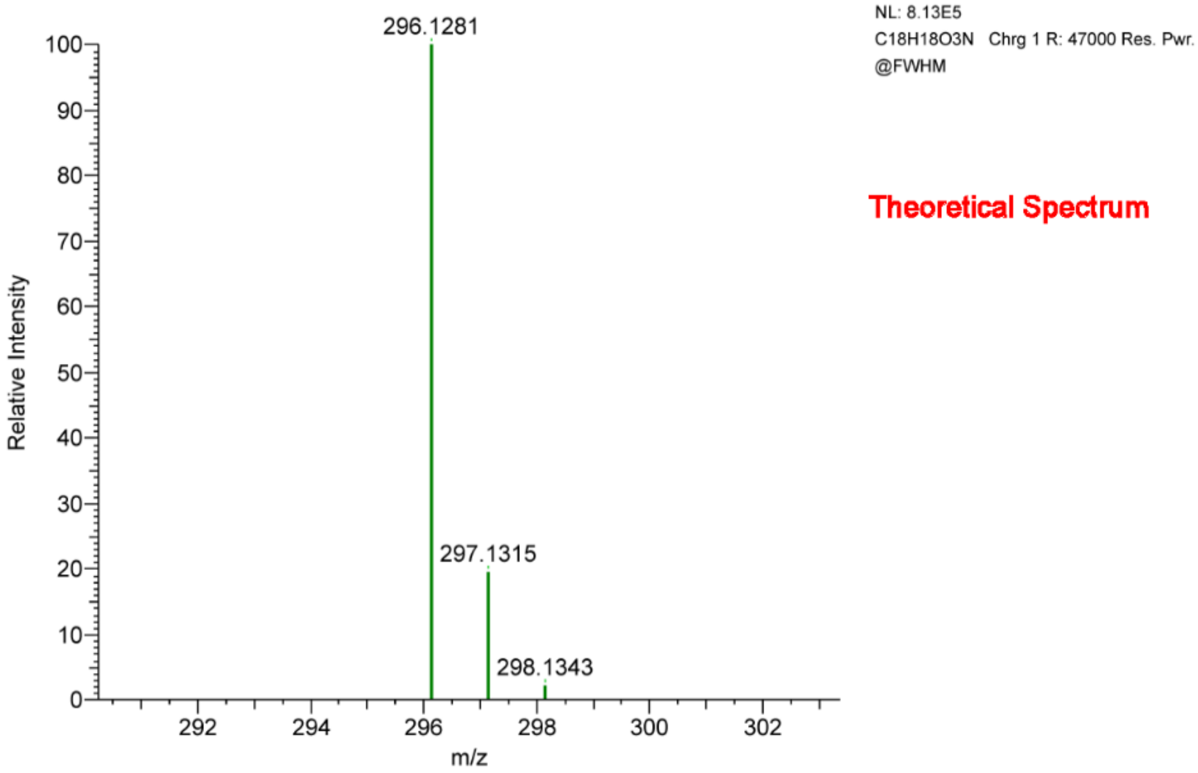

3-(2-(naphthalen-1-yloxy)ethyl)pyridine 1-oxide (15)

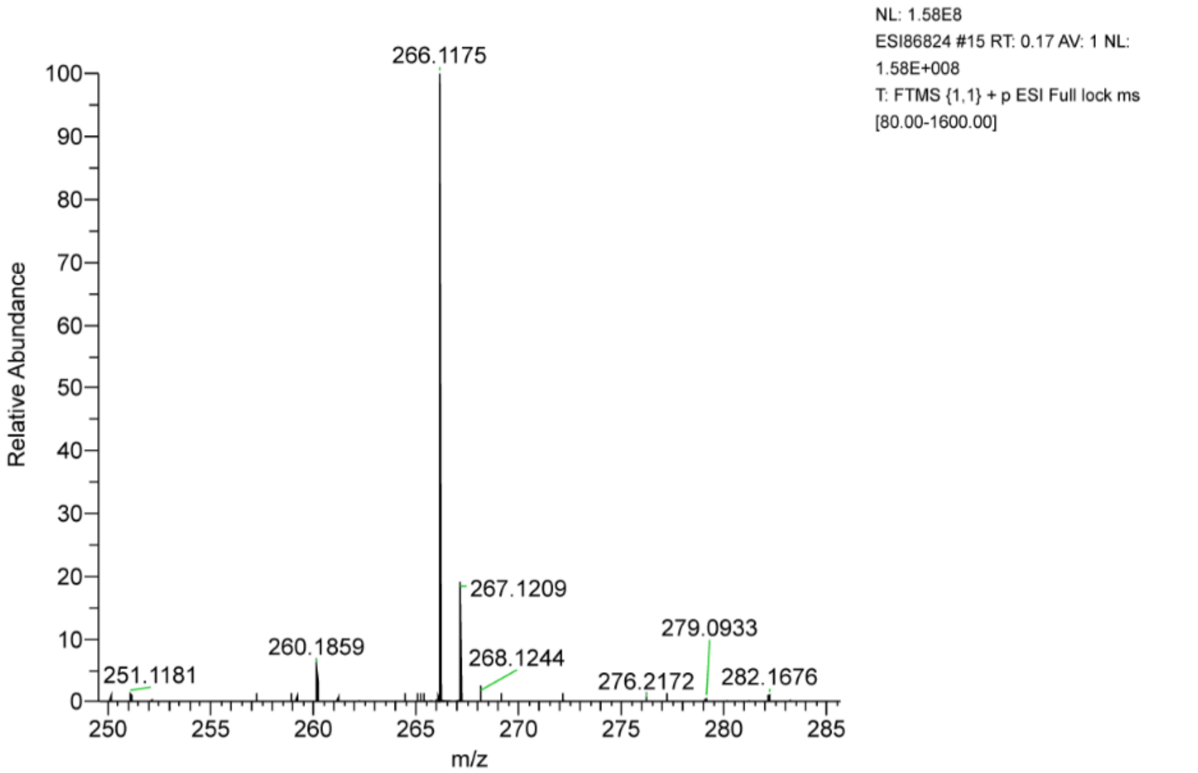

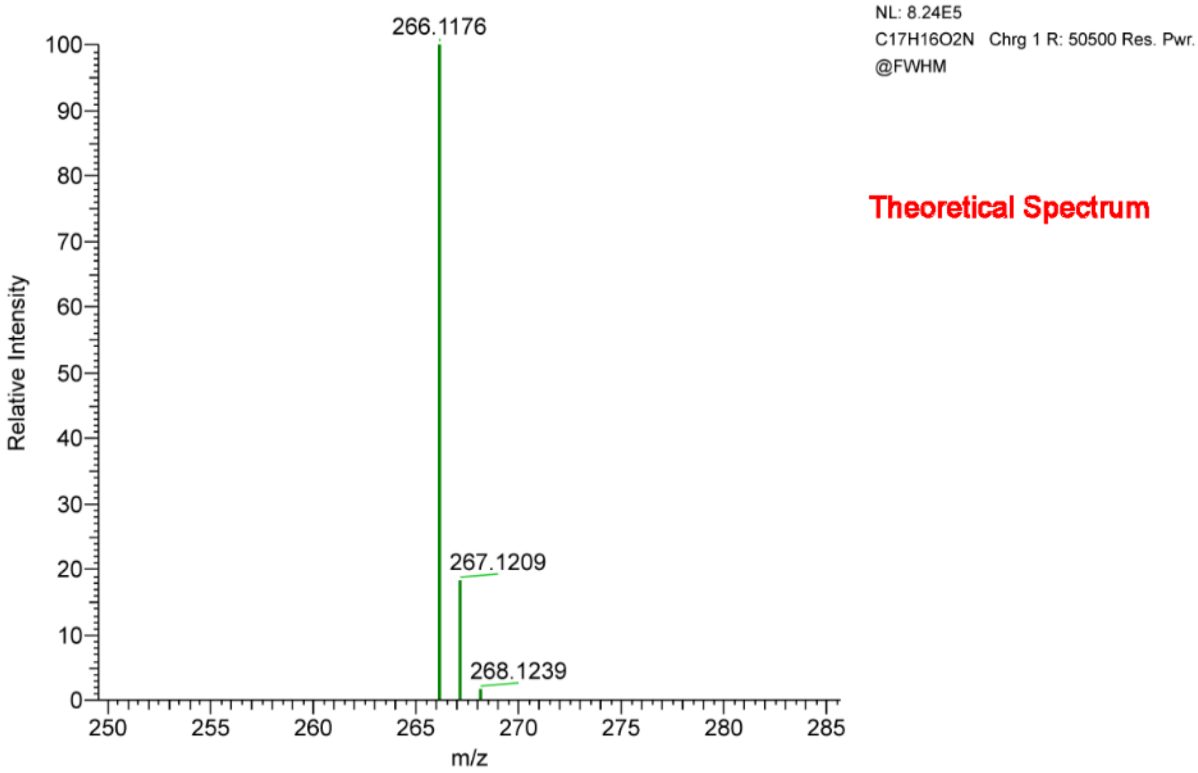

3-(2-((4-chloro-5,6,7,8-tetrahydronaphthalen-1-yl)oxy)ethyl)pyridine 1-oxide (16)

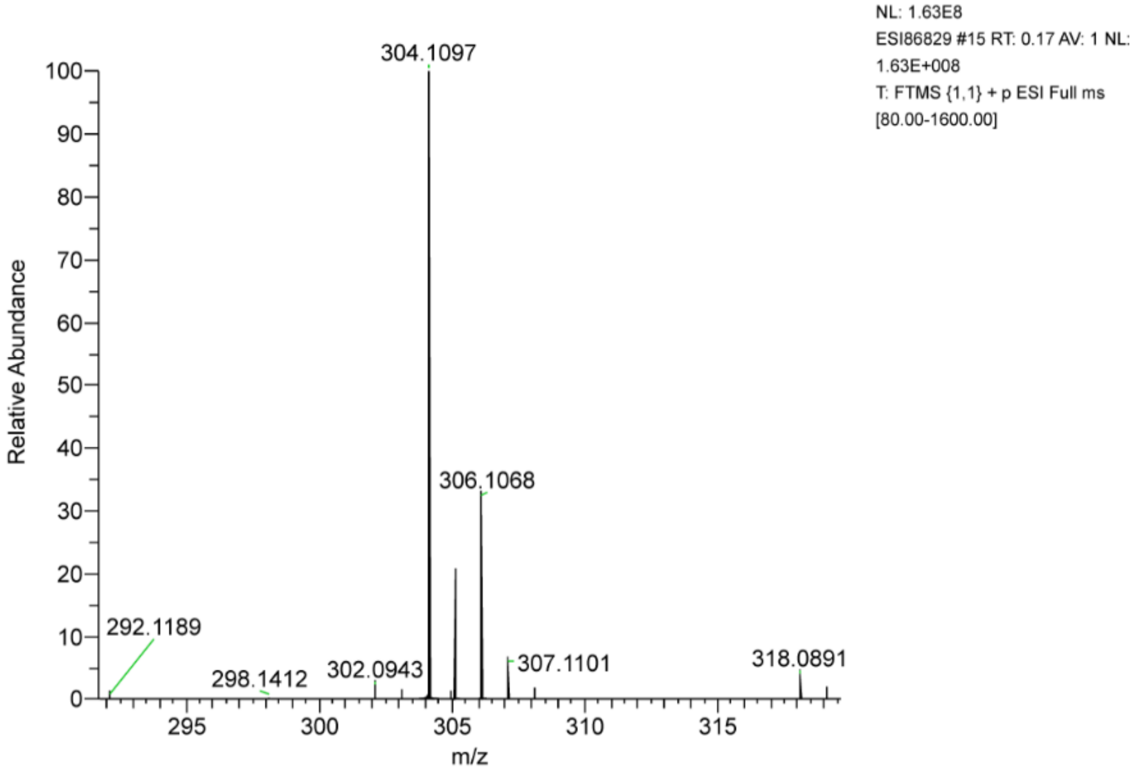

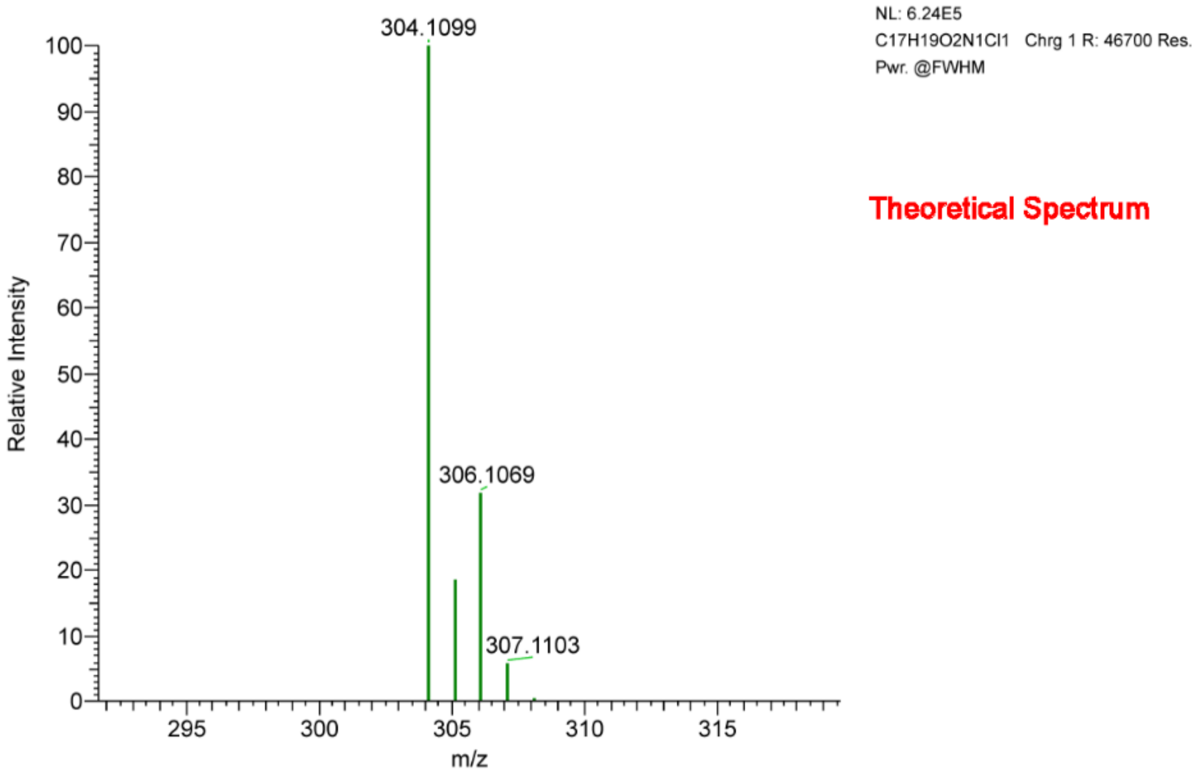

3-(2-((1,2,3,4-tetrahydronaphthalen-1-yl)oxy)ethyl)pyridine 1-oxide (17)

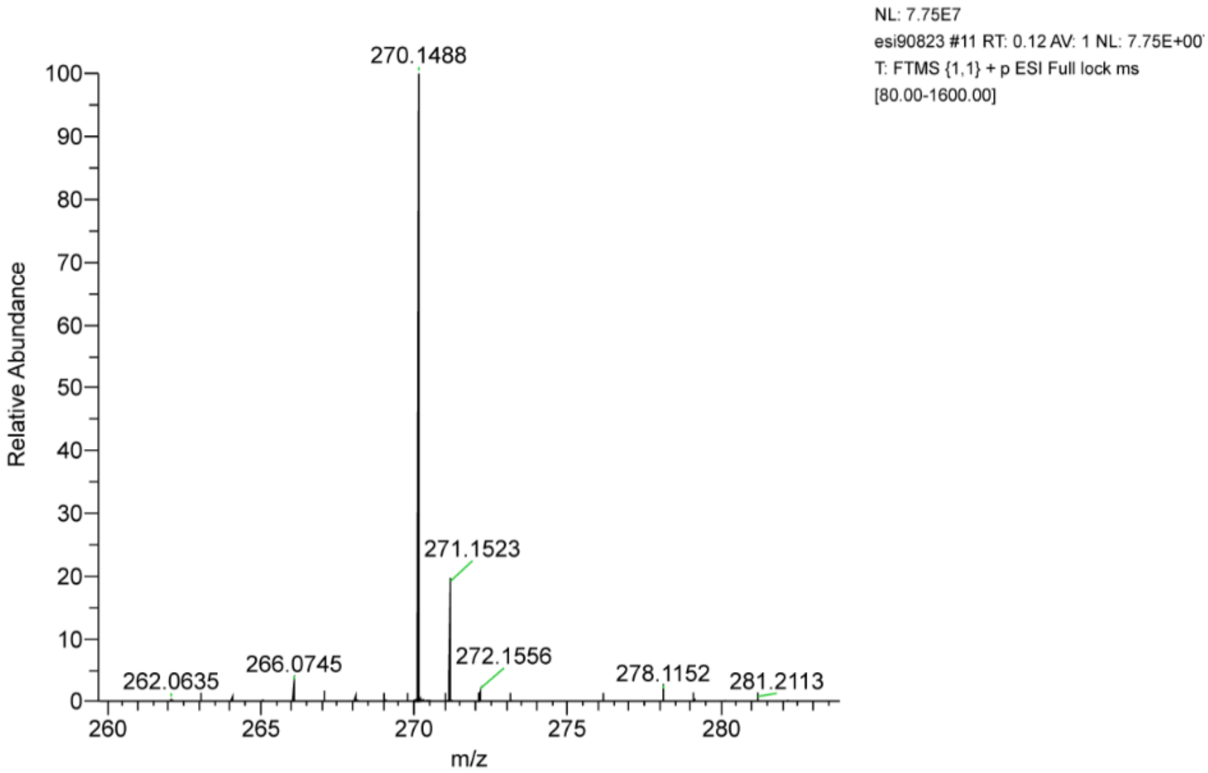

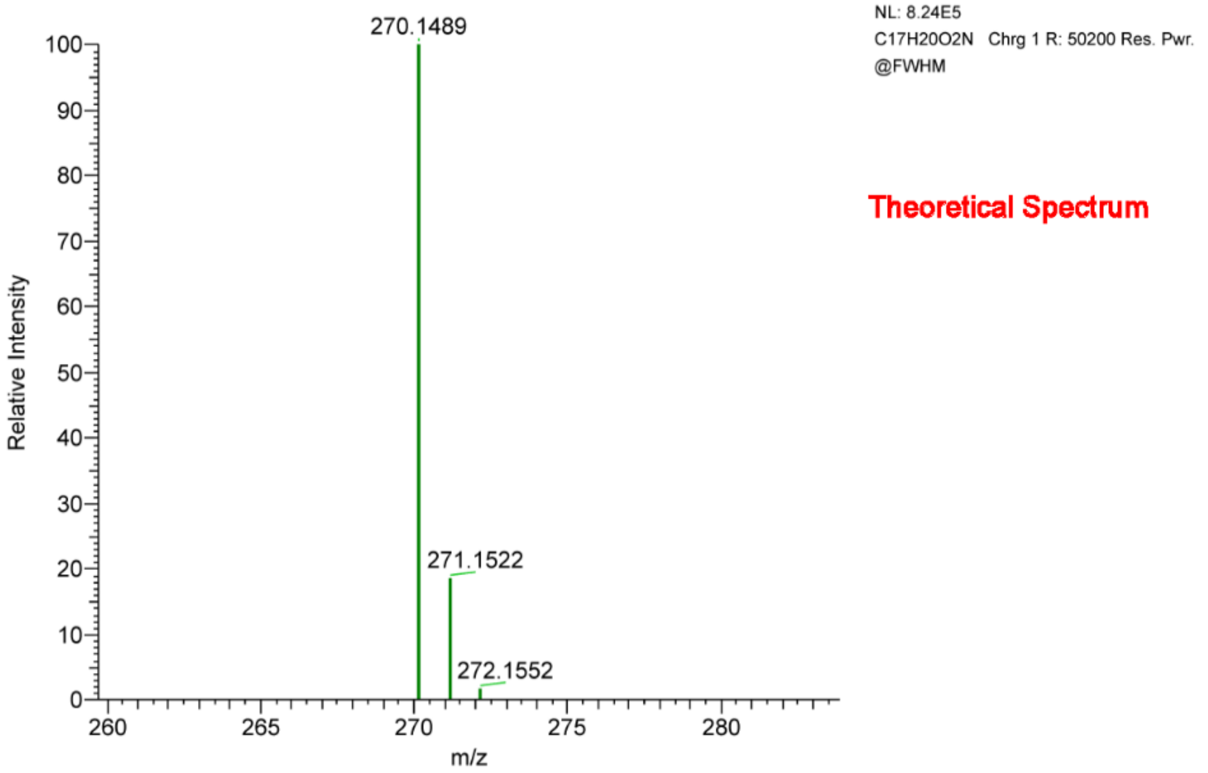

3-(2-((2,3-dihydro-1H-inden-1-yl)oxy)ethyl)pyridine 1-oxide (18)

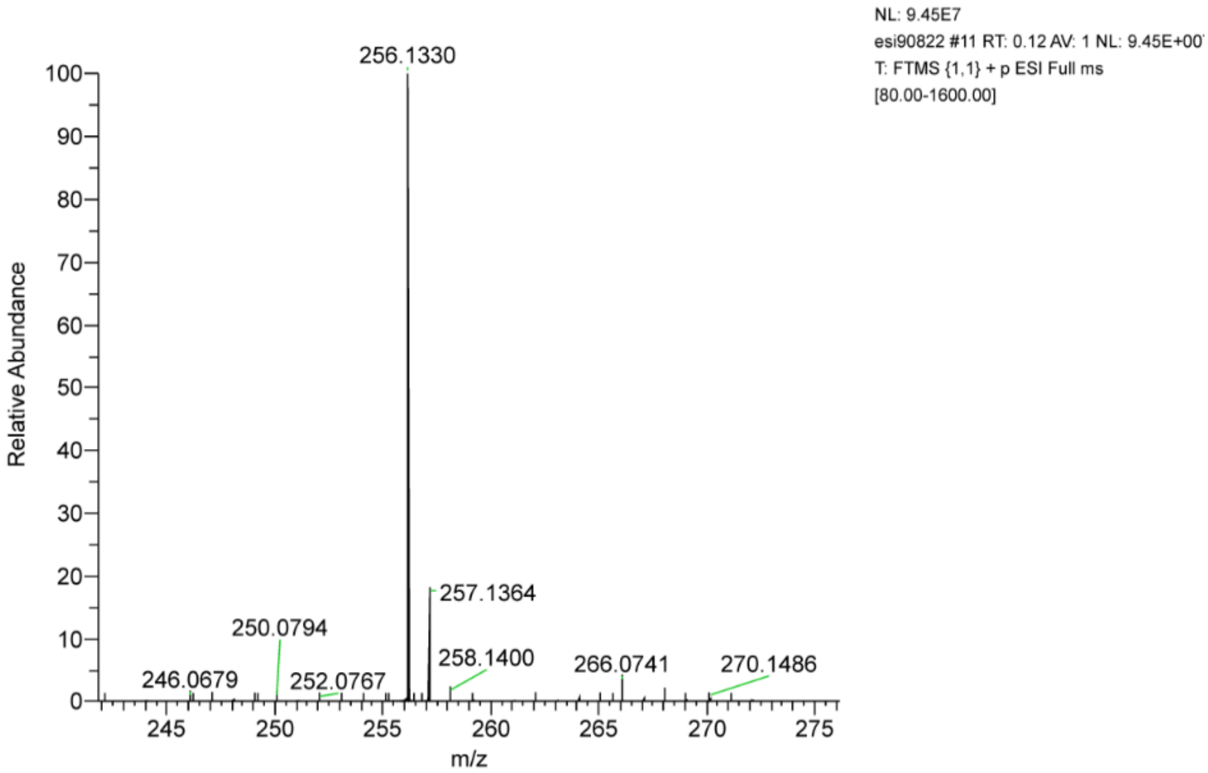

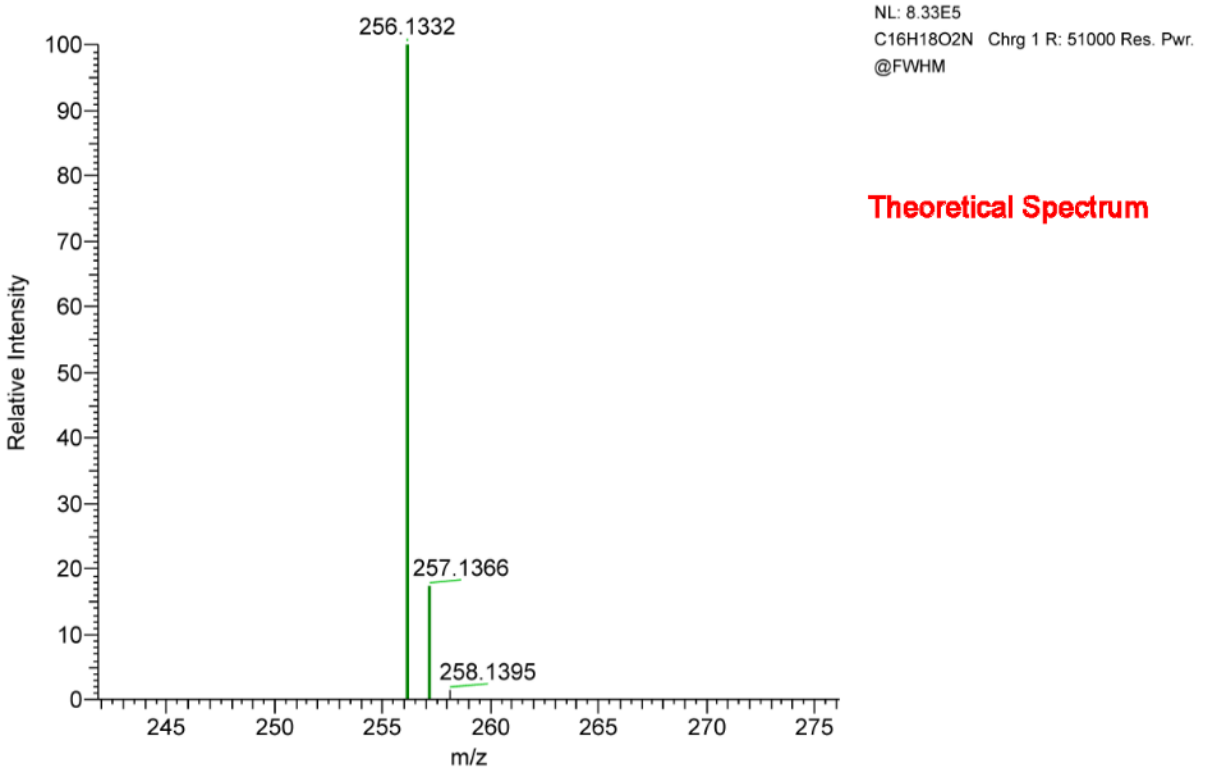

3-(2-(4-chlorophenoxy)ethyl)pyridine 1-oxide (19)

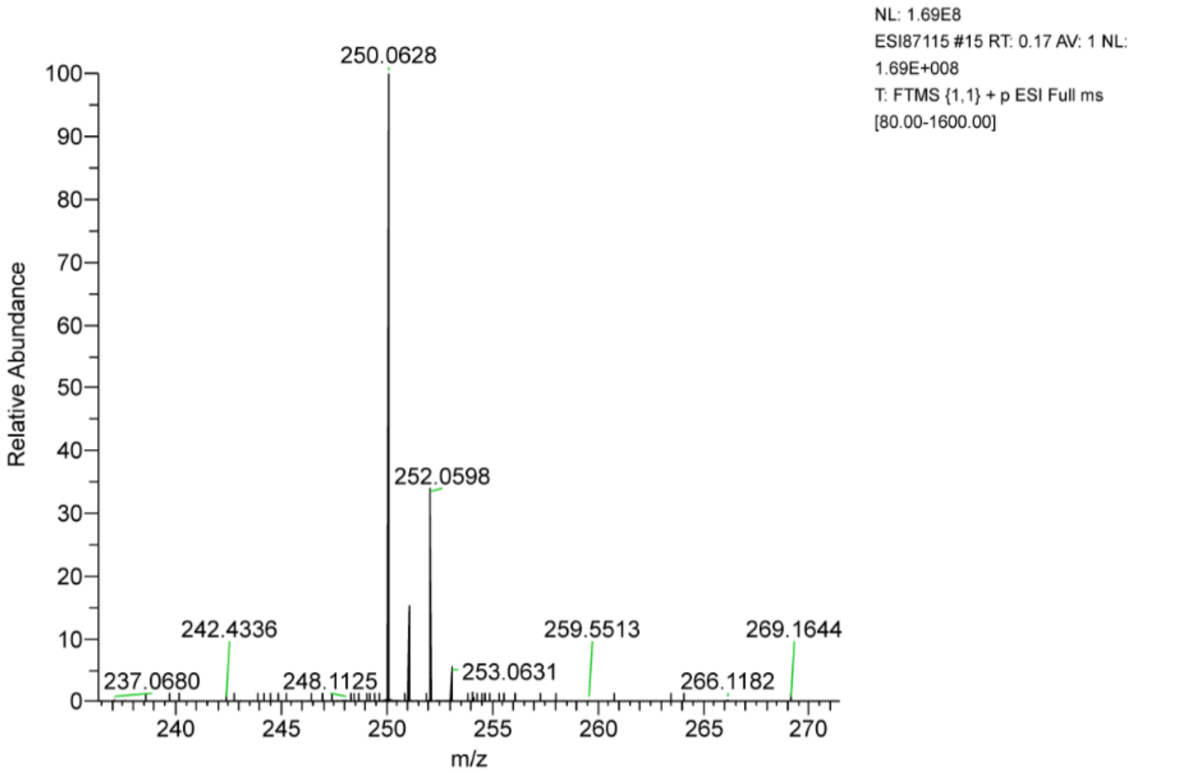

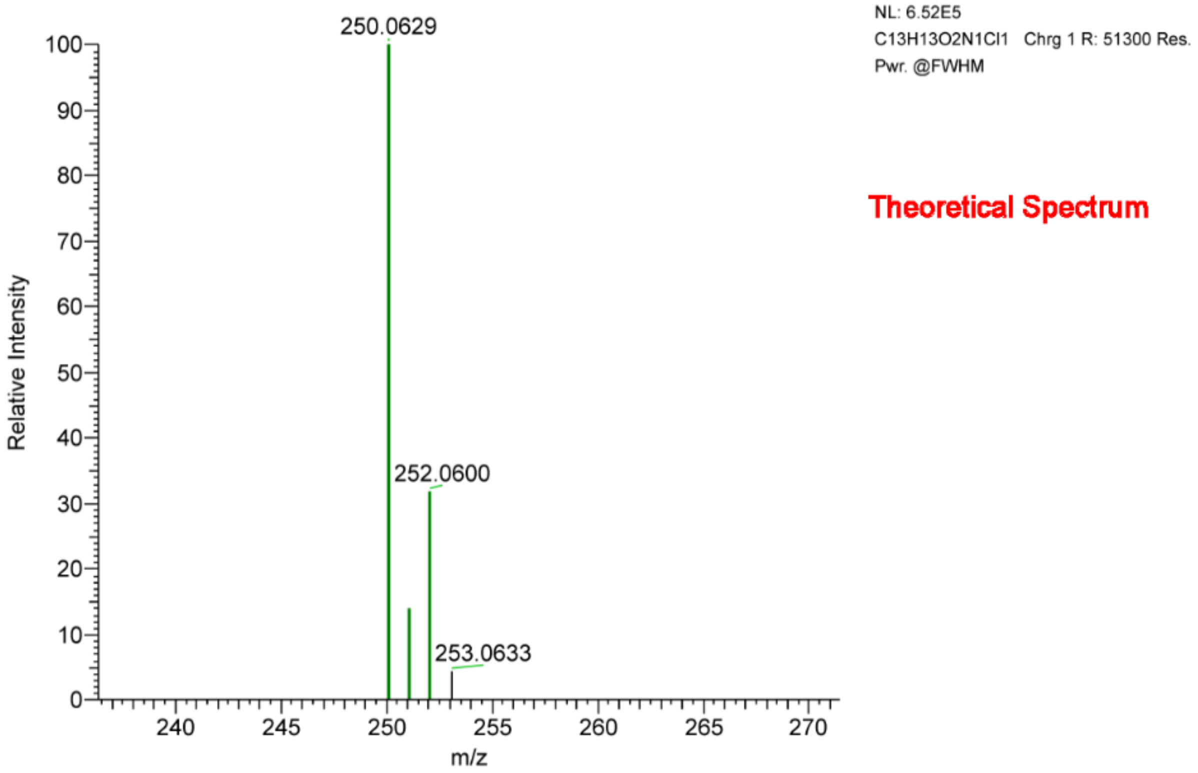

3-(2-(4-bromophenoxy)ethyl)pyridine 1-oxide (20)

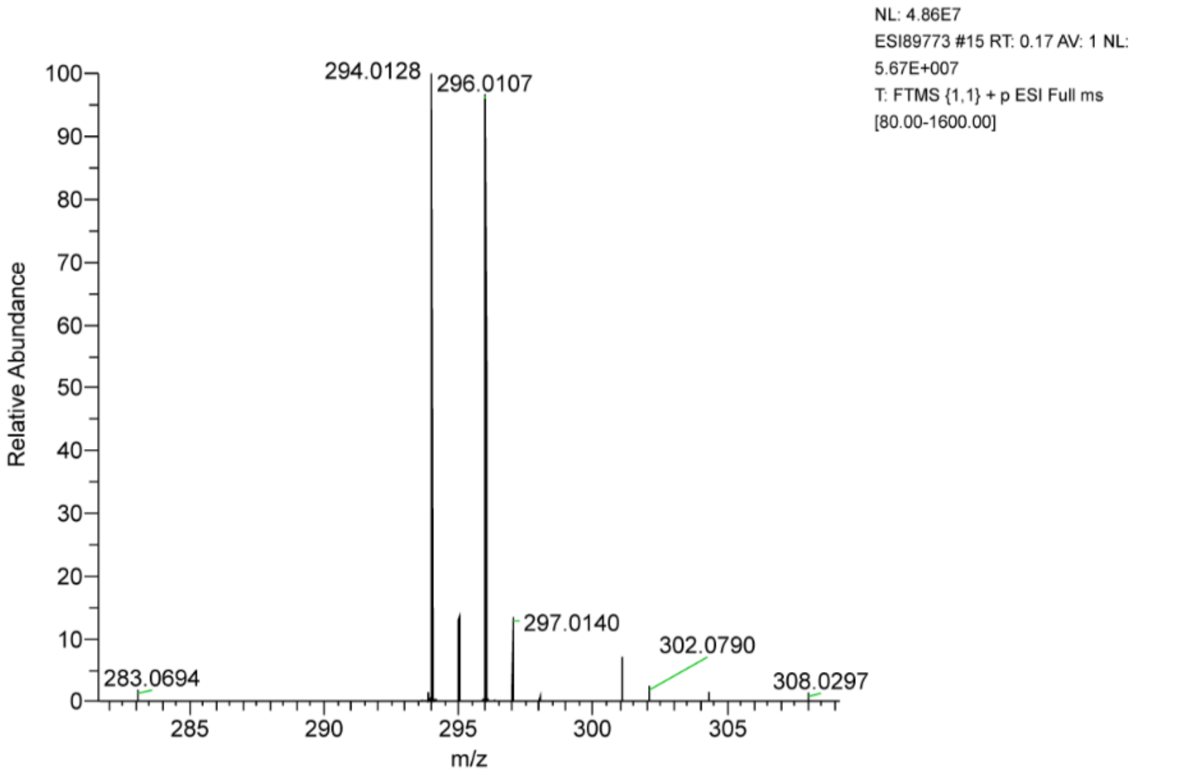

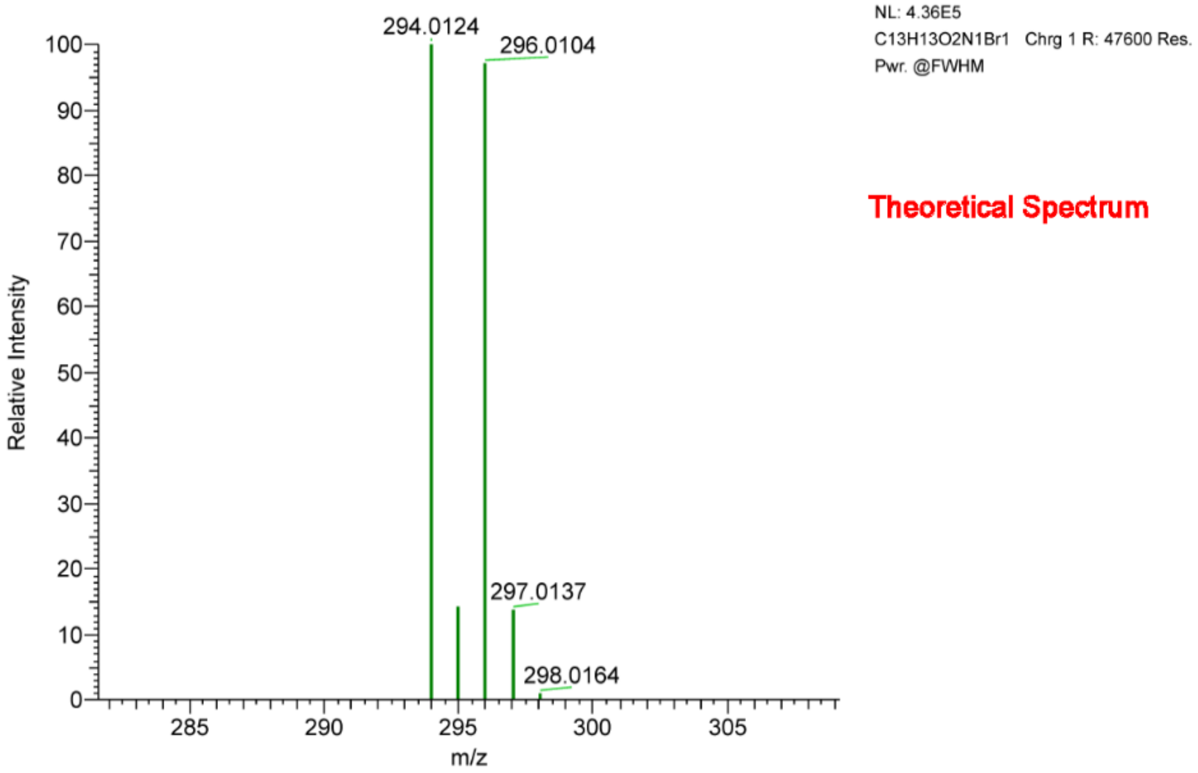

3-(2-(4-cyanophenoxy)ethyl)pyridine 1-oxide (21)

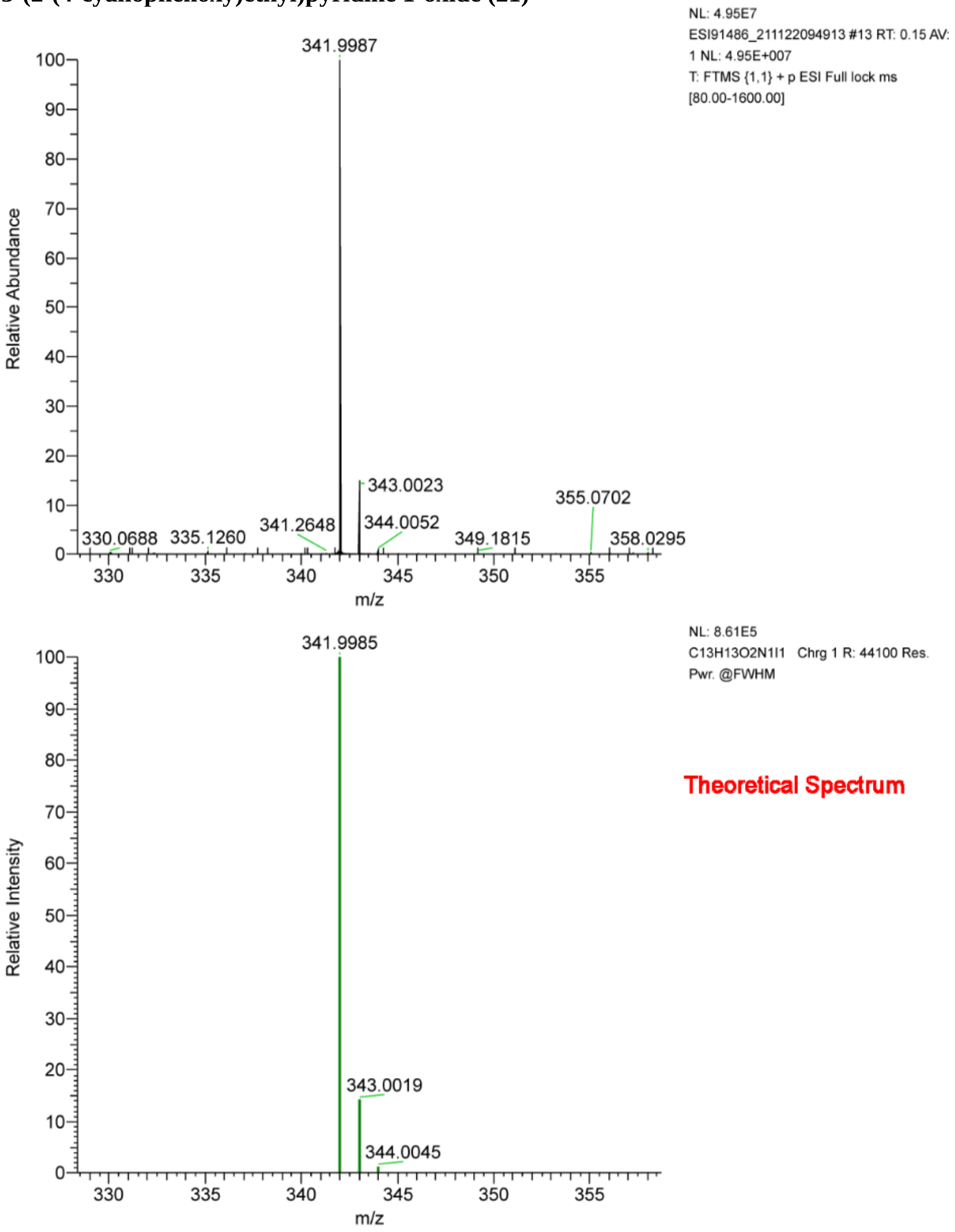

3-(2-(4-cyanophenoxy)ethyl)pyridine 1-oxide (22)

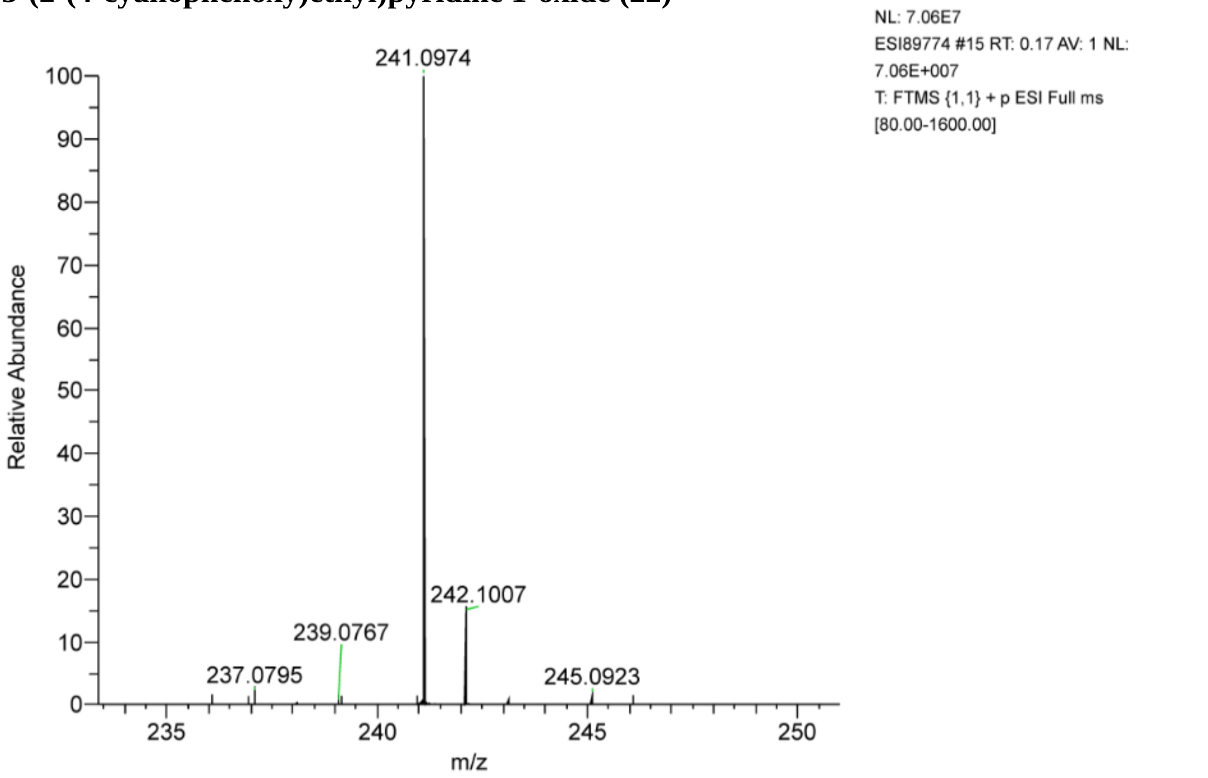

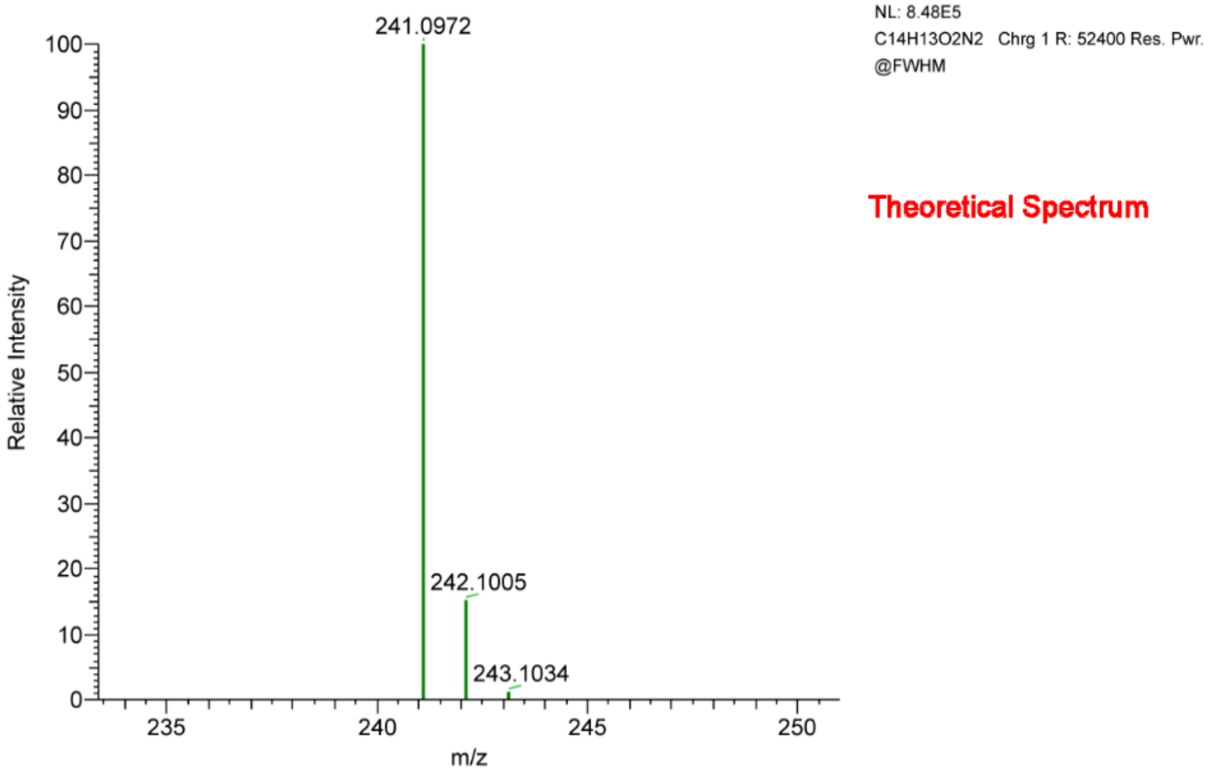

3-(2-(4-nitrophenoxy)ethyl)pyridine 1-oxide (23)

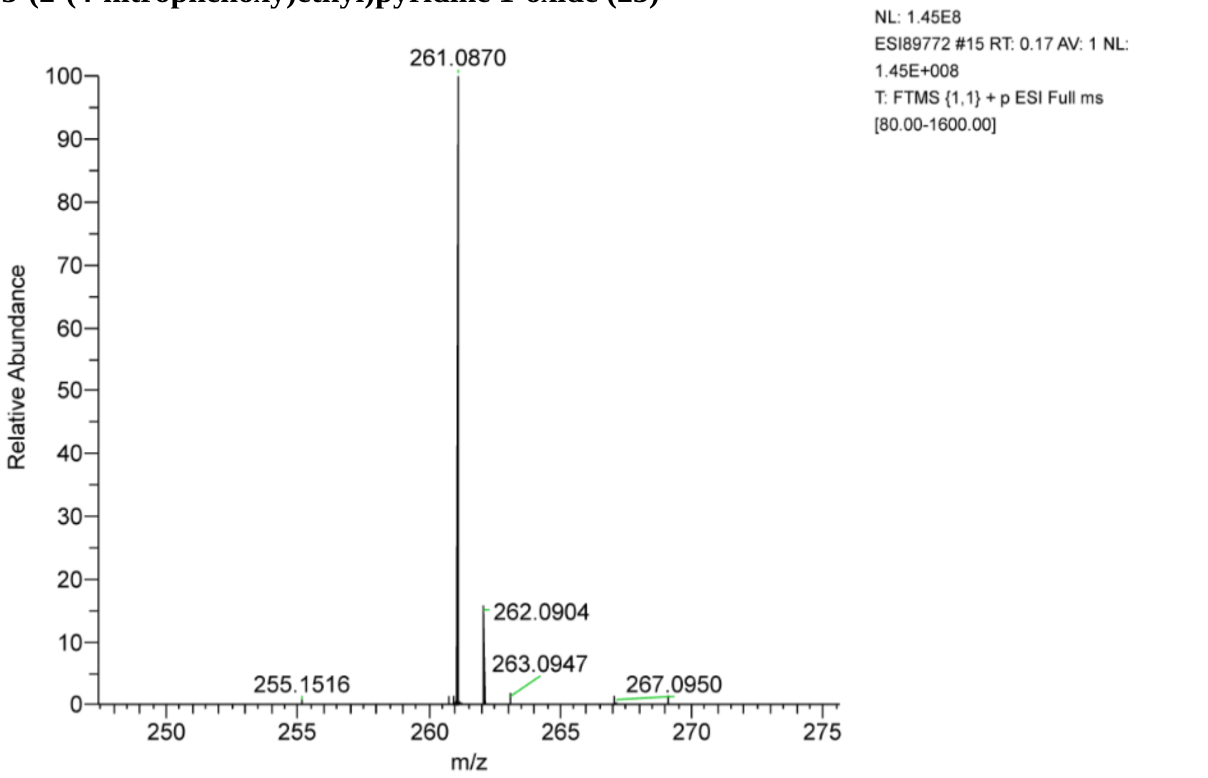

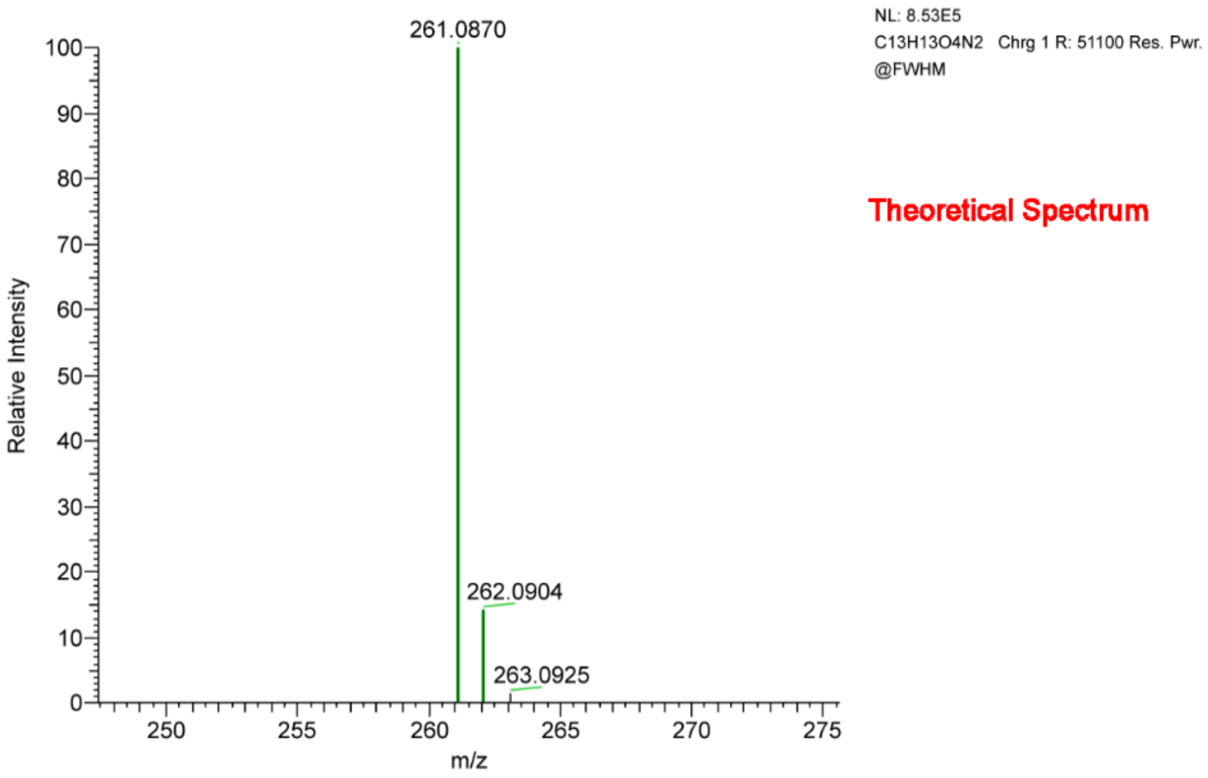

3-(2-(4-chloro-2,3-dimethylphenoxy)ethyl)pyridine 1-oxide (24)

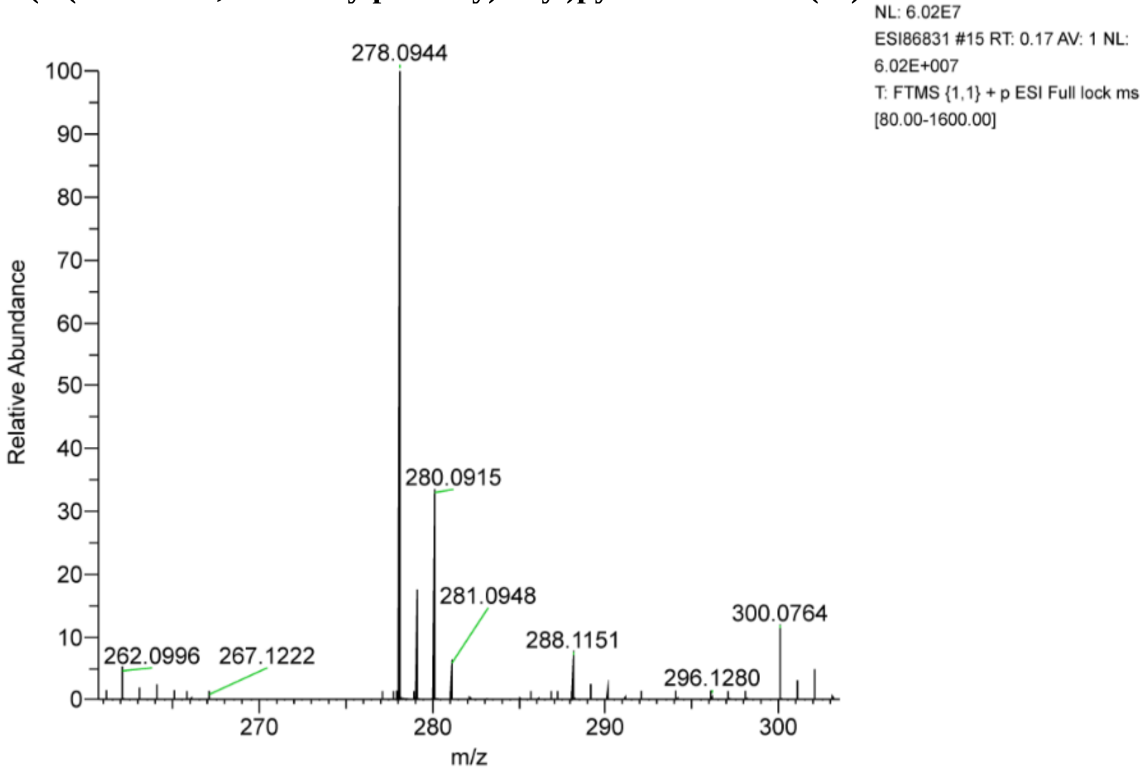

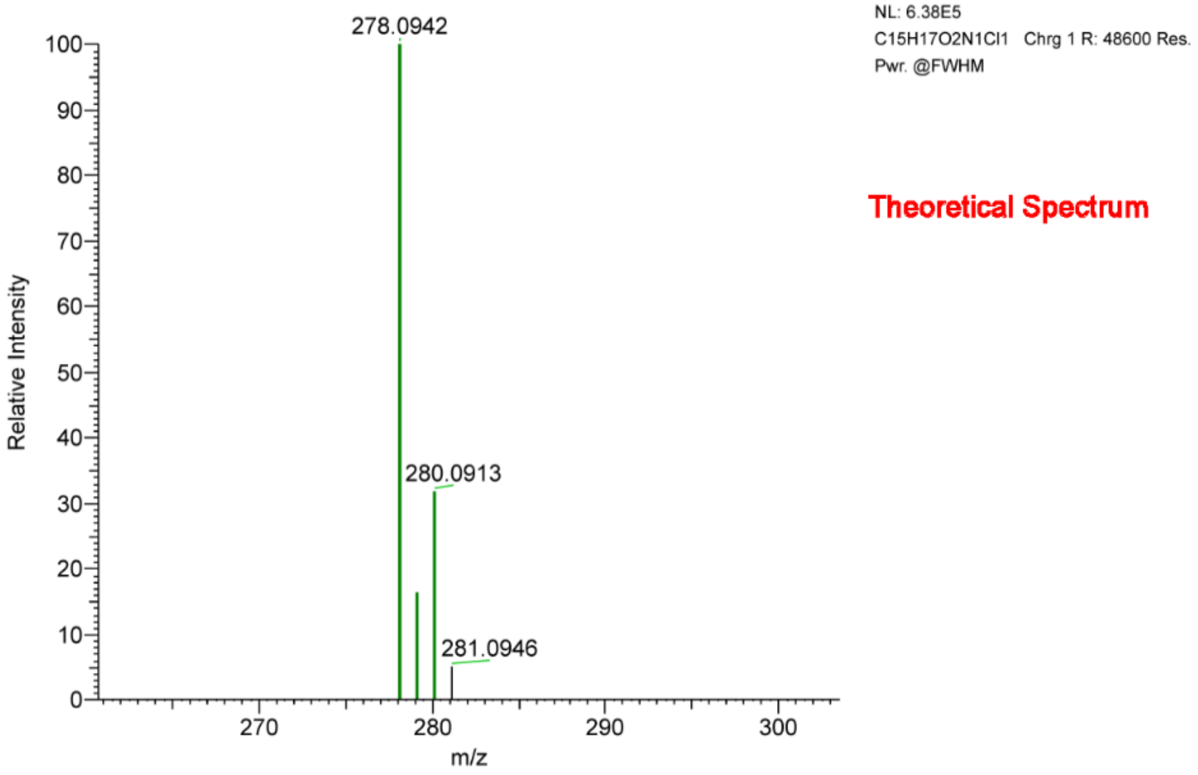

3-(2-(2,3-dichlorophenoxy)ethyl)pyridine 1-oxide (25)

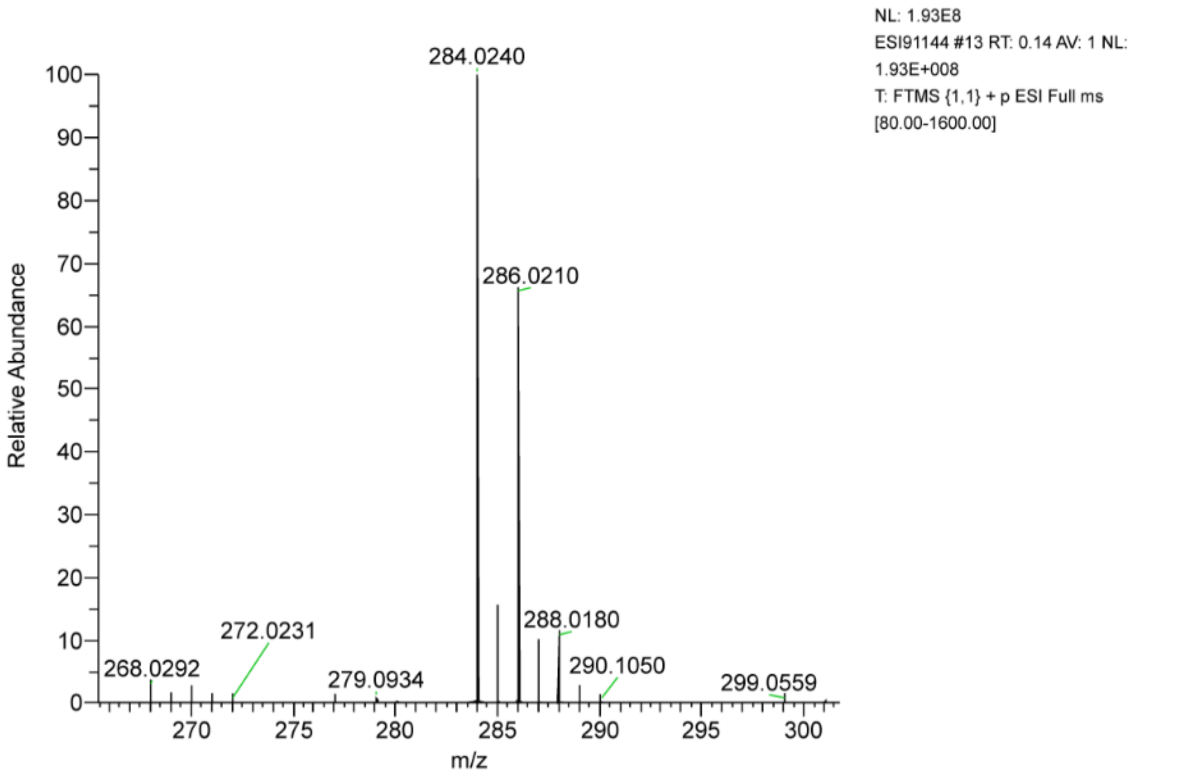

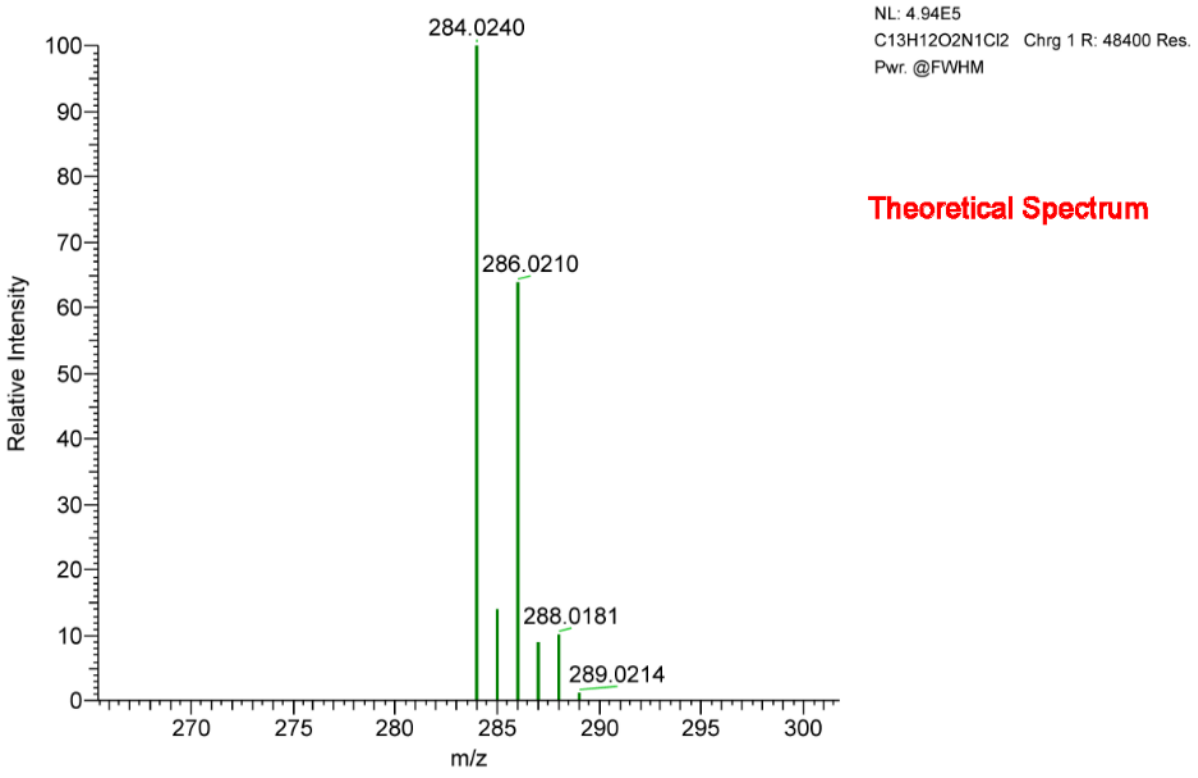

3-(2-(4-chloro-2-iodophenoxy)ethyl)pyridine 1-oxide (26)

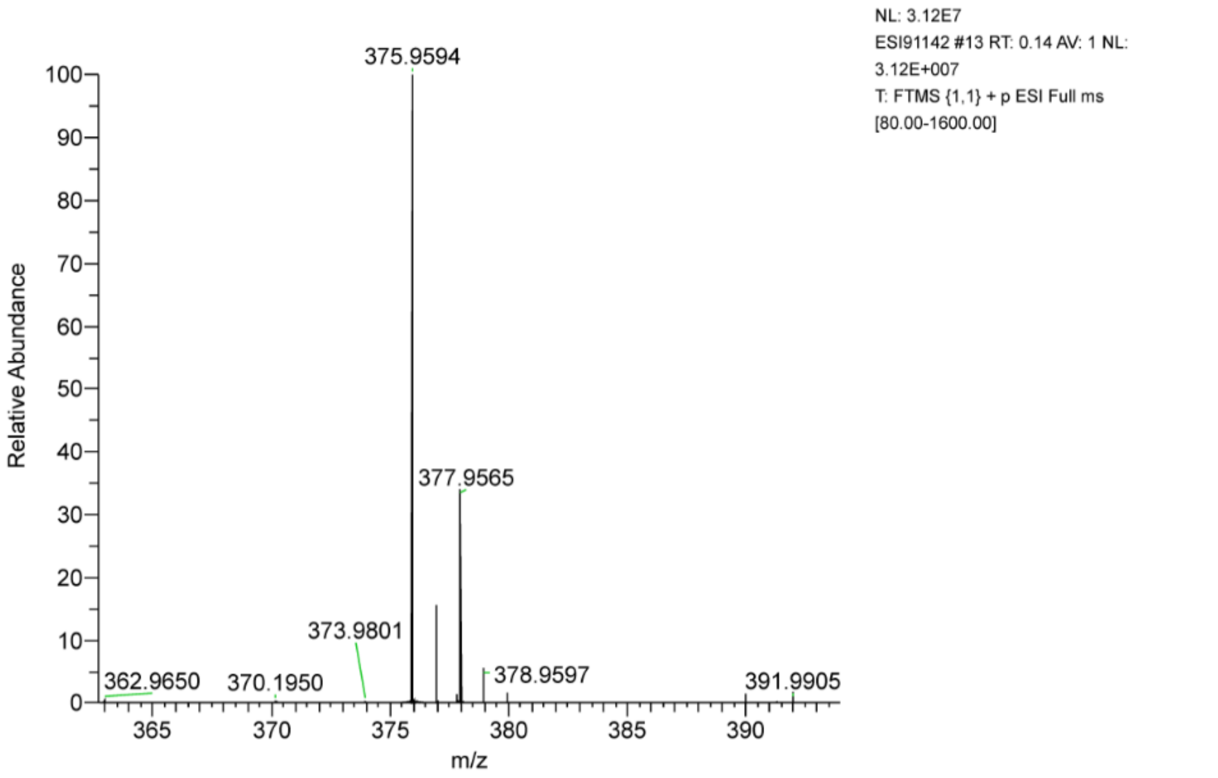

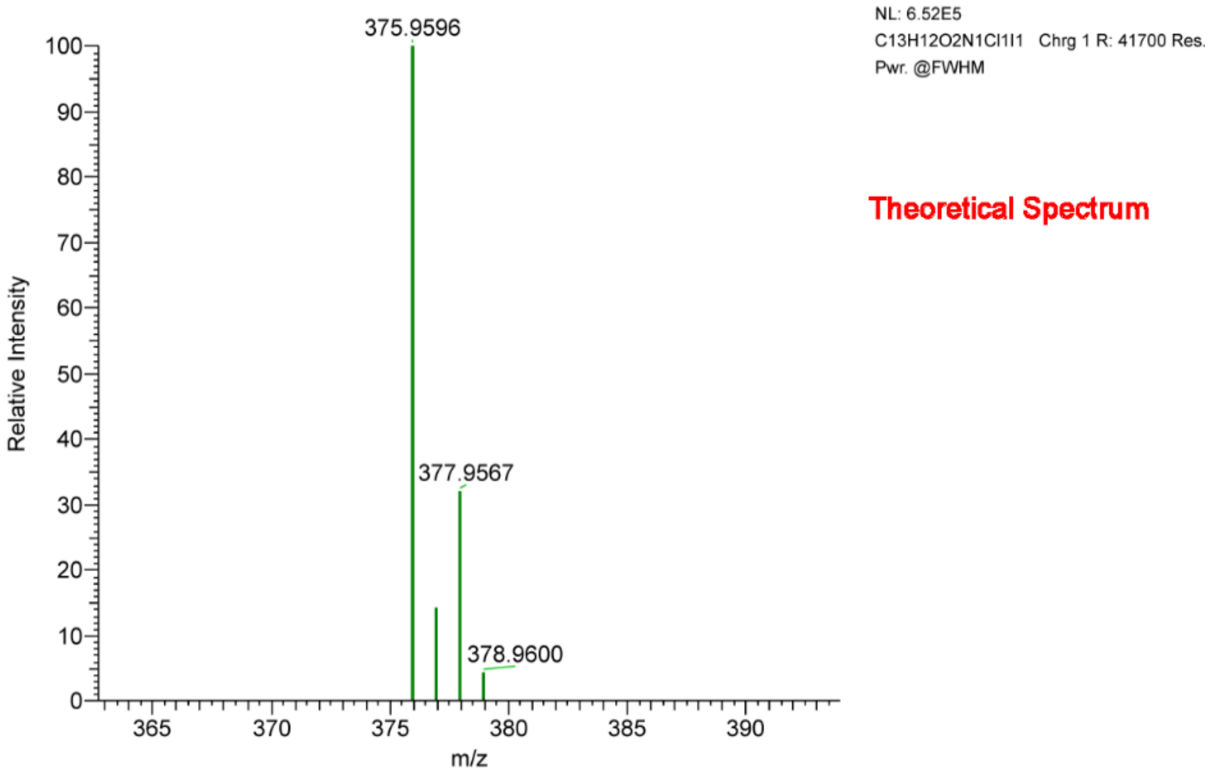

3-(2-(4-chloro-2-cyclohexylphenoxy)ethyl)pyridine 1-oxide (27)

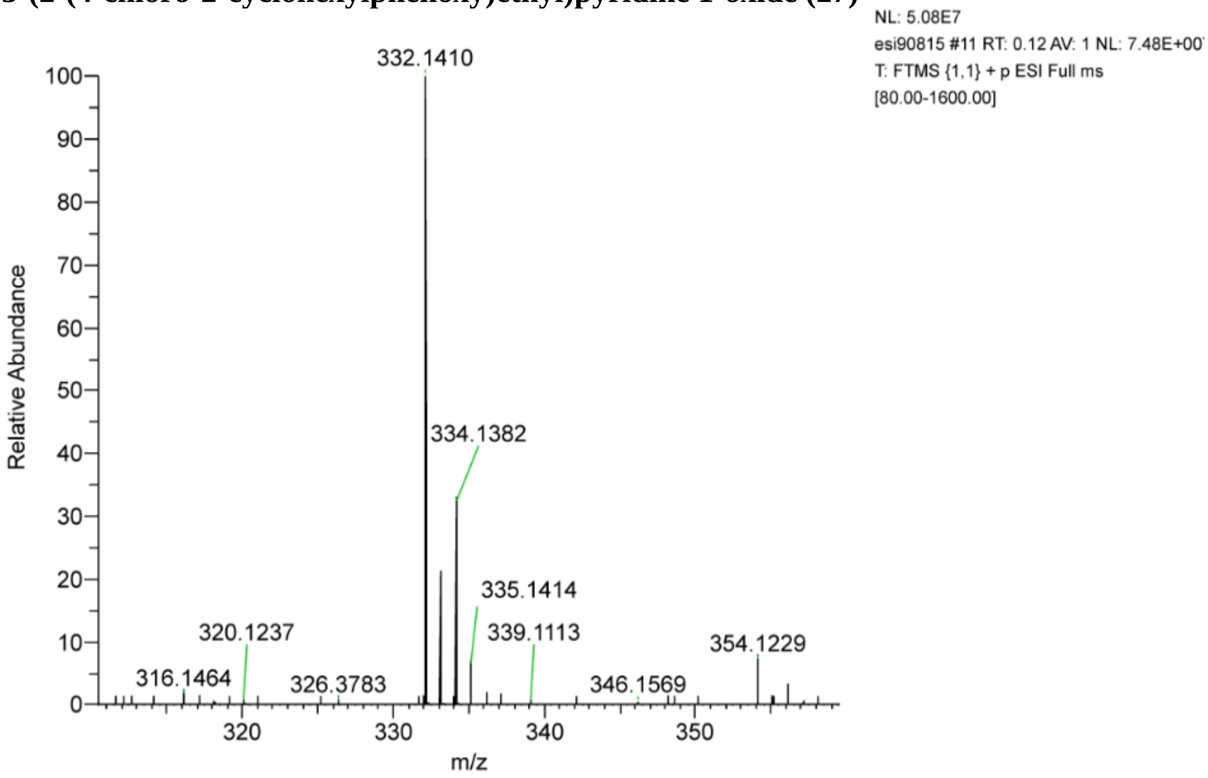

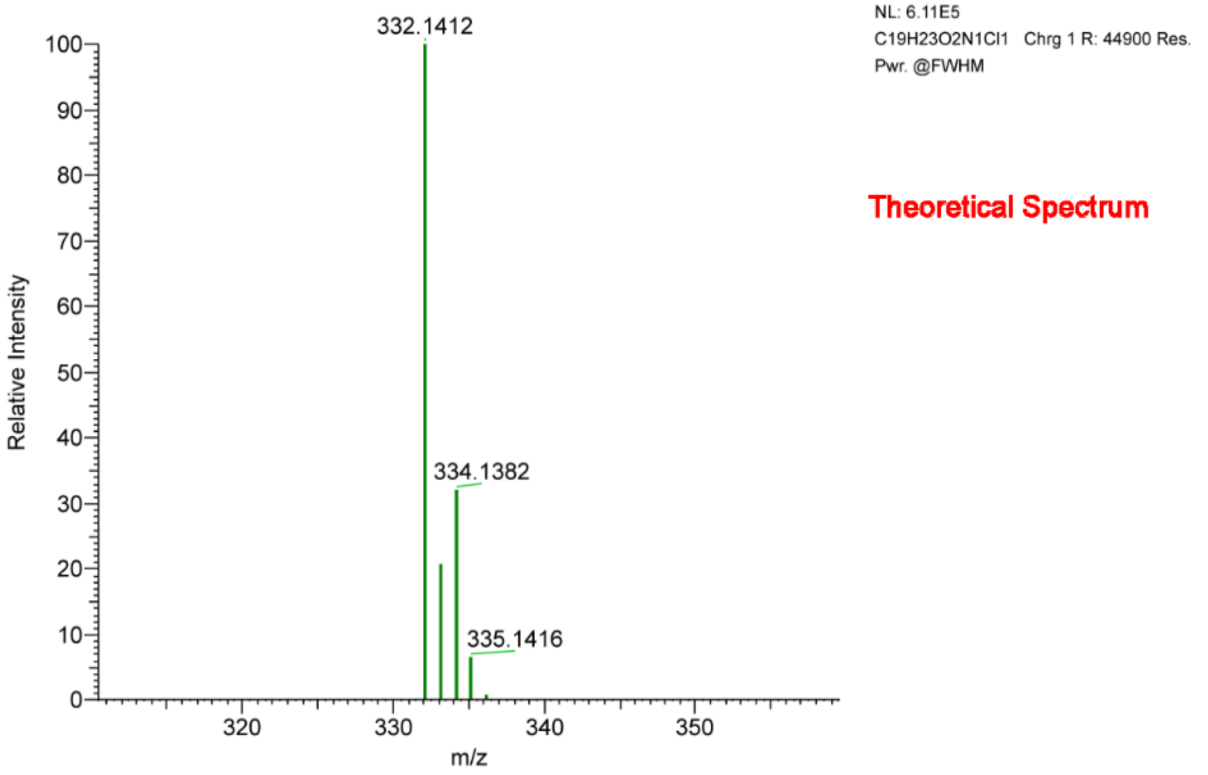

3-(2-((5-chloro-[1,1'-biphenyl]-2-yl)oxy)ethyl)pyridine 1-oxide (28)

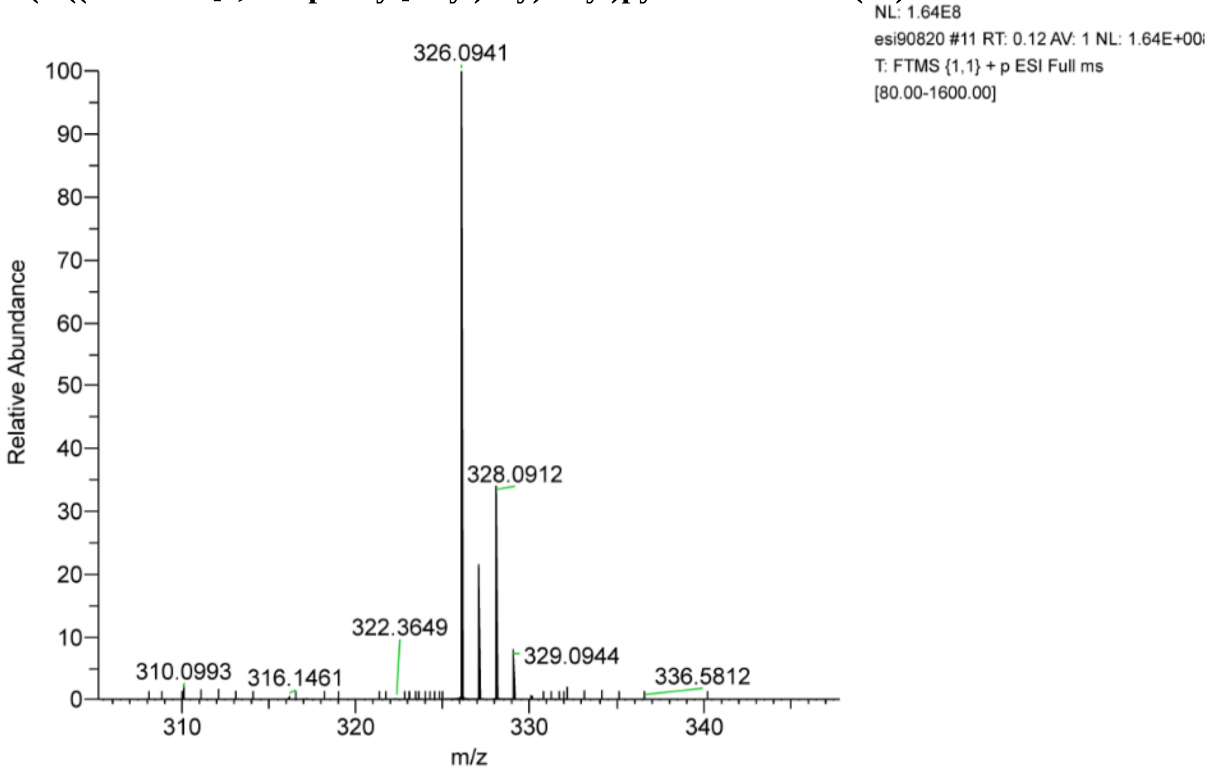

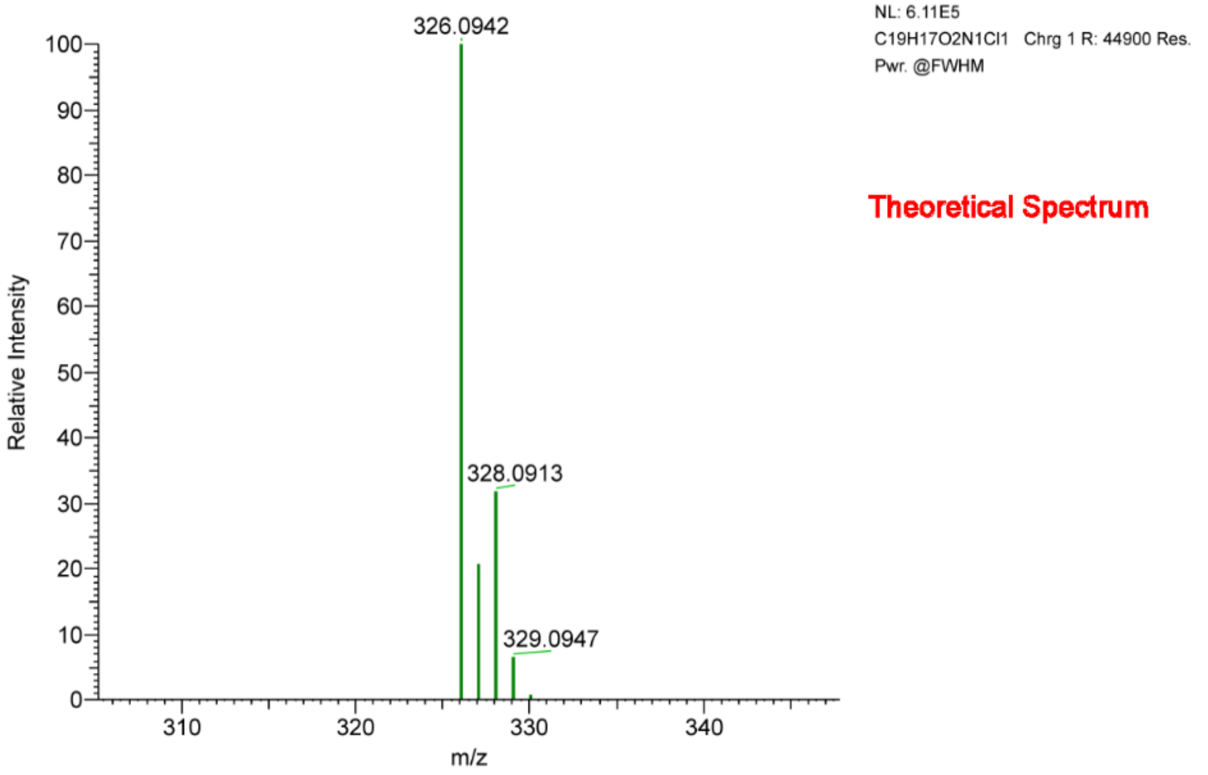

3-(2-(2,3,4-trichlorophenoxy)ethyl)pyridine 1-oxide (29)

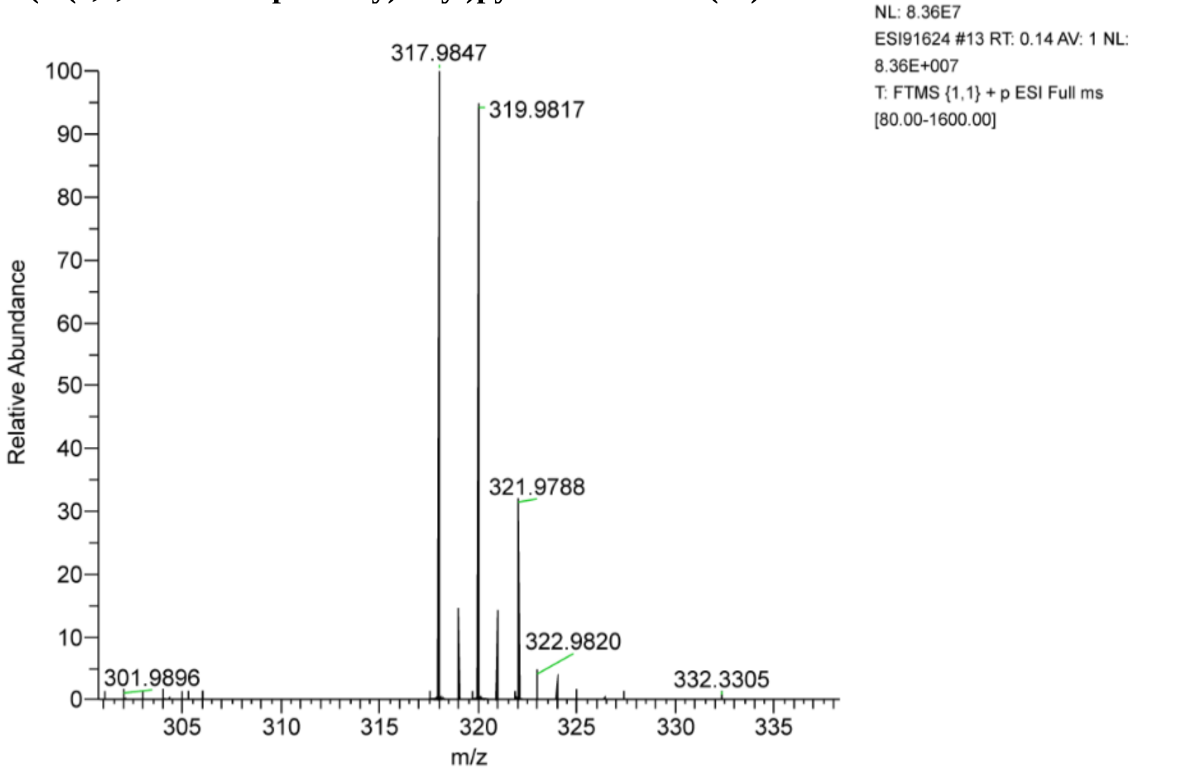

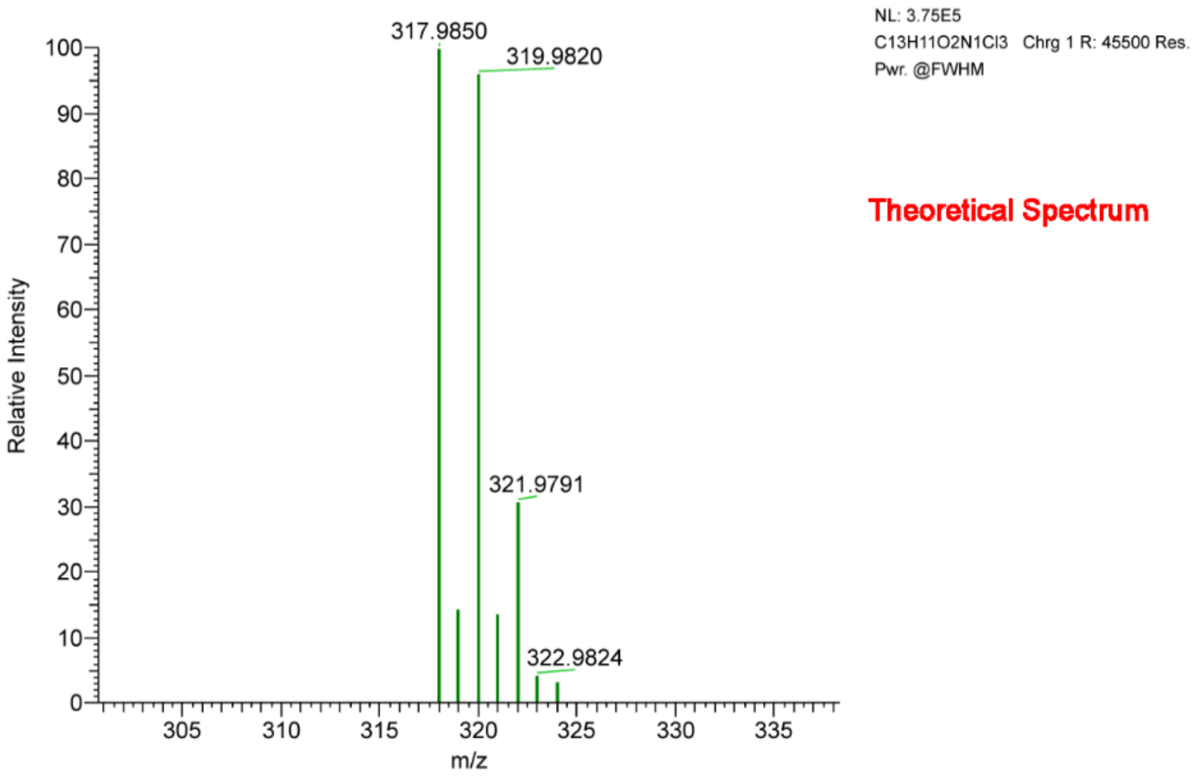

3-(2-(4-chloro-3,5-dimethylphenoxy)ethyl)pyridine 1-oxide (30)

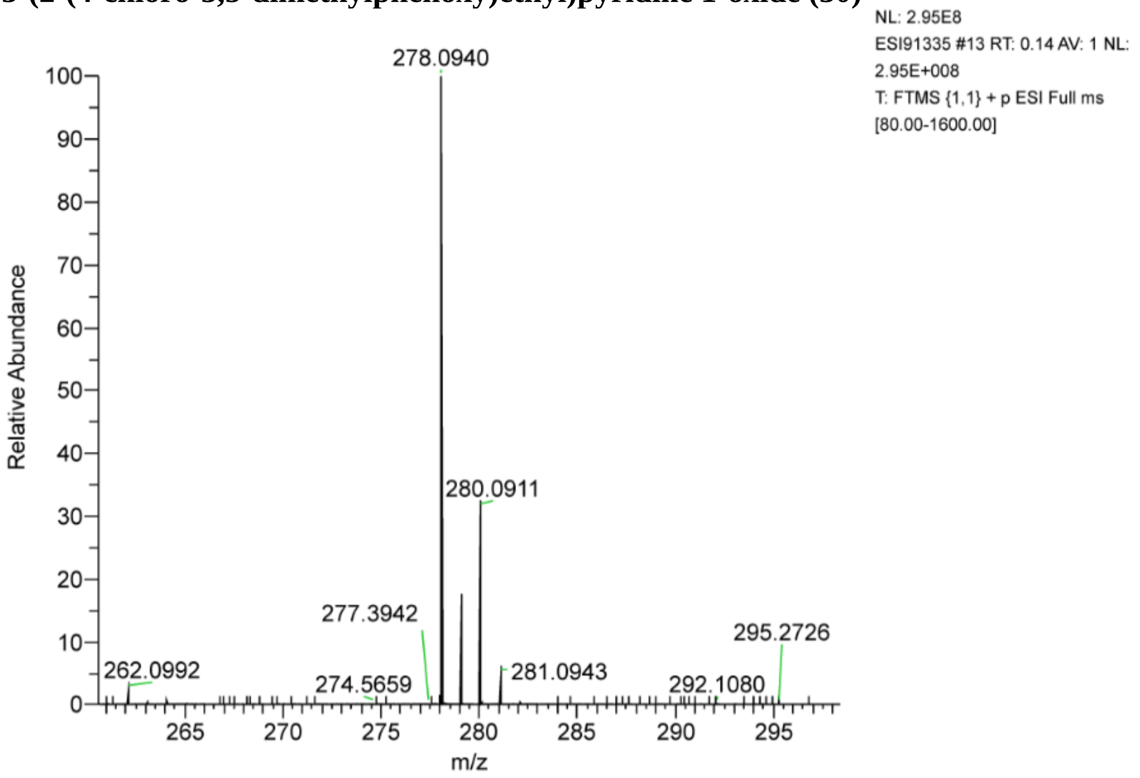

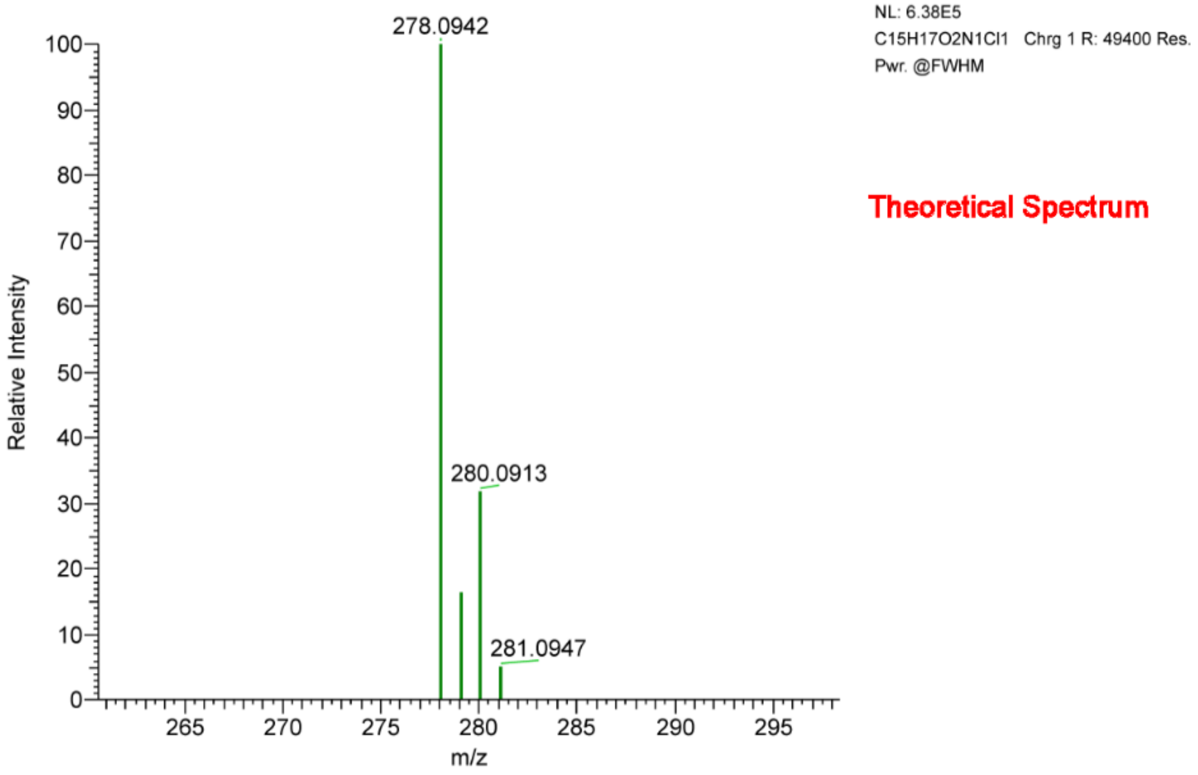

3-(2-(3,4,5-trimethylphenoxy)ethyl)pyridine 1-oxide (31)

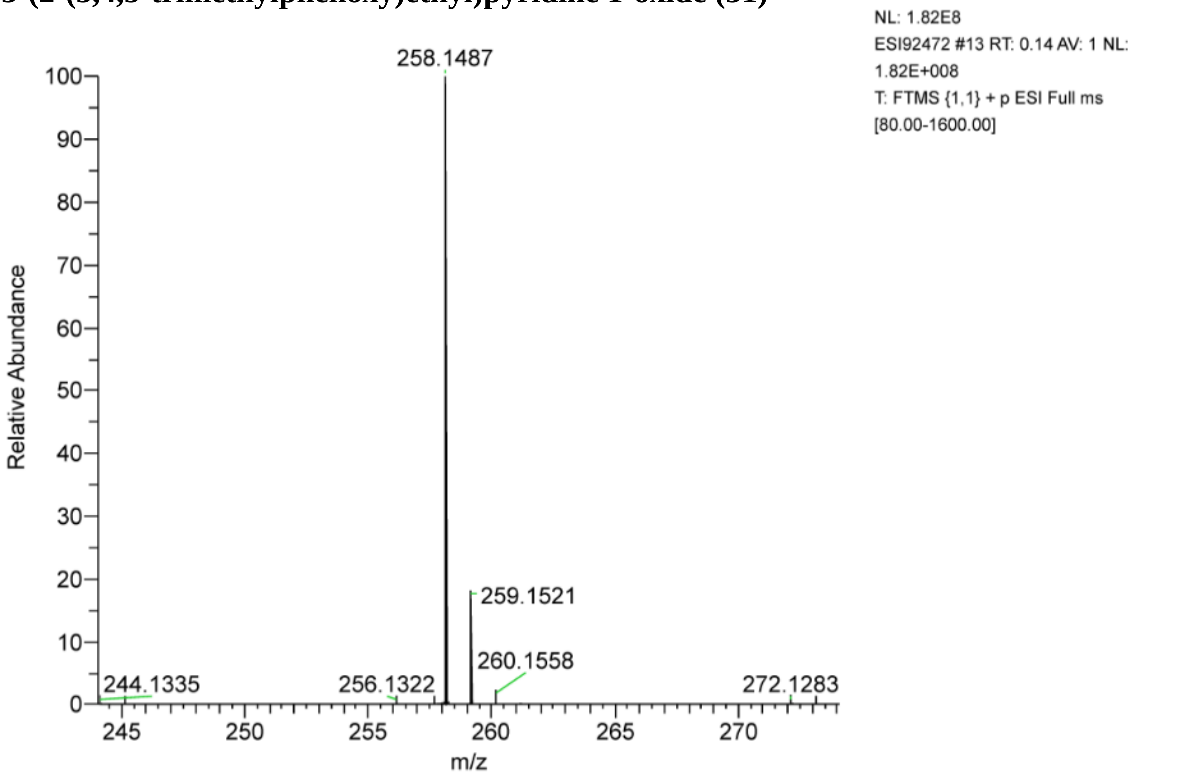

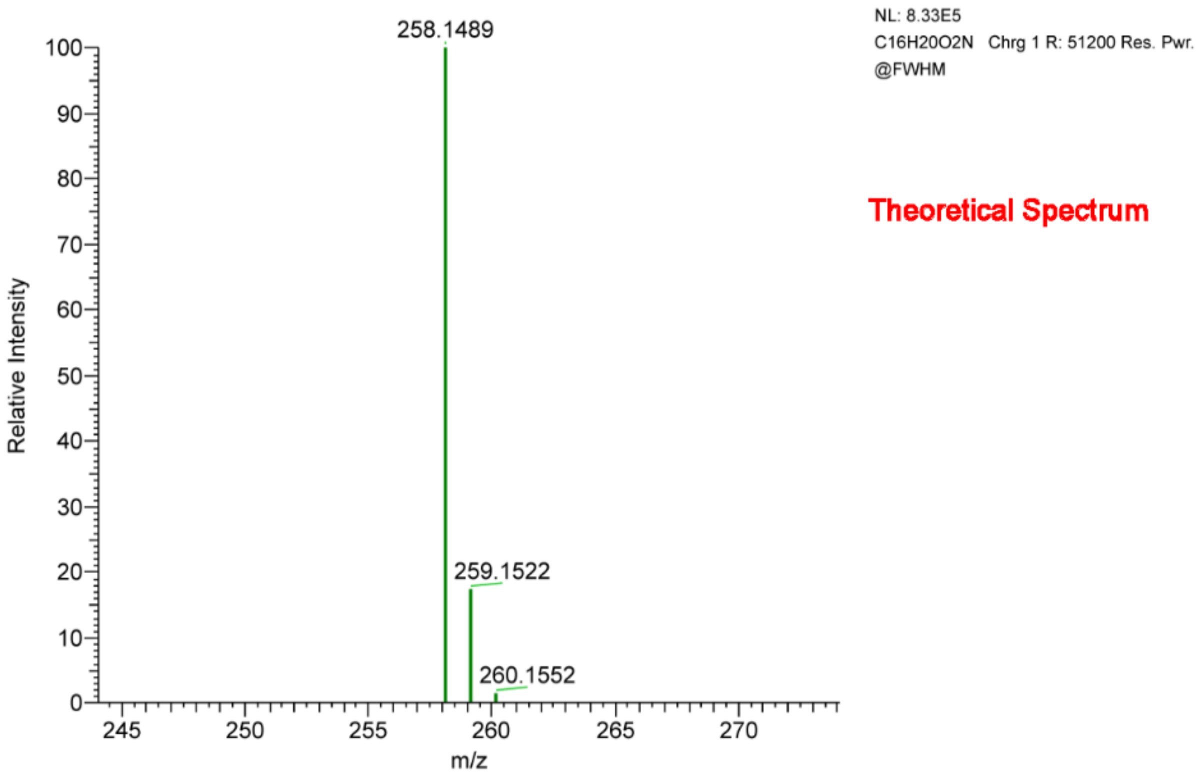

3-(2-(4-chloro-3-iodophenoxy)ethyl)pyridine 1-oxide (32)

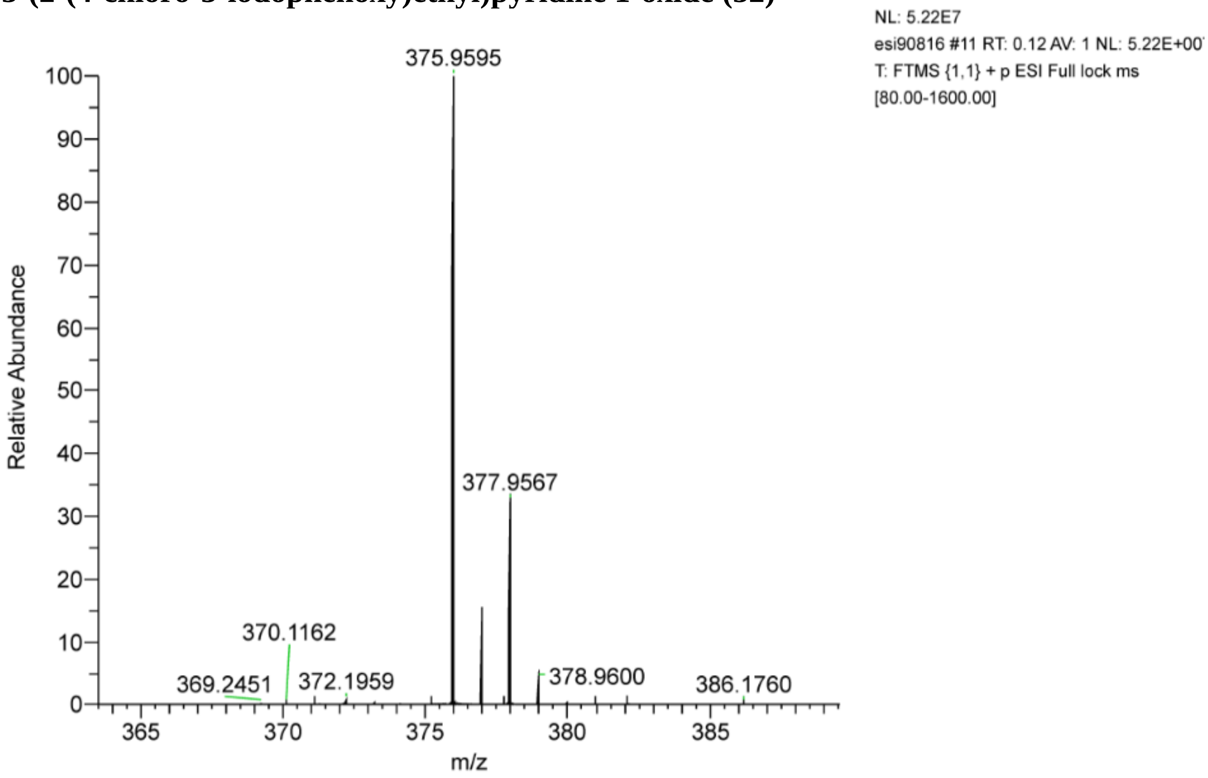

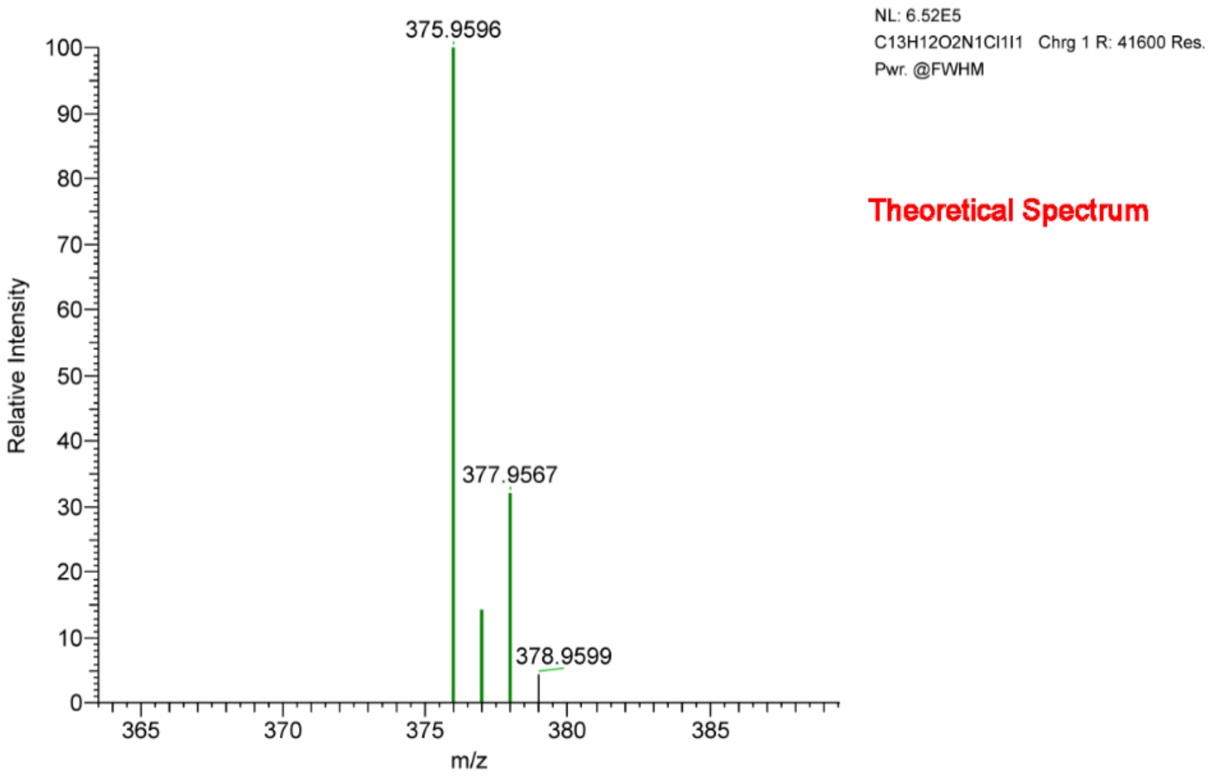

3-(2-(3-bromo-4-chlorophenoxy)ethyl)pyridine 1-oxide (33)

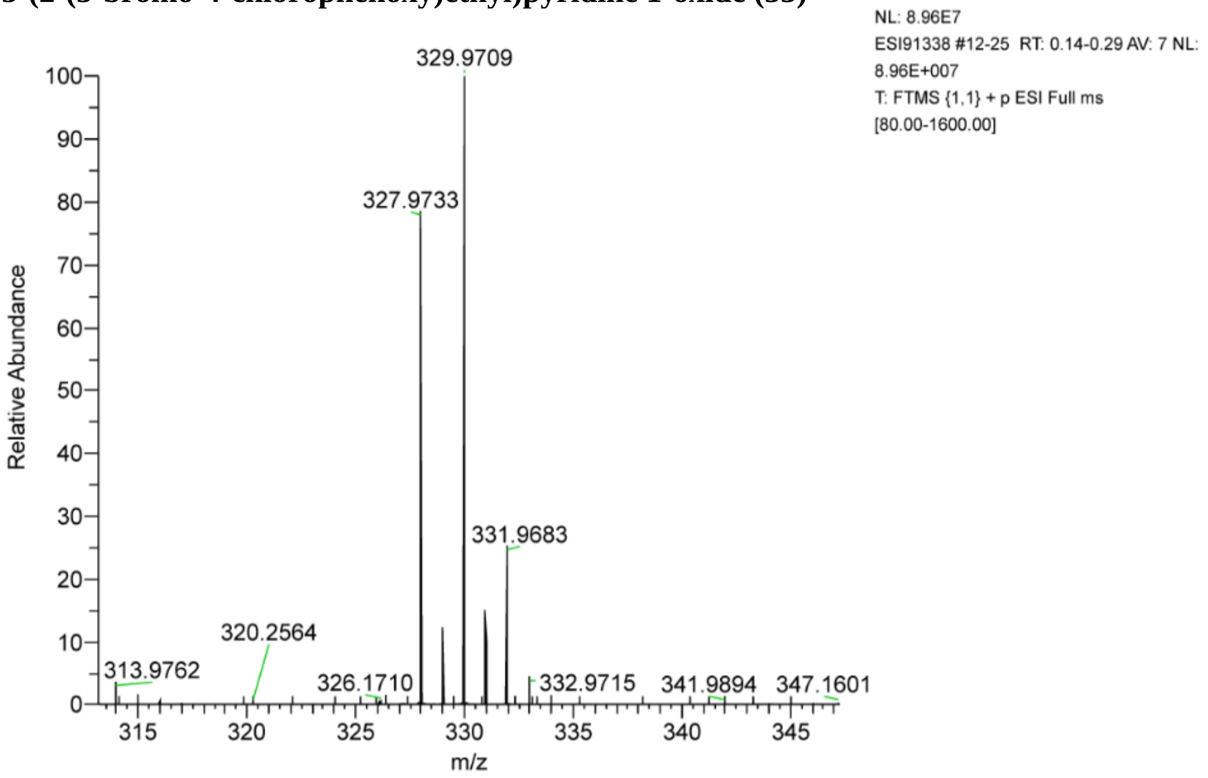

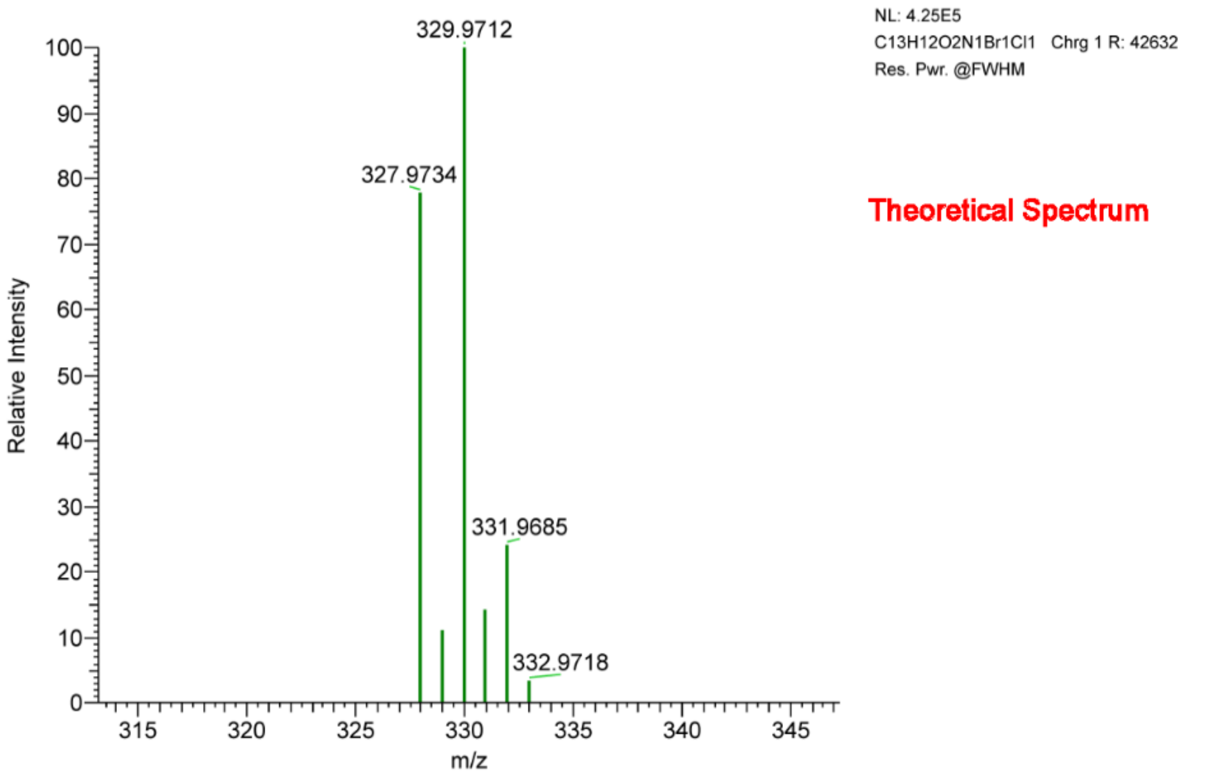

3-(2-(3,4-dichlorophenoxy)ethyl)pyridine 1-oxide (34)

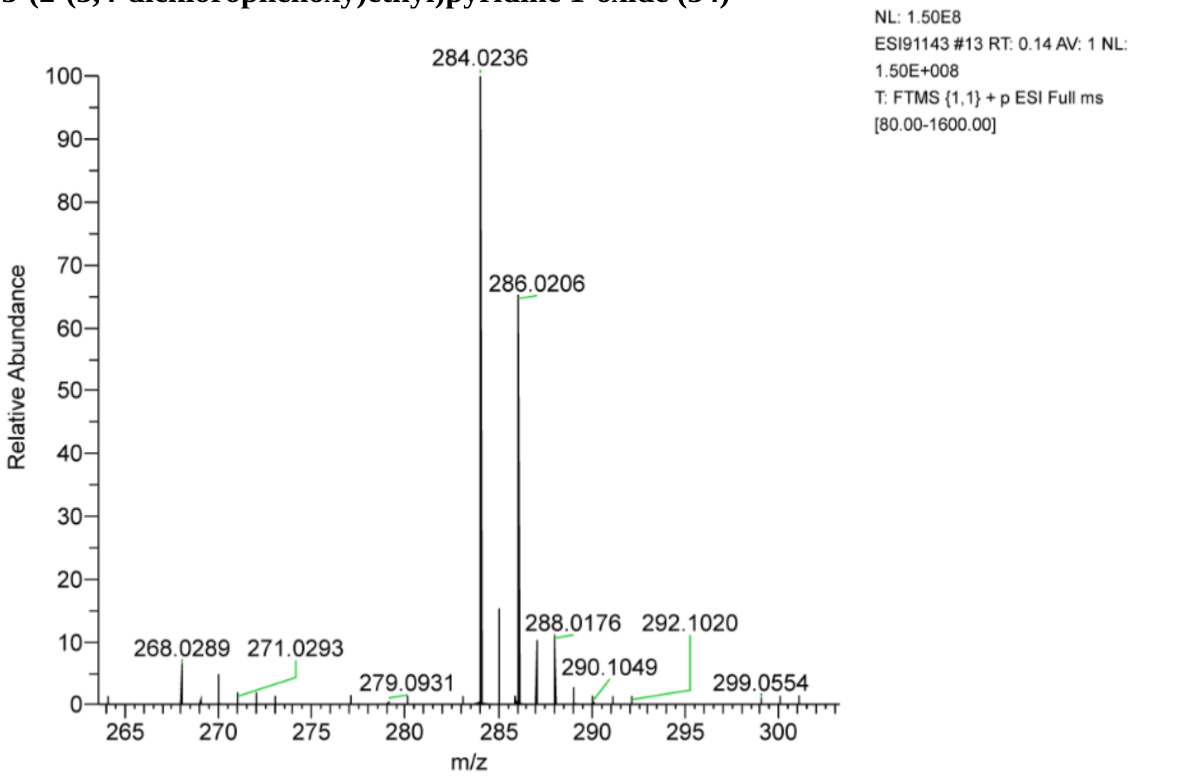

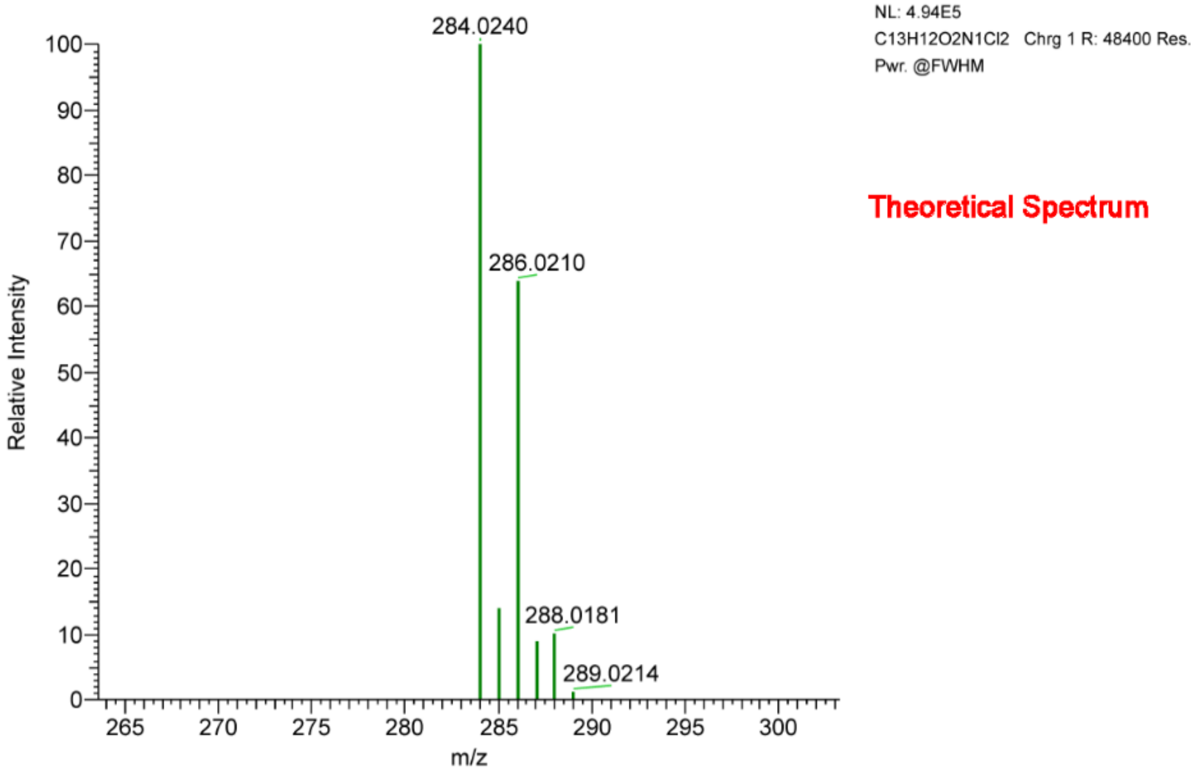

3-(2-(4-chloro-3-fluorophenoxy)ethyl)pyridine 1-oxide (35)

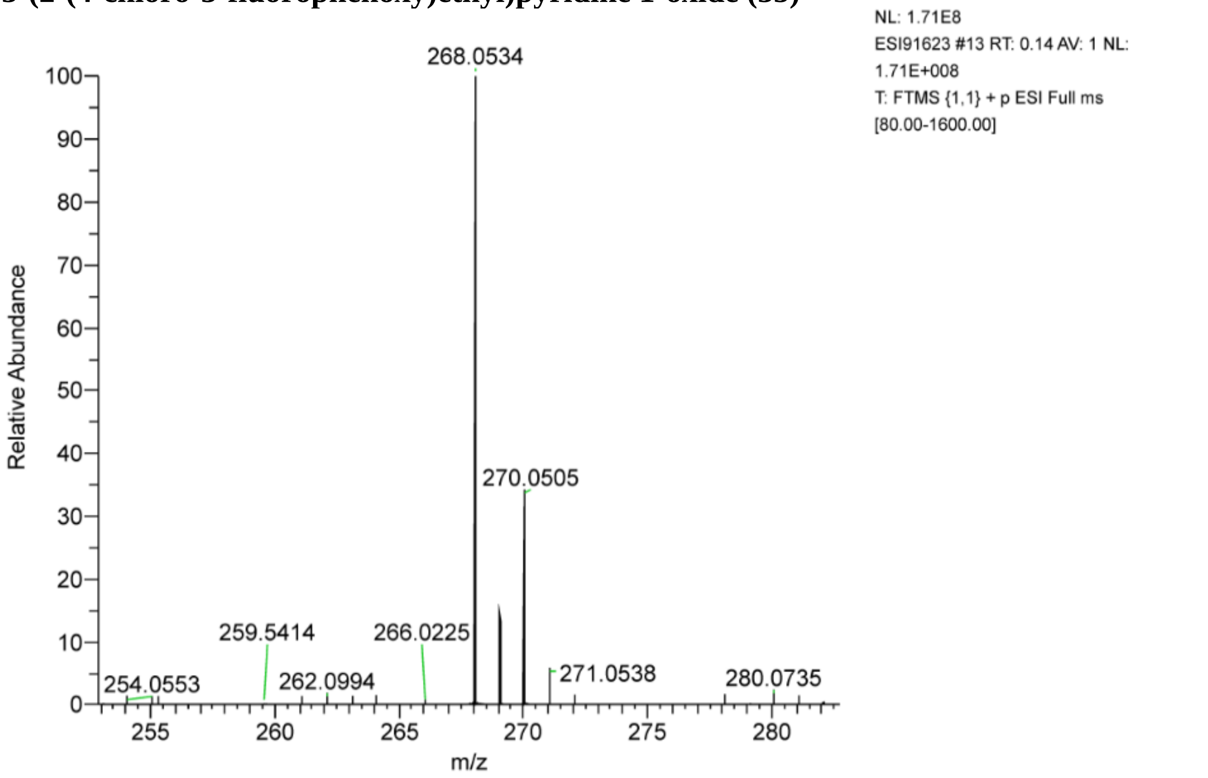

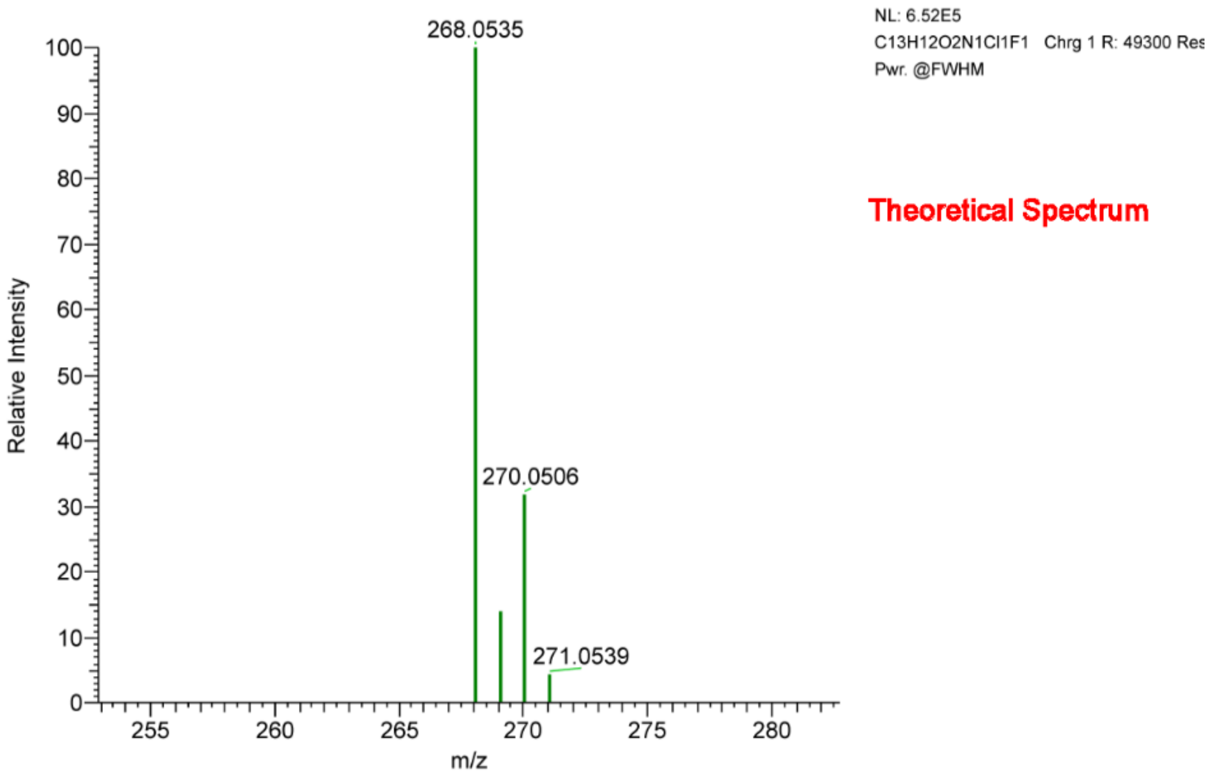

3-(2-((6-chloro-[1,1'-biphenyl]-3-yl)oxy)ethyl)pyridine 1-oxide (36)

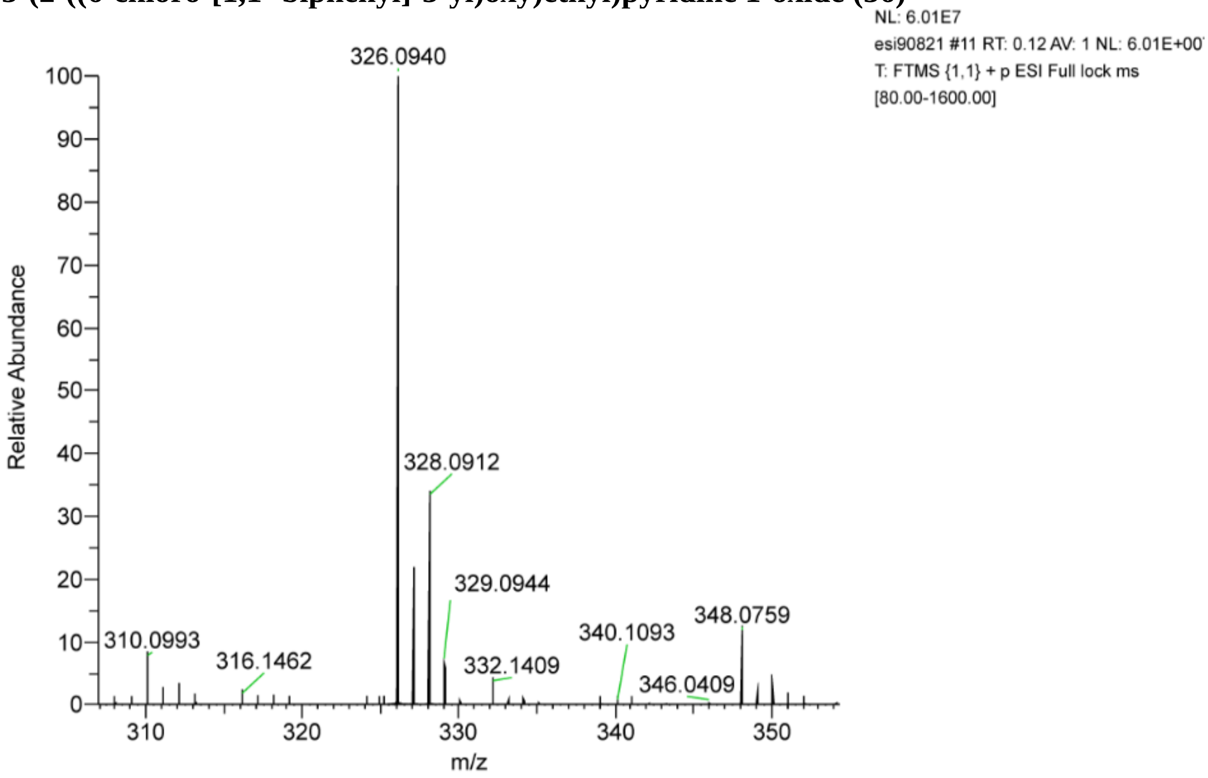

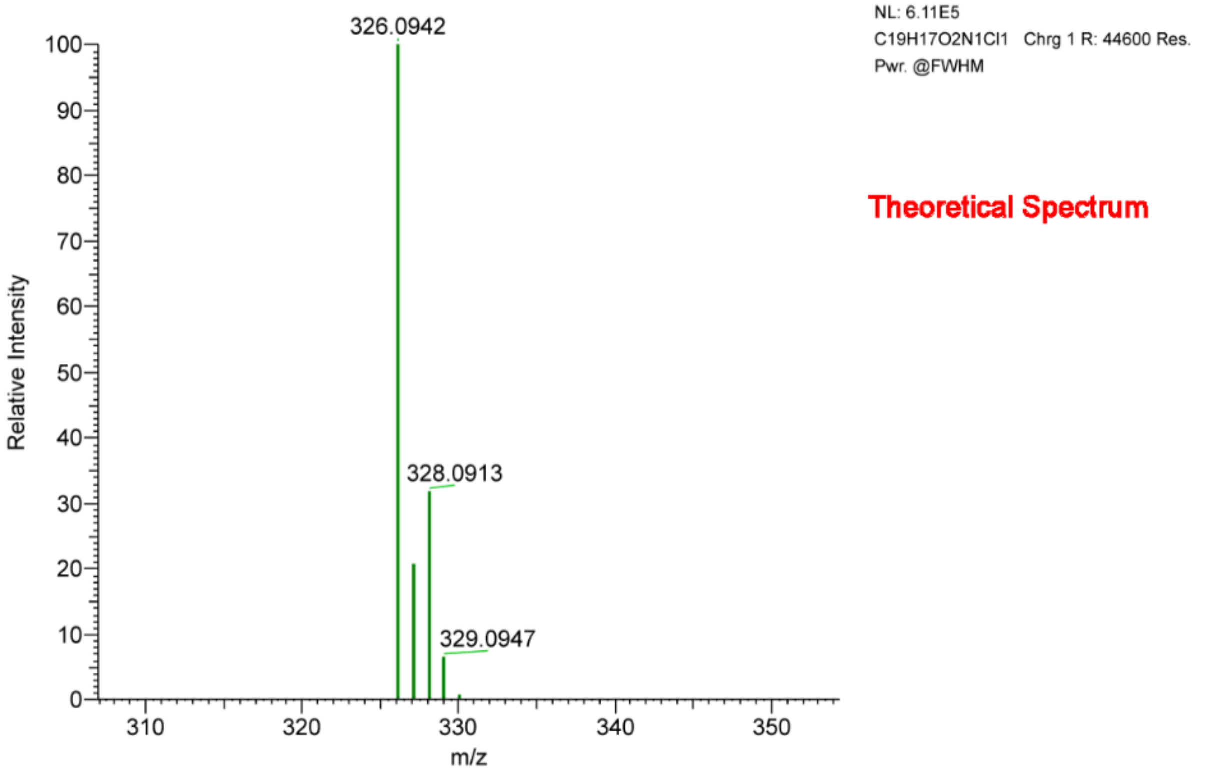

3-(2-(4-chloro-3-methylphenoxy)ethyl)pyridine 1-oxide (37)

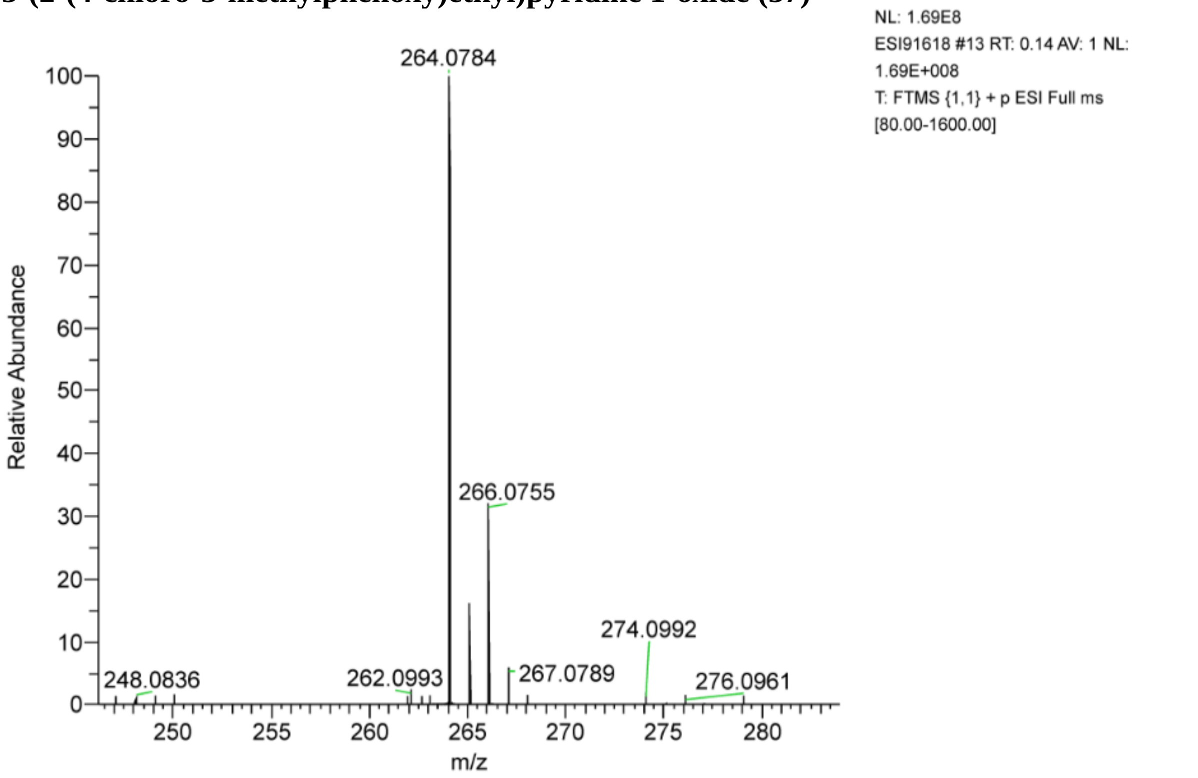

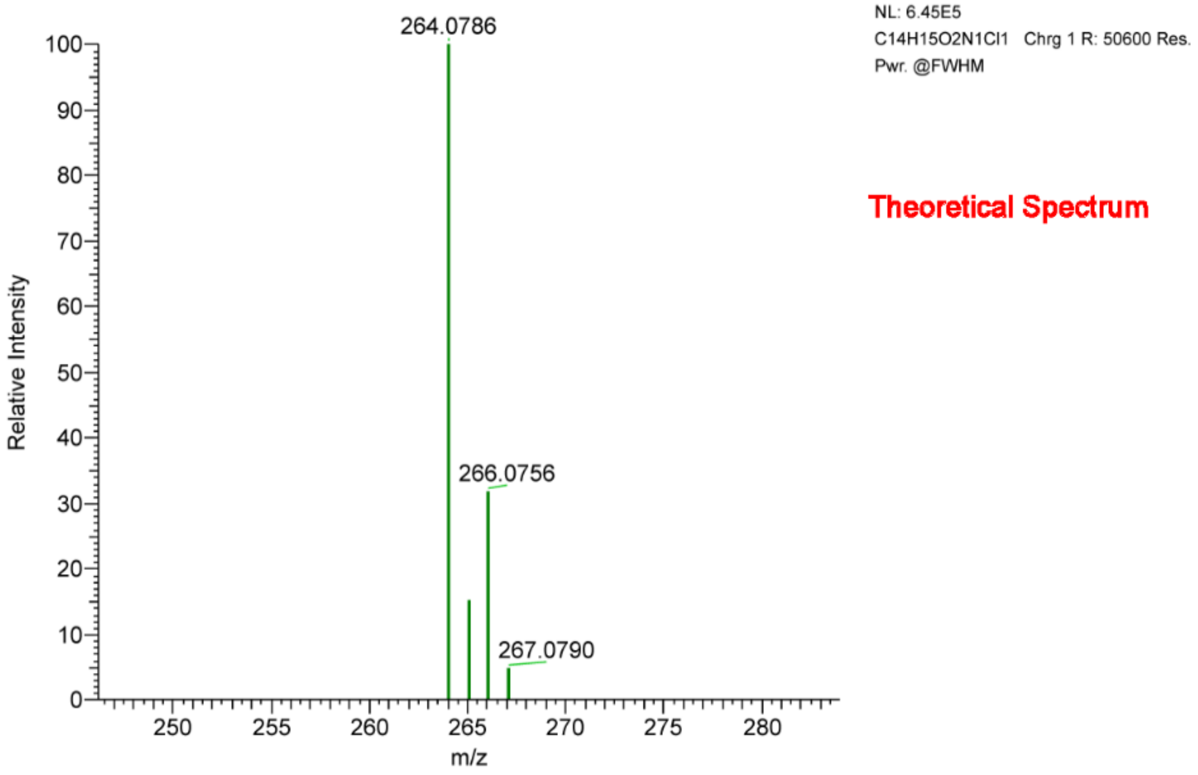

3-(2-(4-chloro-3-nitrophenoxy)ethyl)pyridine 1-oxide (38)

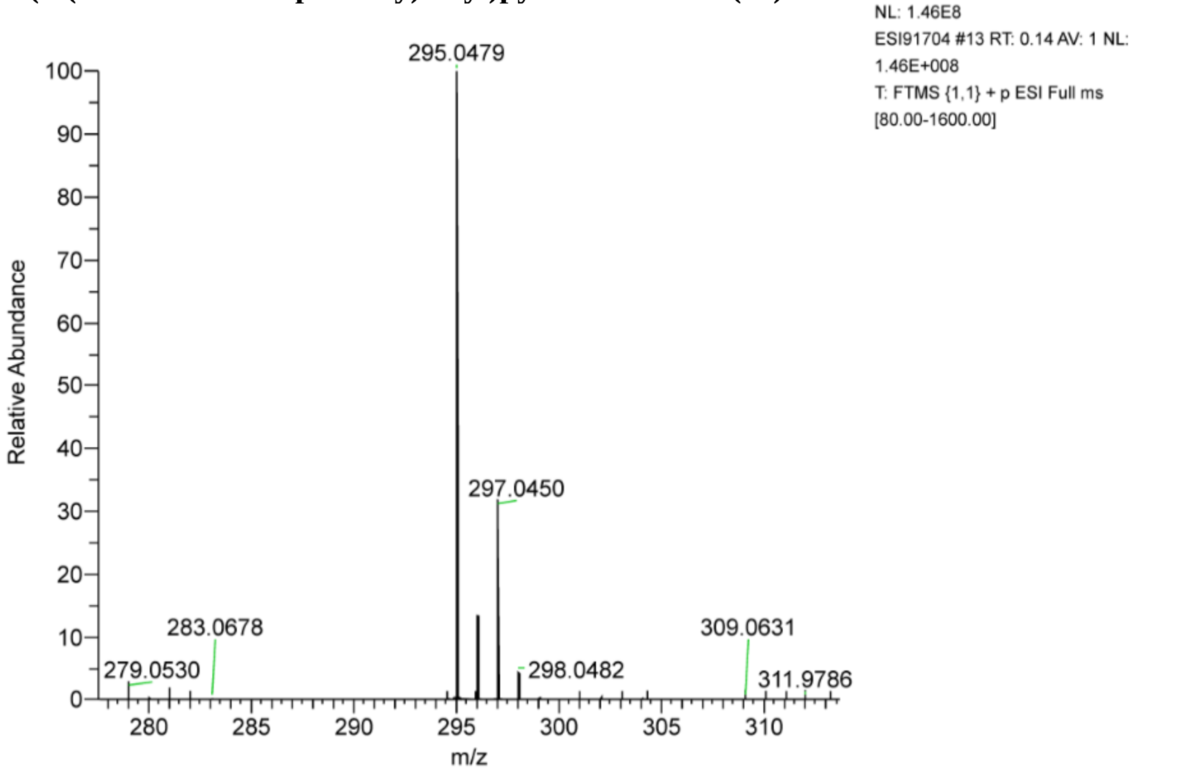

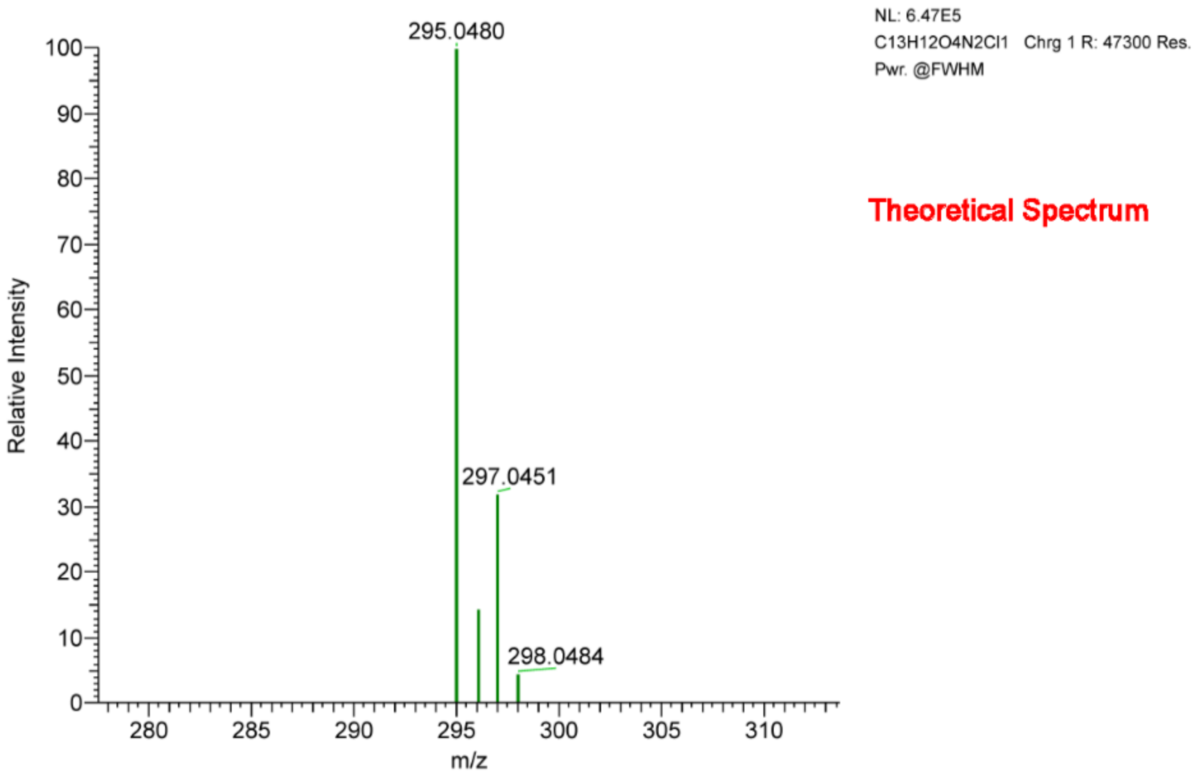

3-(2-(4-chloro-3-ethylphenoxy)ethyl)pyridine 1-oxide (39)

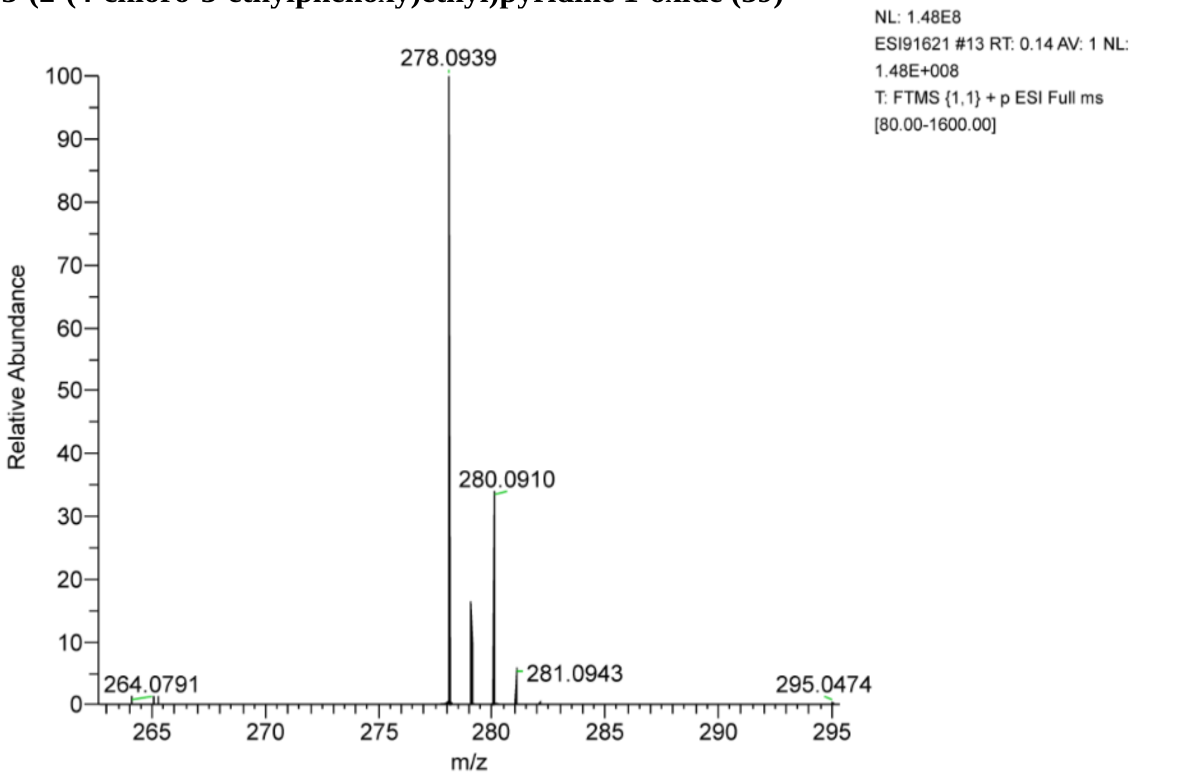

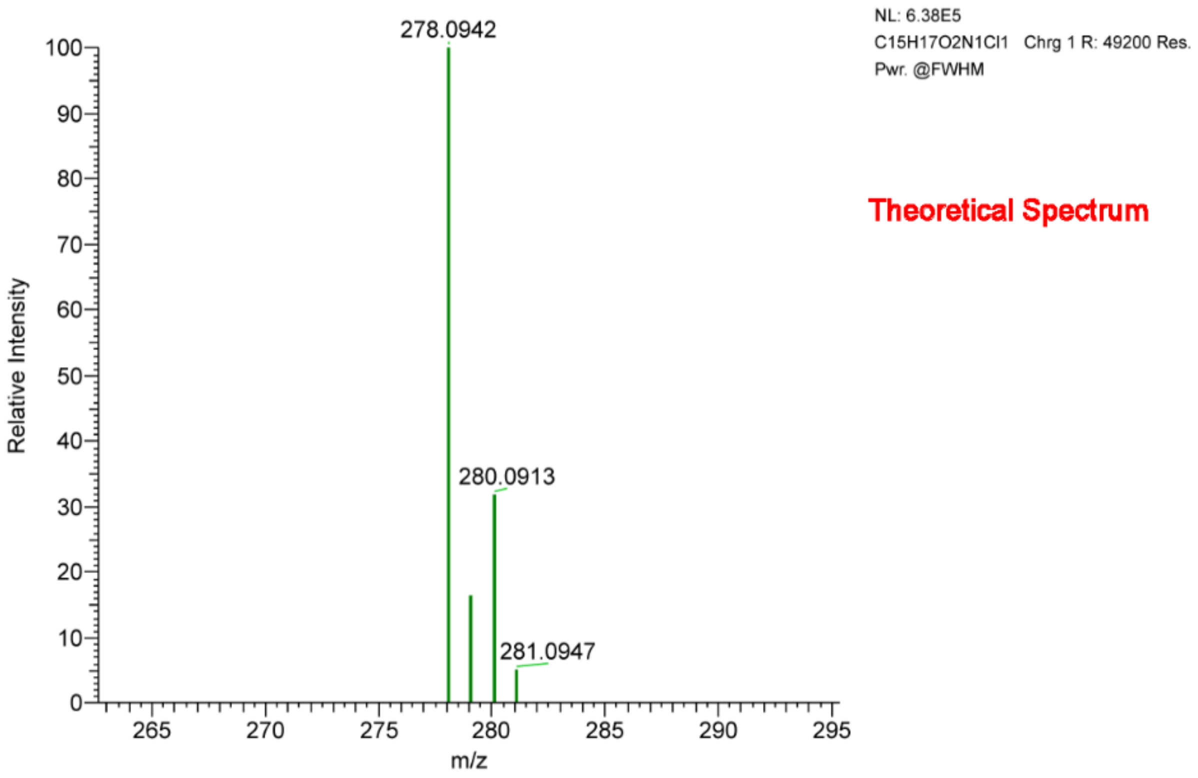

3-(2-(4-chloro-3-methoxyphenoxy)ethyl)pyridine 1-oxide (40)

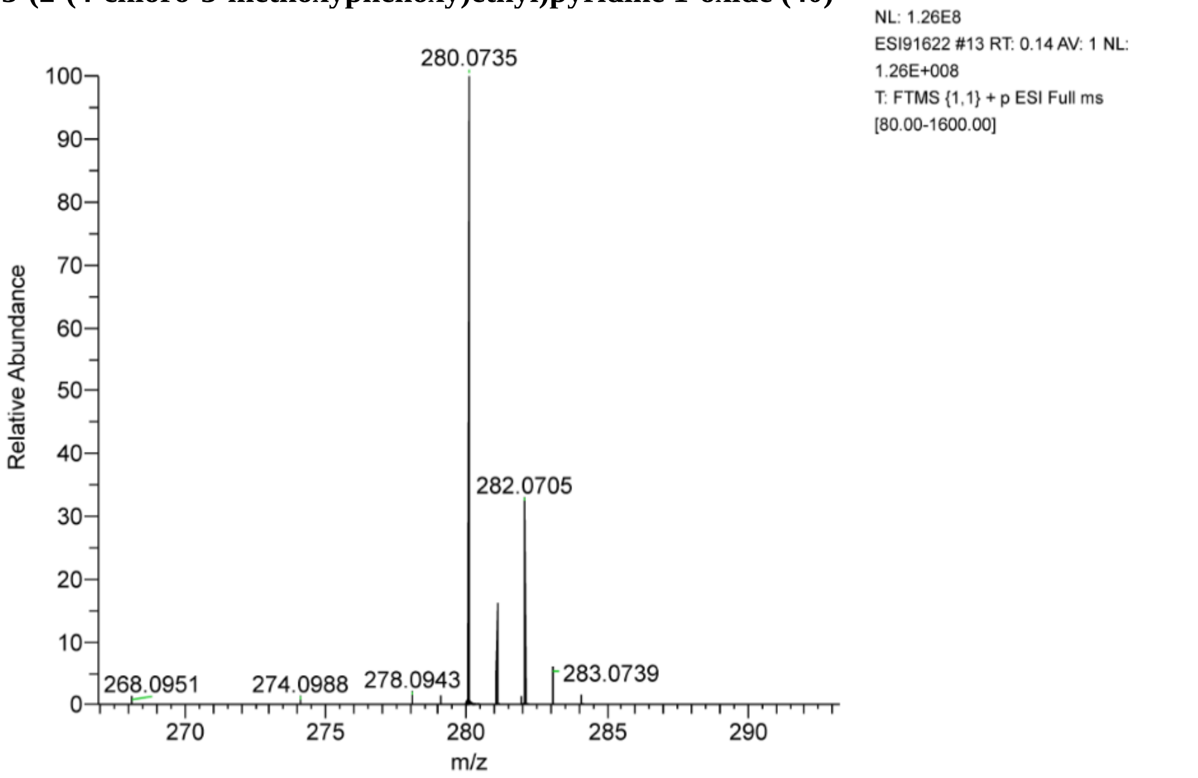

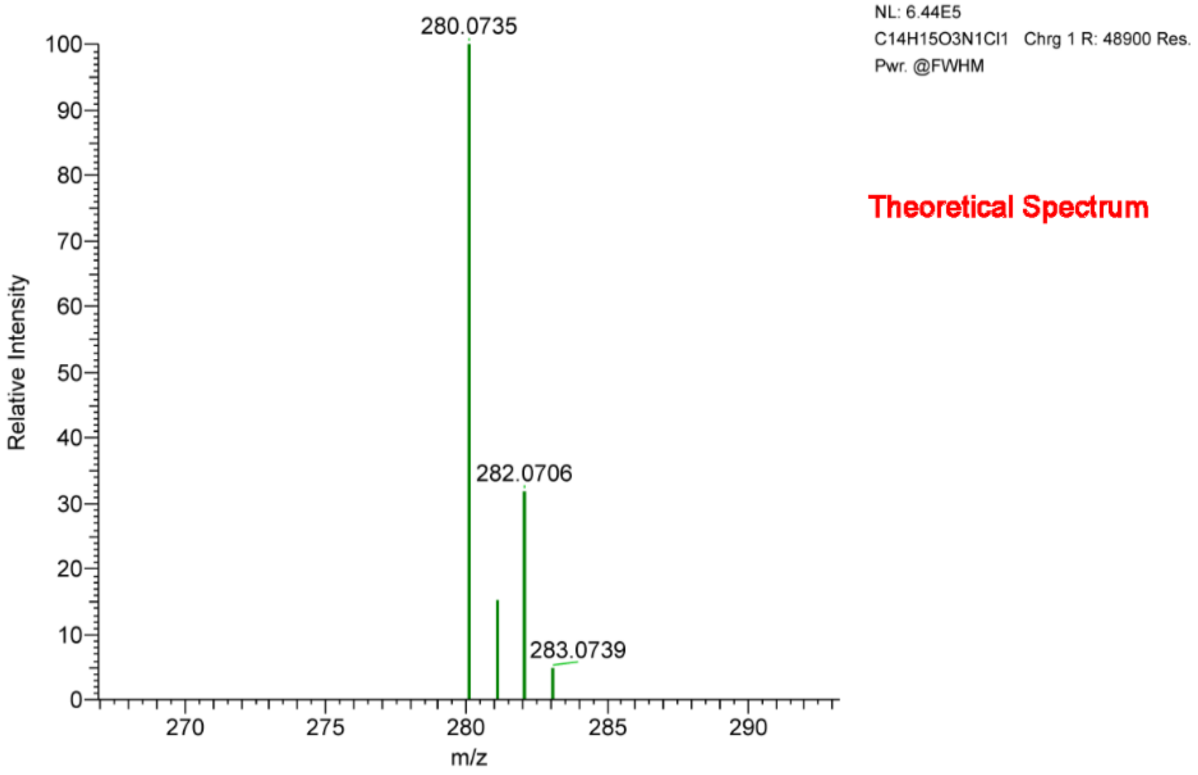

3-(2-(4-chloro-3-cyclopropylphenoxy)ethyl)pyridine 1-oxide (41)

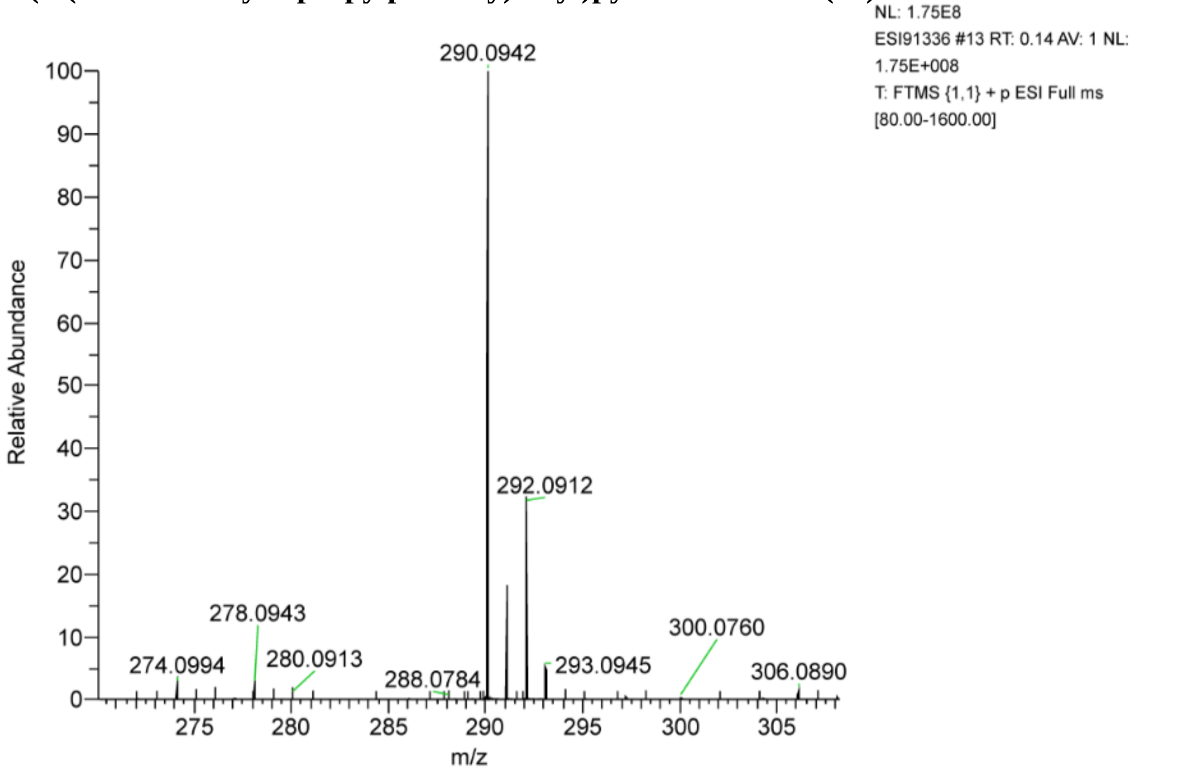

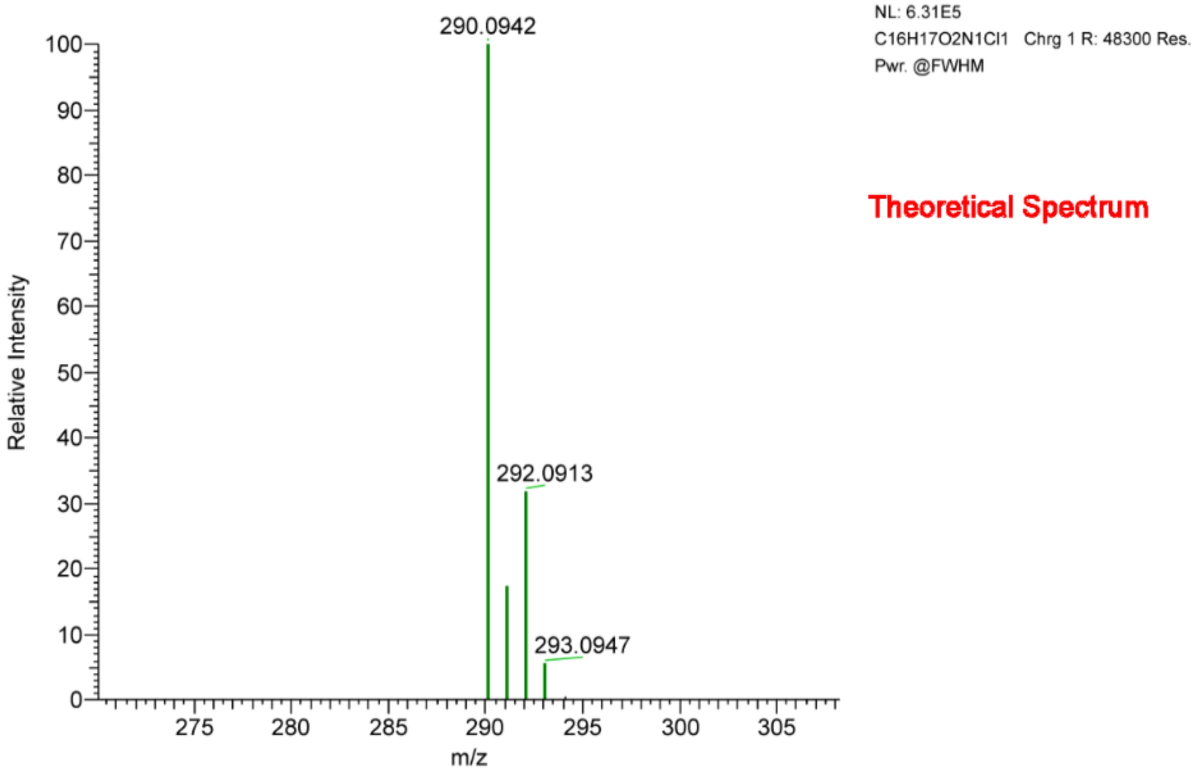

3-(2-(4-chloro-3-(trifluoromethyl)phenoxy)ethyl)pyridine 1-oxide (42)

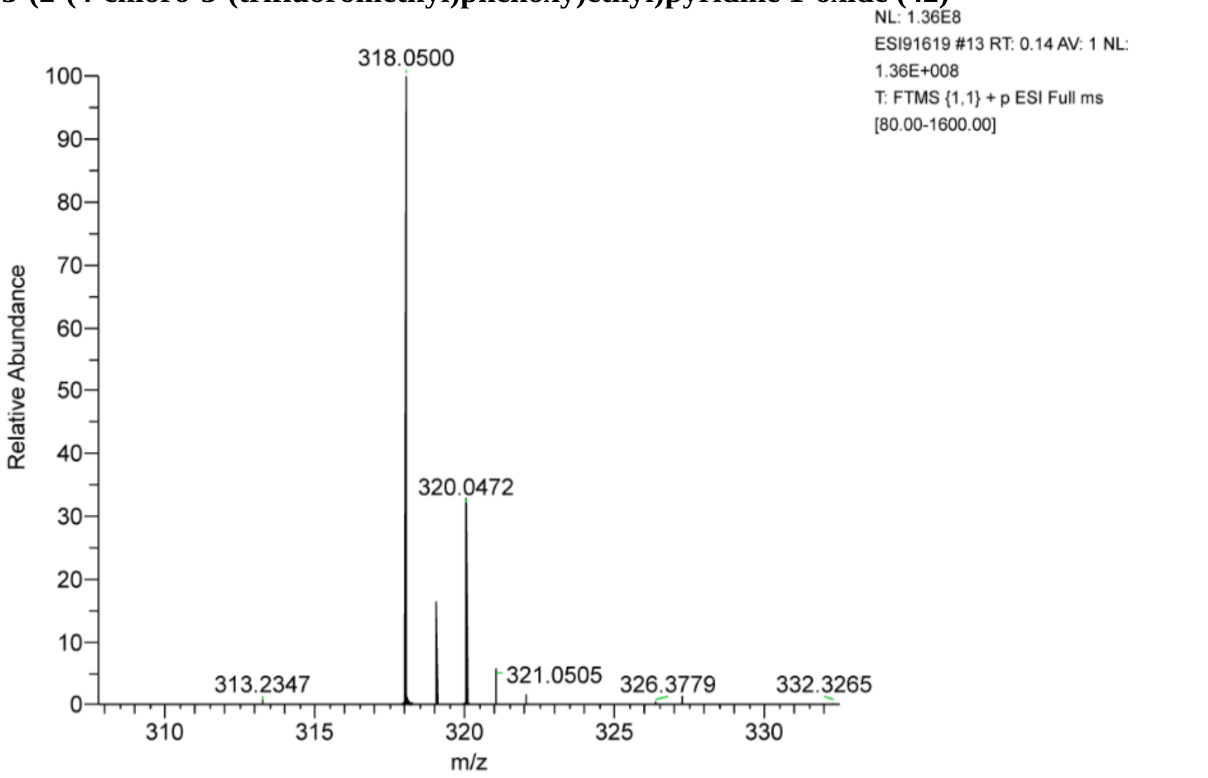

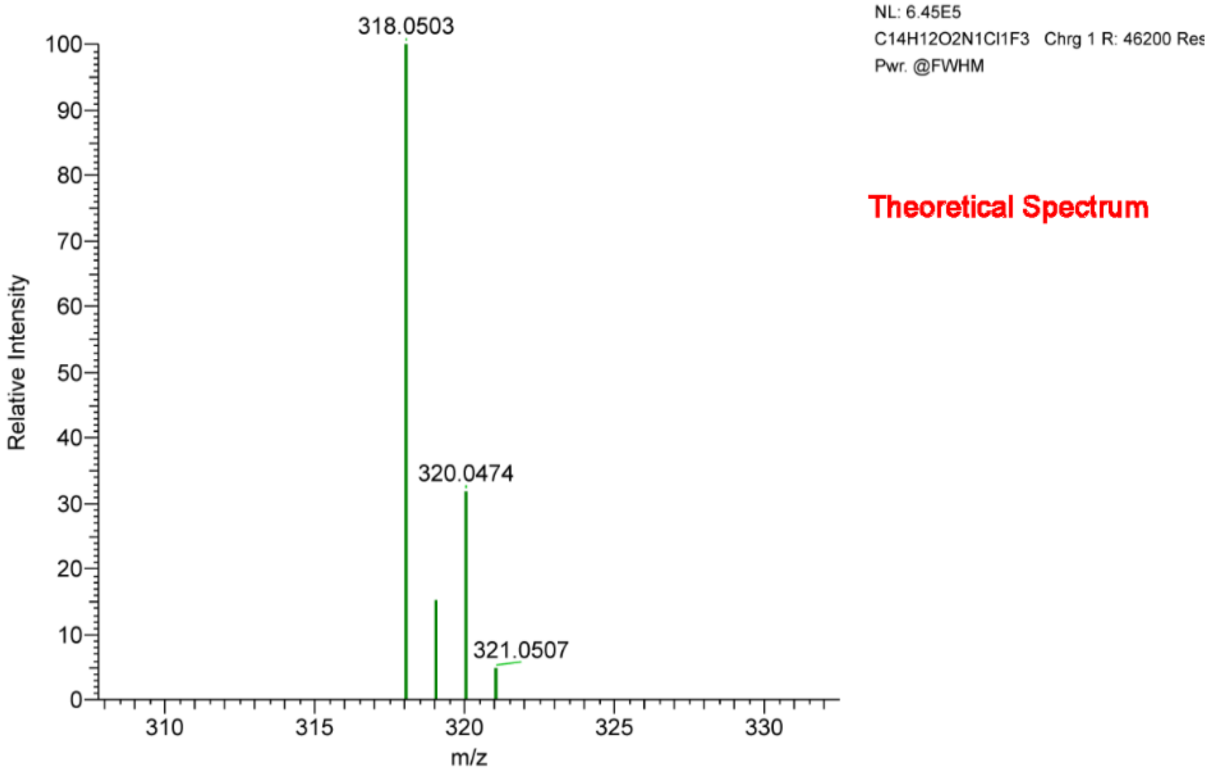

3-(2-(3,5-bis(trifluoromethyl)phenoxy)ethyl)pyridine 1-oxide (43)

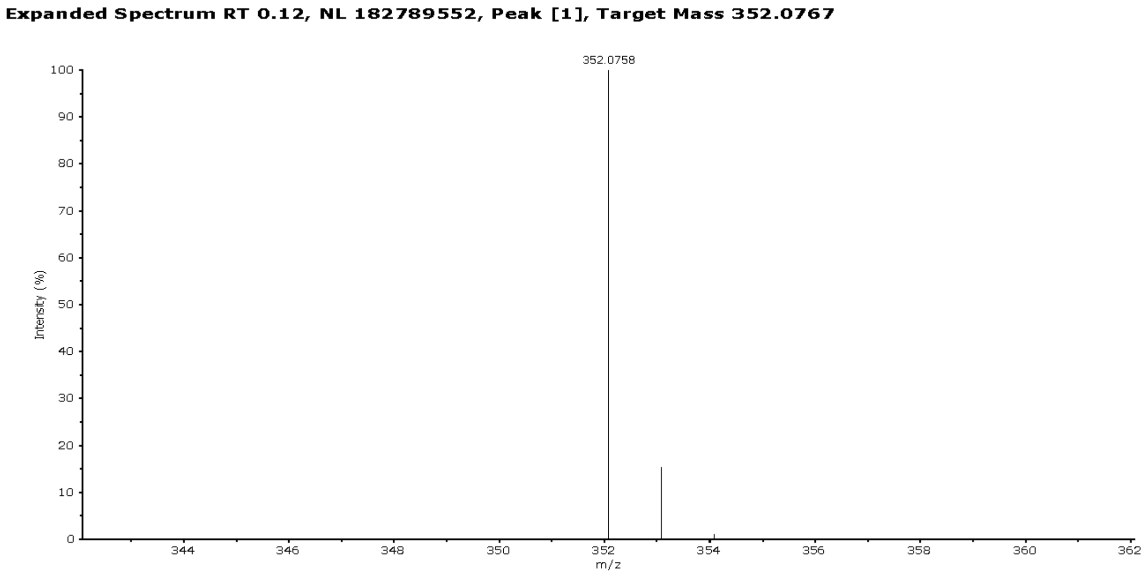

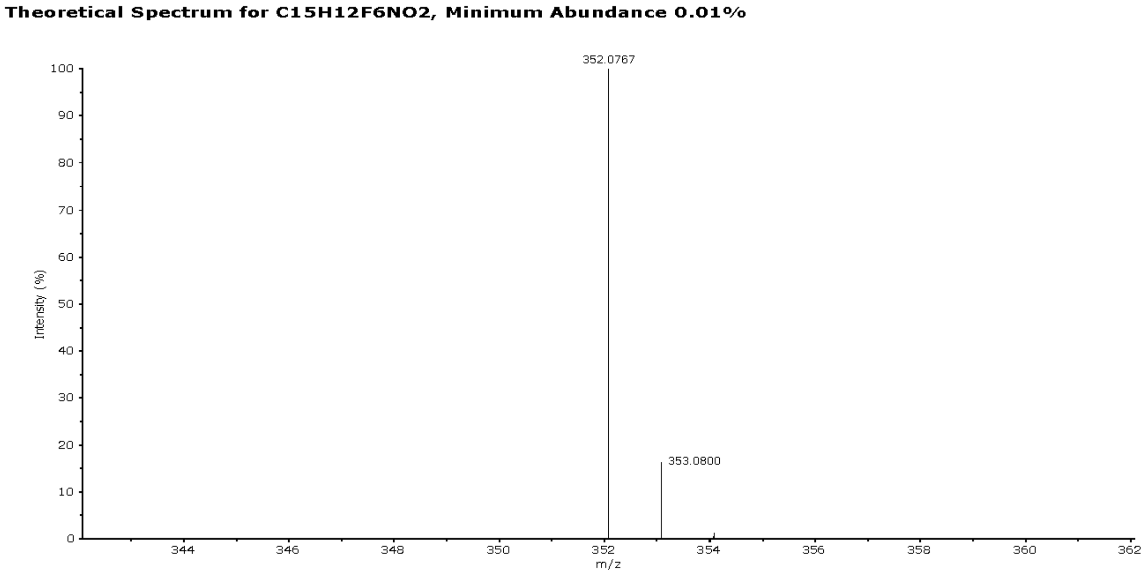

3-(((4-chloronaphthalen-1-yl)oxy)methyl)pyridine 1-oxide (44)

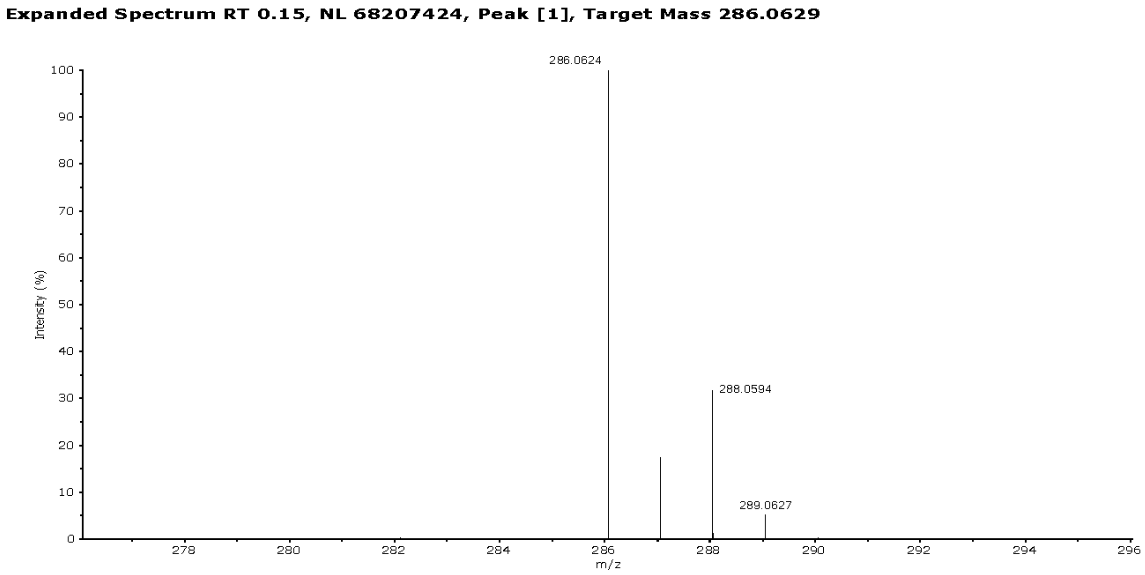

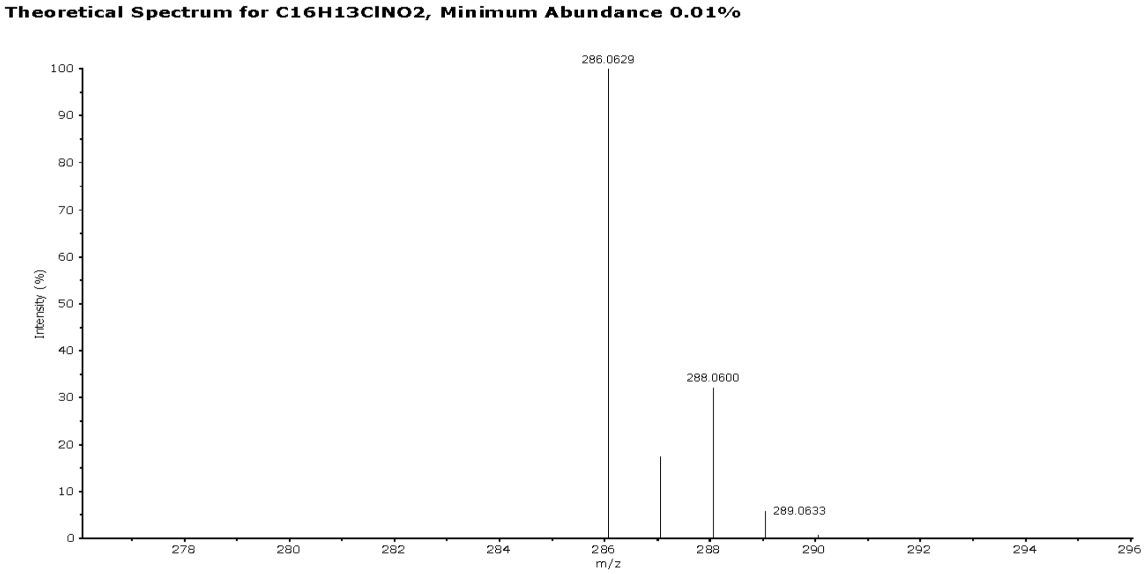

3-(3-((4-chloronaphthalen-1-yl)oxy)propyl)pyridine 1-oxide (45)

Expanded Spectrum RT 0.29, NL 52248132, Peak [1], Target Mass 314.0942

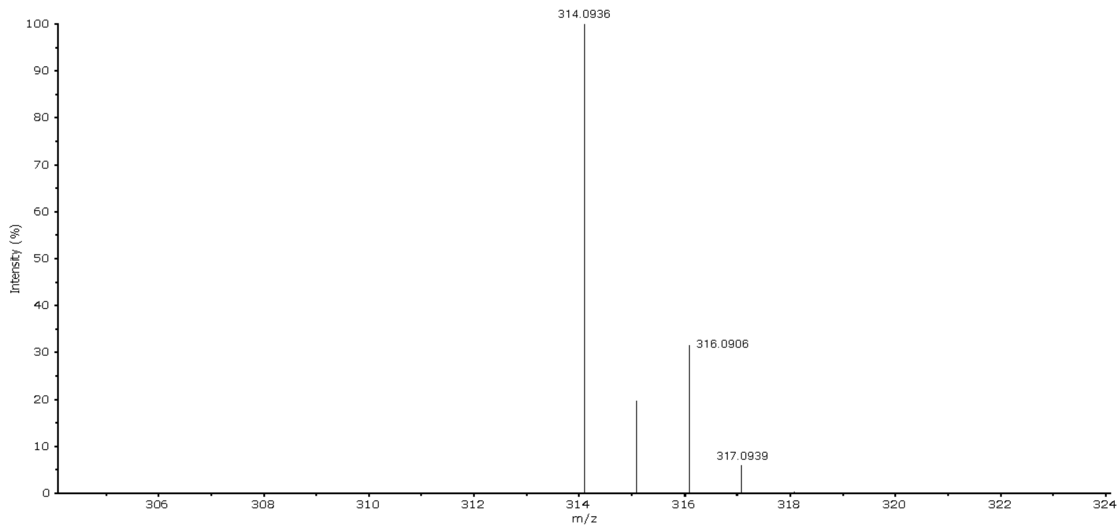

Theoretical Spectrum for C18H17ClNO2, Minimum Abundance 0.01%

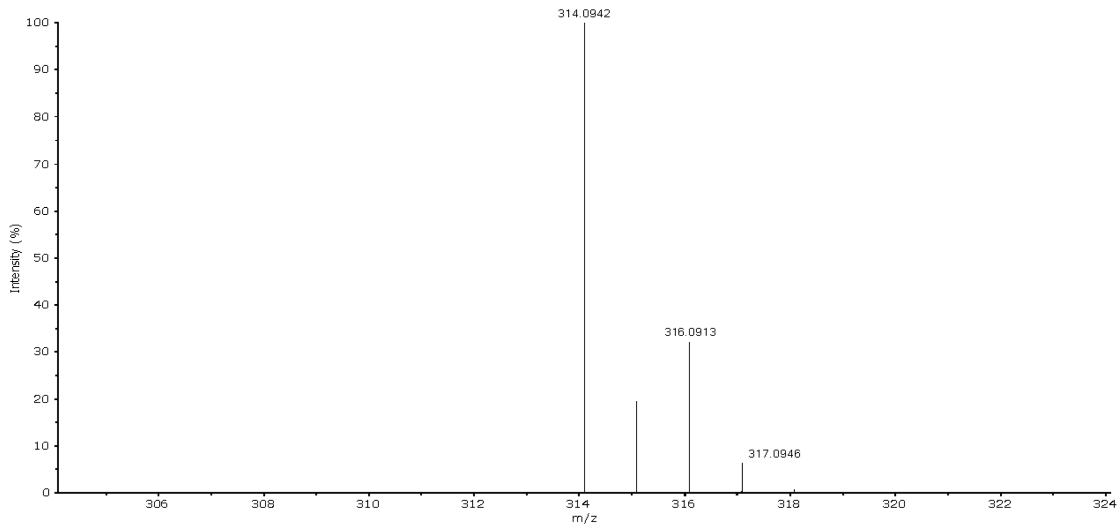

3-(3-(naphthalen-1-yl)propyl)pyridine 1-oxide (46)

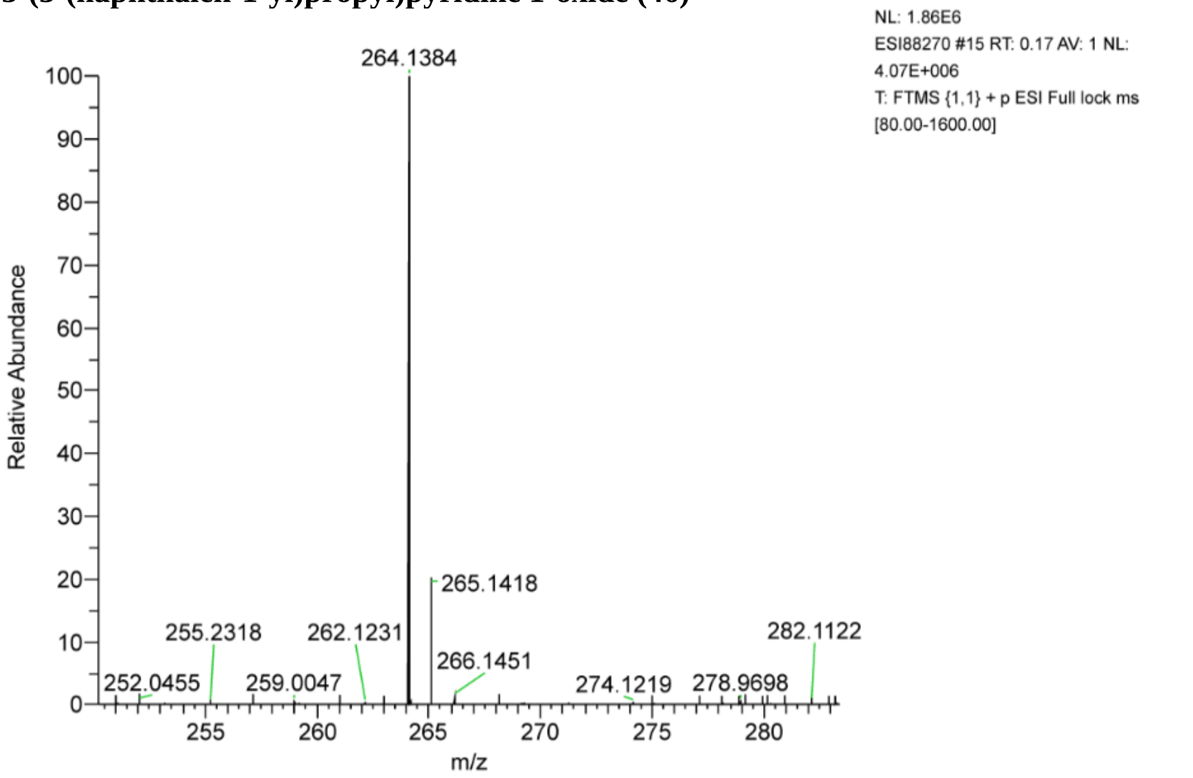

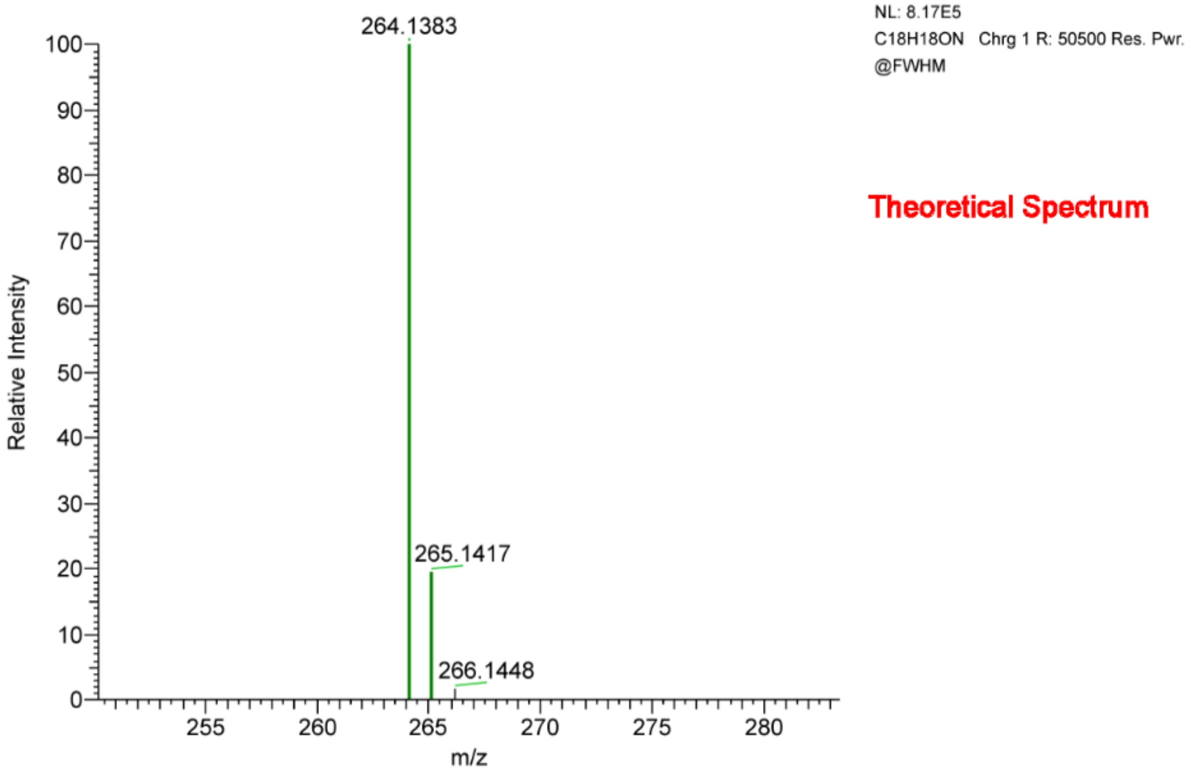

3-(3-(naphthalen-1-yl)propanoyl)pyridine 1-oxide (47)

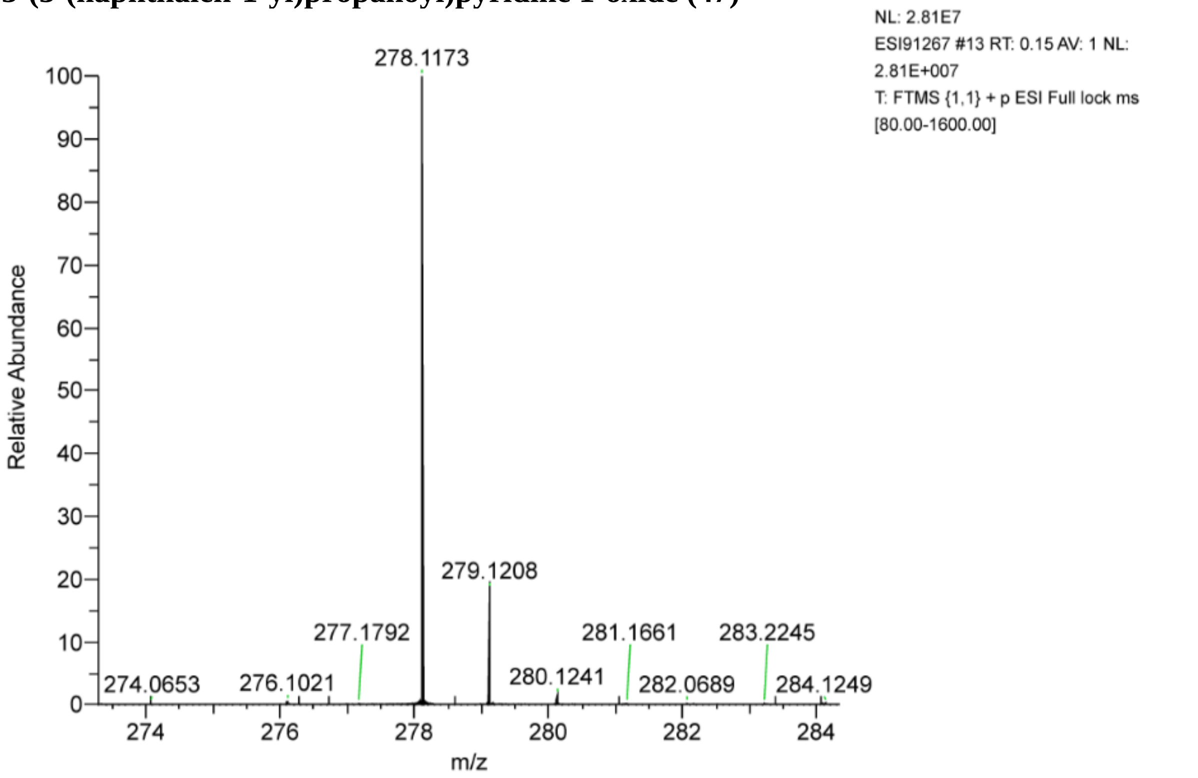

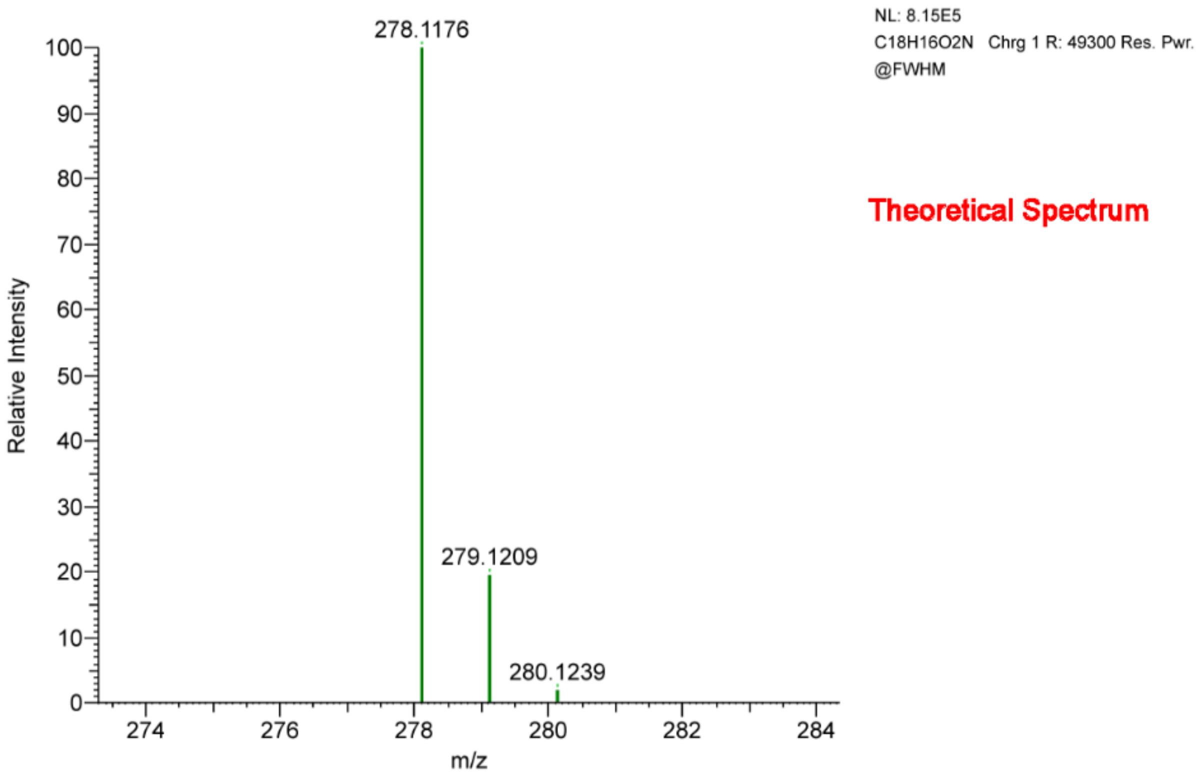

3-(4-chlorophenethoxy)pyridine 1-oxide (48)

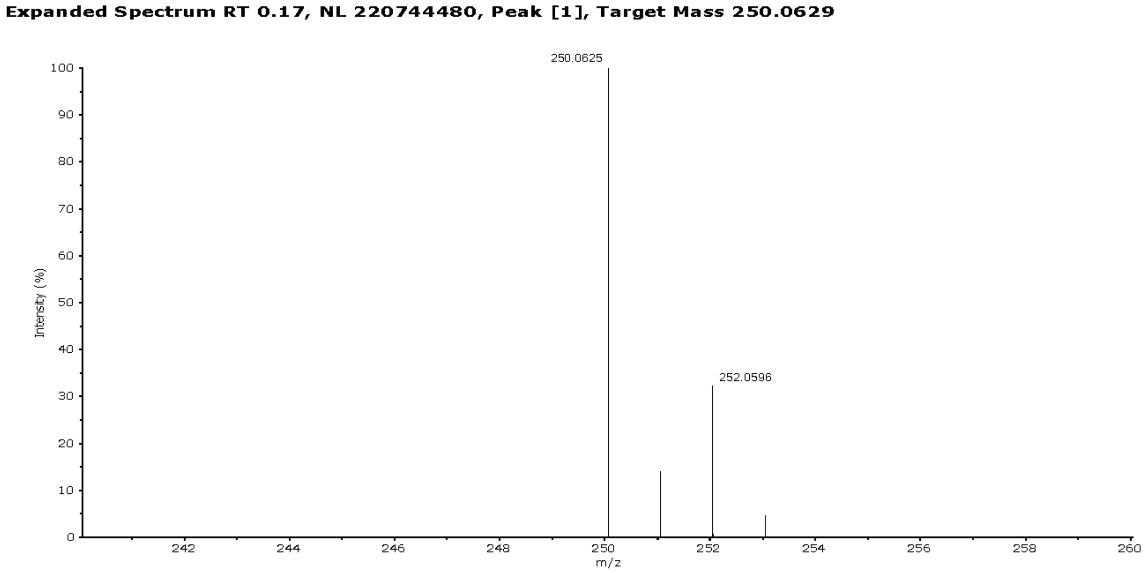

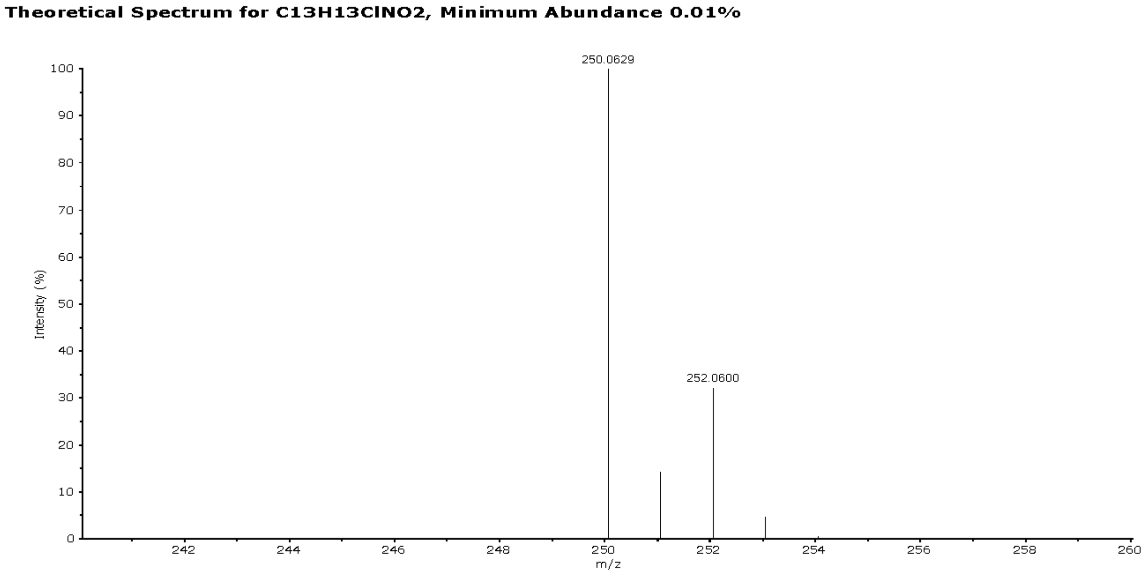

3-(3-(4-chlorophenyl)propyl)pyridine 1-oxide (49)

Expanded Spectrum RT 0.22, NL 195968096, Peak [2], Target Mass 248.0837

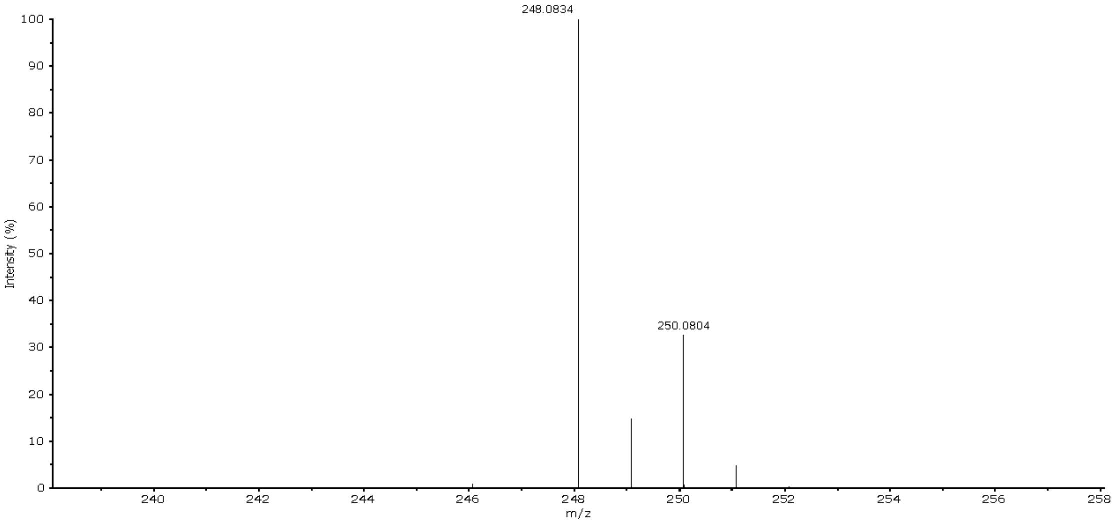

Theoretical Spectrum for C14H15ClNO, Minimum Abundance 0.01%

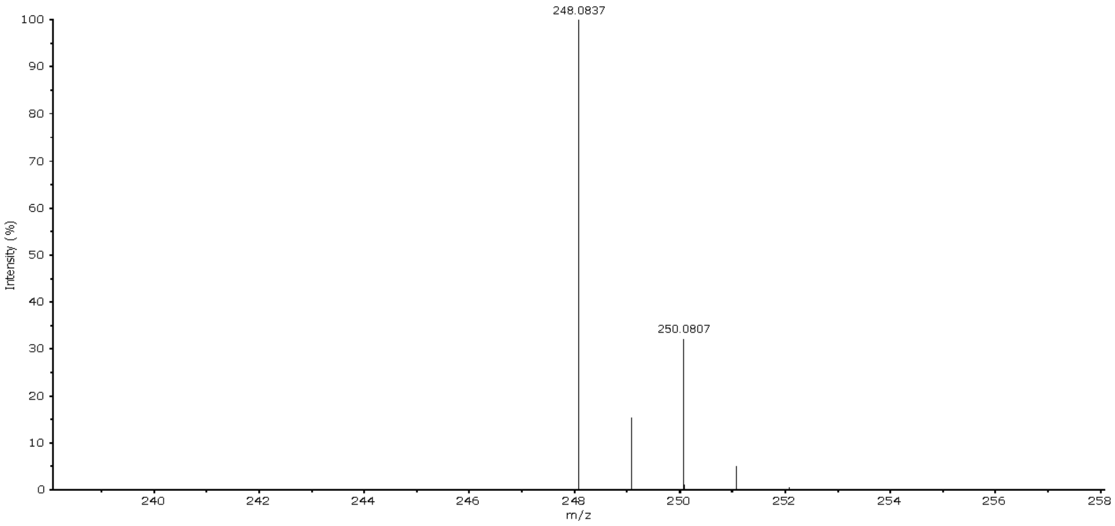

3-(3-(4-chlorophenyl)propanoyl)pyridine 1-oxide (50)

Expanded Spectrum RT 0.21, NL 48552320, Peak [1], Target Mass 262.0629

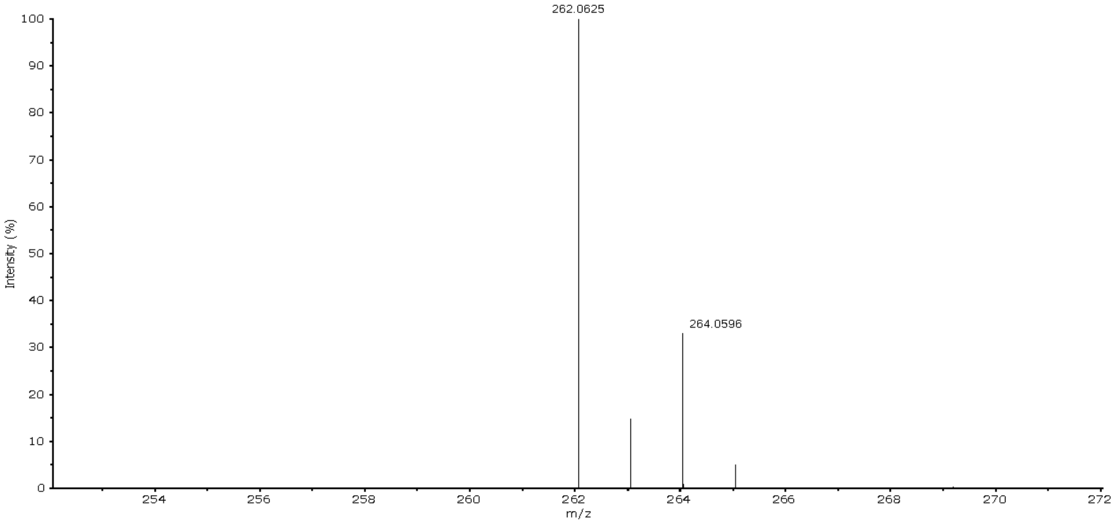

Theoretical Spectrum for C14H13ClNO2, Minimum Abundance 0.01%

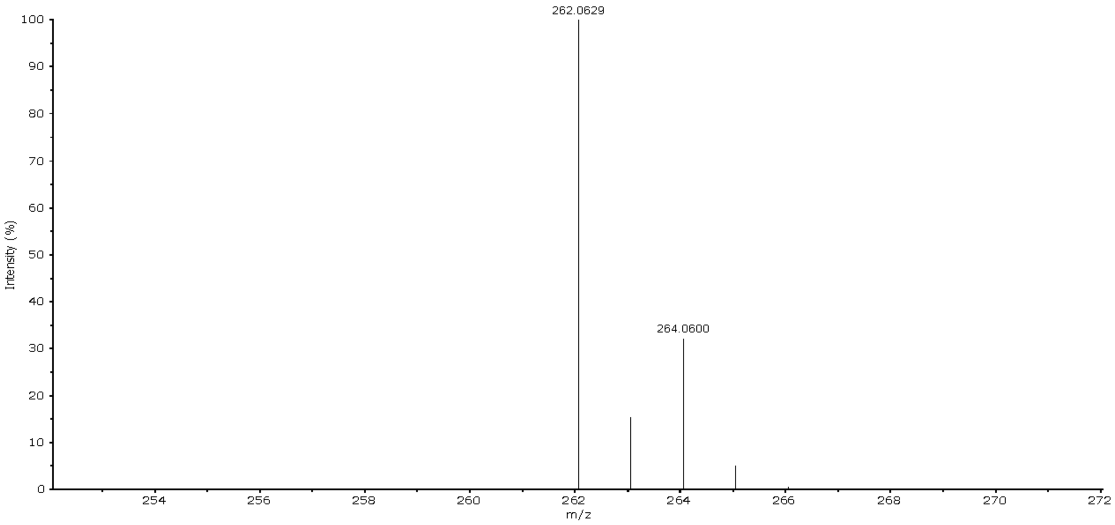

3-(2-((4-chlorophenyl)sulfonyl)ethyl)pyridine 1-oxide (51)

Expanded Spectrum RT 0.18, NL 123203920, Peak [1], Target Mass 298.0299

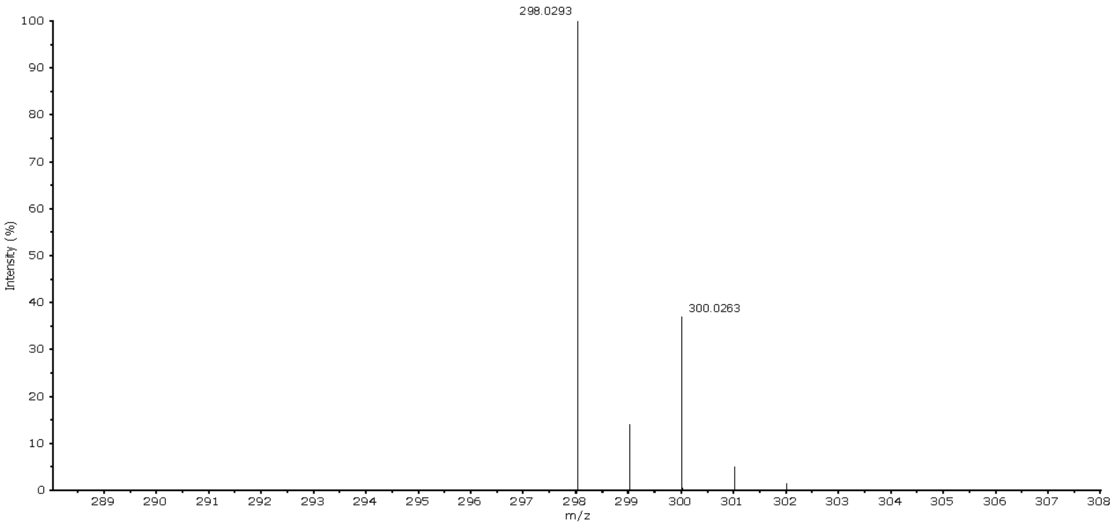

Theoretical Spectrum for C13H13ClNO3S, Minimum Abundance 0.01%

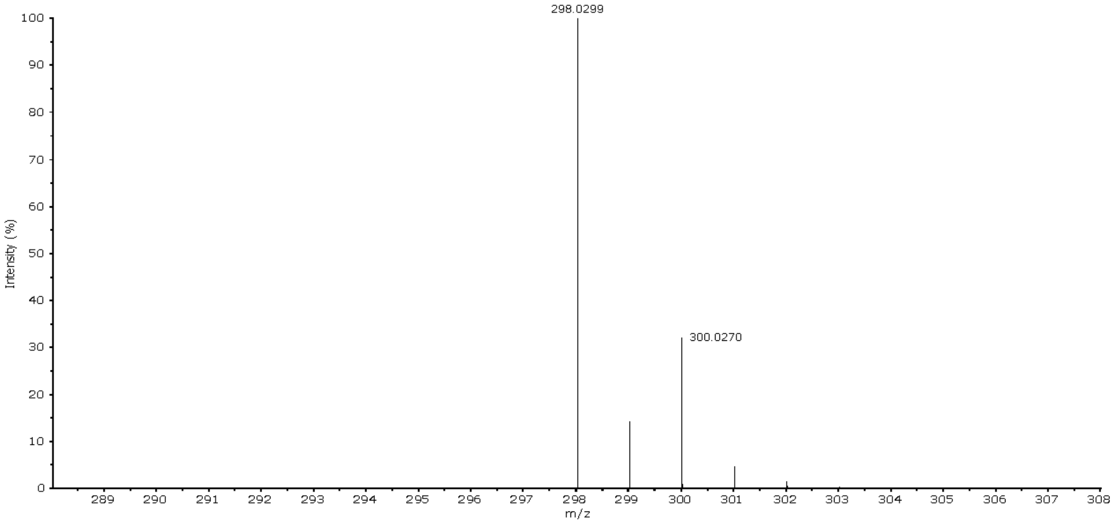

3-(3-(4-chlorophenyl)-3-fluoropropyl)pyridine 1-oxide (52)

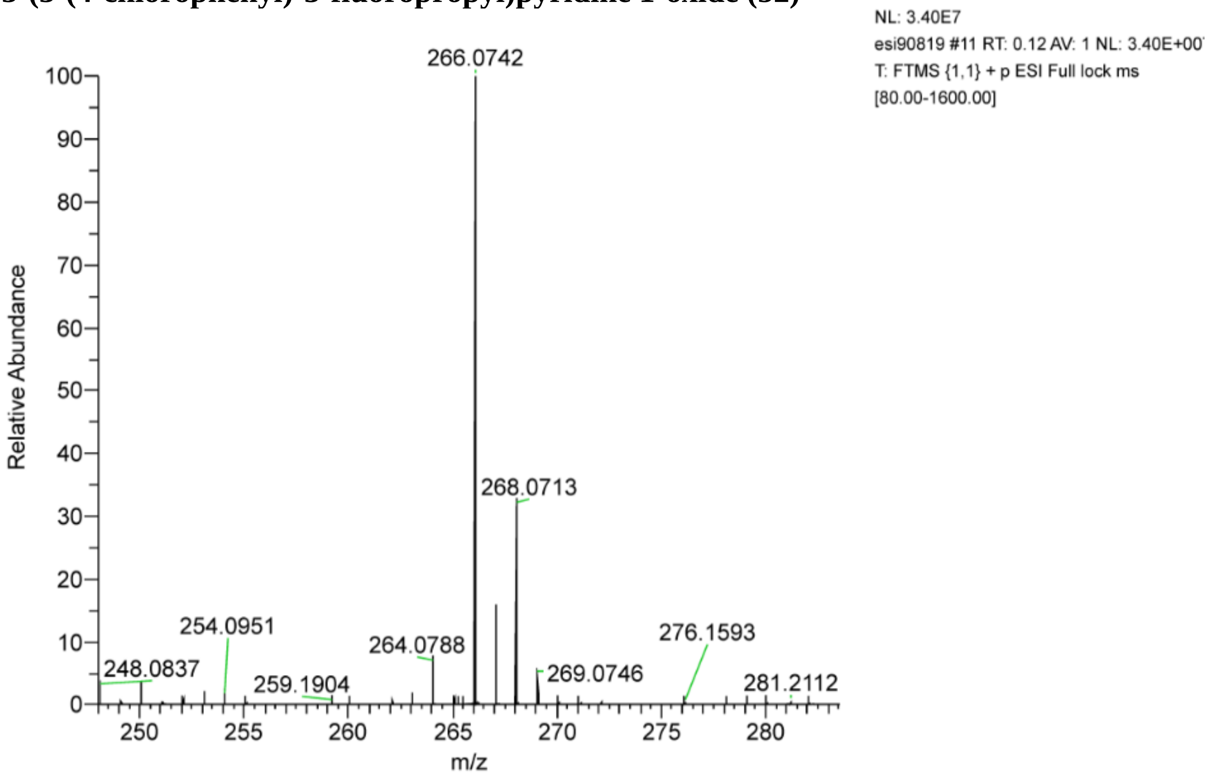

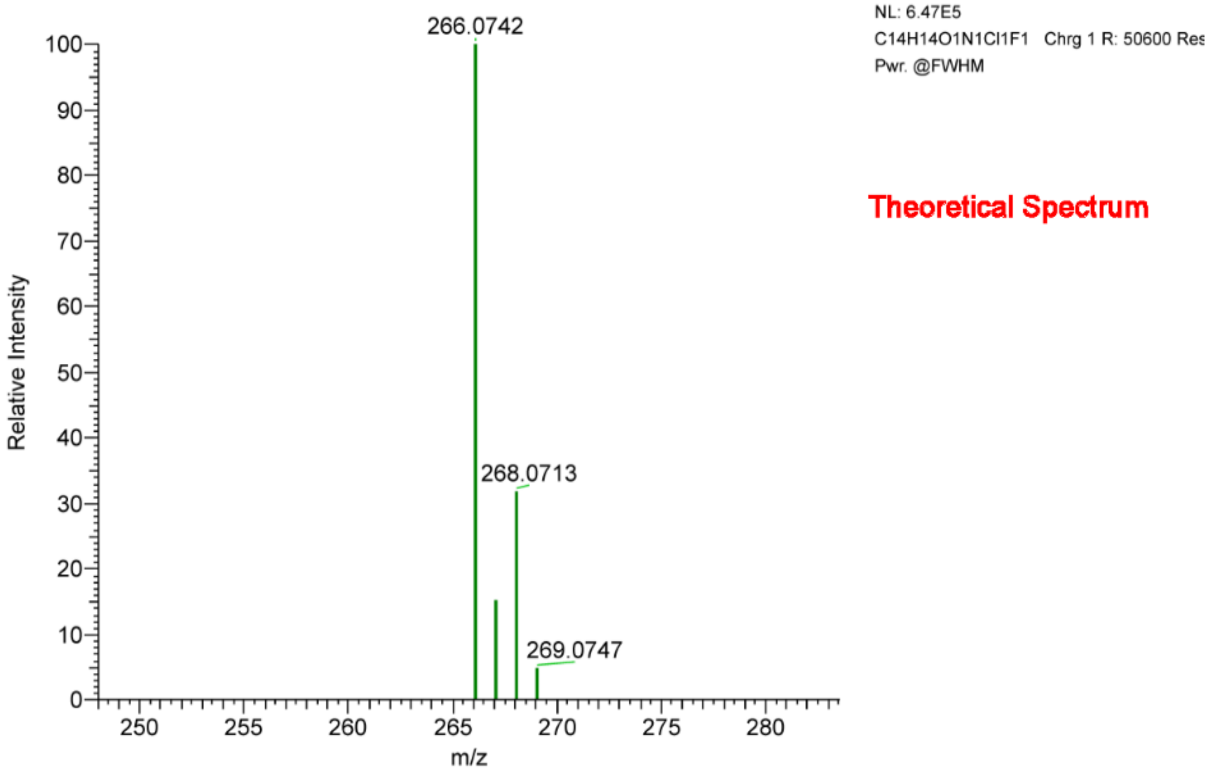

3-(3-(4-chlorophenyl)-3-hydroxypropyl)pyridine 1-oxide (53)

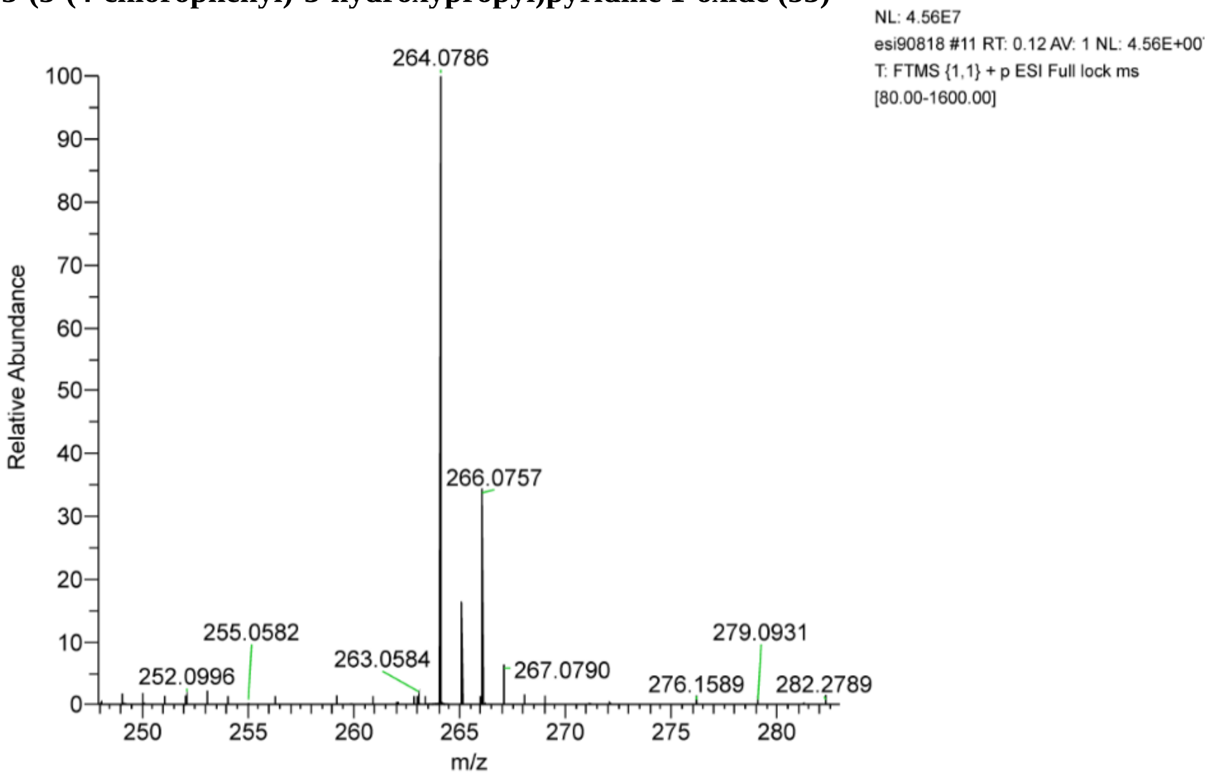

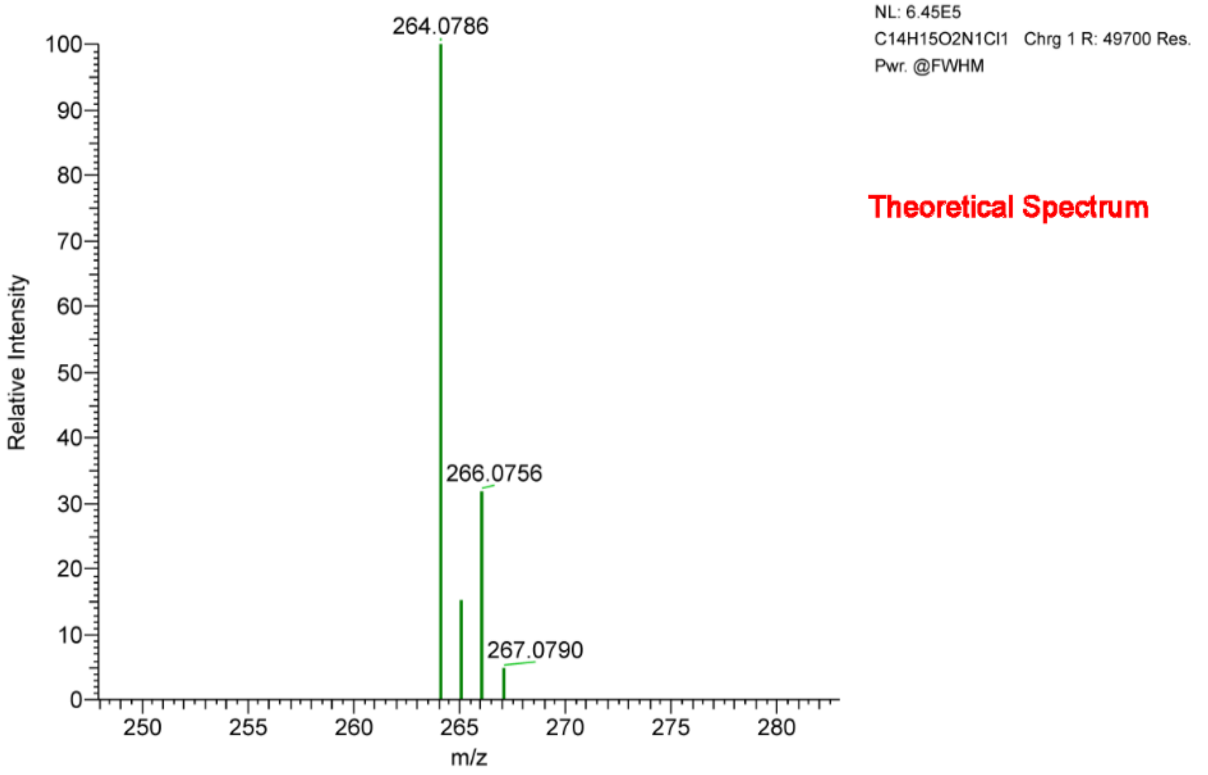

3-(3-(4-chlorophenyl)-3-oxopropyl)pyridine 1-oxide (54)

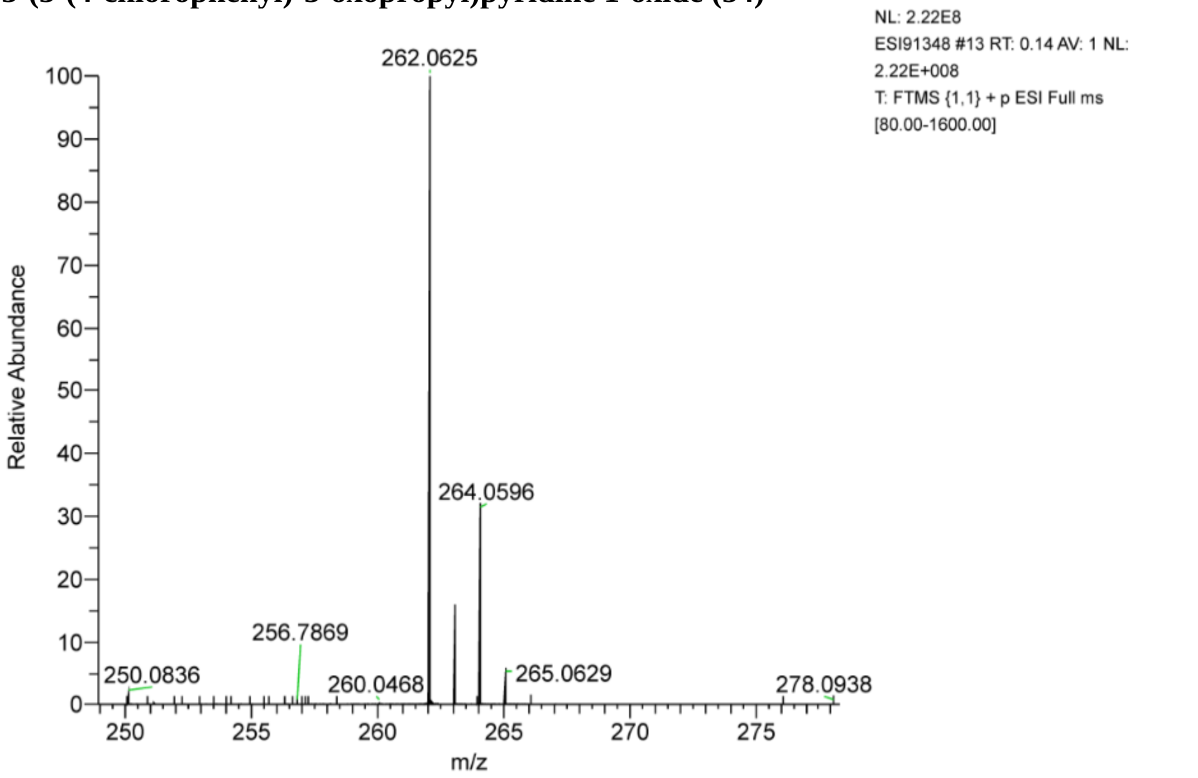

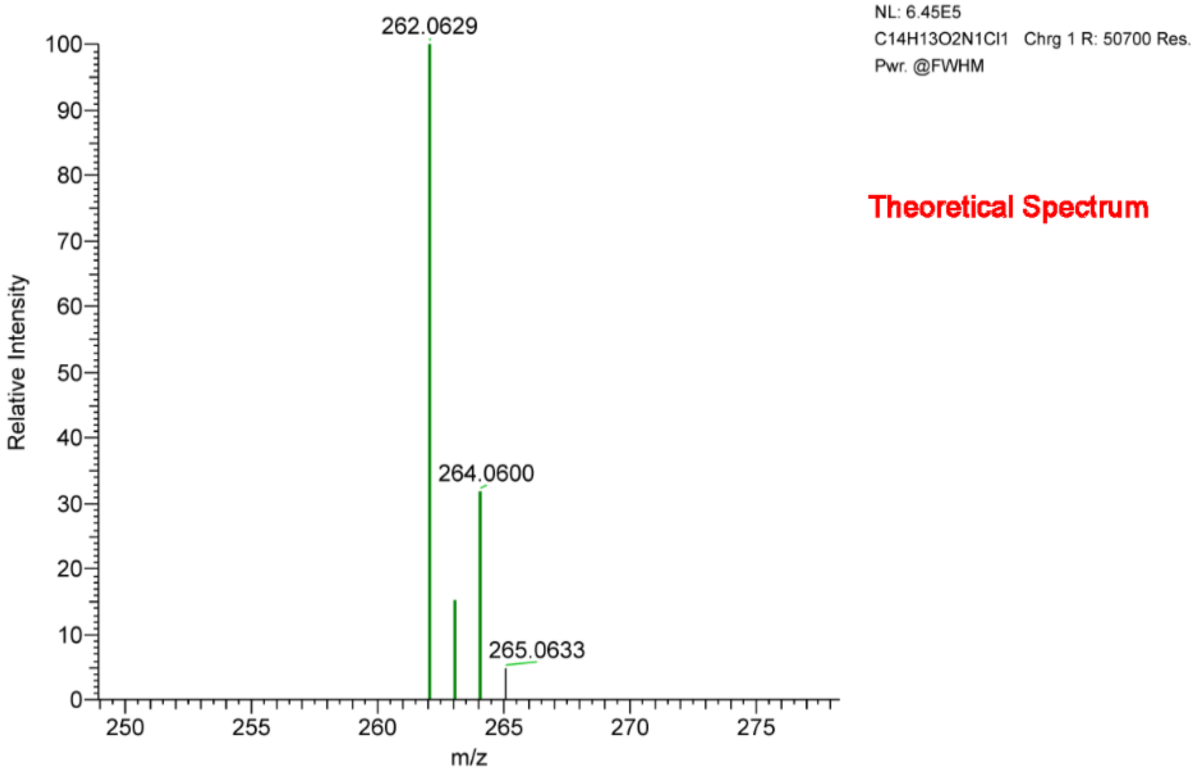

3-((4-chlorophenethyl)amino)pyridine 1-oxide (55)

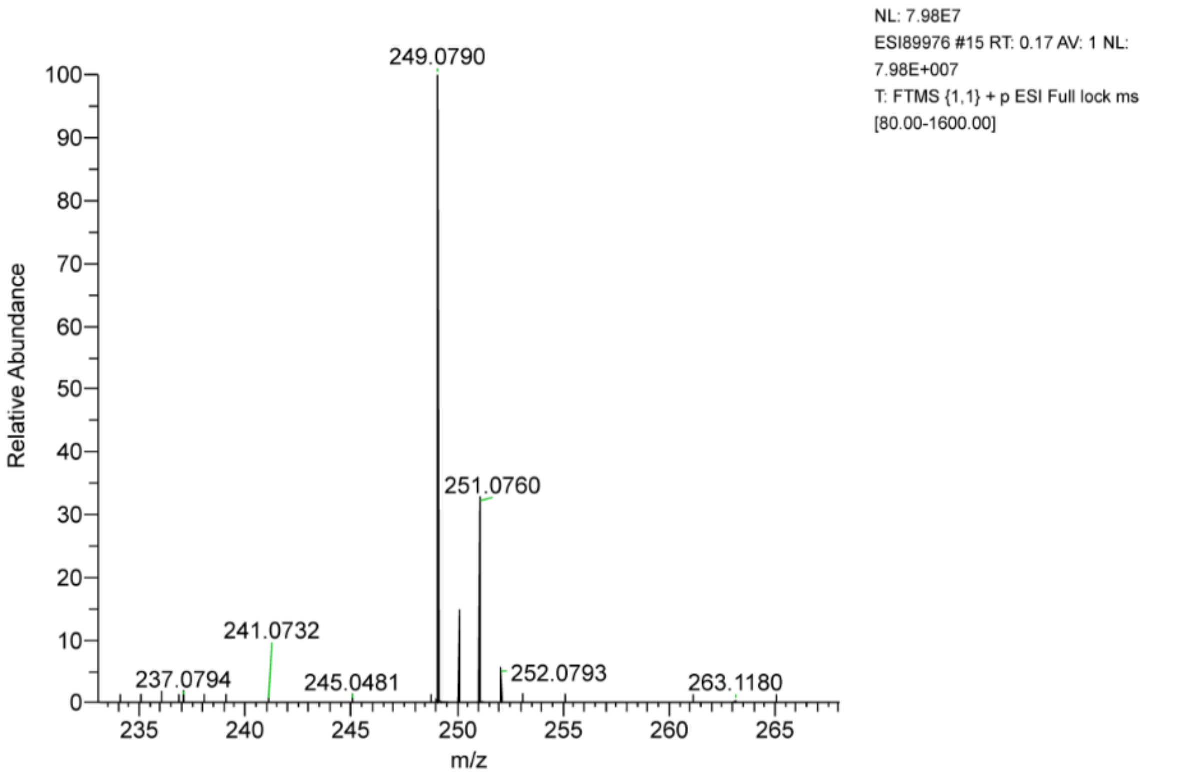

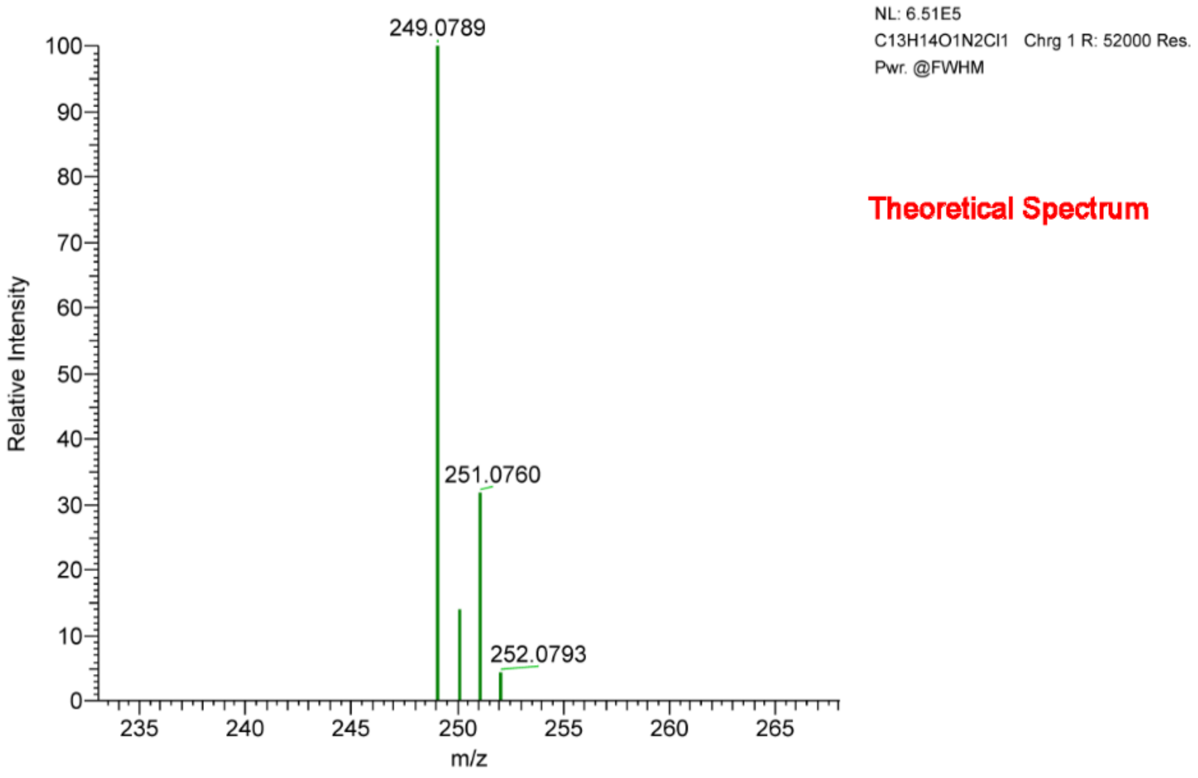

2-(2-((4-chloronaphthalen-1-yl)oxy)ethyl)-6-methoxypyridine (56)

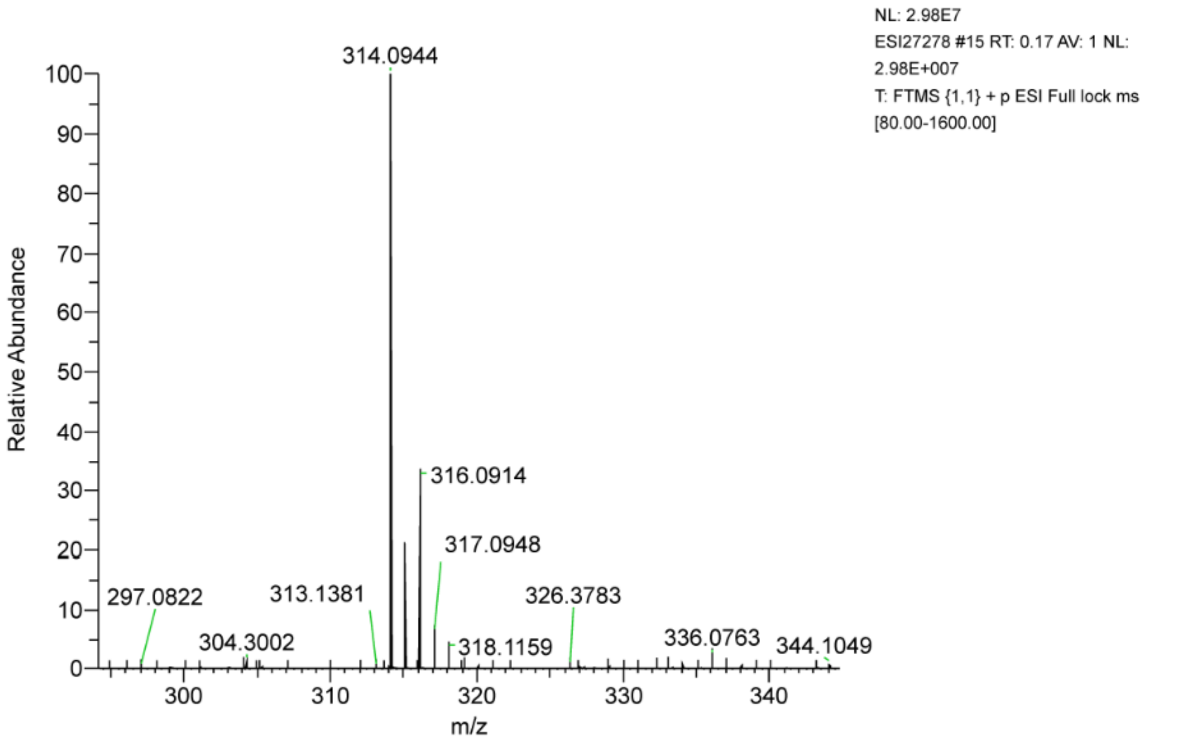

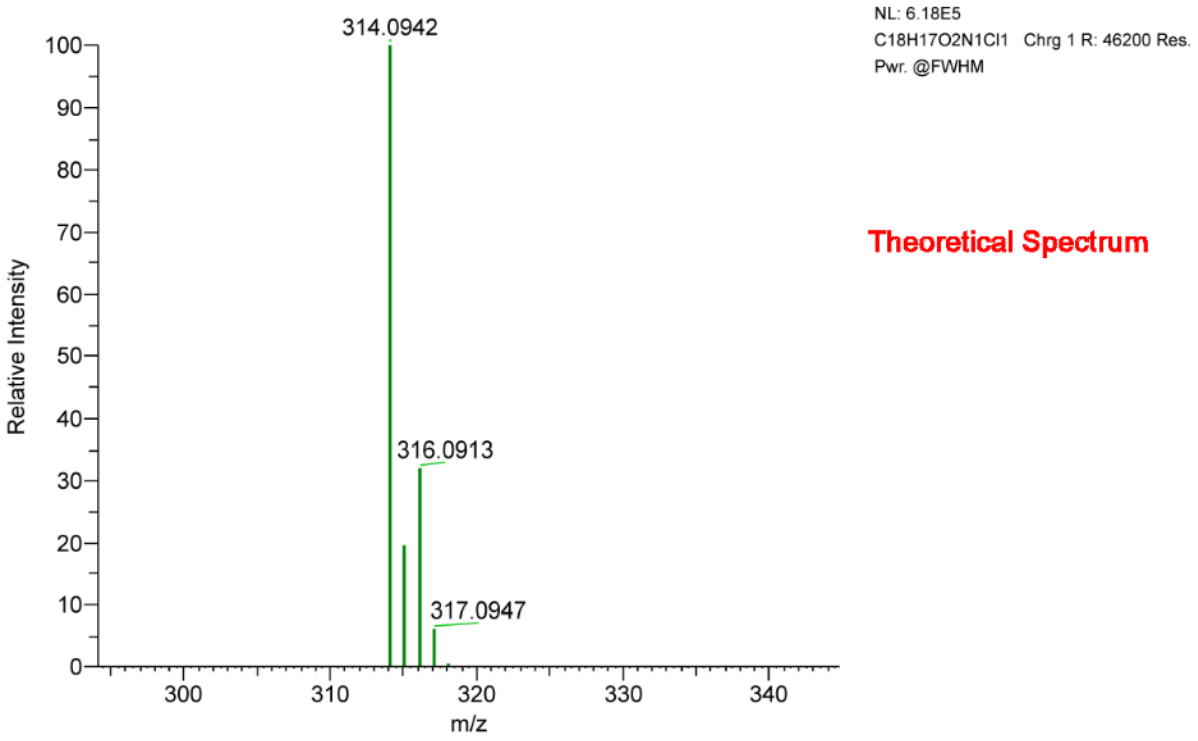

4-(2-((4-chloronaphthalen-1-yl)oxy)ethyl)-2-methoxypyridine (57)

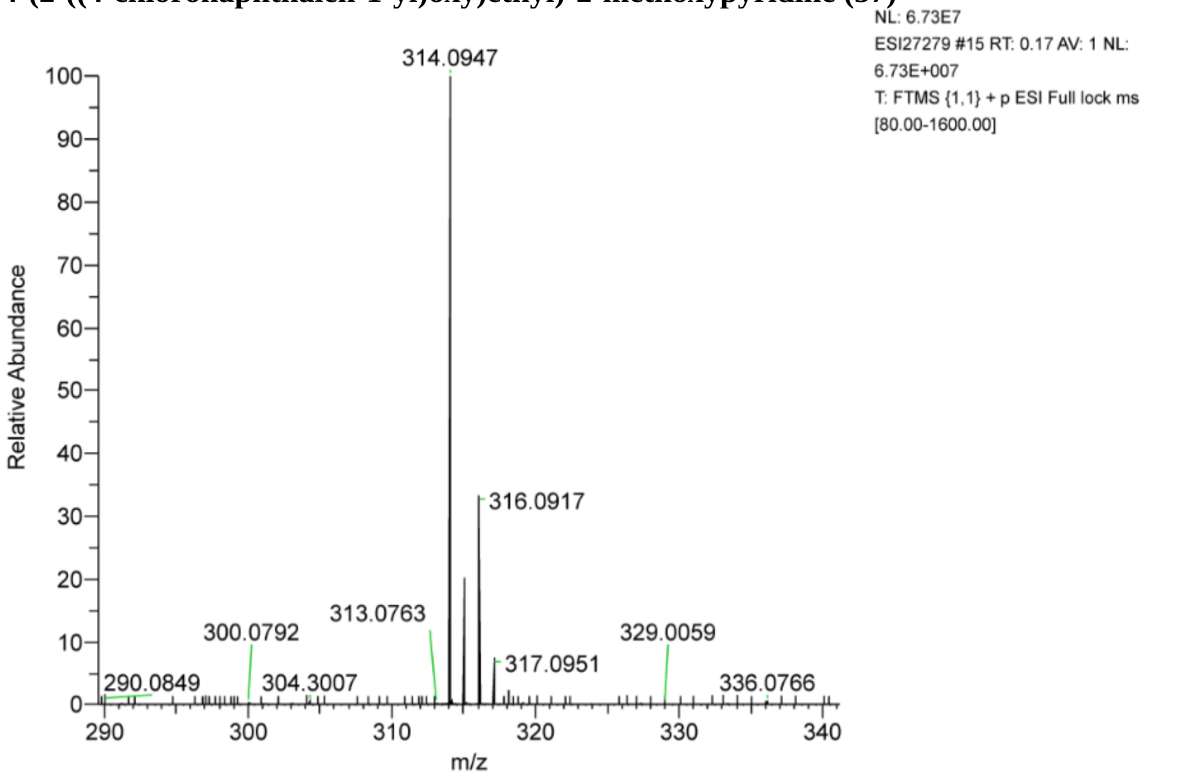

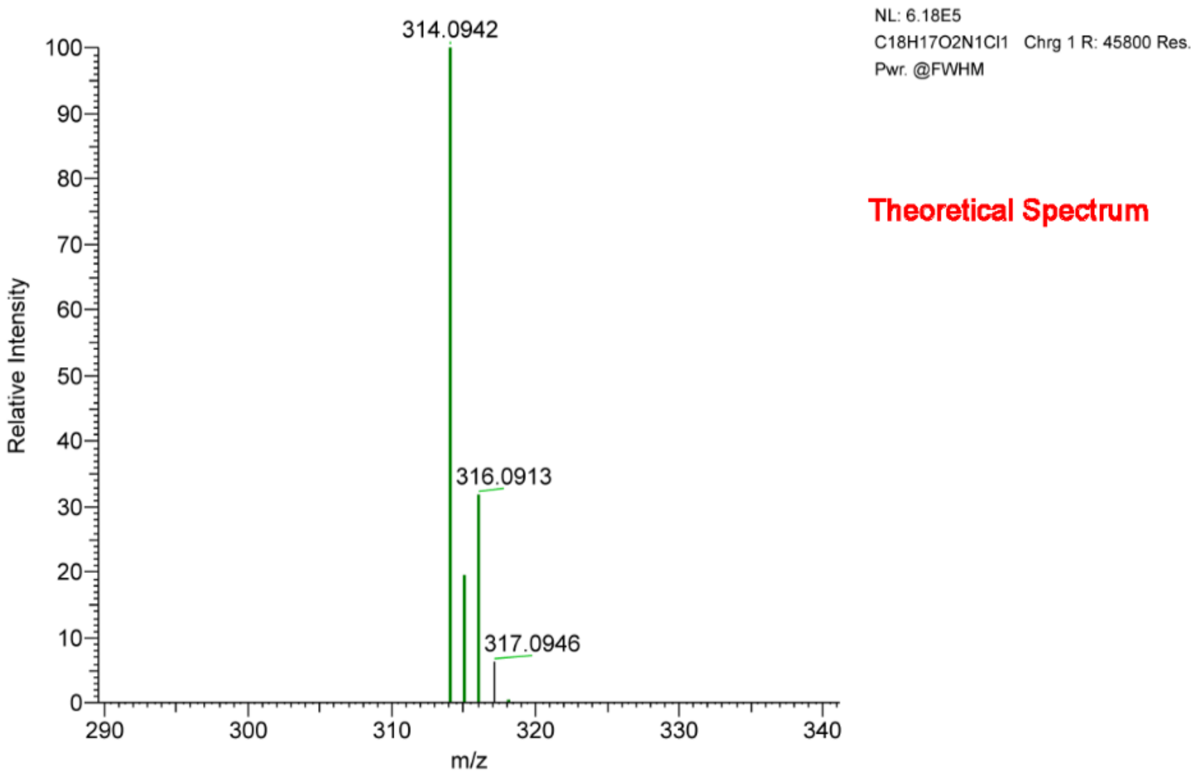

6-(2-((4-chloronaphthalen-1-yl)oxy)ethyl)pyridin-2-ol (58)

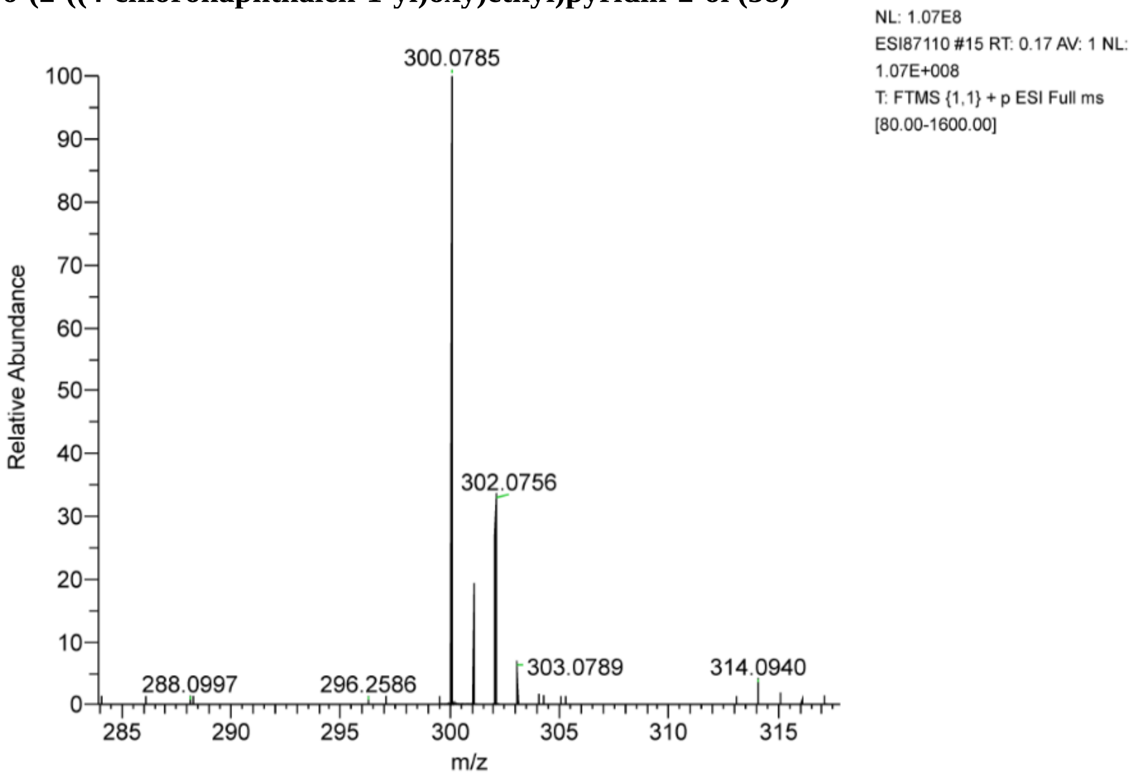

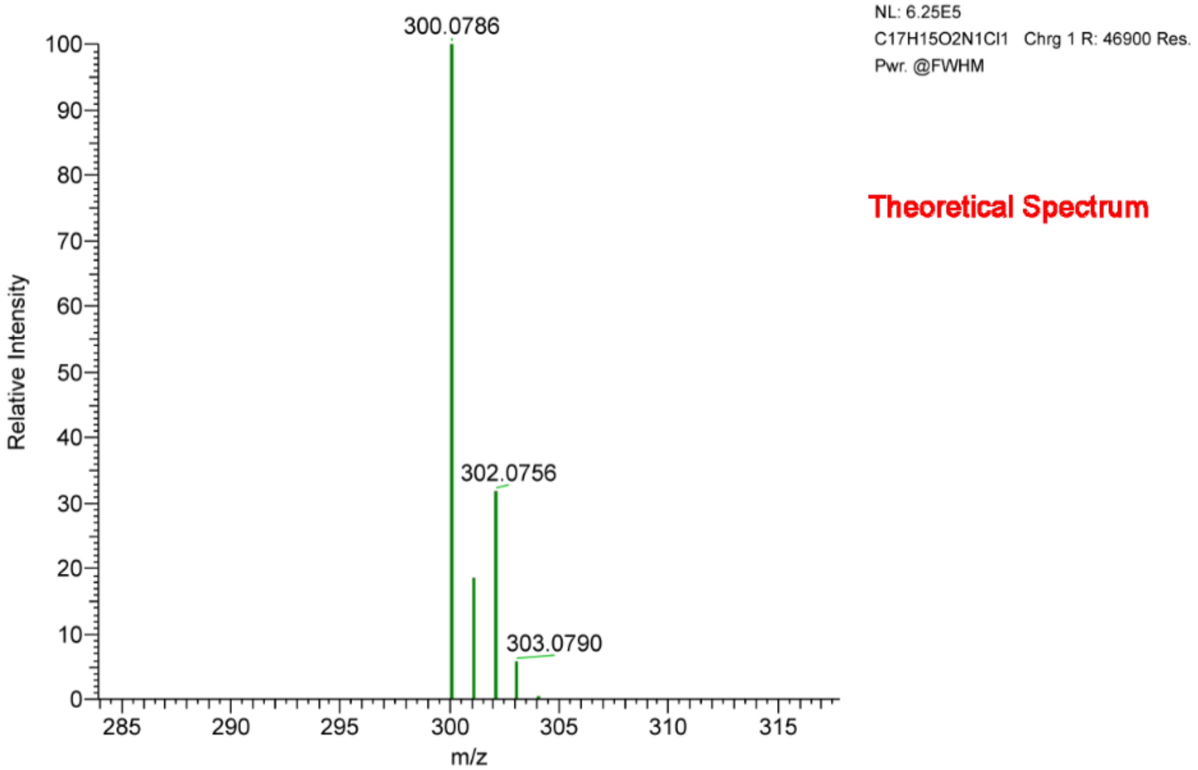

4-(2-((4-chloronaphthalen-1-yl)oxy)ethyl)pyridin-2-ol (59)

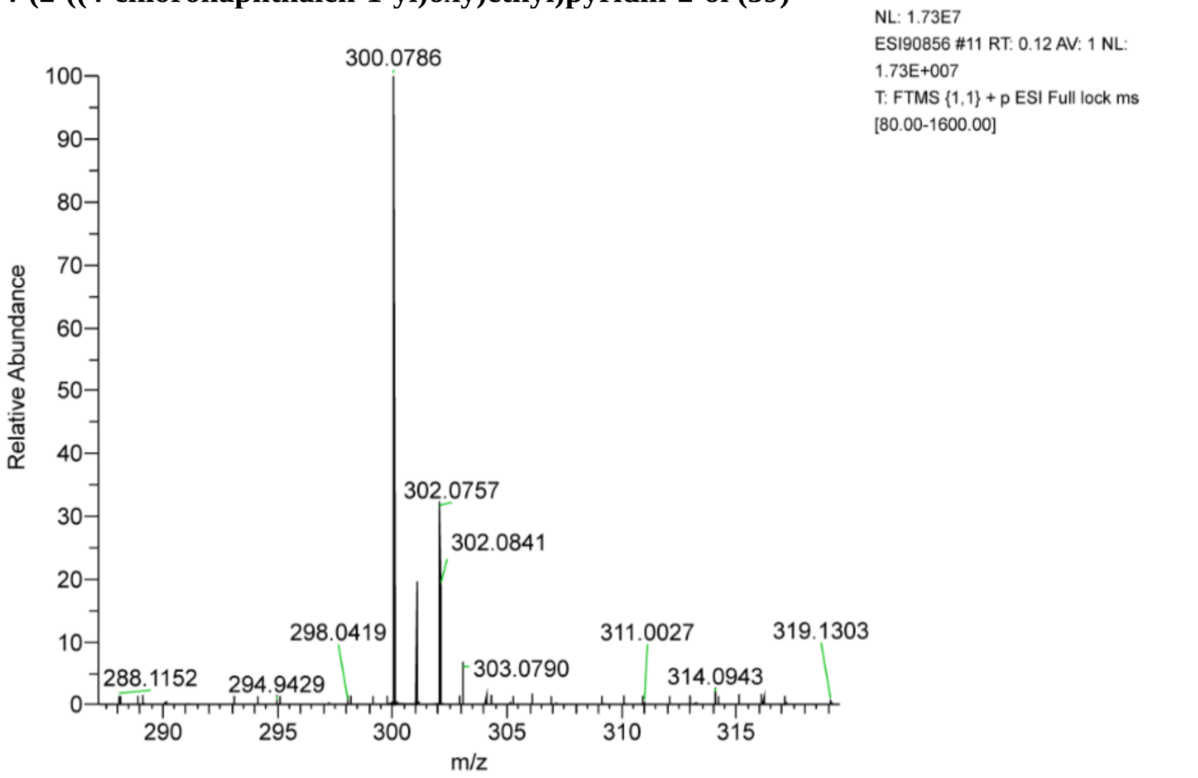

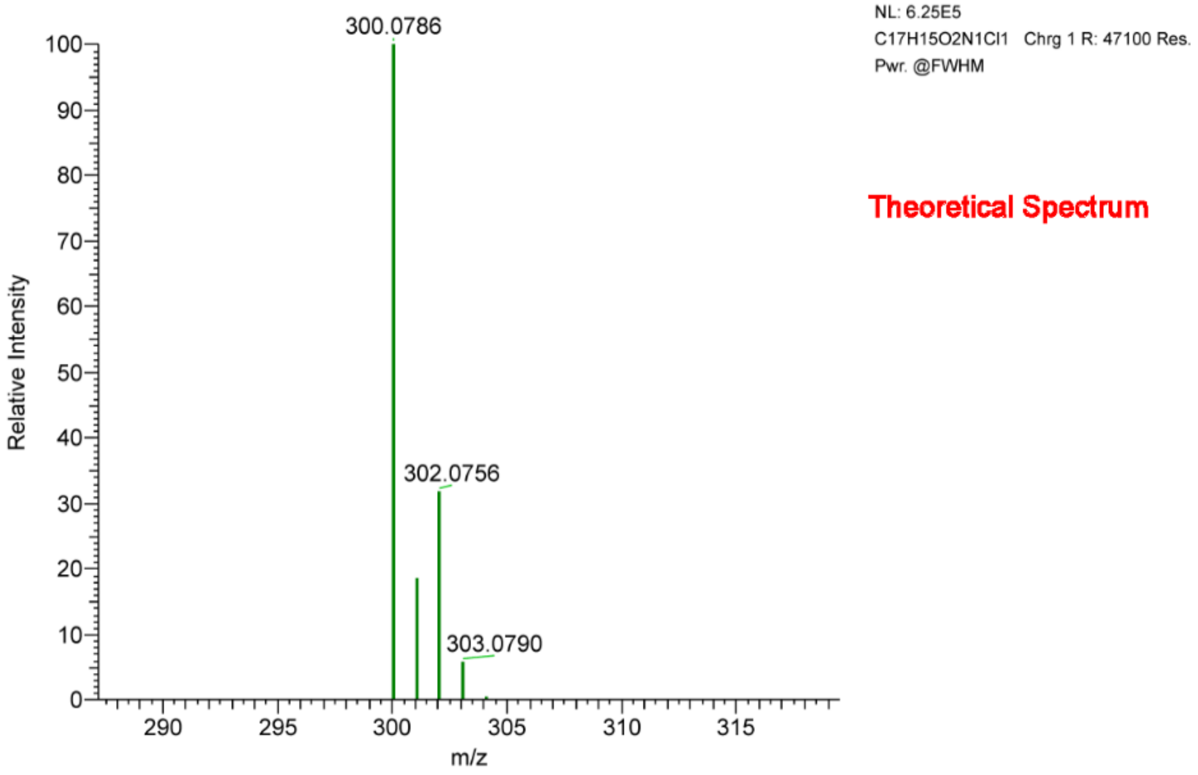

6-(2-(4-chlorophenoxy)ethyl)pyridin-2-ol (60)

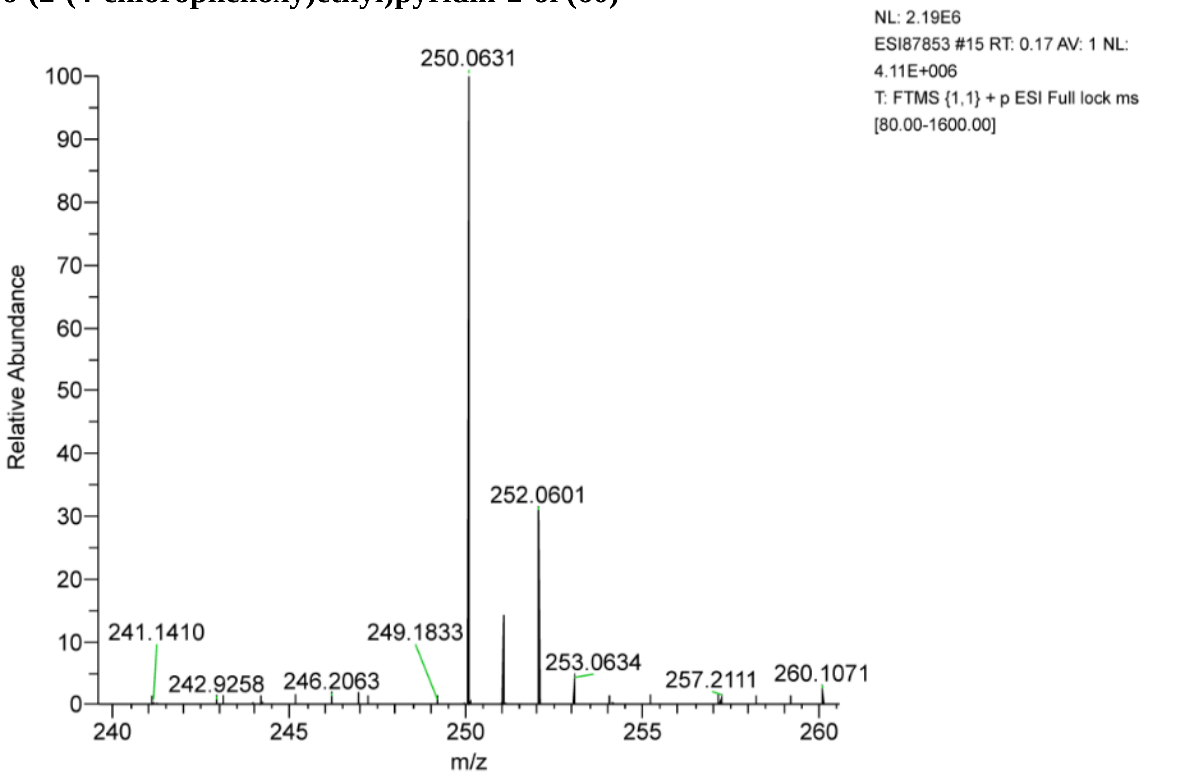

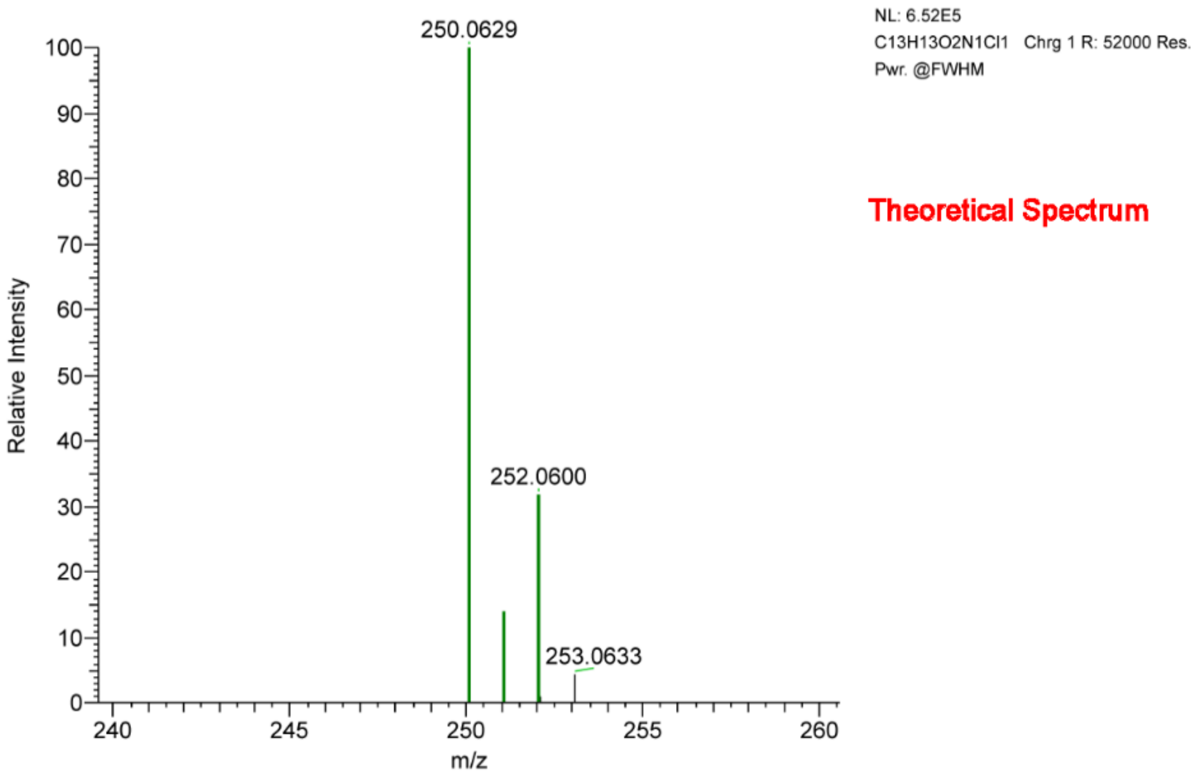

5-(2-((4-chloronaphthalen-1-yl)oxy)ethyl)-2-ethylpyridine 1-oxide (62)

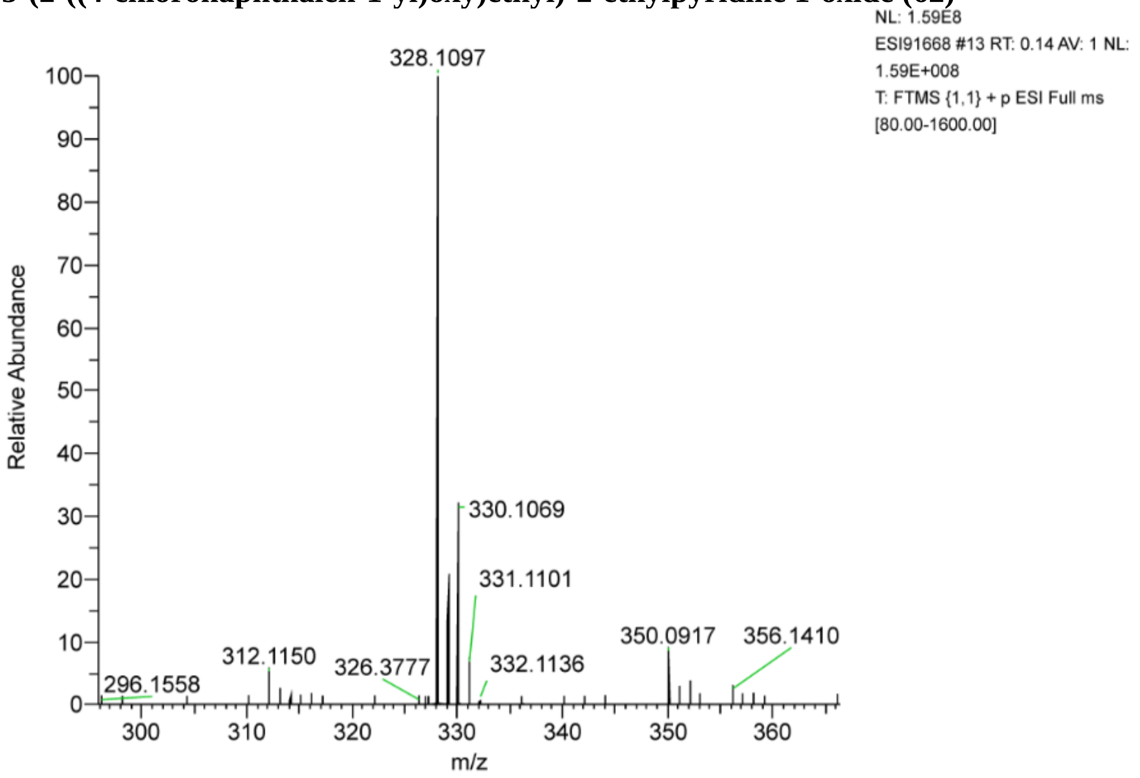

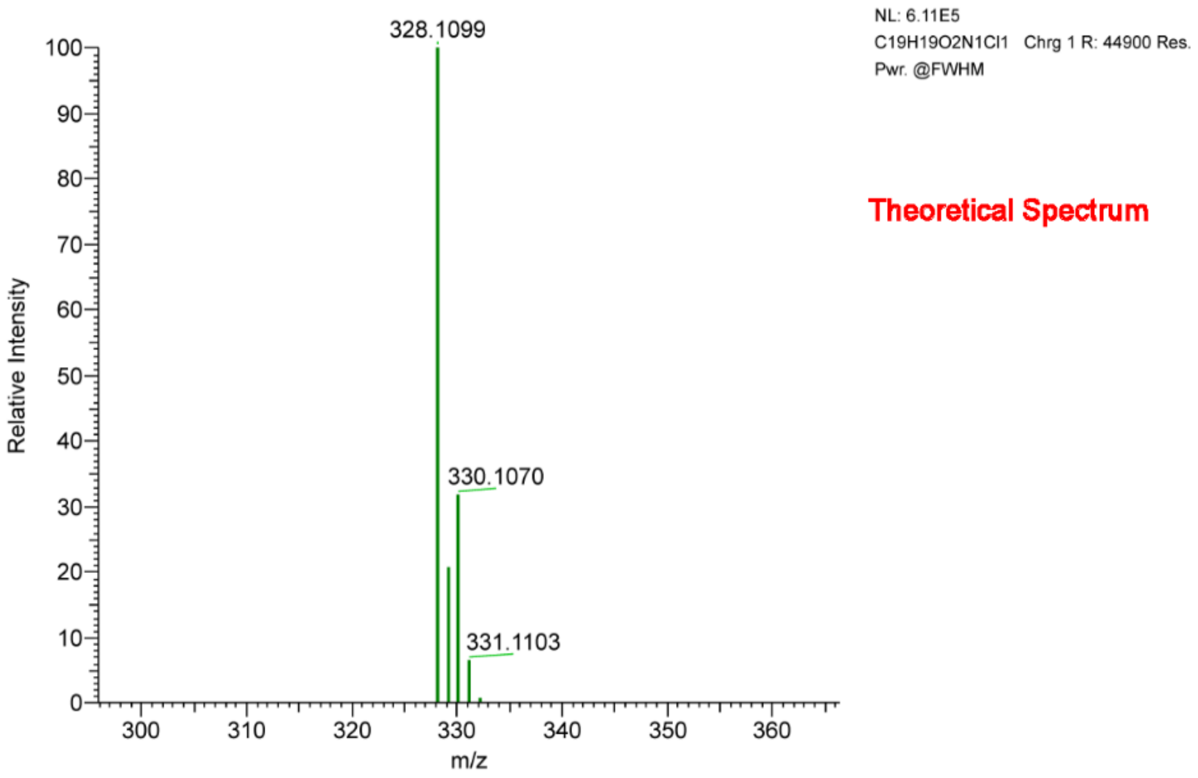

3-(2-((4-chloronaphthalen-1-yl)oxy)ethyl)-2,6-dimethylpyridine 1-oxide (63)

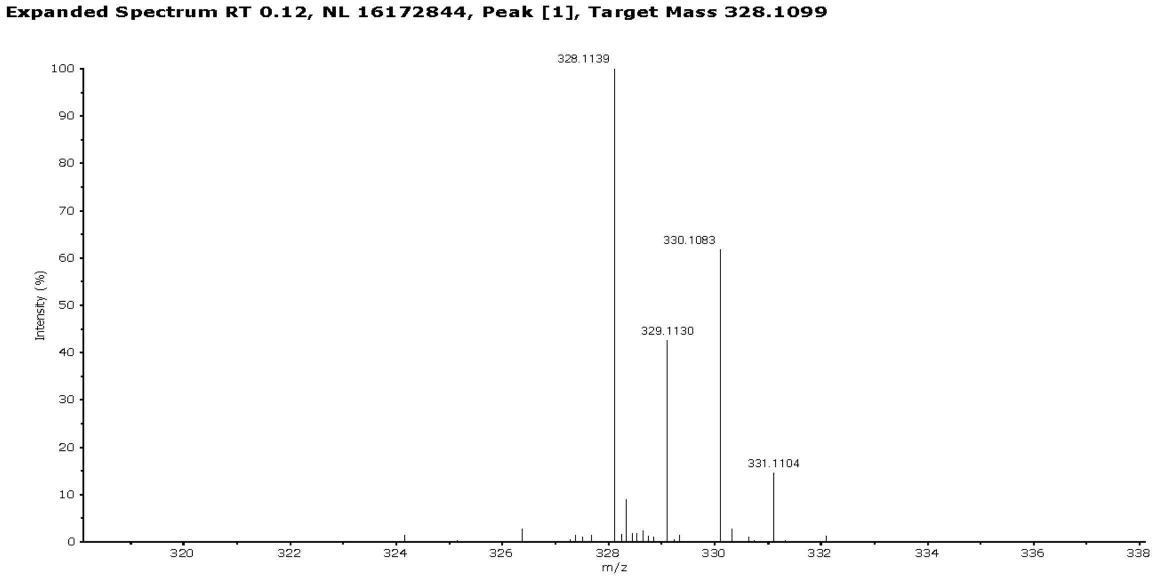

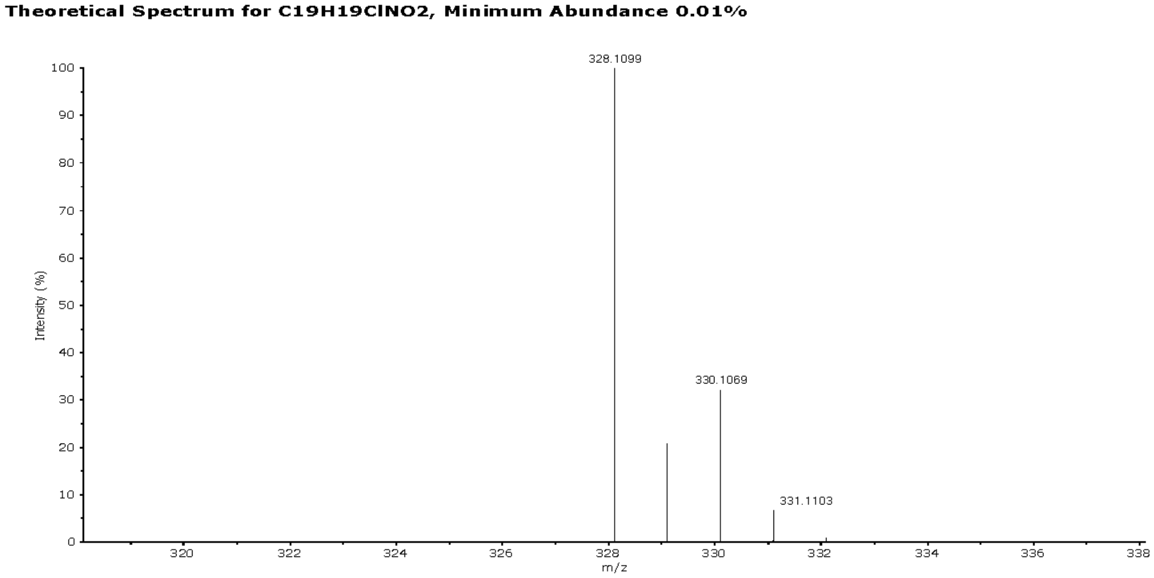

3-(3-(4-chloronaphthalen-1-yl)propyl)-5-methoxypyridine 1-oxide (64)

Expanded Spectrum RT 0.14, NL 148243328, Peak [1], Target Mass 298.0993

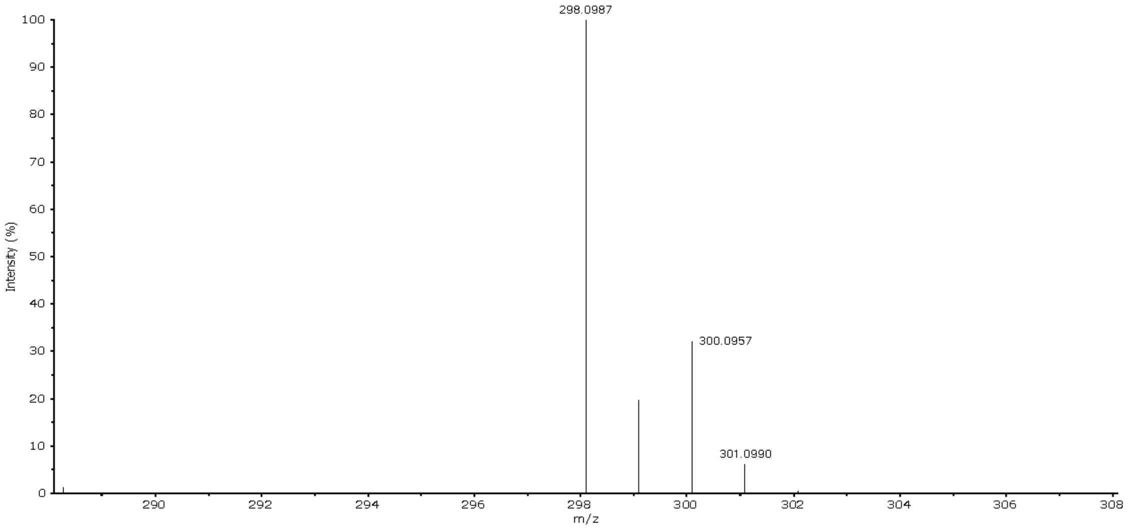

Theoretical Spectrum for C18H17ClNO, Minimum Abundance 0.01%

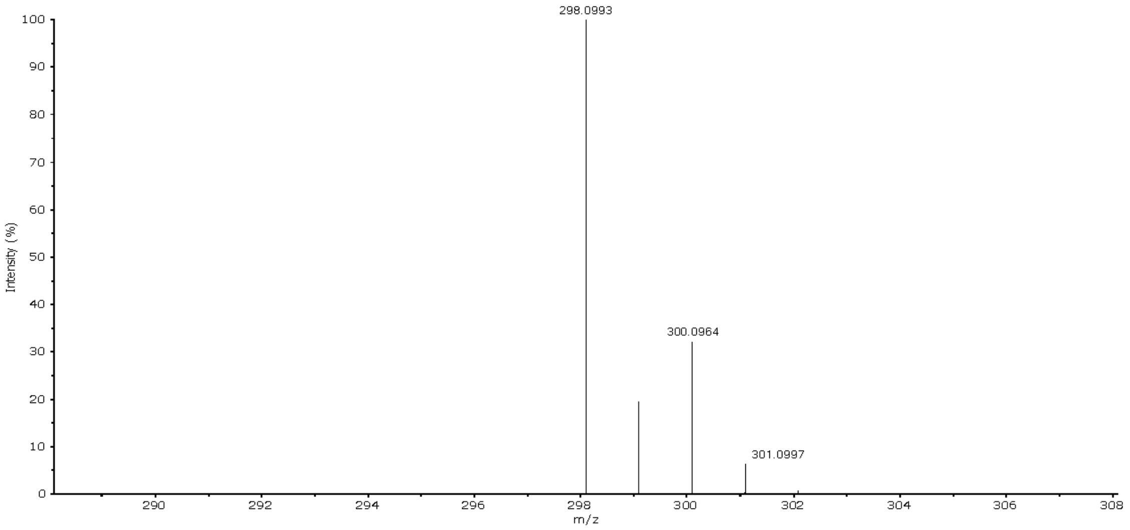

3-(3-(4-chloronaphthalen-1-yl)propyl)-5-methoxypyridine 1-oxide (65)

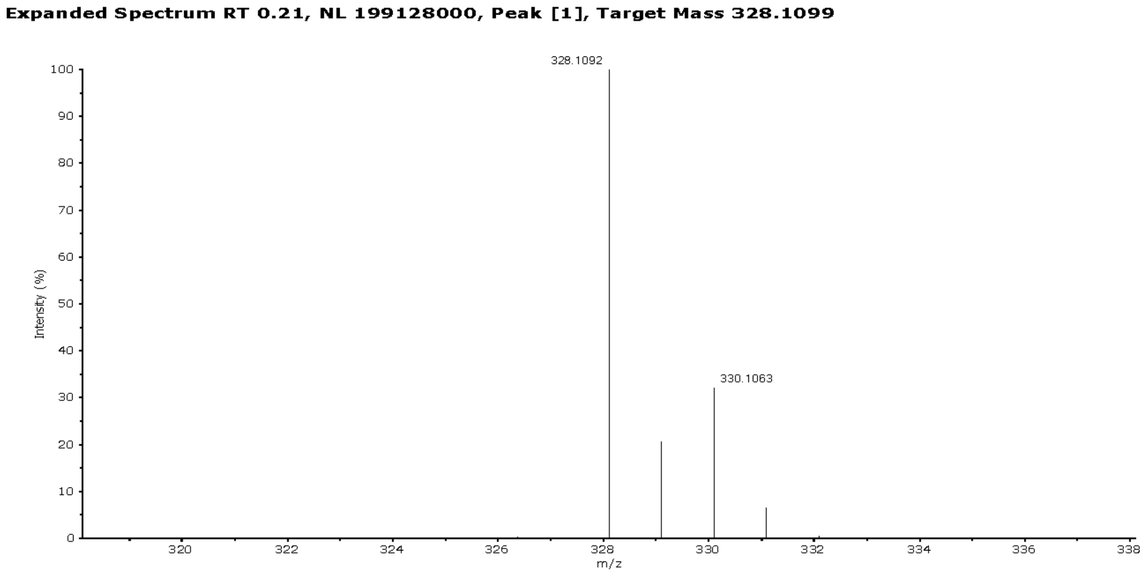

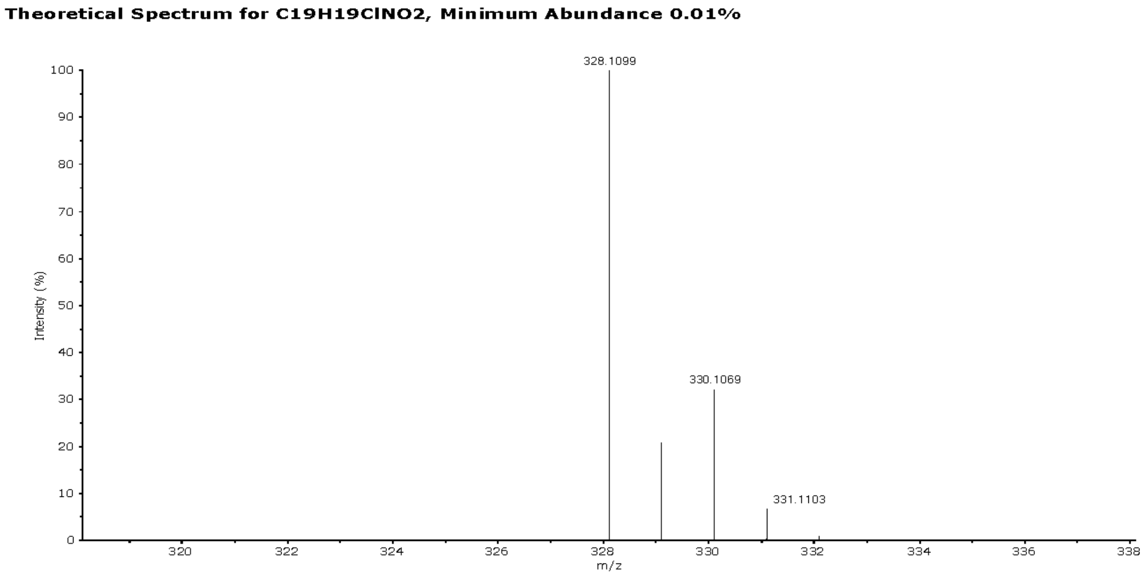

3-(3-(4-chloronaphthalen-1-yl)propyl)-5-hydroxypyridine 1-oxide (66)

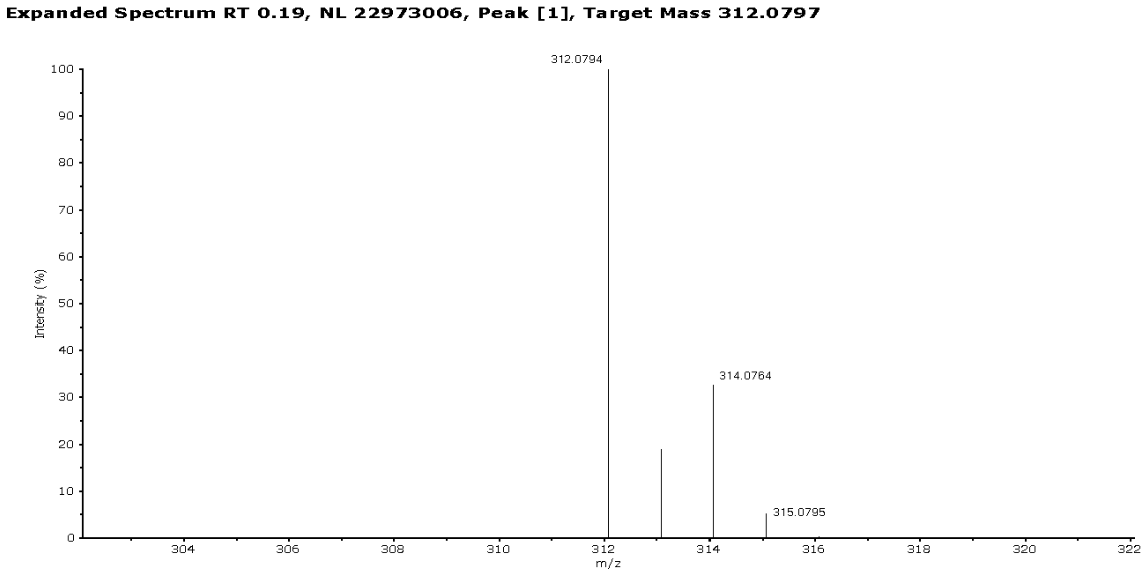

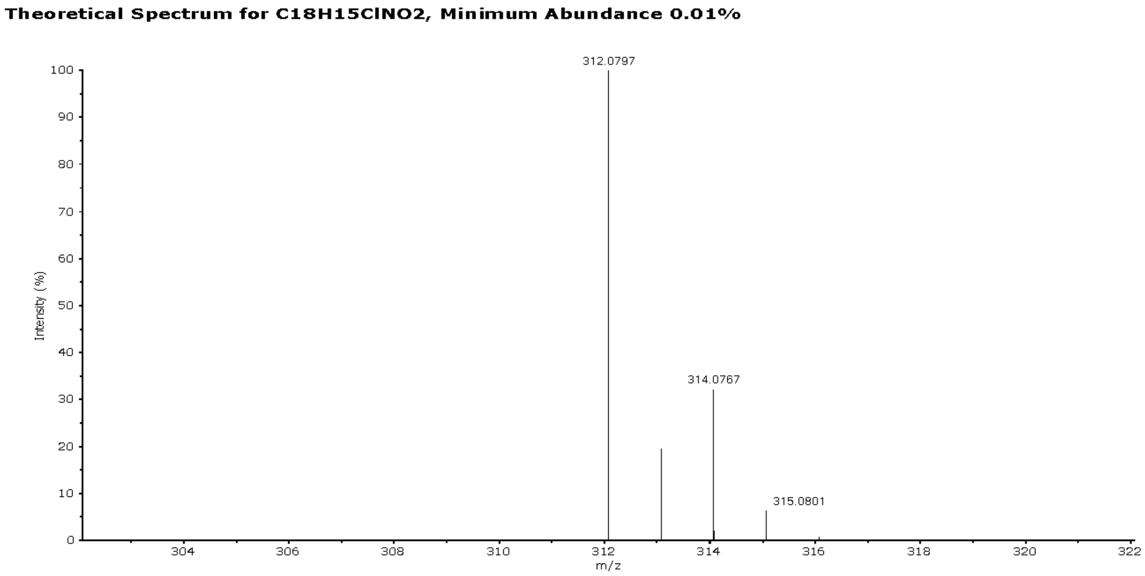

3-(2-((4-chloronaphthalen-1-yl)oxy)ethyl)-5-fluoropyridine 1-oxide (67)

Expanded Spectrum RT 0.16, NL 119632992, Peak [1], Target Mass 318.0692

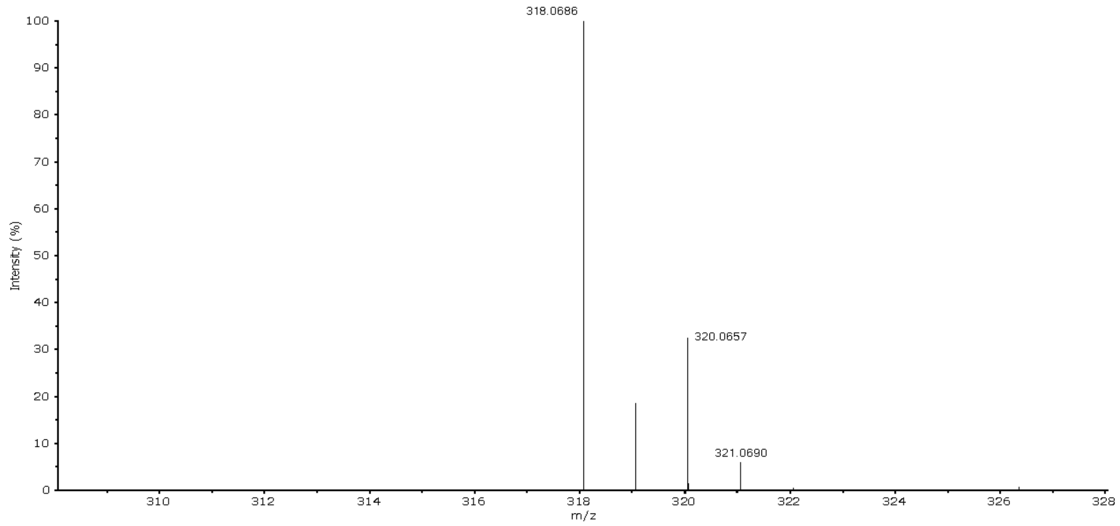

Theoretical Spectrum for C17H14ClFNO2, Minimum Abundance 0.01%

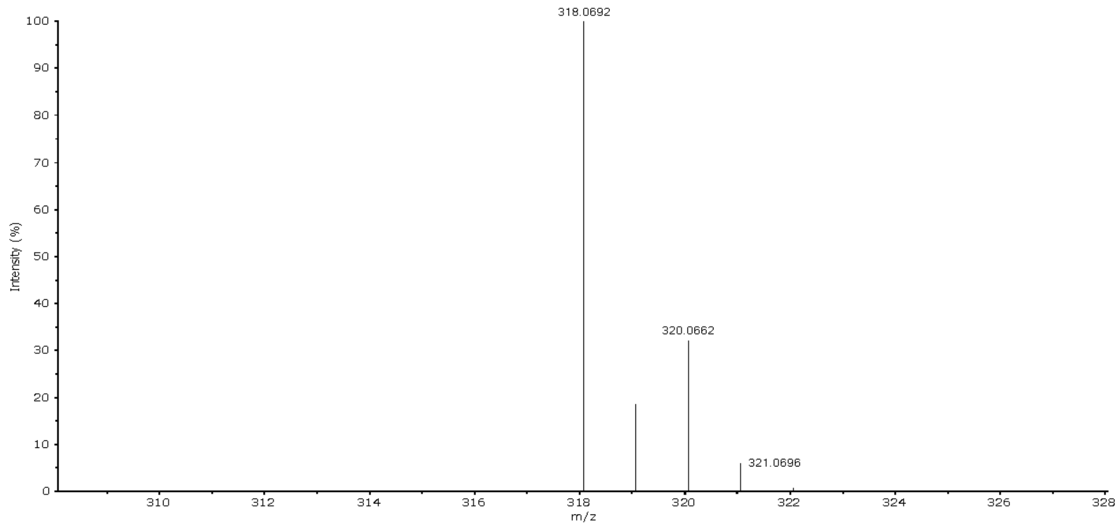

3-(3-(3,5-bis(trifluoromethyl)phenyl)propyl)-5-hydroxypyridine 1-oxide (68)

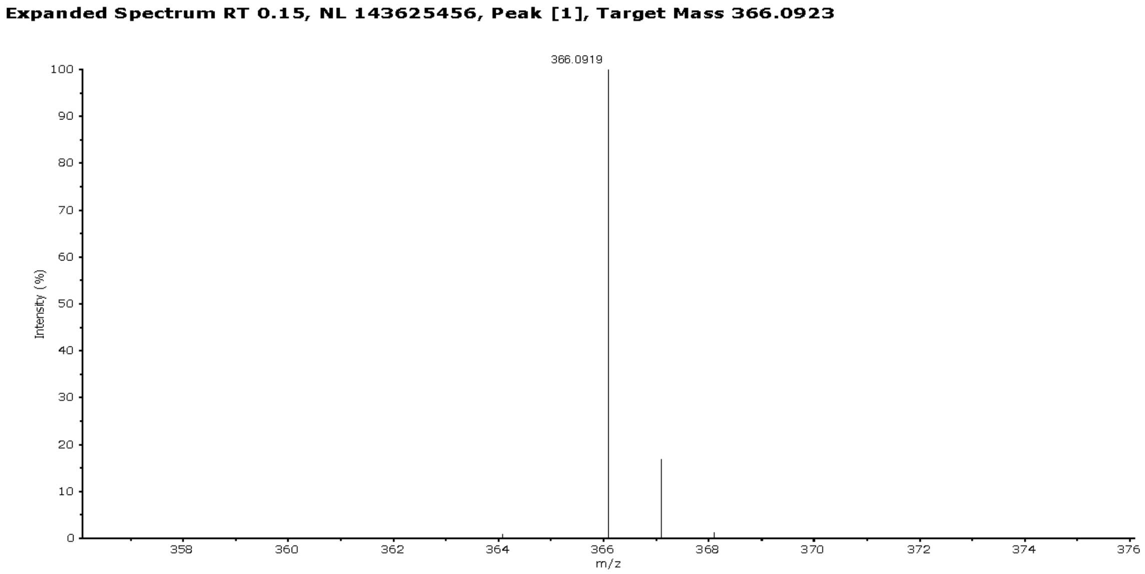

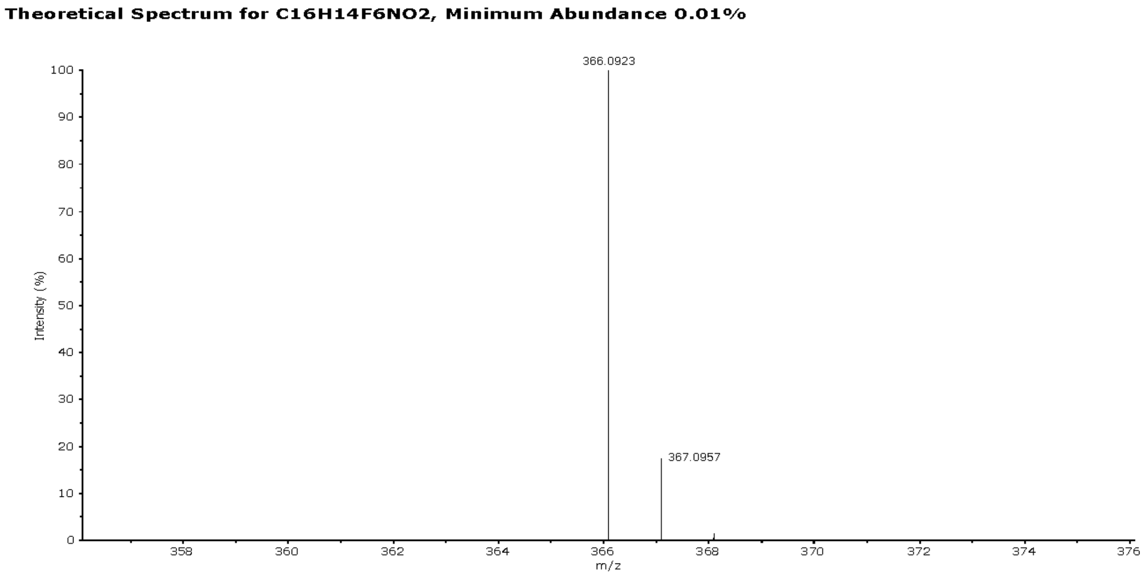

3-(3-(4-chloro-3-(trifluoromethyl)phenyl)propyl)-5-hydroxypyridine 1-oxide (69)

Expanded Spectrum RT 0.17, NL 111246184, Peak [1], Target Mass 332.0660

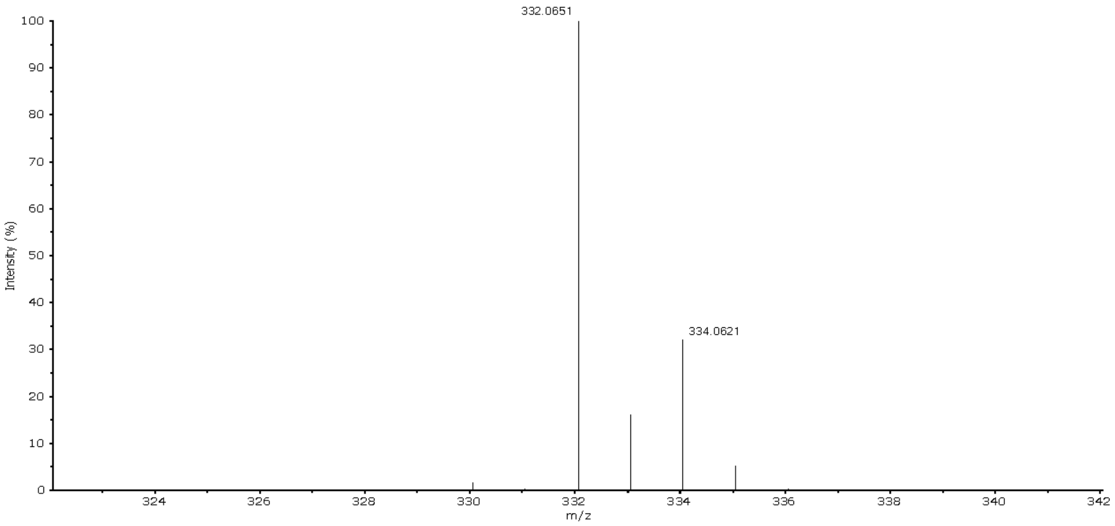

Theoretical Spectrum for C15H14ClF3NO2, Minimum Abundance 0.01%

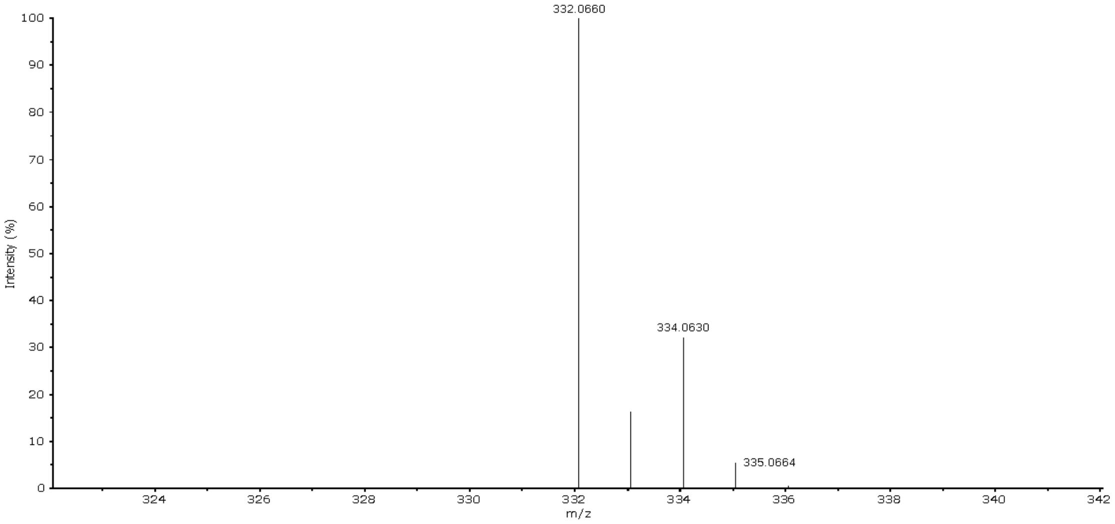

Supplement: Supplementary file 1 — jm3c00951_si_001.pdf [file jm3c00951_si_001.pdf]
